# Charting the Chemical and Mechanistic Scope of Light-Triggered Protein Ligation

**DOI:** 10.1021/jacsau.1c00530

**Published:** 2022-02-08

**Authors:** Daniel F. Earley, Amaury Guillou, Simon Klingler, Rachael Fay, Melanie Gut, Faustine d’Orchymont, Shamisa Behmaneshfar, Linus Reichert, Jason P. Holland

**Affiliations:** Department of Chemistry, University of Zurich, Winterthurerstrasse 190, Zurich CH-8057, Switzerland

**Keywords:** photochemistry, protein ligation, radiochemistry, zirconium, density functional theory, mechanisms

## Abstract

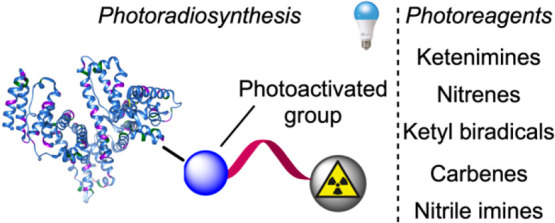

The creation of discrete, covalent bonds between a protein and a functional molecule like a drug, fluorophore, or radiolabeled complex is essential for making state-of-the-art tools that find applications in basic science and clinical medicine. Photochemistry offers a unique set of reactive groups that hold potential for the synthesis of protein conjugates. Previous studies have demonstrated that photoactivatable desferrioxamine B (DFO) derivatives featuring a para-substituted aryl azide (ArN_3_) can be used to produce viable zirconium-89-radiolabeled monoclonal antibodies (^89^Zr-mAbs) for applications in noninvasive diagnostic positron emission tomography (PET) imaging of cancers. Here, we report on the synthesis, ^89^Zr-radiochemistry, and light-triggered photoradiosynthesis of ^89^Zr-labeled human serum albumin (HSA) using a series of 14 different photoactivatable DFO derivatives. The photoactive groups explore a range of substituted, and isomeric ArN_3_ reagents, as well as derivatives of benzophenone, a para-substituted trifluoromethyl phenyl diazirine, and a tetrazole species. For the compounds studied, efficient photochemical activation occurs inside the UVA-to-visible region of the electromagnetic spectrum (∼365–450 nm) and the photochemical reactions with HSA in water were complete within 15 min under ambient conditions. Under standardized experimental conditions, photoradiosynthesis with compounds **1**–**14** produced the corresponding ^89^ZrDFO-PEG_3_-HSA conjugates with decay-corrected isolated radiochemical yields between 18.1 ± 1.8% and 62.3 ± 3.6%. Extensive density functional theory (DFT) calculations were used to explore the reaction mechanisms and chemoselectivity of the light-induced bimolecular conjugation of compounds **1**–**14** to protein. The photoactivatable DFO-derivatives operate by at least five distinct mechanisms, each producing a different type of bioconjugate bond. Overall, the experimental and computational work presented here confirms that photochemistry is a viable option for making diverse, functionalized protein conjugates.

## Introduction

Chemical modification of proteins plays an essential role in the development of many state-of-the-art diagnostic and therapeutic agents used in fundamental science and clinical medicine.^1−3^ For instance, the high affinity and specificity of monoclonal antibodies (mAbs) provides the basis of molecularly targeted antibody-drug conjugates (ADCs) via functionalization with a cargo molecule.^4,5^ The cargo can be a cytotoxic drug for therapeutic intervention, a fluorophore for optical imaging, or a radioactive complex for applications in positron-emission tomography (PET) imaging or radioimmunotherapy. Formation of a chemically and metabolically stable covalent bond between the protein and the exogenous cargo molecule is central to many successful bioconjugation strategies. Traditional protein bioconjugation methods usually exploit the native reactivity of amino acid side chain groups to create new linkages. Widely used methods include the reaction of free sulfhydryl groups of cysteine (Cys) residues on proteins with reagents bearing Michael acceptors, or the use of electrophilic reagents including activated esters or isothiocyanates that react with the nucleophilic ε-NH_2_ group on lysine (Lys) residues to form biocompatible, and generally stable, amide or thiourea bonds.^5^ Conjugation chemistries based on these routes are highly successful in the clinic, but there is growing appreciation that the nature of the bioconjugate bond can influence the pharmacokinetics and overall performance of the protein conjugate in vivo.

The last two decades have witnessed rapid growth in the use of bioorthogonal, site-specific labeling technologies to create stoichiometrically precise and regiospecific protein conjugates.^2,6,7^ The rationale that underpins the shift to bioorthogonal methods is that, when compared to randomly functionalized protein conjugates, monodispersed reagents will have reduced biochemical variability with congruent improvements in both target-to-background contrast and therapeutic indices. Many innovative and elegant “tag and modify” approaches^8^ show promise in the preclinical setting but are yet to have an impact in the clinic, where, at least in the context of radioimmunoconjugates, the classic technologies of amide and thiourea bond formation remain prevalent. Until recently, lysine labeling on protein was considered by many to be a random or stochastic process,^5^ but experimental evidence has counteracted this notion. The chemical reactivity of amino acid residues depends on the local environment within the native protein, and importantly, the pH of the reaction medium. The p*K*_a_ of the protonated ammonium ion on the side chain of lysine residues in proteins can vary dramatically, with p*K*_a_ values from ∼5 to >11.^9,10^ The identification of new reagents, and control over the reaction conditions, has led to site-specific lysine functionalization.^11−13^ Site-selective labeling of native histidine,^14^ arginine,^15^ and tyrosine^16^ groups has also been reported. As bioorthogonal chemistry continues to develop, it is possible that new, site-specific methods will eventually be translated to the clinic, which is particularly important for ADC technologies.^6^ Nevertheless, alternative methods that provide simple, fast, and reliable ways of creating a variety of new bioconjugate bonds are still required.

The use of photochemistry to produce covalent bonds to protein was originally introduced by Westheimer and co-workers in 1962^17^ and later developed into the concept of photoaffinity labeling (PAL).^18−21^ Since then, many different photoactivatable reagents^22^ have been reported, yet the basic concept and challenge remains the same: How can we control the extreme reactivity of photoinduced intermediates, which are often radical species with lifetimes in the microsecond to picosecond time scale, to create discrete covalent bonds to protein with high efficiency? In PAL, the photoactivation step is preceded by the reversible formation of a noncovalent preassociation complex between the target protein and a photoactivatable ligand.^23^ The photoactivatable ligand is designed to bind to a target epitope with high affinity and selectivity, displacing the position of equilibrium toward the complex. Subsequent photon absorption by the ligand chromophore generates the reactive intermediate in situ, effectively facilitating a pseudo-first-order reaction, and covalent capture by the protein. Other factors such as desolvation and shielding of the ligand from the influence of oxygen or other components of the aqueous-phase medium contribute to the success of PAL for target identification. However, the necessity of using a ligand that preassociates with a target protein at an established binding site is a fundamental limitation of PAL.

**Figure 1:**
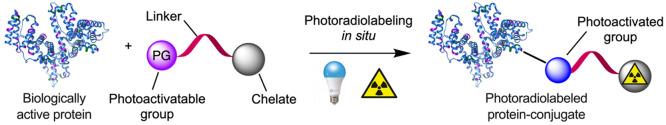
Overview of the one-pot, light-triggered bioconjugation and radiolabeling of biologically active proteins using a metal binding chelate functionalized with a photoactivatable group (PG).

Inspired by previous studies,^24−34^ we began exploring the use of light-triggered chemistry to access biochemically viable monoclonal antibody-based radiotracers for applications in PET imaging and radioimmunotherapy (Figure 1).^35−43^ The photoconjugation chemistry of labeling proteins using ArN_3_ derivatives is chemoselective for lysine residues^23^ but, presently, is not site-selective. We note that important works from Rousselot et al.^44^ in 1997, and more recently, MacMillan and co-workers,^16^ provide encouraging precedents that demonstrate site-specific phototriggered bioconjugation of proteins is possible. In this context, our long-term objective is to develop generalizable, and ultimately, chemo- and regio-specific photochemical methods for producing bioconjugate bonds to protein and to explore the influence of these new linkages on the pharmacokinetic performance of our radiolabeled protein-conjugates. Controlling the extreme reactivity of light-triggered chemistry and circumventing the requirement to form a preassociation complex between the cargo molecule and a generic protein are critical to achieving this objective. In other words, new reagents are required that facilitate bimolecular (i.e., true second-order) reactivity between the photogenerated intermediates and the protein.

Here, we report the synthesis, characterization, radiolabeling, and protein conjugation chemistry of 14 different photoactivatable compounds and their ^89^Zr-radiolabeled metal ion complexes, based on derivatization of the desferrioxamine B (DFO) chelate. Compounds synthesized and evaluated include aryl azide (ArN_3_), benzophenone (BP), diazirine (DA), and tetrazole (Tz) derivatives, which upon the absorption of a photon, create the highly reactive nitrene/ketenimine, ketyl biradical, triplet carbene, and electrophilic nitrile imine intermediates, respectively.^23^ For each compound, light-induced bimolecular protein conjugation between the ^89^Zr-labeled complexes and human serum albumin (HSA) was performed and the degree of labeling was assessed by measuring the decay-corrected radiochemical yields of the isolated ^89^Zr-labeled protein. Computational studies using density functional theory (DFT) provide detailed insights into the mechanisms of photochemical activation, thermodynamic parameters, and chemoselectivity of the subsequent secondary photochemical (dark) reactions that can occur between the photogenerated intermediates and different amino acid residues. Bioconjugation reactions between the 14 photoactivatable compounds (**1**–**14**) and HSA cover at least five distinct mechanisms that generate different types of bioconjugate bonds. Overall, this work expands the chemical and mechanistic scope of light-induced protein ligation and demonstrates that light-triggered chemistry^45^ is a widely applicable route to access high-value functionalized proteins with a range of covalent linkages that are not readily accessible using traditional reagents and methods.

## Methods

Full details on the methods, materials, synthesis, and characterization of all compounds are presented in the Supporting Information. Experimental NMR spectra and high-resolution electrospray ionization mass spectrometry (HR-ESI-MS) analyses are given in Figures S1–S234. Radiochemical and chromatographic data are also presented in Tables S1 and S2 and Figures S235–S250. Computational details and supporting data are presented in Tables S3–S11 and Figures S251–S272. Cartesian coordinates of the optimized structures are available from the corresponding author.

## Results and Discussion

### Synthesis

The synthetic route toward photoactivatable derivatives of the hexadentate chelate, DFO (compounds **1**–**14**) bearing a water-solubilizing *tris*-polyethylene glycol (PEG_3_) spacer is presented in Scheme 1.^40^ In general, DFO-PEG_3_ derivatives were synthesized in four linear steps starting from a carboxylic acid derivative of the photoactivatable unit. Final compounds were isolated by using either semipreparative reverse-phase high-performance liquid chromatography (HPLC) or C-18 flash chromatography in overall yields of up to 36%. All steps involving light-sensitive reagents were performed in the dark. Where applicable, final compounds were characterized by multinuclear ^1^H-, ^13^C{^1^H}-, and ^19^F{^1^H}-NMR spectroscopy, high-resolution mass spectrometry (Figures S1–S209), and HPLC. In addition to the DFO-PEG_3_ derivatives, we synthesized and tested the photoradiolabeling of an engineered scFv-Fc antibody fragment (onartuzumab) with six non-PEGylated photoactivatable DFO-derivatives (compounds **60**, **62**, **63**, and **65**–**67**, Figures S210–S234). Successful protein conjugation was observed (Figure S235) but the limited aqueous-phase solubility of most of these compounds made it difficult to obtain reproducible starting conditions for the photoradiochemistry. Therefore, the following work focuses on the use of the more soluble DFO-PEG_3_-derivatives (**1**–**14**).

**Scheme 1:**
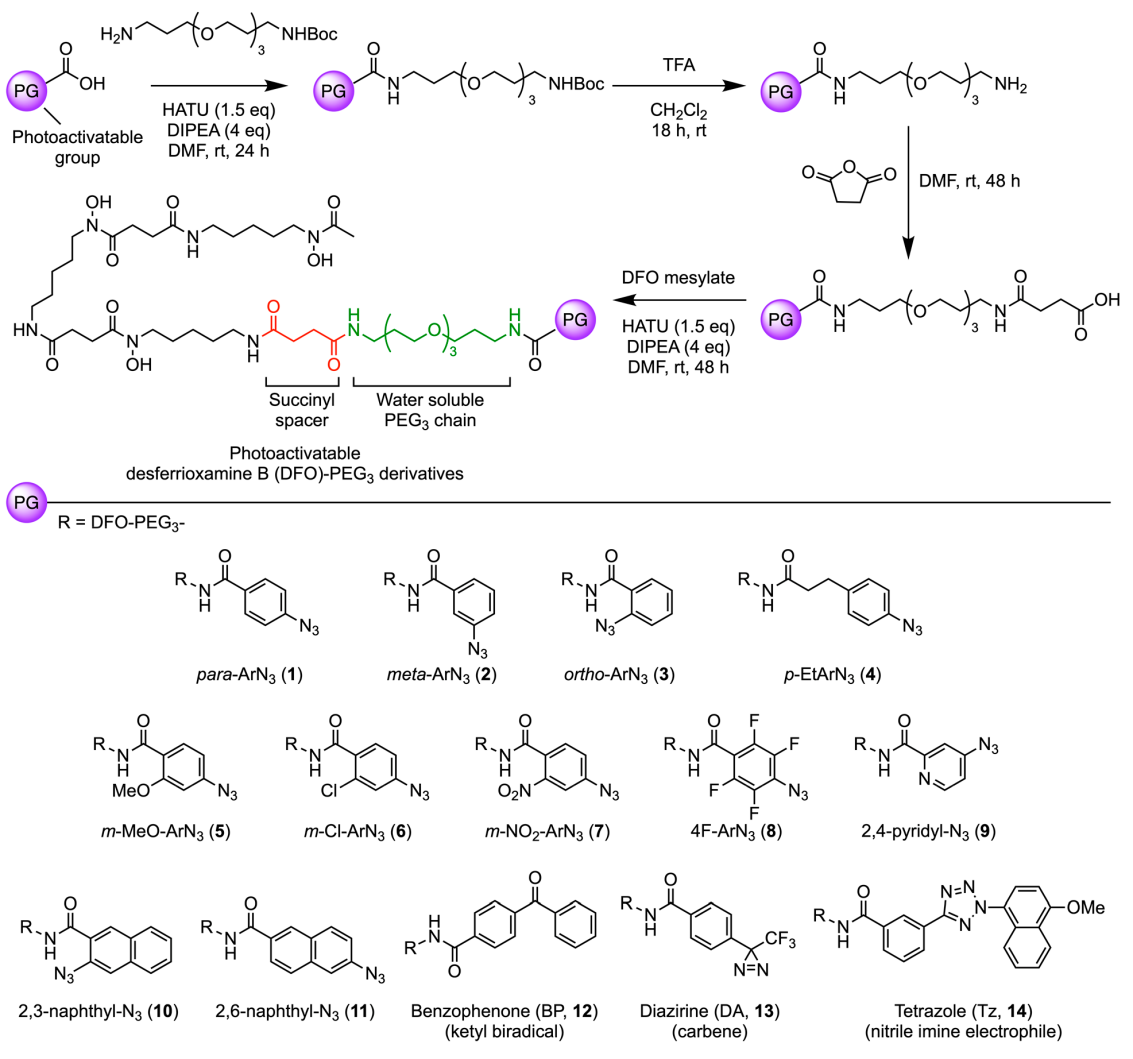
General Route for the Synthesis of Photoactivatable DFO-PEG_3_ Derivatives **1**–**14**

After isolating the DFO-PEG_3_-derivatives (**1**–**14**), the corresponding nonradioactive ^nat^Zr^4+^ metal ion complexes (^nat^Zr-**1**^+^ to ^nat^Zr-**14**^+^) were prepared and characterized by analytical reverse-phase HPLC and HR-ESI-MS (Figure S236A and Table S1). These nonradioactive ^nat^Zr complexes were used as standards to characterize and confirm the successful radiosynthesis of the equivalent ^89^Zr-radiolabeled complexes (^89^Zr-**1**^+^ to ^89^Zr-**14**^+^) by comparison of the retention times from analytical reverse-phase HPLC measurements. Starting from a preneutralized stock solution of [^89^Zr][Zr(C_2_O_4_)_4_]^4–^ (also known as ^89^Zr-oxalate), quantitative ^89^Zr-radiolabeling of compounds **1**–**14** was observed in aqueous solution (pH8.0–8.5) in <10 min at room temperature. The ^89^Zr-radiolabeling of these small DFO-PEG_3_ derivatives is complete in a few seconds but to ensure full conversion, the reactions were run under standardized conditions for 10 min. Radiochemical conversions (RCCs) of >95% for each of the ^89^Zr complexes (^89^Zr-**1**^+^ to ^89^Zr-**14**^+^) were confirmed by both radio-instant thin-layer chromatography (radio-iTLC; 50 mM DTPA eluent, pH 7.4) and radio-HPLC (Figure S236B).

### Photoradiosynthesis of ^89^Zr-Labeled Human Serum Albumin (HSA) Conjugates

**Scheme 2:**
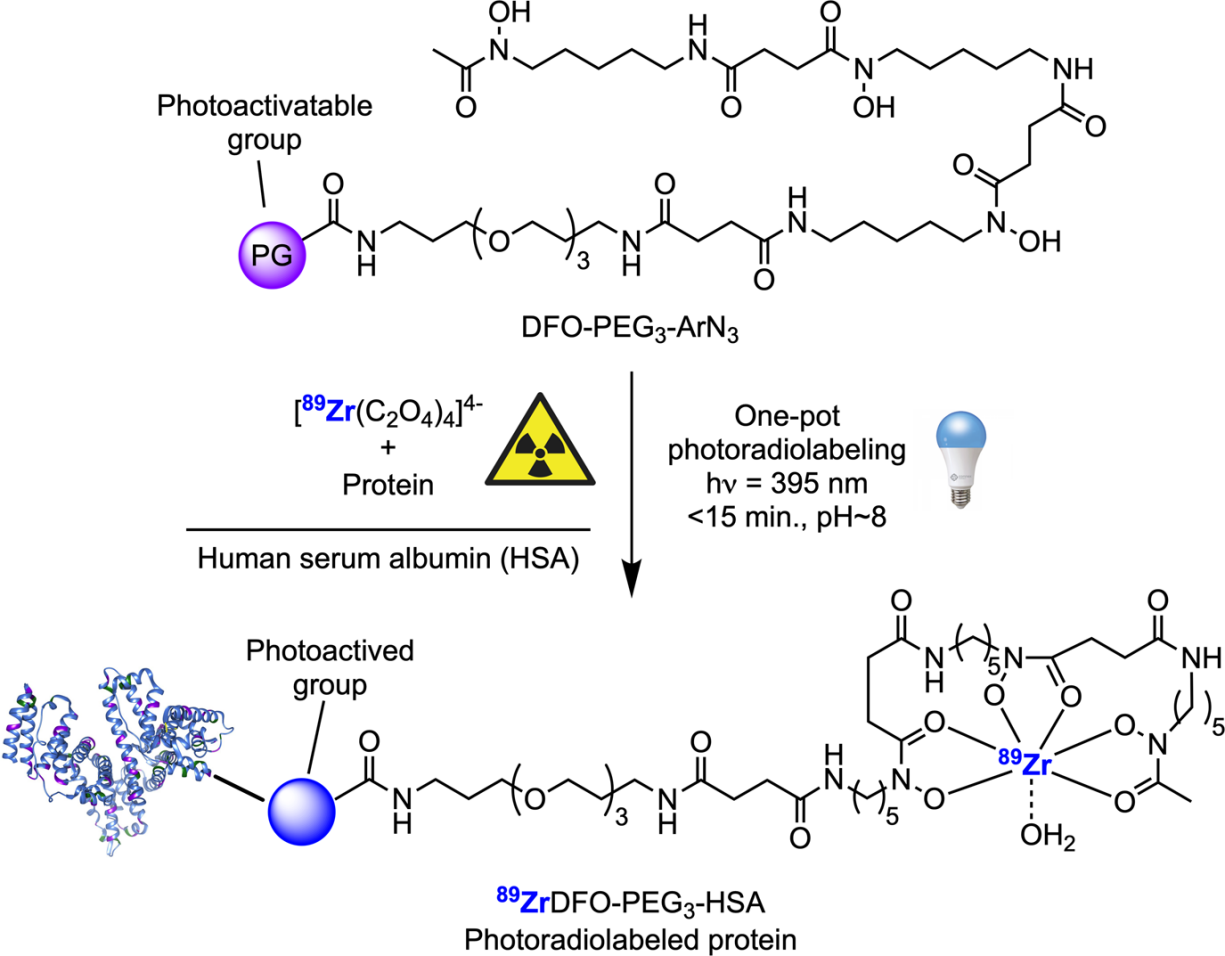
Photoradiosynthesis of ^89^Zr-Labeled HSA Protein Conjugates Using Photoactivatable DFO-PEG_3_-Derivatives

**Figure 2:**
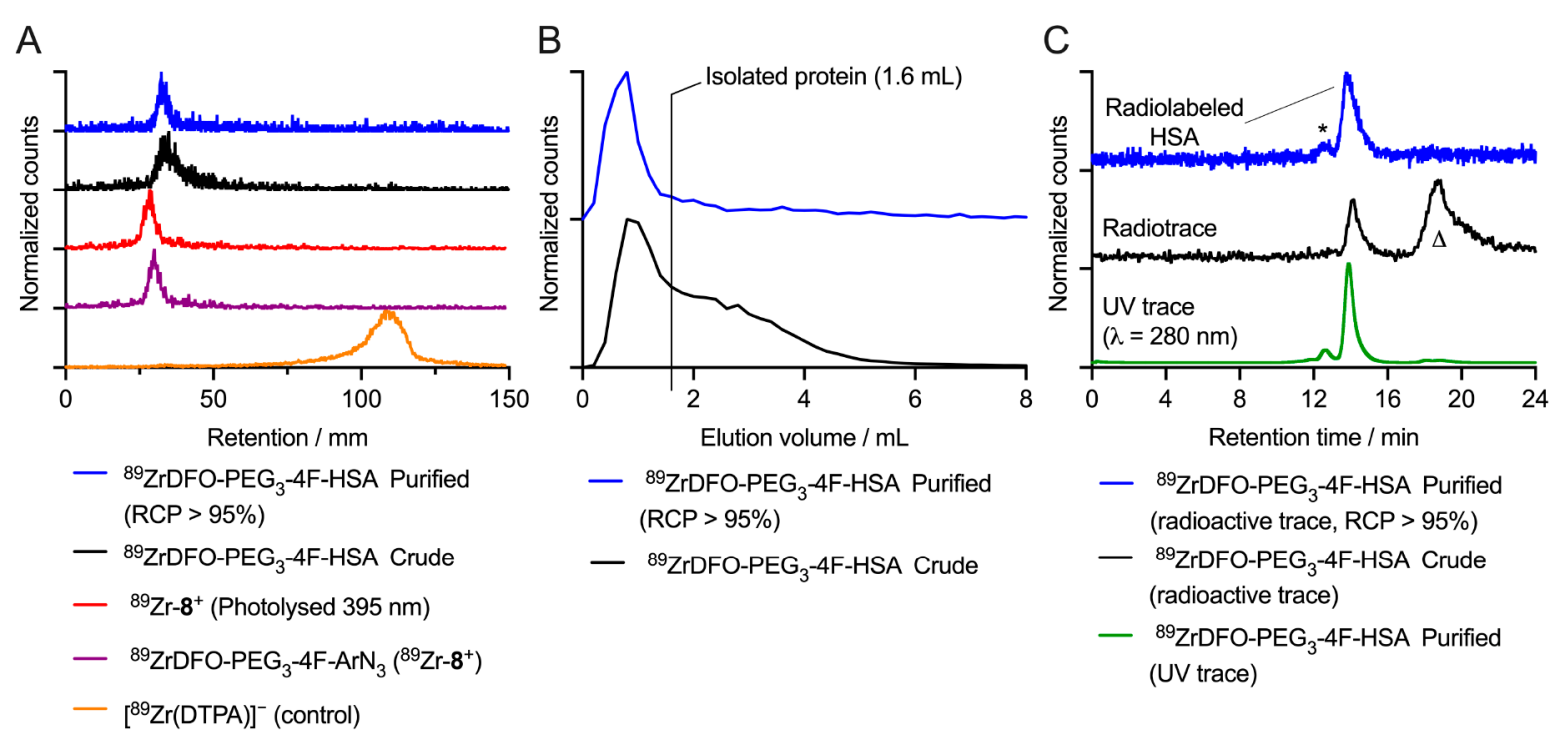
Representative characterization data for the radiochemical synthesis of ^89^ZrDFO-PEG_3_-4F-HSA starting from DFO-PEG_3_-2,3,5,6-tetrafluoro-*para*-ArN_3_ (4F-ArN_3_, **8**) and [^89^Zr][Zr(C_2_O_4_)_4_]^4–^. (A) Radio-iTLC chromatograms showing the retention of ^89^Zr-radioactivity at the baseline (*R*_f_ = 0.0–0.1) for the crude (black) and purified (blue) samples of ^89^ZrDFO-PEG_3_-4F-HSA, and for ^89^Zr-**8**^+^ before (purple) and after (red) photolysis. The chromatogram of [^89^Zr(DTPA)]^−^ is presented as a control (orange trace) showing elution of ^89^Zr^4+^ ions at the solvent front (*R*_f_ = 0.9–1.0) in the absence of a DFO-based molecule. (B) Analytical PD-10-SEC elution profiles of the crude (black) and purified (blue) samples of ^89^ZrDFO-PEG_3_-4F-HSA. (C) SEC-HPLC chromatograms of the crude (black) and purified ^89^ZrDFO-PEG_3_-4F-HSA product (blue). For comparison, elution of the protein component in the purified ^89^ZrDFO-PEG_3_-4F-HSA product monitored by electronic absorption at 280 nm is shown (green trace). Note: * = aggregated protein; Δ = radiolabeled small-molecule byproducts that are removed during purification. Equivalent data for the other compounds are presented in Figures S237–S250.

Next, we performed the one-pot photoradiosynthesis of ^89^ZrDFO-PEG_3_-labeled human serum albumin (^89^ZrDFO-PEG_3_-HSA) starting from compounds **1**–**14** and aliquots from separate stock solutions of ^89^Zr-oxalate and HSA (Scheme 2). HSA was used as a model globular protein to facilitate the development of standardized reaction conditions and because clinical-grade antibodies like trastuzumab or onartuzumab are prohibitively expensive. Photochemical reactions are sensitive to the experimental geometry.^46^ Therefore, we used a standardized experimental setup for all photoradiosynthesis reactions.^41^ The setup involved the direct, top-down irradiation of a reaction mixture that was stirred gently in a transparent glass vial. The reaction mixture contained an initial HSA protein to DFO chelate molar ratio of 1:1.05, a fixed amount of protein (33.5 nmol, to give a final concentration of 224 μM), and ∼7.9 MBq of ^89^Zr-oxalate.^41^ The pH of all reactions was controlled and adjusted to pH 8.0–8.4 with aliquots of Na_2_CO_3_, and the total reaction volume was 150 μL. First, the complexes ^89^Zr-**1**^+^ to ^89^Zr-**14**^+^ were prepared in situ by adding an appropriate aliquot of the preneutralized [^89^Zr][Zr(C_2_O_4_)_4_]^4–^ stock solution to a stirred solution of the ligand (**1**–**14**). Quantitative complexation was confirmed by radio-iTLC before adding the aliquot of HSA. Then the pH was checked, and the reaction volume set to 150 μL by using metal-ion-free (Chelex-treated) water. The reaction mixture was irradiated at 395 nm (100% LED intensity equivalent to ∼355 mW) for 15 min at room temperature (23 ± 1 °C). The reaction temperature did not change during the irradiation. After irradiation, aliquots (100 μL) of the crude reaction mixtures were purified by manual, preparative size-exclusion chromatography (SEC) using PD-10 columns (GE Healthcare) that contain Sephadex G-25 medium. After loading the crude reaction aliquot onto the PD-10 column and discarding the dead volume, we eluted the column with sterile PBS and collected high-purity samples of ^89^ZrDFO-PEG_3_-HSA (RCP > 90%) in the high-molecular-weight fraction (total elution volume = 1.6 mL). Aliquots of the crude reaction mixtures that were retained after the photoradiosynthesis, and aliquots of the purified ^89^ZrDFO-PEG_3_-HSA samples, were analyzed by using three different chromatographic methods including analytical radio-iTLC, analytical PD-10-SEC, and fully automated SEC-HPLC (Figure 2 and Figures S237–S250). All photoradiosynthesis reactions were performed in at least triplicate using independent experiments, and (except for compound **14**) by at least two different senior scientists to avoid systematic, user-dependent errors.

Representative experimental data obtained from the synthesis of ^89^ZrDFO-PEG_3_-4F-HSA, and starting from the DFO-PEG_3_-2,3,5,6-tetrafluoro-*para*-ArN_3_ compound (4F-ArN_3_; **8**), are presented in Figure 2. As expected, radio-iTLC analysis confirmed that ^89^Zr-radiolabeling of **8** gave ^89^Zr-**8**^+^ (Figure 2A, purple trace) in quantitative radiochemical conversion (RCC) and was determined by measuring the retention of the activity at the baseline (*R*_f_ = 0.0–0.1) on silica-gel impregnated glass-fiber strips that were eluted with diethylenetriamine pentaacetate (DTPA; 50 mM, pH7.4). As a control, the elution profile of “free” ^89^Zr^4+^ ions, which produce the [^89^Zr(DTPA)]^−^ complex in the radio-iTLC conditions and elutes at the solvent front (*R*_f_ = 0.9–1.0), is shown by the orange trace (Figure 2A). Control reactions in which ^89^Zr-**8**^+^ was photolyzed in the absence of HSA confirmed that the ^89^Zr-metal ion remained bound to the DFO chelate even after exposure to intense UVA light (Figure 2A, red trace). After photoradiosynthesis in the presence of HSA, and preparative isolation of the ^89^ZrDFO-PEG_3_-4F-HSA, radio-iTLC analysis of both the crude reaction mixture and the purified product (Figure 2A, black and blue traces, respectively) confirmed that the ^89^Zr-activity was bound to the protein. Analytical PD-10-SEC elution profiles of the crude (black) and purified (blue) samples of ^89^ZrDFO-PEG_3_-4F-HSA are presented in Figure 2B confirming that the ^89^Zr-radioactivity coelutes in the high-molecular-weight protein fraction. Accurate analysis of the protein labeled fraction in the crude samples, and quantification of the radiochemical purity (RCP) of the isolated product was obtained by using analytical SEC-HPLC monitored by electronic absorption at 280 nm and by radioactivity detection (Figure 2C). Under SEC-HPLC conditions, the HSA protein eluted with a major peak at 14.0 min corresponding to the monomeric protein, and a small peak at shorter retention time (12.9 min) corresponding to the dimeric (or potentially multimeric) protein aggregate (Figure 2C, green trace). ^89^Zr-radiolabeling of the protein was observed in the SEC-HPLC chromatogram of the crude reaction mixture (Figure 2C, black trace). Successful purification of ^89^ZrDFO-PEG_3_-4F-HSA from the small-molecule byproducts (indicated by the Δ symbol), which are likely associated with hydrolysis adducts obtained after photolysis of ^89^Zr-**8**^+^ in aqueous media, was confirmed by SEC-HPLC (Figure 2C, blue trace).^42^ Overall, ^89^ZrDFO-PEG_3_-4F-HSA was isolated and formulated in sterile PBS with a decay-corrected radiochemical yield (RCY) of 40.3 ± 1.4% (*n* = 3; note: RCY values correspond to the mean with errors reported as one standard deviation), and with an RCP of >95%. The amount of ^89^Zr-activity associated with the small HSA protein aggregate peak (indicated by the * symbol in Figure 2C) was <5% and no additional increase in protein aggregation was observed when compared with the initial HSA stock solution. Equivalent experimental data on the photoradiosynthesis of ^89^ZrDFO-PEG_3_-HSA conjugates produced by using the other photoactivatable compounds (**1**–**7** and **9**–**14**) are presented in Figures S237–S250.

**Figure 3:**
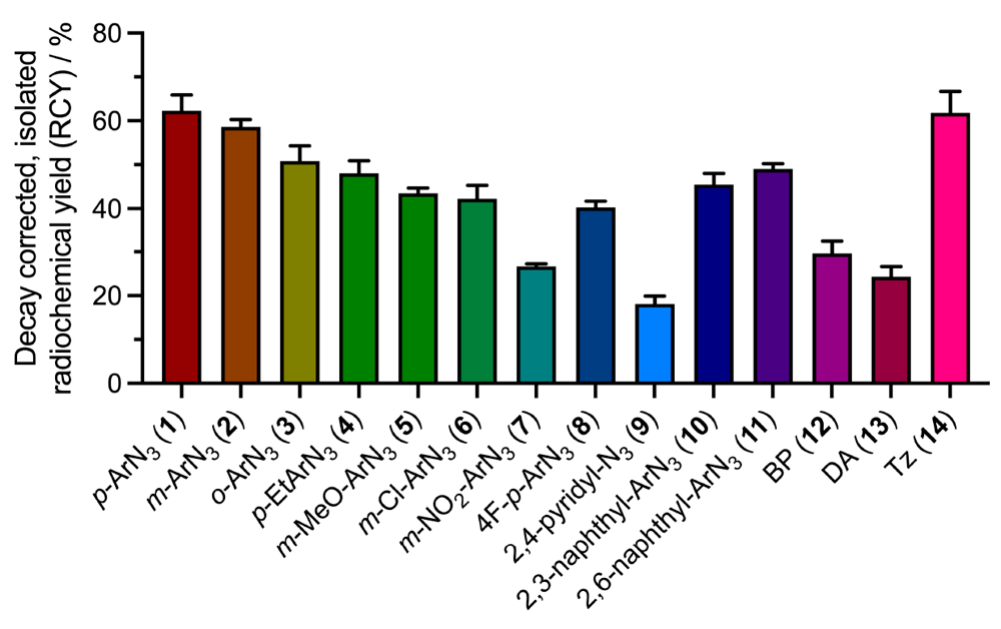
Bar chart showing the decay-corrected, isolated radiochemical yield (RCY, expressed as a percentage with respect to the initial amount of [^89^Zr][Zr(C_2_O_4_)_4_]^4–^ starting reagent added to each reaction) of the 14 different ^89^ZrDFO-PEG_3_-HSA products synthesized by using photoactivatable chelates **1**–**14**. Error bars represent one standard deviation about the mean calculated from independent replicates (compound **1**, *n* = 5; compounds **2**–**14**, *n* = 3).

A bar chart showing the experimental decay-corrected RCYs of the isolated ^89^ZrDFO-PEG_3_-HSA conjugates produced from compounds **1**–**14** is presented in Figure 3 (for numerical data, see Table S2). Remarkably, under the same experimental conditions, all photoactivatable compounds produced new ^89^Zr-radiolabeled HSA conjugates with RCP > 95%. After correcting the isolated yields for minor variations in the RCP of the isolated products (measured by integration of the SEC-HPLC data), the decay-corrected RCYs showed a variation across the series of compounds. The two most successful reagents that gave the highest RCYs were DFO-PEG_3_-*para*-ArN_3_ derivative (**1**; RCY = 62.3 ± 3.6%, *n* = 5), and the tetrazole derivative DFO-PEG_3_-Tz (**14**; RCY = 61.8 ± 4.9%, *n* = 3). These reagents are known to produce powerful electrophiles (a ketenimine species for **1**, and a nitrile imine for **14**) after photoinduced activation and elimination of dinitrogen (vide infra). The lowest conjugation yield was obtained for the DFO-PEG_3_-2,4-pyridyl-N_3_ derivative (**9**), which gave an isolated decay-corrected RCY of 18.1 ± 1.8% (*n* = 3) and likely reacts via an open-shell nitrene or nitrenium ion in high-polarity solvents.^47^ Notably, both the benzophenone derivative (DFO-PEG_3_-BP, **12**), which reacts via a triplet ketyl biradical species,^48^ and the diazirine derivative (DFO-PEG_3_-DA, **13**), which generates a triplet carbene,^22^ also gave the corresponding ^89^Zr-labeled HSA-conjugates in RCYs of 29.6 ± 2.8% (*n* = 3) and 24.3 ± 2.3% (*n* = 3), respectively. Mechanistic features of the photoinduced reactivity of compounds **1**–**14**, the competition between different reaction channels, and the chemoselectivity of the various photogenerated intermediates toward different reactive groups found on proteins are explored in the following sections.

Overall, our experimental results confirm that photoactivatable chelates functionalized with an unmodified ArN_3_ group (**1**–**4**) or tetrazine-species like compound **14** give the highest bioconjugate yields with protein under conditions that are applicable to the production of protein-based radiotracers for PET imaging. Limitations of the photochemical approach include the incomplete radiochemical conversion as indicated by decay-corrected RCYs that peak at ∼60%. Efficient separation of the radiolabeled protein component from the small-molecule byproducts is likely to require improvements in the size-exclusion methods that are commonly used. To this end, our recent work indicates that significant improvements can be made on increasing the RCP of the products by switching from classic size-exclusion using PD-10 desalting columns to custom-made gel filtration columns filled with Sephadex G-100, which provides a higher separation efficiency for the purification of high-molecular-weight proteins >100 kDa.^49^ At present, several thermally mediated protein conjugation methods offer the advantage of site-specific labeling, a feature that is important in the design of antibody–drug conjugates.^2,6,7^ However, as noted in the introduction, translation of these site-specific methods is nontrivial. We believe that with further experimental development, photochemical methods can also be developed for site-specific labeling and clinical applications.^16,44^

#### Computational Studies on the Mechanisms and Chemoselectivity of Light-Induced Protein Ligation with Model Compounds **1**–**14**

After establishing the empirical basis of light-induced protein conjugation by using different DFO-PEG_3_ derivatives, we next used density functional theory (DFT) calculations to explore the mechanisms of photoactivation and subsequent secondary (dark) reactivity of compounds **1**–**14** with biologically relevant model nucleophiles. Our aims were to explore the thermodynamic landscape and competition different reaction channels, as well as the potential chemoselectivity of various photoinduced intermediates toward common reactive groups found in HSA and other proteins. The separate sections below report computational studies on the reactivity of the different model compounds that represent the ArN_3_ derivatives (**1**–**11**), benzophenone (BP; **12**), diazirine (DA; **13**), and tetrazole (Tz; **14**). All calculations were performed by using DFT implemented in *Gaussian16*. To simplify the calculations, we truncated the structures of compounds **1**–**14** by replacing the DFO-PEG_3_ unit with a methyl group attached to the photoactivatable moiety via an *N*-methylamide. The amide group was retained in the model structures to ensure that the electronic properties of the aryl rings were as close as possible to the experimental molecules. The calculated energetics were obtained from structures that were optimized without symmetry constraints by using the PW6B95/6-311++G(d,p) methodology. All calculations employed intrinsic solvation via the default polarizable continuum model (PCM) using water as the solvent. Full details on the computation methods are given in the Supporting Information.

### Overview of Aryl Nitrene Chemistry

**Scheme 3:**
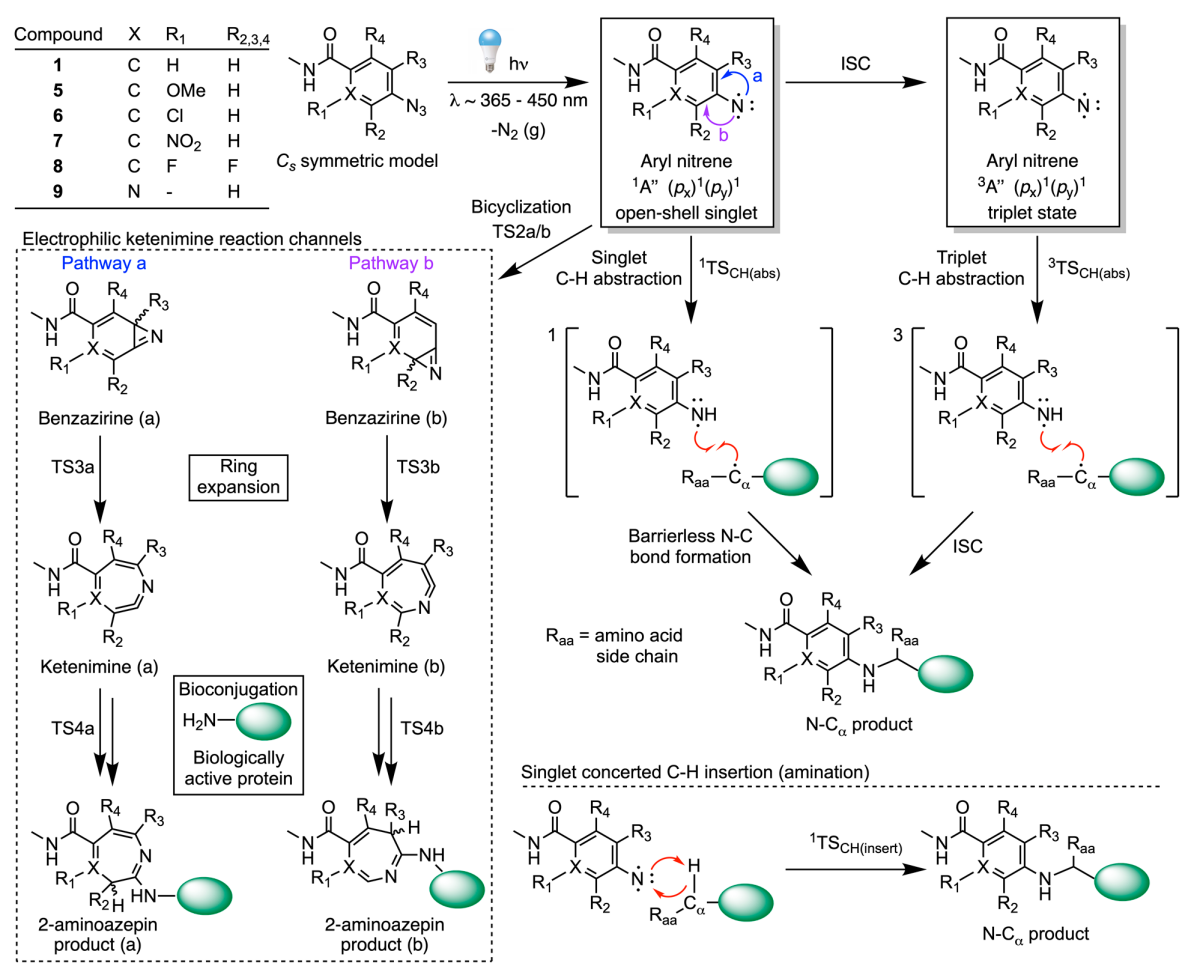
Mechanistic Pathways Following the Photoactivation of an Aryl Azide and Subsequent Reaction with a Biologically Active Protein

Fleet et al. were the first to report the use of an ArN_3_ derivative (in their case, a *meta*-nitro-substituted ArN_3_) for PAL of proteins in 1969.^50^ Prior to this, the photochemically induced ring expansion of phenyl azide (PhN_3_) was reported by Doering and Odum in 1966.^51^ An overview of the reaction pathways that are accessible to the aryl nitrene after photon absorption and dinitrogen elimination is presented in Scheme 3. The established photochemistry of phenylnitrene versus phenylcarbene was summarized by Platz.^52^ In contrast to reagents that generate aryl carbenes, the synthetic utility of aryl nitrenes, made by either thermal^53^ or photochemical activation of the corresponding ArN_3_, have long been considered as “bad” reagents in organic chemistry.^52^ The origin of this, arguably unfair, characterization resides in the fact that aryl nitrenes exhibit extremely short lifetimes in both gas and solution phase. Aryl nitrenes undergo vibrational deactivation and ring expansion, which in solution leads to extremely short lifetimes in the range of 0.1–13.0 ns.^54−56^ This short lifetime means that aryl nitrenes typically undergo a unimolecular rearrangement that is faster than essentially all bimolecular processes that would be relevant in the formation of a covalent bond with protein.^52^ Unlike aryl carbenes, whose chemistry is dominated by the triplet species, the larger singlet-to-triplet energy gap in aryl nitrenes decreases the rate of intersystem crossing (ISC)^54^ and favors reactivity via an open-shell singlet species. Under ambient conditions, the singlet aryl nitrene has a low energetic barrier toward intramolecular rearrangement, which leads to the formation of an intermediate seven-membered ketenimine species, which is a powerful electrophile. The rate limiting step on the ketenimine pathway is assigned to N-insertion into the aromatic ring of the aryl nitrene to give a bicyclic benzazirine intermediate via the transition state, TS2 (Scheme 3). Ring expansion via TS3, which involves breaking of the C–C bond, produces a ketenimine intermediate that has been characterized by matrix isolation methods using infrared and electronic absorption spectroscopy.^55−57^ Nucleophilic attack (TS4; Scheme 3) at the ketenimine, and proton rearrangement produces a 2-substituted azepin ring. Depending on the substitution pattern and symmetry of the initial aryl azide, two alternative reaction channels (pathways a and b; Scheme 3) can occur giving rise to possible geometric isomers for the 2-substituted azepin products.

As an alternative to ketenimine formation, it has been proposed that “stabilization” of the singlet nitrene can facilitate bond insertion reactions. These bond insertion reactions can conceivably occur via a two-step hydrogen atom abstraction and radical recombination process (on both singlet and triplet potential energy surfaces [PES]), or through a concerted pathway involving singlet nitrene insertion into a C–H or X–H bond (Scheme 3). To increase the stability of the singlet aryl nitrene species, and potentially facilitate bimolecular chemistry via nitrene bond insertion reactions, the groups of Platz and Keana introduced 2,3,5,6-tetrafluoro-phenylazide derivatives as new reagents for PAL.^58,59^ Marcinek et al.^60^ confirmed the longer lifetime of the singlet nitrenes derived from 4-azido-2,3,5,6-tetrafluorobenzamides. Computational studies by Karney and Borden^61^ concluded that the increased tendency of 4-azido-2,3,5,6-tetrafluorobenzamides to undergo nitrene-based chemistry rather than intramolecular rearrangement was due to steric factors that raised the energetic barrier to bicyclization (i.e., a higher energy TS2; Scheme 3). Further details on the mechanisms of ArN_3_ photochemistry, including the reactivity of the nitrene inside a defined protein environment,^62^ have been elaborated by the experimental and computational work from several groups.^54,62−71^ New ArN_3_-based reagents continue to be developed and a recent example includes 5-azido-2-aminopyridine that undergoes reversible ISC to facilitate nitrene-mediated PAL.^47^

### Photochemical Reactivity of Aryl Azides: Ketenimine Pathway

Computational results showing the calculated reaction coordinate for the photochemical activation, intramolecular rearrangement, and nucleophilic attack at the ketenimine species by methylamine (representing a model nucleophilic of Lys) on a model of compound **8** are presented in Figure 4. A detailed mechanistic scheme is presented in Figure S251. Numerical data are collected in Tables S3–S6 and equivalent plots of the calculated reaction coordinates for the other ArN_3_ models of compounds (**1**–**7** and **9**–**11**) are given in Figures S252–S272.

**Figure 4:**
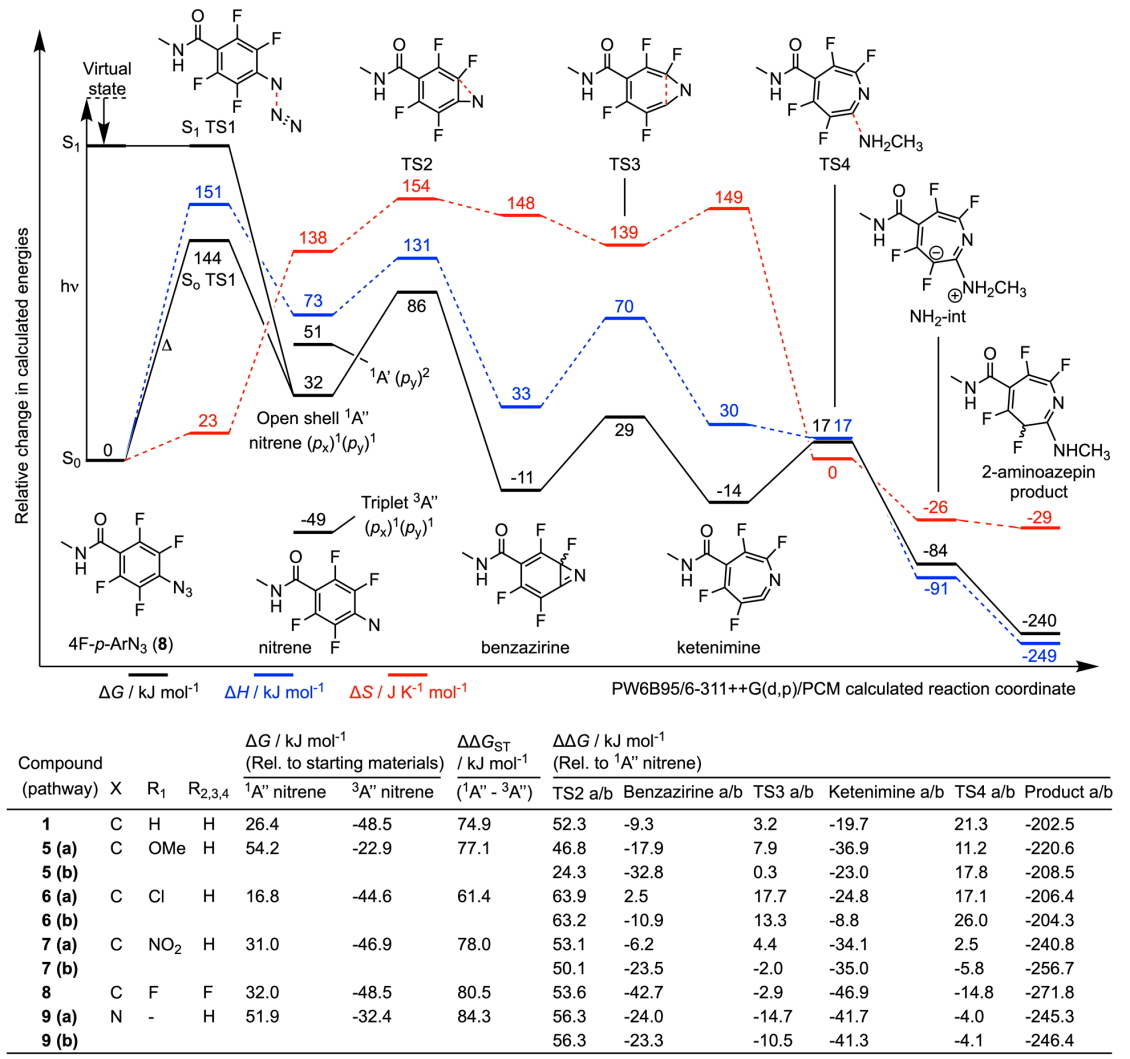
DFT calculated energetics on the singlet-state ketenimine pathway showing the intramolecular rearrangement of model compound **8** and subsequent nucleophilic addition of methylamine to form the 2-aminoazepin product. Pertinent data for several of the electronically diverse ArN_3_ models based on compounds **1** and **5**–**9** are also presented. Note: labels a and b in the compound column correspond to the alternative ketenimine pathways shown in Scheme 3.

Photon absorption by the model ArN_3_ species populates the lowest energy electronically excited singlet state (S_1_), which undergoes an essentially barrierless elimination of dinitrogen to form the ^1^A′′ (*p*_*x*_)^1^(*p*_*y*_)^1^ open-shell singlet nitrene via S_1_-TS1. Although all structures were optimized without symmetry constraints, the nitrenes converge with pseudo-*C*_s_ symmetry, and for clarity in distinguishing the different electronic states, the corresponding symmetry representations are used throughout. Our previous experimental estimates of the quantum yield for photochemical activation of compound **60** (Figure S235) gave a value of 4.35 ± 0.43%, which corresponds to nitrene formation for one in every ∼23 photons that are absorbed by the ArN_3_ (at ∼365 nm).^37^ We note here that photochemical activation of *para-*ArN_3_ species occurs over a wide window that starts in the UV region (<365 to 395 nm)^40,41^ and extends into the visible spectrum (at least 450 nm; Figure S238).

For model compound **8**, a calculated difference in free energy between the singlet and triplet nitrene, ΔΔ*G*_ST_ (^1^A′′ – ^3^A′′), was 80.5 kJ mol^–1^. The model *meta*-Cl-substituted ArN_3_ based on compound **6** gave the lowest ΔΔ*G*_ST_ value of 61.4 kJ mol^–1^, but overall, the calculations are consistent with the experimental data that indicate that ISC in aryl nitrenes is slow and that secondary photochemistry is dominated by reactivity of the singlet species.^52,54^

The calculated free energy barrier for the first step of intramolecular rearrangement to give the benzazirine species is given by TS2. For ease of comparison between the different model species, values are given relative to the energy of the ^1^A′′ nitrene. For model **8**, ΔΔ*G*(TS2) is 53.6 kJ mol^–1^, which is comparable to the values calculated for the nonsubstituted model **1**, the *meta*-NO_2_–ArN_3_ (model **7**), and the 2,4-pyridyl-N_3_ derivative (model **9**), where in each case, the thermodynamic barrier to rearrangement is lower than the singlet–triplet energy gap. All calculated values of TS2 are found to be thermodynamically accessible under the ambient conditions of our protein ligation reactions. It is interesting that our calculations suggest that there is no difference in the barrier to rearrangement when the ortho-positions with respect to the nitrogen atom are substituted with H or F atoms (models **1** versus **8**). This result contrasts with data from Karney and Borden^61^ whose calculations in the gas-phase agreed with experiments,^68,69,72,73^ and that suggested ortho-fluorination raises the barrier. We note that the differences observed between these previous reported calculations and the computational results here are likely due to the use of a DFT model, which cannot fully describe the electronic complexity of the open-shell nitrene species. Differences may also be expected between the published experimental (organic solvents) and computation studies (in vacuo), and our experiments and calculations which are performed in water. Except for the meta-substituted methoxy-derivative (model **5**), where rearrangement strongly favors ketenimine pathway b (ΔΔ*G*(TS2b) = 24.3 kJ mol^–1^ versus 46.8 kJ mol^–1^ for TS2b), the calculations indicated that no chemically significant difference between the two geometric pathways (a and b) exists for the dissymmetric models **6**, **7**, and **9**. For models investigated (Figures S252–S271), the transition state for ring expansion (TS3) was not rate determining and the rate limiting step to rearrangement was assigned to bicyclization (TS2). For model **8**, the ketenimine has a calculated free energy −46.9 kJ mol^–1^ lower than the ^1^A′′ nitrene. Indeed, most ketenimine species were found to be more stable than the corresponding ^1^A′′ nitrene, suggesting that the position of equilibrium lies heavily toward the ring expansion intermediate.

Except for the *m*-OMe derivative (model **5**), the calculated free energy barrier toward nucleophilic attack of the ketenimine by the MeNH_2_ was not rate limiting (Tables S3–S6). For instance, the energy of TS4 lies 32.0 kJ mol^–1^ above the corresponding ketenimine for model **8**, 41.0 kJ mol^–1^ for model **1**, and the highest and lowest values of 48.0 and 29.2 kJ mol^–1^ are assigned to models **5a** and **7b**, respectively. In all cases, nucleophilic attack by MeNH_2_, which represents a model of the lysine side chain, is calculated to be thermodynamically spontaneous and kinetically feasible under the experimental conditions.

### Photochemical Reactivity of Aryl Azides: Nitrene Bond Insertion Pathway

**Figure 5:**
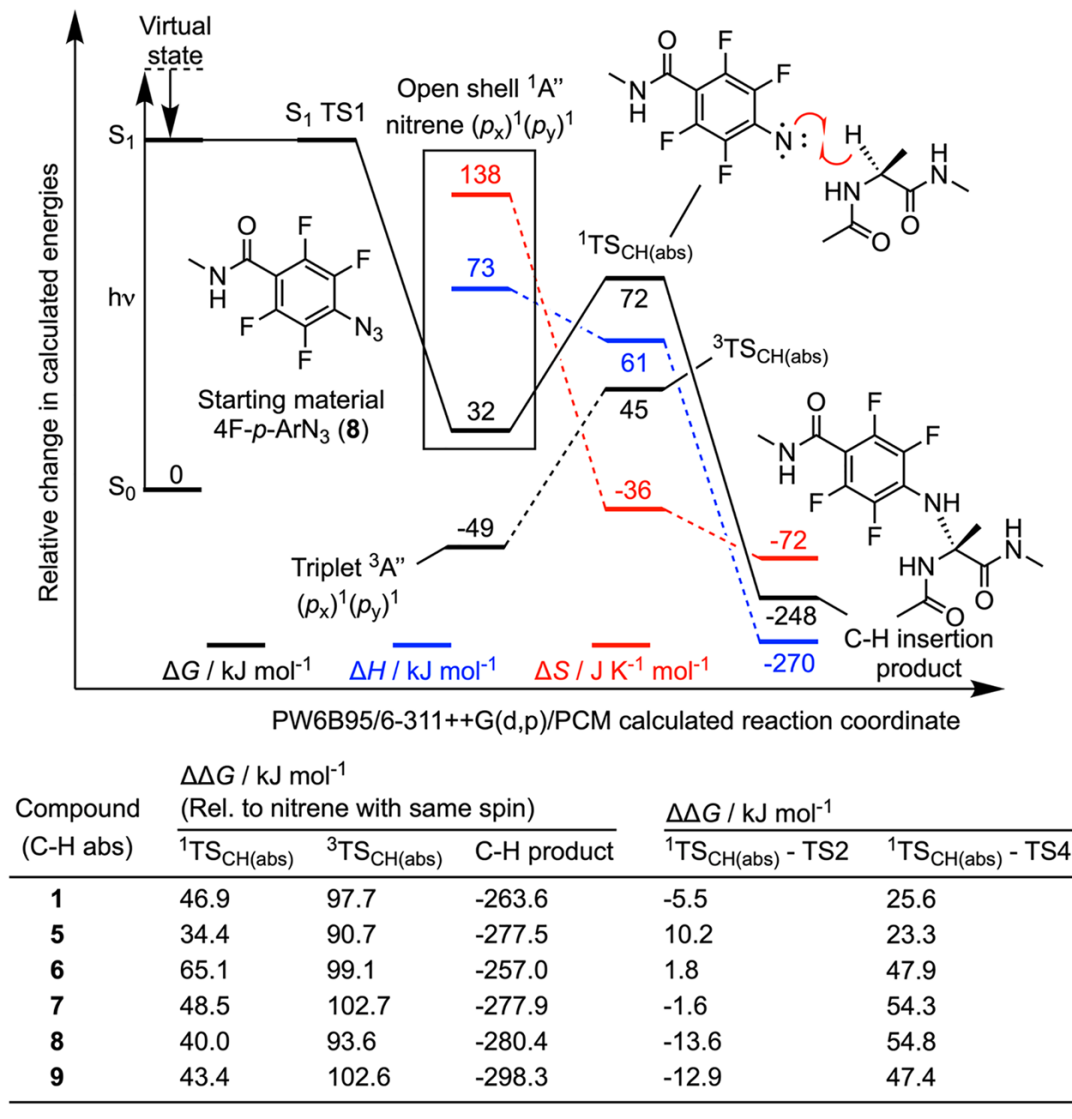
DFT calculated energetics on the singlet-state C_α_–H abstraction pathway showing the intermolecular reaction between model compound **8** and a model alanine to give the C_α_–H insertion product. Equivalent numerical data for model compounds **1**, **5**, **6**, **7**, and **9** are presented in Table S7. Note that for comparison, the value of TS4 in the tabulated data refers to MeNH_2_ attack at the corresponding ketenimine (see Figure 4).

Next, we explored the mechanisms and thermodynamics of nitrene bond insertion into the C_α_–H bond of a model of alanine. A plot of the calculated reaction coordinate for model **8**, and pertinent data for selected compounds, is presented in Figure 5 (numerical data are given in Table S7). Nitrene bond insertion can, in theory, proceed through a concerted or a sequential two-step process. All attempts to identify a transition state that would correspond to a concerted insertion of the N atom of the nitrene into the C_α_–H failed to locate a stationary point. The biradical nature of both the ^1^A′′ and ^3^A′′ species favors H atom abstraction. In all cases, the energy of the transition state that corresponds to breaking the C_α_–H bond and simultaneous formation of the N–H bond was found to be considerably lower in energy on the singlet PES than for the triplet. For example, for model **8**, the difference in energy of the singlet ^1^TS_CH(abs)_ relative to the ^1^A′′ nitrene was 40.0 kJ mol^–1^, whereas the equivalent energy difference for ^3^TS_CH(abs)_ on the triplet PES was 93.6 kJ mol^–1^. In general, this larger barrier is consistent with the increased lifetime of triplet species. After H atom abstraction and formation of the N–H bond, spontaneous radical recombination, and C_α_–N bond formation occurs on the singlet surface, with no additional intermediates found between the structure of ^1^TS_CH(abs)_ and the product. On the triplet surface, ISC is required before radical recombination can occur, which would facilitate separation of the two radicals by diffusion and reduce the yield of protein ligation. Across the different model nitrene species, H atom abstraction has the lowest barrier for the *m*-OMe model (**5**; ΔΔ*G*(^1^TS_CH(abs)_) = 34.4 kJ mol^–1^) with the highest barrier found for the *m*-Cl model **6** (ΔΔ*G*(^1^TS_CH(abs)_) = 65.1 kJ mol^–1^).

Competition between bimolecular nitrene bond insertion and unimolecular rearrangement to the ketenimine is reflected by the difference in energy between the associated transitions states (^1^TS_CH(abs)_ and TS2; Figure 5). Here, a negative value of ΔΔ*G*(^1^TS_CH(abs)_ – TS2) indicates that C_α_–H abstraction is thermodynamically more favorable than intramolecular rearrangement. The calculations show that model compounds **1**, **7**, **8**, and **9** favor bond insertion, whereas the *m*-OMe (**5**) and *m*-Cl derivatives (**6**) have lower barriers to bicyclization and ring expansion. Model **8** has the largest difference between the two transition states with ΔΔ*G*(^1^TS_CH(abs)_ – TS2) of −13.6 kJ mol^–1^ and for comparison, the value for model **1** is only −5.5 kJ mol^–1^. These results are consistent with the experimental data and indicate that if the nitrene is formed in situ in a defined protein environment, as with the classic preassociation mechanism of PAL, bioconjugate bonds are likely to be formed by X–H (where X = C, N, O, and S) insertion. However, when the photoactivatable ligand does not have a binding site with the protein, and therefore is unlikely to form a noncovalent preassociation complex, as is the case in our photoradiosynthesis of ^89^ZrDFO-labeled HSA, the lifetime of the ^1^A′′ nitrene is too short to allow efficient bimolecular chemistry to occur via nitrene insertion. Instead, unimolecular rearrangement to the more stable and longer-lived ketenimine will occur for all model compounds tested. Collectively, the calculations provide strong support that protein ligation with our photoactivatable DFO-PEG_3_-ArN_3_ species (**1**–**11**) produces HSA conjugates featuring a 2-substituted azepin linker.

### Chemoselectivity of Nucleophilic Addition to Ketenimines

**Figure 6:**
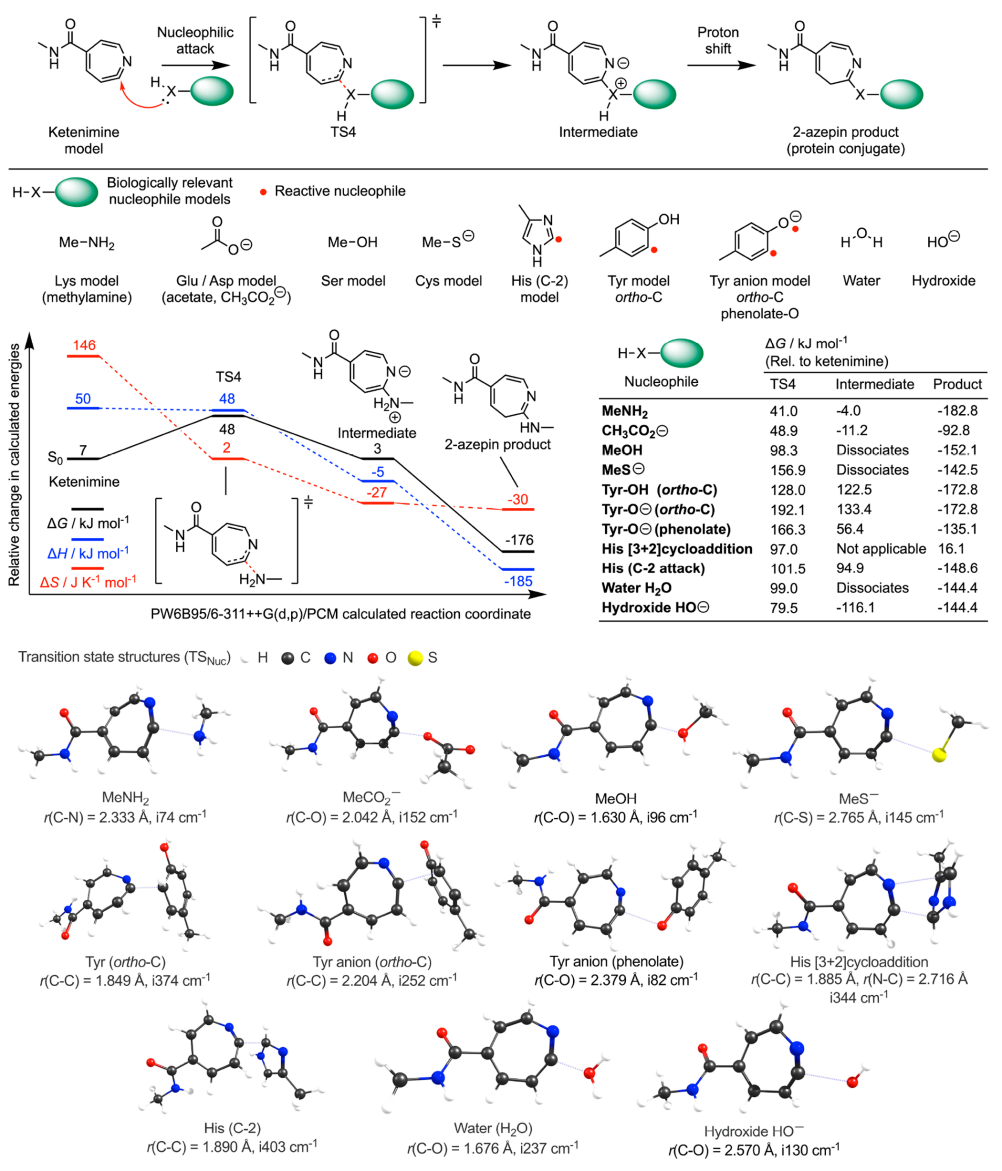
Mechanism and DFT calculated energetics on the nucleophilic addition of different biologically relevant nucleophile models to the electrophilic ketenimine model generated from photoactivation of compound **1**. Note: For the reaction coordinate, energetic values are given with respect to the starting materials. Numerical data are presented in Table S8.

Having established that bimolecular reactivity between the ArN_3_ species and HSA is likely mediated through the ketenimine pathway, we next used DFT to investigate the chemoselectivity of the nucleophilic attack. The detailed mechanism, structures of the nine different model nucleophiles tested, a plot of the calculated reaction coordinate for model compound **1**, and pictures showing the optimized transition states corresponding to the nucleophilic attack at the ketenimine are presented in Figure 6. Numerical data are given in Table S8. For concision, we focused on nucleophilic attack using model **1**, because DFO-PEG_3_-*p*-ArN_3_ gives the highest experimental RCYs in the photoradiosynthesis of ^89^ZrDFO-labeled HSA. The chosen nucleophiles represent valid computational models for Lys, Glu, Asp, Ser/Thr, Cys, His, and Tyr amino acids. We also included water and hydroxide anions that lead to the formation of the 2-hydroxyazepin byproduct via solvent quenching.^42^

The relative difference in calculated free energy between the ketenimine and the optimized transition states (TS4) indicate that nucleophilic attack by the primary amine MeNH_2_ (effectively modeling the ε-NH_2_ group on Lys) has the lowest barrier with Δ*G*(TS4) of only 41.0 kJ mol^–1^. After nucleophilic addition, proton rearrangement yields the 3*H*-2-aminoazepin product.^51^ The 3*H*-2-aminoazepin was found to be relatively stable in vivo.^37,42^ With the MeCO_2_^–^ anion, Δ*G*(TS4) was found to be 48.9 kJ mol^–1^, which is 7.9 kJ mol^–1^ higher in energy than the barrier to the ketenimine reaction with MeNH_2_. At a temperature of 298.15 K, this free energy difference corresponds to a Boltzmann factor of 0.042, meaning that the rate of MeNH_2_ attack at the ketenimine from model **1** is ∼24 times faster than MeCO_2_^–^. Importantly, if a carboxylate anion attacks the ketenimine, it gives the 3*H*-azepin-2-yl carboxylate product, which is likely to be unstable with respect to hydrolysis and subsequent formation of the 2-hydroxyazepin byproduct. Hence, if bioconjugate bonds are initially formed with Glu or Asp amino residues, the labeled HSA protein is likely to be unstable under physiological conditions where hydrolysis releases ^89^ZrDFO-PEG_3_-2-hydroxyazepin. Interestingly, the hydrolysis reaction would simultaneously release the original free carboxylate anion, and hence, the mechanism is potentially catalytic.

Nucleophilic attacks at the ketenimine by the other model amino acid nucleophiles were all found to be thermodynamically uncompetitive. The transition states associated with hydrolysis, either by water or hydroxide anions, were calculated to be much higher in energy with Δ*G*(TS4) values of 99.0 and 79.5 kJ mol^–1^, respectively. This is an important result that is consistent with our experimental observations that photoradiosynthesis with ArN_3_ compounds **1**–**11** produces stable ^89^ZrDFO-PEG_3_-HSA conjugates in aqueous conditions at pH 8.0–8.4. From the DFT calculations, water is uncompetitive as a nucleophile but as pH increases, the higher concentration of hydroxide anions could present a plausible competing reaction that would decrease the overall yield of functionalized protein. Crucially, our previous experimental work provides full support for this conclusion. Studies on the pH dependence of photoradiosynthesis found a strong pH dependence with the highest RCYs obtained between pH 8.0 and 8.7 and decreased yields when the pH was increased to >9.1.^35,49^ It is fortuitous that the ketenimines investigated show an energetic preference for reaction with amine nucleophiles and are sufficiently stable in water, which facilitates bimolecular protein ligation.

### Photochemical Reactivity and Chemoselectivity of Protein Ligation with Benzophenone Model **12**

The solution-phase photochemistry of benzophenone (BP) was originally studied by Moore et al. in 1961, who confirmed that electronic excitation generates a triplet ketyl biradical.^74^ Later, Galardy and Craig^75^ demonstrated that BP is an effective photochemical probe for investigating ligand–protein interactions. Since then, BP derivatives have been used extensively in PAL.^76−79^ For a comprehensive overview of the excited-state photochemistry of BP derivatives, the reader is referred to the excellent review by Dormán et al.^48^

**Figure 7:**
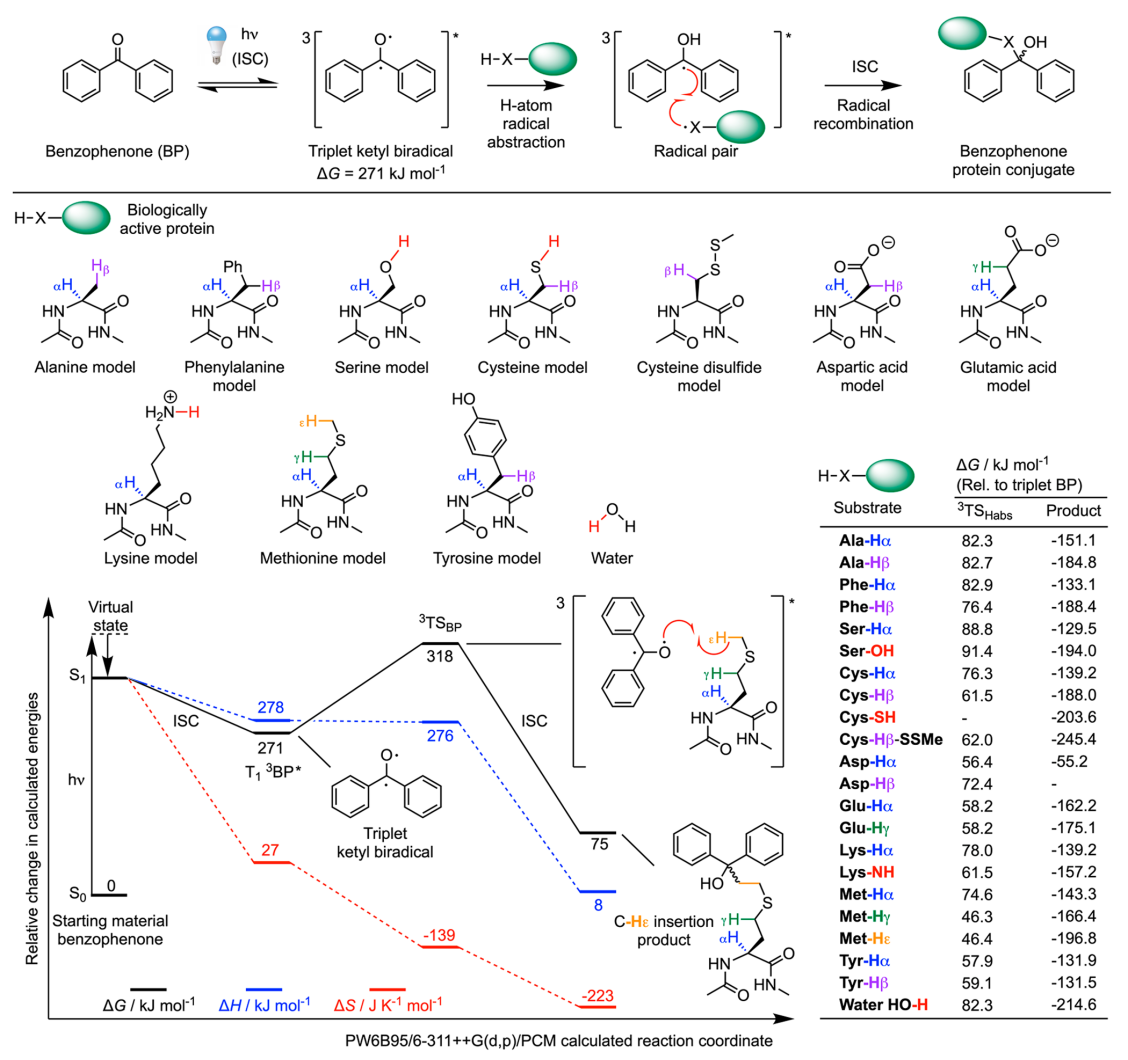
Mechanism and DFT calculated energetics on the triplet-state ketyl biradical hydrogen atom abstraction pathway between model benzophenone (BP) **12** and model amino acids to give the corresponding X–H insertion products. Numerical data are presented in Table S9.

The mechanism of photochemical activation of BP model **12** to the electronically excited triplet T_1_(n,π*) state and subsequent bond insertion through a two-step H atom radical abstraction followed by ISC and radical recombination is shown in Figure 7 (see also Table S9). We used DFT calculations to investigate the chemoselectivity of the triplet ketyl biradical (T_1_–^3^BP*) with 10 different models of amino acids. The rate-determining step involves H atom radical abstraction by the triplet T_1_–^3^BP* via the transition state ^3^TS_BP_.^48^ Subsequent ISC and surface hopping facilitates radical recombination and the formation of a covalent bond between the protein and the BP derivative.

When comparing the calculated change in free energy of the different values of ^3^TS_BP_ relative to the ^3^BP* species, the calculations indicate that the ketyl diradical shows a powerful thermodynamic preference for H_γ_ and H_ε_ positions on methionine. The transition states for Met-H_γ_ and Met-H_ε_ abstractions were found to be considerably lower in energy than all other model reactants with Δ*G*(^3^TS_BP_) values of 46.3 and 46.4 kJ mol^–1^, respectively. In contrast, the value of ^3^TS_BP_ for H atom abstraction at the Met-H_α_ position lies at 74.6 kJ mol^–1^ above the ^3^BP* excited state intermediate. This thermodynamic preference for methionine is fully consistent with the experimental report from Rihakova et al.^80^ who used BP-derivatives to develop a methionine proximity assay (MPA) for scanning the ligand binding sites of G-protein coupled receptors.^48^ Later, Wittelsberger^81^ confirmed these observations and described how methionine acts as a “magnet” in BP-based PAL. The fact that our DFT results are consistent with the experimental data on Met-selectivity provide confidence in the calculations.

The relatively high thermodynamic barriers of ^3^BP* insertion into most X–H bonds (where X = C, O, S, and N) is also consistent with our experiments where photoradiosynthesis using DFO-PEG_3_-BP (**12**) gave the corresponding ^89^ZrDFO-PEG_3_-HSA conjugate in a RCY of 29.6 ± 2.8% (*n* = 3). When the reaction is performed in aerated solution under ambient conditions of temperature and pressure, the lower isolated yield of the radiolabeled protein is consistent with the decreased rate of the reaction with HSA that arises because of the higher barriers. These increased energy barriers mean that the effective concentration of thermodynamically accessible amino acid residues (mainly Met) is lower than the available nucleophilic Lys residues that are required for conjugation via the ArN_3_/ketenimine photochemistry. Nevertheless, the use of BP derivatives does offer a potential photochemical mechanism for developing site-specific labeled proteins and has already been exploited by several groups for light-activated site-specific labeling of native (nonfunctionalized) mAbs.^82−85^ In spite of the lower RCY obtained with compound **12**, the results provide an encouraging precedent that bimolecular protein ligation is accessible with BP derivatives. One attractive feature of BP chemistry is that the two different aryl rings can be easily functionalized with electronically active substituents, which offers a way of controlling the stability and reactivity of the ketyl biradical.^86^

### Photochemical Reactivity and Chemoselectivity of Protein Ligation with Diazirine Model **13**

**Figure 8:**
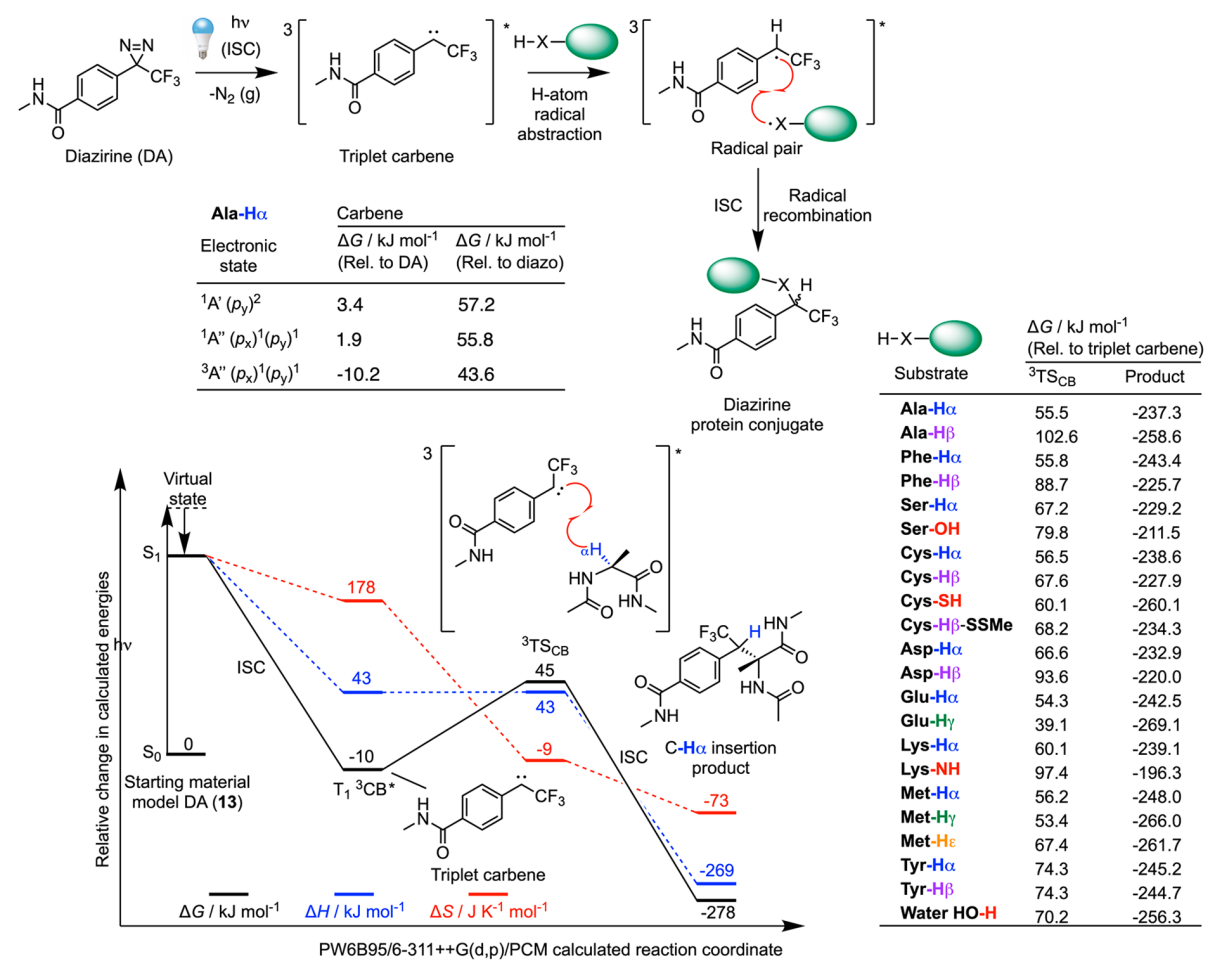
Mechanism and DFT calculated energetics on the triplet-state hydrogen atom abstraction pathway between model diazirine compound **13** and model alanine to give the two-step C_α_–H insertion product. Full computational data are presented in Table S10. Note: energetic predictions for the open-shell carbene species are presented as a guide only, as a complete description of the electronic structure is not possible with the DFT methodology used here.

Aryl diazirines (aryl-DAs) were first used in PAL by Smith and Knowles in 1973.^87^ In particular, the para-substituted trifluoromethyl phenyl diazirine reagents^88^ have become popular in drug-target discovery.^89−91^ Recent work has also demonstrated that carbenes produced from photoactivation of aryl-DAs facilitate accurate mapping of protein–ligand binding sites.^92^ In 2021, Mosolino et al.^93^ reported detailed structure–activity relationships in substituted aryl-DAs, revealing that electron-rich systems show an increased tendency toward C–H insertion. This enhancement in C–H insertion was assigned to an increased stabilization of the singlet carbene species. However, for the vast majority of aryl-DA compounds used in PAL, facile and reversible ISC may occur, which arises from the small energy gap between the singlet and triplet electronic states of the carbene.^52^ To the best of our knowledge, no distinct chemoselective labeling has been observed for aryl diazirine species. However, recent studies on aliphatic diazirines have shown selectivity toward Cys,^94^ as well as preference for reactions with Tyr, Glu, and Asp residues.^95^ On the basis of these observations with aliphatic diazirines, it is reasonable to suppose that aryl-DA reagents may also exhibit preferential reactivity with certain amino acid residues on protein. To probe this effect, we used DFT calculations to investigate the reaction coordinate between model aryl-DA (**13**) and model amino acids (Figure 8 and Table S10).

Interestingly, the DFT calculations indicate that the cyclic isomer of model **13** is 54 kJ mol^–1^ less stable than the linear structure. The existence of two isomeric structures for diazirine species is well established and detailed studies on the stepwise release of dinitrogen have been reported.^96,97^ Photon absorption leads to the loss of dinitrogen to give a singlet. The ^1^A′′(*p*_*x*_)^1^(*p*_*y*_)^1^ open-shell and ^1^A′(*p*_*y*_)^2^ closed-shell singlet carbenes are predicted to lie close in energy. The triplet carbene ^3^A′′(*p*_*x*_)^1^(*p*_*y*_)^1^ is calculated to be the most stable intermediate, and in our calculations, the relatively small singlet–triplet energy gap of 12.1 kJ mol^–1^ is consistent with the experimental data that show that carbenes undergo rapid ISC.^52,90^

First, we calculated the barrier (TS_CB_) toward C_α_–H abstraction on the Ala-H_α_ model for each of the different spin states of the carbene produced from model **13** (Figure 8). Data indicate that the free energy barrier is lowest for the triplet state where Δ*G*(^3^TS_CB_) = 55.5 kJ mol^–1^. Given that ISC is expected to be fast, the DFT results are consistent with the mechanism of two-step carbene insertion into protein bonds occurring via the triplet pathway. As with the chemistry of BP derivatives (vide supra), subsequent ISC and radical recombination is likely to be fast, leading to the formation of a covalent bond between the protein and the aryl-DA derivative.

Next, we explored the chemoselectivity of the two-step carbene insertion into the three different accessible C–H bonds on a panel of model amino acids. Remarkably, for methionine, our data indicate an opposite trend for carbene-based H atom abstraction using model **13**, to that observed for the reactivity of the BP derivative (model **12**). The barrier Δ*G*(^3^TS_CB_) for the Met-H_α_ abstraction was 56.2 kJ mol^–1^ and was 53.4 and 67.4 kJ mol^–1^ for Met-H_γ_ and Met-H_ε_, respectively. Reasons why the chemoselectivity on methionine is inverted are not apparent from the calculations but these data are consistent with the experimental situation in which no equivalent of the BP methionine selectivity has been observed for aryl-DA compounds. The calculations predict that Δ*G*(^3^TS_CB_) for Glu-H_γ_ has the lowest barrier of 39.1 kJ mol^–1^. Aliphatic carbenes have also shown some preference for reactions with Glu and Asp residues,^95^ but to the best of our knowledge, equivalent experiments are yet to be reported for aryl-DA derivatives.

### Photochemical Reactivity and Chemoselectivity of Protein Ligation with Tetrazole Model **14**

The generation of nitrile imine intermediates from tetrazoles was first reported in 1967.^98^ More recently, Qing and co-workers developed tetrazoles as “photoclick” reagents for applications in protein labeling.^99^ Initially, the reactivity of the nitrile imine produced after photoactivation of tetrazoles was proposed to be bioorthogonal,^100,101^ where preinstallation of a reactive alkene on the protein facilitated site-selective (and fluorogenic) conjugation via a [3 + 2] cycloaddition reaction. However, subsequent work^102,103^ disproved the biorthogonality, and further experiments demonstrated that the nitrile imine can react with many different nucleophiles, as well as the amino acid residues Glu, Asp, Trp, Pro, His, Ser, Tyr, and Asn.^102−106^ Nitrile imine reactivity is known to be sensitive to pH, and this fact was exploited by Bach et al., who developed tetrazole assays for proteome-wide profiling of Asp and Glu residues in living bacteria.^107^ To the best of our knowledge, computational studies on the chemoselectivity of tetrazoles have not been reported previously.

**Figure 9:**
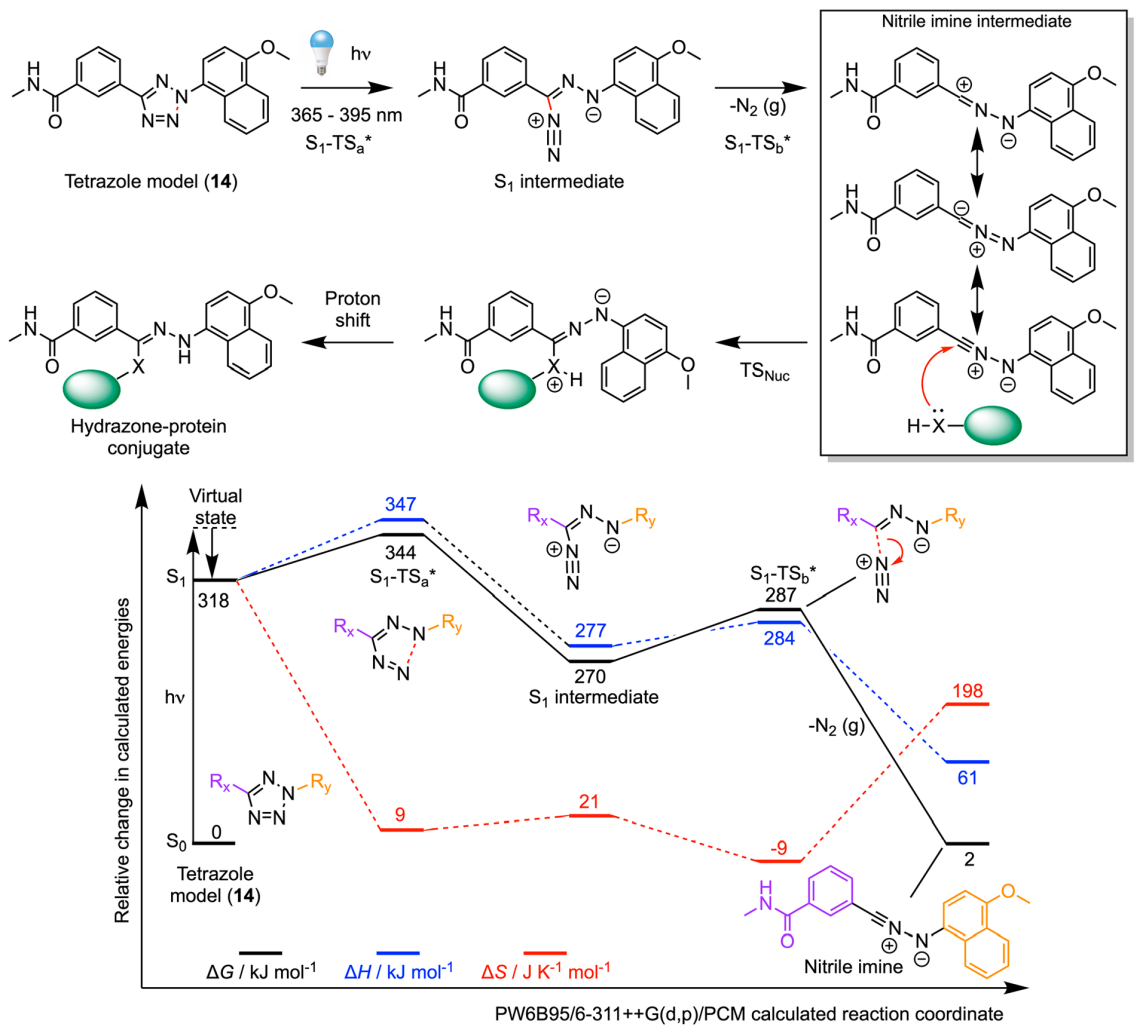
Mechanism and DFT calculated energetics on the photoactivation of a model tetrazole (**14**) to give the reactive nitrile imine electrophile. Numerical data are presented in the Table S11. Note: energetic predictions for the S_1_-state species are presented as a guide only, as a complete description of the electronic structure is not possible with the DFT methodology used here. The calculated reaction profile is qualitatively similar to the results presented by Menzel et al.^109^ but caution should be used when comparing the numbers versus experimental data.

The mechanisms of photochemical activation, and the calculated reaction coordinate leading to the formation of the key nitrile imine intermediate produced from photoactivation of model **14** is presented in Figure 9. Others have also used experimental and computational tools to study the formation and reactivity of nitrile imines generated from photoactivation of tetrazoles, but mainly in the context of cycloaddition reactions.^108,109^ Interestingly, after the initial photon absorption, nitrile imine production occurs on the first excited singlet PES through a two-step mechanism. The first step is rate determining where the transition state S_1_-TS_a_* is accessible under ambient conditions (ΔΔ*G*(S_1_-TS_a_*) = 26 kJ mol^–1^) and involves N–N bond cleavage to give the excited state S_1_ intermediate. Next, dinitrogen is spontaneously released to give the ground state (S_0_) nitrile imine via a slightly lower energy barrier (ΔΔ*G*(S_1_-TS_b_*) = 17 kJ mol^–1^).

**Figure 10:**
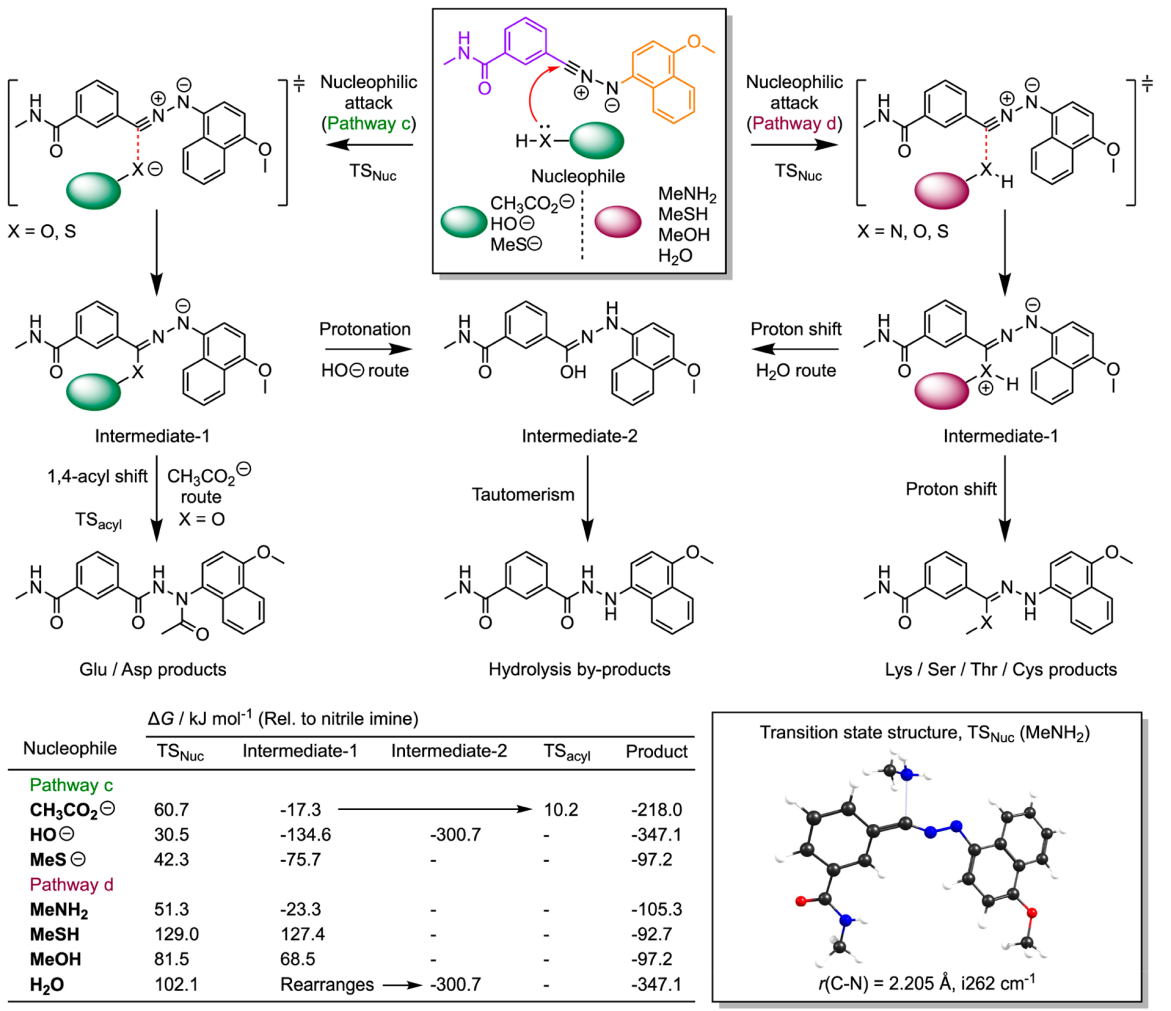
Mechanisms and DFT calculated energetics on the attack of the nitrile imine electrophile generated from photoactivation of model tetrazole **14** by several biologically relevant nucleophiles (see also Table S11).

At this juncture, nucleophilic attack at the nitrile imine can occur by at least two different pathways that differ based on the protonation state of the incoming nucleophile and with the subsequent possibilities for intermediate rearrangement (Figure 10; pathway c involves anionic nucleophiles, whereas pathway d involves attack by neutral protic nucleophiles). The calculated rate-determining transition state barriers for attack at the nitrile imine by the models of relevant biological nucleophiles indicate that reaction with primary amines (MeNH_2_ modeling Lys) has a low barrier, Δ*G*(TS_Nuc_), of 51.3 kJ mol^–1^. The equivalent free energy barrier for reaction with carboxylate groups, (MeCO_2_^–^ modeling Glu and Aps residues) lies at 60.7 kJ mol^–1^ above the energy of the nitrile imine, whereas quenching with either water or hydroxide anions has a barrier of 102.1 and 30.5 kJ mol^–1^, respectively. One of lowest barriers was found for the addition of the MeS^–^ anion (Δ*G*(TS_Nuc_) = 42.3 kJ mol^–1^). Given that the p*K*_a_ value of the thiol group on Cys is usually around 8.6, it is plausible that under our experimental conditions, labeling of free Cys residues on HSA can occur. However, we note that HSA has only one available free Cys, with the other Cys residues involved in the formation of disulfide bridges. Therefore, it is statistically more likely that HSA labeling using compound **14** occurs via reactivity of the Lys, Glu, or Asp residues.

The calculations are fully consistent with the reported pH-dependence of the nucleophilic attack at the nitrile imine.^102^ Our DFT results predict that under more basic conditions, hydroxide anions can effectively compete with bimolecular protein ligation reactions of the nitrile imine. In contrast, under our experimental conditions, where the hydroxide anion concentration is significantly lower than the protein (or nucleophile) concentration, successful labeling is observed. At acidic or near neutral pH, water cannot compete with amine, carboxylate, or anionic sulfhydryl attack at the nitrile imine. Under acidic conditions (below pH 7), protein ligation is likely mediated by Glu or Asp residues. In our photoradiosynthesis reactions, ^89^Zr-radiolabeling of HSA using compound **14** is likely to involve the formation of several different types of bioconjugate bond produced mainly from the reactivity of Lys, Glu, Asp, and potentially Cys. Overall, these calculations suggest that the chemoselectivity of native protein labeling using photoactivation of tetrazoles can be controlled, to some degree, by modifying the reaction pH.

## Conclusions

The use of photochemically initiated reactions to produce new covalent bonds with native (unmodified) proteins is proven to be a general and widely applicable route for accessing functionalized protein conjugates. The synthetic and radiochemical work presented confirms that ^89^Zr-labeled proteins can be produced from 14 different photoactivatable derivatives of the metal-ion-binding chelate DFO. Under standardized experimental conditions, photoradiosynthesis using compounds **1**–**14** produced the corresponding ^89^ZrDFO-PEG_3_-HSA conjugates with decay-corrected isolated radiochemical yields between 18.1 ± 1.8% and 62.3 ± 3.6%. Detailed computational studies were used to investigate the photochemical activation steps and chemoselectivity arising from the light-triggered reactivity of our model compounds. The photoactivatable compounds studied include substituted aryl azides, benzophenone, aryl diazirine, and tetrazole derivatives, whereby successful bimolecular protein conjugation with compounds **1**–**14** involves at least five distinct mechanisms, each producing a different type of bioconjugate bond. Collectively, the experiments and calculations provide a deeper understanding of the reaction mechanisms through which photochemistry can be used to create new functionalized proteins, such as ^89^Zr-labeled mAbs, for applications in molecular imaging. The next frontier in this field will involve the development of site-selective photochemical reagents to access well-defined and stoichiometrically precise protein conjugates.

## Supporting Information Available

Experimental details, NMR spectra, and high-resolution mass spectrometry data for all new compounds, radiochemical data, and computational details including the calculated thermodynamics; and Cartesian coordinates of optimized structures are available on request (PDF)
