# Supplementary material for: Charting the Chemical and Mechanistic Scope of Light-Triggered Protein Ligation
Source: JACS Au. 2022 Feb 8;2(3):646–64. doi: 10.1021/jacsau.1c00530 (PMC8970001; doi:10.1021/jacsau.1c00530)
Supplement: Supplementary file 1 — au1c00530_si_001.pdf [file au1c00530_si_001.pdf]

Supporting Information

Charting the chemical and mechanistic scope of light-triggered protein ligation

Daniel F. Earley,‡ Amaury Guillou,‡ Simon Klingler, Rachael Fay, Melanie Gut, Faustine d'Orchymont, Shamisa Behmaneshfar, Linus Reichert and Jason P. Holland*

University of Zurich, Department of Chemistry, Winterthurerstrasse 190, CH-8057, Zurich, Switzerland

*** Corresponding author:**
Prof. Dr Jason P. Holland
Tel: +41.44.63.53.990
E-mail: jason.holland@chem.uzh.ch
Website: www.hollandlab.org

‡ **Co-first authors:**
Dr Daniel F. Earley
Email: daniel.earley@chem.uzh.ch

Dr Amaury Guillou
Email: amaury.guillou@chem.uzh.ch

Table of Contents

Methods and Materials *15*

General **15**

Photochemistry **16**

Radioactivity and radioactive measurements **17**

^{89}Zr-radioactive stocks **17**

^{89}Zr-Radiolabeling of ligands **18**

^{89}Zr-photoradiolabeling of protein **18**

Statistical analysis **18**

Density functional theory calculations **19**

Synthesis and Characterisation Data *21*

General Procedures **21**

General Procedure A: Diazo-transfer of aryl amine derivatives. 21

General Procedure B: PEGylation of photoactivatable carboxylic acid derivatives via HATU mediated amide coupling. 21

General Procedure C: TFA mediated deprotection of Boc-protected amines. 22

General Procedure D: Carboxylic acid functionalisation of photoactivatable PEG_3 derivatives. 22

General Procedure E: HATU mediated amide coupling of carboxylic acid functionalized photoactivatable PEG_3 derivatives to desferrioxamine B mesylate. 22

General Procedure F: Carboxylic acid activation to the corresponding N-hydroxysuccinimide ester 23

General Procedure G: N-Hydroxysuccinimide ester activated coupling to DFO mesylate 23

Synthesis of DFO-PEG_3-para-ArN_3 (1) **24**

Synthesis of DFO-PEG_3-meta-ArN_3 (2) **25**

Synthesis of DFO-PEG_3-ortho-ArN_3 (3) **27**

Synthesis of DFO-PEG_3-para-EtArN_3 (4) **29**

Synthesis of DFO-PEG_3-meta-OMe-ArN_3 (5) **30**

Synthesis of DFO-PEG_3-meta-Cl-ArN_3 (6) **32**

Synthesis of DFO-PEG_3-meta-NO_2-ArN_3 (7) **34**

Synthesis of DFO-PEG_3-4F-ArN_3 (8) **36**

Synthesis of DFO-PEG_3-2,4-pyridyl-N_3 (9) **38**

Synthesis of DFO-PEG_3-2,3-naphthyl-N_3 (10) **41**

Synthesis of DFO-PEG_3-2,6-naphthyl-N_3 (11) **43**

Synthesis of DFO-PEG_3-Benzophenone (12) **45**

Synthesis of DFO-PEG_3-Diazirine (13) **47**

Synthesis of DFO-PEG_3-OMe-Tetrazole (14) **49**

Synthesis of non-PEGylated DFO-ArN_3 derivatives *128*

Synthesis of DFO-para-ArN_3 (60) **128**

Synthesis of DFO-4F-p-ArN_3 (62) **129**

Synthesis of DFO-m-NO_2-ArN_3 (63) **130**

Synthesis of DFO-2,6-naphthyl-N_3 (65) **131**

Synthesis of DFO-Benzophenone (66) **132**

Synthesis of DFO-Diazirine (67) **133**

Radiochemistry results ***144***

^{89}Zr-photoradiolabeling of with non-PEGylated DFO compounds **144**

^{nat}Zr labeling of compounds 1 to 14 .... **145**

Table S1. Reverse-phase HPLC retention times and HR-ESI-MS data for the ^{nat}Zr/^{89}Zr complexes of compounds 1 to 14. .... **146**

Photoradiolabeling of human serum albumin (HSA) with ^{89}Zr **147**

Table S2. Experimentally measured decay-corrected isolated radiochemical yields (RCYs) for the photoradiosynthesis of ^{89}ZrDFO-PEG$_3$-HSA derivatives using compounds 1 to 14 .... **154**

Computational results ***155***

Table S3. Calculated energetics for the photo-initiated reaction of two model aryl azides with methylamine and acetate using the PW6B95/6-311++G(d,p) methodology with a PCM (water) solvation model. **156**

Table S4. Calculated energetics for the photo-initiated reaction of two model aryl azides with methylamine and acetate using the PW6B95/6-311++G(d,p) methodology with a PCM (water) solvation model. **158**

Table S5. Calculated energetics for the photo-initiated reaction of two model aryl azides with methylamine and acetate using the PW6B95/6-311++G(d,p) methodology with a PCM (water) solvation model. **160**

Table S6. Calculated energetics for the photo-initiated reaction of two model aryl azides with methylamine and acetate using the PW6B95/6-311++G(d,p) methodology with a PCM (water) solvation model. **162**

Table S7. Calculated energetics relative to the starting materials using the PW6B95/6-311++G(d,p) methodology with a PCM (water) solvation model for the C-H abstraction and bond insertion of nitrenes into the C-H bonds of model alanine and methionine. **185**

Table S8. Calculated energetics relative to the starting materials using the PW6B95/6-311++G(d,p) methodology with a PCM (water) solvation model for the nucleophilic addition of various biologically relevant nucleophile models to the ketenimine produced after photoactivation of model compound 1. .... **185**

Table S9. Calculated energetics relative to the starting materials using the PW6B95/6-311++G(d,p) methodology with a PCM (water) solvation model for the C-H abstraction transition state and product formed between the excited triplet ketyl biradical of benzophenone (BP) and selected amino acid model compounds. **186**

Table S10. Calculated energetics relative to the starting materials using the PW6B95/6-311++G(d,p) methodology with a PCM (water) solvation model for the formation of different carbene electronic states, and the associated C-H abstraction transition states and products formed between model diazirine 13 and selected amino acid model compounds. **187**

Table S11. Calculated energetics relative to the starting materials using the PW6B95/6-311++G(d,p) methodology with a PCM (water) solvation model for the nucleophilic attack at the nitrile imine formed between model tetrazole 14 and different biologically relevant nucleophiles. **188**

References ***189***

List of Figures

Figure S1. ^{1}H (400 MHz, DMSO-d^6, 298 K) NMR spectrum of compound **1** 52
Figure S2. ^{13}C (101 MHz, DMSO-d^6, 298 K) NMR spectrum of compound **1** 52
Figure S3. HR-ESI-MS spectrum of compound **1** 52
Figure S4. ^{1}H-^{1}H (COSY, DMSO-d^6, 298 K) 2D NMR spectrum of compound **1** 53
Figure S5. ^{1}H-^{13}C (HSQC, DMSO-d^6, 298 K) 2D NMR spectrum of compound **1** 53
Figure S6. ^{1}H (400 MHz, DMSO-d^6, 298 K) NMR spectrum of compound **15** 54
Figure S7. ^{13}C (101 MHz, DMSO-d^6, 298 K) NMR spectrum of compound **15** 54
Figure S8. HR-ESI-MS spectrum of compound **15** 54
Figure S9. ^{1}H (400 MHz, $CDCl_3$, 298 K) NMR spectrum of compound **16** 55
Figure S10. ^{13}C (101 MHz, $CDCl_3$, 298 K) NMR spectrum of compound **16** 55
Figure S11. HR-ESI-MS spectrum of compound **16** 55
Figure S12. ^{1}H (400 MHz, $CDCl_3$, 298 K) NMR spectrum of compound **17** 56
Figure S13. ^{13}C (101 MHz, $CDCl_3$, 298 K) NMR spectrum of compound **17** 56
Figure S14. HR-ESI-MS spectrum of compound **17** 56
Figure S15. ^{1}H (400 MHz, MeOD-d^4, 298 K) NMR spectrum of compound **18** 57
Figure S16. ^{13}C (101 MHz, MeOD-d^4, 298 K) NMR spectrum of compound **18** 57
Figure S17. HR-ESI-MS spectrum of compound **18** 57
Figure S18. ^{1}H (400 MHz, DMSO-d^6, 298 K) NMR spectrum of compound **2** 58
Figure S19. ^{13}C (101 MHz, DMSO-d^6, 298 K) NMR spectrum of compound **2** 58
Figure S20. HR-ESI -MS spectrum of compound **2** 58
Figure S21. ^{1}H-^{1}H (COSY, DMSO-d^6, 298 K) 2D NMR spectrum of compound **2** 59
Figure S22. ^{1}H-^{13}C (HSQC, DMSO-d^6, 298 K) 2D NMR spectrum of compound **2** 59
Figure S23. ^{1}H (400 MHz, MeOD-d^4, 298 K) NMR spectrum of compound **19** 60
Figure S24. ^{13}C (101 MHz, MeOD-d^4, 298 K) NMR spectrum of compound **19** 60
Figure S25. HR-ESI-MS spectrum of compound **19** 60
Figure S26. ^{1}H (400 MHz, $CDCl_3$, 298 K) NMR spectrum of compound **20** 61
Figure S27. ^{13}C (101 MHz, $CDCl_3$, 298 K) NMR spectrum of compound **20** 61
Figure S28. HR-ESI-MS spectrum of compound **20** 61
Figure S29. ^{1}H (400 MHz, $CDCl_3$, 298 K) NMR spectrum of compound **21** 62
Figure S30. ^{13}C (101 MHz, $CDCl_3$, 298 K) NMR spectrum of compound **21** 62
Figure S31. HR-ESI-MS spectrum of compound **21** 62
Figure S32. ^{1}H (400 MHz, $CDCl_3$, 298 K) NMR spectrum of compound **22** 63

Figure S33. ^{13}C (101 MHz, $CDCl_3$, 298 K) NMR spectrum of compound **22** 63
Figure S34. HR-ESI-MS spectrum of compound **22** 63
Figure S35. ^{1}H (400 MHz, DMSO-d^6, 298 K) NMR spectrum of compound **3** 64
Figure S36. ^{13}C (101 MHz, DMSO-d^6, 298 K) NMR spectrum of compound **3** 64
Figure S37. HR-ESI-MS spectrum of compound **3** 64
Figure S38. ^{1}H-^{1}H (COSY, DMSO-d^6, 298 K) 2D NMR spectrum of compound **3** 65
Figure S39. ^{1}H-^{13}C (COSY, DMSO-d^6, 298 K) 2D NMR spectrum of compound **3** 65
Figure S40. ^{1}H (500 MHz, DMSO-d^6, 298 K) NMR spectrum of compound **4** 66
Figure S41. ^{13}C (126 MHz, DMSO-d^6, 298 K) NMR spectrum of compound **4** 66
Figure S42. HR-ESI-MS spectrum of compound **4** 66
Figure S43. ^{1}H-^{1}H (COSY, DMSO-d^6, 298 K) 2D NMR spectrum of compound **4** 67
Figure S44. ^{1}H-^{13}C (HSQC, DMSO-d^6, 298 K) 2D NMR spectrum of compound **4** 67
Figure S45. ^{1}H (400 MHz, $CDCl_3$, 298 K) NMR spectrum of compound **23** 68
Figure S46. ^{13}C (101 MHz, $CDCl_3$, 298 K) NMR spectrum of compound **23** 68
Figure S47. HR-ESI-MS spectrum of compound **23** 68
Figure S48. ^{1}H (400 MHz, $CDCl_3$, 298 K) NMR spectrum of compound **24** 69
Figure S49. ^{13}C (101 MHz, $CDCl_3$, 298 K) NMR spectrum of compound **24** 69
Figure S50. HR-ESI-MS spectrum of compound **24** 69
Figure S51. ^{1}H (400 MHz, $CDCl_3$, 298 K) NMR spectrum of compound **25** 70
Figure S52. ^{13}C (101 MHz, $CDCl_3$, 298 K) NMR spectrum of compound **25** 70
Figure S53. HR-ESI-MS spectrum of compound **25** 70
Figure S54. ^{1}H (400 MHz, $CDCl_3$, 298 K) NMR spectrum of compound **26** 71
Figure S55. ^{13}C (101 MHz, $CDCl_3$, 298 K) NMR spectrum of compound **26** 71
Figure S56. HR-ESI-MS spectrum of compound **26** 71
Figure S57. ^{1}H (400 MHz, DMSO-d^6, 298 K) NMR spectrum of compound **5** 72
Figure S58. ^{13}C (126 MHz, DMSO-d$^6{}_3$, 298 K) NMR spectrum of compound **5** 72
Figure S59. HR-ESI-MS spectrum of compound **5** 72
Figure S60. ^{1}H-^{1}H (COSY, DMSO-d^6, 298 K) 2D NMR spectrum of compound **5** 73
Figure S61. ^{1}H-^{13}C (HSQC, DMSO-d^6, 298 K) 2D NMR spectrum of compound **5** 73
Figure S62. ^{1}H (400 MHz, MeOD-d^4, 298 K) NMR spectrum of compound **27** 74
Figure S63. ^{13}C (101 MHz, MeOD-d^4, 298 K) NMR spectrum of compound **27** 74
Figure S64. HR-ESI-MS spectrum of compound **27** 74
Figure S65. ^{1}H (400 MHz, $CDCl_3$, 298 K) NMR spectrum of compound **28** 75
Figure S66. ^{13}C (101 MHz, $CDCl_3$, 298 K) NMR spectrum of compound **28** 75

Figure S67. HR-ESI-MS spectrum of compound **28** ... 75
Figure S68. ^{1}H (400 MHz, $CDCl_3$, 298 K) NMR spectrum of compound **29** ... 76
Figure S69. ^{13}C (101 MHz, $CDCl_3$, 298 K) NMR spectrum of compound **29** ... 76
Figure S70. HR-ESI-MS spectrum of compound **29** ... 76
Figure S71. ^{1}H (400 MHz, $CDCl_3$, 298 K) NMR spectrum of compound **30** ... 77
Figure S72. ^{13}C (101 MHz, $CDCl_3$, 298 K) NMR spectrum of compound **30** ... 77
Figure S73. HR-ESI-MS spectrum of compound **30** ... 77
Figure S74. ^{1}H (400 MHz, DMSO-d^6, 298 K) NMR spectrum of compound **6** ... 78
Figure S75. ^{13}C (101 MHz, DMSO-d^6, 298 K) NMR spectrum of compound **6** ... 78
Figure S76. HR-ESI-MS spectrum of compound **6** ... 78
Figure S77. ^{1}H-^{1}H (COSY, DMSO-d^6, 298 K) 2D NMR spectrum of compound **6** ... 79
Figure S78. ^{1}H-^{13}C (HSQC, DMSO-d^6, 298 K) 2D NMR spectrum of compound **6** ... 79
Figure S79. ^{1}H (400 MHz, MeOD-d^4, 298 K) NMR spectrum of compound **31** ... 80
Figure S80. ^{13}C (101 MHz, MeOD-d^4, 298 K) NMR spectrum of compound **31** ... 80
Figure S81. HR-ESI-MS spectrum of compound **31** ... 80
Figure S82. ^{1}H (400 MHz, $CDCl_3$, 298 K) NMR spectrum of compound **32** ... 81
Figure S83. ^{13}C (101 MHz, $CDCl_3$, 298 K) NMR spectrum of compound **32** ... 81
Figure S84. HR-ESI-MS spectrum of compound **32** ... 81
Figure S85. ^{1}H (400 MHz, $CDCl_3$, 298 K) NMR spectrum of compound **33** ... 82
Figure S86. ^{13}C (101 MHz, $CDCl_3$, 298 K) NMR spectrum of compound **33** ... 82
Figure S87. HR-ESI-MS spectrum of compound **33** ... 82
Figure S88. ^{1}H (400 MHz, MeOD-d^4, 298 K) NMR spectrum of compound **34** ... 83
Figure S89. ^{13}C (101 MHz, MeOD-d^4, 298 K) NMR spectrum of compound **34** ... 83
Figure S90. HR-ESI-MS spectrum of compound **34** ... 83
Figure S91. ^{1}H (400 MHz, DMSO-d^6, 298 K) NMR spectrum of compound **7** ... 84
Figure S92. ^{13}C (101 MHz, DMSO-d^6, 298 K) NMR spectrum of compound **7** ... 84
Figure S93. HR-ESI-MS spectrum of compound **7** ... 84
Figure S94. ^{1}H-^{1}H (COSY, DMSO-d^6, 298 K) 2D NMR spectrum of compound **7** ... 85
Figure S95. ^{1}H-^{13}C (HSQC, DMSO-d^6, 298 K) 2D NMR spectrum of compound **7** ... 85
Figure S96. ^{1}H (400 MHz, $CDCl_3$, 298 K) NMR spectrum of compound **35** ... 86
Figure S97. ^{13}C (101 MHz, $CDCl_3$, 298 K) NMR spectrum of compound **35** ... 86
Figure S98. ^{19}F (376 MHz, $CDCl_3$, 298 K) NMR spectrum of compound **35** ... 86
Figure S99. HR-ESI-MS spectrum of compound **35** ... 87
Figure S100. ^{1}H (400 MHz, $CDCl_3$, 298 K) NMR spectrum of compound **36** ... 87

Figure S101. ^{13}C (101 MHz, $CDCl_3$, 298 K) NMR spectrum of compound **36** 87
Figure S102. ^{19}F (376 MHz, $CDCl_3$, 298 K) NMR spectrum of compound **36** 88
Figure S103. HR-ESI-MS spectrum of compound **36** 88
Figure S104. ^{1}H (400 MHz, $CDCl_3$, 298 K) NMR spectrum of compound **37** 88
Figure S105. ^{13}C (101 MHz, $CDCl_3$, 298 K) NMR spectrum of compound **37** 89
Figure S106. ^{19}F (376 MHz, $CDCl_3$, 298 K) NMR spectrum of compound **37** 89
Figure S107. HR-ESI-MS spectrum of compound **37** 89
Figure S108. ^{1}H (400 MHz, DMSO-d^6, 298 K) NMR spectrum of compound **8** 90
Figure S109. ^{13}C (101 MHz, DMSO-d^6, 298 K) NMR spectrum of compound **8** 90
Figure S110. ^{19}F (376 MHz, DMSO-d^6, 298 K) NMR spectrum of compound **8** 90
Figure S111. HR-ESI-MS spectrum of compound **8** 91
Figure S112. ^{1}H-^{1}H (COSY, DMSO-d^6, 298 K) 2D NMR spectrum of compound **8** 92
Figure S113. ^{1}H-^{13}C (HSQC, DMSO-d^6, 298 K) 2D NMR spectrum of compound **8** 92
Figure S114. ^{1}H (400 MHz, D_2O, 298 K) NMR spectrum of compound **38** 93
Figure S115. ^{13}C (101 MHz, D_2O, 298 K) NMR spectrum of compound **38** 93
Figure S116. HR-ESI-MS spectrum of compound **38** 93
Figure S117. ^{1}H (400 MHz, $CDCl_3$, 298 K) NMR spectrum of compound **39** 94
Figure S118. ^{13}C (101 MHz, $CDCl_3$, 298 K) NMR spectrum of compound **39** 94
Figure S119. HR-ESI-MS spectrum of compound **39** 94
Figure S120. ^{1}H (400 MHz, $CDCl_3$, 298 K) NMR spectrum of compound **40** 95
Figure S121. ^{13}C (101 MHz, $CDCl_3$, 298 K) NMR spectrum of compound **40** 95
Figure S122. HR-ESI-MS spectrum of compound **40** 95
Figure S123. ^{1}H (400 MHz, MeOD-d^4, 298 K) NMR spectrum of compound **41** 96
Figure S124. ^{13}C (101 MHz, MeOD-d^4, 298 K) NMR spectrum of compound **41** 96
Figure S125. HR-ESI-MS spectrum of compound **41** 96
Figure S126. ^{1}H (400 MHz, DMSO-d^6, 298 K) NMR spectrum of compound **9** 97
Figure S127. ^{13}C (101 MHz, DMSO-d^6, 298 K) NMR spectrum of compound **9** 97
Figure S128. HR-ESI-MS spectrum of compound **9** 97
Figure S129. ^{1}H-^{1}H (COSY, DMSO-d^6, 298 K) 2D NMR spectrum of compound **9** 98
Figure S130. ^{1}H-^{13}C (HSQC, DMSO-d^6, 298 K) 2D NMR spectrum of compound **9** 98
Figure S131. ^{1}H (400 MHz, $CDCl_3$, 298 K) NMR spectrum of compound **42** 99
Figure S132. ^{13}C (101 MHz, $CDCl_3$, 298 K) NMR spectrum of compound **42** 99
Figure S133. HR-ESI-MS spectrum of compound **42** 99
Figure S134. ^{1}H (400 MHz, $CDCl_3$, 298 K) NMR spectrum of compound **43** 100

Figure S135. ^{13}C (101 MHz, $CDCl_3$, 298 K) NMR spectrum of compound **43** 100
Figure S136. HR-ESI-MS spectrum of compound **43** 100
Figure S137. ^{1}H (400 MHz, $CDCl_3$, 298 K) NMR spectrum of compound **44** 101
Figure S138. ^{13}C (101 MHz, $CDCl_3$, 298 K) NMR spectrum of compound **44** 101
Figure S139. HR-ESI-MS spectrum of compound **44** 101
Figure S140. ^{1}H (400 MHz, MeOD-d^4, 298 K) NMR spectrum of compound **45** 102
Figure S141. ^{13}C (101 MHz, MeOD-d^4, 298 K) NMR spectrum of compound **45** 102
Figure S142. HR-ESI-MS spectrum of compound **45** 102
Figure S143. ^{1}H (400 MHz, DMSO-d^6, 298 K) NMR spectrum of compound **10** 103
Figure S144. ^{13}C (101 MHz, DMSO-d^6, 298 K) NMR spectrum of compound **10** 103
Figure S145. HR-ESI-MS spectrum of compound **10** 103
Figure S146. ^{1}H-^{1}H (COSY, DMSO-d^6, 298 K) 2D NMR spectrum of compound **10** 104
Figure S147. ^{1}H-^{13}C (HSQC, DMSO-d^6, 298 K) 2D NMR spectrum of compound **10** 104
Figure S148. ^{1}H (400 MHz, DMSO-d^6, 298 K) NMR spectrum of compound **46** 105
Figure S149. ^{13}C (101 MHz, DMSO-d^6, 298 K) NMR spectrum of compound **46** 105
Figure S150. HR-ESI-MS spectrum of compound **46** 105
Figure S151. ^{1}H (400 MHz, $CDCl_3$, 298 K) NMR spectrum of compound **47** 106
Figure S152. ^{13}C (101 MHz, $CDCl_3$, 298 K) NMR spectrum of compound **47** 106
Figure S153. HR-ESI-MS spectrum of compound **47** 106
Figure S154. ^{1}H (400 MHz, $CDCl_3$, 298 K) NMR spectrum of compound **48** 107
Figure S155. ^{13}C (101 MHz, $CDCl_3$, 298 K) NMR spectrum of compound **48** 107
Figure S156. HR-ESI-MS spectrum of compound **48** 107
Figure S157. ^{1}H (400 MHz, MeOD-d^4, 298 K) NMR spectrum of compound **49** 108
Figure S158. ^{13}C (101 MHz, MeOD-d^4, 298 K) NMR spectrum of compound **49** 108
Figure S159. HR-ESI-MS spectrum of compound **49** 108
Figure S160. ^{1}H (400 MHz, DMSO-d^6, 298 K) NMR spectrum of compound **11** 109
Figure S161. ^{13}C (101 MHz, DMSO-d^6, 298 K) NMR spectrum of compound **11** 109
Figure S162. HR-ESI-MS spectrum of compound **11** 109
Figure S163. ^{1}H-^{1}H (101 MHz, DMSO-d^6, 298 K) 2D NMR spectrum of compound **11** 110
Figure S164. ^{1}H-^{13}C (101 MHz, DMSO-d^6, 298 K) 2D NMR spectrum of compound **11** 110
Figure S165. ^{1}H (400 MHz, $CDCl_3$, 298 K) NMR spectrum of compound **50** 111
Figure S166. ^{13}C (101 MHz, $CDCl_3$, 298 K) NMR spectrum of compound **50** 111
Figure S167. HR-ESI-MS spectrum of compound **50** 111
Figure S168. ^{1}H (400 MHz, $CDCl_3$, 298 K) NMR spectrum of compound **51** 112

Figure S169. ^{13}C (101 MHz, $CDCl_3$, 298 K) NMR spectrum of compound **51** 112
Figure S170. HR-ESI-MS spectrum of compound **51** 112
Figure S171. ^{1}H (400 MHz, $CDCl_3$, 298 K) NMR spectrum of compound **52** 113
Figure S172. ^{13}C (101 MHz, $CDCl_3$, 298 K) NMR spectrum of compound **52** 113
Figure S173. HR-ESI-MS spectrum of compound **52** 113
Figure S174. ^{1}H (400 MHz, DMSO-d^6, 298 K) NMR spectrum of compound **12** 114
Figure S175. ^{13}C (101 MHz, DMSO-d^6, 298 K) NMR spectrum of compound **12** 114
Figure S176. HR-ESI-MS spectrum of compound **12** 114
Figure S177. ^{1}H-^{1}H (COSY, DMSO-d^6, 298 K) 2D NMR spectrum of compound **12** 115
Figure S178. ^{1}H-^{13}C (HSQC, DMSO-d^6, 298 K) 2D NMR spectrum of compound **12** 115
Figure S179. ^{1}H (400 MHz, MeOD-d^4, 298 K) NMR spectrum of compound **53** 116
Figure S180. ^{13}C (101 MHz, MeOD-d^4, 298 K) NMR spectrum of compound **53** 116
Figure S181. ^{19}F (376 MHz, MeOD-d^4, 298 K) NMR spectrum of compound **53** 116
Figure S182. HR-ESI-MS spectrum of compound **53** 117
Figure S183. ^{1}H (400 MHz, MeOD-d^4, 298 K) NMR spectrum of compound **54** 117
Figure S184. ^{13}C (101 MHz, MeOD-d^4, 298 K) NMR spectrum of compound **54** 117
Figure S185. ^{19}F (376 MHz, MeOD-d^4, 298 K) NMR spectrum of compound **54** 118
Figure S186. HR-ESI-MS spectrum of compound **54** 118
Figure S187. ^{1}H (400 MHz, MeOD-d^4, 298 K) NMR spectrum of compound **55** 118
Figure S188. ^{13}C (101 MHz, MeOD-d^4, 298 K) NMR spectrum of compound **55** 119
Figure S189. ^{19}F (376 MHz, MeOD-d^4, 298 K) NMR spectrum of compound **55** 119
Figure S190. HR-ESI-MS spectrum of compound **55** 119
Figure S191. ^{1}H (400 MHz, DMSO-d^6, 298 K) NMR spectrum of compound **13** 120
Figure S192. ^{13}C (126 MHz, DMSO-d^6, 298 K) NMR spectrum of compound **13** 120
Figure S193. ^{19}F (376 MHz, DMSO-d^6, 298 K) NMR spectrum of compound **13** 120
Figure S194. HR-ESI-MS spectrum of compound **13** 121
Figure S195. ^{1}H-^{1}H (COSY, DMSO-d^6, 298 K) 2D NMR spectrum of compound **13** 122
Figure S196. ^{1}H-^{13}C (HSQC, DMSO-d^6, 298 K) 2D NMR spectrum of compound **13** 122
Figure S197. ^{1}H (400 MHz, DMSO-d^6, 298 K) NMR spectrum of compound **56** 123
Figure S198. ^{13}C (101 MHz, DMSO-d^6, 298 K) NMR spectrum of compound **56** 123
Figure S199. HR-ESI-MS spectrum of compound **56** 123
Figure S200. ^{1}H (400 MHz, $CDCl_3$, 298 K) NMR spectrum of compound **57** 124
Figure S201. ^{13}C (101 MHz, $CDCl_3$, 298 K) NMR spectrum of compound **57** 124
Figure S202. HR-ESI-MS spectrum of compound **57** 124

Figure S203. ^{1}H (400 MHz, MeOD-d^4, 298 K) NMR spectrum of compound **58** 125
Figure S204. ^{13}C (126 MHz, $CDCl_3$, 298 K) NMR spectrum of compound **58** 125
Figure S205. HR-ESI-MS spectrum of compound **58** 125
Figure S206. ^{1}H (400 MHz, DMSO-d^6, 298 K) NMR spectrum of compound **14** 126
Figure S207. ^{13}C (126 MHz, DMSO-d^6, 298 K) NMR spectrum of compound **14** 126
Figure S208. HR-ESI-MS spectrum of compound **14** 126
Figure S209. ^{1}H-^{13}C (HSQC, DMSO-d^6, 298 K) 2D NMR spectrum of compound **14** 127
Figure S210. ^{1}H (400 MHz, $CDCl_3$, 298 K) NMR spectrum of compound **59** 134
Figure S211. ^{1}H (500 MHz, DMSO-d^6, 298 K) NMR spectrum of compound **60** 134
Figure S212. ^{13}C (126 MHz, DMSO-d^6, 298 K) NMR spectrum of compound **60** 134
Figure S213. HR-ESI-MS spectrum of compound **60** 135
Figure S214. ^{1}H (500 MHz, $CDCl_3$, 298 K) NMR spectrum of compound **61** 135
Figure S215. ^{1}H (500 MHz, DMSO-d^6, 298 K) NMR spectrum of compound **62** 135
Figure S216. ^{13}C (126 MHz, DMSO-d^6, 298 K) NMR spectrum of compound **62** 136
Figure S217. HR-ESI-MS spectrum of compound **62** 136
Figure S218. ^{1}H-^{1}H (COSY, DMSO-d^6, 298 K) 2D NMR spectrum of compound **62** 137
Figure S219. ^{1}H-^{13}C (HSQC, DMSO-d^6, 298 K) 2D NMR spectrum of compound **62** 137
Figure S220. ^{1}H (500 MHz, DMSO-d^6, 298 K) NMR spectrum of compound **63** 138
Figure S221. ^{13}C (126 MHz, DMSO-d^6, 298 K) NMR spectrum of compound **63** 138
Figure S222. HR-ESI-MS spectrum of compound **63** 138
Figure S223. ^{1}H-^{1}H (COSY, DMSO-d^6, 298 K) 2D NMR spectrum of compound **63** 139
Figure S224. ^{1}H-^{13}C (HSQC, DMSO-d^6, 298 K) 2D NMR spectrum of compound **63** 139
Figure S225. ^{1}H (400 MHz, $CDCl_3$, 298 K) NMR spectrum of compound **64** 140
Figure S226. ^{1}H (500 MHz, DMSO-d^6, 298 K) NMR spectrum of compound **65** 140
Figure S227. ^{13}C (126 MHz, DMSO-d^6, 298 K) NMR spectrum of compound **65** 140
Figure S228. ^{1}H (400 MHz, DMSO-d^6, 298 K) NMR spectrum of compound **66** 141
Figure S229. ^{13}C (126 MHz, DMSO-d^6, 298 K) NMR spectrum of compound **66** 141
Figure S230. ^{1}H-^{13}C (HSQC, DMSO-d^6, 298 K) 2D NMR spectrum of compound **66** 142
Figure S231. HR-ESI-MS spectrum of compound **66** 142
Figure S232. ^{1}H (500 MHz, DMSO-d^6, 298 K) NMR spectrum of compound **67** 143
Figure S233. ^{13}C (126 MHz, DMSO-d^6, 298 K) NMR spectrum of compound **67** 143
Figure S234. HR-ESI-MS spectrum of compound **67** 143

Figure S235. Data on the one-pot ^{89}Zr-radiolabeling and photo-induced protein conjugation between the photoactivatable non-PEGylated DFO derivatives, and the monovalent, monoclonal scFv-Fc engineered antibody fragment onartuzumab. 144
Figure S236. Reverse-phase HPLC chromatograms for (A) the ^{nat}Zr and (B) the ^{89}Zr complexes formed with compounds **1** to **14**.. 145
Figure S237. Characterization data for the radiochemical synthesis of [^{89}Zr]ZrDFO-PEG$_3$-*p*-azepin-HSA using DFO-PEG$_3$-*para*-ArN$_3$ (**1**) (irradiation at 395 nm). (A) Radio-iTLC chromatograms, (B) analytical PD-10-SEC profiles, and (C) SEC-HPLC chromatograms of the crude and purified product (*: aggregated protein, Δ: radiolabeled small molecules).[5] .. 147
Figure S238. Characterization data for the radiochemical synthesis of [^{89}Zr]ZrDFO-PEG$_3$-*p*-azepin-HSA using DFO-PEG$_3$-*para*-ArN$_3$ (**1**) (irradiation in the visible region at 450 nm). (A) Radio-iTLC chromatograms, (B) analytical PD-10-SEC profiles, and (C) SEC-HPLC chromatograms of the crude and purified product (*: aggregated protein, Δ: radiolabeled small molecules). 147
Figure S239. Characterization data for the radiochemical synthesis of [^{89}Zr]ZrDFO-PEG$_3$-*m*-azepin-HSA using DFO-PEG$_3$-*meta*-ArN$_3$ (**2**). (A) Radio-iTLC chromatograms, (B) analytical PD-10-SEC profiles, and (C) SEC-HPLC chromatograms of the crude and purified product (*: aggregated protein, Δ: radiolabeled small molecules). 148
Figure S240. Characterization data for the radiochemical synthesis of [^{89}Zr]ZrDFO-PEG$_3$-*o*-azepin-HSA using DFO-PEG$_3$-*ortho*-ArN$_3$ (**3**). (A) Radio-iTLC chromatograms, (B) analytical PD-10-SEC profiles, and (C) SEC-HPLC chromatograms of the crude and purified product (*: aggregated protein, Δ: radiolabeled small molecules). 148
Figure S241. Characterization data for the radiochemical synthesis of [^{89}Zr]ZrDFO-PEG$_3$-*p*-Et-azepin-HSA using DFO-PEG$_3$-*p*-EtArN$_3$ (**4**). (A) Radio-iTLC chromatograms, (B) analytical PD-10-SEC profiles, and (C) SEC-HPLC chromatograms of the purified product (blue) and UV/vis (280 nm; red) of the protein. (*: aggregated protein, Δ: radiolabeled small molecules).[5] 149
Figure S242. Characterization data for the radiochemical synthesis of [^{89}Zr]ZrDFO-PEG$_3$-*m*-OMe-azepin-HSA using DFO-PEG$_3$-*m*-OMe-ArN$_3$ (**5**). (A) Radio-iTLC chromatograms, (B) analytical PD-10-SEC profiles, and (C) SEC-HPLC chromatograms of the crude and purified product (*: aggregated protein, Δ: radiolabeled small molecules). 149
Figure S243. Characterization data for the radiochemical synthesis of [^{89}Zr]ZrDFO-PEG$_3$-*m*-Cl-azepin-HSA using DFO-PEG$_3$-*m*-Cl-ArN$_3$ (**6**). (A) Radio-iTLC chromatograms, (B)

analytical PD-10-SEC profiles, and (C) SEC-HPLC chromatograms of the crude and purified product (*: aggregated protein, Δ: radiolabeled small molecules).. 150
Figure S244. Characterization data for the radiochemical synthesis of [^{89}Zr]ZrDFO-PEG$_3$-*m*-NO$_2$-azepin-HSA using DFO-PEG$_3$-*m*-NO$_2$-ArN$_3$ (**7**). (A) Radio-iTLC chromatograms, (B) analytical PD-10-SEC profiles, and (C) SEC-HPLC chromatograms of the crude and purified product (*: aggregated protein, Δ: radiolabeled small molecules).. 150
Figure S245. Characterization data for the radiochemical synthesis of [^{89}Zr]ZrDFO-PEG$_3$-2,4-pyridyl-HSA using DFO-PEG$_3$-2,4-pyridyl-ArN$_3$ (**9**). (A) Radio-iTLC chromatograms, (B) analytical PD-10-SEC profiles, and (C) SEC-HPLC chromatograms of the crude and purified product (*: aggregated protein, Δ: radiolabeled small molecules)........................... 151
Figure S246. Characterization data for the radiochemical synthesis of [^{89}Zr]ZrDFO-PEG$_3$-2,3-naphthyl-azepin-HSA using DFO-PEG$_3$-2,3-naphthyl-ArN$_3$ (**10**). (A) Radio-iTLC chromatograms, (B) analytical PD-10-SEC profiles, and (C) SEC-HPLC chromatograms of the crude and purified product (*: aggregated protein, Δ: radiolabeled small molecules)..... 151
Figure S247. Characterization data for the radiochemical synthesis of [^{89}Zr]ZrDFO-PEG$_3$-2,6-naphthyl-HSA using DFO-PEG$_3$-2,6-naphthyl-ArN$_3$ (**11**). (A) Radio-iTLC chromatograms, (B) analytical PD-10-SEC profiles, and (C) SEC-HPLC chromatograms of the crude and purified product (*: aggregated protein, Δ: radiolabeled small molecules)..... 152
Figure S248. Characterization data for the radiochemical synthesis of [^{89}Zr]ZrDFO-PEG$_3$-BP-HSA using DFO-PEG$_3$-BP (**12**). (A) Radio-iTLC chromatograms, (B) analytical PD-10-SEC profiles, and (C) SEC-HPLC chromatograms of the crude and purified product (*: aggregated protein, Δ: radiolabeled small molecules). 152
Figure S249. Characterization data for the radiochemical synthesis of [^{89}Zr]ZrDFO-PEG$_3$-DA-HSA using DFO-PEG$_3$-DA (**13**). (A) Radio-iTLC chromatograms, (B) analytical PD-10-SEC profiles, and (C) SEC-HPLC chromatograms of the crude and purified product (*: aggregated protein, Δ: radiolabeled small molecules). 153
Figure S250. Characterization data for the radiochemical synthesis of [^{89}Zr]ZrDFO-PEG$_3$-Tz-HSA using DFO-PEG$_3$-Tz (**14**). (A) Radio-iTLC chromatograms, (B) analytical PD-10-SEC profiles, and (C) SEC-HPLC chromatograms of the crude and purified product (*: aggregated protein, Δ: radiolabeled small molecules).. 153
Figure S251. Proposed mechanism of photochemical and thermal activation, intramolecular rearrangement, and nucleophilic attack at model aryl azide compounds.............................. 155

Figure S252. DFT calculated (PW6B95/6-311++G(d,p)/PCM) reaction coordinate showing the reaction of unsubstituted phenylazide, PhN_3 (non-substituted model **H**) with methylamine ($MeNH_2$). 164
Figure S253. DFT calculated (PW6B95/6-311++G(d,p)/PCM) reaction coordinate showing the reaction of $CH_3C(O)NH$-ArN_3 aryl azide (model inverted amide) with methylamine. ... 165
Figure S254. DFT calculated (PW6B95/6-311++G(d,p)/PCM) reaction coordinate showing the reaction of *para*-ArN_3 (model **1**) with methylamine. 166
Figure S255. DFT calculated (PW6B95/6-311++G(d,p)/PCM) reaction coordinate showing the reaction of *meta*-ArN_3 (model **2** pathway a) with methylamine. 167
Figure S256. DFT calculated (PW6B95/6-311++G(d,p)/PCM) reaction coordinate showing the reaction of *meta*-ArN_3 (model **2** pathway b) with methylamine. 168
Figure S257. DFT calculated (PW6B95/6-311++G(d,p)/PCM) reaction coordinate showing the reaction of *ortho*-ArN_3 (model **3** pathway a) with methylamine. 169
Figure S258. DFT calculated (PW6B95/6-311++G(d,p)/PCM) reaction coordinate showing the reaction of *ortho*-ArN_3 (model **3** pathway b) with methylamine. 170
Figure S259. DFT calculated (PW6B95/6-311++G(d,p)/PCM) reaction coordinate showing the reaction of *para*-$EtArN_3$ (model **4**) with methylamine. 171
Figure S260. DFT calculated (PW6B95/6-311++G(d,p)/PCM) reaction coordinate showing the reaction of *ortho*-MeO-ArN_3 (model **5** pathway a) with methylamine. 172
Figure S261. DFT calculated (PW6B95/6-311++G(d,p)/PCM) reaction coordinate showing the reaction of *ortho*-MeO-ArN_3 (model **5** pathway b) with methylamine. 173
Figure S262. DFT calculated (PW6B95/6-311++G(d,p)/PCM) reaction coordinate showing the reaction of *ortho*-Cl-ArN_3 (model **6** pathway a) with methylamine. 174
Figure S263. DFT calculated (PW6B95/6-311++G(d,p)/PCM) reaction coordinate showing the reaction of *ortho*-Cl-ArN_3 (model **6** pathway b) with methylamine. 175
Figure S264. DFT calculated (PW6B95/6-311++G(d,p)/PCM) reaction coordinate showing the reaction of *ortho*-NO_2-ArN_3 (model **7** pathway a) with methylamine. 176
Figure S265. DFT calculated (PW6B95/6-311++G(d,p)/PCM) reaction coordinate showing the reaction of *ortho*-NO_2-ArN_3 (model **7** pathway b) with methylamine. 177
Figure S266. DFT calculated (PW6B95/6-311++G(d,p)/PCM) reaction coordinate showing the reaction of 4F-*p*-ArN_3 (model **8**) with methylamine. 178
Figure S267. DFT calculated (PW6B95/6-311++G(d,p)/PCM) reaction coordinate showing the reaction of 2,4-pyridyl-ArN_3 (model **9** pathway a) with methylamine. 179

Figure S268. DFT calculated (PW6B95/6-311++G(d,p)/PCM) reaction coordinate showing the reaction of 2,4-pyridyl-ArN_3 (model **9** pathway b) with methylamine. 180
Figure S269. DFT calculated (PW6B95/6-311++G(d,p)/PCM) reaction coordinate showing the reaction of 2,3-naphthyl-ArN_3 (model **10** pathway a) with methylamine. 181
Figure S270. DFT calculated (PW6B95/6-311++G(d,p)/PCM) reaction coordinate showing the reaction of 2,3-naphthyl-ArN_3 (model **10** pathway b) with methylamine. 182
Figure S271. DFT calculated (PW6B95/6-311++G(d,p)/PCM) reaction coordinate showing the reaction of 2,6-naphthyl-ArN_3 (model **11** pathway a) with methylamine. 183
Figure S272. DFT calculated (PW6B95/6-311++G(d,p)/PCM) reaction coordinate showing the reaction of 2,6-naphthyl-ArN_3 (model **11** pathway b) with methylamine. 184

Methods and Materials

General

All reagents and anhydrous solvents were purchased from commercial sources [Sigma-Aldrich (St. Louis, USA), Merck (Darmstadt, Germany), Tokyo Chemical Industry (Eschborn, Germany), or Fluorochem Ltd (Hadfield, UK)] and were used without any further purification unless otherwise stated. All aqueous reactions were carried out using MilliQ H_2O (>18.2 M MΩ·cm at 25 ºC, Merck, Darmstadt, Germany). All anhydrous reactions were carried out in oven-dried glassware under an inert atmosphere. Reactions (where possible) were monitored using thin layer chromatography (TLC) analysis on aluminium plates coated with Silica Gel 60 F_{254} (E. Merck), and were visualized by short-wave ultra-violet irradiation (254 nm; where applicable), stained with ninhydrin in EtOH or propargyl alcohol and Cu(I)Br in EtOH, followed by charring at ~200 ºC.[1] Purification was carried out by flash chromatography on a column of silica gel 60 (0.040-0.063 mm) or by reversed-phase C18 column chromatography using a Teledyne Isco CombiFlash® Rf+ Lumen flash chromatography system fitted with RediSep Rf Gold® reversed-phase C18 columns (5 to 50 g), eluting in a gradient of 0 to 100% of solvent B (MeOH with 0.1% TFA added). Solvent A: MilliQ H_2O with 0.1% TFA added. Evaporation of solvents was performed under reduced pressure by using a rotary evaporator (Rotavapor R-300, Büchi Labortechnik AG, Flawil, Switzerland).

^{1}H NMR, ^{13}C{^{1}H} NMR and ^{19}F NMR experiments were performed by using deuterated solvents (Sigma-Aldrich, St. Louis, USA) on a Bruker AV-400 (^{1}H: 400 MHz, ^{13}C: 101 MHz, ^{18}F: 376 MHz) or a Bruker AV-500 (^{1}H: 500 MHz, ^{13}C: 126 MHz) spectrometer. Chemical shifts (δ) for ^{1}H, ^{13}C and ^{18}F spectra are reported in parts per million (ppm) and are relative to the residual solvent peak (or an internal reference). Coupling constants (*J*) are reported in Hz. Peak multiplicities are abbreviated as follows: s (singlet), d (doublet), dd (doublet of doublets), t (triplet), q (quartet), quint (quintet), m (multiplet), and br s (broad singlet). Two-dimensional ^{1}H-^{1}H correlation spectroscopy (COSY), ^{13}C heteronuclear single quantum coherence (HSQC) and ^{13}C heteronuclear multiple bond correlation (HMBC) NMR experiments were also performed to aid in the assignment of the ^{1}H and ^{13}C spectra. High-resolution electrospray ioniation mass spectrometry (HR-ESI-MS) was performed in either positive or negative ionisation mode (as indicated) using a Bruker MaXis QTOF-MS instrument (Bruker Daltronics GmbH, Bremen, Germany) and all samples were measured by the mass spectrometry service at the Department of Chemistry, University of Zurich.

Analytical high-performance liquid chromatography (HPLC) experiments were performed using a Hitachi Chromaster Ultra Rs system fitted with a reversed-phase VP 250/4 Nucleodur C18 HTec (4 mm ID × 250 mm, 5µm, Macherey-Nagel, Düren, Germany) column. This system was also fitted to a FlowStar^{2} LB 514 radioactivity detector (Berthold Technologies, Zug, Switzerland) equipped with a 20 µL PET cell (MX-20-6, Berthold Technologies) for analyzing radiochemical reactions. Size-exclusion high-performance liquid chromatography (SEC-HPLC) experiments (for protein samples) were performed using a Rigol HPLC system (Contrec AG, Dietikon, Switzerland) equipped with an Enrich SEC 650 size-exclusion column (24 mL volume, 10 mm ID × 300 mm, Bio-Rad Laboratories, Basel, Switzerland). Electronic

absorption was measured at 280 nm. Purities of synthetic intermediates after chromatographic purification were judged to be >90% by analysis of ^{1}H and $^{13}C\{^{1}H\}$ NMR spectra. Purities of final compounds were ≥95% (NMR or HPLC analysis), after reverse-phase C18 chromatography.

Photochemistry

Photochemical conjugation experiments were performed in transparent glass vials using an ultra-violet light-emitting diode (LED; 395 nm). The LED intensity was adjusted using a digital UV-LED controller (Opsytec Dr. Gröbel GmbH, Ettlingen, Germany), where 100% corresponded to a power of approximately 355 mW at 395 nm. LED intensity was measured by using a S470C Thermal Power Sensor Head Volume Absorber, 0.25 – 10.6 µm, 0.1 mW – 5W, Ø15 mm. The LED (395 nm) had a maximum emission intensity at 389.9 nm (FWHM of 9.1 nm). Photochemical reactions were stirred gently by adding a small magnetic stir bar to the reaction vial and employing a slow stirring rate (< 100 rpm) to avoid potential mechanical damage to the protein. Detailed procedures and reaction times are indicated in the experimental section. The temperature of all photochemical conjugation reactions was 23 ± 2 ºC (ambient conditions).

Radioactivity and radioactive measurements

All instruments for measuring radioactivity were calibrated and maintained in accordance with previously reported routine quality control procedures.[2] $[^{89}Zr][Zr(C_2O_4)_4]^{4-}$ was obtained as a solution in ~1.0 M aqueous oxalic acid from PerkinElmer (Boston, MA, manufactured by the BV Cyclotron VU, Amsterdam, The Netherlands) and was used without further purification. Radioactive reactions were monitored by using instant thin-layer chromatography (radio-iTLC). Glass-fibre iTLC plates impregnated with silica-gel (iTLC-SG, Agilent Technologies) were developed by using an aqueous mobile phase containing DTPA (50 mM, pH7.1) and were analysed on a radio-TLC detector (SCAN-RAM, LabLogic Systems Ltd, Sheffield, United Kingdom). Radiochemical conversion (RCC) was determined by integrating the data obtained by the radio-TLC plate reader and determining both the percentage of radiolabeled product from the peak present at the baseline ($R_f = 0.0$) and 'free' ^{89}Zr at the solvent front ($R_f = 1.0$; present in the analyses as $[^{89}Zr][Zr(DTPA)]^{-}$). Integration and data analyses were performed by using the software Laura version 5.0.4.29 (LabLogic). Appropriate background and decay corrections were applied throughout.

Radiochemical purities (RCPs) of labelled protein samples were determined by size-exclusion chromatography (SEC) by using two different columns and techniques. The first technique (SEC-HPLC) used an automated size-exclusion column (Bio-Rad Laboratories, ENrich SEC 70, 10 ± 2 µm, 10 mm ID × 300 mm) connected to a Rigol HPLC system (Contrec AG, Dietikon, Switzerland) equipped with a UV/visible detector (absorption measured at 220, 254 and 280 nm) as well as a radioactivity detector (FlowStar^2 LB 514, Berthold Technologies, Zug, Switzerland). Isocratic elution with phosphate buffered saline (PBS, pH7.4) was used. The second method used a manual procedure involving size-exclusion column chromatography and a PD-10 desalting column (Sephadex G-25 resin, 85-260 µm, 14.5 mm ID × 50 mm, >30 kDa, GE Healthcare). For analytical procedures, PD-10 columns were eluted with PBS. A total of 40 × 200 µL fractions were collected up to a final elution volume of 8 mL. Note that the loading/dead-volume of the PD-10 columns is precisely 2.5 mL which was discarded prior to aliquot collection. For quantification of radioactivity, each fraction was measured on a gamma counter (HIDEX Automatic Gamma Counter, Hidex AMG, Turku, Finland) using an energy window between 480 – 558 keV for ^{89}Zr (511 keV emission) and a counting time of 30 s. Appropriate background and decay corrections were applied throughout. PD-10 SEC columns were also used for preparative purification and reformulation of radiolabeled products (in sterile PBS; pH 7.4) by collecting a fraction of the eluate corresponding to the high molecular weight protein (>30 kDa high-purity fraction eluted in the range 0.0 mL to 1.6 mL as indicated for each experiment). Decay-corrected, isolated radiochemical yields (RCYs) of the labeled products were determined by using a dose calibrator (ISOMED 2010 Activimeter, Nuklear-Medizintechnik Dresden GmbH, Germany) and values were corrected for minor differences in RCP of the isolated material (as determined by SEC-HPLC).

^{89}Zr-radioactive stocks

Stock solutions of $[^{89}Zr][Zr(C_2O_4)_4]^{4-}$ were prepared on several occasions using the same procedure. As an example, a stock solution of $[^{89}Zr][Zr(C_2O_4)_4]^{4-}$ was prepared by adding ^{89}Zr

radioactivity from the source (224.0 MBq, 200 µL in ~1.0 M aqueous oxalic acid; PerkinElmer) to an Eppendorf tube. The solution was neutralized by the addition of aliquots of 1.0 M Na_2CO_3(aq) (total volume of 160 µL added, final pH ~8.0, final volume ~360 µL, final activity = 214.7 MBq). Caution: Acid neutralization with Na_2CO_3 releases CO_2(g) and care should be taken to ensure that no radioactivity escapes the microcentrifuge tube. After CO_2 evolution ceased, the neutralized solution was allowed to equilibrate to room temperature and then several different reactions were performed by using the same stock solutions.

^{89}Zr-Radiolabeling of ligands

*Radiosynthesis and characterisation of [^{89}Zr]-ZrDFO-PEG$_3$-complexes (^{89}Zr-**1**$^+$ to ^{89}Zr-**14**$^+$)*

Radiolabeling reactions to prepare [^{89}Zr]ZrDFO-PEG$_3$-ligands (^{89}Zr-**1**$^+$ to ^{89}Zr-**14**$^+$) were accomplished by the addition of an aliquot of neutralized [^{89}Zr][$Zr(C_2O_4)_4$]$^{4-}$ stock solution (~3 MBq) to an aqueous solution of DFO-PEG$_3$-ligand (e.g. for **1**, 11 µL of 3.28 mg mL^{-1} stock [10% DMSO in H_2O]) with a total reaction volume of 50 µL. The geometry of all reactions was identical. The reactions were monitored by radio-iTLC (50 mM aqueous DTPA at pH 7.0) and complexation was found to be complete in less than 10 min at 23 ºC giving a radiochemical conversion (RCC) >99% (R_f = 0.06 – 0.17) for all compounds. The products were characterized by analytical HPLC (Nucleodur EC 250/4, 4 mm ID × 250 mm (C18 HTec, 5 µm) at a flow rate of 1 mL min^{-1} with a linear gradient of A (H_2O containing 0.1% TFA) and B (MeOH containing 0.1% TFA): t = 0 min, 60% A; t = 11 min, 5% A. Note: the UV/vis detector and radioactivity detector were arranged serially with an offset time of approximately 0.10-0.30 min (depending on temperature). The identity of the radiolabeled compounds (^{89}Zr-**1**$^+$ to **14**$^+$) was confirmed by co-injection with an authenticated sample of non-radiolabeled complex ^{nat}Zr-**1**$^+$ to ^{nat}Zr-**14**$^+$.

^{89}Zr-photoradiolabeling of protein

Photochemical conjugation to HSA

A solution of HSA (77.25 mg mL^{-1}, 30 µL, 33.5 nmol, MW = 69,080 g mol^{-1}) was incubated with DFO-PEG$_3$-compound (**1** – **14**) (e.g. for **1**, 3.26 mM, 11 µL, 35.9 nmol) in Chelex-treated metal ion free water (94 µL) at pH 8 – 8.4 (pH was adjusted with the addition of 0.1 M aqueous $NaHCO_3$ or 0.1 M aqueous HCl as required) and ^{89}Zr(oxalate) (7.942 MBq, 15 µL) to give a total reaction volume of 150 µL. In each reaction, the final concentration of HSA protein was 224 µM and the concentration of the photoactivatable DFO ligand (**1** – **14**) was ~235 µM (representing an initial protein-to-DFO ratio of 1:1.05). Reactions were irradiated at 100% LED intensity (395 nm) for 15 min. After the irradiation, aliquots (100 µL) of the crude reaction mixtures were purified by preparative PD-10 SEC (collecting the 0.0 – 1.6 mL high molecular weight fraction using sterile PBS as an eluent). Crude and purified aliquots were analysed by using analytical radio-iTLC, manual PD-10 SEC and automated SEC-HPLC. All photoradiolabeling experiments were performed in triplicate using independent experiments and by at least two different senior scientists to avoid systematic user-dependent errors.

Statistical analysis

Where appropriate, data were analysed by the unpaired, two-tailed Student's t-test. Differences at the 95% confidence level (P-value <0.05) were considered to be statistically significant. Data

analysis was performed using Microsoft Excel for MAC (version 16.16.10) and GraphPad Prism 7 for MAC OS X (version 7.0e).

Density functional theory calculations

All calculations were conducted using density functional theory (DFT) as implemented in the Gaussian16 Revision A.03 suite of *ab initio* quantum chemistry programs.[3] Calculations were performed by using the unrestricted uPW6B95 exchange-correlation functionals and the triple-ζ 6-311++G(d,p) basis set by Pople and co-workers. Normal self-consistent field (SCF) and geometry convergence criteria were employed throughout. No symmetry constraints were used, and all optimized structures correspond to the C_1 symmetric species. All structures were optimized in solution phase using a polarizable continuum model (PCM) without symmetry constraints. Solvated phase calculations were implemented by using the SCRF keyword with default parameters and selecting water as the solvent (dielectric constant, $\varepsilon = 78.3553$). Harmonic frequency analysis based on analytical second derivative was used to characterize the optimized structures as local minima or first order saddle points (transition states) on the potential energy surface. The nature of all transition state structures was confirmed by performing intrinsic reaction coordinate (IRC) calculations to ensure that the saddle point connected the correct local minima. The choice of solvation model reflects the aqueous phase conditions employed in the photoradiochemical synthesis of protein-conjugates. Optimized structures and molecular orbitals were analyzed by using Chemcraft (version 1.8, build 536b). To simplify calculations, model structures were used in which the desferrioxamine connection to the photoactivatable group was replaced by either CH_3-, H- or $CH_3NHC(O)$- group. Excited state calculations employed the CIS methodology ($n > 3$ states) where structures were fully optimized (including solvent field relaxation) and all excited state properties were calculated by using PW6B95/6-311++G(d,p)/PCM methodology employing the current density.

For calculations on the nitrene species, structures were optimized without symmetry constraints (C_1 symmetric) but for convenience, the different electronic states of the nitrene are referred to using their pseudo-C_s symmetric symmetry characters. For closed-shell singlet species, the calculations converged to the restricted result. Calculations on the aryl nitrene species in the open-shell singlet (pseudo-^{1}A'' state) or triplet (pseudo-^{3}A'' state) required the unrestricted formalism. Ground state geometry optimizations of aryl nitrene (ArN) specifying the singlet state generally converged to the higher energy ^{1}A' state with a $(p_y)^2$ electronic configuration where both electrons occupied the in-plane p_y orbital (with respect to the plane of the aryl ring). Checks on the stability of the wavefunction confirmed that this state was unstable with respect to both the lower energy open-shell ^{1}A'' state $(p_x)^1(p_y)^1$ configuration and also the lower energy pseudo-^{3}A'' state. Geometry optimisation of the aryl nitrene in the triplet state was accomplished by specifying the triple spin state in the root section. Optimisation to the lowest energy open-shell singlet pseudo-^{1}A'' state required control over the molecular orbital population using the Guess=(always,alter) keyword. The initial orbital population guess placed both the alpha and beta electrons in the highest occupied molecular orbitals (HOMOs), which corresponded with the in-plane p_y orbital on the N atom. In both the alpha and beta orbital manifolds, the lowest unoccupied molecular orbital (LUMO) corresponded to the out-of-plane p_x orbital on the N atom. Convergence to the pseudo-^{1}A'' state was achieved by switching the

orbital occupancy in the beta manifold. Note that spin contamination was observed in the optimized open-shell (pseudo-$^1A_2$ state) calculations with the spin expectation value of $\langle S^2 \rangle$ = 1.000 indicating a 50:50 mixture with a triplet contamination. Automatic spin annihilation reduced this value to ~0.25 but in all cases the energetics of the pseudo-$^1A''$ state was estimated by using the sum method reported by Ziegler *et al*.[4] Equivalent methods were employed in the calculations on the other photoactivatable species studied in this work.

Synthesis and Characterisation Data

General Procedures

General Procedure A: Diazo-transfer of aryl amine derivatives.

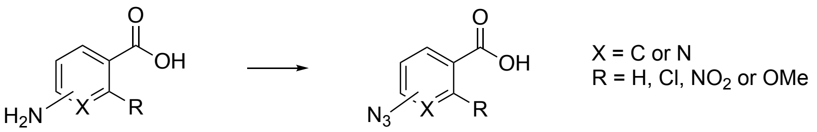

To a cooled (0 °C) suspension of aminobenzoic acid derivative (1.0 equiv) in conc. H_2SO_4 (1.5 mL per mmol) and AcOH (1.5 mL per mmol), a solution of $NaNO_2$ (1.1 equiv) in H_2O (1 mL) was added dropwise before the reaction mixture was allowed to warm to rt and then stirred for 3 h. After this time, the reaction mixture was cooled to 0 °C and a solution of NaN_3 (1.1 equiv) in H_2O (1mL) was added dropwise and the reaction was stirred for a further 1 h. The reaction mixture was then extracted with EtOAc (3 × 50 mL), the organic layer was separated and dried over anh. $MgSO_4$, filtered, and concentrated under reduced pressure to give the desired azido products as off-white to orange solids (27 to 99% yield).

General Procedure B: PEGylation of photoactivatable carboxylic acid derivatives via HATU mediated amide coupling.

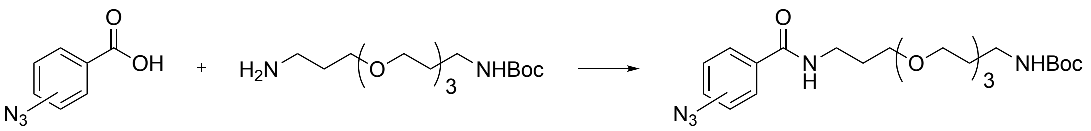

In a round bottom flask equipped with N_2, the photoactivatable carboxylic acid derivative (1.0 equiv) was dissolved in anh. DMF (5 mL per mmol). HATU (1.5 equiv) and DIPEA (4 equiv) were then added and the reaction mixture was stirred for 30 min. Then, a solution of *N*-Boc-4,7,10-trioxa-1,13-tridecanediamine (1.5 equiv) in anh. DMF (3 mL per mmol) was added dropwise and the reaction mixture was protected from light and stirred for 16 h. The solvent was removed under reduced pressure and the crude residue was purified by reverse-phase flash column chromatography (C18; H_2O with 0.1% TFA to 100 % MeOH with 0.1% TFA) to give the corresponding PEG_3 functionalized azido derivatives as colorless to pale yellow oils (8 to 88% yield).

General Procedure C: TFA mediated deprotection of Boc-protected amines.

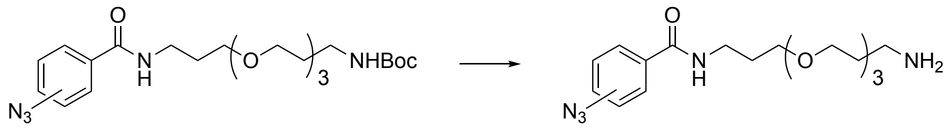

The *N*-Boc-protected starting material (1.0 equiv) was dissolved in CH_2Cl_2 (10 mL per mmol; ~0.1 M) and cooled to 0 °C, and then TFA (1 mL per mmol) was added dropwise. The reaction mixture was protected from light, and then allowed to warm slowly to rt, and then stirred for between 1 to 16 h (monitored by TLC or NMR). The reaction mixture was then concentrated under reduced pressure, co-evaporating with cyclohexane and where necessary purified by reverse-phase flash column chromatography (C18; H_2O with 0.1% TFA to 100 % MeOH with 0.1% TFA) to give the deprotected compounds (the 'free' primary amines) as a colorless to yellow oils (66 to 99%).

General Procedure D: Carboxylic acid functionalisation of photoactivatable PEG$_3$ derivatives.

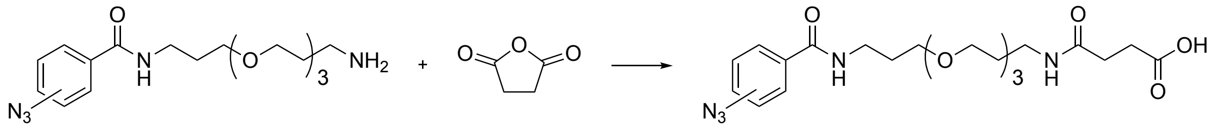

The primary amine compound (1.0 equiv) was taken up in anh. DMF (2 mL per mmol). Succinic anhydride (6.0 equiv) was added and the reaction mixture was stirred at rt for 16 h. The reaction was monitored by TLC or NMR and upon completion, the solvent was removed under reduced pressure and the crude mixture was purified by reverse-phase flash column chromatography (C18; H_2O with 0.1% TFA to 100 % MeOH with 0.1% TFA) to give compound the 'free' carboxylic acid compounds as colorless to brown oils (29 to 91%).

General Procedure E: HATU mediated amide coupling of carboxylic acid functionalized photoactivatable PEG$_3$ derivatives to desferrioxamine B mesylate.

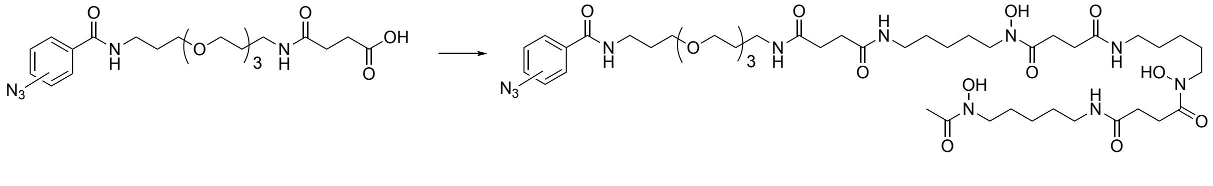

The carboxylic acid functionalized photoactivatable PEG$_3$ derivative starting material (1.0 equiv) was dissolved in anh. DMF (5 mL per 0.05 mmol) to which HATU (1.5 equiv) and

desferrioxamine B (DFO) mesylate (1.2 equiv) were then added sequentially, and the reaction mixture was stirred at rt for 30 min. Then, DIPEA (4 equiv) was added and the reaction was stirred under N_2 at rt for 16 to 72 h (monitored by TLC or NMR). Upon completion, the reaction mixture was concentrated under reduced pressure and the crude residue was purified by reverse-phase flash column chromatography (C18; H_2O with 0.1% TFA to 100 % MeOH with 0.1% TFA) followed by washing with ice-cold acetone (6 × 5 mL; solid product separated by centrifugation between each wash) to give the photoactivatable DFO-PEG$_3$ derivatives as off-white to pale red/brown solids (20 to 73%).

General Procedure F: Carboxylic acid activation to the corresponding N-hydroxysuccinimide ester

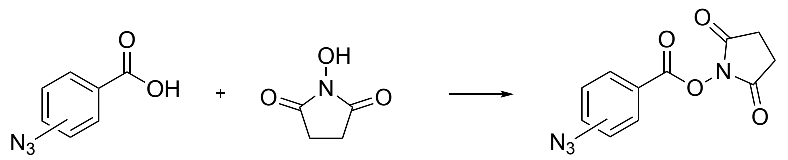

The carboxylic acid functionalized photoactivatable derivative (1.0 equiv) was suspended in CH_2Cl_2 (5 mL per 1 mmol) to which *N*-hydroxysuccinimide (NHS) (1.2 equiv) and *N*-ethyl-*N′*-(3-dimetylaminopropyl)-carbodiimide hydrochloride (EDC; 1.2 equiv) were then added and the reaction mixture was stirred under N_2 at rt for 16 h (monitored by TLC or NMR). Upon completion the reaction mixture was diluted with CH_2Cl_2 and washed with water and brine. The organic layer was then dried over anhydrous $MgSO_4$, filtered, and concentrated under reduced pressure. The crude residue was purified by column chromatography [SiO_2; in a gradient of 2:1 to 1:1 (Hexane:EtOAc)] to give the NHS-activated derivatives as a pale-orange solid (44 to 95%).

General Procedure G: N-Hydroxysuccinimide ester activated coupling to DFO mesylate

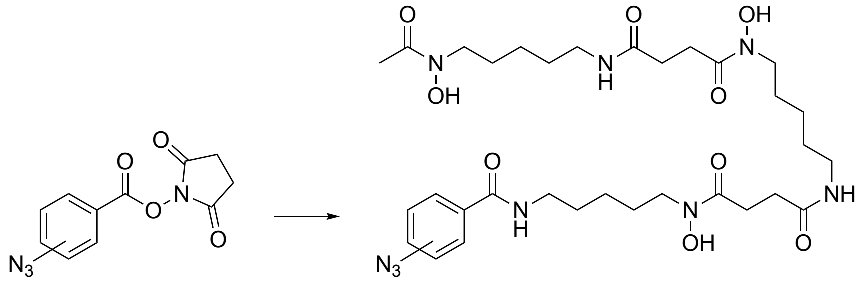

To a suspension of DFO mesylate (1.0 equiv) in anh. DMF (5 mL per 0.05 mmol) was added the NHS-activated ester derivative starting material (1.2 to 2.0 equiv) and Et_3N (4 equiv) and the reaction mixture was stirred under an N_2 atmosphere at rt for 16 h (monitored by TLC or

NMR). Upon completion, the reaction mixture was concentrated under reduced pressure and the crude residue was washed with ice-cold acetone (6 × 5 mL; solid product separated by centrifugation between each wash) to give the photoactivatable DFO derivatives as an off-white solid (21 to 36%).

Synthesis of DFO-PEG$_3$-para-ArN$_3$ (1)

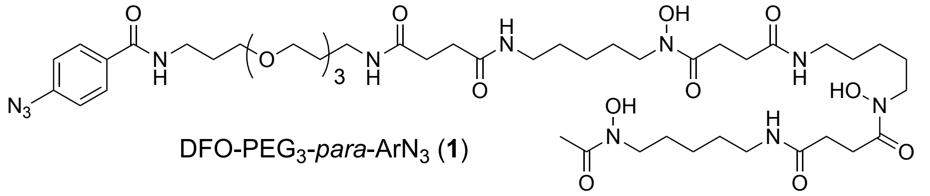

DFO-PEG$_3$-*para*-ArN$_3$ (**1**)

Compound **1** was prepared in accordance with the reported procedure.[5] **^{1}H NMR:** (DMSO-d^6, 400 MHz, 298 K) δ (ppm) 9.66 (br s, 2H, N*H*), 8.44 (br s, 1H, N*H*), 7.88 (d, 2H, ^3J = 8 Hz, CH_{Ar}), 7.76 (br s, 3H, N-O*H*), 7.19 (d, 2H, ^3J = 8 Hz, CH_{Ar}), 3.57-3.30 (m, 20H, CH_2), 3.12-3.00 (m, 6H, CH_2), 1.62-1.57 (m, 2H, CH_2), 1.51-1.48 (m, 4H, CH_2), 1.40-1.37 (m, 4H, CH_2), 1.34-1.04 (m, 6H, CH_2). **^{13}C{^{1}H} NMR:** (DMSO-d^6, 101 MHz, 298 K) δ (ppm) 172.4 (C=O), 171.8 (C=O), 171.7 (C=O), 171.6 (C=O), 165.7 (C=O), 142.6 (C_{qt}), 131.6 (C_{qt}), 129.5 (CH_{Ar}), 119.3 (CH_{Ar}), 70.3 (CH_2), 70.2 (CH_2), 70.0 (CH_2), 70.0 (CH_2), 68.7 (CH_2), 68.5 (CH_2), 47.5 (CH_2), 47.2 (CH_2), 38.9 (CH_2), 38.7 (CH_2), 37.2 (CH_2), 36.3 (CH_2), 31.4 (CH_2), 31.3 (CH_2), 30.4 (CH_2), 29.8 (CH_2), 29.3 (CH_2), 28.0 (CH_2), 26.5 (CH_2), 23.9 (CH_2), 20.8 (CH_3). **HR-ESI-MS:** *m*/*z* calcd. for $[C_{60}H_{75}N_6O_8+H]^{2+}$ 504.78960, found 504.78962. **HPLC:** R_t = 8.80 min (40 to 95% MeOH with 0.1% TFA, 11 min).

Synthesis of DFO-PEG$_3$-meta-ArN$_3$ (2)

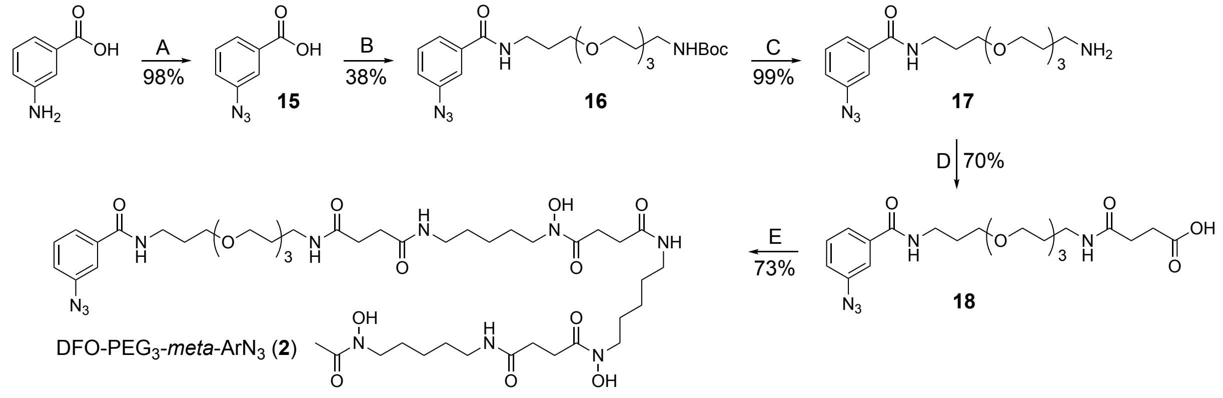

Scheme S1. Synthetic pathway toward DFO-PEG$_3$-*meta*-ArN$_3$ (**2**)

Compound 15

Compound **15** was prepared from 3-aminobenzoic acid (1.00 g, 7.29 mmol) according to **General Procedure A** to give **15** as an off-white solid (1.17 g, 98% yield). **^{1}H NMR:** (DMSO-d^6, 400 MHz, 298 K) δ (ppm) 7.75 (dt, *J* = 7.7, 1.3 Hz, 1H, CH$_{Ar}$), 7.57 (dd, *J* = 3.3, 1.5 Hz, 1H, CH$_{Ar}$), 7.53 (d, *J* = 7.9 Hz, 1H, CH$_{Ar}$), 7.38 (ddd, *J* = 8.1, 2.5, 1.1 Hz, 1H, CH$_{Ar}$). **^{13}C{^{1}H} NMR:** (DMSO-d^6, 101 MHz, 298 K) δ (ppm) 166.5 (C=O), 142.0 (C$_{qt}$), 132.7 (C$_{qt}$), 131.2 (CH$_{Ar}$), 125.9 (CH$_{Ar}$), 123.5 (CH$_{Ar}$), 120.2 (CH$_{Ar}$). **HR-ESI-MS:** *m*/*z* calcd. for [$C_7H_4N_3O_2$-H]$^-$ 162.04600, found 162.03087.

Compound 16

Compound **16** was prepared from compound **15** (300 mg, 1.84 mmol) according to **General Procedure B** to give **16** as a colorless oil (321 mg, 38% yield). **^{1}H NMR:** (CDCl$_3$, 400 MHz, 298 K) δ (ppm) 7.51 (d, *J* = 6.0 Hz, 1H, CH$_{Ar}$), 7.42 (d, *J* = 7.7 Hz, 1H, CH$_{Ar}$), 7.36 (d, *J* = 2.5 Hz, 1H, CH$_{Ar}$), 7.21 (td, *J* = 8.0, 2.5 Hz, 1H, CH$_{Ar}$), 6.94 (dd, *J* = 8.1, 2.8 Hz, 1H, CH$_{Ar}$), 3.56 – 3.25 (m, 14H, CH$_2$), 3.01 (q, *J* = 6.6 Hz, 2H, CH$_2$), 1.80 – 1.66 (m, 2H, CH$_2$), 1.54 (td, *J* = 6.4, 2.0 Hz, 2H, CH$_2$), 1.26 (d, *J* = 2.8 Hz, 9H, C(CH$_3$)$_3$). **^{13}C{^{1}H} NMR:** (CDCl$_3$, 101 MHz, 298 K) δ (ppm) 166.1 (C=O), 155.8 (C=O), 140.1 (C$_{qt}$), 136.3 (C$_{qt}$), 129.5 (CH$_{Ar}$), 124.4 (CH$_{Ar}$), 121.3 (CH$_{Ar}$), 118.1 (CH$_{Ar}$), 79.0 ($\underline{C}$(CH$_3$)$_3$), 70.1 (CH$_2$), 70.0 (CH$_2$), 69.9 (CH$_2$), 69.9 (CH$_2$), 69.9 (CH$_2$), 69.8 (CH$_2$), 69.1 (CH$_2$), 38.4 (CH$_2$), 38.1 (CH$_2$), 29.4 (CH$_2$), 28.6 (CH$_2$), 28.1 (C($\underline{C}$H$_3$)$_3$). **HR-ESI-MS:** *m*/*z* calcd. for [$C_{22}H_{35}N_5O_6$+H]$^+$ 466.26656, found 466.26627.

Compound 17

Compound **17** was prepared from compound **16** (321 mg, 0.69 mmol) according to **General Procedure C** to give **17** as a pale-yellow oil (252 mg, 99% yield). **^{1}H NMR:** ($CDCl_3$, 400 MHz, 298 K) δ (ppm) 7.49 (dt, J = 7.9, 1.4 Hz, 1H, CH_{Ar}), 7.45 – 7.37 (m, 3H, CH_{Ar}), 7.37 – 7.28 (m, 2H, CH_{Ar}), 7.17 (dd, J = 7.5, 2.2 Hz, 1H, CH_{Ar}), 3.75 (t, J = 5.2 Hz, 2H, CH_2), 3.69 – 3.55 (m, 9H, CH_2), 3.51 (q, J = 6.6 Hz, 2H, CH_2), 3.27 (q, J = 5.7 Hz, 2H, CH_2), 1.96 (p, J = 5.2 Hz, 2H, CH_2), 1.86 (p, J = 6.4 Hz, 2H, CH_2). **^{13}C{^{1}H} NMR:** ($CDCl_3$, 101 MHz, 298 K) δ (ppm) 168.9 (C=O), 141.1 (C_{qt}), 135.0 (C_{qt}), 130.3 (CH_{Ar}), 124.0 (CH_{Ar}), 122.7 (CH_{Ar}), 118.0 (CH_{Ar}), 71.1 (CH_2), 70.1 (CH_2), 69.8 (CH_2), 69.7 (CH_2), 69.4 (CH_2), 69.1 (CH_2), 41.4 (CH_2), 38.3 (CH_2), 29.0 (CH_2), 26.0 (CH_2). **HR-ESI-MS:** *m/z* calcd. for $[C_{17}H_{27}N_5O_4+H]^+$ 366.21358, found 366.21334.

Compound 18

Compound **18** was prepared from compound **17** (252 mg, 0.68 mmol) according to **General Procedure D** to give **18** as a colorless oil (225 mg, 70% yield). **^{1}H NMR:** (MeOD-d^4, 400 MHz, 298 K) δ (ppm) 7.60 (dt, J = 7.7, 1.3 Hz, 1H, CH_{Ar}), 7.51 – 7.43 (m, 2H, CH_{Ar}), 7.20 (ddd, J = 8.0, 2.4, 1.0 Hz, 1H, CH_{Ar}), 3.71 – 3.51 (m, 10H, CH_2), 3.51 – 3.43 (m, 4H, CH_2), 3.23 (t, J = 6.8 Hz, 2H, CH_2), 2.58 (t, J = 6.7 Hz, 2H, CH_2), 2.45 (t, J = 7.1 Hz, 2H, CH_2), 1.87 (p, J = 6.5 Hz, 2H, CH_2), 1.72 (p, J = 6.5 Hz, 2H, CH_2). **^{13}C{^{1}H} NMR:** (MeOD-d^4, 101 MHz, 298 K) δ (ppm) 176.0 (C=O), 174.3 (C=O), 168.8 (C=O), 141.9 (C_{qt}), 137.5 (C_{qt}), 132.4 (CH_{Ar}), 125.3 (CH_{Ar}), 122.9 (CH_{Ar}), 118.9 (CH_{Ar}), 71.4 (CH_2), 71.2 (CH_2), 71.1 (CH_2), 70.1 (CH_2), 69.8 (CH_2), 38.7 (CH_2), 37.8 (CH_2), 31.6 (CH_2), 30.3 (CH_2). **HR-ESI-MS:** *m/z* calcd. for $[C_{21}H_{31}N_5O_7+H]^+$ 466.23017, found 466.22991.

DFO-PEG$_3$-*meta*-ArN$_3$ (2)

DFO-PEG$_3$-*meta*-ArN$_3$, compound **2** was prepared from compound **22** (225 mg, 0.483 mmol) according to **General Procedure E** to give **2** as an off-white solid (361 mg, 73% yield). **^{1}H NMR:** (DMSO-d^6, 400 MHz, 298 K) δ (ppm) 8.55 (t, J = 5.7 Hz, 1H, N*H*), 7.78 (q, J = 4.6, 3.5 Hz, 3H, O*H*), 7.65 (d, J = 7.7 Hz, 1H, CH_{Ar}), 7.55 (t, J = 2.0 Hz, 1H, CH_{Ar}), 7.48 (t, J = 7.9 Hz, 1H, CH_{Ar}), 7.25 (dd, J = 8.0, 2.3 Hz, 1H, CH_{Ar}), 3.48 (ddt, J = 23.8, 8.0, 3.9 Hz, 14H, CH_2), 3.37 (t, J = 6.3 Hz, 2H, CH_2), 3.31 (q, J = 6.6 Hz, 2H, CH_2), 3.02 (dq, J = 22.5, 6.3, 5.9 Hz, 7H), 2.65 – 2.55 (m, 3H, CH_2), 2.28 (d, J = 6.6 Hz, 6H, CH_2), 1.96 (s, 3H, CH_3), 1.75 (p, J = 6.6 Hz, 2H, CH_2), 1.59 (p, J = 6.7 Hz, 2H, CH_2), 1.50 (m, 5H, CH_2), 1.38 (m, 5H, CH_2), 1.20 (m, 4H, CH_2).**^{13}C{^{1}H} NMR:** (DMSO-d^6, 101 MHz, 298 K) δ (ppm) 172.0 (C=O), 171.4 172.0

(C=O), 171.3 172.0 (C=O), 171.2 172.0 (C=O), 165.2 172.0 (C=O), 139.7 (C_{qt}), 136.4 (C_{qt}),130.0 (CH_{Ar}), 123.9 (CH_{Ar}), 121.7 (CH_{Ar}), 117.7 (CH_{Ar}), 69.8 (CH_2), 69.8 (CH_2), 69.6 (CH_2), 69.6 (CH_2), 68.3 (CH_2), 68.1 (CH_2), 47.1 (CH_2), 46.8 (CH_2), 38.5 (CH_2), 38.4 (CH_2), 36.8 (CH_2), 35.8 (CH_2), 31.0 (CH_2), 30.9 (CH_2), 29.9 (CH_2), 29.4 (CH_2), 29.3 (CH_2), 28.9 (CH_2), 27.6 (CH_2), 26.1 (CH_2), 23.5 (CH_2), 20.4 (CH_3). **HR-ESI-MS:** *m/z* calcd. for $[C_{46}H_{77}N_{11}O_{14}+H]^+$ 1008.57297, found 1008.57119 **HPLC:** R_t = 8.81 min (40 to 95% MeOH with 0.1% TFA, 11 min).

Synthesis of DFO-PEG$_3$-ortho-ArN$_3$ (3)

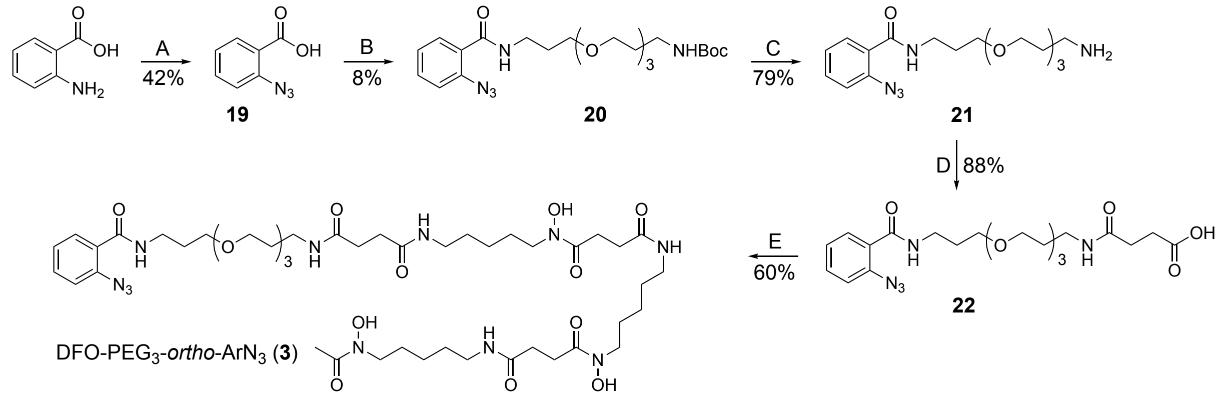

Scheme S2. Synthetic pathway toward DFO-PEG$_3$-*ortho*-ArN$_3$ (**3**)

Compound 19

Compound **19** was prepared from 2-aminobenzoic acid (2.00 g, 14.6 mmol) according to **General Procedure A** to give **19** as an off-white solid (1.00 g, 42% yield).**^{1}H NMR:** (MeOD-d^4, 400 MHz, 298 K) δ (ppm) 7.06 (dd, J = 7.8, 1.7 Hz, 1H, CH_{Ar}), 6.77 (td, J = 7.8, 1.7 Hz, 1H, CH_{Ar}), 6.50 (d, J = 8.0 Hz, 1H, CH_{Ar}), 6.41 (t, J = 7.6 Hz, 1H, CH_{Ar}). **^{13}C{^{1}H} NMR:** (MeOD-d^4, 101 MHz, 298 K) δ (ppm) 167.3 (C=O), 139.8 (C_{qt}), 133.9 (C_{qt}), 133.1 (CH_{Ar}), 131.7 (CH_{Ar}), 131.5 (CH_{Ar}), 124.4 (CH_{Ar}). **HR-ESI-MS:** *m/z* calcd. for $[C_7H_4N_3O_2{-}H]^-$ 162.03090, found 162.03087.

Compound 20

Compound **20** was prepared from compound **19** (300 mg, 1.84 mmol) according to **General Procedure B** to give **20** as a colorless oil (110 mg, 8% yield). **^{1}H NMR:** ($CDCl_3$, 400 MHz, 298 K) δ (ppm) 8.05 (dd, J = 7.9, 1.7 Hz, 1H, CH_{Ar}), 7.62 (s, 1H, CH_{Ar}), 7.47 (td, J = 7.7, 1.7 Hz, 1H, CH_{Ar}), 7.25 – 7.15 (m, 2H, CH_{Ar}), 3.70 – 3.45 (m, 14H, CH_2), 3.19 (t, J = 6.5 Hz, 2H, CH_2), 1.91 (p, J = 6.2 Hz, 2H, CH_2), 1.72 (p, J = 6.3 Hz, 2H, CH_2), 1.42 (s, 9H, $C(CH_3)_3$). **^{13}C{^{1}H} NMR:** ($CDCl_3$, 101 MHz, 298 K) δ (ppm) 164.9 (C=O), 156.2 (C=O), 137.1 (C_{qt}),

132.2 (CH_{Ar}), 132.0 (CH_{Ar}), 125.9 (C_{qt}), 125.2 (CH_{Ar}), 118.6 (CH_{Ar}), 79.1 ($\underline{C}(CH_3)_3$), 70.7 (CH_2), 70.7 (CH_2), 70.5 (CH_2), 70.3 (CH_2), 69.8 (CH_2), 69.7 (CH_2), 38.2 (CH_2), 31.0 (CH_2), 29.8 (CH_2), 29.3 (CH_2), 28.6 ($C(\underline{C}H_3)_3$). **HR-ESI-MS:** *m/z* calcd. for $[C_{22}H_{35}N_5O_6–H]^-$ 466.26601, found 466.26580.

Compound 21

Compound **21** was prepared from compound **20** (110 mg, 0.236 mmol) according to **General Procedure C** to give **21** as a pale-yellow oil (68 mg, 79% yield). **^{1}H NMR:** ($CDCl_3$, 400 MHz, 298 K) δ (ppm) 7.95 (dd, J = 7.8, 1.6 Hz, 2H, CH_{Ar}), 7.90 (br s, 2H, N*H_2*), 7.83 (d, J = 5.9 Hz, 1H, CH_{Ar}), 7.45 (td, J = 7.7, 1.6 Hz, 1H, CH_{Ar}), 7.22 – 7.09 (m, 2H, CH_{Ar}), 3.71 – 3.42 (m, 15H, CH_2), 3.22 – 3.11 (m, 2H, CH_2), 3.06 (qd, J = 7.4, 4.0 Hz, 2H, CH_2), 1.91 (p, J = 5.5 Hz, 2H, CH_2), 1.82 (p, J = 6.3 Hz, 2H, CH_2). **^{13}C{^{1}H} NMR:** ($CDCl_3$, 101 MHz, 298 K) δ (ppm) 165.6 (C=O), 137.2 (C_{qt}), 132.5 (CH_{Ar}), 131.8 (CH_{Ar}), 125.2 (C_{qt}), 124.9 (CH_{Ar}), 118.6 (CH_{Ar}), 70.2 (CH_2), 70.2 (CH_2), 69.8 (CH_2), 69.7 (CH_2), 68.8 (CH_2), 42.3 (CH_{Ar}), 39.9 (CH_2), 37.7 (CH_2), 29.4 (CH_2), 26.3 (CH_2). **HR-ESI-MS:** *m/z* calcd. for $[C_{17}H_{27}N_5O_4+H]^+$ 366.21413, found 366.21304.

Compound 22

Compound **22** was prepared from compound **21** (86 mg, 0.236 mmol) according to **General Procedure D** to give **22** as a colorless oil (97 mg, 88% yield). **^{1}H NMR:** ($CDCl_3$, 400 MHz, 298 K) δ (ppm) 7.97 (dd, J = 7.8, 1.7 Hz, 1H, CH_{Ar}), 7.74 (t, J = 5.6 Hz, 1H, N*H*), 7.45 (td, J = 7.7, 1.6 Hz, 1H, CH_{Ar}), 7.22 – 7.12 (m, 2H, CH_{Ar}), 7.07 (t, J = 5.6 Hz, 1H, N*H*), 3.66 – 3.43 (m, 14H, CH_2), 3.29 (q, J = 6.0 Hz, 2H, CH_2), 2.60 (t, J = 6.7 Hz, 2H, CH_2), 2.46 (t, J = 6.7 Hz, 2H, CH_2), 1.86 (p, J = 6.3 Hz, 2H, CH_2), 1.71 (p, J = 6.0 Hz, 2H, CH_2). **^{13}C{^{1}H} NMR:** ($CDCl_3$, 101 MHz, 298 K) δ (ppm) 175.2 (C=O), 173.0 (C=O), 165.4 (C=O), 137.1 (C_{qt}), 132.4 (CH_{Ar}), 131.8 (CH_{Ar}), 125.3 (C_{qt}), 125.2 (CH_{Ar}), 118.6 (CH_{Ar}), 70.4 (CH_2), 70.4 (CH_2), 70.2 (CH_2), 70.0 (CH_2), 69.8 (CH_2), 69.4 (CH_2), 38.2 (CH_2), 38.1 (CH_2), 30.8 (CH_2), 30.0 (CH_2), 29.2 (CH_2), 28.6 (CH_2). **HR-ESI-MS:** *m/z* calcd. for $[C_{21}H_{30}N_5O_7–H]^-$ 464.21507, found 464.21471.

DFO-PEG$_3$-*ortho*-ArN$_3$ (3)

DFO-PEG$_3$-*ortho*-ArN$_3$, compound **3** was prepared from compound **22** (97 mg, 0.208 mmol) according to **General Procedure E** to give **3** as an off-white solid (125 mg, 60% yield). **^{1}H NMR:** (DMSO-d^6, 400 MHz, 298 K) δ (ppm) 8.28 (t, J = 5.7 Hz, 1H, N*H*), 7.78 (t, J = 5.5 Hz,

4H, O*H*), 7.50 (dq, *J* = 6.6, 2.2, 1.7 Hz, 2H, CH_{Ar}), 7.32 (d, *J* = 8.3 Hz, 1H, CH_{Ar}), 7.23 (t, *J* = 7.5 Hz, 1H, CH_{Ar}), 3.56 – 3.41 (m, 16H, CH_2), 3.37 (t, *J* = 6.4 Hz, 3H, CH_2), 3.28 (q, *J* = 6.5 Hz, 2H, CH_2), 3.01 (ddd, *J* = 19.6, 9.7, 4.7 Hz, 8H, CH_2), 2.57 (t, *J* = 6.8 Hz, 4H, CH_2), 2.28 (d, *J* = 7.4 Hz, 8H, CH_2), 1.96 (s, 3H, CH_3), 1.82 – 1.70 (m, 3H, CH_2), 1.60 (q, *J* = 6.7 Hz, 3H, CH_2), 1.56 – 1.45 (m, 6H, CH_2), 1.43 – 1.32 (m, 6H, CH_2), 1.21 (m, 6H, CH_2). **$^{13}C\{^1H\}$ NMR:** (DMSO-d^6, 101 MHz, 298 K) δ (ppm) 172.0 (C=O), 171.4 (C=O), 171.3 (C=O), 171.2 (C=O), 170.2 (C=O), 165.5 (C=O), 136.5 (C_{qt}), 131.3 (CH_{Ar}), 129.6 (CH_{Ar}), 128.8 (C_{qt}), 124.9 (CH_{Ar}), 119.6 (CH_{Ar}), 69.8 (CH_2), 69.6 (CH_2), 69.6 (CH_2), 68.2 (CH_2), 68.1 (CH_2), 47.1 (CH_2), 46.8 (CH_2), 38.5 (CH_2), 36.6 (CH_2), 35.8 (CH_2), 31.0 (CH_2), 30.9 (CH_2), 30.0 (CH_2), 29.4 (CH_2), 29.2 (CH_2) (CH_2), 28.9 (CH_2), 27.6 (CH_2), 26.1 (CH_2), 23.5 (CH_2), 20.4 (CH_3). **HR-ESI-MS:** *m/z* calcd. for $[C_{46}H_{78}N_{11}O_{14}+H]^+$ 1008.57242, found 1008.57234. **HPLC:** R_t = 9.03 min (40 to 95% MeOH with 0.1% TFA, 11 min).

Synthesis of DFO-PEG$_3$-para-EtArN$_3$ (4)

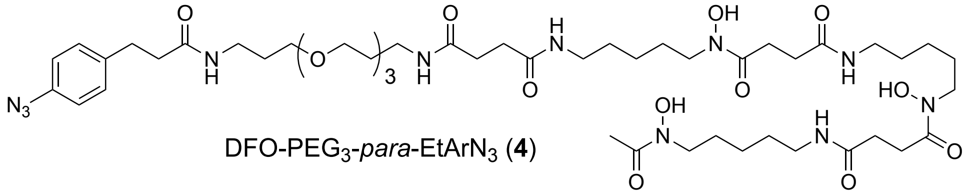

Compound **4** was prepared in accordance with the reported procedure.[5] **^1H NMR:** (DMSO-d^6, 500 MHz, 298 K) δ (ppm) 9.62 (m, 2H, O*H*), 7.77 (q, *J* = 5.0, 4.6 Hz, 4H, N*H*), 7.23 (d, *J* = 8.4 Hz, 2H, CH_{Ar}), 7.02 (d, *J* = 8.4 Hz, 2H, CH_{Ar}), 3.57 – 3.42 (m, 14H, CH_2), 3.37 (t, *J* = 6.4 Hz, 2H, CH_2), 3.32 (t, *J* = 6.4 Hz, 2H, CH_2), 3.10 – 2.94 (m, 10H, CH_2), 2.79 (t, *J* = 7.6 Hz, 2H, CH_2), 2.57 (t, *J* = 7.4 Hz, 4H, CH_2), 2.33 (t, *J* = 7.7 Hz, 3H, CH_2), 2.29 – 2.23 (m, 7H, CH_2), 1.96 (s, 3H, CH_3), 1.58 (dt, *J* = 13.7, 6.8 Hz, 4H, CH_2), 1.49 (t, *J* = 7.2 Hz, 5H, CH_2), 1.41 – 1.34 (m, 5H, CH_2), 1.24 – 1.17 (m, 5H, CH_2). **$^{13}C\{^1H\}$ NMR:** (DMSO-d^6, 126 MHz, 298 K) δ (ppm) 172.0 (C=O), 171.3 (C=O), 171.2 (C=O), 171.1 (C=O), 171.0 (C=O), 170.1 (C=O), 162.3 (C=O), 138.4 (C_{qt}), 136.9 (C_{qt}), 129.8 (CH_{Ar}), 118.9 (CH_{Ar}), 69.7 (CH_2), 69.5 (CH_2), 68.0 (CH_2), 68.0 (CH_2), 47.1 (CH_2), 46.8 (CH_2), 38.4 (CH_2), 38.4 (CH_2), 36.9 (CH_2), 35.8 (CH_2), 35.7 (CH_2), 30.9 (CH_2), 30.9 (CH_2), 30.4 (CH_2), 29.9 (CH_2), 29.4 (CH_2), 29.3 (CH_2), 28.8 (CH_2), 27.6 (CH_2), 26.0 (CH_2), 23.5 (CH_2), 20.3 (CH_3). **HR-ESI-MS:** *m/z* calcd. for $[C_{48}H_{81}N_{11}O_{14}+H]^{2+}$ 518.80550, found 518.80571. **HPLC:** R_t = 9.17 min (40 to 95% MeOH with 0.1% TFA, 11 min).

Synthesis of DFO-PEG₃-meta-OMe-ArN₃ (5)

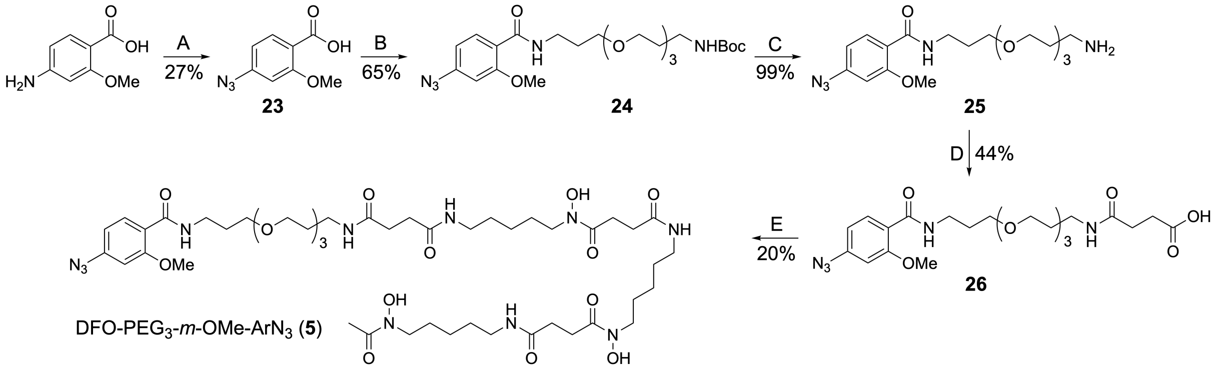

Scheme S3. Synthetic pathway toward DFO-PEG3-*meta*-OMe-ArN3 (**5**)

Compound 23

Compound **23** was prepared from 4-amino-2-methoxybenzoic acid (300 mg, 1.79 mmol) according to **General Procedure A** to give **23** as an orange solid (92 mg, 27% yield). **^{1}H NMR:** ($CDCl_3$, 400 MHz, 298 K) δ (ppm) 8.17 (d, J = 8.5 Hz, 1H, CH_{Ar}), 6.83 (dd, J = 8.5, 2.0 Hz, 1H, CH_{Ar}), 6.61 (d, J = 2.0 Hz, 1H, CH_{Ar}), 4.06 (s, 3H, OCH_3). **^{13}C{^{1}H} NMR:** ($CDCl_3$, 101 MHz, 298 K) δ (ppm) 165.1 (C=O), 159.5 (C_{qt}), 147.2 (C_{qt}), 135.6 (CH_{Ar}), 114.2 (C_{qt}), 112.5 (CH_{Ar}), 102.8 (CH_{Ar}), 57.0 (OCH_3). **HR-ESI-MS:** *m/z* calcd. for $[C_8H_7N_3O_3+Na]^+$ 217.0380, found 217.0379.

Compound 24

Compound **24** was prepared from compound **23** (73 mg, 0.38 mmol) according to **General Procedure B** to give **24** as an orange oil (122 mg, 65% yield). **^{1}H NMR:** ($CDCl_3$, 400 MHz, 298 K) δ (ppm) 8.16 (d, J = 8.5 Hz, 1H, CH_{Ar}), 8.10 (br s, 1H, N*H*), 6.78 (dd, J = 8.5, 2.1 Hz, 1H, CH_{Ar}), 6.55 (d, J = 2.1 Hz, 1H, CH_{Ar}), 3.66 – 3.59 (m, 8H, CH_2), 3.59 – 3.54 (m, 4H, CH_2), 3.52 (t, J = 6.1 Hz, 2H, CH_2), 3.20 (t, J = 6.6 Hz, 2H, CH_2), 1.90 (p, J = 6.4 Hz, 2H, CH_2), 1.75 (p, J = 6.2 Hz, 2H, CH_2) 1.44 (s, 9H, $C(CH_3)_3$). **^{13}C{^{1}H} NMR:** ($CDCl_3$, 101 MHz, 298 K) δ (ppm) 166.3 (C=O), 159.0 (TFA), 159.4 (TFA), 158.8 (C=O), 144.8 (C_{qt}), 133.7 (CH_{Ar}), 117.8 (C_{qt}), 116.5 (TFA), 113.7 (TFA), 111.4 (CH_{Ar}), 102.5 (CH_{Ar}), 70.4 (CH_2), 70.3 (CH_2), 70.2 (CH_2), 70.1 (CH_2), 69.3 (CH_2), 56.7 (OCH_3), 38.5 (CH_2), 37.7 (CH_2), 29.5 (CH_2), 29.2 ($C(\underline{C}H_3)_3$). **HR-ESI-MS:** *m/z* calcd. for $[C_{22}H_{37}N_5 O_7+H]^+$ 518.25852, found 518.25804.

Compound 25

Compound **25** was prepared from compound **24** (110 mg, 0.486 mmol) according to **General Procedure C** to give **25** as an orange oil (130 mg, 99% yield). **^{1}H NMR:** ($CDCl_3$, 400 MHz,

298 K) δ (ppm) 8.05 (dd, J = 11.3, 8.5 Hz, 1H, CH_{Ar}), 7.87 (s, 3H, N*H*), 6.70 (dd, J = 8.5, 2.0 Hz, 1H, CH_{Ar}), 6.51 (d, J = 2.0 Hz, 1H, CH_{Ar}), 3.91 (s, 3H, OCH_3), 3.68 (t, J = 5.3 Hz, 2H, CH_2), 3.64 – 3.51 (m, 8H, CH_2), 3.46 (dt, J = 18.8, 6.2 Hz, 3H, CH_2), 3.20 (q, J = 5.6 Hz, 2H, CH_2), 1.95 (h, J = 8.7, 7.0 Hz, 2H, CH_2), 1.81 (h, J = 6.2 Hz, 2H, CH_2), 1.22 (s, 1H, N*H*). **^{13}C{^{1}H} NMR:** ($CDCl_3$, 101 MHz, 298 K) δ (ppm) 165.4 (C=O), 158.9 (C=O), 145.0 (C_{qt}), 133.6 (CH_{Ar}), 117.5 (C_{qt}), 111.4 (C_{qt}), 102.5 (CH_{Ar}), 70.4 (CH_2), 70.2 (CH_2), 69.7 (CH_2), 69.7 (CH_2), 69.6 (CH_2), 68.5 (CH_2), 56.2 (OCH_3), 40.3 (CH_2), 37.2 (CH_2), 29.7 (CH_2), 26.2 (CH_2). **HR-ESI-MS:** *m/z* calcd. for $[C_{18}H_{29}N_5O_5+H]^+$ 396.22415, found 396.22395.

Compound 26

Compound **26** was prepared from compound **25** (88 mg, 0.222 mmol) according to **General Procedure D** to give **26** as a yellow oil (48 mg, 44% yield). **^{1}H NMR:** ($CDCl_3$, 400 MHz, 298 K) δ (ppm) 8.09 (d, J = 8.0 Hz, 1H, CH_{Ar}), 8.02 (br s, 2H, N*H*), 7, 6.75 (dd, J = 5.6, 2.0 Hz, 1H, CH_{Ar}), 6.53 (d, J = 2.0 Hz, 1H, CH_{Ar}), 3.93 – 3.96 (s, 3H, OCH_3), 3.62 (qd, J = 5.1, 2.3 Hz, 6H, CH_2), 3.54 (m, 8H, CH_2), 3.33 (q, J = 5.8 Hz, CH_2), 2.68 – 2.59 (m, 2H, CH_2), 2.51 (dd, J = 7.9, 5.3 Hz, 2H, CH_2), 1.87 (p, J = 6.3 Hz, 2H, CH_2), 1.75 (t, J = 6.3 Hz, 2H, CH_2). **^{13}C{^{1}H} NMR:** ($CDCl_3$, 101 MHz, 298 K) δ (ppm) 175.3 (C=O), 173.5 (C=O), 165.6 (C=O), 158.9 (C_{qt}), 145.1 (C_{qt}), 133.8 (CH_{Ar}), 117.6 (C_{qt}), 111.6 (CH_{Ar}), 102.6 (CH_{Ar}), 70.4 (CH_2), 70.4 (CH_2), 70.2 (CH_2), 70.0 (CH_2), 69.9 (CH_2), 69.2 (CH_2), 56.3 (OCH_3), 38.4 (CH_2), 37.7 (CH_2), 30.9 (CH_2), 30.1 (CH_2), 29.4 (CH_2), 28.5 (CH_2).

DFO-PEG$_3$-*m*-OMe-ArN$_3$ (5)

DFO-PEG$_3$-*m*-OMe-ArN$_3$, compound **5**, was prepared from compound **26** (48 mg, 0.097 mmol) according to **General Procedure E** to give **5** as a yellow solid (20 mg, 20% yield). **^{1}H NMR:** (DMSO-d^6, 400 MHz, 298 K) δ (ppm) 9.63 (s, 1H, N*H*), 9.60 (s, 2H, N*H*), 8.13 (d, J = 8.3 Hz, 1H, CH_{Ar}), 7.83 – 7.73 (br s, 4H, O*H*), 6.81 (dd, J = 8.3, 2.1 Hz, 1H, CH_{Ar}), 6.78 (d, J = 2.1 Hz, 1H, CH_{Ar}), 3.51 (td, J = 5.3, 4.8, 2.7 Hz, 6H, CH_2), 3.45 (dq, J = 7.1, 4.6, 3.2 Hz, 10H, CH_2), 3.90 (s, 3H, OCH_3), 3.48 (m, 2H, CH_2), 3.09 – 3.03 (m, 2H, CH_2), 2.99 (t, J = 6.2 Hz, 2H, CH_2), 2.57 (t, J = 7.3 Hz, 2H, CH_2), 2.26 (s, 8H, CH_2), 1.96 (s, 3H, CH_3), 1.73 (p, J = 6.6 Hz, 2H, CH_2), 1.59 (p, J = 6.7 Hz, 2H, CH_2), 1.57 – 1.49 (m, 6H, CH_2), 1.39 – 1.35 (m, 6H, CH_2), 1.24 – 1.21 (m, 6H, CH_2). **^{13}C{^{1}H} NMR:** (DMSO-d^6, 126 MHz, 298 K) δ (ppm) 172.0 (C=O), 171.3 (C=O), 171.2 (C=O), 171.1 (C=O), 170.1 (C=O), 164.0 (C=O), 158.2 (C_{qt}), 143.1 (C_{qt}), 132.2 (CH_{Ar}), 119.8 (C_{qt}), 111.0 (CH_{Ar}), 103.1 (C_{Ar}), 69.8 (CH_2), 69.6 (CH_2), 69.5 (CH_2), 68.5 (CH_2), 68.0 (CH_2), 56.2 (OCH_3), 47.1 (CH_2), 38.4 (CH_2), 36.8 (CH_2), 35.8 (CH_2), 30.9

(CH_2), 29.9 (CH_2), 29.4 (CH_2), 29.3 (CH_2), 28.8 (CH_2), 27.6 (CH_2), 26.0 (CH_2), 23.5 (CH_2), 20.3 (CH_3). **HR-ESI-MS:** *m/z* calcd. for $[C_{47}H_{81}N_{11}O_{15}+H]^{2+}$ 519.79513, found 519.79519. **HPLC:** R_t = 8.82 min (40 to 95% MeOH with 0.1% TFA, 11 min).

Synthesis of DFO-PEG₃-meta-Cl-ArN₃ (6)

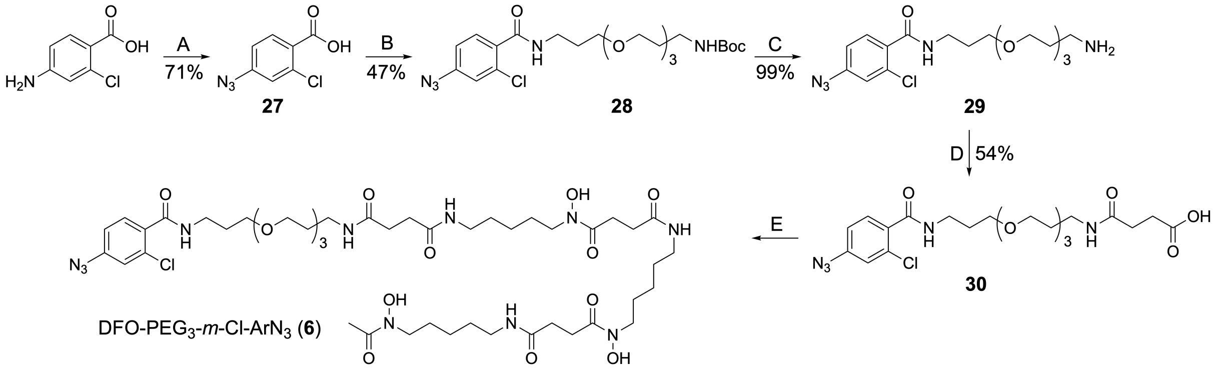

Scheme S4. Synthetic pathway toward DFO-PEG$_3$-*meta*-Cl-ArN$_3$ (**6**)

Compound 27

Compound **27** was prepared from 4-amino-2-chlorobenzoic acid (300 mg, 1.75 mmol) according to **General Procedure A** to give **27** as a yellow solid (246 mg, 71% yield). **^{1}H NMR:** (MeOD-d^4, 400 MHz, 298 K) δ (ppm) 7.92 (d, *J* = 8.4 Hz, 1H, CH_{Ar}), 7.15 (d, *J* = 2.3 Hz, 1H, CH_{Ar}), 7.07 (dd, *J* = 8.4, 2.3 Hz, 1H, CH_{Ar}). **^{13}C{^{1}H} NMR:** (MeOD-d^4, 101 MHz, 298 K) δ (ppm) 167.9 (C=O), 146.0 (C_{qt}), 136.4 (C_{qt}), 134.4 (CH_{Ar}), 127.8 (C_{qt}), 122.4 (CH_{Ar}), 118.4 (CH_{Ar}). **HR-ESI-MS:** *m/z* calcd. for $[C_7H_3N_3\ O_2Cl+H]^+$ 195.99193, found 195.99184.

Compound 28

Compound **28** was prepared from compound **27** (225mg, 1.14 mmol) according to **General Procedure B** to give **28** as an orange oil (266 mg, 47% yield). **^{1}H NMR:** ($CDCl_3$, 400 MHz, 298 K) δ (ppm) 7.63 (d, *J* = 8.3 Hz, 1H, CH_{Ar}), 7.01 (d, *J* = 2.2 Hz, 1H, CH_{Ar}), 6.94 (dd, *J* = 8.3, 2.2 Hz, 1H, CH_{Ar}), 5.28 (s, 1H, N*H*), 3.69 – 3.38 (m, 14H, CH_2), 3.17 (t, *J* = 6.5 Hz, 2H, CH_2), 1.88 (p, *J* = 6.0 Hz, 2H, CH_2), 1.8 – 1.62 (m, 2H, CH_2), 1.40 (s, 9H, $C(C\underline{H}_3)_3$). **^{13}C{^{1}H} NMR:** ($CDCl_3$, 101 MHz, 298 K) δ (ppm) 165.7 (C=O), 156.1 (C=O), 142.8 (C=O), 132.3 (C_{qt}), 132.0 (C_{qt}), 131.7 (CH_{Ar}), 120.5 (CH_{Ar}), 117.6 (CH_{Ar}), 79.1 ($\underline{C}(CH_3)_3$), 70.6 (CH_2), 70.5 (CH_2), 70.4 (CH_2), 70.2 (CH_2), 69.6 (CH_2), 38.7 (CH_2), 29.7 (CH_2), 29.0 (CH_2), 28.5 ($C(\underline{C}H_3)_3$). **HR-ESI-MS:** *m/z* calcd. for $[C_{22}H_{35}N_5O_6Cl+H]^+$ 500.22704, found 500.22692.

Compound 29

Compound **29** was prepared from compound **28** (243 mg, 0.486 mmol) according to **General Procedure C** to give **29** as a yellow oil (194 mg, 99% yield). **^{1}H NMR:** ($CDCl_3$, 400 MHz, 298 K) δ (ppm) 8.09 (br s, 1H, N*H*), 7.83 (br s, 3H, N*H*), 7.60 (d, 1H, CH_{Ar}), 7.09 (m, 1H, CH_{Ar}), 7.02 (d, 1H, CH_{Ar}), 6.96 (dd, 1H, CH_{Ar}), 3.80 – 3.41 (m, 14H, CH_2), 3.20 (br s, 2H, CH_2), 1.97 – 1.93 (m, 2H, CH_2), 1.88 – 1.83 (m, 2H, CH_2). **$^{13}C\{^1H\}$ NMR:** ($CDCl_3$, 101 MHz, 298 K) δ (ppm) 166.8 (C=O), 143.3 (C_{qt}), 132.4 (C_{qt}), 131.6 (CH_{Ar}), 131.2 (C_{qt}), 120.6 (CH_{Ar}), 117.8 (CH_{Ar}), 70.7 (CH_2), 70.4 (CH_2), 69.9 (CH_2), 69.8 (CH_2), 69.6 (CH_2), 68.8 (CH_2), 40.5 (CH_2), 37.8 (CH_2), 29.4 (CH_2), 26.2 (CH_2).

Compound 30

Compound **30** was prepared from compound **29** (194 mg, 0.486 mmol) according to **General Procedure D** to give **30** as a yellow oil (130 mg, 54% yield). **^{1}H NMR:** ($CDCl_3$, 400 MHz, 298 K) δ (ppm) 7.57 (d, *J* = 11.4 Hz, 1H, CH_{Ar}), 7.18 (s, 1H, N*H*), 7.07 (s, 1H, N*H*), 7.02 – 6.90 (m, 4H, N*H*, CH_{Ar}), 3.67 – 3.40 (m, 14H, CH_2), 3.35 – 3.22 (m, 2H, CH_2), 2.57 (t, *J* = 6.6 Hz, 2H, CH_2), 2.44 (t, *J* = 6.8 Hz, 2H, CH_2), 1.92 – 1.82 (m, 2H, CH_2), 1.76 – 1.66 (m, 2H, CH_2). **$^{13}C\{^1H\}$ NMR:** ($CDCl_3$, 101 MHz, 298 K) δ (ppm) 175.3 (C=O), 173.1 (C=O), 166.5 (C=O), 143.1 (C_{qt}), 132.3 (C_{qt}), 131.4 (C_{qt}), 131.4 (CH_{Ar}), 120.5 (CH_{Ar}), 117.6 (CH_{Ar}), 70.3 (CH_2), 70.1 (CH_2), 70.0 (CH_2), 69.6 (CH_2), 38.4 (CH_2), 30.8 (CH_2), 29.9 (CH_2), (CH_2), 28.9 (CH_2), 28.7 (CH_2). **HR-ESI-MS:** *m/z* calcd. for $[C_{21}H_{31}N_5O_7Cl+H]^+$ 500.19065, found 500.19069.

DFO-PEG$_3$-*m*-Cl-ArN$_3$ (6)

DFO-PEG$_3$-*m*-Cl-ArN$_3$, compound **6**, was prepared from compound **30** (84.0 mg, 0.168 mmol) according to **General Procedure E** to give **6** as a white solid (80 mg, 46% yield). **^{1}H NMR:** (DMSO-d^6, 400 MHz, 298 K) δ (ppm) 9.61 (d, *J* = 17.7 Hz, 2H, O*H*), 8.38 (t, *J* = 5.6 Hz, 1H, N*H*), 7.85–7.68 (m, 1H, O*H*), 7.44 (d, *J* = 8.3 Hz, 1H, CH_{Ar}), 7.25 (d, *J* = 2.2 Hz, 1H, CH_{Ar}), 7.14 (dd, *J* = 8.3, 2.2 Hz, 1H, CH_{Ar}), 3.57 – 3.41 (m, 16H, CH_2), 3.38 (t, *J* = 6.4 Hz, 2H, CH_2), 3.26 (q, *J* = 6.6 Hz, 2H, CH_2), 3.13 – 3.04 (m, 2H, CH_2), 2.99 (m, 6H, CH_2), 2.89 (s, 2H, CH_2), 2.57 (t, *J* = 7.4 Hz, 2H, CH_2), 2.28 (d, *J* = 7.6 Hz, 2H, CH_2), 1.96 (s, 3H, CH_3), 1.73 (t, *J* = 6.6 Hz, 2H, CH_2), 1.59 (t, *J* = 6.7 Hz, 2H, CH_2), 1.53 – 1.42 (m, 6H, CH_2), 1.37 (q, 6H, CH_2), 1.22 (m, 6H, CH_2). **$^{13}C\{^1H\}$ NMR:** (DMSO-d^6, 126 MHz, 298 K) δ (ppm) 172.0 (C=O), 171.4 (C=O), 171.3 (C=O), 171.2 (C=O), 170.2 (C=O), 165.7 (C=O), 160.9 (C_{qt}), 141.6 (C_{qt}), 133.6 (C_{qt}), 131.3 (C_{qt}), 130.31 (CH_{Ar}), 120.1 (CH_{Ar}), 117.9 (CH_{Ar}), 69.8 (CH_2), 69.6 (CH_2), 69.6

(CH_2), 68.1 (CH_2), 47.1 (CH_2), 46.8 (CH_2), 44.4 (CH_2), 31.0 (CH_2), 30.9 (CH_2), 30.7 (CH_2), 30.0 (CH_2), 29.4 (CH_2), 29.2 (CH_2), 28.8 (CH_2), 27.6 (CH_2), 26.07 (CH_2), 23.5 (CH_2), 20.4 (CH_3). **HR-ESI-MS:** *m/z* calcd. for $[C_{46}H_{76}N_{11}O_{14}Cl+H]^+$ 1042.53345, found 1042.53372. **HPLC:** R_t = 8.72 min (40 to 95% MeOH with 0.1% TFA, 11 min).

Synthesis of DFO-PEG₃-meta-NO₂-ArN₃ (7)

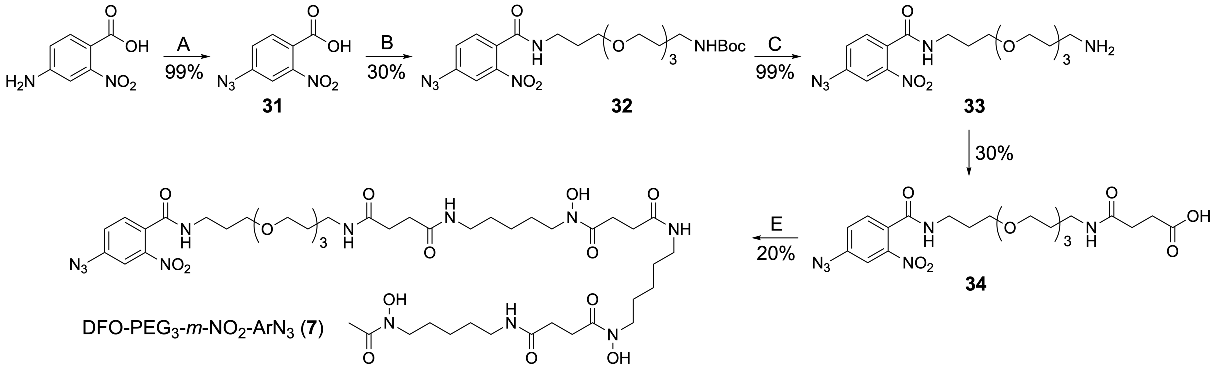

Scheme S5. Synthetic pathway toward DFO-PEG$_3$-*meta*-NO$_2$-ArN$_3$ (**7**)

Compound 31

Compound **31** was prepared from 4-amino-2-nitrobenzoic acid (200 mg, 1.10 mmol) according to **General Procedure A** to give **31** as a yellow solid (230 mg, 99% yield). **^{1}H NMR:** (MeOD-d^4, 400 MHz, 298 K) δ (ppm) 7.92 (d, *J* = 8.4 Hz, 1H, CH_{Ar}), 7.49 (d, *J* = 2.2 Hz, 1H, CH_{Ar}), 7.41 (dd, *J* = 8.4, 2.2 Hz, 1H, CH_{Ar}). **^{13}C{^{1}H} NMR:** (MeOD-d^4, 101 MHz, 298 K) δ (ppm) 166.7 (C=O), 152.2 (C_{qt}), 146.3 (C_{qt}), 133.3 (CH_{Ar}), 123.5 (C_{qt}), 123.1 (CH_{Ar}), 115.4 (CH_{Ar}). **HR-ESI-MS:** *m/z* calcd. for $[C_7H_3N_4O_4–H]^-$ 207.01598, found 207.01587.

Compound 32

Compound **32** was prepared from compound **31** (230 mg, 1.10 mmol) according to **General Procedure B** to give **32** as an orange oil (171 mg, 30% yield). **^{1}H NMR:** ($CDCl_3$, 400 MHz, 298 K) δ (ppm) 7.48 – 7.37 (m, 2H, CH_{Ar}), 7.30 (s, 1H, N*H*), 7.15 (dd, *J* = 8.2, 2.6 Hz, 1H, CH_{Ar}), 5.00 (s, 1H, N*H*), 3.60 – 3.44 (m, 6H, CH_2), 3.42 – 3.27 (m, 8H, CH_2), 3.04 (q, *J* = 6.3 Hz, 2H, CH_2), 1.77 (q, *J* = 6.1 Hz, 2H, CH_2), 1.59 (p, *J* = 6.6 Hz, 2H, CH_2), 1.28 (d, *J* = 3.1 Hz, 9H, $C(C\underline{H}_3)_3$). **^{13}C{^{1}H} NMR:** ($CDCl_3$, 101 MHz, 298 K) δ (ppm) 165.4 (C=O), 155.9 (C=O), 147.8 (C_{qt}), 142.4 (C_{qt}), 130.3 (CH_{Ar}), 129.1 (C_{qt}), 123.0 (CH_{Ar}), 114.5 (CH_{Ar}), 78.7 ($\underline{C}(CH_3)_3$), 70.2 (CH_2), 70.1 (CH_2), 69.8 (CH_2), 69.8 (CH_2), 69.5 (CH_2), 69.2 (CH_2), 38.2 (CH_2), 29.4

(CH_2), 28.4 (CH_2), 28.2 (C(<u>C</u>H_3)$_3$). **HR-ESI-MS:** *m/z* calcd. for $[C_{22}H_{35}N_6O_8+H]^+$ 511.25109, found 511.25193.

Compound 33

Compound **33** was prepared from compound **32** (148 mg, 0.290 mmol) according to **General Procedure C** to give **33** as an orange oil (204 mg, 99% yield).**^{1}H NMR:** ($CDCl_3$, 400 MHz, 298 K) δ (ppm) 12.72 (s, 2H, N*H*), 7.48 – 7.41 (m, 2H, CH_{Ar}), 7.18 (dd, *J* = 8.2, 2.2 Hz, 1H, CH_{Ar}), 3.64 (t, *J* = 5.2 Hz, 2H, CH_2), 3.59 – 3.42 (m, 10H, CH_2), 3.36 (q, *J* = 6.6 Hz, 2H, CH_2), 3.10 (q, *J* = 5.6 Hz, 2H, CH_2), 1.82 (p, *J* = 5.2 Hz, 2H, CH_2), 1.75 (p, *J* = 6.5 Hz, 2H, CH_2), 1.33 (s, 1H, N*H*). **^{13}C{^{1}H} NMR:** ($CDCl_3$, 101 MHz, 298 K) δ (ppm) 167.4 (C=O), 148.0 (C_{qt}), 143.4 (C_{qt}), 130.4 (CH_{Ar}), 127.5 (C_{qt}), 123.4 (CH_{Ar}), 114.1 (CH_{Ar}), 70.8 (CH_2), 67.0 (CH_2), 69.6 (CH_2), 69.5 (CH_2), 69.2 (CH_2), 68.3 (CH_2), 41.0 (CH_2), 37.4 (CH_2), 28.7 (CH_2), 26.9 (CH_2), 26.0 (CH_2). **HR-ESI-MS:** *m/z* calcd. for $[C_{17}H_{27}N_6O_6+H]^+$ 411.19866, found 411.19829.

Compound 34

Compound **34** was prepared from compound **33** (119 mg, 0.290mmol) according to **General Procedure D** to give **34** as a yellow oil (44 mg, 30% yield). **^{1}H NMR:** (MeOD-d^4, 400 MHz, 298 K) δ (ppm) 7.71 (d, *J* = 2.3 Hz, 1H, CH_{Ar}), 7.60 (d, *J* = 8.2 Hz, 1H, CH_{Ar}), 7.46 (dd, *J* = 8.2, 2.3 Hz, 1H, CH_{Ar}), 3.62 (m, 9H, CH_2), 3.48 (dt, *J* = 24.8, 6.5 Hz, 6H, CH_2), 3.37 – 3.29 (m, 1H, CH_2), 3.25 (t, *J* = 6.8 Hz, 2H, CH_2), 2.57 (t, *J* = 7.2 Hz, 2H, CH_2), 2.44 (t, *J* = 6.8 Hz, 2H, CH_2), 1.88 (p, *J* = 6.5 Hz, 2H, CH_2), 1.74 (p, *J* = 6.5 Hz, 2H, CH_2). **^{13}C{^{1}H} NMR:** (MeOD-d^4, 101 MHz, 298 K) δ (ppm) 176.1 (C=O), 174.4 (C=O), 168.5 (C=O), 149.3 (C_{qt}), 144.4 (C_{qt}), 131.7 (CH_{Ar}), 130.0 (C_{qt}), 124.7 (CH_{Ar}), 116.1 (CH_{Ar}), 71.5 (CH_2), 71.5 (CH_2), 71.2 (CH_2), 71.2 (CH_2), 69.8 (CH_2), 69.7 (CH_2), 38.4 (CH_2), 37.8 (CH_2), 31.6 (CH_2), 30.3 (CH_2), 30.3 (CH_2), 30.1 (CH_2). **HR-ESI-MS:** *m/z* calcd. for $[C_{21}H_{31}N_6O_9+H]^+$ 511.21470, found 511.21470.

DFO-PEG$_3$-*m*-NO$_2$-ArN$_3$ (7)

DFO-PEG$_3$-*m*-NO$_2$-ArN$_3$, compound **7**, was prepared from compound **34** (44.0 mg, 0.086 mmol) according to **General Procedure E** to give **7** as a yellow solid (22 mg, 20% yield). **^{1}H NMR:** (DMSO-d^6, 500 MHz, 298 K) δ (ppm) 9.60 (br s, 3H, O*H*), 8.66 (br s, 1H, N*H*), 7.76 (br s, 4H, N*H*), 7.71 (d, *J* = 2.3 Hz, 1H, CH_2), 7.62 (d, *J* = 8.3 Hz, 1H, CH_{Ar}), 7.49 (dd, *J* = 8.3, 2.3 Hz, 1H, CH_{Ar}), 3.53 – 3.43 (m, 14H, CH_2), 3.38 (t, *J* = 6.4 Hz, 2H, CH_2), 3.24 (q, *J* = 6.6

Hz, 2H, CH_2), 3.05 – 2.89 (m, 8H, CH_2), 2.59 – 2.56 (m, 5H, CH_2), 2.26 (br s, 9H, CH_2), 1.96 (s, 3H, CH_3), 1.74 (p, J = 6.6 Hz, 2H, CH_2), 1.61 (p, J = 6.7 Hz, 2H, CH_2), 1.57 – 1.51 (m, 7H, CH_2), 1.49 – 1.41 (m, 7H, CH_2), 1.35 – 1.19 (m, 8H, CH_2). **$^{13}C\{^1H\}$ NMR:** (DMSO-d^6, 126 MHz, 298 K) δ (ppm) 172.0 (C=O), 171.3 (C=O),171.3 (C=O), 171.1 (C=O), 170.1 (C=O), 164.5 (C=O), 148.5 (CH_{Ar}), 142.1 (C_{qt}), 130.5 (C_{qt}), 128.3 (C_{qt}), 123.3 (CH_{Ar}), 114.8 (CH_{Ar}), 69.8 (CH_2), 69.6 (CH_2), 69.5 (CH_{Ar}), 68.1 (CH_2), 67.9 (CH_2), 47.1 (CH_2), 46.8 (CH_2), 38.4 (CH_2), 36.5 (CH_2), 35.8 (CH_2), 30.9 (CH_2), 30.9 (CH_2), 29.9 (CH_2), 29.4 (CH_2), 29.0 (CH_2), 28.8 (CH_2), 27.6 (CH_2), 26.0 (CH_2), 23.5 (CH_2), 20.3 (CH_3). **HR-ESI-MS:** *m/z* calcd. for $[C_{46}H_{76}N_{12}O_{16}+H]^+$ 1075.53945, found 1075.54019. **HPLC:** R_t = 8.18 min (40 to 95% MeOH with 0.1% TFA, 11 min).

Synthesis of DFO-PEG$_3$-4F-ArN$_3$ (8)

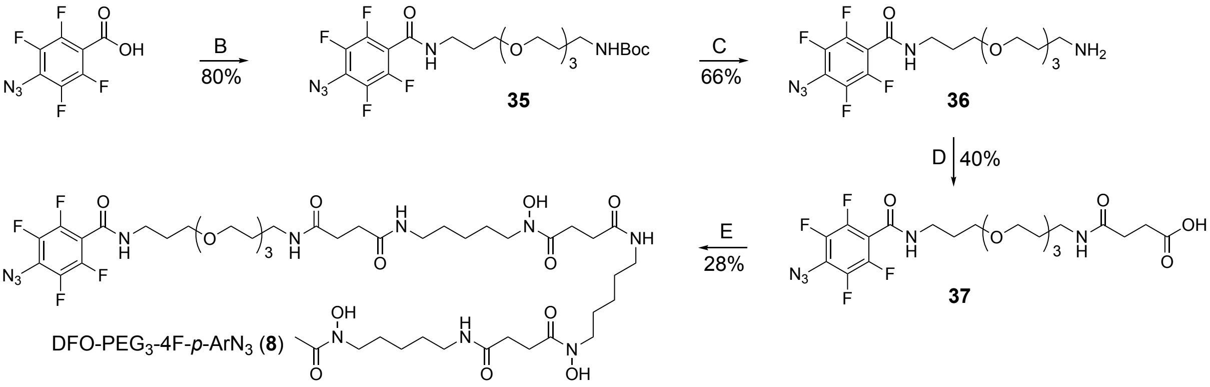

Scheme S6. Synthetic pathway toward DFO-PEG$_3$-4F-*para*-ArN$_3$ (**8**)

Compound 35

Compound **35** was prepared from 4-azido-2,3,5,6-tetrafluorobenzoic acid (301 mg, 1.28 mmol) according to **General Procedure B** to give **35** as a pale-yellow oil (550 mg, 80% yield). **^1H NMR:** ($CDCl_3$, 400 MHz, 298 K) δ (ppm) 7.36 (br s, 1H), 4.96 (br s, 1H, N*H*), 3.60 – 3.38 (m, 14H, CH_2), 3.09 (t, J = 6.9, 5.9 Hz, 2H, CH_2), 1.81 (p, J = 5.9 Hz, 2H, CH_2), 1.65 (p, J = 6.2 Hz, 2H, CH_2), 1.33 (s, 9H, C($\underline{C}H_3)_3$). **$^{13}C\{^1H\}$ NMR:** ($CDCl_3$, 101 MHz, 298 K) δ (ppm) 157.6 (C=O), 156.1 (C=O), 145.8 – 144.5 (CF_{Ar}), 143.3 – 142.2 (CF_{Ar}), 142.09 – 141.11 (CF_{Ar}), 139.2 – 139.1 (CF_{Ar}), 121.3 – 121.1 (C_{qt}), 112.4 – 112.0 (C_{qt}), 79.0 ($\underline{C}(CH_3)_3$), 70.4 (CH_2), 70.3 (CH_2), 70.1 (CH_2), 70.0 (CH_2), 69.5 (CH_2), 69.3 (CH_2), 38.5 (CH_2), 29.6 (CH_2), 28.5 (CH_2), 28.3 (C($\underline{C}H_3)_3$). **$^{19}F\{^1H\}$ NMR:** ($CDCl_3$, 376 MHz, 298 K) δ (ppm) –141.3 (CF_{Ar}), –141.5 (CF_{Ar}), –151.2 (2 × CF_{Ar}). **HR-ESI-MS:** *m/z* calcd. for $[C_{22}H_{32}N_5O_6F_4+H]^+$ 538.22832, found 538.22830.

Compound 36

Compound **36** was prepared from compound **35** (396 mg, 0.733 mmol) according to **General Procedure C** to give **36** as a pale-yellow oil (211 mg, 66% yield). **^{1}H NMR:** ($CDCl_3$, 400 MHz, 298 K) δ (ppm) 9.25 (s, 4H, N*H*), 3.78 (t, *J* = 5.2 Hz, 2H, CH_2), 3.70 – 3.49 (m, 12H, CH_2), 3.26 (q, *J* = 5.5 Hz, 2H, CH_2), 1.97 (p, *J* = 5.2 Hz, 2H, CH_2), 1.86 (p, *J* = 6.4 Hz, 2H, CH_2). **$^{13}C\{^1H\}$ NMR:** ($CDCl_3$, 101 MHz, 298 K) δ (ppm) 158.7 (C=O), 145.3 (CF_{Ar}), 142.8 (CF_{Ar}), 141.8 (CF_{Ar}), 139.3 (CF_{Ar}), 117.5 (C_{qt}), 114.6 (C_{qt}), 77.5 (CH_2), 77.2 (CH_2), 76.8 (CH_2), 70.7 (CH_2), 70.2 (CH_2), 69.7 (CH_2), 69.5 (CH_2), 68.6 (CH_2), 40.5 (CH_2), 37.6 (CH_2), 29.8 (CH_2), 28.9 (CH_2), 26.2 (CH_2). **$^{19}F\{^1H\}$ NMR:** ($CDCl_3$, 376 MHz, 298 K) δ (ppm) –141.4 (CF_{Ar}), –141.6 (CF_{Ar}), –150.6 (CF_{Ar}), –150.7 (CF_{Ar}). **HR-ESI-MS:** *m/z* calcd. for $[C_{17}H_{23}N_5O_4F_4+H]^+$ 438.17589, found 438.17579.

Compound 37

Compound **37** was prepared from compound **36** (120 mg, 0.27 mmol) according to **General Procedure D** to give **37** as a yellow oil (59 mg, 40% yield). **^{1}H NMR:** ($CDCl_3$, 400 MHz, 298 K) δ (ppm) 7.37 (q, *J* = 6.1 Hz, 1H, N*H*), 7.01 (s, 1H, N*H*), 3.59–3.31 (m, 16H, CH_2), 2.59 (m, 2H, CH_2), 2.47 (m, 2H, CH_2), 1.86 (m, 2H, CH_2), 1.73 (m, 2H, CH_2). **$^{13}C\{^1H\}$ NMR:** ($CDCl_3$, 101 MHz, 298 K) δ (ppm) 175.6 (C=O), 173.2 (C=O), 158.2 (C=O), 145.3 (C_{qt}), 142.8 (C_{qt}), 141.7 (CF_{Ar}), 139.2 (CF_{Ar}), 121.6 (CF_{Ar}), 111.8 (CF_{Ar}), 70.3 (CH_2), 70.0 (CH_2), 69.9 (CH_2), 69.9 (CH_2), 69.9 (CH_2), 69.4 (CH_2), 30.8 (CH_2), 29.9 (CH_2), 28.6 (CH_2), 28.6 (CH_2). **$^{19}F\{^1H\}$ NMR:** ($CDCl_3$, 376 MHz, 298 K) δ (ppm) –138.6 (CF_{Ar}), –142.8 (CF_{Ar}), –148.2 (CF_{Ar}), –153.2 (CF_{Ar}). **HR-ESI-MS:** *m/z* calcd. for $[C_{21}H_{28}N_5O_4F_4+Na]^+$ 560.17388, found 560.17386.

DFO-PEG3-4F-ArN3 (8)

DFO-PEG3-4F-ArN3, compound **8**, was prepared from compound **37** (53 mg, 0.098 mmol) according to **General Procedure E** to give **8** as a white solid (27 mg, 28% yield). **^{1}H NMR:** (DMSO-d^6, 400 MHz, 298 K) δ (ppm) 9.61 (d, *J* = 17.6 Hz, 2H, O*H*), 8.86 (t, *J* = 5.8 Hz, 1H, N*H*), 7.76 (m, 3H, N*H*), 3.54–3.41 (m, 16H, CH_2), 3.40 – 3.36 (m, 2H, CH_2), 3.32–3.27 (m, 2H, CH_2), 2.57 (t, *J* = 7.6 Hz, 2H, CH_2), 2.34 – 2.23 (m, 2H, CH_2), 1.96 (s, 3H, CH_3), 1.72 (q, *J* = 6.6 Hz, 2H, CH_2), 1.60 (q, *J* = 6.7 Hz, 2H, CH_2), 1.54–1.44 (m, 1H, CH_2), 1.43 – 1.31 (m, 6H, CH_2), 1.27 – 1.14 (m, 6H, CH_2). **^{13}C{^{1}H} NMR:** (DMSO-d^6, 101 MHz, 298 K) δ (ppm) 172.0 (C=O), 171.3 (C=O), 171.2 (C=O), 171.1 (C=O), 170.1 (C=O), 144.5 – 143.1 (CF_{Ar}), 142.3 – 141.4 (CF_{Ar}), 141.0 (CF_{Ar}), 138.9 (CF_{Ar}), 122.0 (C_{qt}), 112.4(C_{qt}), 69.7 (CH_2), 69.6 (CH_2), 69.5 (CH_2), 68.0 (CH_2), 67.6 (CH_2), 47.1 (CH_2), 46.8 (CH_2), 38.4 (CH_2), 38.4 (CH_2), 36.5 (CH_2), 35. (CH_2)8, 30.9 (CH_2), 30.9 (CH_2), 29.9 (CH_2), 29.4 (CH_2), 28.9 (CH_2), 28.8 (CH_2), 27.6 (CH_2), 26.0 (CH_2), 23.5 (CH_2), 20.3 (CH_3). **^{19}F{^{1}H} NMR:** (DMSO-d^6, 376 MHz, 298 K) δ (ppm) –146.3 (CF_{Ar}), –146.4 (CF_{Ar}), –154.9 (CF_{Ar}), –155.1 (CF_{Ar}). **HR-ESI-MS:** *m/z* calcd. for $[C_{46}H_{73}N_{11}O_{14}F_4+Na]^+$ 1102.51668, found 1102.51719. **HPLC:** R_t = 8.25 min (40 to 95% MeOH with 0.1% TFA, 11 min).

Synthesis of DFO-PEG3-2,4-pyridyl-N3 (9)

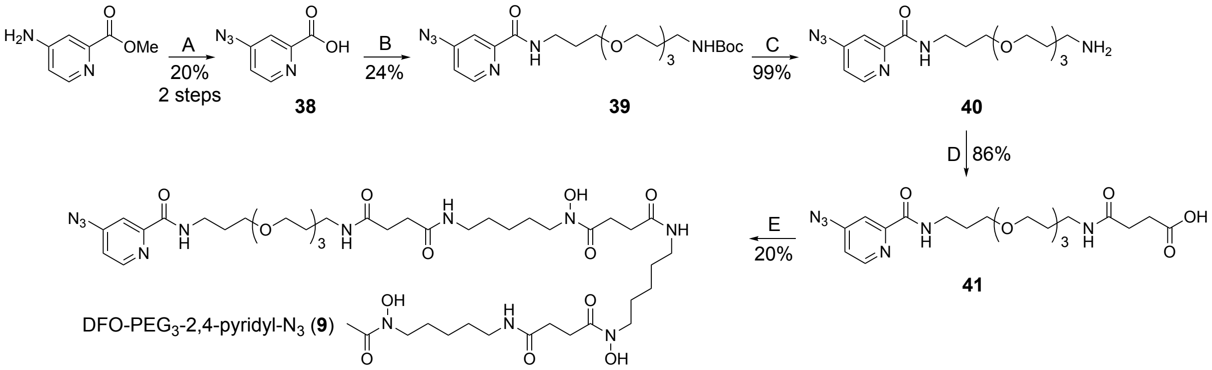

Scheme S7. Synthetic pathway toward DFO-PEG3-2,4-pyridyl-N3 (**9**)

Compound 38

To a stirred solution of methyl 4-aminopicolinate (530 mg, 3.09 mmol) in DMF (6 mL) and H_2O (1 mL), NaN_3 (402 mg, 6.18 mmol) was added and the reaction mixture was stirred at 80 °C for 24 h. After this time, the reaction was cooled to rt and the mixture was concentrated under reduced pressure. The resulting residue was dissolved in EtOAc (20 mL), washed with water (3 × 30 mL) and brine (1 × 30 mL), separated and the organic layer was dried over anh. Na_2SO_4, before being filtered and concentrated under reduced pressure to obtain the methyl 4-

azidopicolinate intermediate as an orange oil. To a stirred solution of the methyl 4-azidopicolinate intermediate (310 mg, 1.74 mmol) in MeOH (6 mL) and H_2O (1 mL), NaOH (104 mg, 2.61 mmol) was added and the reaction mixture was stirred at rt for 24 h. After this time, the reaction mixture was acidified (pH 3) by the addition of 1 M HCl(aq.), extracted with EtOAc (3 × 30 mL), separated and the organic layer was dried over anh. Na_2SO_4, filtered and concentrated under reduced pressure to give compound **38** as a pale pink solid (100 mg, 20% yield over 2 steps). **^{1}H NMR:** (D_2O, 400 MHz, 298 K) δ (ppm) 8.58 (d, J = 6.6 Hz, 1H, CH_{Ar}), 7.95 (d, J = 2.5 Hz, 1H, CH_{Ar}), 7.67 (dd, J = 6.6, 2.5 Hz, 1H, CH_{Ar}). **^{13}C{^{1}H} NMR:** (D_2O, 101 MHz, 298 K) δ (ppm) 162.0 (C=O), 160.4 (C_{qt}), 144.6 (C_{qt}), 142.0 (CH_{Ar}), 118.2 (CH_{Ar}), 117.0 (CH_{Ar}). **HR-ESI-MS:** *m/z* calcd. for $[C_6H_4N_4O_2+H]^+$ 165.04125, found 165.04076.

Compound 39

Compound **39** was prepared from compound **38** (63 mg, 0.384 mmol) according to **General Procedure B** to give **39** as a yellow oil (45 mg, 24% yield). **^{1}H NMR:** ($CDCl_3$, 400 MHz, 298 K) δ (ppm) 8.41 (d, J = 5.4 Hz, 1H, CH_{Ar}), 8.31 (br s, 1H, N*H*), 7.85 (d, J = 2.3 Hz, 1H, CH_{Ar}), 6.98 (dd, J = 5.3, 2.3 Hz, 1H, CH_{Ar}), 3.69 – 3.48 (m, 12H, CH_2), 3.18 (m, 2H, CH_2), 1.88 (p, J = 6.2 Hz, 2H, CH_2), 1.71 (p, J = 6.2 z, 2H, CH_2), 1.39 (s, 9H, $C(CH_3)_3$). **^{13}C{^{1}H} NMR:** ($CDCl_3$, 101 MHz, 298 K) δ (ppm) 163.6 (C=O), 156.1 (C=O), 152.1 (C_{qt}), 150.5 (C_{qt}), 149.5 (CH_{Ar}), 116.2 (CH_{Ar}), 112.5 (CH_{Ar}), 78.9 ($\underline{C}(CH_3)_3$), 70.7 (CH_2), 70.5 (CH_2), 70.3 (CH_2), 69.7 (CH_2), 69.6 (CH_2), 38.6 (CH_2), 37.6 (CH_2), 29.0 (CH_2), 29.3 (CH_2), 28.5 ($C(\underline{C}H_3)_3$). **HR-ESI-MS:** *m/z* calcd. for $[C_{21}H_{35}N_6O_6+H]^+$ 467.26126, found 467.26107.

Compound 40

Compound **40** was prepared from compound **39** (70 mg, 0.150 mmol) according to **General Procedure C** to give **40** as a yellow oil (108 mg, 99% yield). **^{1}H NMR:** ($CDCl_3$, 400 MHz, 298 K) δ (ppm) 8.60 (d, J = 5.9 Hz, 1H, CH_{Ar}), 7.99 (d, J = 2.3 Hz, 1H, CH_{Ar}), 7.50 (s, 3H, N*H*), 7.22 (dd, J = 5.9, 2.3 Hz, 1H, CH_{Ar}), 3.74 (t, J = 5.3 Hz, 2H, CH_2), 3.61 (m, 8H, CH_2), 3.51 (m, 4H, CH_2), 3.27 (m, 2H, CH_2), 1.96 (m, 2H, CH_2), 1.83 (m, 2H, CH_2). **^{13}C{^{1}H} NMR:** ($CDCl_3$, 101 MHz, 298 K) δ (ppm) 162.2 (C=O), 148.9 (C_{qt}), 147.7 (C_{qt}), 117.1 (CH_{Ar}), 114.3 (CH_{Ar}), 70.9 (CH_2), 70.2 (CH_2), 69.7 (CH_2), 69.6 (CH_2), 69.3 (CH_2), 68.3 (CH_2), 41.1 (CH_2), 37.4 (CH_2), 29.8 (CH_2), 29.1 (CH_2), 27.0 (CH_2), 26.1 (CH_2). **HR-ESI-MS:** *m/z* calcd. for $[C_{16}H_{27}N_6O_4+H]^+$ 367.20883, found 367.20919.

Compound 41

Compound **41** was prepared from compound **40** (55 mg, 0.150 mmol) according to **General Procedure D** to give **41** as a pale-yellow oil (60 mg, 86% yield). **^{1}H NMR:** (MeOD-d^4, 400 MHz, 298 K) δ (ppm) 8.56 (d, J = 5.6 Hz, 1H, CH_{Ar}), 7.80 (d, J = 2.3 Hz, 1H, CH_{Ar}), 7.30 (dd, J = 5.6, 2.3 Hz, 1H, CH_{Ar}), 3.71 – 3.35 (m, 15H, CH_2), 3.24 (td, J = 6.8, 2.2 Hz, 2H, CH_2), 2.58 (t, J = 6.9 Hz, 2H), 2.46 (q, J = 6.7 Hz, 2H, CH_2), 1.89 (p, J = 6.3 Hz, 2H, CH_2), 1.73 (p, J = 6.5 Hz, 2H, CH_2). **^{13}C{^{1}H} NMR:** (MeOD-d^4, 101 MHz, 298 K) δ (ppm) 176.1 (C=O), 174.8 (C=O), 164.8 (C=O), 153.6 (C_{qt}), 151.8 (C_{qt}), 150.2 (CH_{Ar}), 117.8 (CH_{Ar}), 114.7(CH_{Ar}), 71.5 (CH_2), 71.5 (CH_2), 71.3 (CH_2), 71.2 (CH_2), 70.3 (CH_2), 69.8 (CH_2), 68.6 (CH_2), 68.6 (CH_2), 52.2 (CH_2), 38.6 (CH_2), 37.8 (CH_2), 31.6 (CH_2), 31.4 (CH_2), 30.3 (CH_2), 30.3 (CH_2), 30.2 (CH_2). **HR-ESI-MS:** *m/z* calcd. for $[C_{20}H_{31}N_6O_7 +H]^+$ 467.22487, found 467.2505.

DFO-PEG$_3$-2,4-pyridyl-N$_3$ (9)

DFO-PEG$_3$-2,4-pyridyl-N$_3$, compound **9**, was prepared from compound **41** (60.0 mg, 0.129 mmol) according to **General Procedure E** to give **9** as an off-white solid (26 mg, 20% yield). **^{1}H NMR:** (DMSO-d^6, 400 MHz, 298 K) δ (ppm) 9.63 (br s, 2H, O*H*), 8.55 (d, J = 5.4 Hz,1H, CH_{Ar}) 7.84 – 7.71 (br s, 4H, O*H*), 7.65 (d, J = 2.3 Hz, 1H, CH_{Ar}), 7.33 (dd, J = 5.4, 2.3 Hz, 1H, CH_{Ar}), 3.83 (m, 11H, CH_2), 3.55 – 3.48 (m, 7H, CH_2), 3.48 – 3.40 (m, 9H, CH_2), 3.36 (q, J = 7.4, 6.9 Hz, 5H, CH_2), 3.11 – 2.95 (m, 7H, CH_2), 2.57 (t, J = 7.4 Hz, 3H, CH_2), 2.27 (s, 8H, CH_2), 1.96 (s, 3H, CH_3), 1.76 (p, J = 6.6 Hz, 3H, CH_2), 1.59 (p, J = 6.6 Hz, 3H, CH_2), 1.49 (m, 5H, CH_2), 1.38 (m, J = 8.5, 5.7, 4.0 Hz, 6H, CH_2), 1.20 (m, J = 9.7 Hz, 6H, CH_2). **^{13}C{^{1}H} NMR:** (DMSO-d^6, 101 MHz, 298 K) δ (ppm) 171.3 (C=O), 171.3 (C=O), 171.2 (C=O), 163.0 (C=O), 151.8 (C_{qt}), 149.9 (C_{qt}), 116.6 (CH_{Ar}), 112.1 (CH_{Ar}), 69.8 (CH_2), 69.78 (CH_2), 69.6 (CH_2), 69.5 (CH_2), 68.6 (CH_2), 68.1 (CH_2), 47.1 (CH_2), 46.8 (CH_2), 38.4 (CH_2), 36.8 (CH_2), 35.8 (CH_2), 30.9 (CH_2), 30.9 (CH_2), 29.9 (CH_2), 29.4 (CH_2), 29.2 (CH_2), 28.8 (CH_2), 27.6 (CH_2), 26.1 (CH_2), 23.5 (CH_2), 20.4 (CH_3). **HR-ESI-MS:** *m/z* calcd. for $[C_{45}H_{76}N_{12}O_{14}+H]^+$ 1009.56767, found 1009.56686. **HPLC:** R_t = 8.27 min (40 to 95% MeOH with 0.1% TFA, 11 min).

Synthesis of DFO-PEG$_3$-2,3-naphthyl-N$_3$ (10)

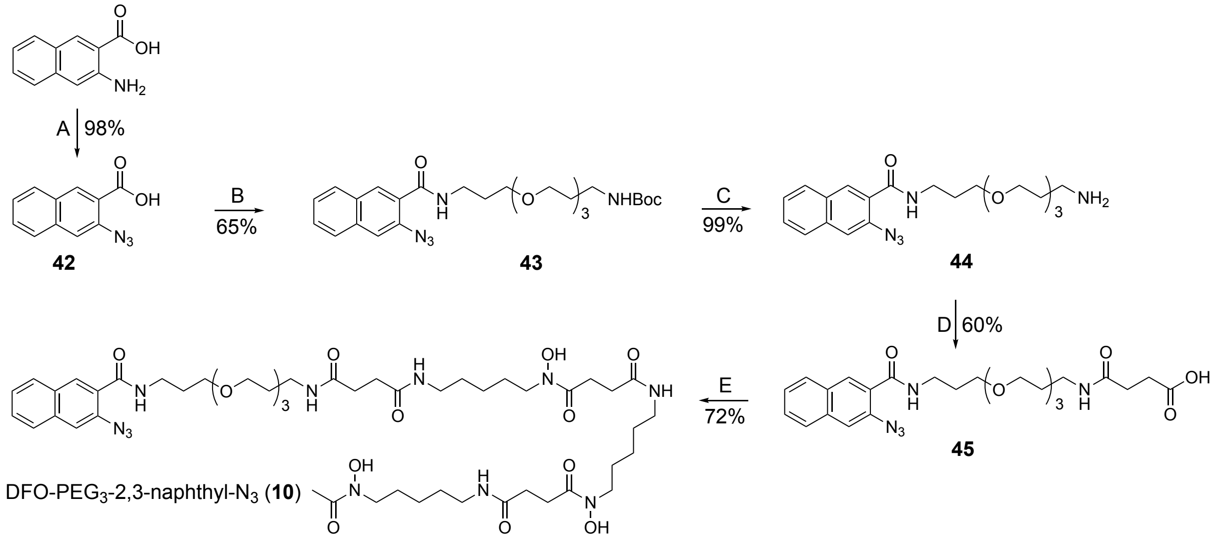

Scheme S8. Synthetic pathway toward DFO-PEG$_3$-2,3-naphthyl-N$_3$ (**10**)

Compound 42

Compound **42** was prepared from 3-amino-2-naphthoic acid (200 mg, 1.07 mmol) according to **General Procedure A** to give **42** as a pink solid (223 mg, 98% yield). Note: 2 mL of DMSO was added to solubilize the starting material. **^{1}H NMR:** (400 MHz, MeOD) δ (ppm) 7.62 (s, 1H, O*H*), 7.11 (d, *J* = 8.2 Hz, 1H, CH$_{Ar}$), 7.06 (d, *J* = 8.3 Hz, 1H, CH$_{Ar}$), 6.91 (s, 1H, CH$_{Ar}$), 6.79 (ddd, *J* = 8.3, 6.8, 1.3 Hz, 1H, CH$_{Ar}$), 6.69 (ddd, *J* = 8.2, 6.8, 1.2 Hz, 1H, CH$_{Ar}$). **^{13}C{^{1}H} NMR:** (101 MHz, MeOD) δ (ppm) 166.4 (C=O), 127.8 (C$_{qt}$), 125.2 (CH$_{Ar}$), 122.6 (C$_{qt}$), 120.6 (CH$_{Ar}$), 120.4 (CH$_{Ar}$), 118.6 (CH$_{Ar}$), 118.0 (CH$_{Ar}$), 109.1 (CH$_{Ar}$). **HR-APCI-MS:** *m/z* calcd. for $[C_{11}H_6N_3O_2–H]^-$ 212.04655, found 212.04652.

Compound 43

Compound **43** was prepared from compound **42** (213 mg, 0.99 mmol) according to **General Procedure B** to give **43** as a pale-red oil (334 mg, 65% yield). **^{1}H NMR:** (CDCl$_3$, 400 MHz, 298 K) δ (ppm) 8.52 – 8.32 (m, 1H, CH$_{Ar}$), 7.77 (m, 1H, CH$_{Ar}$), 7.68 (m, 2H, CH$_{Ar}$), 7.49 – 7.30 (m, 3H, CH$_{Ar}$), 3.64 – 3.29 (m, 15H, CH$_2$), 3.18 – 3.03 (m, 2H, CH$_2$), 1.86 (q, *J* = 6.5 Hz, 2H, CH$_2$), 1.61 (q, *J* = 6.6 Hz, 2H, CH$_2$), 1.40 – 1.26 (m, 9H, C(CH$_3$)$_3$). **^{13}C{^{1}H} NMR:** (CDCl$_3$, 101 MHz, 298 K) δ (ppm) 165.0 (C=O), 156.0 (C=O), 134.5 (C$_{qt}$), 134.3 (C$_{qt}$), 132.7 (CH$_{Ar}$), 130.3 (C$_{qt}$), 128.9 (CH$_{Ar}$), 128.5 (C$_{qt}$), 126.2 (CH$_{Ar}$),116.0 (CH$_{Ar}$), 78.9 (<u>C</u>(CH$_3$)$_3$), 70.5 (CH$_2$), 70.5 (CH$_2$), 70.5 (CH$_2$), 70.3 (CH$_2$), 70.1 (CH$_2$), 69.7 (CH$_2$), 69.7 (CH$_2$), 69.4 (CH$_2$), 38.6 (CH$_2$), 38.2 (CH$_2$), 38.2 (CH$_2$), 29.6 (CH$_2$), 29.2 (CH$_2$), 28.4 (C(<u>C</u>H$_3$)$_3$). **HR-APCI-MS:** *m/z* calcd. for $[C_{26}H_{37}N_5O_6+H]^+$ 516.28166, found 516.28208.

Compound 44

Compound **44** was prepared from compound **43** (334 mg, 0.65 mmol) according to **General Procedure C** to give **44** as a yellow oil (269 mg, 99% yield). **^{1}H NMR:** ($CDCl_3$, 400 MHz, 298 K) δ (ppm) 8.41 (s, 1H, CH_{Ar}), 8.00 (t, *J* = 5.9 Hz, 1H, CH_{Ar}), 7.78 (d, *J* = 8.1 Hz, 1H, CH_{Ar}), 7.66 (d, *J* = 8.2 Hz, 1H, CH_{Ar}), 7.58 – 7.45 (m, 4H, CH_{Ar}), 7.38 (td, *J* = 7.4, 6.7, 1.1 Hz, 1H, CH_{Ar}), 3.72 – 3.40 (m, 16H, CH_2), 3.19 (q, *J* = 5.4 Hz, 2H, CH_2), 1.89 (p, *J* = 5.3 Hz, 2H, CH_2), 1.80 (dq, *J* = 11.9, 6.3 Hz, 2H, CH_2). **^{13}C{^{1}H} NMR:** ($CDCl_3$, 101 MHz, 298 K) δ (ppm) 166.3 (C=O), 134.8 (C_{qt}), 134.2 (C_{qt}), 133.1 (CH_{Ar}), 130.2 (C_{qt}), 129.0 (CH_{Ar}), 126.5 (CH_{Ar}), 126.2 (CH_{Ar}), 123.4 (CH_{Ar}), 116.2 (CH_{Ar}), 70.8 (CH_2), 70.1 (CH_2), 69.6 (CH_2), 69.6 (CH_2), 69.3 (CH_2), 68.5 (CH_2), 41.0 (CH_2), 38.7 (CH_2), 37.8 (CH_2), 29.3 (CH_2), 26.0 (CH_2). **HR-ESI-MS:** *m/z* calcd. for □$C_{21}H_{29}N_5O_4$+H□$^{+}$ 416.22923, found 416.22901.

Compound 45

Compound **45** was prepared from compound **44** (269 mg, 0.65 mmol) according to **General Procedure D** to give **45** as a yellow oil (200 mg, 60% yield). **^{1}H NMR:** (MeOD-d^{4}, 400 MHz, 298 K) δ (ppm) 8.12 (s, 1H, CH_{Ar}), 7.86 (d, *J* = 8.2 Hz, 1H, CH_{Ar}), 7.81 (d, *J* = 8.2 Hz, 1H, CH_{Ar}), 7.62 (s, 1H, CH_{Ar}), 7.56 (ddd, *J* = 8.2, 6.8, 1.3 Hz, 1H, CH_{Ar}), 7.48 (ddd, *J* = 8.1, 6.8, 1.2 Hz, 1H, CH_{Ar}), 3.75 – 3.39 (m, 14H), 3.23 (t, *J* = 6.8 Hz, 2H), 2.59 (t, *J* = 6.9 Hz, 2H), 2.45 (t, *J* = 7.0 Hz, 2H), 1.93 (p, *J* = 6.5 Hz, 2H), 1.71 (p, *J* = 6.5 Hz, 2H). **^{13}C{^{1}H} NMR:** (MeOD-d^{4}, 101 MHz, 298 K) δ 174.7 (C=O), 173.0 (C=O), 167.2 (C=O), 134.6 (C_{qt}), 134.5 (C_{qt}), 130.2 (CH_{Ar}), 128.2 (CH_{Ar}), 128.1 (CH_{Ar}), 126.8 (C_{qt}), 126.3 (CH_{Ar}), 126.0 (CH_{Ar}), 116.2 (CH_{Ar}), 70.2 (CH_2), 70.1 (CH_2), 69.9 (CH_2), 69.8 (CH_2), 68.8 (CH_2), 68.5 (CH_2), 37.4 (CH_2), 36.5 (CH_2), 30.3 (CH_2), 29.0 (CH_2), 29.0 (CH_2). **HR-ESI-MS:** *m/z* calcd. for $[C_{25}H_{32}N_5O_7–H]^{–}$ 516.24527, found 514.23069.

DFO-PEG$_3$-2,3-naphthyl-N$_3$ (10)

DFO-PEG$_3$-2,3-naphthyl-N$_3$, compound **10**, was prepared from compound **45** (200 mg, 0.39 mmol) according to **General Procedure E** to give **10** as a pale-yellow solid (294 mg, 72% yield). **^{1}H NMR:** (DMSO-d^{6}, 400 MHz, 298 K) δ (ppm) 9.64 (br s, 2H, O*H*), 8.43 (t, *J* = 5.6 Hz, 1H, N*H*), 8.10 (s, 1H, CH_{Ar}), 7.98 (t, *J* = 8.8 Hz, 2H, CH_{Ar}), 7.88 (s, 1H, CH_{Ar}), 7.78 (q, *J* = 5.2 Hz, N*H*), 7.67 – 7.57 (m, 1H, CH_{Ar}), 7.57 – 7.45 (m, 1H, CH_{Ar}), 3.62 – 3.42 (m, 15H, CH_2), 3.36 (m, 4H, CH_2), 3.04 (m, 8H, CH_2), 2.71 – 2.55 (m, 3H, CH_2), 2.29 (d, *J* = 7.5 Hz, 7H, CH_2), 1.98 (s, 3H, CH_3), 1.79 (p, *J* = 6.6 Hz, 2H, CH_2), 1.61 (m, 4H, CH_2), 1.51 (m, 5H,

CH_2), 1.39 (m, 5H, CH_2), 1.31 – 1.10 (m, 5H, CH_2). **$^{13}C\{^1H\}$ NMR:** (DMSO-d^6, 101 MHz, 298 K) δ (ppm) 172.5 (C=O), 171.8 (C=O), 171.7 (C=O), 171.6 (C=O), 166.0 (C=O), 134.9 (C_{qt}), 134.2 (C_{qt}), 130.3 (CH_{Ar}), 129.8 (CH_{Ar}), 129.0 (C_{qt}), 128.7 (CH_{Ar}), 128.4 (CH_{Ar}), 127.1 (C_{qt}), 126.6 (CH_{Ar}), 117.2 (CH_{Ar}), 70.3 (CH_2), 70.2 (CH_2), 70.1 (CH_2), 70.0 (CH_2), 68.7 (CH_2), 68.5 (CH_2), 47.5 (CH_2), 47.3 (CH_2), 37.1 (CH_2), 36.3 (CH_2), 31.4 (CH_2), 31.4 (CH_2), 30.4 (CH_2), 29.8 (CH_2), 29.7 (CH_2), 29.3 (CH_2), 28.0 (CH_2), 26.5 (CH_2), 24.0 (CH_2), 20.8 (CH_3). **HR-ESI-MS:** *m/z* 1058.58890 calcd for $[C_{50}H_{79}N_{11}O_{14}+H]^+$ found 1058.58829. **HPLC:** R_t = 9.06 min (40 to 95% MeOH with 0.1% TFA, 11 min).

Synthesis of DFO-PEG$_3$-2,6-naphthyl-N$_3$ (11)

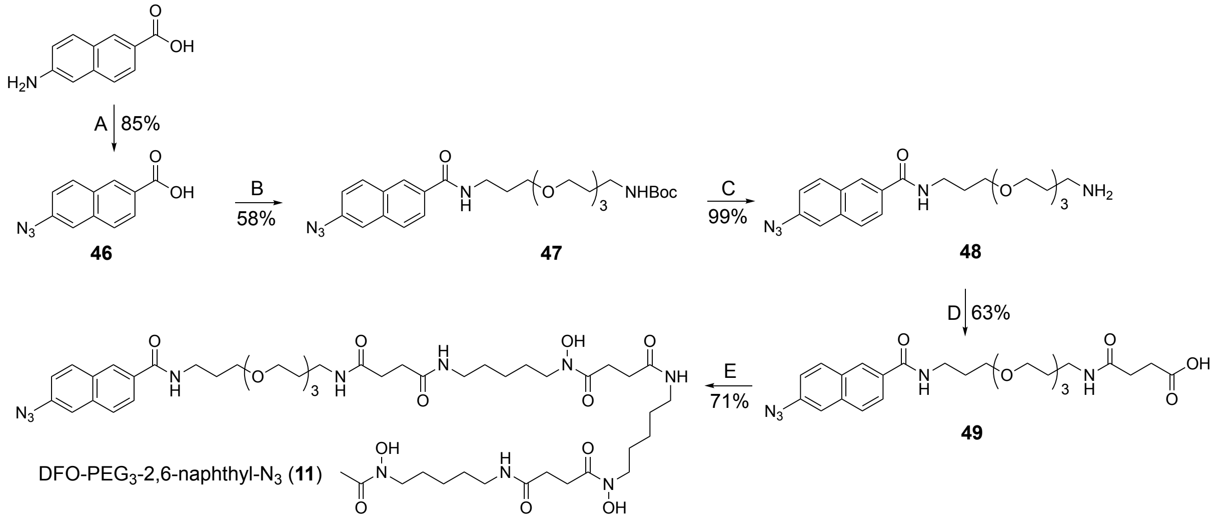

Scheme S9. Synthetic pathway toward DFO-PEG$_3$-2,6-naphthyl-N$_3$ (**11**)

Compound 46

Compound **46** was prepared from 6-amino-2-naphthoic acid (200 mg, 1.07 mmol) according to **General Procedure A** to give **46** as a red solid (192 mg, 85% yield). Note: 2 mL of DMSO was added to solubilize the starting material. **^1H NMR:** (DMSO-d^6, 400 MHz, 298 K) δ (ppm) 8.59 (d, *J* = 1.3 Hz, 1H, CH_{Ar}), 8.16 (d, *J* = 8.8 Hz, 1H, CH_{Ar}), 7.98 (d, *J* = 1.8 Hz, 2H, CH_{Ar}), 7.74 (d, *J* = 2.3 Hz, 1H, CH_{Ar}), 7.34 (dd, *J* = 8.8, 2.3 Hz, 1H, CH_{Ar}). **$^{13}C\{^1H\}$ NMR:** (DMSO-d^6, 400 MHz, 298 K) δ (ppm) 167.3 (C=O), 139.5 (C_{qt}), 135.7 (C_{qt}), 131.5 (CH_{Ar}), 130.5 (CH_{Ar}), 129.7 (C_{qt}), 127.5 (C_{qt}), 127.3 (CH_{Ar}), 126.3 (CH_{Ar}), 119.8 (CH_{Ar}), 115.7 (CH_{Ar}). **HR-APCI-MS:** *m/z* calcd. for $[C_{11}H_6N_3O_2–H]^-$ 212.04655, found 212.05654.

Compound 47

Compound **47** was prepared from compound **46** (179 mg, 0.84 mmol) according to **General Procedure B** to give **47** as a pale-red oil (251 mg, 58% yield). **^{1}H NMR:** ($CDCl_3$, 400 MHz, 298 K) δ (ppm) 8.22 (d, *J* = 1.8 Hz, 1H, CH_{Ar}), 7.80 (td, *J* = 5.8, 2.9 Hz, 2H, CH_{Ar}), 7.67 (d, *J* = 8.6 Hz, 1H, CH_{Ar}), 7.42 (t, *J* = 5.4 Hz, 1H, CH_{Ar}), 7.35 (d, *J* = 2.2 Hz, 1H, CH_{Ar}), 7.08 (dd, *J* = 8.8, 2.3 Hz, 1H, CH_{Ar}), 3.68 – 3.42 (m, 10H, CH_2), 3.42 – 3.20 (m, 4H, CH_2), 3.07 (t, *J* = 6.5 Hz, 2H, CH_2), 1.92 – 1.78 (m, 2H, CH_2), 1.59 (q, *J* = 6.3 Hz, 2H, CH_2), 1.33 (s, 9H, $C(CH_3)_3$). **$^{13}C\{^1H\}$ NMR:** ($CDCl_3$, 400 MHz, 298 K) δ (ppm) 167.1 (C=O), 156.0 (C=O), 139.2 (C_{qt}), 135.2 (C_{qt}), 131.6 (CH_{Ar}), 131.0 (CH_{Ar}), 130.2 (C_{qt}), 127.5 (C_{qt}), 127.1 (CH_{Ar}), 125.0 (CH_{Ar}), 119.5 (CH_{Ar}), 115.5 (CH_{Ar}), 79.0 ($\underline{C}(CH_3)_3$), 70.6 (CH_2), 70.4 (CH_2), 70.4 (CH_2), 70.2 (CH_2), 70.1 (CH_2), 69.4 (CH_2) 39.0 (CH_2), 29.6 (CH_2), 28.9 (CH_2), 28.4 ($C(\underline{C}H_3)_3$). **HR-APCI-MS:** *m/z* calcd. for $[C_{26}H_{37}N_5O_6+H]^+$ 516.28166, found 516.28228.

Compound 48

Compound **48** was prepared from compound **47** (251 mg, 0.49 mmol) according to **General Procedure C** to give **48** as a pale-red oil (202 mg, 99% yield). **^{1}H NMR:** ($CDCl_3$, 400 MHz, 298 K) δ (ppm) δ 8.17 (s, 1H, CH_{Ar}), 7.74 (dd, *J* = 8.7, 4.6 Hz, 3H, CH_{Ar}), 7.67 (d, *J* = 7.5 Hz, 3H, CH_{Ar}), 7.61 (d, *J* = 8.6 Hz, 1H, CH_{Ar}), 7.27 (d, *J* = 2.2 Hz, 1H, CH_{Ar}), 7.04 (dd, *J* = 8.7, 2.2 Hz, 1H, CH_{Ar}), 3.65 – 3.33 (m, 16H, CH_2), 3.13 (q, *J* = 5.5 Hz, 2H, CH_2), 1.85 (p, *J* = 5.3 Hz, 2H. CH_2), 1.76 (p, *J* = 6.3 Hz, 2H, CH_2). **$^{13}C\{^1H\}$ NMR:** ($CDCl_3$, 101 MHz, 298 K) δ (ppm) 168.4 (C=O), 139.5 (C_{qt}), 135.4 (C_{qt}), 131.1 (CH_{Ar}), 130.4 (CH_{Ar}), 130.1 (C_{qt}), 127.7 (C_{qt}), 127.3 (CH_{Ar}), 124.7 (CH_{Ar}), 119.6 (CH_{Ar}), 115.4 (CH_{Ar}), 70.6 (CH_2), 70.1 (CH_2), 69.6 (CH_2), 69.5 (CH_2), 69.3 (CH_2), 68.9 (CH_2), 40.7 (CH_2), 37.9 (CH_2), 29.3 (CH_2), 26.1 (CH_2). **HR-ESI-MS:** *m/z* calcd. for □$C_{21}H_{29}N_5O_4$+H□$^+$ 416.22923, found 416.22885.

Compound 49

Compound **49** was prepared from compound **48** (202 mg, 0.49 mmol) according to **General Procedure D** to give **49** as a pale-red oil (157 mg, 63% yield). **^{1}H NMR:** (MeOD-d^4, 400 MHz, 298 K) δ (ppm) 8.27 (d, *J* = 1.8 Hz, 1H, CH_{Ar}), 7.94 – 7.81 (m, 2H, CH_{Ar}), 7.77 (d, *J* = 8.7 Hz, 1H, CH_{Ar}), 7.44 (d, *J* = 2.3 Hz, 1H, CH_{Ar}), 7.15 (dd, *J* = 8.7, 2.3 Hz, 1H, CH_{Ar}), 3.59 (m, 8H, CH_2), 3.55 – 3.46 (m, 4H), 3.42 (t, *J* = 6.2 Hz, 2H, CH_2), 3.20 (t, *J* = 6.9 Hz, 2H, CH_2), 2.57 (t, *J* = 7.0 Hz, 2H, CH_2), 2.44 (t, *J* = 7.0 Hz, 2H, CH_2), 1.90 (p, *J* = 6.5 Hz, 2H, CH_2), 1.68 (p, *J* = 6.5 Hz, 2H, CH_2). **$^{13}C\{^1H\}$ NMR:** (MeOD-d^4, 101 MHz, 298 K) δ (ppm) 174.7 (C=O), 173.0 (C=O), 168.3 (C=O), 139.4 (C_{qt}), 135.4 (C_{qt}), 131.1 (CH_{Ar}), 130.8 (CH_{Ar}), 130.1 (C_{qt}), 127.3

(C_{qt}), 127.0 (CH_{Ar}), 124.7 (CH_{Ar}), 119.3 (CH_{Ar}), 115.2 (CH_{Ar}), 70.1 (CH_2), 70.1 (CH_2), 69.9 (CH_2), 69.8 (CH_2), 68.9 (CH_2), 68.4 (CH_2), 37.5 (CH_2), 36.5 (CH_2), 30.3 (CH_2), 29.1 (CH_2), 29.0 (CH_2), 29.0 (CH_2). **HR-ESI-MS:** *m/z* calcd. for $[C_{25}H_{33}N_5O_7+H]^+$ 516.24527, found 516.24557.

DFO-PEG$_3$-2,6-naphthyl-N$_3$ (11)

DFO-PEG$_3$-2,6-naphthyl-N$_3$, compound **11**, was prepared from compound **49** (157 mg, 0.31 mmol) according to **General Procedure E** to give **11** as a pale-red solid (224 mg, 71% yield). **^{1}H NMR:** (DMSO-d^6, 400 MHz, 298 K) δ (ppm) 9.63 (br s, 2H, O*H*), 8.60 (t, *J* = 5.6 Hz, 1H, N*H*), 8.43 (s, 1H, CH_{Ar}), 8.07 (d, *J* = 8.8 Hz, 1H, CH_{Ar}), 7.95 (d, *J* = 2.1 Hz, 2H, CH_{Ar}), 7.79 (m, 3H, N*H*), 7.77 – 7.69 (m, 1H, CH_{Ar}), 7.33 (dd, *J* = 8.7, 2.3 Hz, 1H, CH_{Ar}), 3.57 – 3.35 (m, 22H, CH_2), 3.14 – 2.92 (m, 7H, CH_2), 2.59 (dd, *J* = 8.7, 5.8 Hz, 3H, CH_2), 2.29 (d, *J* = 6.7 Hz, 7H, CH_2), 1.98 (s, 3H, CH_3), 1.81 (p, *J* = 6.7 Hz, 2H, CH_2), 1.60 (p, *J* = 6.7 Hz, 2H, CH_2), 1.50 (m, 4H, CH_2), 1.39 (m, 5H, CH_2), 1.30 – 1.12 (m, 5H, CH_2). **^{13}C{^{1}H} NMR:** (DMSO-d^6, 101 MHz, 298 K) δ (ppm) 172.5 (C=O), 171.8 (C=O), 171.7 (C=O), 171.6 (C=O), 166.5 (C=O), 139.1 (C_{qt}), 135.2 (C_{qt}), 132.0 (CH_{Ar}), 131.5 (CH_{Ar}), 130.2 (C_{qt}), 127.8 (CH_{Ar}), 127.5 (CH_{Ar}), 125.7 (CH_{Ar}), 120.1 (CH_{Ar}), 116.1 (CH_{Ar}), 70.3 (CH_2), 70.2 (CH_2), 70.1 (CH_2), 70.0 (CH_2), 68.8 (CH_2), 68.5 (CH_2), 47.5 (CH_2), 47.3 (CH_2), 39.3 (CH_2), 38.9 (CH_2), 37.3 (CH_2), 36.3 (CH_2), 31.4 (CH_2), 30.4 (CH_2), 29.9 (CH_2), 29.8 (CH_2), 29.3 (CH_2), 28.0 (CH_2), 26.5 (CH_2), 24.0 (CH_2), 20.8 (CH_3). **HR-ESI-MS:** *m/z* calcd. for $[C_{50}H_{79}N_{11}O_{14}+H]^+$ 1058.58890, found 1058.58818. **HPLC:** R_t = 9.69 min (40 to 95% MeOH with 0.1% TFA, 11 min).

Synthesis of DFO-PEG$_3$-Benzophenone (12)

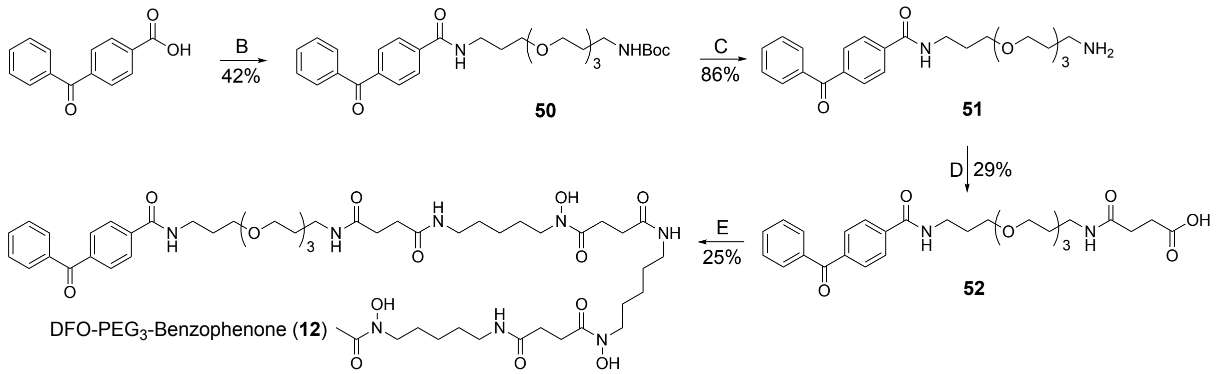

Scheme S10. Synthetic pathway toward DFO-PEG$_3$-Benzophenone (**12**)

Compound 50

Compound **50** was prepared from 4-benzoylbenzoic acid (301 mg, 1.33 mmol) according to **General Procedure B** to give **50** as a pale-yellow oil (423 mg, 42% yield). **^{1}H NMR:** ($CDCl_3$, 400 MHz, 298 K) δ (ppm) 7.93 (d, *J* = 8.2 Hz, 2H, CH_{Ar}), 7.86 – 7.76 (m, 4H, CH_{Ar}), 7.61 (t, *J* = 7.4 Hz, 1H, CH_{Ar}), 7.49 (t, *J* = 7.7 Hz, 2H, CH_{Ar}), 3.72 – 3.56 (m, 10H, CH_2), 3.50 – 3.40 (m, 4H, CH_2), 3.16 (t, *J* = 6.5 Hz, 2H, CH_2), 1.92 (p, *J* = 5.7 Hz, 2H, CH_2), 1.69 (p, *J* = 6.4 Hz, 2H, CH_2), 1.41 (s, 9H, $C(CH_3)_3$). **^{13}C{^{1}H} NMR:** ($CDCl_3$, 101 MHz, 298 K) δ (ppm) 196.2 (C=O), 166.6 (C=O), 156.5 (C=O), 140.0 (C_{qt}), 138.1 (C_{qt}), 137.2 (C_{qt}), 133.0 (CH_{Ar}), 130.2 (CH_{Ar}), 130.2 (CH_{Ar}), 128.6 (CH_{Ar}), 127.2 (CH_{Ar}), 71.1 (CH_2), 70.6 (CH_2), 70.6 (CH_2), 70.4 (CH_2), 70.2 (CH_2), 69.6 (CH_2), 39.5 (CH_2), 38.8 (CH_2), 29.7 (CH_2), 28.8 (CH_2), 28.6 ($C(\underline{C}H_3)_3$). **HR-ESI-MS:** *m/z* calcd. for $[C_{29}H_{40}N_2O_7+Na]^+$ 551.27277, found 551.27209.

Compound 51

Compound **51** was prepared from compound **50** (423 mg, 0.39 mmol) according to **General Procedure C** to give **51** as a pale-yellow oil (293 mg, 86% yield). **^{1}H NMR:** ($CDCl_3$, 400 MHz, 298 K) δ (ppm) 7.95 (d, *J* = 8.3 Hz, 2H, CH_2), 7.85 – 7.75 (m, 5H, CH_2), 7.65 – 7.57 (m, 2H, CH_2), 7.49 (t, *J* = 7.6 Hz, 2H, CH_2), 5.44 (s, 1H, N*H*), 3.74 (t, *J* = 5.3 Hz, 2H, CH_2), 3.67 – 3.50 (m, 12H, CH_2), 3.25 (q, *J* = 5.5 Hz, 2H, CH_2), 2.02 – 1.85 (m, 4H, CH_2). **^{13}C{^{1}H} NMR:** ($CDCl_3$, 101 MHz, 298 K) δ (ppm) 170.4 (C=O), 167.6 (C=O), 159.3 (C_{qt}), 152.3 (C_{qt}), 148.6 (C_{qt}), 133.1 (CH_{Ar}), 130.5 (CH_{Ar}), 130.2 (CH_{Ar}), 128.6 (CH_{Ar}), 127.4 (CH_{Ar}), 71.1 (CH_2), 70.4 (CH_2), 69.9 (CH_2), 69.8 (CH_2), 69.4 (CH_2), 69.2 (CH_2), 40.9 (CH_2), 37.9 (CH_2), 29.5 (CH_2), 26.1 (CH_2). **HR-ESI-MS:** *m/z* calcd. for $[C_{24}H_{32}N_2O_5+H]^+$ 429.23840, found 429.23842.

Compound 52

Compound **52** was prepared from compound **51** (293 mg, 0.68 mmol) according to **General Procedure D** to give **52** as an off-white solid (103 mg, 29% yield). **^{1}H NMR:** ($CDCl_3$, 400 MHz, 298 K) δ (ppm) 7.91 (d, *J* = 8.3 Hz, 2H, CH_{Ar}), 7.84 – 7.73 (m, 4H, CH_{Ar}), 7.68 (t, *J* = 5.6 Hz, 1H, CH_{Ar}), 7.64 – 7.58 (m, 1H, CH_{Ar}), 7.48 (t, *J* = 7.7 Hz, 2H, CH_{Ar}), 7.09 (t, *J* = 5.6 Hz, 1H, CH_{Ar}), 3.64 (d, *J* = 3.9 Hz, 6H, CH_2), 3.61 – 3.55 (m, 4H, CH_2), 3.52 – 3.44 (m, 5H, CH_2), 3.30 (q, *J* = 6.1 Hz, 2H, CH_2), 2.63 (t, *J* = 6.6 Hz, 2H, CH_2), 2.50 (t, *J* = 6.6 Hz, 2H, CH_2), 1.90 (q, *J* = 6.0 Hz, 2H, CH_2), 1.71 (p, *J* = 6.1 Hz, 2H, CH_2). **^{13}C{^{1}H} NMR:** ($CDCl_3$, 101 MHz, 298 K) δ (ppm) 196.5 (C=O), 175.4 (C=O), 173.5 (C=O), 167.5 (C=O), 140.2 (C_{qt}), 137.6 (C_{qt}), 137.0 (C_{qt}), 133.2 (CH_{Ar}), 130.2 (CH_{Ar}), 130.2 (CH_{Ar}), 128.6 (CH_{Ar}), 127.3 (CH_{Ar}), 70.4 (CH_2), 70.3 (CH_2), 70.3 (CH_2), 70.1 (CH_2), 70.0 (CH_2), 69.7 (CH_2), 39.1 (CH_2), 38.1

(CH_2), 30.9 (CH_2), 30.0 (CH_2), 28.8, (CH_2) 28.6 (CH_2). **HR-ESI-MS:** *m/z* calcd. for $[C_{28}H_{36}N_2O_8+Na]^+$ 551.23639, found 551.23690.

DFO-PEG3-Benzophenone (12)

DFO-PEG3-Benzophenone, compound **12**, was prepared from compound **52** (102 mg, 0.19 mmol) according to **General Procedure E** to give **12** as an off-white solid (47 mg, 25% yield). **^{1}H NMR:** (DMSO-d^6, 400 MHz, 298 K) δ (ppm) 8.65 (t, J = 5.6 Hz, 1H, NH), 7.99 (d, J = 8.4 Hz, 2H, CH_{Ar}), 7.81 – 7.67 (m, 8H, CH_{Ar} + NH), 7.58 (t, J = 7.7 Hz, 2H, CH_{Ar}), 3.56 – 3.42 (m, 14H, CH_2), 3.35 (dt, J = 13.6, 6.6 Hz, 4H, CH_2), 3.10 – 2.96 (m, 6H, CH_2), 2.57 (t, J = 6.5 Hz, 3H, CH_2), 2.54 (s, 9H, CH_2), 1.96 (s, 3H, CH_3), 1.78 (p, J = 6.6 Hz, 2H, CH_2), 1.59 (p, J = 6.7 Hz, 3H, CH_2), 1.52 – 1.45 (m, 3H, CH_2), 1.38 (m, 4H, CH_2), 1.21 (m, 3H, CH_2). **^{13}C{^{1}H} NMR:** (DMSO-d^6, 101 MHz, 298 K) δ (ppm) 195.4 (C=O), 173.1 (C=O), 171.3 (C=O), 171.2 (C=O), 165.4 (C=O), 162.6 (C=O), 139.0 (C_{qt}), 138.0 (C_{qt}), 136.7 (C_{qt}), 133.0 (CH_{Ar}), 129.7 (CH_{Ar}), 129.5 (CH_{Ar}), 128.7 (CH_{Ar}), 127.3 (CH_{Ar}), 69.8 (CH_2), 69.8 (CH_2), 69.6 (CH_2), 69.5 (CH_2), 68.3 (CH_2), 68.1 (CH_2), 53.6 (CH_2), 47.5 (CH_2), 47.1 (CH_2), 40.4 (CH_2), 38.4 (CH_2), 38.4 (CH_2), 38.4 (CH_2), 38.4 (CH_2), 36.8 (CH_2), 35.8 (CH_2), 30.9 (CH_2), 30.9 (CH_2), 29.9 (CH_2), 29.4 (CH_2), 29.3 (CH_2), 28.8 (CH_2), 27.6 (CH_2), 26.0 (CH_2), 23.5 (CH_2), 20.4 (CH_3). **HR-ESI-MS:** *m/z* calcd. for $[C_{53}H_{82}N_8O_{15}+Na]^+$ 1093.57918, found 1093.57860. **HPLC:** R_t = 8.80 min (40 to 95% MeOH with 0.1% TFA, 11 min).

Synthesis of DFO-PEG3-Diazirine (13)

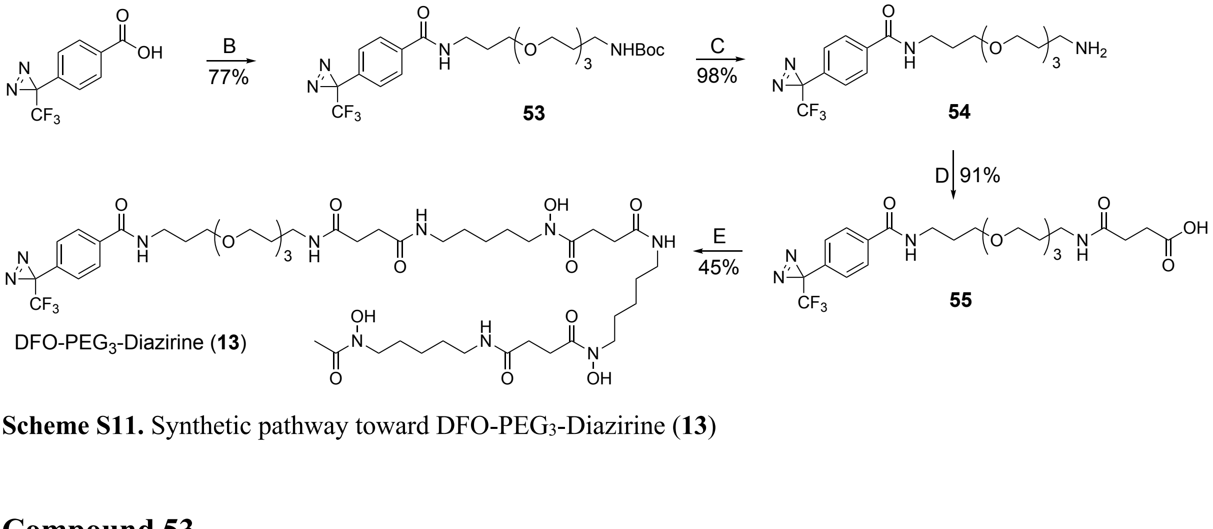

Scheme S11. Synthetic pathway toward DFO-PEG3-Diazirine (**13**)

Compound 53

Compound **53** was prepared from 4-[3-(trifluoromethyl)diazirin-3-yl]benzoic acid (14 mg, 0.07 mmol) according to **General Procedure B** to give **53** as a colorless oil (25 mg, 77% yield). **^{1}H NMR:** (MeOD-d^4, 400 MHz, 298 K) δ (ppm) 7.90 (d, J = 8.6 Hz, 2H, CH_{Ar}), 7.34 (d, J = 8.2

Hz, 2H, CH_{Ar}), 3.68 – 3.54 (m, 9H, CH_2), 3.54 – 3.52 (m, 1H, CH_2), 3.48 (td, *J* = 6.5, 2.8 Hz, 4H, CH_2), 3.11 (t, *J* = 6.8 Hz, 2H, CH_2), 1.88 (p, *J* = 6.5 Hz, 2H, CH_2), 1.70 (p, *J* = 6.5 Hz, 2H, CH_2), 1.42 (s, 9H, $C(CH_3)_3$). **$^{13}C\{^1H\}$ NMR:** (MeOD-d^4, 101 MHz, 298 K) δ (ppm) 168.7 (C=O), 158.3 (C=O), 137.4 (C_{qt}), 133.0 (C_{qt}), 129.0 (CH_{Ar}), 127.7 (CH_{Ar}), 124.8 (C_{qt}), 122.1 (C_{qt}), 79.8 ($\underline{C}(CH_3)_3$), 71.5 (CH_2), 71.5 (CH_2), 71.3 (CH_2), 71.2 (CH_2), 70.2 (CH_2), 69.9 (CH_2), 38.8 (CH_2), 38.7 (CH_2), 30.9 (CH_2), 30.3 (CH_2), 28.8 ($C(\underline{C}H_3)_3$). **$^{19}F\{^1H\}$ NMR:** (MeOD-d^4, 376 MHz, 298 K) δ –66.8. **HR-ESI-MS:** *m/z* calcd. for $[C_{24}H_{35}F_3N_4O_7+H]^+$ 533.25815, found 533.25818.

Compound 54

Compound **54** was prepared from compound **53** (25 mg, 0.05 mmol) according to **General Procedure C** to give **54** as a colorless oil (25 mg, 98% yield). **^1H NMR:** (MeOD-d^4, 400 MHz, 298 K) δ (ppm) 7.91 (d, *J* = 8.6 Hz, 2H, CH_{Ar}), 7.34 (d, *J* = 8.2 Hz, 2H, CH_{Ar}), 3.68 – 3.59 (m, 10H, CH_2), 3.57 (t, *J* = 6.0 Hz, 2H, CH_2), 3.48 (t, *J* = 7.0 Hz, 2H, CH_2), 3.10 (t, *J* = 6.4 Hz, 2H, CH_2), 1.90 (ddt, *J* = 15.3, 13.0, 6.2 Hz, 4H, CH_2), 1.41 – 1.26 (m, 2H, CH_2). **$^{13}C\{^1H\}$ NMR:** (MeOD-d^4, 101 MHz, 298 K) δ (ppm) 168.8 (C=O), 137.2 (C_{qt}), 133.1 (C_{qt}), 129.0 (CH_{Ar}), 127.7 (CH_{Ar}), 124.8 (C_{qt}), 122.1 (C_{qt}), 71.4 (CH_2), 71.1 (CH_2), 71.1 (CH_2), 71.0 (CH_2), 70.3 (CH_2), 69.9 (CH_2), 40.1 (CH_2), 38.6 (CH_2), 30.4 (CH_2), 28.0 (CH_2). **$^{19}F\{^1H\}$ NMR:** (MeOD-d^4, 376 MHz, 298 K) δ (ppm) –66.9. **HR-ESI-MS:** *m/z* calcd. for $[C_{19}H_{27}F_3N_4O_4+H]^+$ 433.20572, found 433.20538.

Compound 55

Compound **55** was prepared from compound **54** (25 mg, 0.05 mmol) according to **General Procedure D** to give **55** as an off-white solid (28 mg, 91% yield). **^1H NMR:** (MeOD-d^4, 400 MHz, 298 K) δ (ppm) 7.90 (d, *J* = 8.6 Hz, 2H, CH_{Ar}), 7.34 (d, *J* = 8.2 Hz, 2H, CH_{Ar}), 3.69 – 3.44 (m, 15H, CH_2), 3.24 (t, *J* = 6.8 Hz, 2H, CH_2), 2.70 (s, 2H, CH_2), 2.62 – 2.54 (m, 2H, CH_2), 2.44 (t, *J* = 6.9 Hz, 2H, CH_2), 1.88 (p, *J* = 6.5 Hz, 2H, CH_2), 1.73 (p, *J* = 6.5 Hz, 2H, CH_2). **$^{13}C\{^1H\}$ NMR:** (MeOD-d^4, 101 MHz, 298 K) δ (ppm) 176.2 (C=O), 174.4 (C=O), 168.7 (C=O), 137.4 (C_{qt}), 133.0 (C_{qt}), 129.0 (CH_{Ar}), 127.7 (CH_{Ar}), 124.8 (C_{qt}), 122.1 (C_{qt}), 71.5 (CH_2), 71.5 (CH_2), 71.3 (CH_2), 71.2 (CH_2), 70.2 (CH_2), 69.8 (CH_2), 38.8 (CH_2), 37.8 (CH_2), 35.4 (CH_2), 31.6 (CH_2), 30.4 (CH_2), 30.3 (CH_2). **$^{19}F\{^1H\}$ NMR:** (MeOD-d^4, 376 MHz, 298 K) δ (ppm) –66.9. **HR-ESI-MS:** *m/z* calcd. for $[C_{23}H_{31}F_3N_4O_7+H]^+$ 533.22176, found 533.22147.

DFO-PEG_3-Diazirine (13)

DFO-PEG3-Diazirine, compound **13**, was prepared from compound **55** (26 mg, 0.05 mmol) according to **General Procedure E** to give **13** as an off-white solid (23 mg, 45% yield). **^{1}H NMR:** (DMSO-d^{6}, 400 MHz, 298 K) δ (ppm) 9.62 (br s, 3H, O*H*), 8.58 (t, *J* = 5.6 Hz, 1H, N*H*), 7.94 (d, *J* = 8.5 Hz, 2H, CH_{Ar}), 7.77 (m, 3H, N*H*), 7.37 (d, *J* = 8.1 Hz, 2H, CH_{Ar}), 3.48 (m, 17H, CH_2), 3.37 (t, *J* = 6.4 Hz, 3H, CH_2), 3.31 (m, 8H, CH_2), 3.05 (m, 2H, CH_2), 3.02 – 2.94 (m, 6H, CH_2), 2.57 (t, *J* = 7.4 Hz, 4H, CH_2), 2.26 (s, 9H, CH_2), 1.96 (s, 3H, CH_3), 1.75 (p, *J* = 6.5 Hz, 2H, CH_2), 1.59 (p, *J* = 6.7 Hz, 3H, CH_2), 1.48 (m, 6H, CH_2), 1.37 (m, 8H, CH_2), 1.22 (m, 4H, CH_2). **^{13}C{^{1}H} NMR:** (DMSO-d^{6}, 126 MHz, 298 K) δ (ppm) 172.0 (C=O), 171.3 (C=O), 171.2 (C=O), 171.2 (C=O), 171.1 (C=O), 171.0 (C=O), 170.1 (C=O), 165.1 (C=O), 136.2 (C_{qt}), 130.1 (C_{qt}), 128.1 (CH_{Ar}), 126.4 (CH_{Ar}), 122.9 (C_{qt}), 120.7 (C_{qt}), 69.8 (CH_2), 69.7 (CH_2), 69.6 (CH_2), 69.5 (CH_2), 68.2 (CH_2), 68.0 (CH_2), 47.1 (CH_2), 46.8 (CH_2), 38.4 (CH_2), 38.4 (CH_2), 38.3 (CH_2), 38.3 (CH_2), 36.8 (CH_2), 35.8 (CH_2), 30.9 (CH_2), 30.9 (CH_2), 29.9 (CH_2), 29.4 (CH_2), 29.4 (CH_2), 29.2 (CH_2), 28.8 (CH_2), 27.6 (CH_2), 26.0 (CH_2), 23.5, (CH_2) 20.3 (CH_3). **^{19}F{^{1}H} NMR:** (DMSO-d^{6}, 376 MHz, 298 K) δ (ppm) –66.9. **HR-ESI-MS** *m/z* calcd. for $[C_{48}H_{77}F_3N_{10}O_{14}+H]^{+}$ 1075.56456, found 1075.56403. **HPLC:** R_t = 9.64 min (40 to 95% MeOH with 0.1% TFA, 11 min).

Synthesis of DFO-PEG3-OMe-Tetrazole (14)

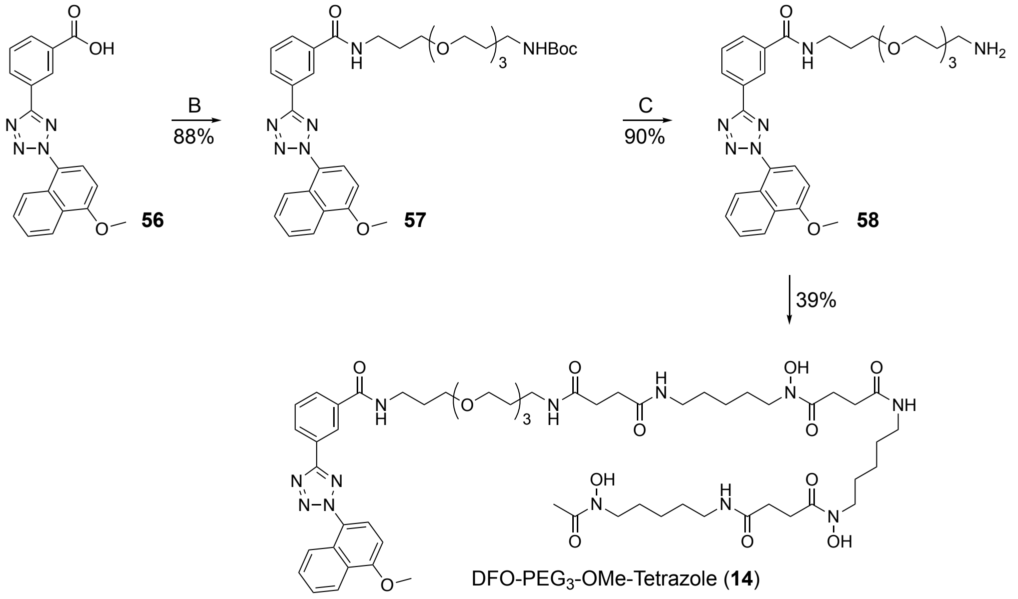

Scheme S12. Synthetic pathway toward DFO-PEG3-OMe-Tetrazole (**14**)

Compound 56

Compound **56** was prepared in accordance with the reported procedure.[6]⁶ **^{1}H NMR:** (DMSO-d^6, 400 MHz, 298 K) δ (ppm) 13.34 (br s, 1H, O*H*), 8.74 (s, 1H, CH_{Ar}), 8.44 (d, *J* = 7.5 Hz, 1H, CH_{Ar}), 8.39-8.33 (m, 1H, CH_{Ar}), 8.16 (d, *J* = 7.5. Hz, 1H, CH_{Ar}), 8.00 (d, *J* = 8.5 Hz, 1H, CH_{Ar}), 7.78 (t, *J* = 7.5 Hz, 1H, CH_{Ar}) 7.75-7.67 (m, 3H, CH_{Ar}), 7.23 (d, *J* = 8.5 Hz, 1H, CH_{Ar}), 4,12 (s, 3H, CH_3).**^{13}C{^{1}H} NMR:** (DMSO-d^6, 101 MHz, 298 K) δ (ppm) 166.7 (C=O), 163.7 (C_{qt}), 132.0 (C_{qt}), 131.4 (C_{qt}), 130.7 (CH_{Ar}), 129.9 (CH_{Ar}), 128.9 (CH_{Ar}), 127.9 (CH_{Ar}), 127.1 (C_{qt}), 126.8 (CH_{Ar}), 125.7 (C_{qt}), 125.3 (C_{qt}), 124.9 (C_{qt}), 122.2 (CH_{Ar}), 121.9 (CH_{Ar}), 103.7 (CH_{Ar}), 56.3 (OCH_3). **HR-ESI-MS:** *m/z* calcd. for $[C_{19}H_{13}O_3N_4–H]^-$ 345.09931, found 345.09903.

Compound 57

Compound **57** was prepared from compound **56** (100 mg, 0.29 mmol) according to **General Procedure B** to give **57** as a pale-yellow oil (165 mg, 88% yield). Note: The crude residue was purified by flash column chromatography (SiO_2, EtOAc). R_f = 0.28 (EtOAc**)** **^{1}H NMR: (**$CDCl_3$, 400 MHz, 298 K) δ (ppm) 8.66 (s, 1H, CH_{Ar}), 8.43-8.37 (m, 2H, CH_{Ar}), 8.02 (d, *J* = 7.9 Hz, 1H), 7.95-7.89 (m, 1H, CH_{Ar}), 7.81 (d, *J* = 8.3 Hz, 1H, CH_{Ar}), 7.65-7.57 (m, 3H, CH_{Ar}), 6.94 (d, *J* = 8.3 Hz, 1H), 4.89 (br s, 1H, N*H*), 4.12 (s, 3H, CH_3), 3.71-3.61 (m, 8H, CH_2), 3.54-3.49 (m, 2H, CH_2), 3.45-3.39 (m, 4H, CH_2), 3.19-3.13 (m, 2H, CH_2), 1.98-1.89 (m, 2H, CH_2), 1.72-1.64 (m, 2H, CH_2), 1.41 (s, 9H, $C(CH_3)_3$). **^{13}C{^{1}H} NMR:** ($CDCl_3$, 101 MHz, 298 K) δ (ppm) 166.7 (C=O), 164.6 (C_{qt}), 157.7 (C_{qt}), 156.2 (C=O), 135.9 (C_{qt}), 129.8 (CH_{Ar}), 129.6 (CH_{Ar}), 129.4 (CH_{Ar}), 128.7 (CH_{Ar}), 128.4 (C_{qt}), 127.7 (C_{qt}), 126.8 (C_{qt}), 126.6 (CH_{Ar}), 126.0 (C_{qt}), 125.4 (CH_{Ar}), 124.3 (CH_{Ar}), 122.7 (CH_{Ar}), 122.4 (CH_{Ar}), 102.7 (CH_{Ar}), 79.2 ($\underline{C}(CH_3)_3$), 70.8 (CH_2), 70.6 (CH_2), 70.5 (CH_2), 70.5 (CH_2), 70.2 (CH_2), 69.6 (CH_2), 56.1 (OCH_3), 39.3 (CH_2), 38.8 (CH_2), 29.7 (CH_2), 29.0 (CH_2), 28.6 ($C(\underline{C}H_3)_3$). **HR-ESI-MS:** *m/z* calcd. for $[C_{34}H_{44}N_6O_7+Na]^+$ 671.31747, found 671.31685.

Compound 58

Compound **58** was prepared from compound **57** (160 mg, 0.25 mmol) according to **General Procedure C** to give **58** as a pale-yellow oil (121 mg, 90 % yield). **^{1}H NMR:** (MeOD-d^4, 400 MHz, 298 K) δ (ppm) 8.70 (s, 1H, CH_{Ar}), 8.46 – 8.39 (m, 2H, CH_{Ar}), 8.01 (d, *J* = 7.8, 1H, CH_{Ar}), 7.86 (d, *J* = 8.4 Hz, 1H, CH_{Ar}), 7.83-7.77 (m, 1H, CH_{Ar}) 7.70 (t, *J* = 7.8 Hz, 1H, CH_{Ar}), 7.67 – 7.60 (m, 2H, CH_{Ar}), 7.14 (d, *J* = 8.4 Hz, 1H, CH_{Ar}), 4.15 (s, 3H, CH_3), 3.66 – 3.48 (m, 14H, CH_2), 2.73 (t, *J* = 6.6 Hz, 2H, CH_2), 1.92 (p, *J* = 6.6 Hz, 2H, CH_2), 1.70 (p, *J* = 6.6 Hz,

2H, CH_2). **$^{13}C\{^1H\}$ NMR:** ($CDCl_3$, 126 MHz, 298 K) δ (ppm) 169.7 (C=O), 165.8 (C_{qt}), 159.1 (C_{qt}), 137.0 (C_{qt}), 130.8 (CH_{Ar}), 130.6 (CH_{Ar}), 130.5 (CH_{Ar}), 129.8 (CH_{Ar}), 129.7 (C_{qt}), 129.0 (C_{qt}), 127.8 (C_{qt}), 127.6 (CH_{Ar}), 127.1 (C_{qt}), 126.7 (CH_{Ar}), 125.9 (CH_{Ar}), 123.6 (CH_{Ar}), 123.1 (C_{qt}), 104.0 (CH_{Ar}), 71.5 (CH_2), 71.5 (CH_2), 71.3 (CH_2), 71.1 (CH_2), 70.4 (CH_2), 70.3 (CH_2), 56.7 (OCH_3), 40.1 (CH_2), 38.9 (CH_2), 32.9 (CH_2), 30.4 (CH_2). **HR-ESI-MS:** *m/z* calcd. for $[C_{29}H_{36}N_6O_5+H]^+$ 549.28199, found 549.28235.

DFO-PEG$_3$-OMe-Tetrazole (14)

Compound **58** (34 mg, 0.062 mmol) was dissolved in DMSO (3 mL). HATU (47.1mg, 0.124 mmol), DFO-Succ (41 mg, 0.062 mmol), and DIPEA (32.4 µL, 0.186 mmol) were added to the reaction which was stirred overnight at rt. The crude reaction was purified by semi-preparative HPLC (60-100% MeOH). Compound **14** was isolated as an off-white solid (28.9 mg, 39% yield). **^1H NMR:** (DMSO-d^6, 400 MHz, 298 K) δ (ppm) 9.68 – 9.55(br s, 3H, O*H*), 8.72 (t, *J* = 5.6 Hz, 1H, N*H*), 8.67 (s, 1H, CH_{Ar}), 8.39-8.32 (m, 2H, CH_{Ar}), 8.06 (d, *J* = 8.0, 1H, CH_{Ar}), 8.00 (d, *J* = 8.3 Hz, 1H, CH_{Ar}), 7.89 – 7.56 (m, 8H CH_{Ar} +N*H*), 7.22 (d, *J* = 8.4 Hz, 1H, CH_{Ar}), 4.12 (s, 3H, CH_3), 3.52 – 3.31 (m, 20H, CH_2), 3.09 – 2.94 (m, 8H, CH_2), 2.57 (t, *J* = 7.3 Hz, 4H, CH_2), 2.32 – 2.21 (m, 8H, CH_2), 1.96 (s, 3H, CH_3), 1.79 (p, *J* = 6.7 Hz, 2H, CH_2), 1.59 (p, *J* = 6.7 Hz, 2H, CH_2), 1.54 – 1.43 (m, 6H, CH_2), 1.44 – 1.30 (m, 6H, CH_2), 1.26 – 1.14 (m, 6H, CH_2). **$^{13}C\{^1H\}$ NMR:** (DMSO-d^6, 126 MHz, 298 K) δ (ppm) 172.0 (C=O), 172.0 (C=O), 171.3 (C=O), 171.2 (C=O), 171.1 (C=O), 170.1 (C=O), 165.4 (C=O), 164.0 (C_{qt}), 157.01 (C_{qt}), 135.6 (C_{qt}), 129.5 (CH_{Ar}), 129.5 (CH_{Ar}), 129.1 (CH_{Ar}), 128.9 (CH_{Ar}), 128.0 (C_{qt}), 126.8 (C_{qt}), 126.8 (CH_{Ar}), 125.7 (C_{qt}), 125.4 (CH_{Ar}), 125.3 (CH_{Ar}), 124.9 (C_{qt}), 122.2 (CH_{Ar}), 121.9 (CH_{Ar}), 103.7 (CH_{Ar}), 69.8 (CH_2), 69.7 (CH_2), 69.6 (CH_2), 69.5 (CH_2), 68.3 (CH_2), 68.0 (CH_2), 56.4 (OCH_3), 47.1 (CH_2), 46.8 (CH_2), 38.4 (CH_2), 38.4 (CH_2), 36.8 (CH_2), 35.8 (CH_2), 30.9 (CH_2), 30.9 (CH_2), 29.9 (CH_2), 29.4 (CH_2), 29.3 (CH_2), 28.8 (CH_2), 27.6 (CH_2), 26.0 (CH_2), 23.5 (CH_2), 20.3 (CH_3). **HR-ESI-MS:** *m/z* calcd for $[C_{58}H_{86}N_{12}O_{15}+Na]^+$ 1213.62278, found, 1213.62316. **HPLC:** R_t = 9.30 min (40 to 95% MeOH with 0.1% TFA, 11 min).

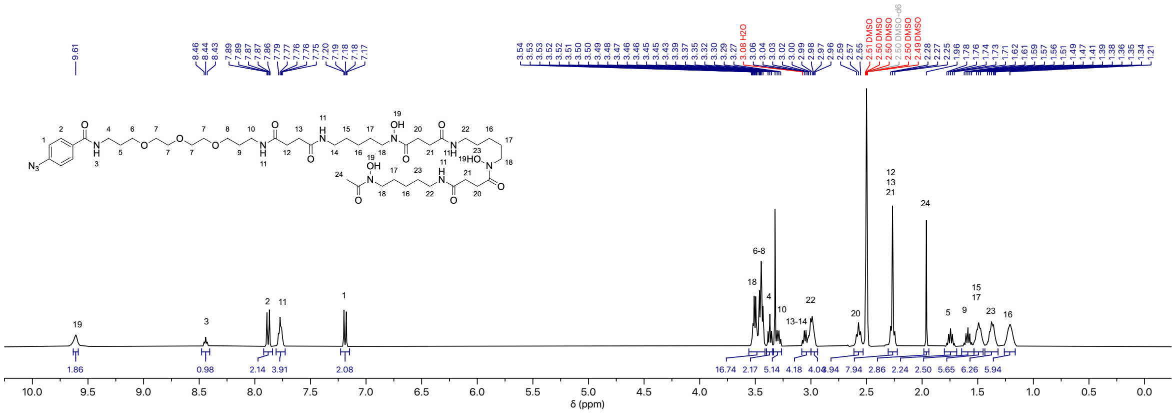

Figure S1. ^{1}H (400 MHz, DMSO-d^{6}, 298 K) NMR spectrum of compound **1**

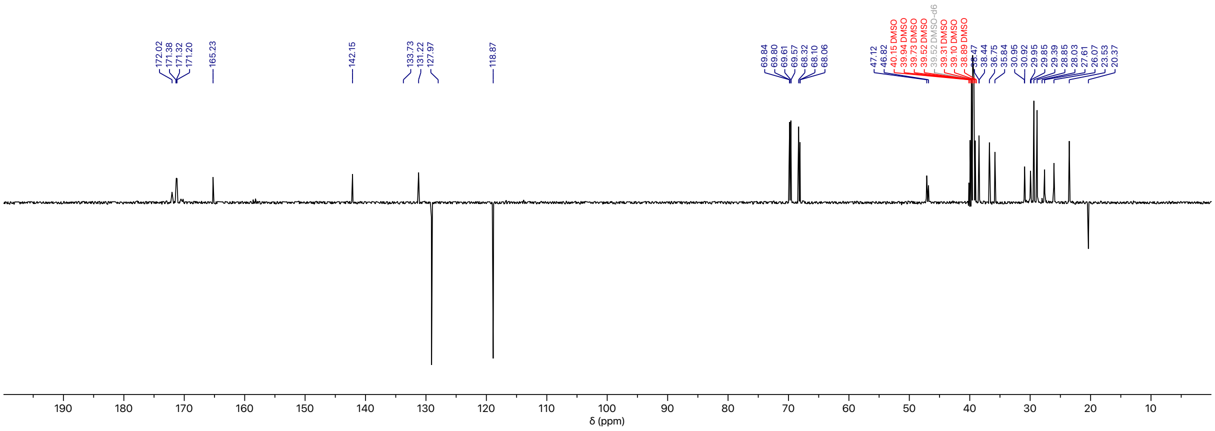

Figure S2. ^{13}C (101 MHz, DMSO-d^{6}, 298 K) NMR spectrum of compound **1**

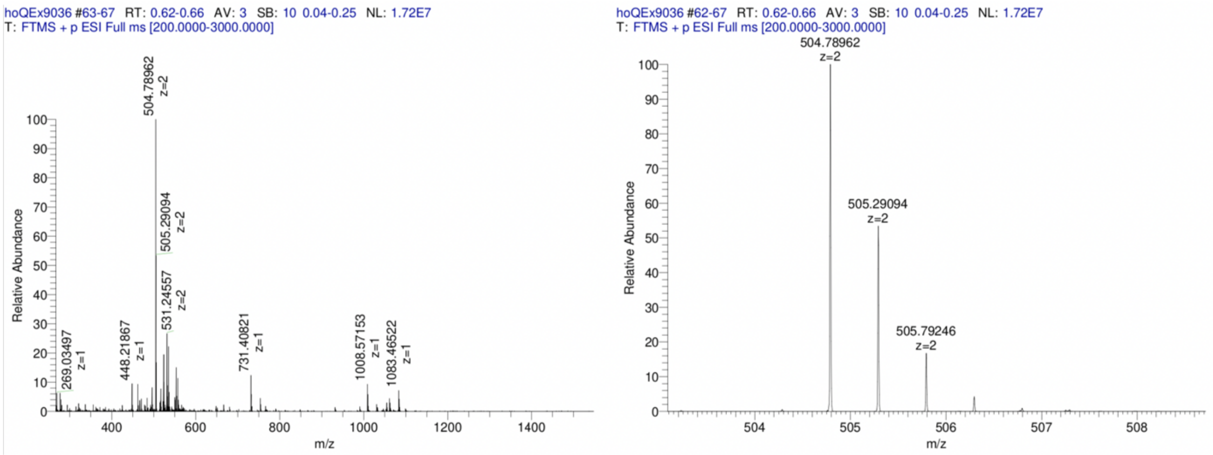

Figure S3. HR-ESI-MS spectrum of compound **1**

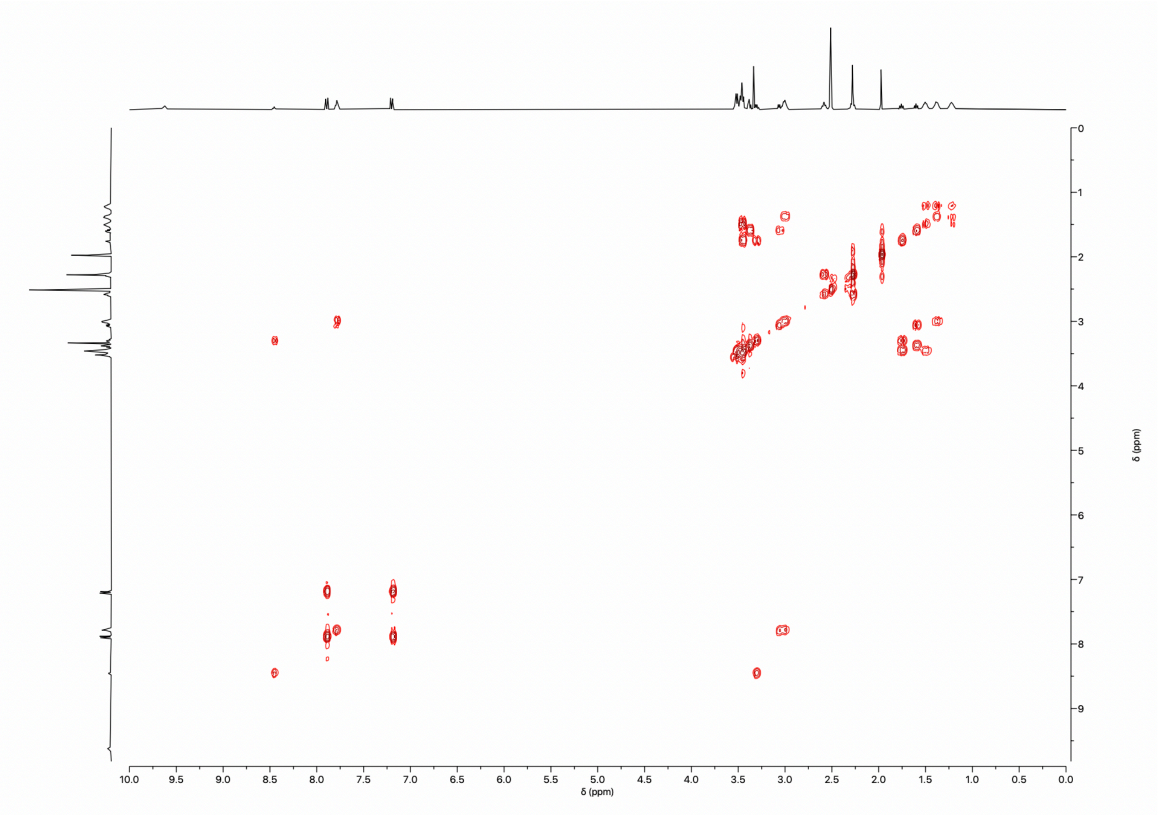

Figure S4. ^{1}H-^{1}H (COSY, DMSO-d^{6}, 298 K) 2D NMR spectrum of compound **1**

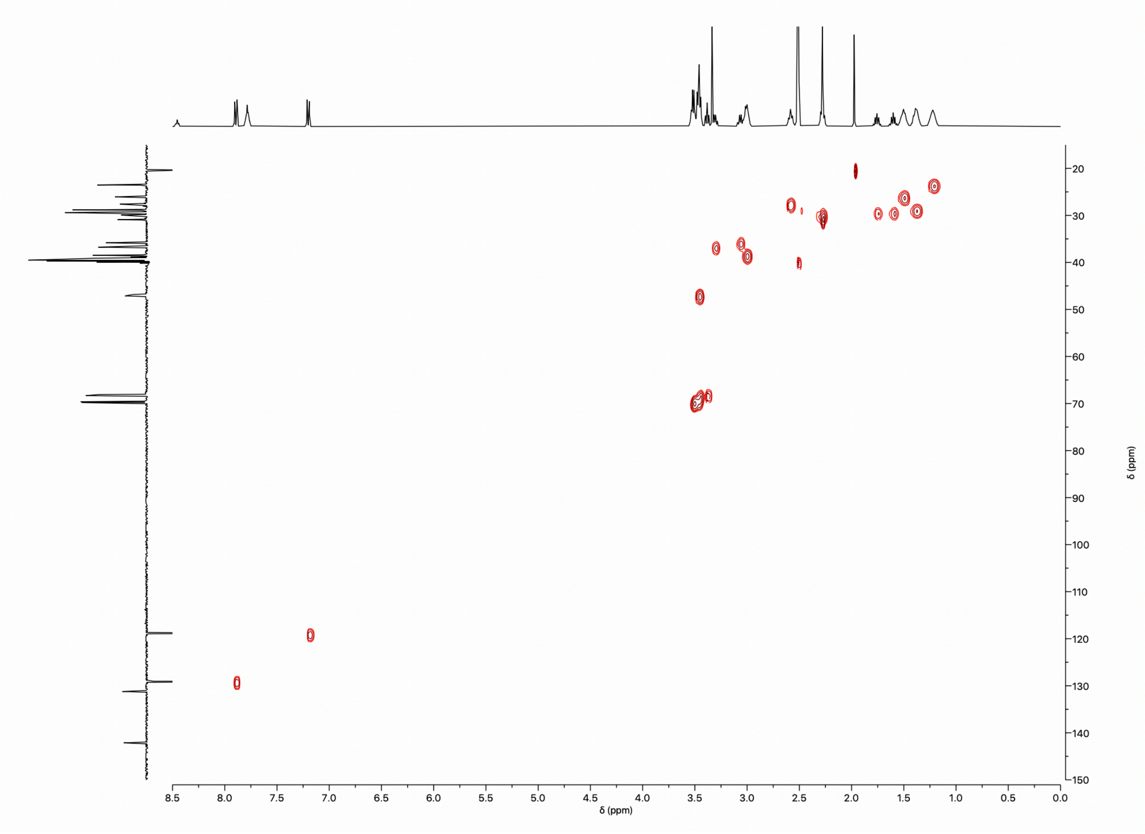

Figure S5. ^{1}H-^{13}C (HSQC, DMSO-d^{6}, 298 K) 2D NMR spectrum of compound **1**

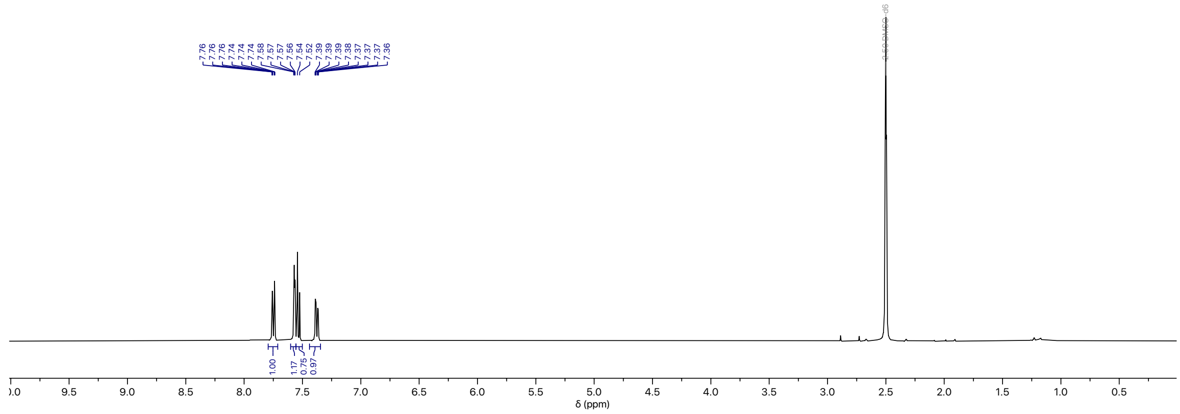

Figure S6. ^{1}H (400 MHz, DMSO-d^{6}, 298 K) NMR spectrum of compound **15**

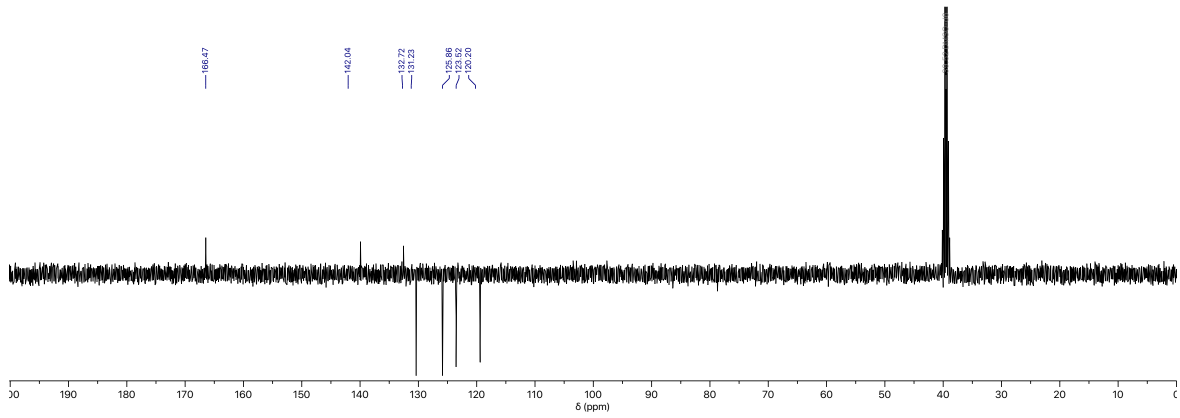

Figure S7. ^{13}C (101 MHz, DMSO-d^{6}, 298 K) NMR spectrum of compound **15**

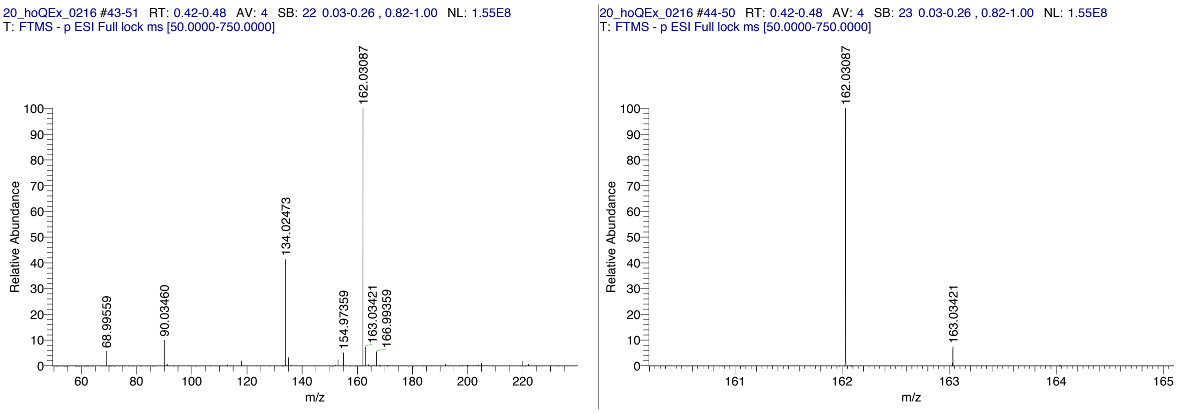

Figure S8. HR-ESI-MS spectrum of compound **15**

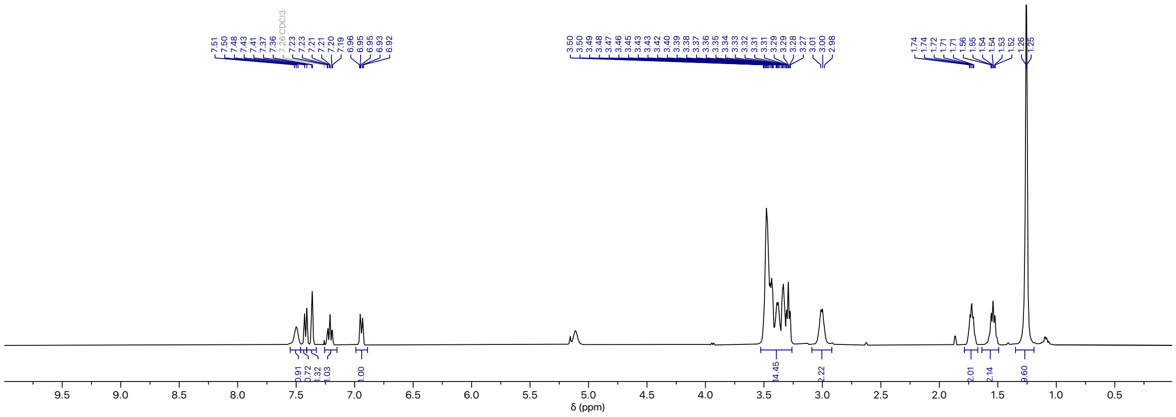

Figure S9. ^{1}H (400 MHz, $CDCl_3$, 298 K) NMR spectrum of compound **16**

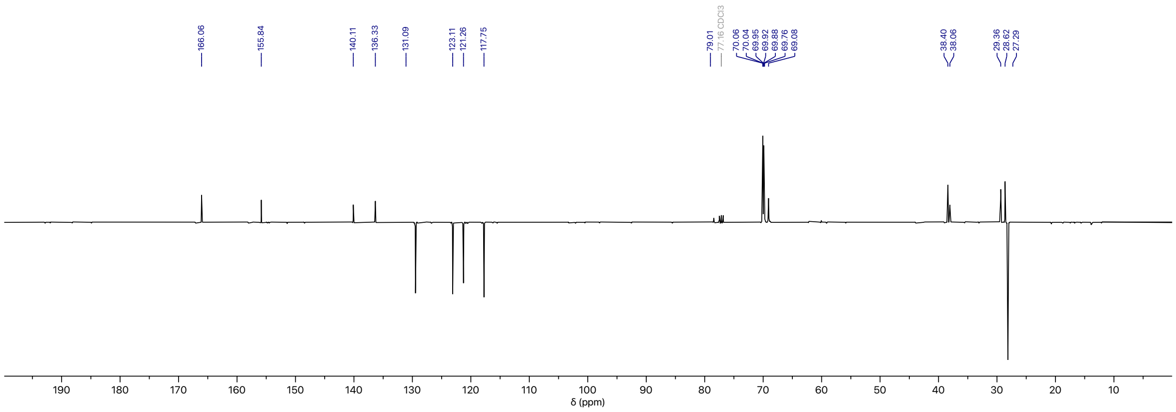

Figure S10. ^{13}C (101 MHz, $CDCl_3$, 298 K) NMR spectrum of compound **16**

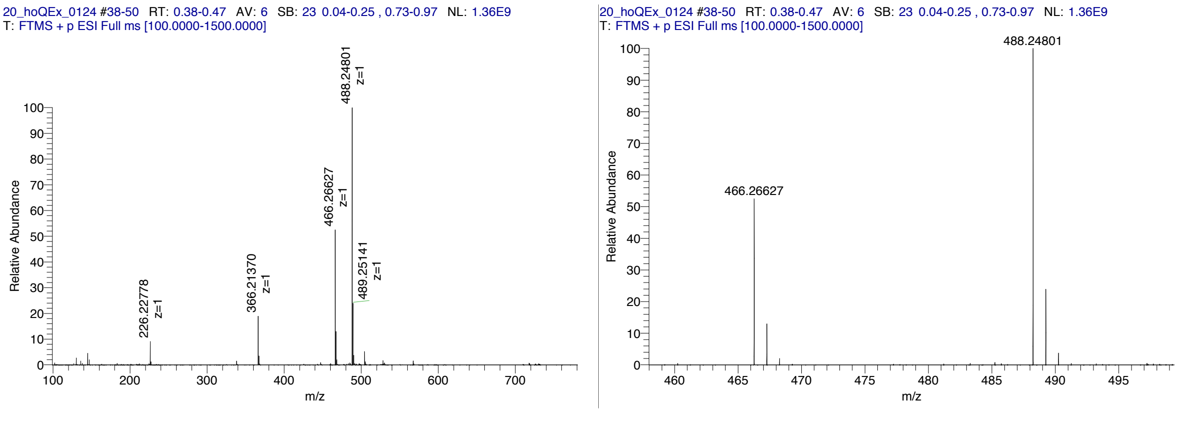

Figure S11. HR-ESI-MS spectrum of compound **16**

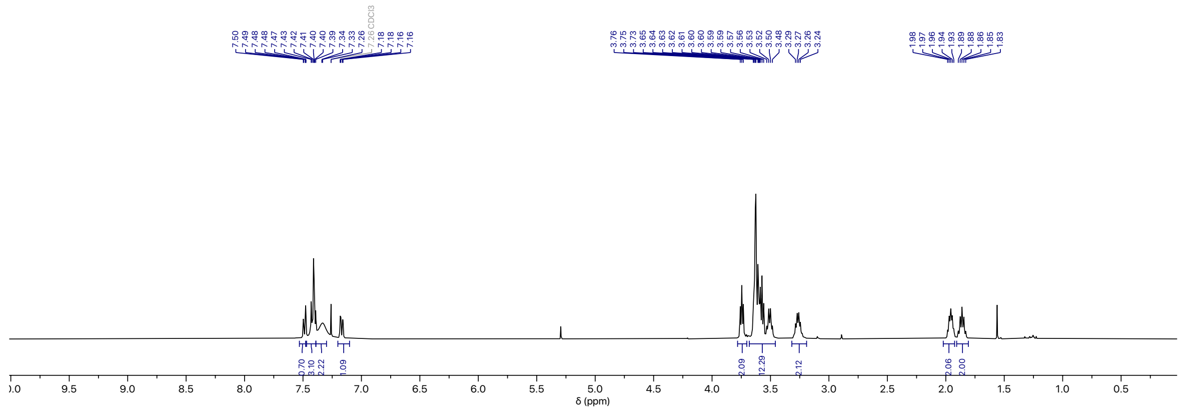

Figure S12. ^{1}H (400 MHz, $CDCl_3$, 298 K) NMR spectrum of compound **17**

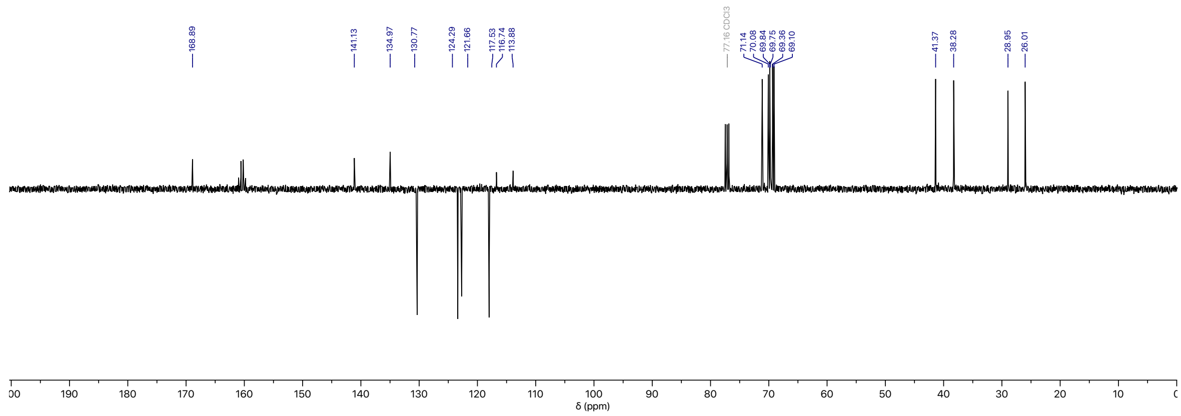

Figure S13. ^{13}C (101 MHz, $CDCl_3$, 298 K) NMR spectrum of compound **17**

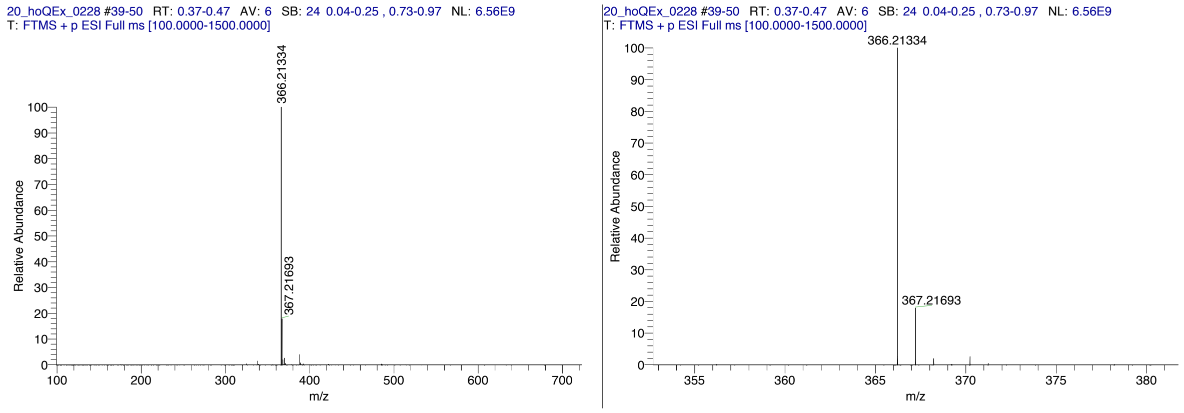

Figure S14. HR-ESI-MS spectrum of compound **17**

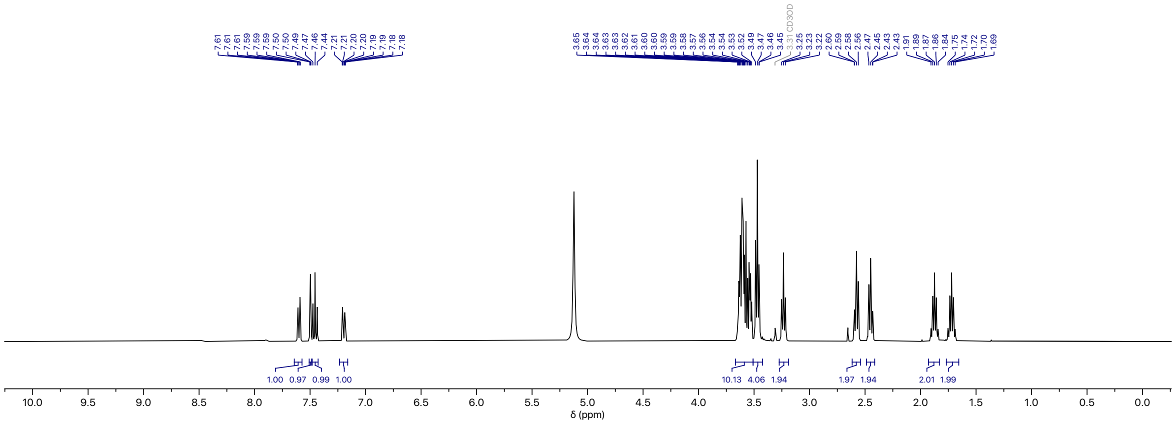

Figure S15. ^{1}H (400 MHz, MeOD-d^{4}, 298 K) NMR spectrum of compound **18**

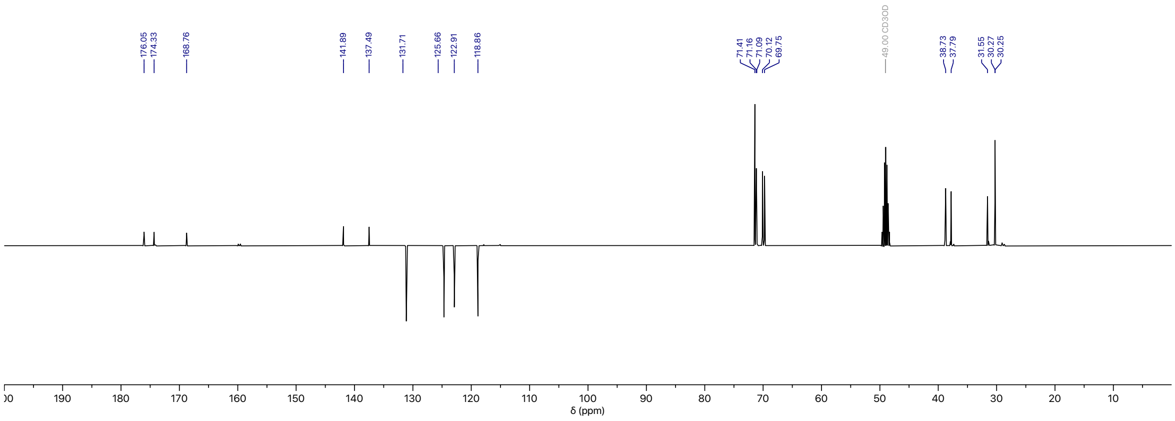

Figure S16. ^{13}C (101 MHz, MeOD-d^{4}, 298 K) NMR spectrum of compound **18**

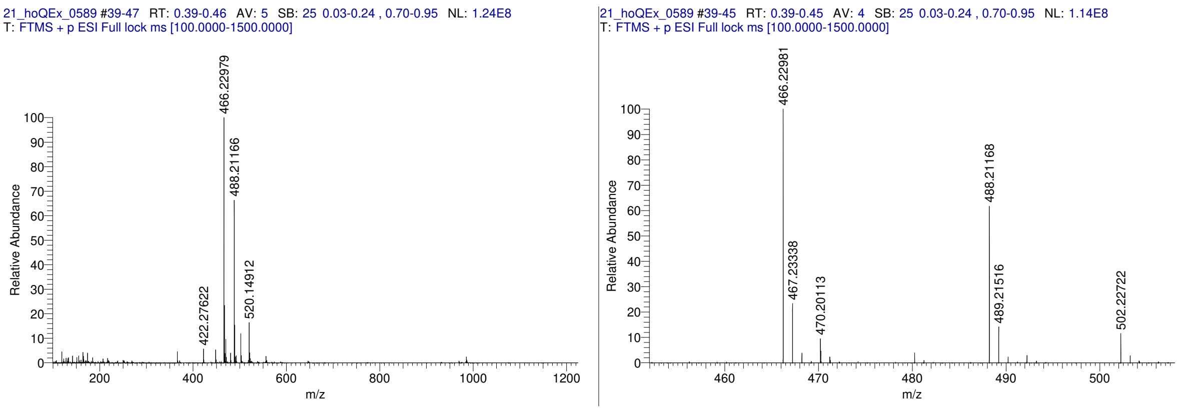

Figure S17. HR-ESI-MS spectrum of compound **18**

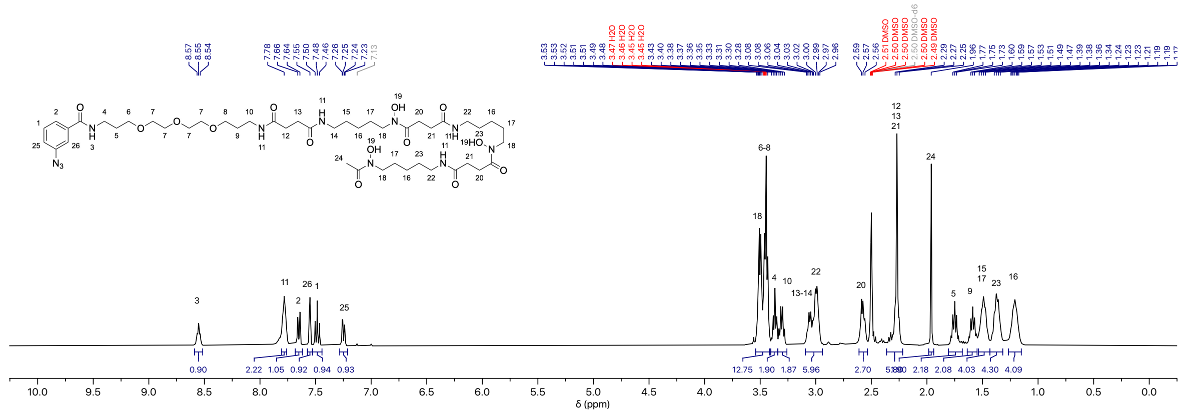

Figure S18. ^{1}H (400 MHz, DMSO-d^{6}, 298 K) NMR spectrum of compound **2**

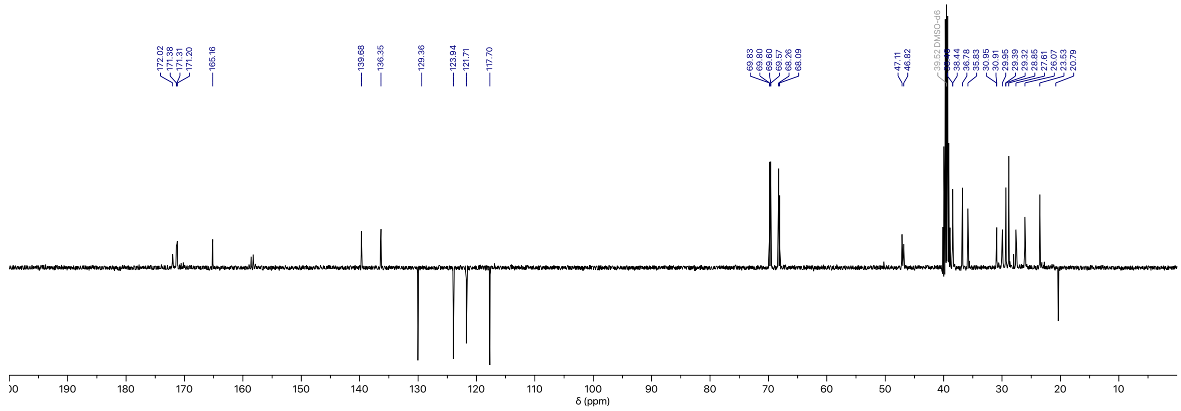

Figure S19. ^{13}C (101 MHz, DMSO-d^{6}, 298 K) NMR spectrum of compound **2**

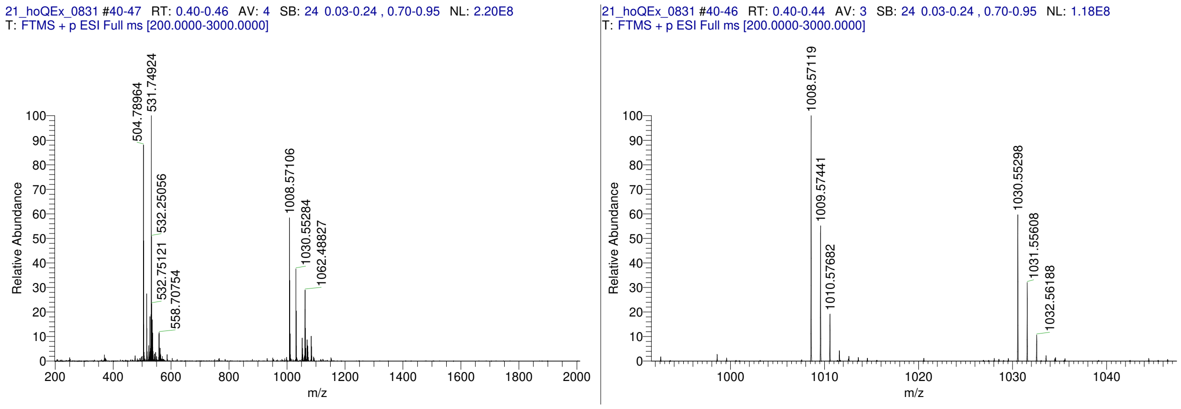

Figure S20. HR-ESI -MS spectrum of compound **2**

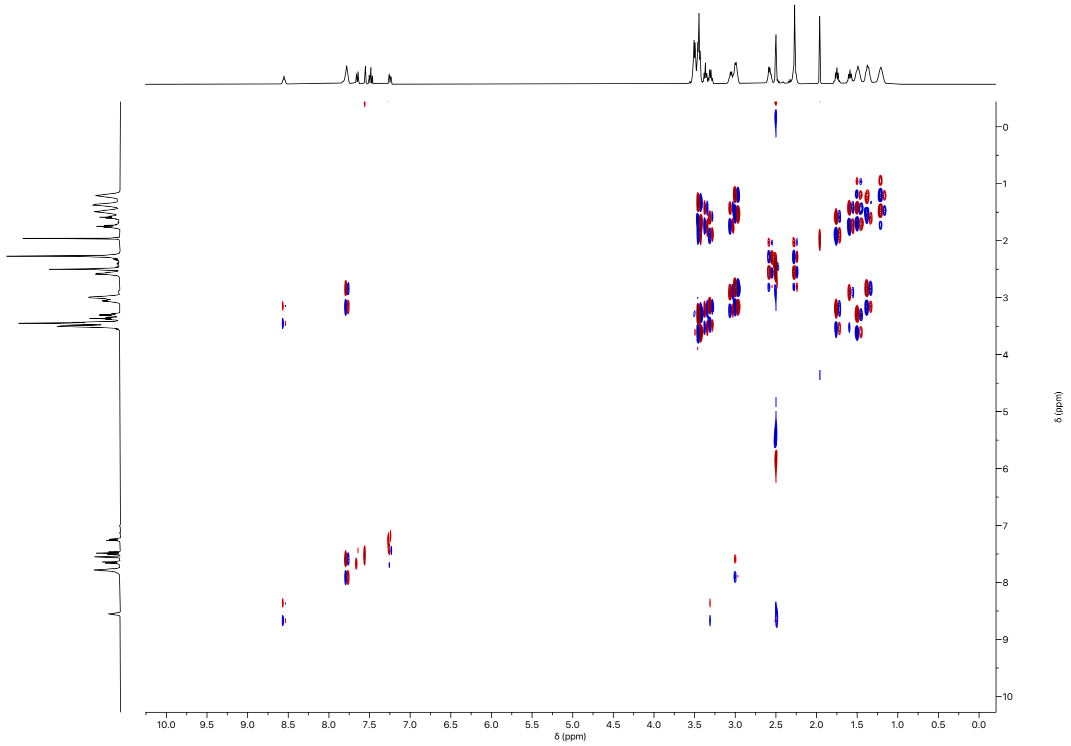

Figure S21. ^{1}H-^{1}H (COSY, DMSO-d^{6}, 298 K) 2D NMR spectrum of compound **2**

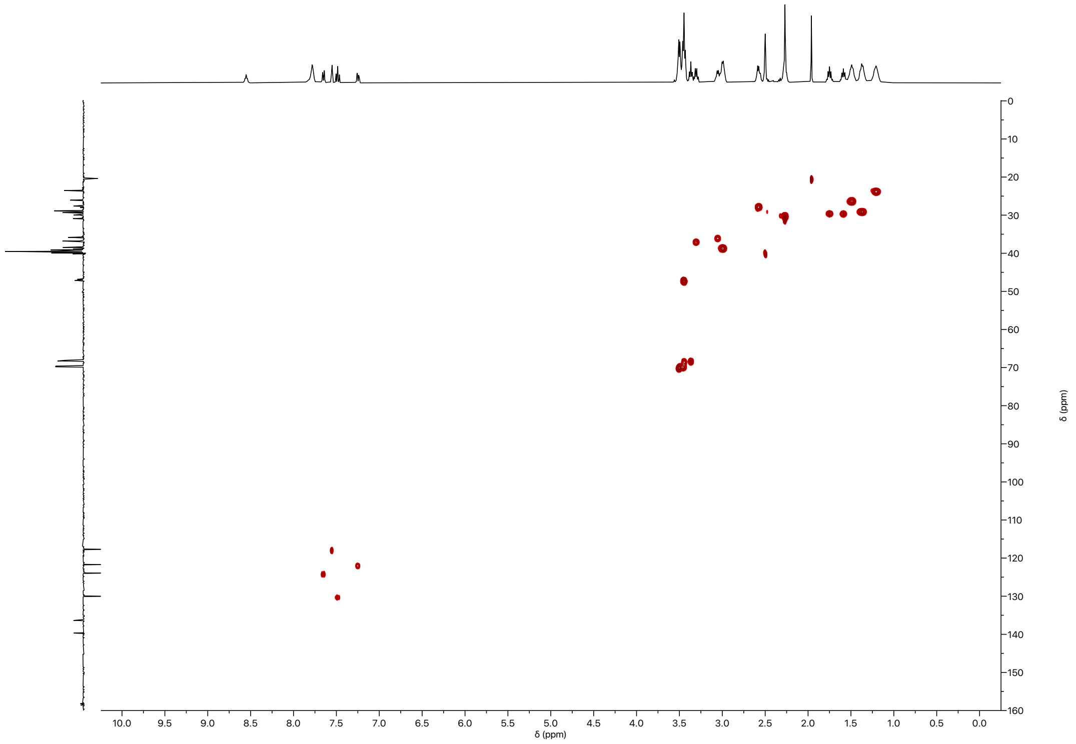

Figure S22. ^{1}H-^{13}C (HSQC, DMSO-d^{6}, 298 K) 2D NMR spectrum of compound **2**

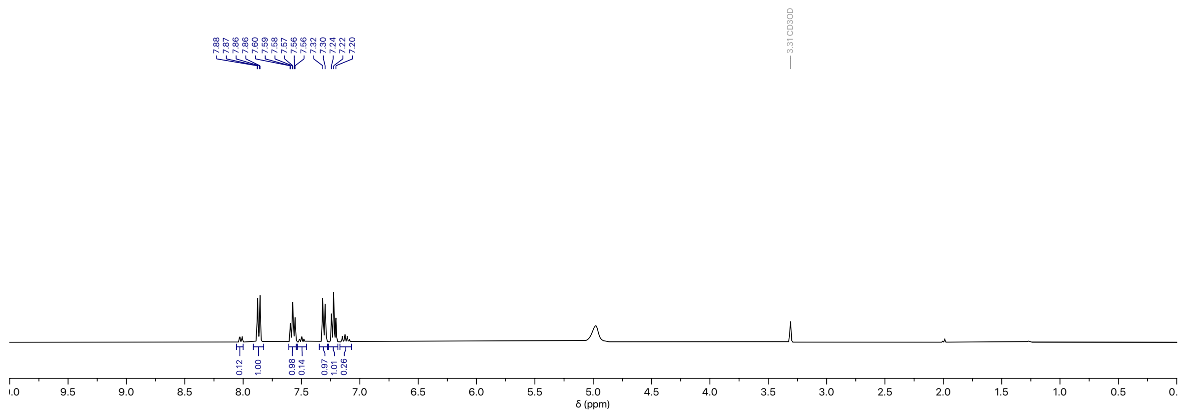

Figure S23. ^{1}H (400 MHz, MeOD-d^{4}, 298 K) NMR spectrum of compound **19**

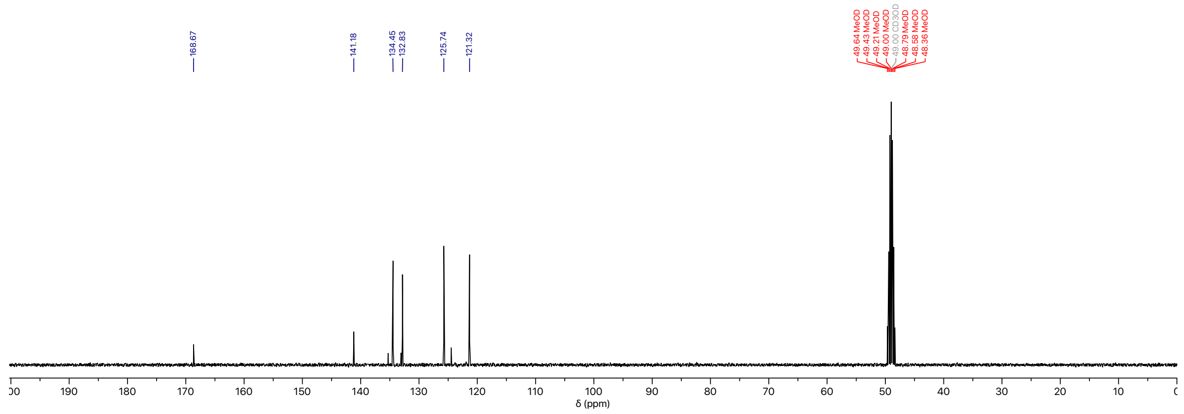

Figure S24. ^{13}C (101 MHz, MeOD-d^{4}, 298 K) NMR spectrum of compound **19**

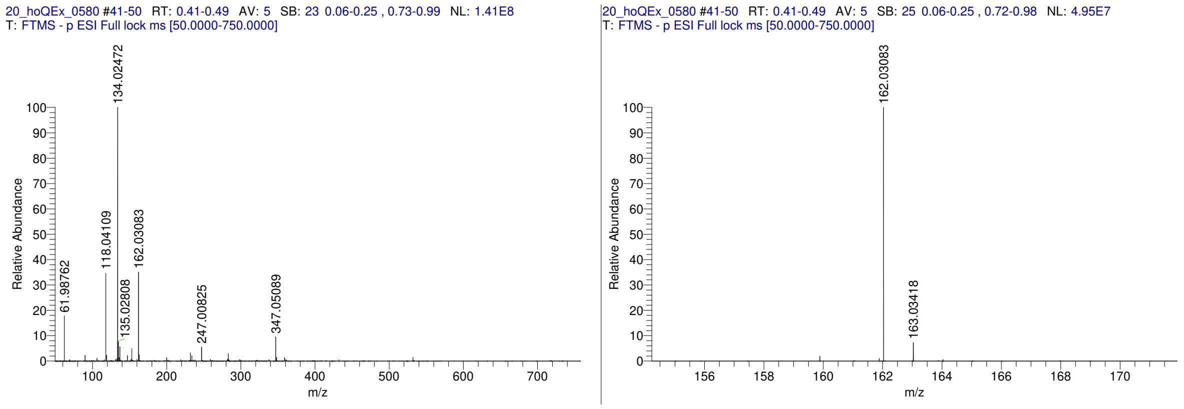

Figure S25. HR-ESI-MS spectrum of compound **19**

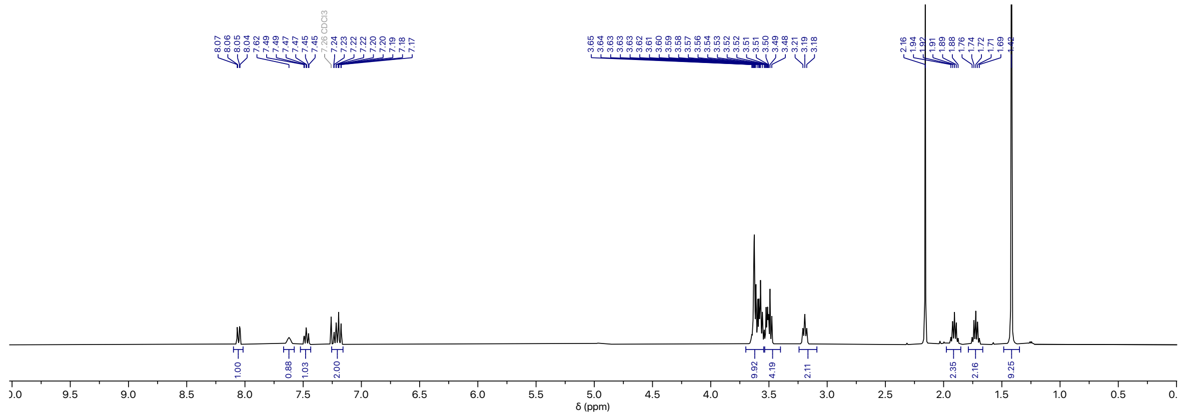

Figure S26. ^{1}H (400 MHz, $CDCl_3$, 298 K) NMR spectrum of compound **20**

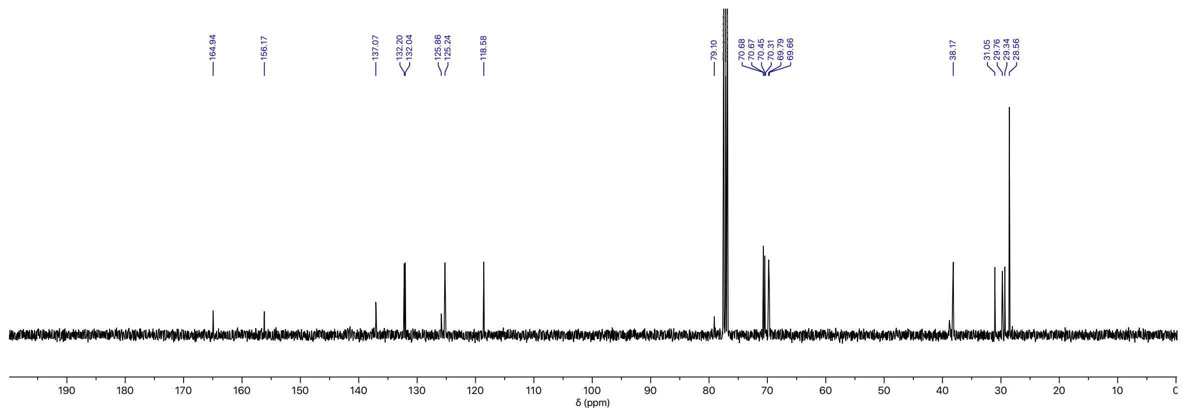

Figure S27. ^{13}C (101 MHz, $CDCl_3$, 298 K) NMR spectrum of compound **20**

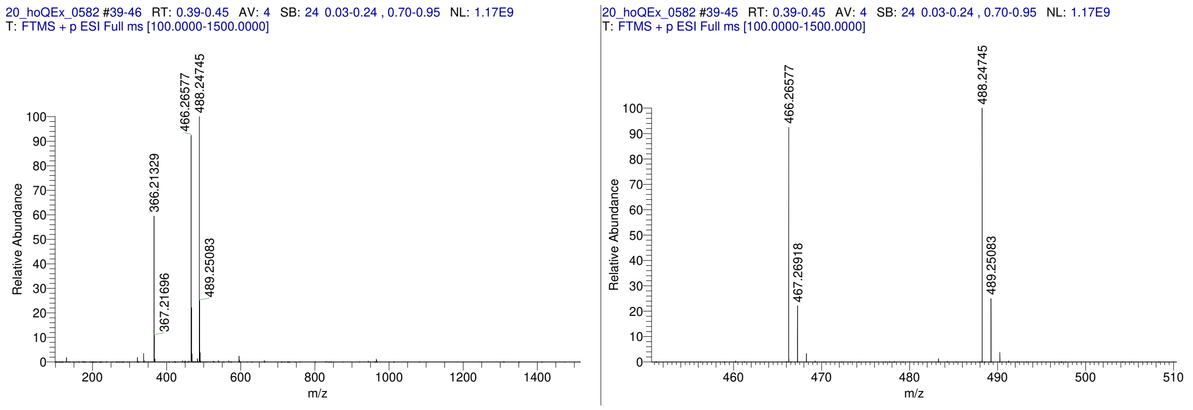

Figure S28. HR-ESI-MS spectrum of compound **20**

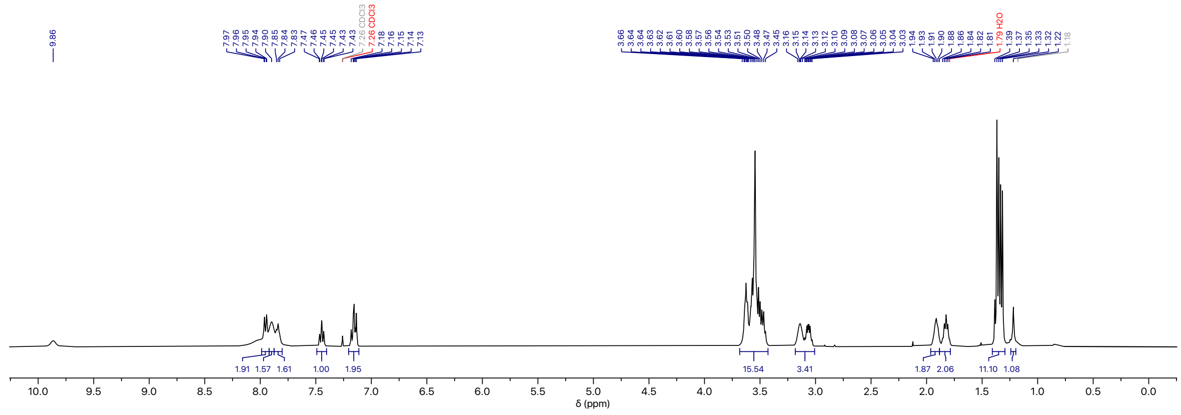

Figure S29. ^{1}H (400 MHz, $CDCl_3$, 298 K) NMR spectrum of compound **21**

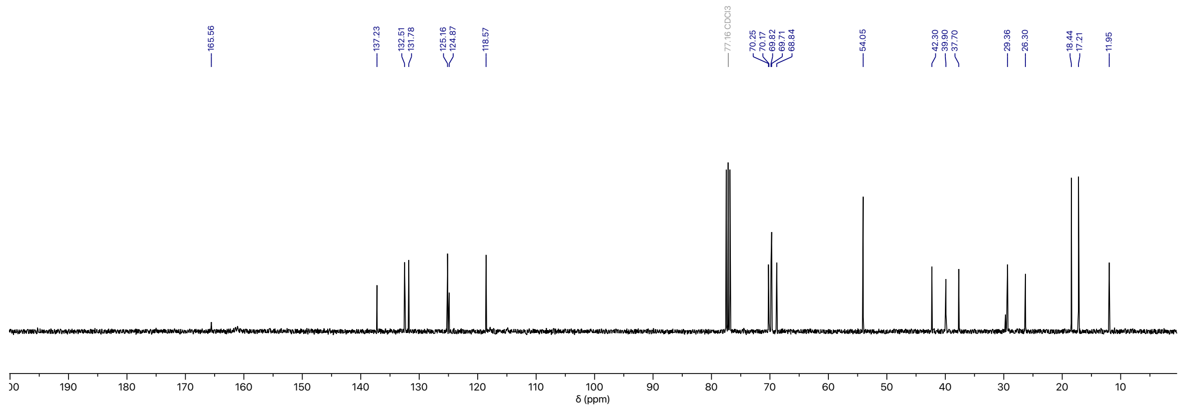

Figure S30. ^{13}C (101 MHz, $CDCl_3$, 298 K) NMR spectrum of compound **21**

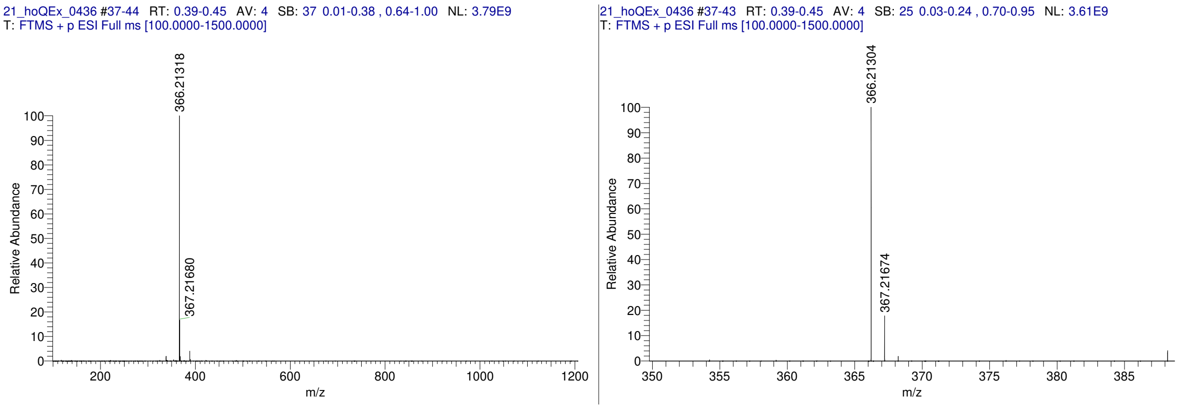

Figure S31. HR-ESI-MS spectrum of compound **21**

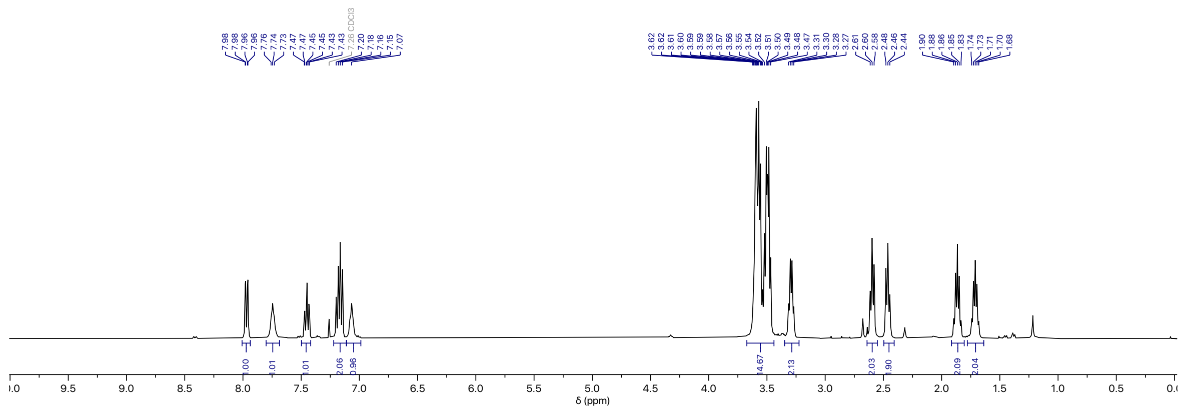

Figure S32. ^{1}H (400 MHz, $CDCl_3$, 298 K) NMR spectrum of compound **22**

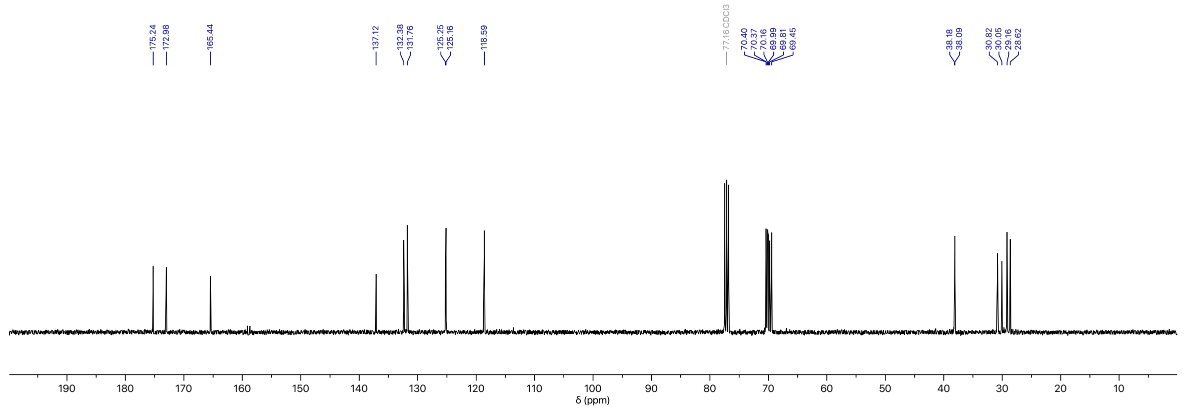

Figure S33. ^{13}C (101 MHz, $CDCl_3$, 298 K) NMR spectrum of compound **22**

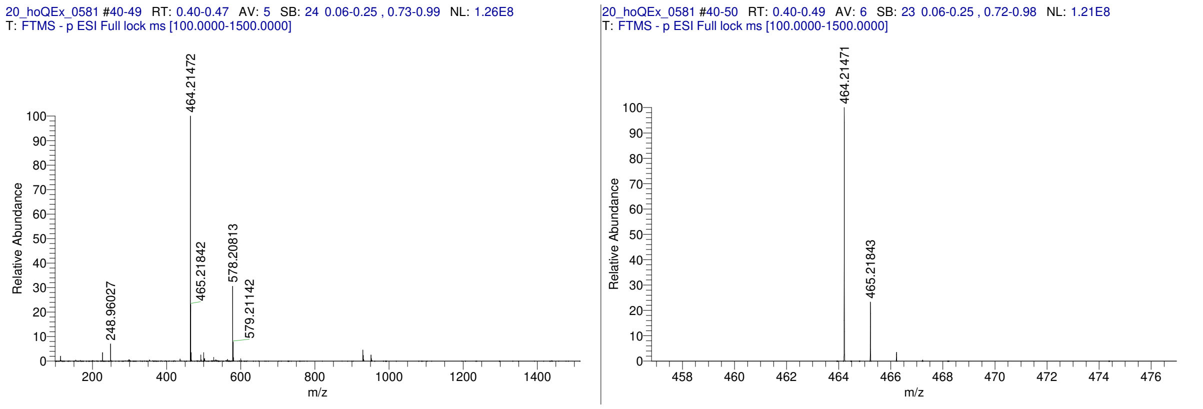

Figure S34. HR-ESI-MS spectrum of compound **22**

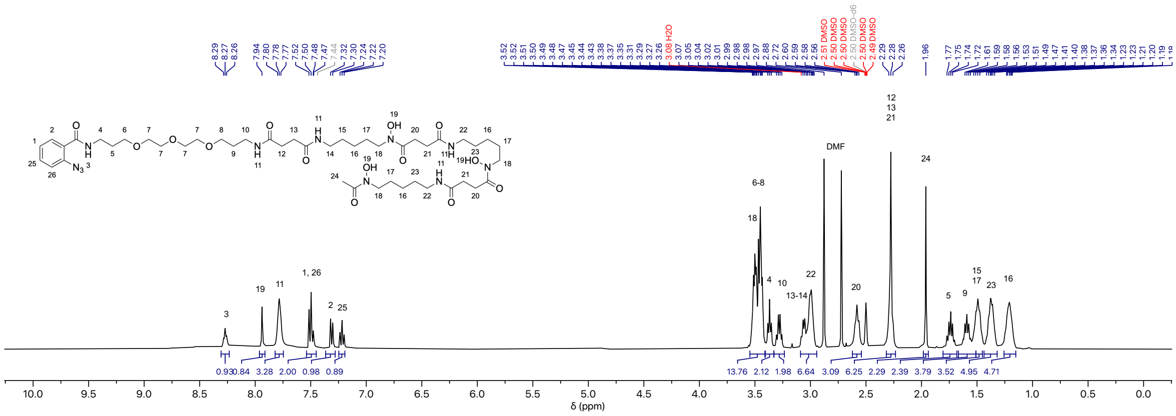

Figure S35. ^{1}H (400 MHz, DMSO-d^{6}, 298 K) NMR spectrum of compound **3**

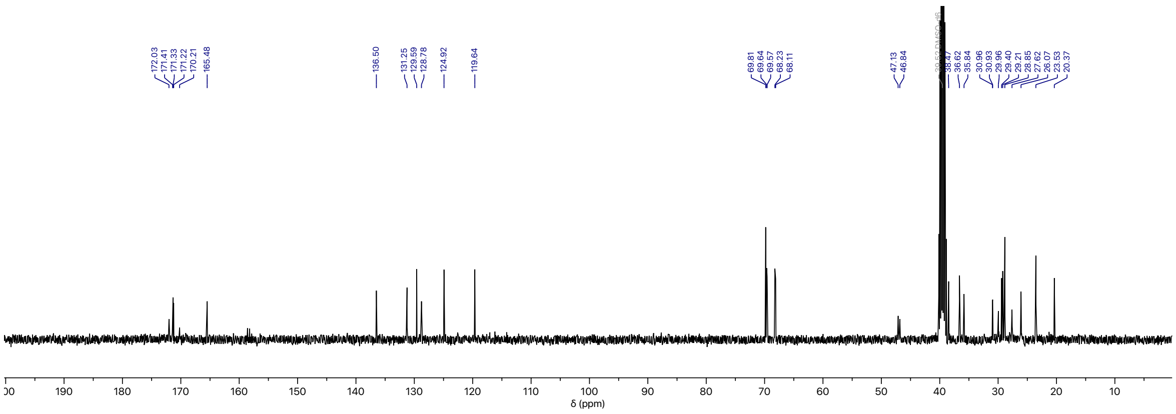

Figure S36. ^{13}C (101 MHz, DMSO-d^{6}, 298 K) NMR spectrum of compound **3**

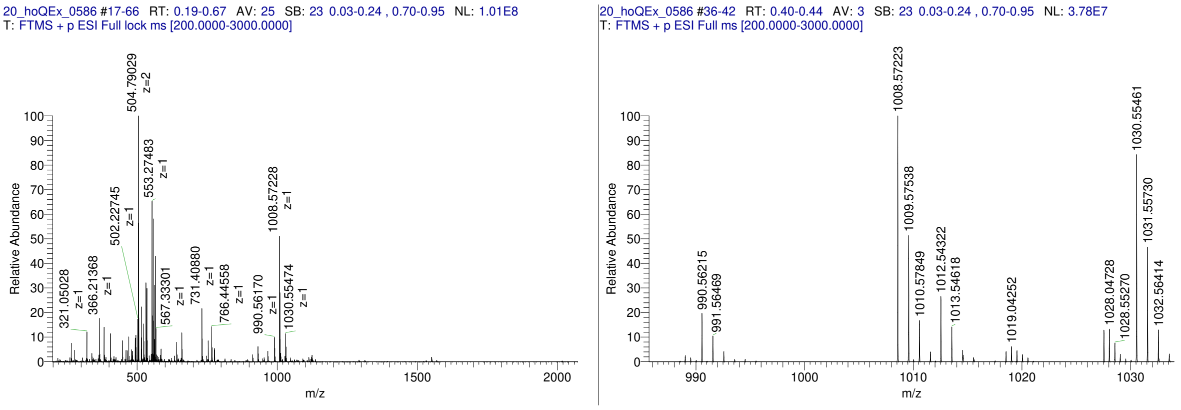

Figure S37. HR-ESI-MS spectrum of compound **3**

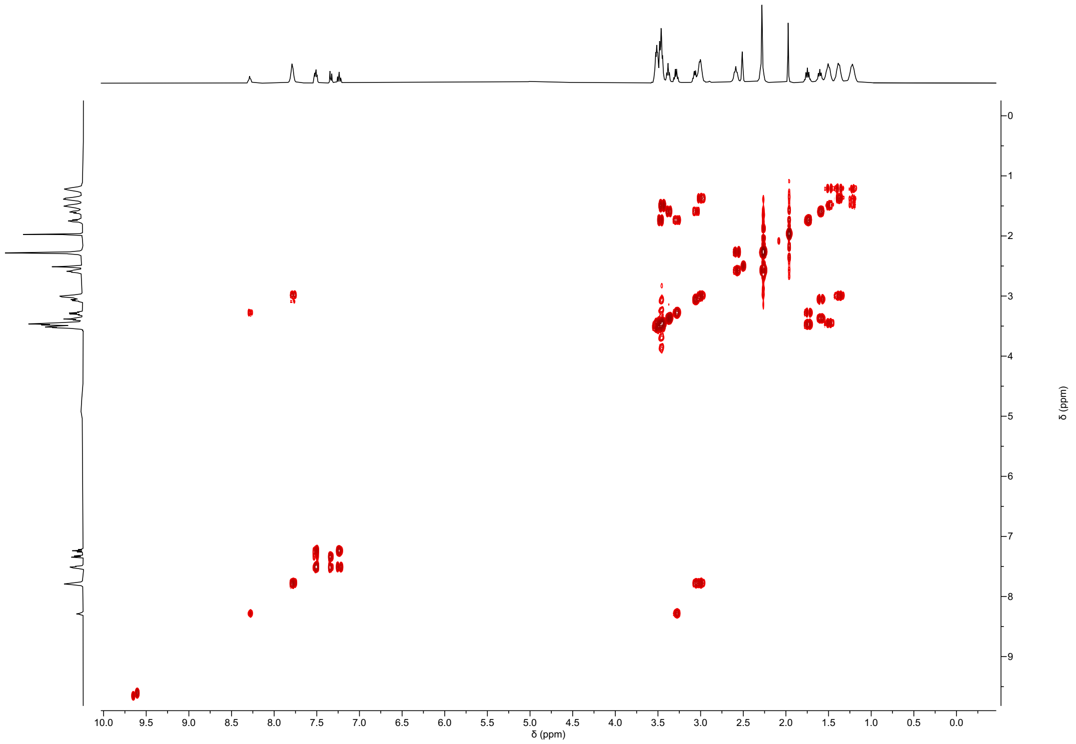

Figure S38. ^{1}H-^{1}H (COSY, DMSO-d^{6}, 298 K) 2D NMR spectrum of compound **3**

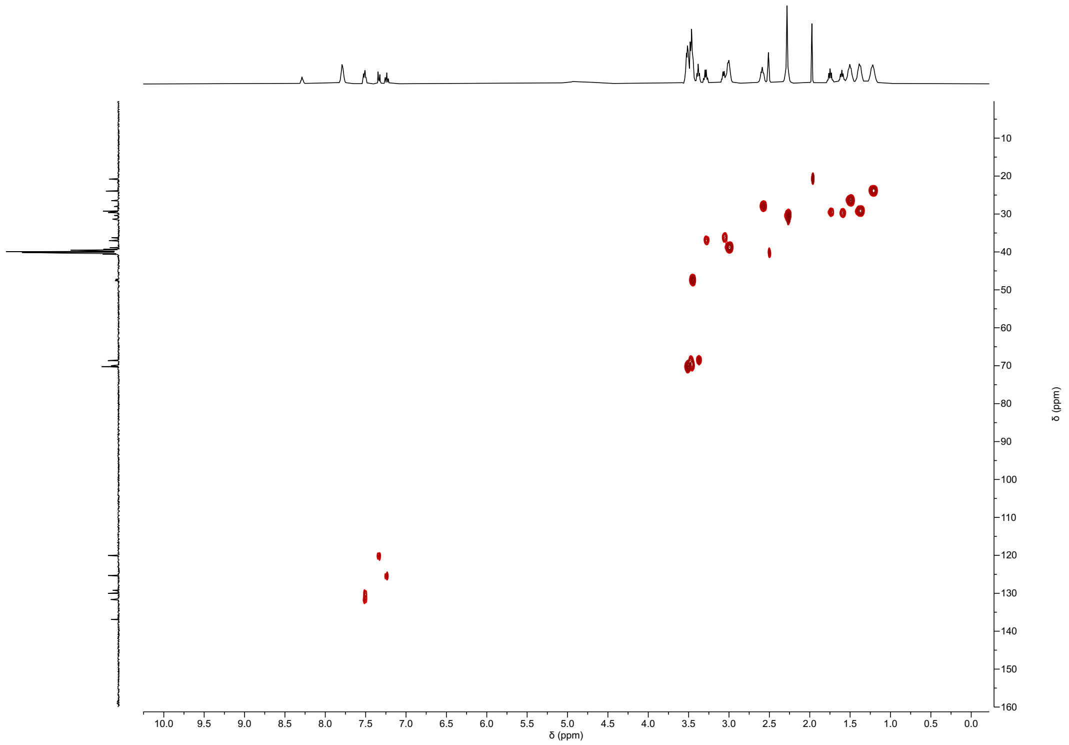

Figure S39. ^{1}H-^{13}C (COSY, DMSO-d^{6}, 298 K) 2D NMR spectrum of compound **3**

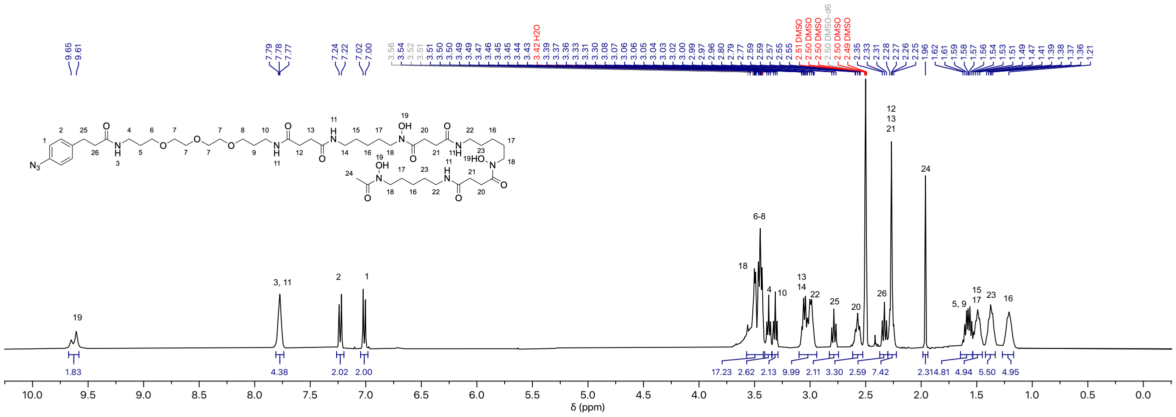

Figure S40. ^{1}H (500 MHz, DMSO-d^{6}, 298 K) NMR spectrum of compound **4**

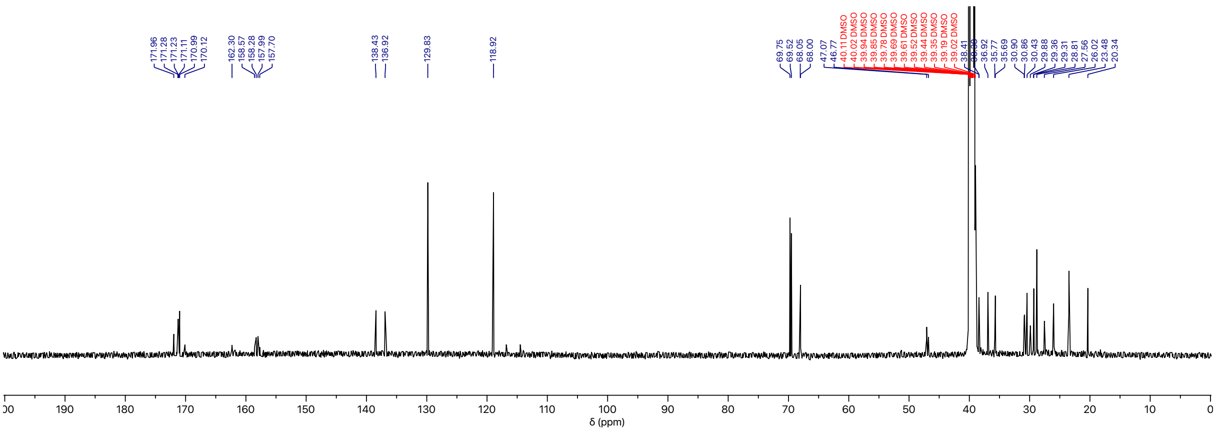

Figure S41. ^{13}C (126 MHz, DMSO-d^{6}, 298 K) NMR spectrum of compound **4**

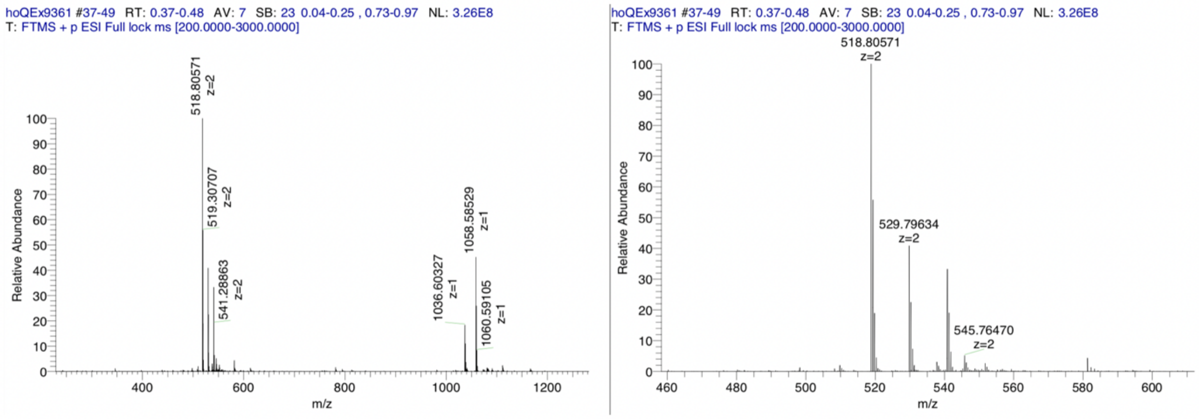

Figure S42. HR-ESI-MS spectrum of compound **4**

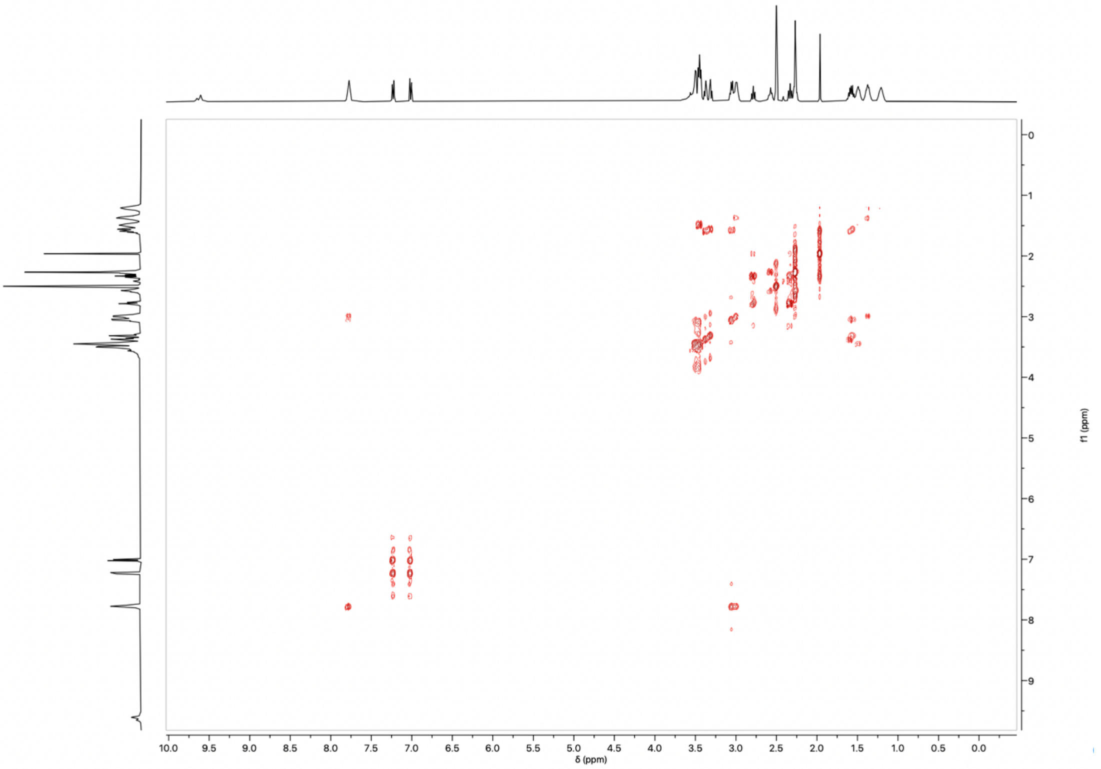

Figure S43. ^{1}H-^{1}H (COSY, DMSO-d^{6}, 298 K) 2D NMR spectrum of compound **4**

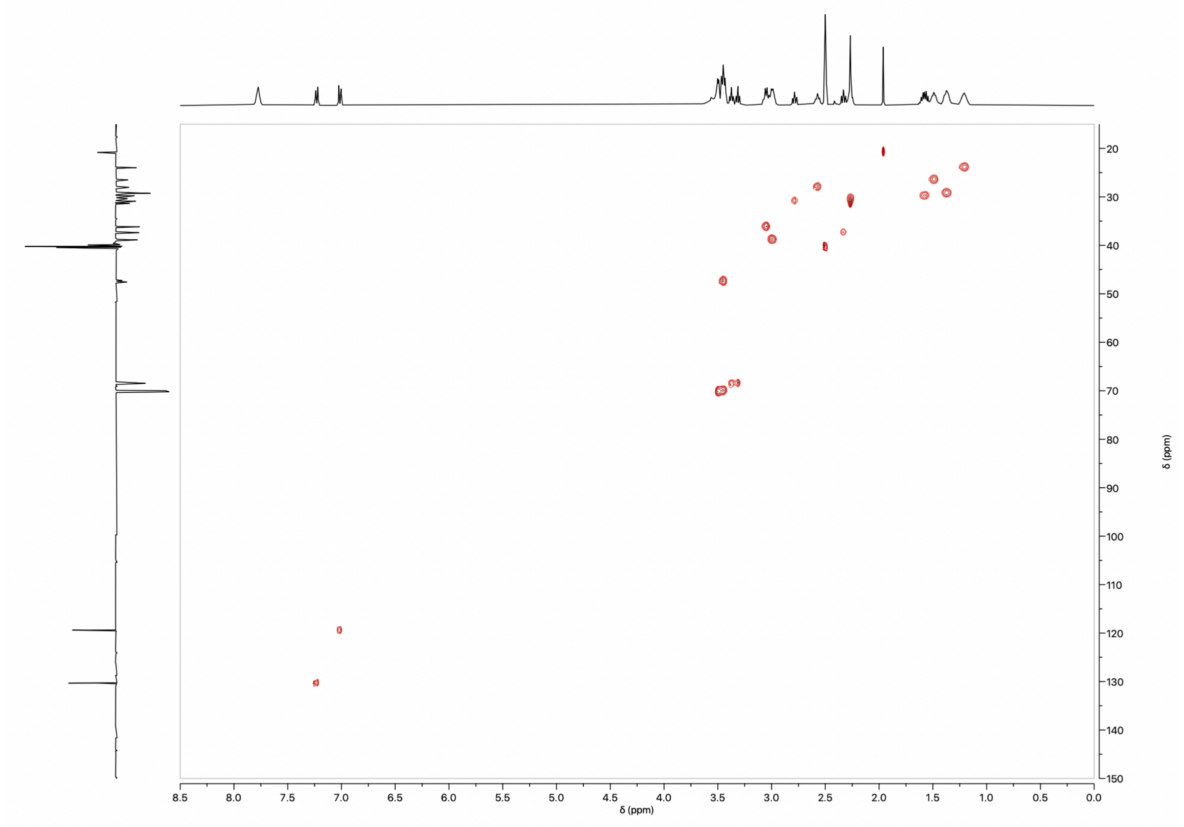

Figure S44. ^{1}H-^{13}C (HSQC, DMSO-d^{6}, 298 K) 2D NMR spectrum of compound **4**

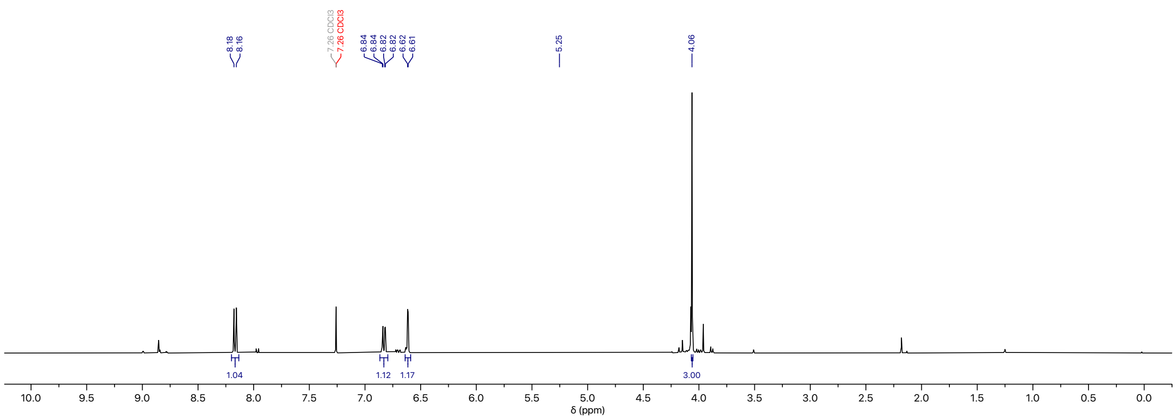

Figure S45. ^{1}H (400 MHz, $CDCl_3$, 298 K) NMR spectrum of compound **23**

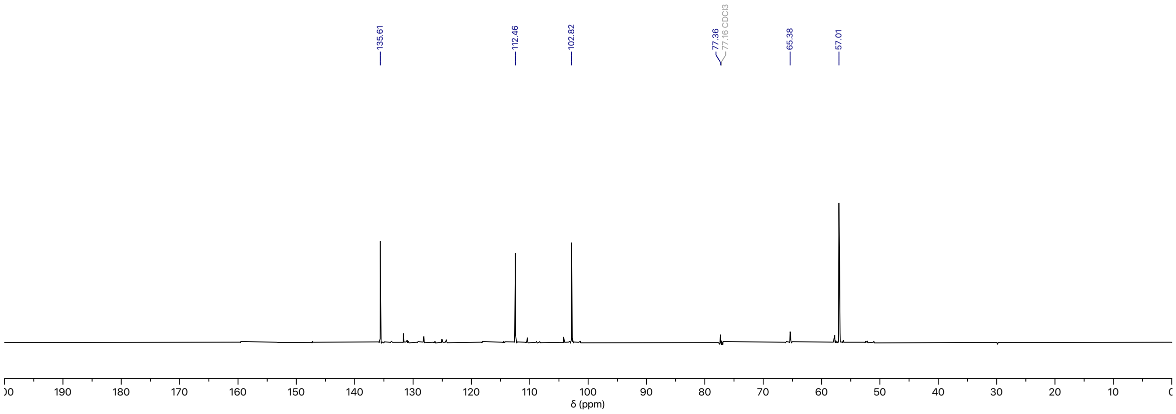

Figure S46. ^{13}C (101 MHz, $CDCl_3$, 298 K) NMR spectrum of compound **23**

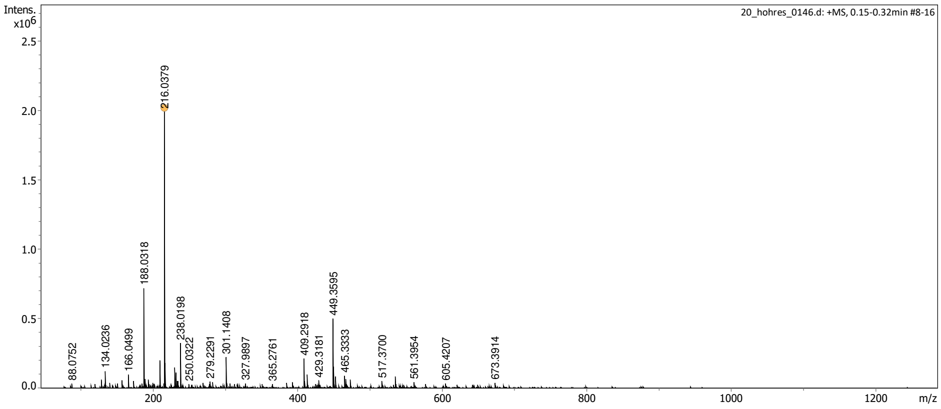

Figure S47. HR-ESI-MS spectrum of compound **23**

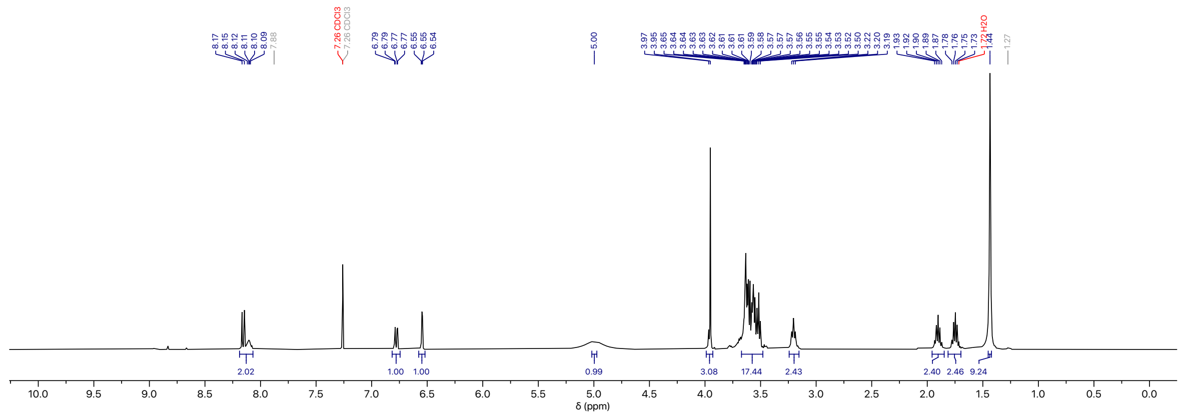

Figure S48. ^{1}H (400 MHz, $CDCl_3$, 298 K) NMR spectrum of compound **24**

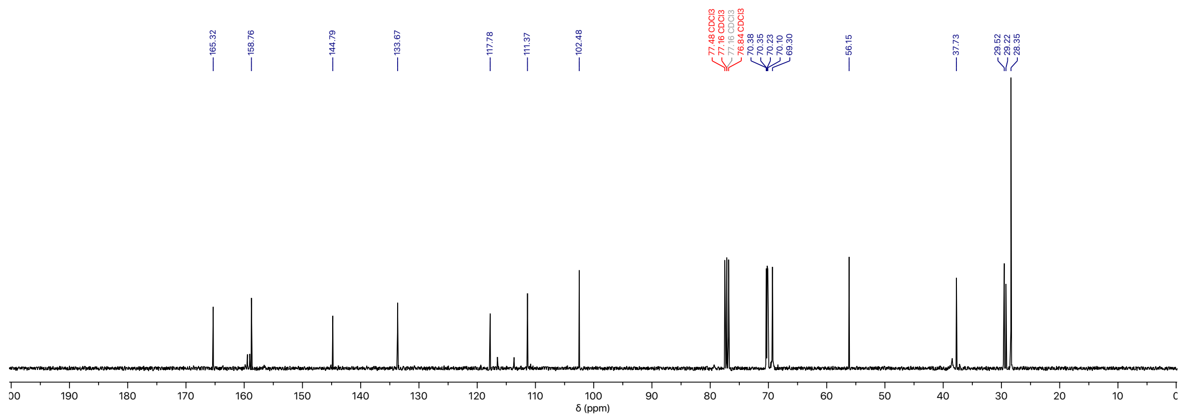

Figure S49. ^{13}C (101 MHz, $CDCl_3$, 298 K) NMR spectrum of compound **24**

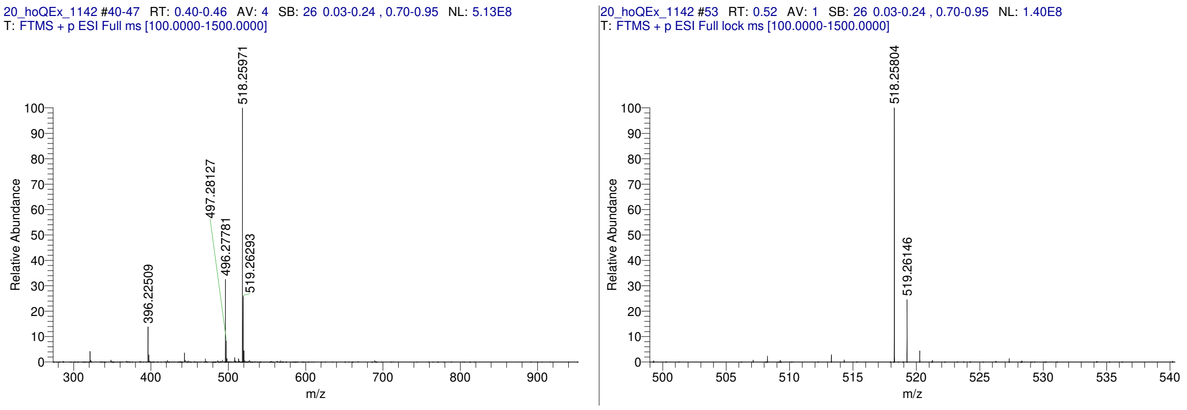

Figure S50. HR-ESI-MS spectrum of compound **24**

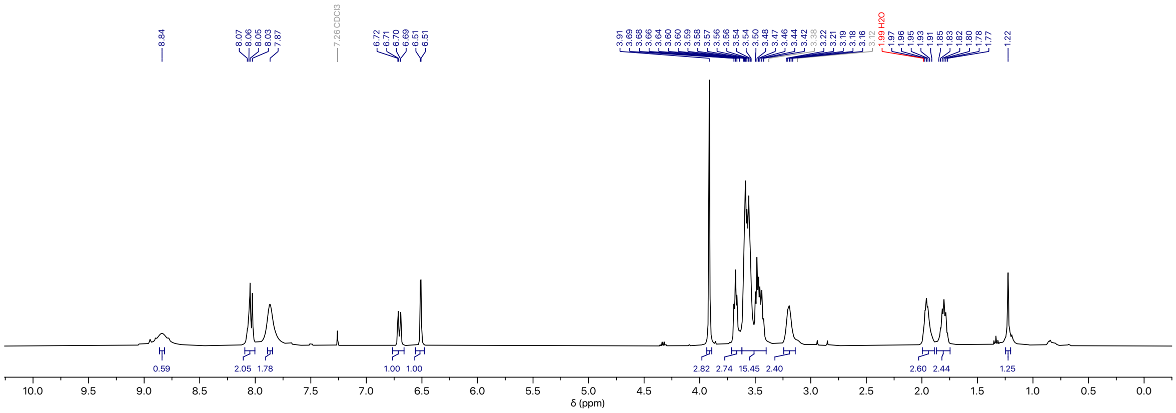

Figure S51. ^{1}H (400 MHz, $CDCl_3$, 298 K) NMR spectrum of compound **25**

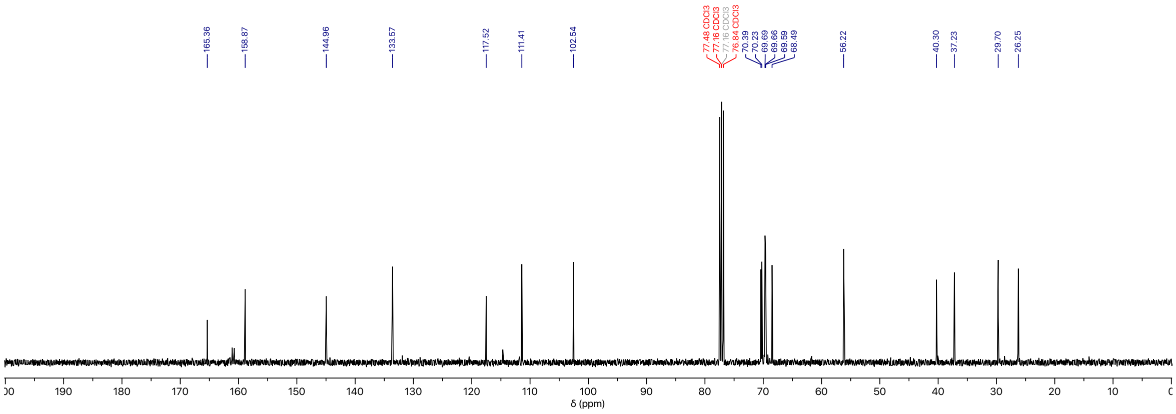

Figure S52. ^{13}C (101 MHz, $CDCl_3$, 298 K) NMR spectrum of compound **25**

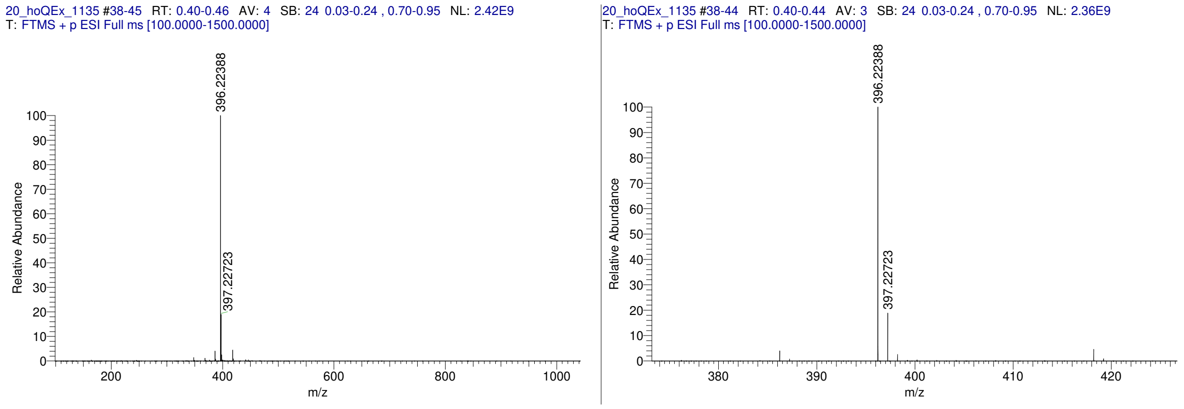

Figure S53. HR-ESI-MS spectrum of compound **25**

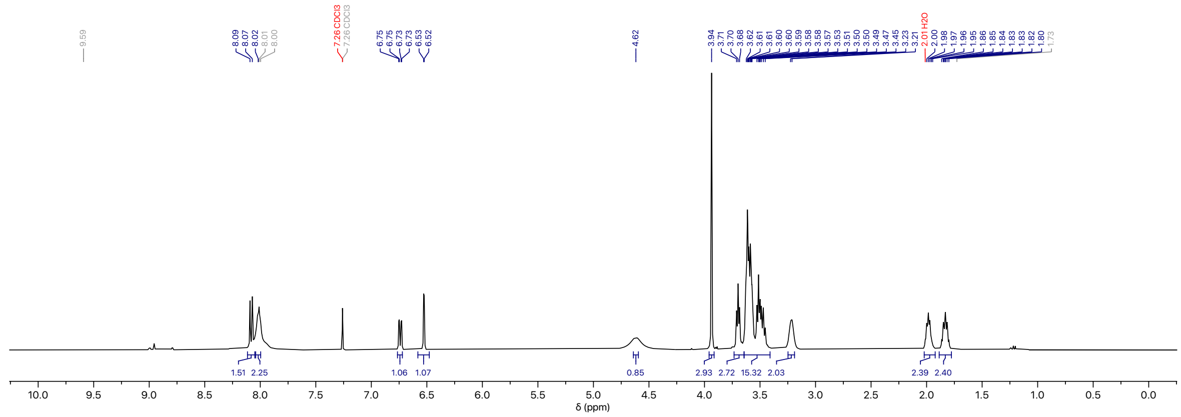

Figure S54. ^{1}H (400 MHz, $CDCl_3$, 298 K) NMR spectrum of compound **26**

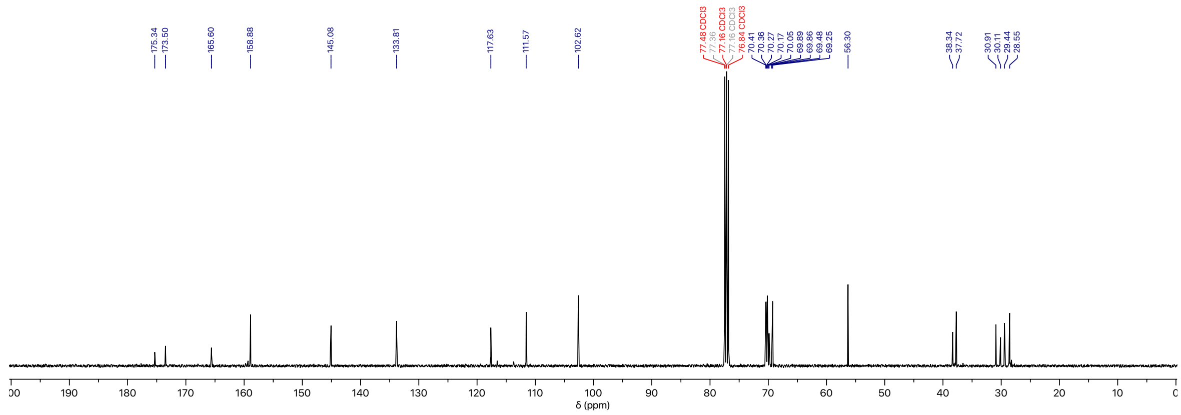

Figure S55. ^{13}C (101 MHz, $CDCl_3$, 298 K) NMR spectrum of compound **26**

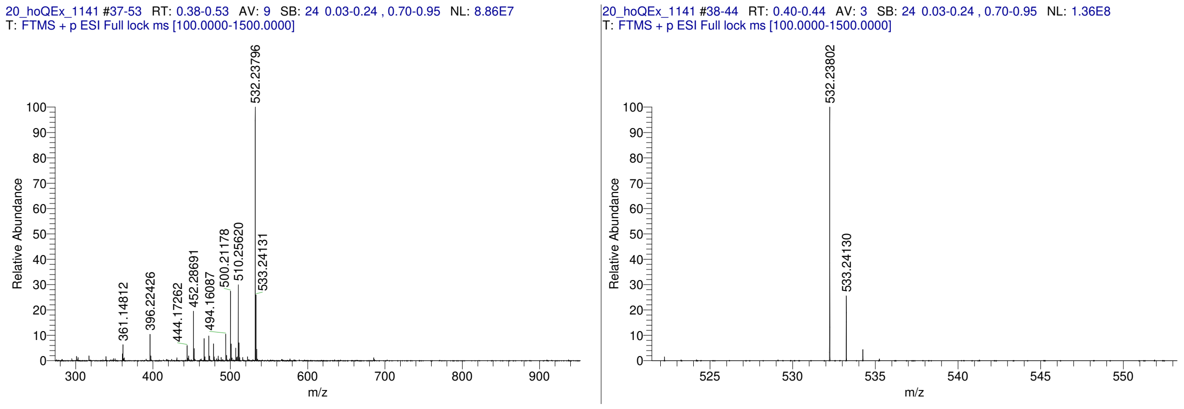

Figure S56. HR-ESI-MS spectrum of compound **26**

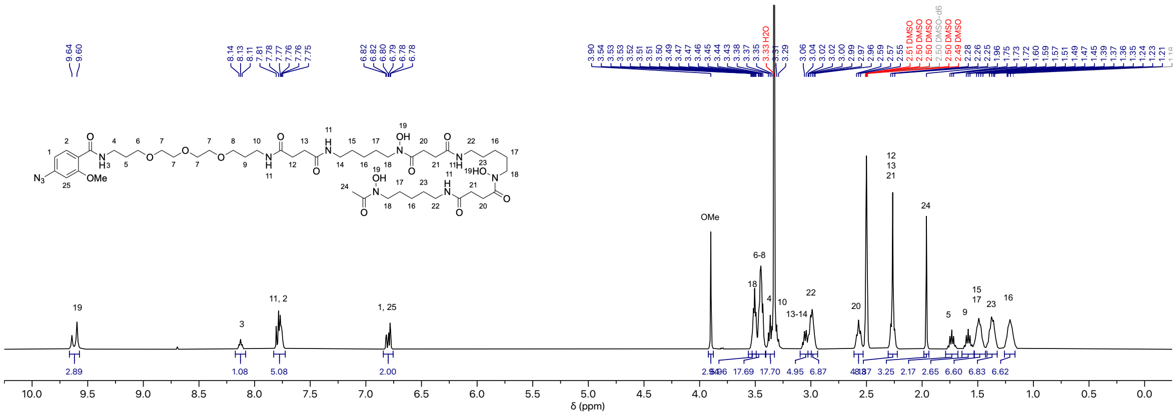

Figure S57. ^{1}H (400 MHz, DMSO-d^6, 298 K) NMR spectrum of compound **5**

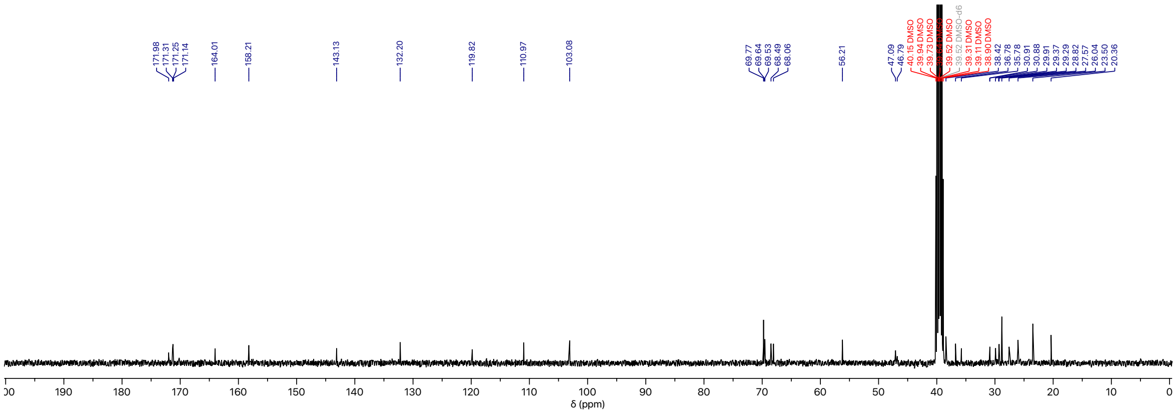

Figure S58. ^{13}C (126 MHz, DMSO-d$^6_3$, 298 K) NMR spectrum of compound **5**

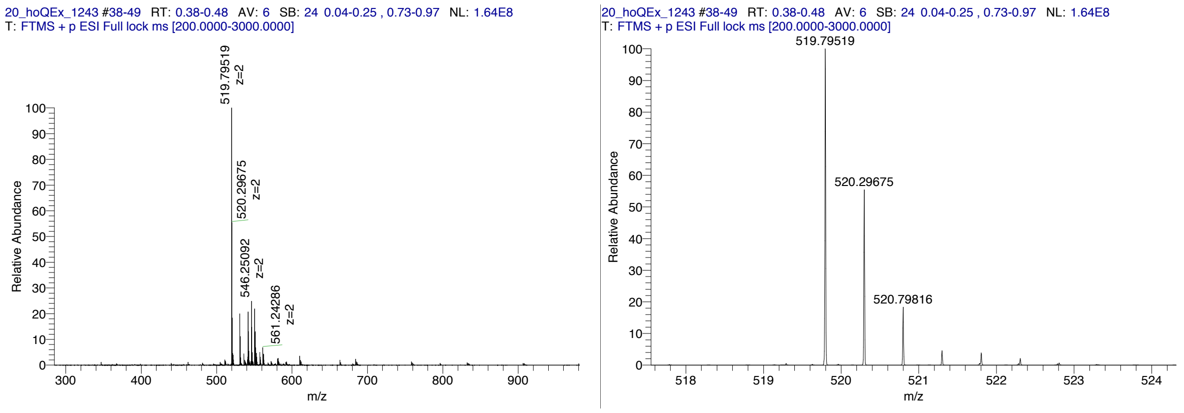

Figure S59. HR-ESI-MS spectrum of compound **5**

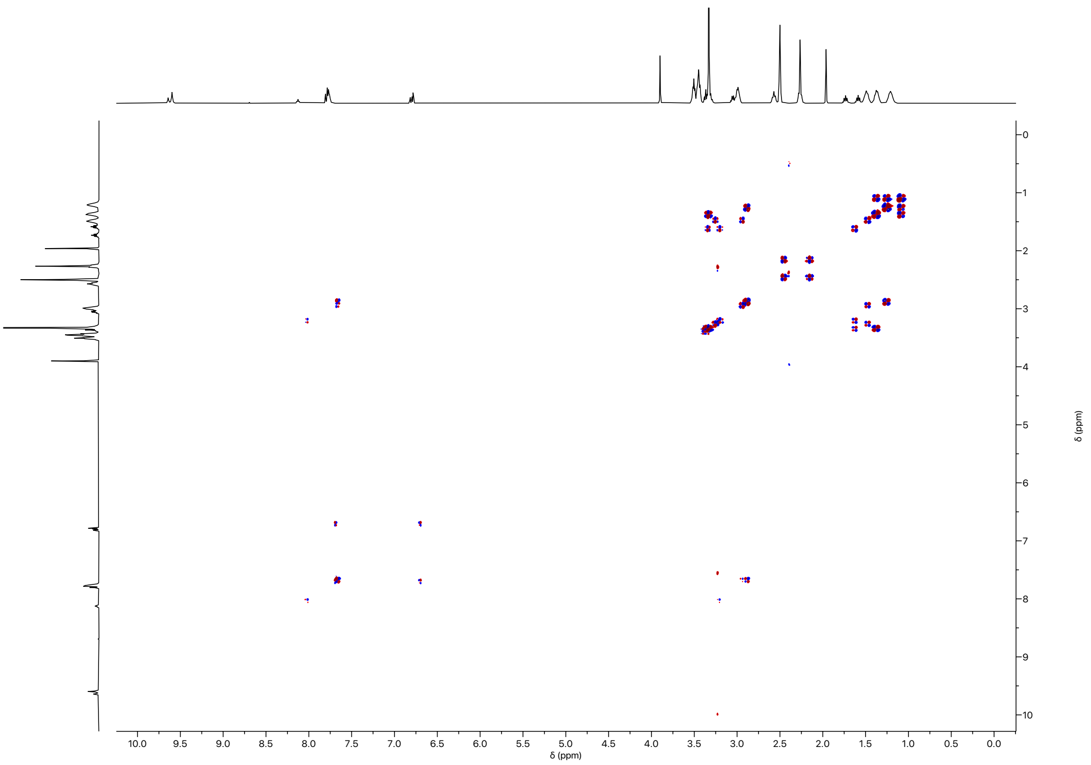

Figure S60. ^{1}H-^{1}H (COSY, DMSO-d^{6}, 298 K) 2D NMR spectrum of compound **5**

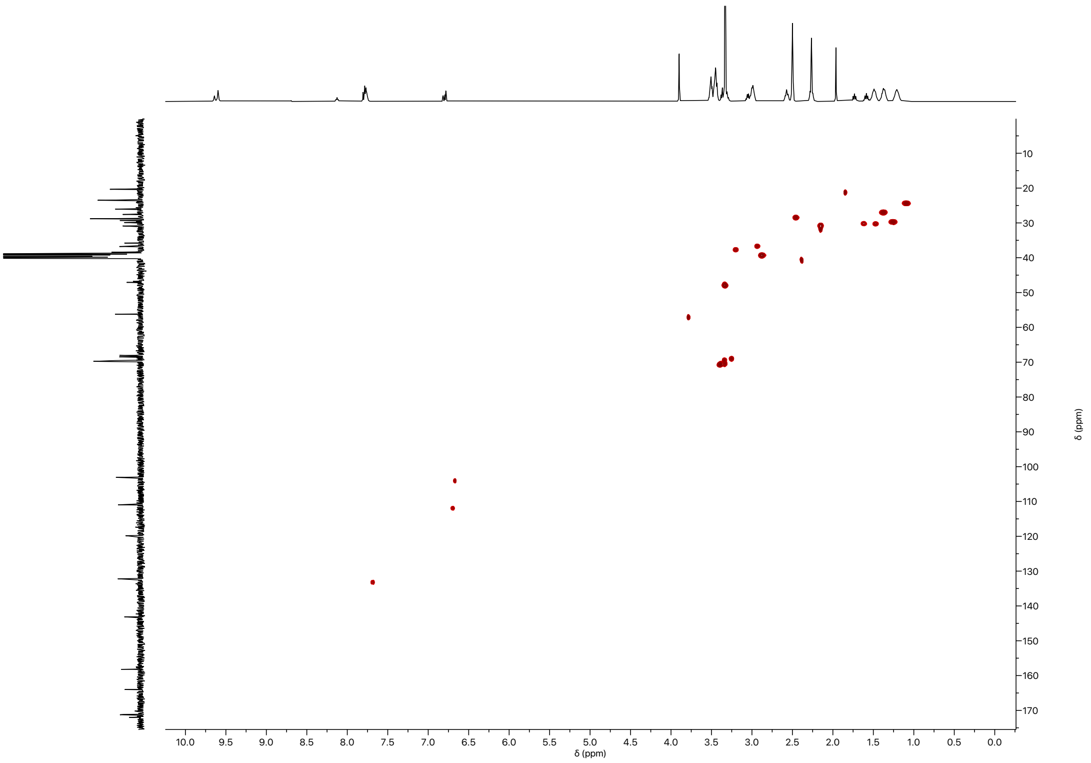

Figure S61. ^{1}H-^{13}C (HSQC, DMSO-d^{6}, 298 K) 2D NMR spectrum of compound **5**

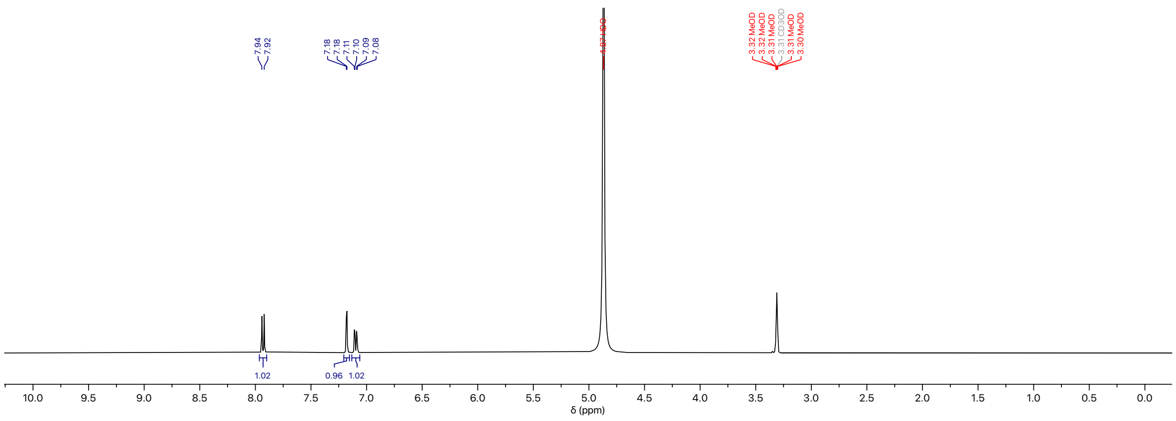

Figure S62. ^{1}H (400 MHz, MeOD-d^{4}, 298 K) NMR spectrum of compound **27**

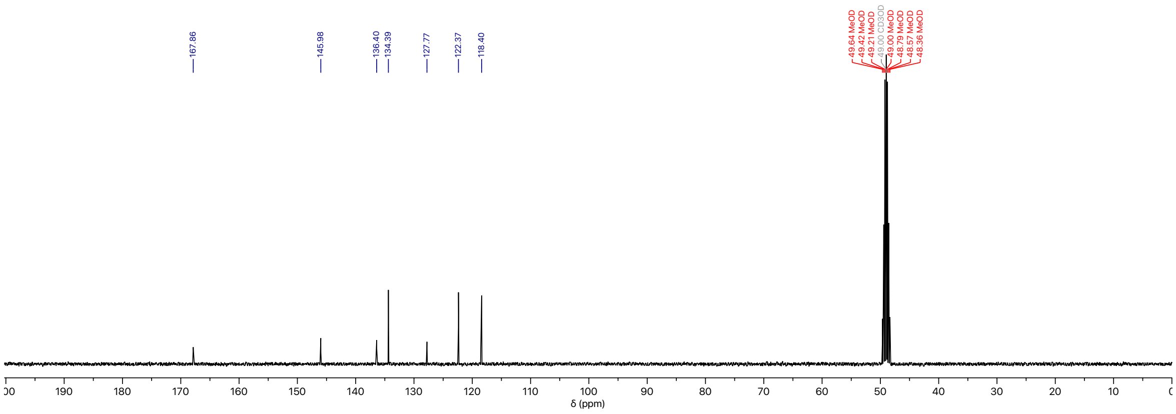

Figure S63. ^{13}C (101 MHz, MeOD-d^{4}, 298 K) NMR spectrum of compound **27**

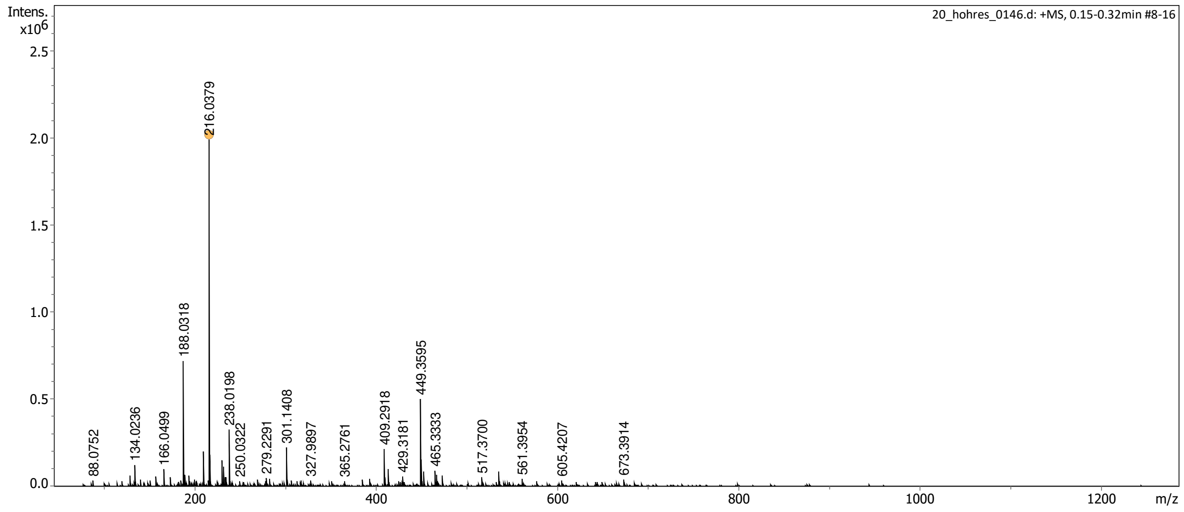

Figure S64. HR-ESI-MS spectrum of compound **27**

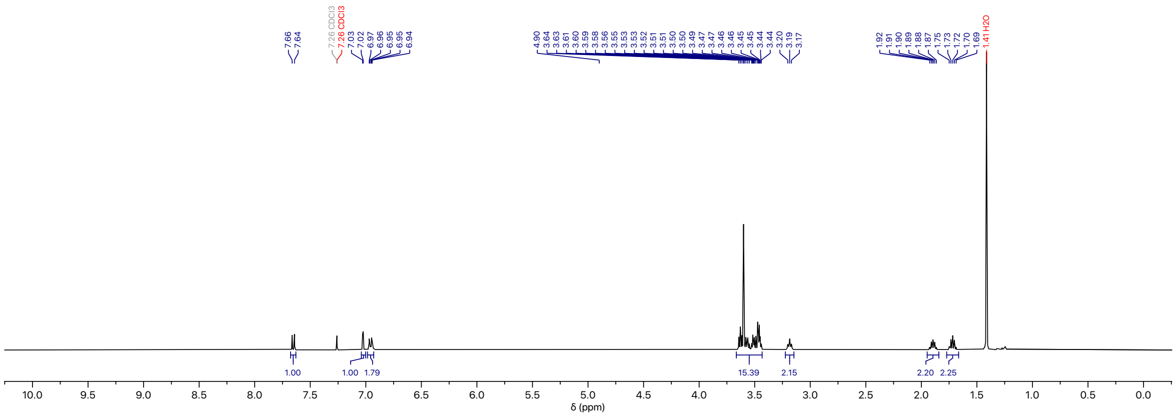

Figure S65. ^{1}H (400 MHz, $CDCl_3$, 298 K) NMR spectrum of compound **28**

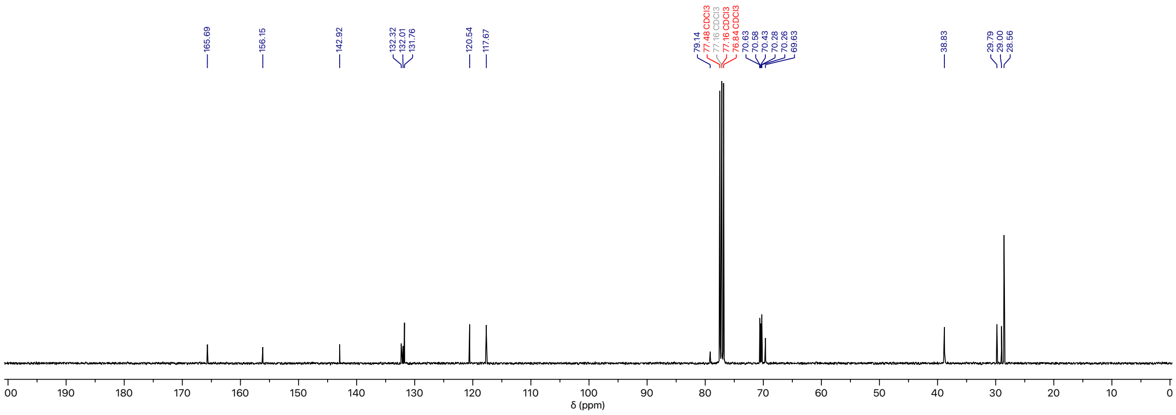

Figure S66. ^{13}C (101 MHz, $CDCl_3$, 298 K) NMR spectrum of compound **28**

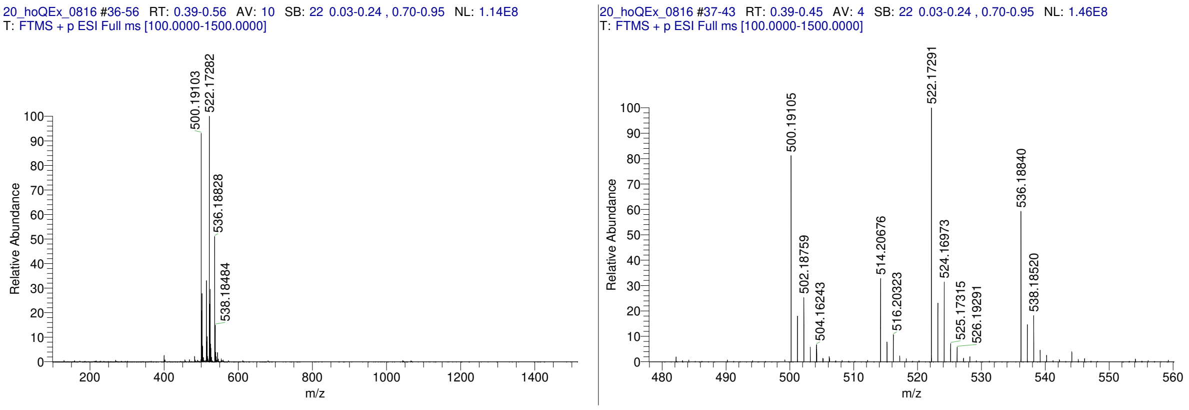

Figure S67. HR-ESI-MS spectrum of compound **28**

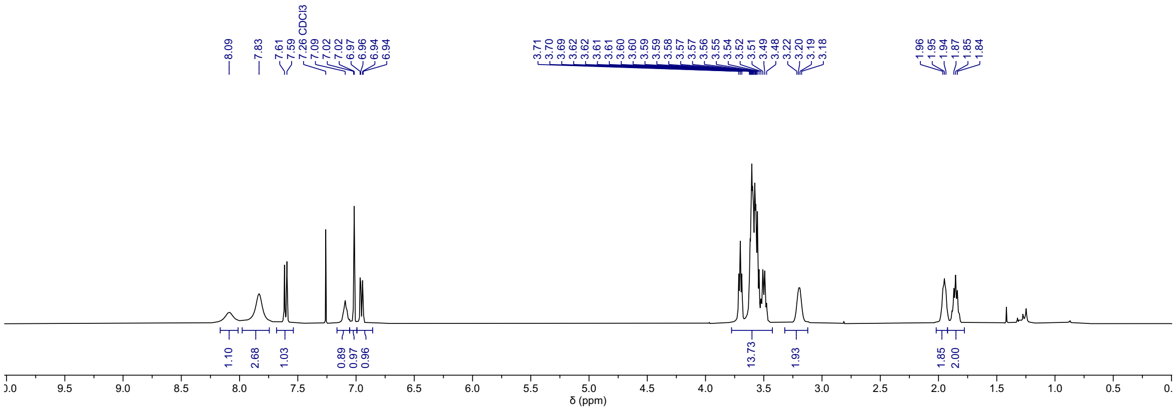

Figure S68. ^{1}H (400 MHz, $CDCl_3$, 298 K) NMR spectrum of compound **29**

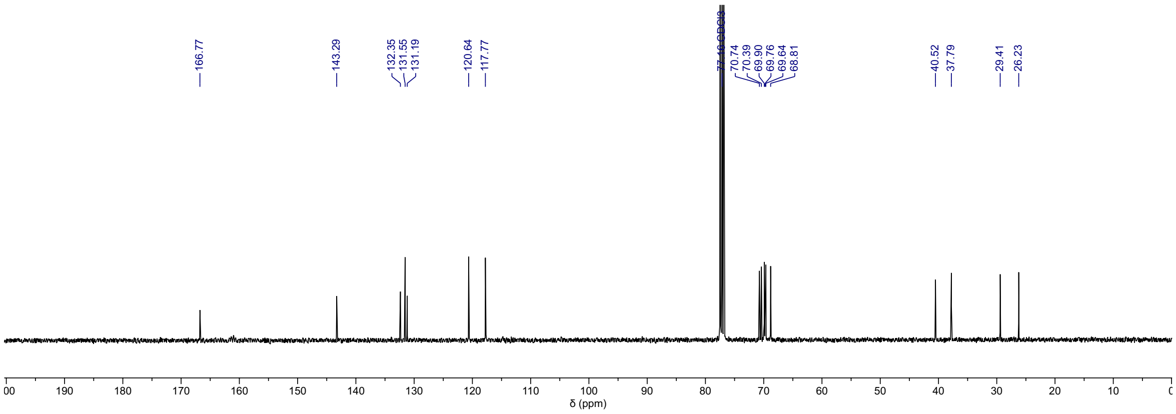

Figure S69. ^{13}C (101 MHz, $CDCl_3$, 298 K) NMR spectrum of compound **29**

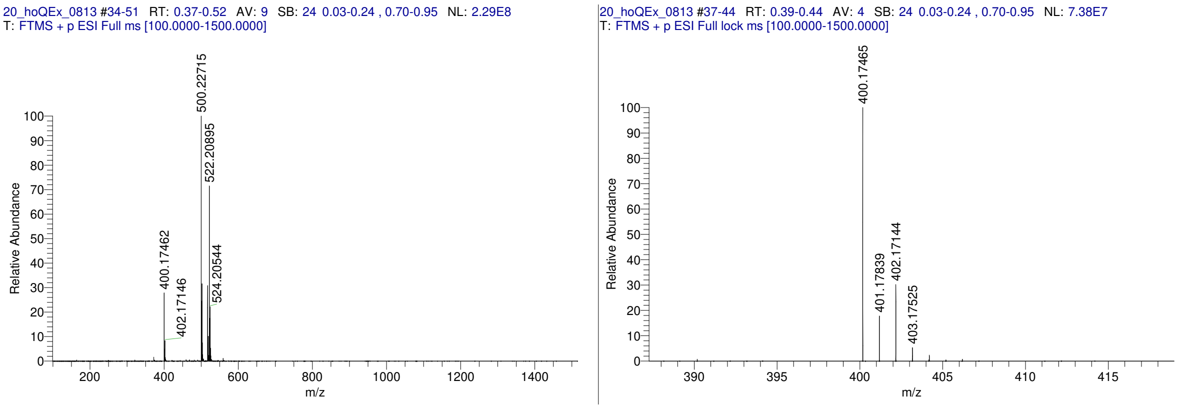

Figure S70. HR-ESI-MS spectrum of compound **29**

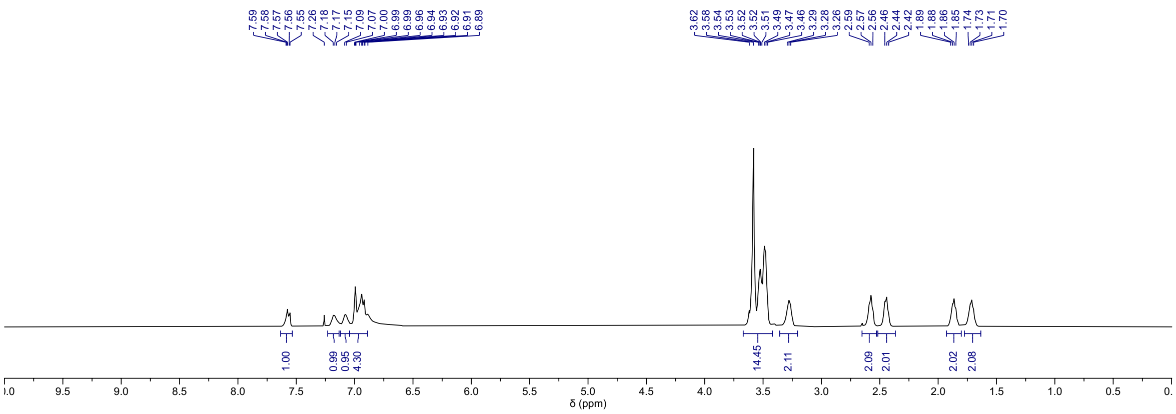

Figure S71. ^{1}H (400 MHz, $CDCl_3$, 298 K) NMR spectrum of compound **30**

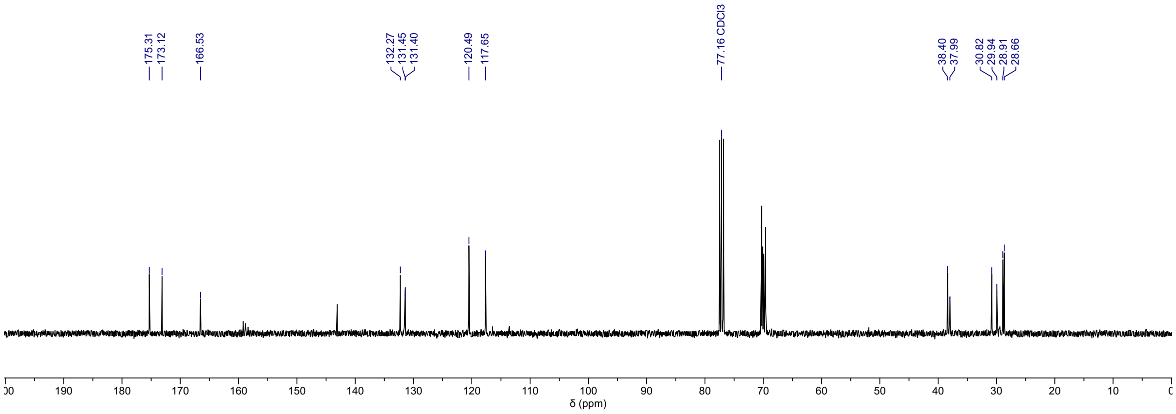

Figure S72. ^{13}C (101 MHz, $CDCl_3$, 298 K) NMR spectrum of compound **30**

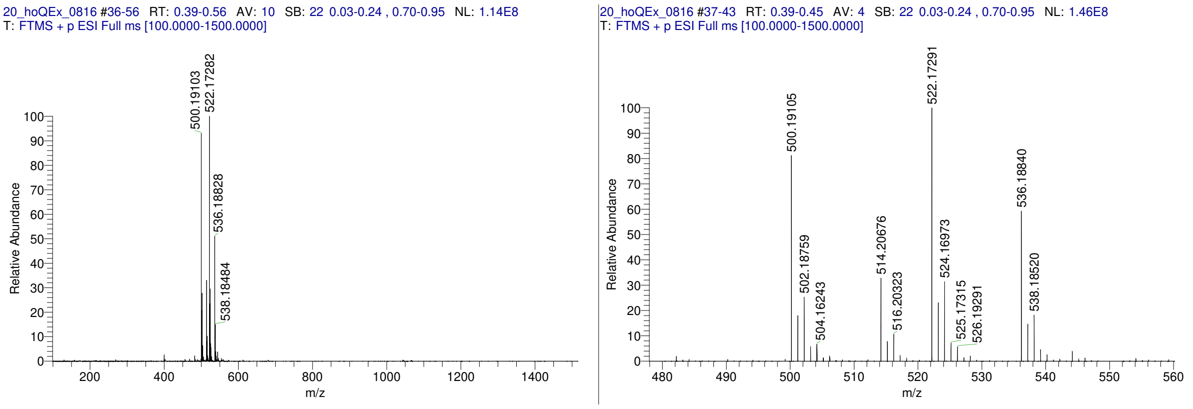

Figure S73. HR-ESI-MS spectrum of compound **30**

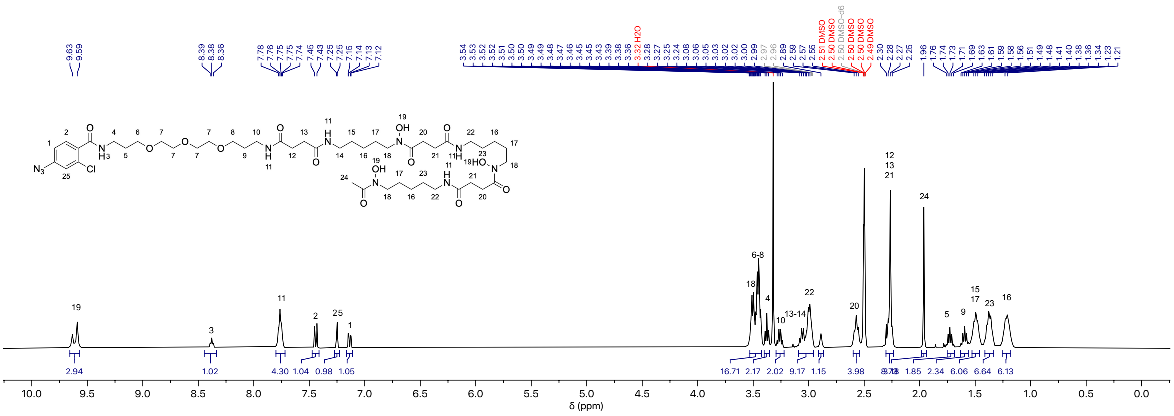

Figure S74. ^{1}H (400 MHz, DMSO-d^{6}, 298 K) NMR spectrum of compound **6**

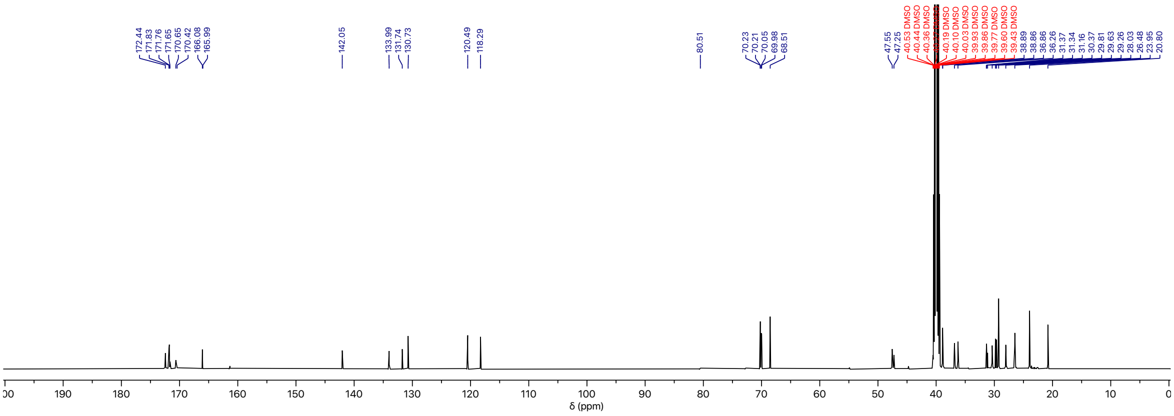

Figure S75. ^{13}C (101 MHz, DMSO-d^{6}, 298 K) NMR spectrum of compound **6**

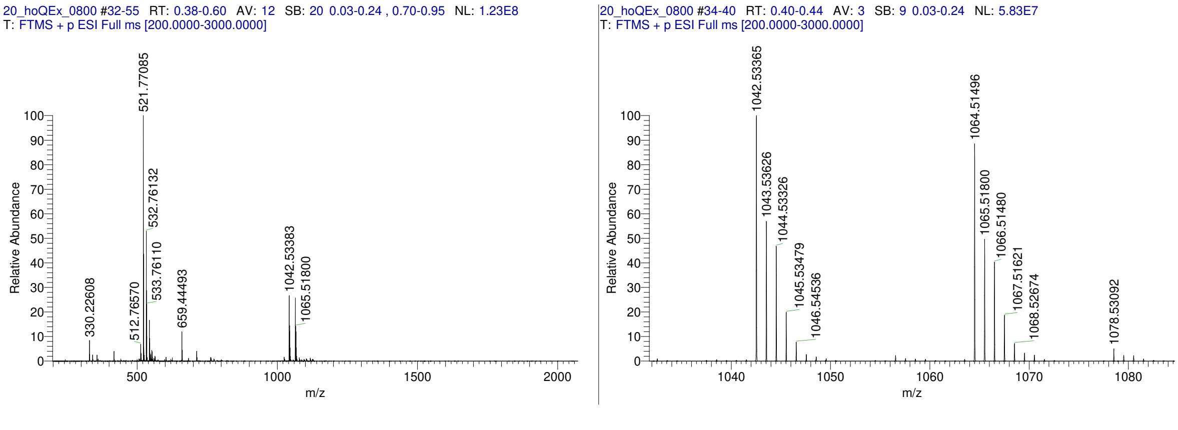

Figure S76. HR-ESI-MS spectrum of compound **6**

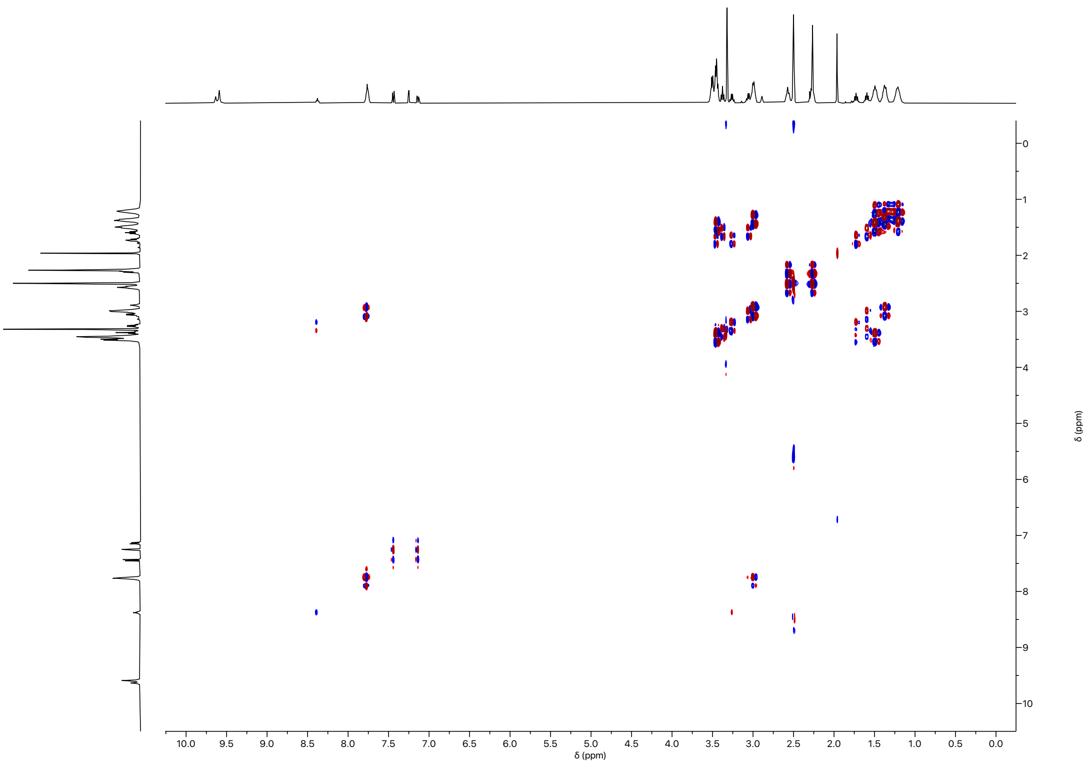

Figure S77. ^{1}H-^{1}H (COSY, DMSO-d^{6}, 298 K) 2D NMR spectrum of compound **6**

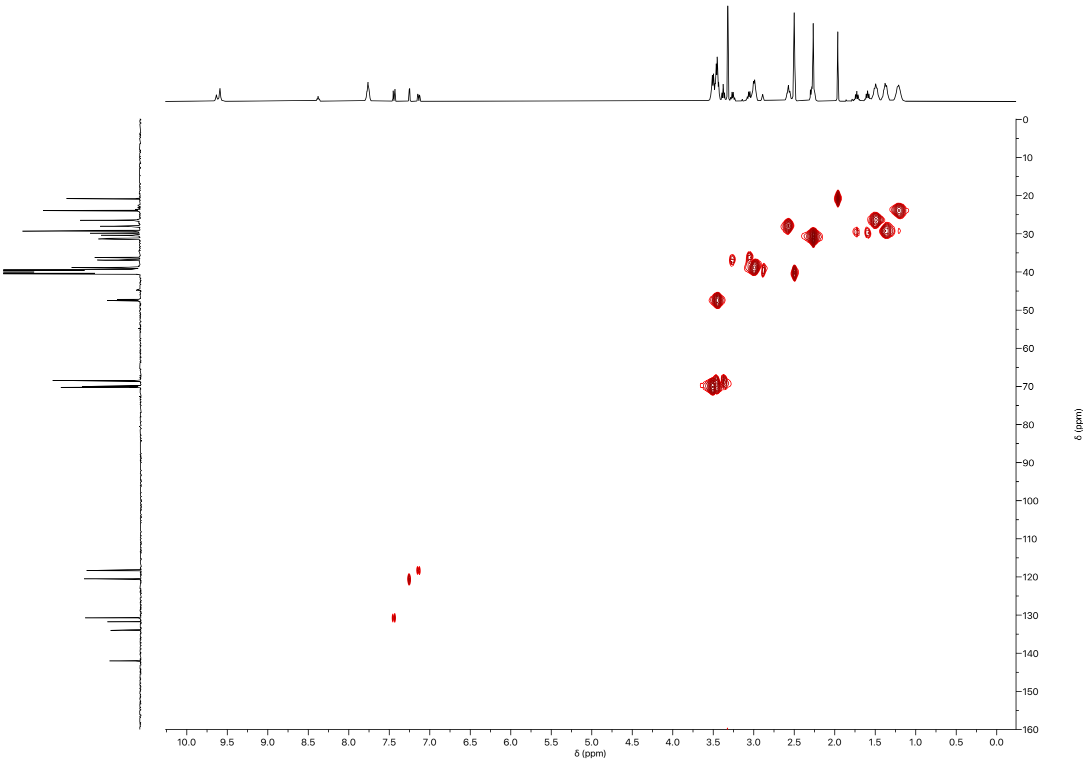

Figure S78. ^{1}H-^{13}C (HSQC, DMSO-d^{6}, 298 K) 2D NMR spectrum of compound **6**

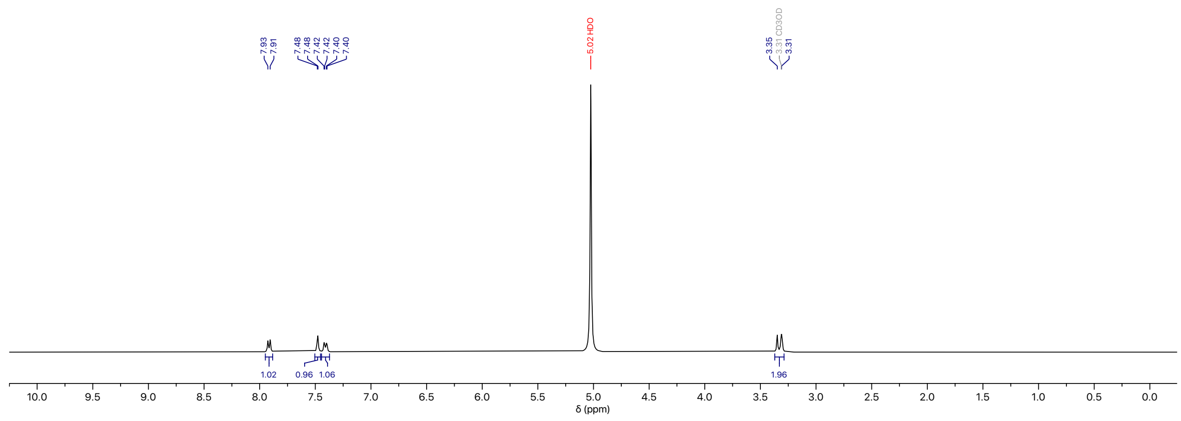

Figure S79. ^{1}H (400 MHz, MeOD-d^{4}, 298 K) NMR spectrum of compound **31**

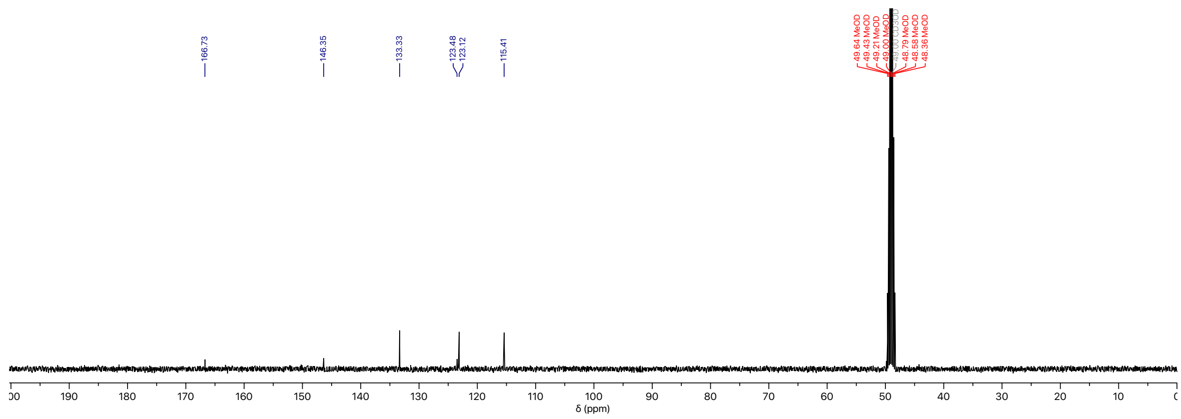

Figure S80. ^{13}C (101 MHz, MeOD-d^{4}, 298 K) NMR spectrum of compound **31**

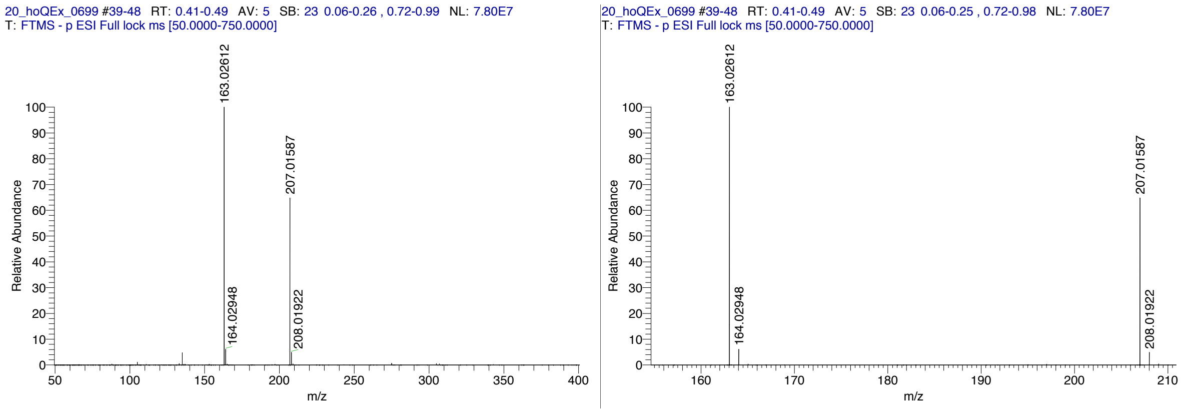

Figure S81. HR-ESI-MS spectrum of compound **31**

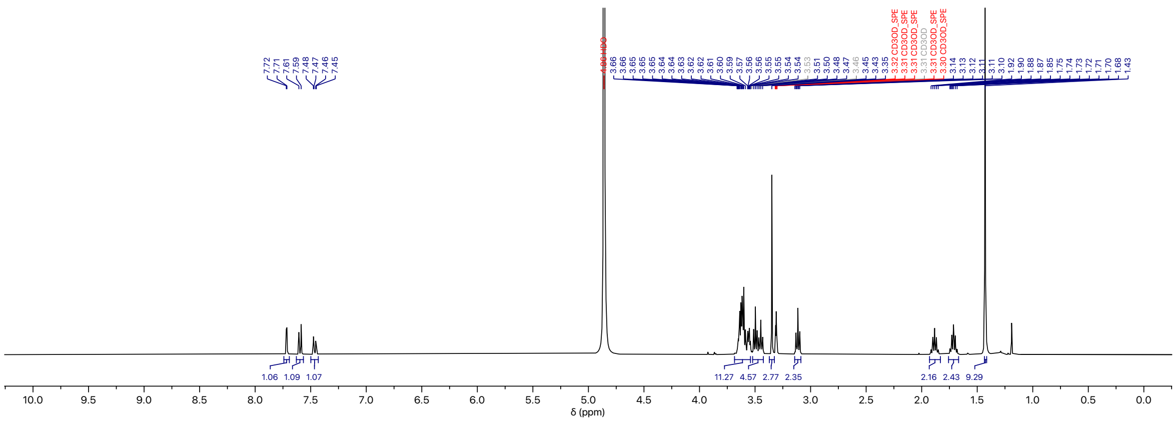

Figure S82. ^{1}H (400 MHz, $CDCl_3$, 298 K) NMR spectrum of compound **32**

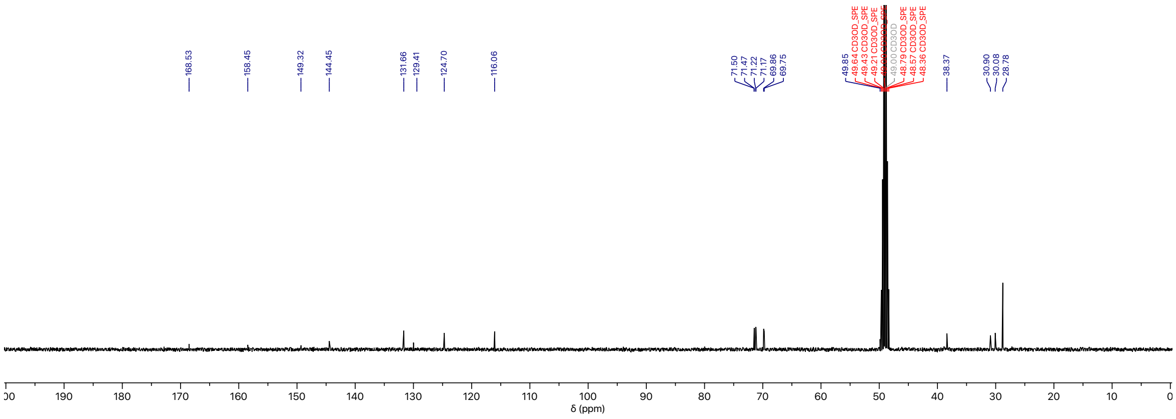

Figure S83. ^{13}C (101 MHz, $CDCl_3$, 298 K) NMR spectrum of compound **32**

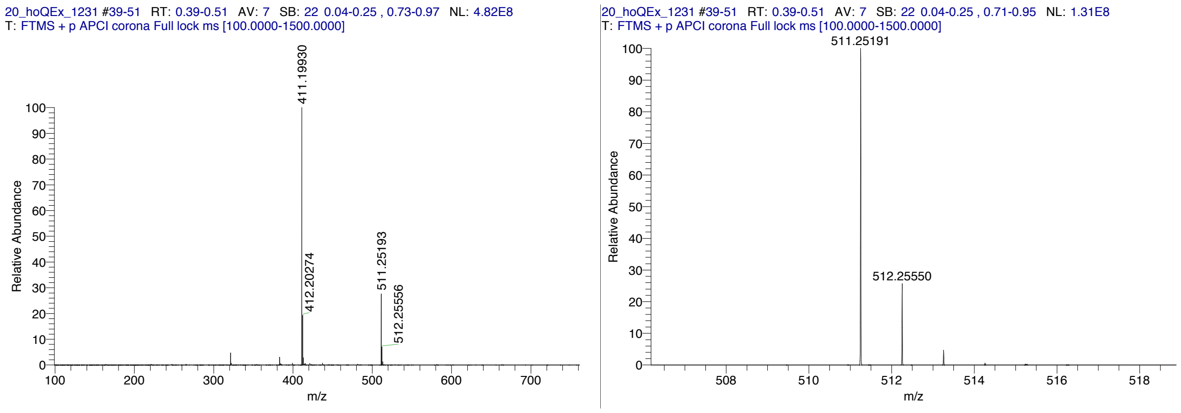

Figure S84. HR-ESI-MS spectrum of compound **32**

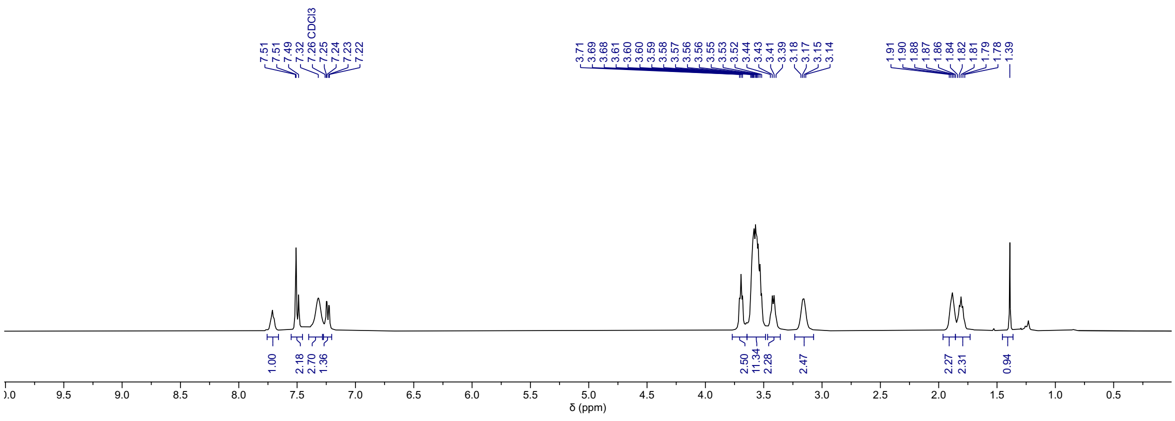

Figure S85. ^{1}H (400 MHz, $CDCl_3$, 298 K) NMR spectrum of compound **33**

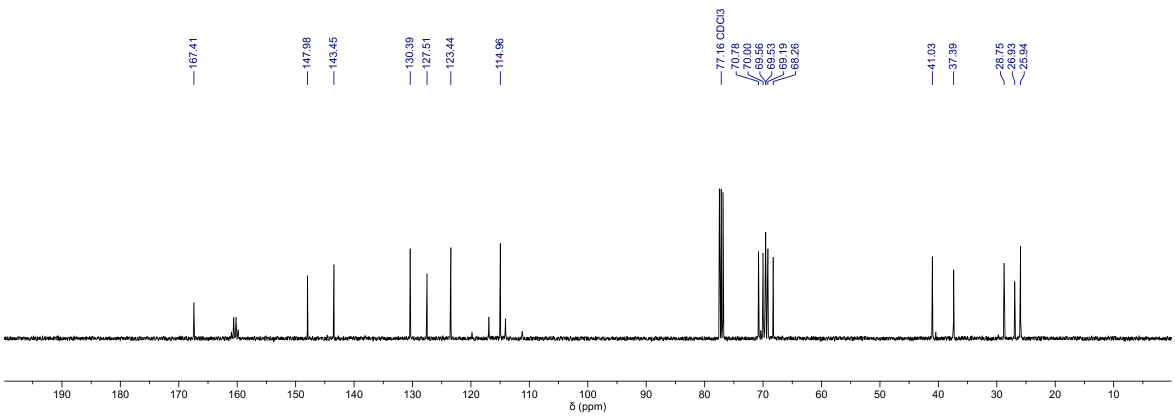

Figure S86. ^{13}C (101 MHz, $CDCl_3$, 298 K) NMR spectrum of compound **33**

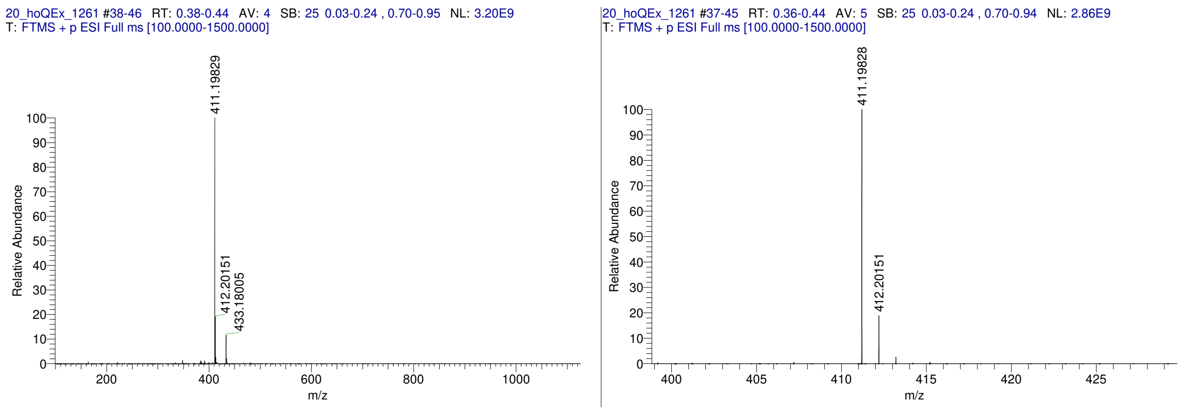

Figure S87. HR-ESI-MS spectrum of compound **33**

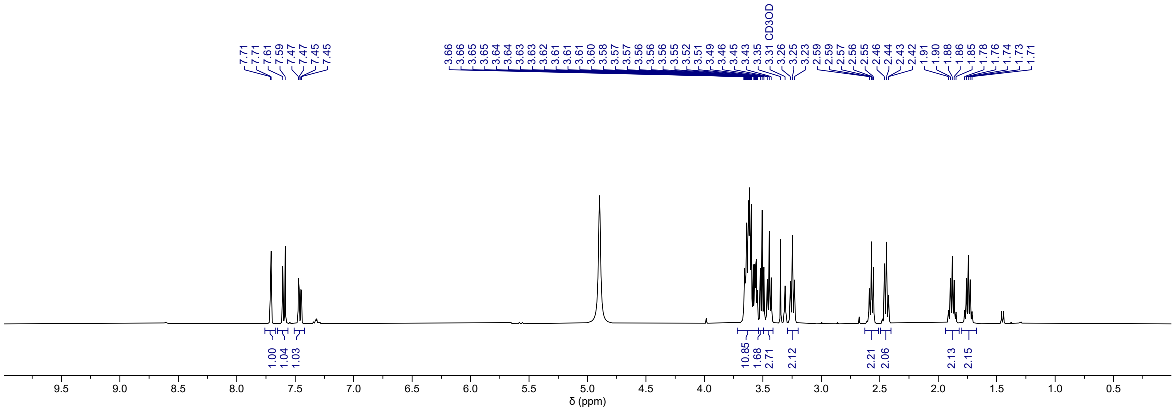

Figure S88. ^{1}H (400 MHz, MeOD-d^{4}, 298 K) NMR spectrum of compound **34**

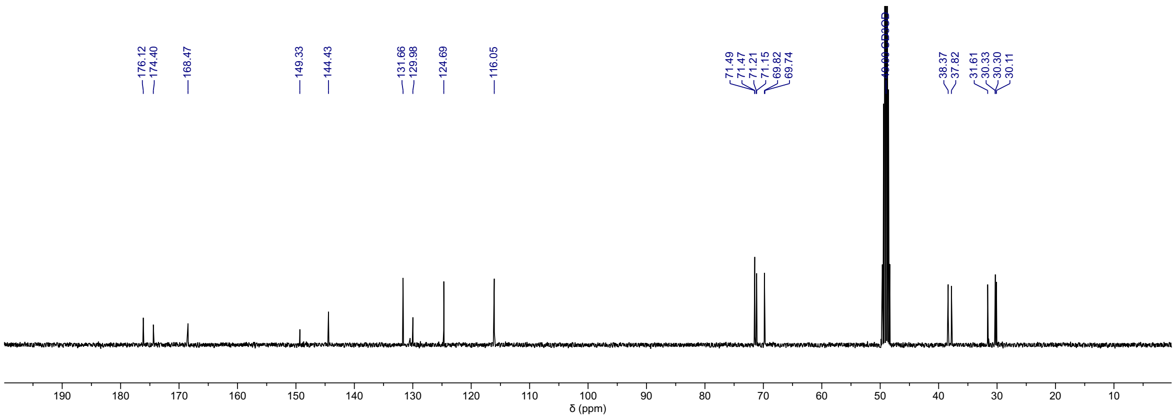

Figure S89. ^{13}C (101 MHz, MeOD-d^{4}, 298 K) NMR spectrum of compound **34**

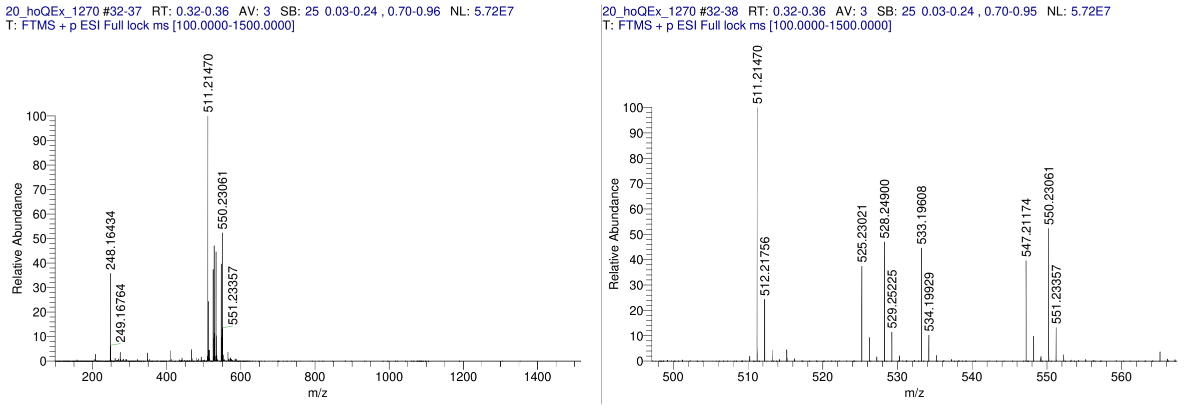

Figure S90. HR-ESI-MS spectrum of compound **34**

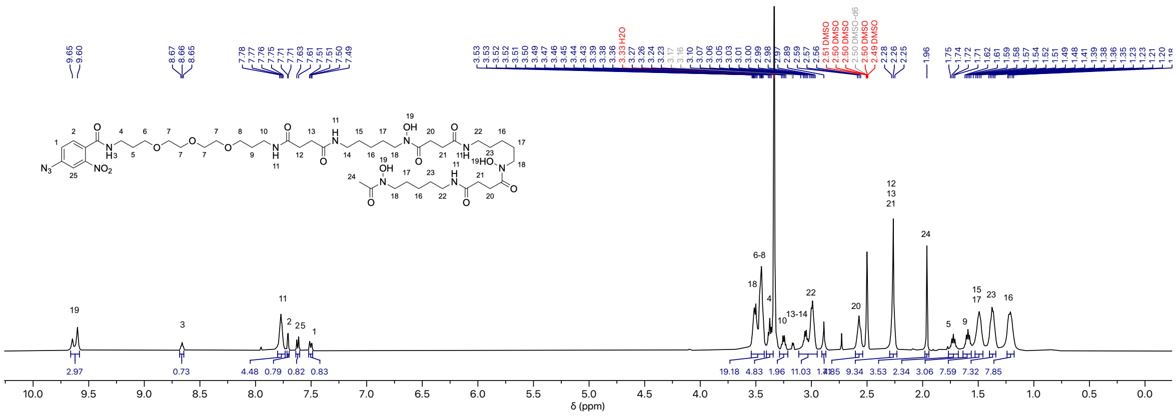

Figure S91. ^{1}H (400 MHz, DMSO-d^{6}, 298 K) NMR spectrum of compound **7**

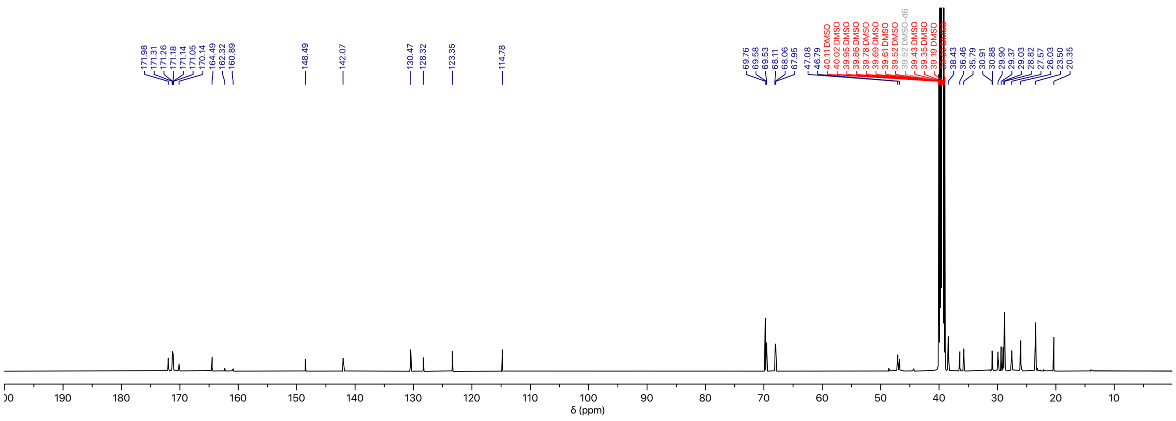

Figure S92. ^{13}C (101 MHz, DMSO-d^{6}, 298 K) NMR spectrum of compound **7**

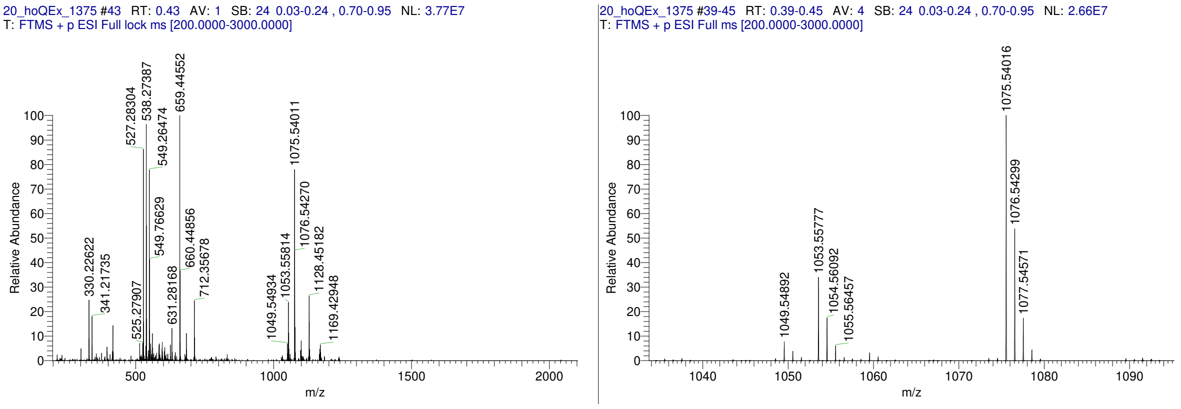

Figure S93. HR-ESI-MS spectrum of compound **7**

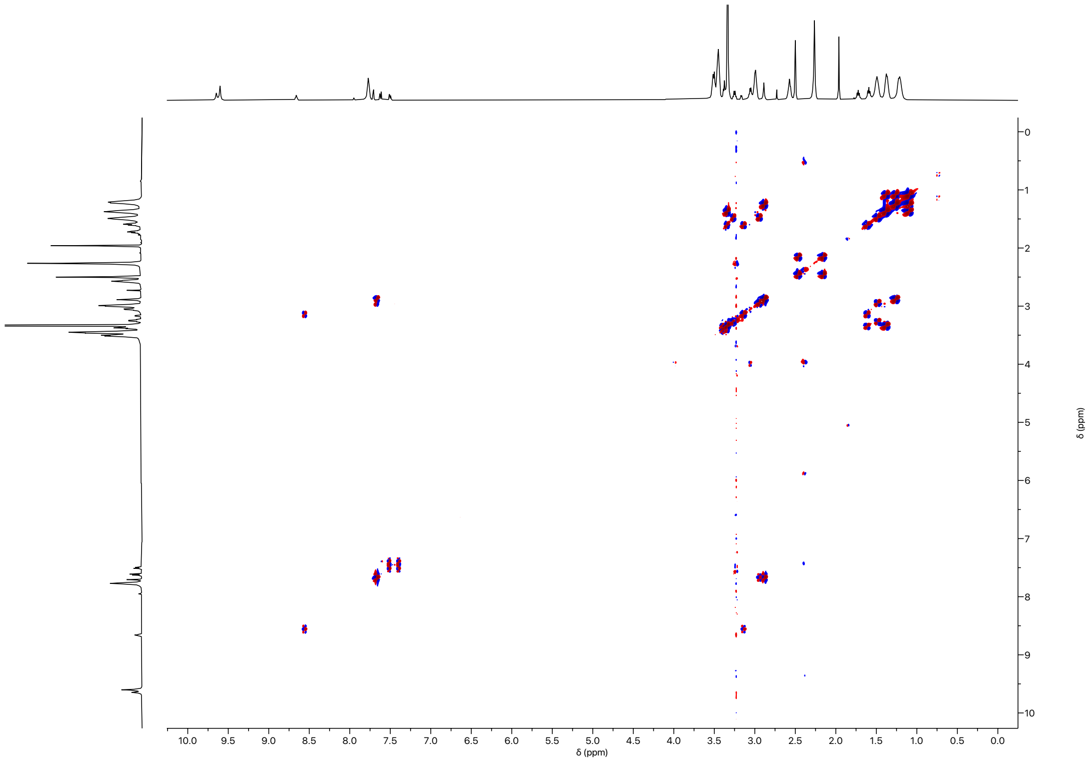

Figure S94. ^{1}H-^{1}H (COSY, DMSO-d^{6}, 298 K) 2D NMR spectrum of compound **7**

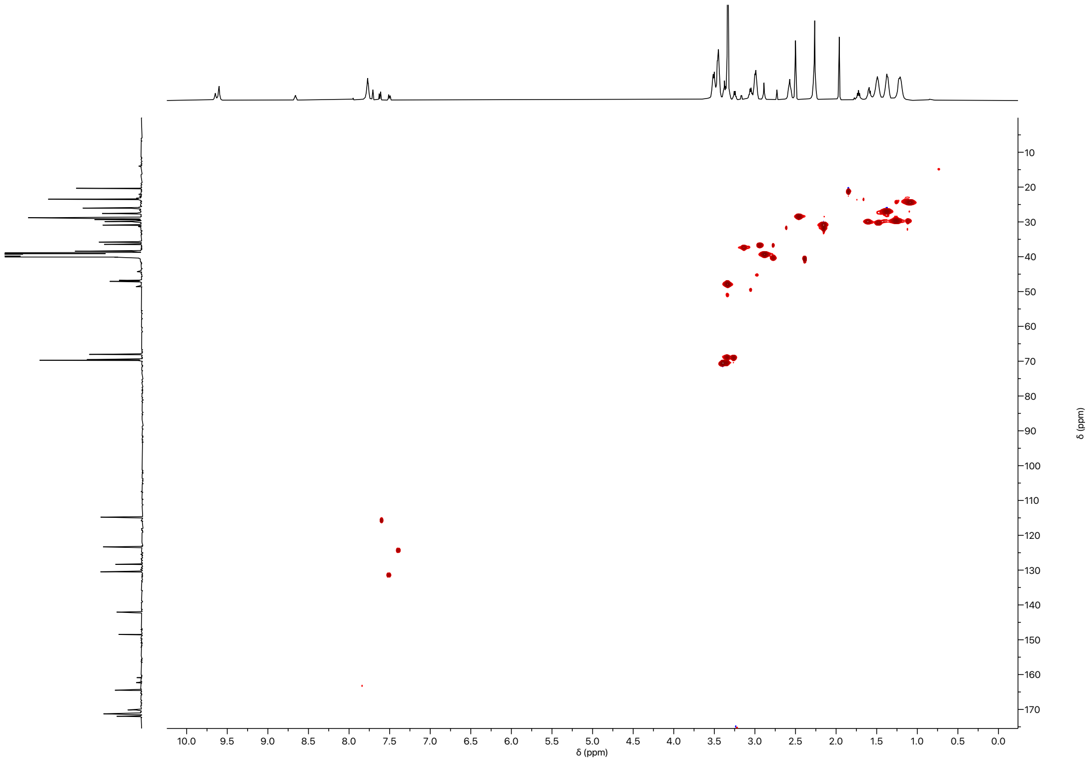

Figure S95. ^{1}H-^{13}C (HSQC, DMSO-d^{6}, 298 K) 2D NMR spectrum of compound **7**

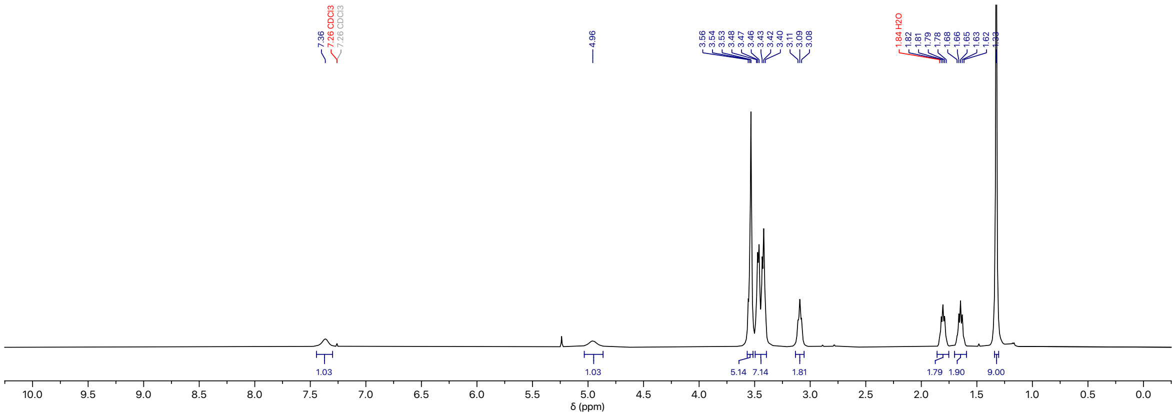

Figure S96. ^{1}H (400 MHz, $CDCl_3$, 298 K) NMR spectrum of compound **35**

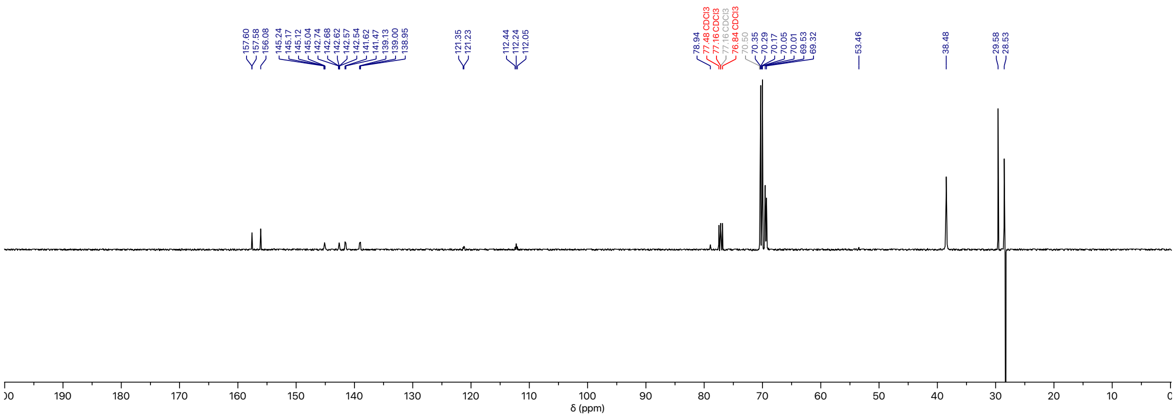

Figure S97. ^{13}C (101 MHz, $CDCl_3$, 298 K) NMR spectrum of compound **35**

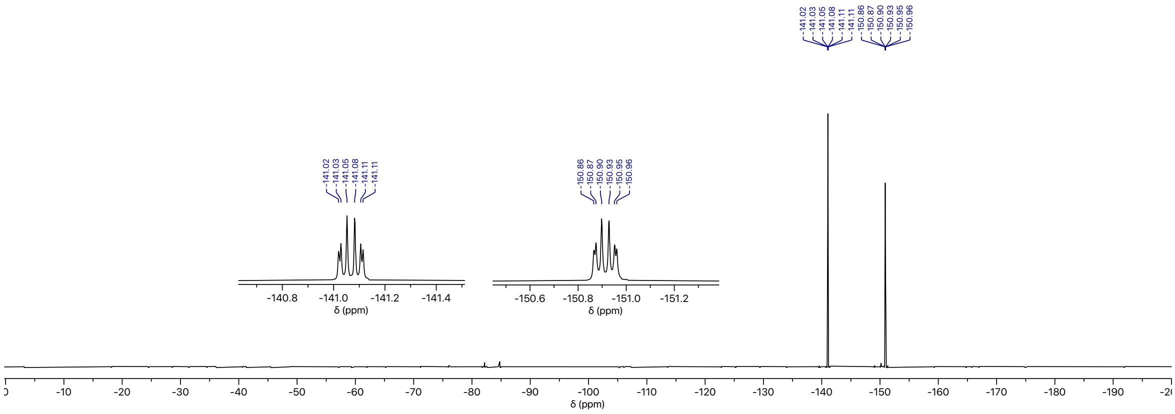

Figure S98. ^{19}F (376 MHz, $CDCl_3$, 298 K) NMR spectrum of compound **35**

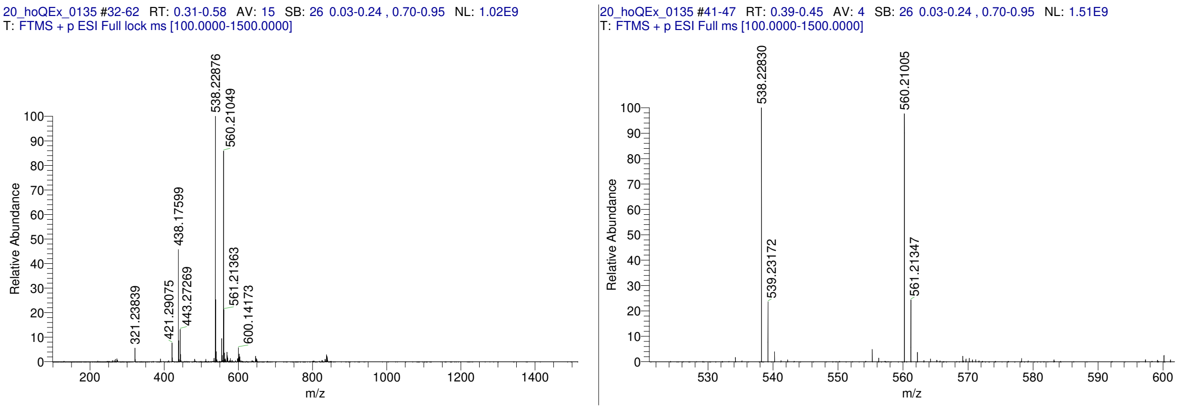

Figure S99. HR-ESI-MS spectrum of compound **35**

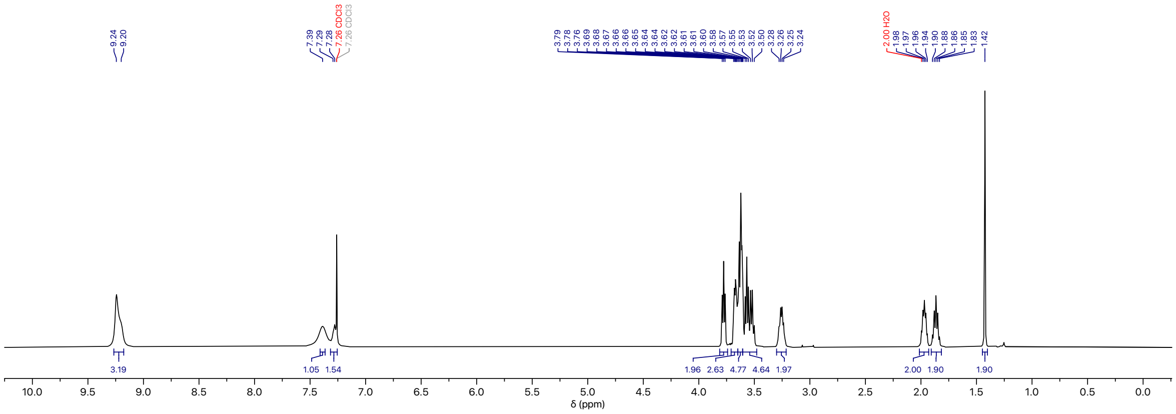

Figure S100. ^{1}H (400 MHz, $CDCl_3$, 298 K) NMR spectrum of compound **36**

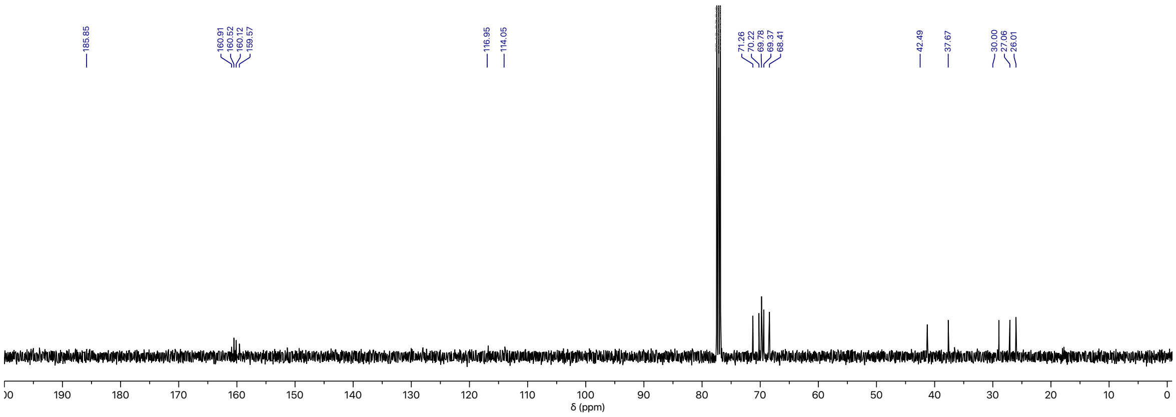

Figure S101. ^{13}C (101 MHz, $CDCl_3$, 298 K) NMR spectrum of compound **36**

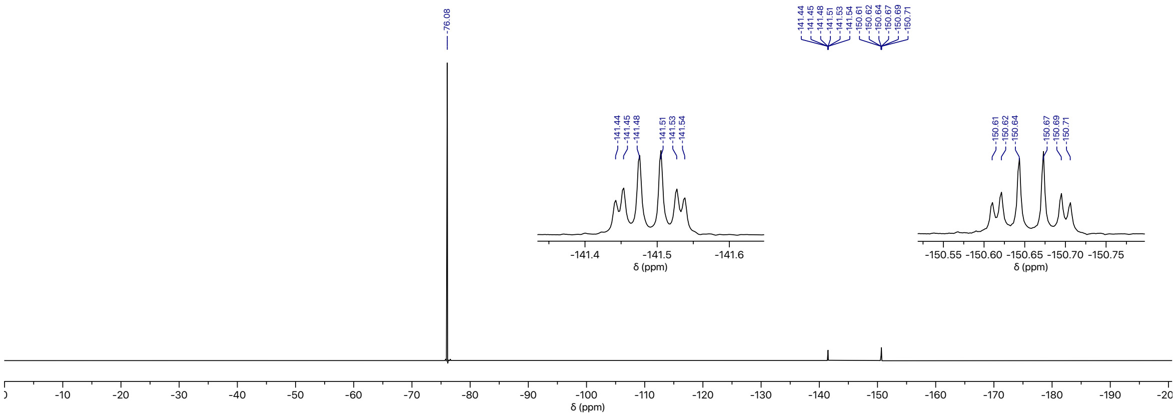

Figure S102. ^{19}F (376 MHz, $CDCl_3$, 298 K) NMR spectrum of compound **36**

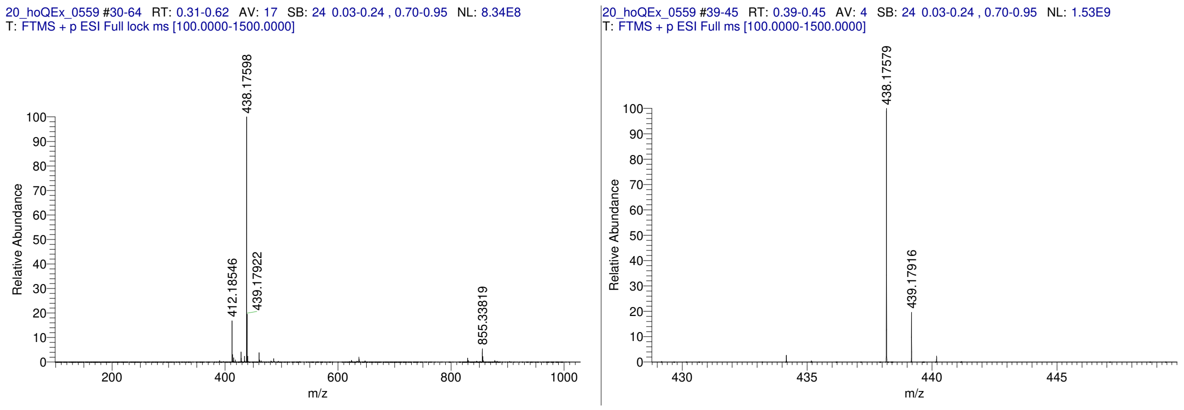

Figure S103. HR-ESI-MS spectrum of compound **36**

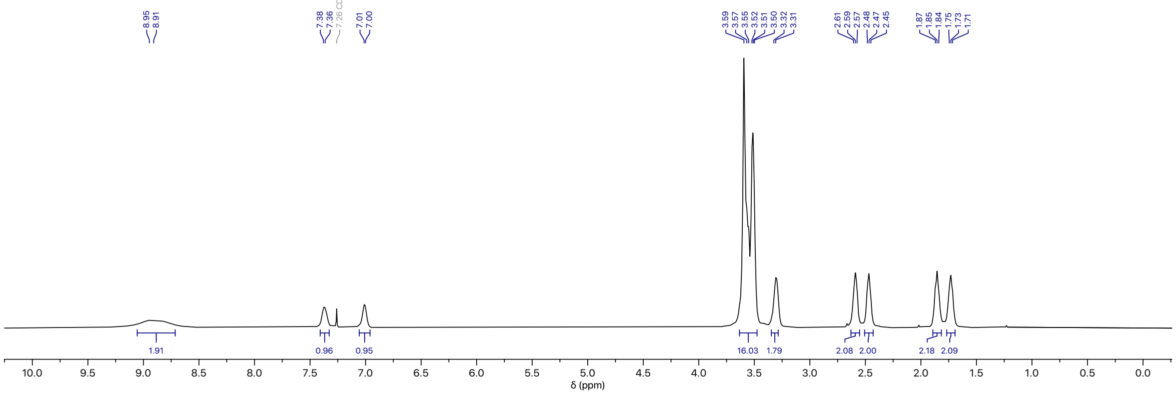

Figure S104. ^{1}H (400 MHz, $CDCl_3$, 298 K) NMR spectrum of compound **37**

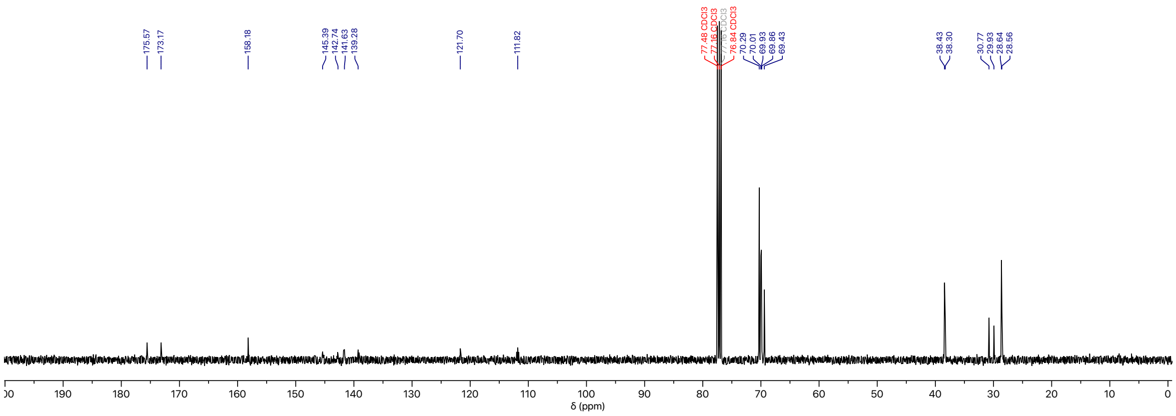

Figure S105. ^{13}C (101 MHz, $CDCl_3$, 298 K) NMR spectrum of compound **37**

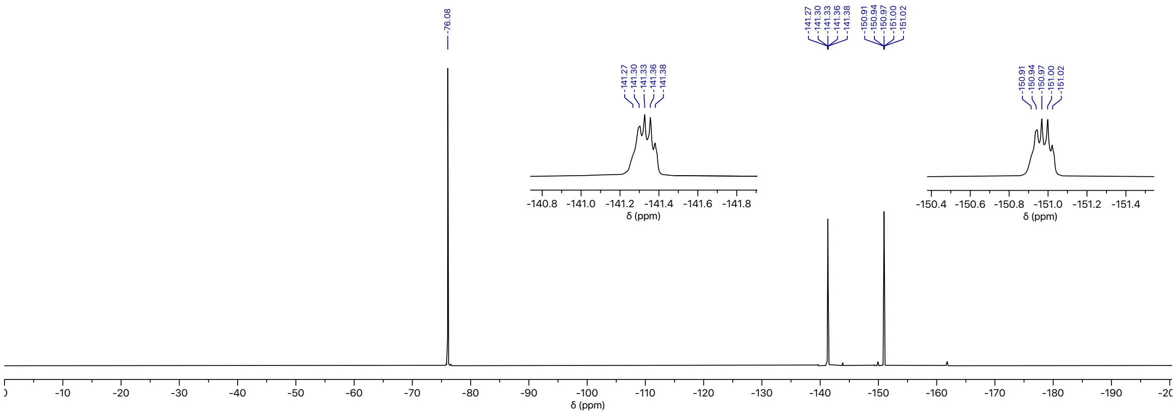

Figure S106. ^{19}F (376 MHz, $CDCl_3$, 298 K) NMR spectrum of compound **37**

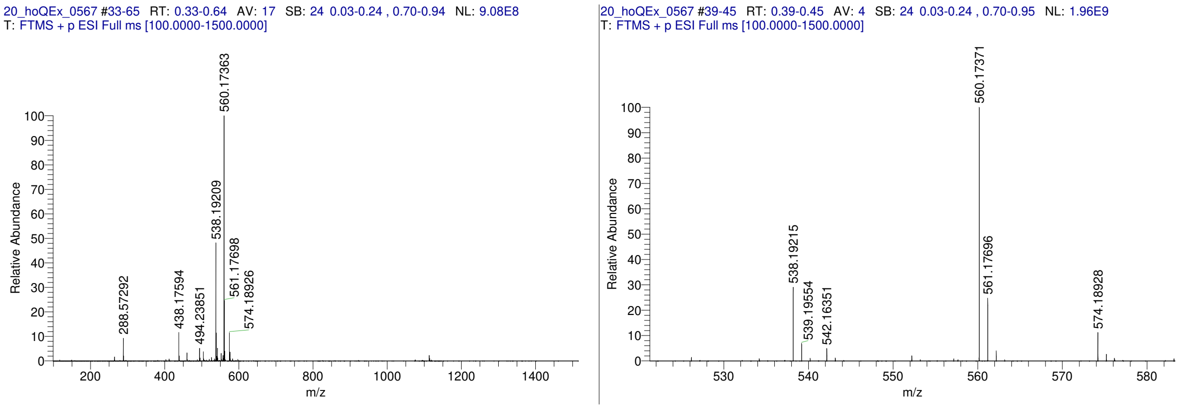

Figure S107. HR-ESI-MS spectrum of compound **37**

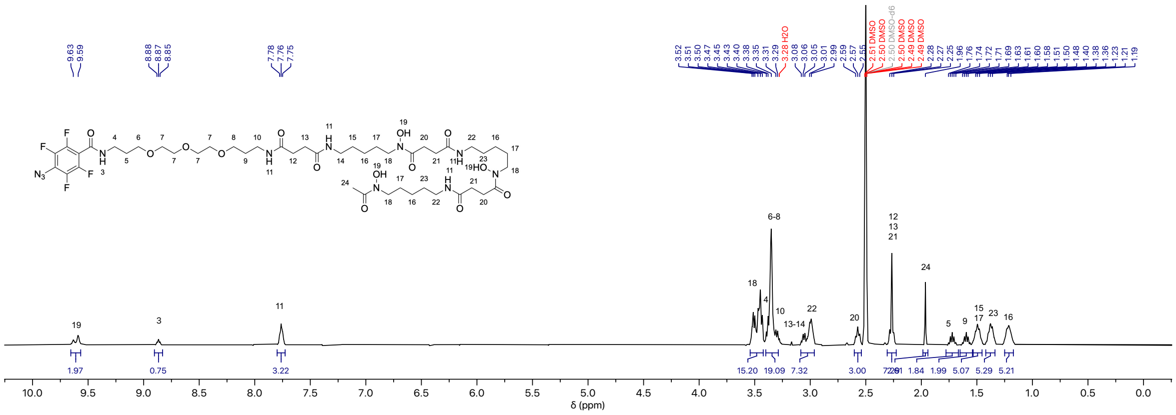

Figure S108. ^{1}H (400 MHz, DMSO-d^{6}, 298 K) NMR spectrum of compound **8**

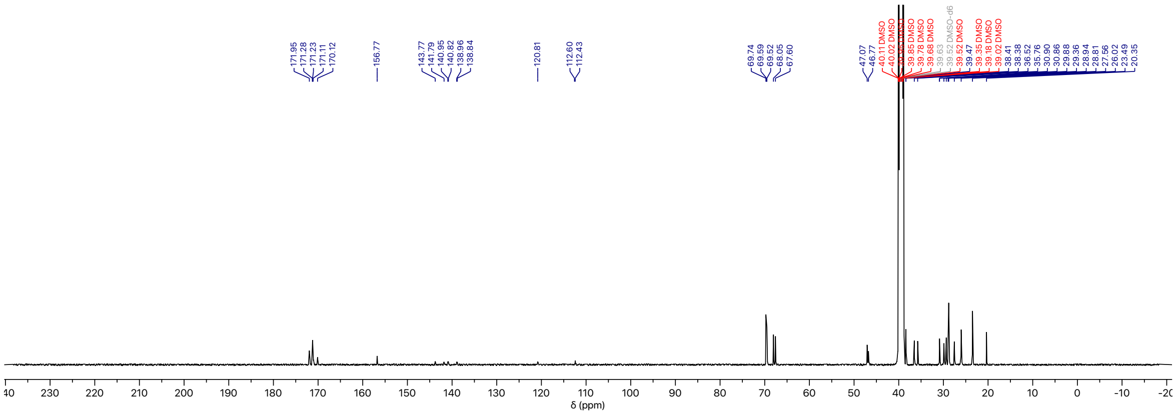

Figure S109. ^{13}C (101 MHz, DMSO-d^{6}, 298 K) NMR spectrum of compound **8**

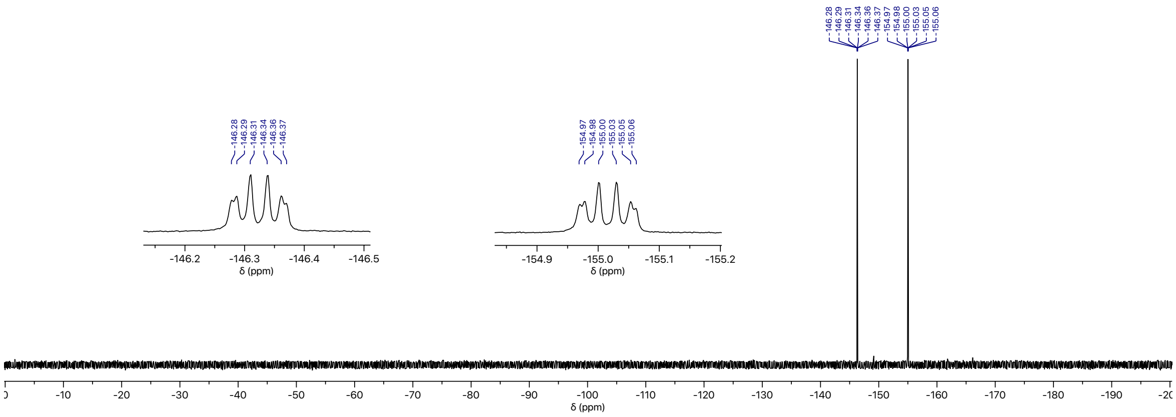

Figure S110. ^{19}F (376 MHz, DMSO-d^{6}, 298 K) NMR spectrum of compound **8**

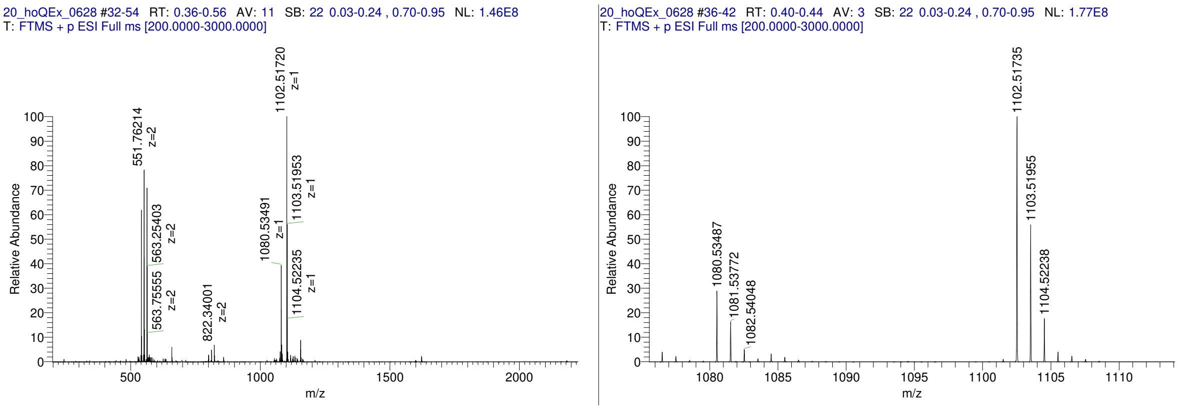

Figure S111. HR-ESI-MS spectrum of compound **8**

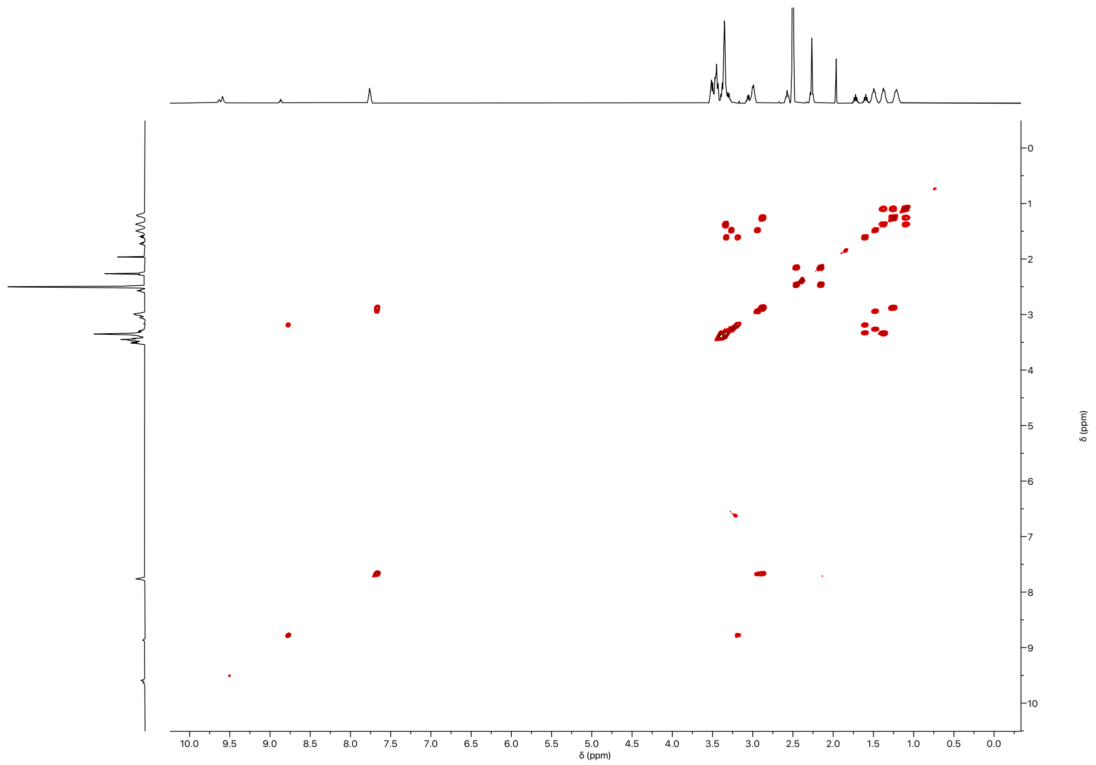

Figure S112. ^1H-^1H (COSY, DMSO-d^6, 298 K) 2D NMR spectrum of compound **8**

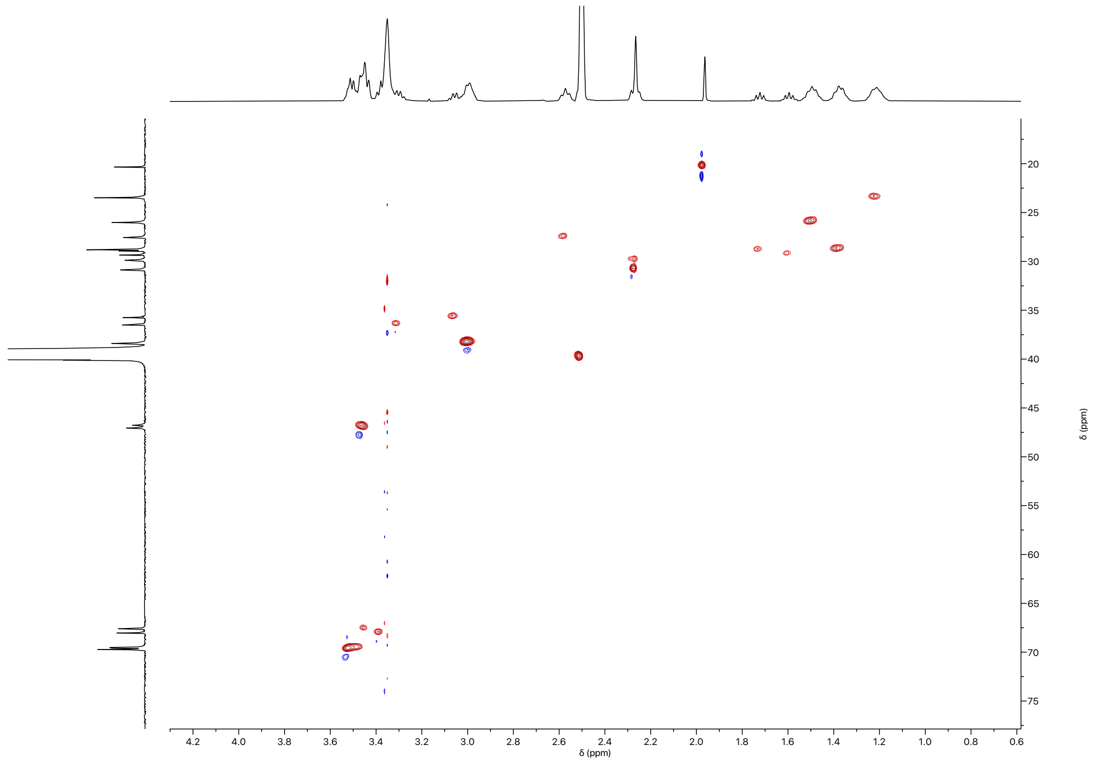

Figure S113. ^1H-^{13}C (HSQC, DMSO-d^6, 298 K) 2D NMR spectrum of compound **8**

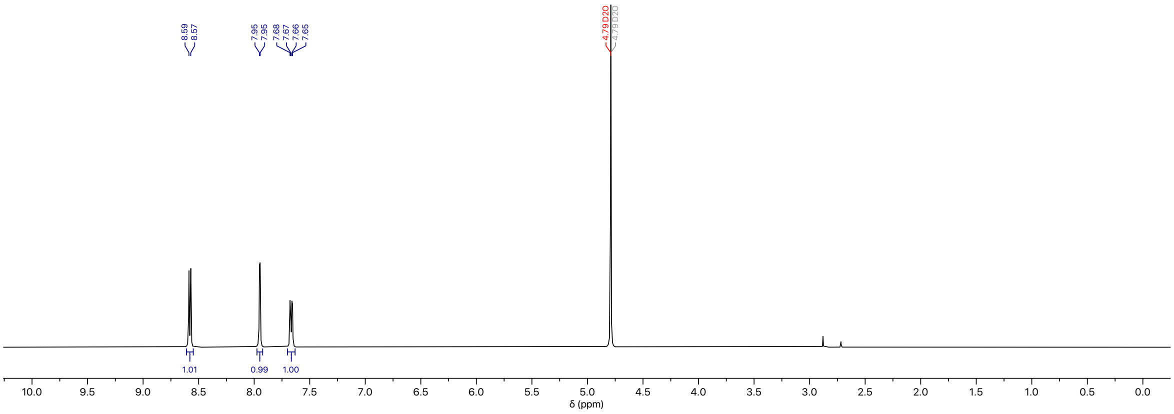

Figure S114. ^1H (400 MHz, D_2O, 298 K) NMR spectrum of compound **38**

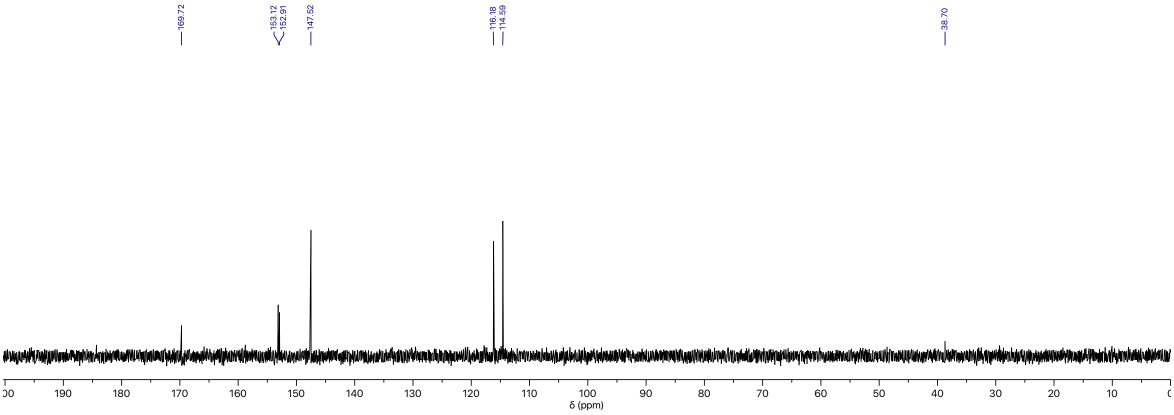

Figure S115. ^{13}C (101 MHz, D_2O, 298 K) NMR spectrum of compound **38**

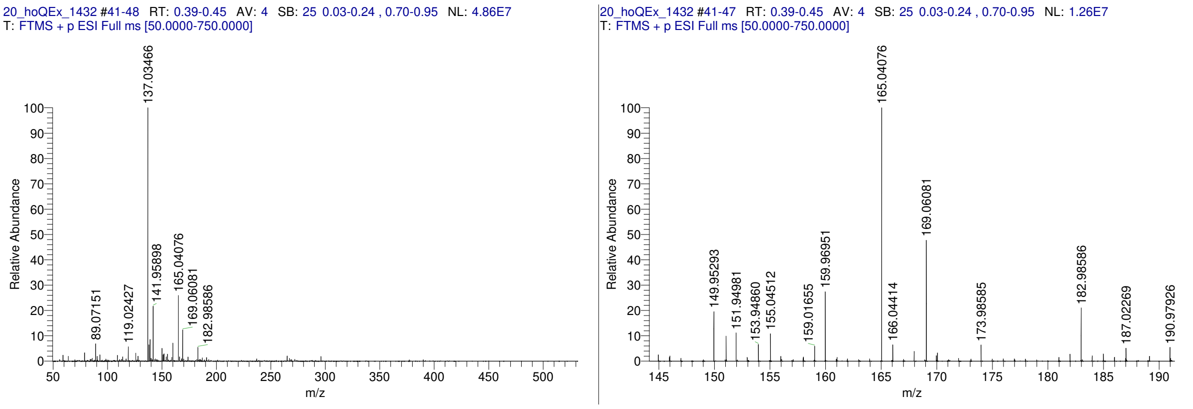

Figure S116. HR-ESI-MS spectrum of compound **38**

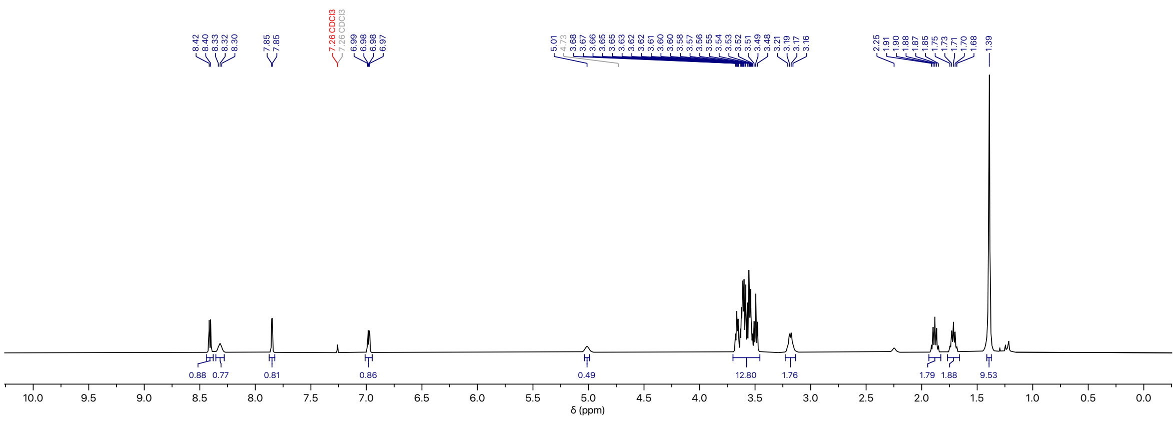

Figure S117. ^{1}H (400 MHz, $CDCl_3$, 298 K) NMR spectrum of compound **39**

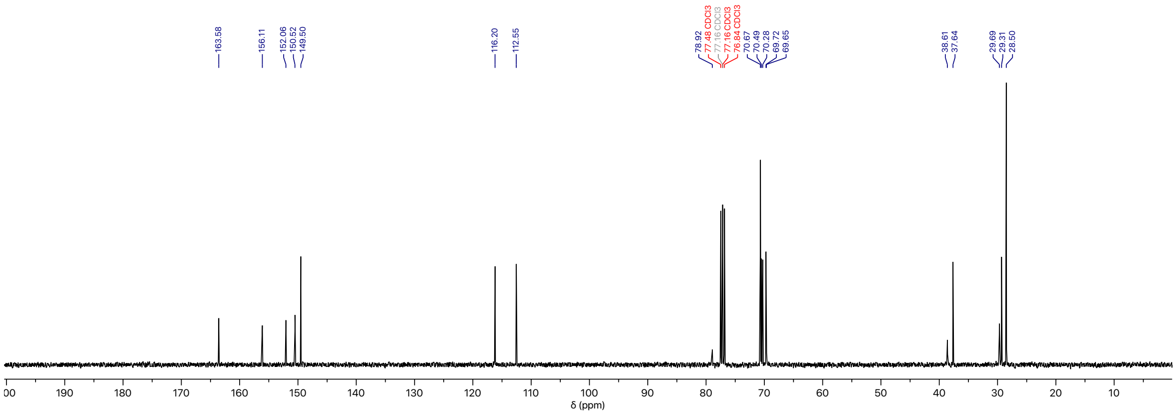

Figure S118. ^{13}C (101 MHz, $CDCl_3$, 298 K) NMR spectrum of compound **39**

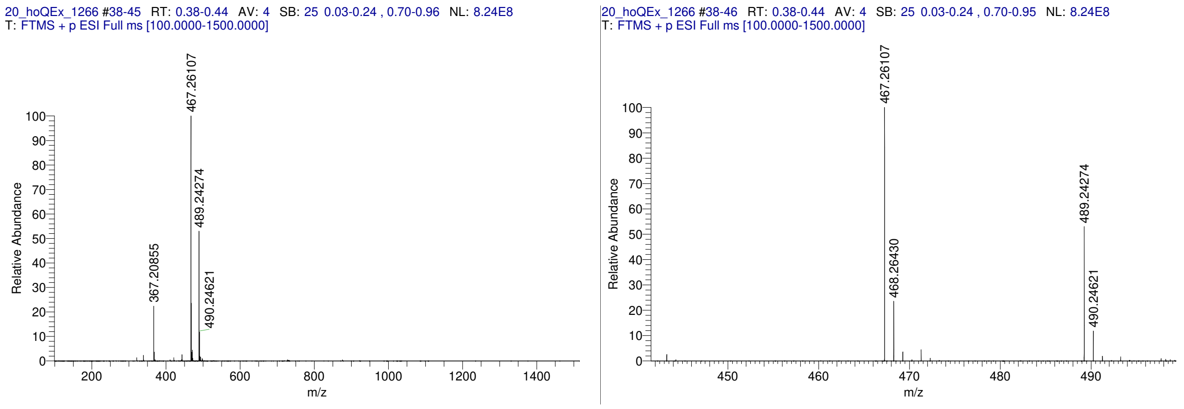

Figure S119. HR-ESI-MS spectrum of compound **39**

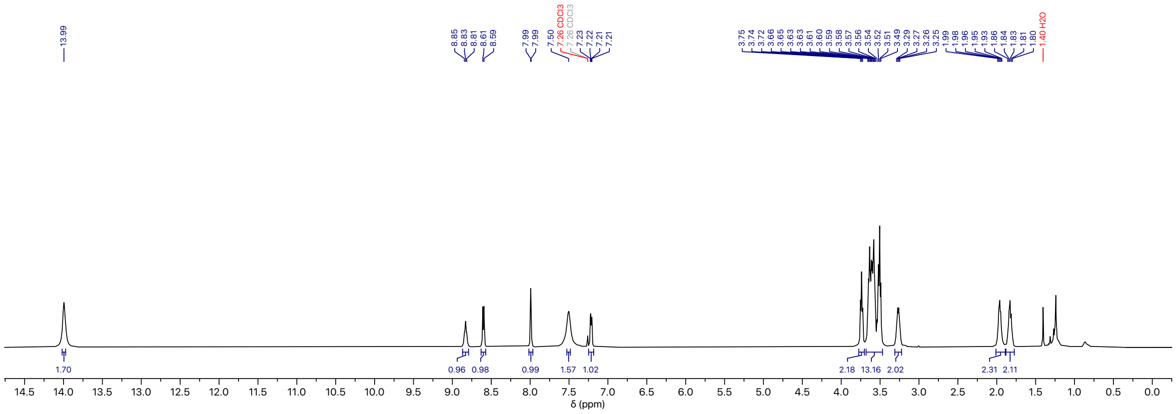

Figure S120. ^{1}H (400 MHz, $CDCl_3$, 298 K) NMR spectrum of compound **40**

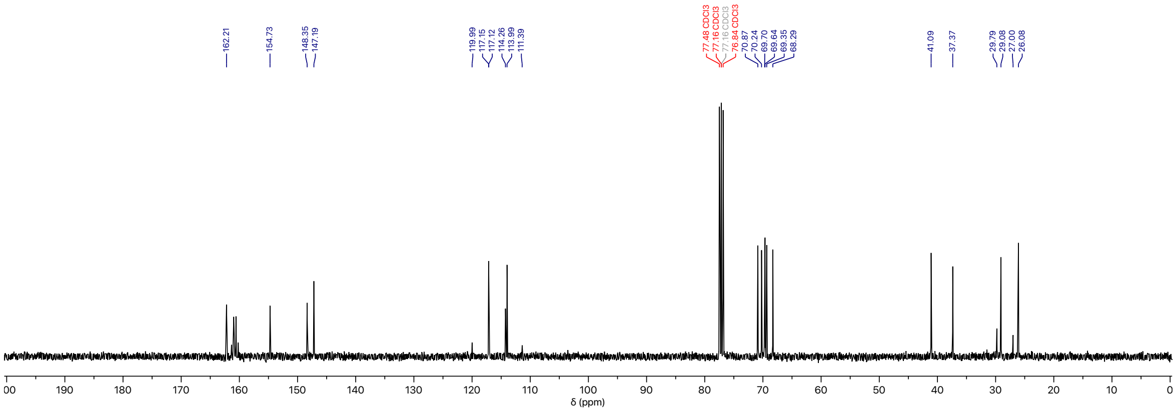

Figure S121. ^{13}C (101 MHz, $CDCl_3$, 298 K) NMR spectrum of compound **40**

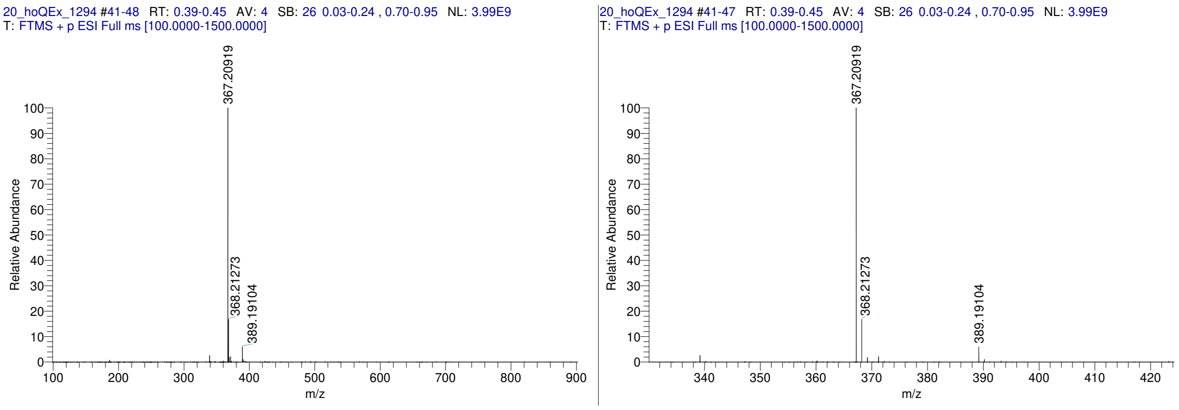

Figure S122. HR-ESI-MS spectrum of compound **40**

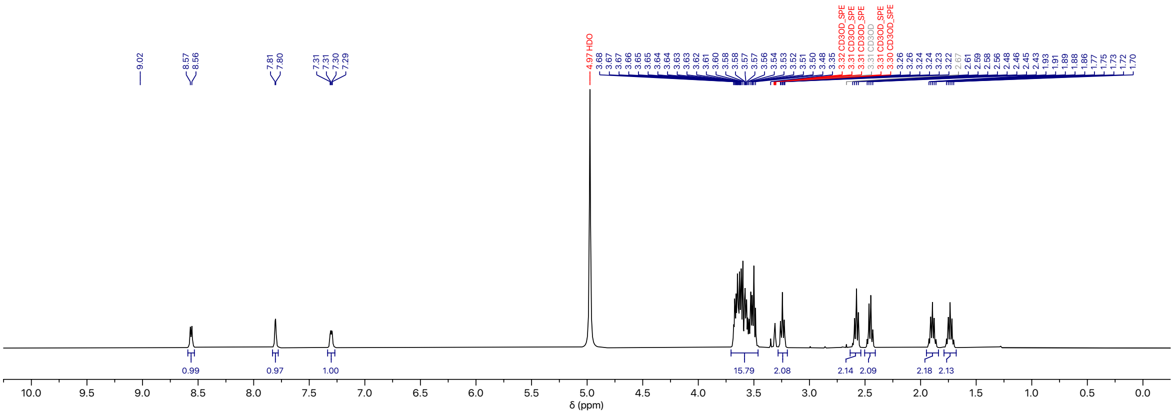

Figure S123. ^{1}H (400 MHz, MeOD-d^{4}, 298 K) NMR spectrum of compound **41**

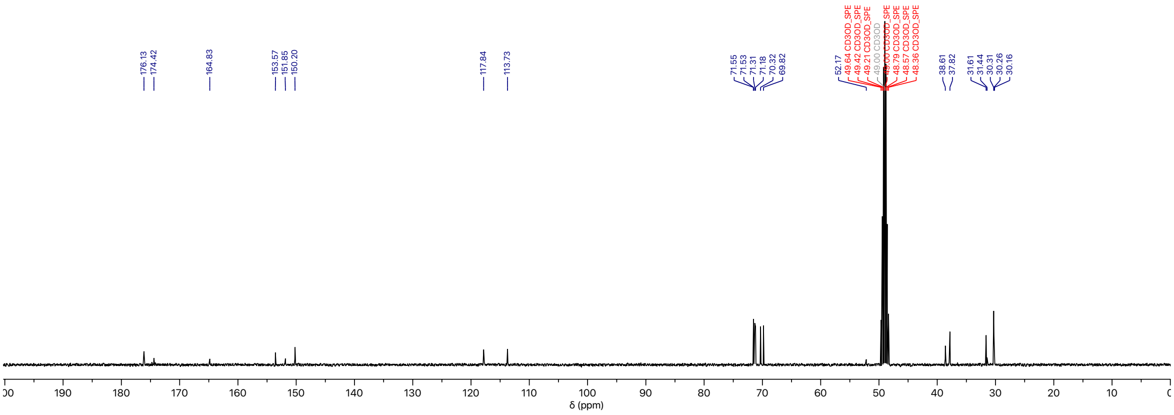

Figure S124. ^{13}C (101 MHz, MeOD-d^{4}, 298 K) NMR spectrum of compound **41**

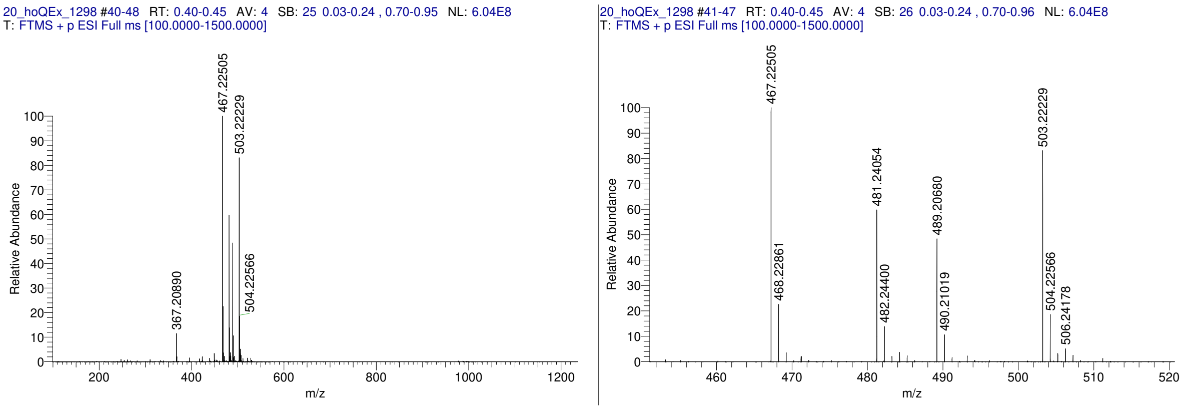

Figure S125. HR-ESI-MS spectrum of compound **41**

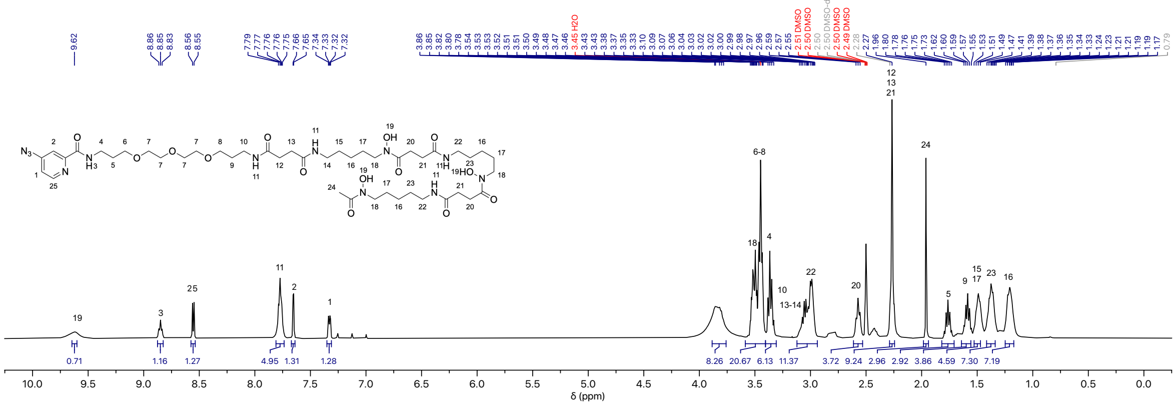

Figure S126. ^{1}H (400 MHz, DMSO-d^{6}, 298 K) NMR spectrum of compound **9**

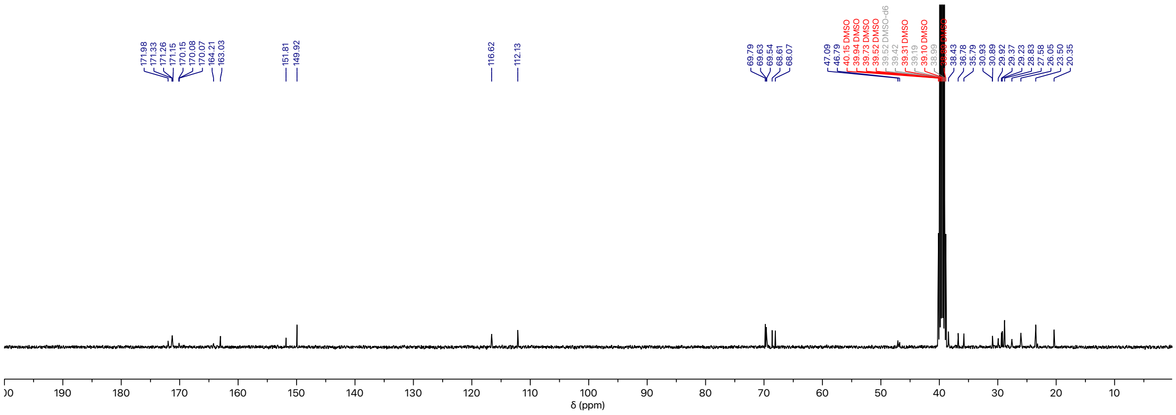

Figure S127. ^{13}C (101 MHz, DMSO-d^{6}, 298 K) NMR spectrum of compound **9**

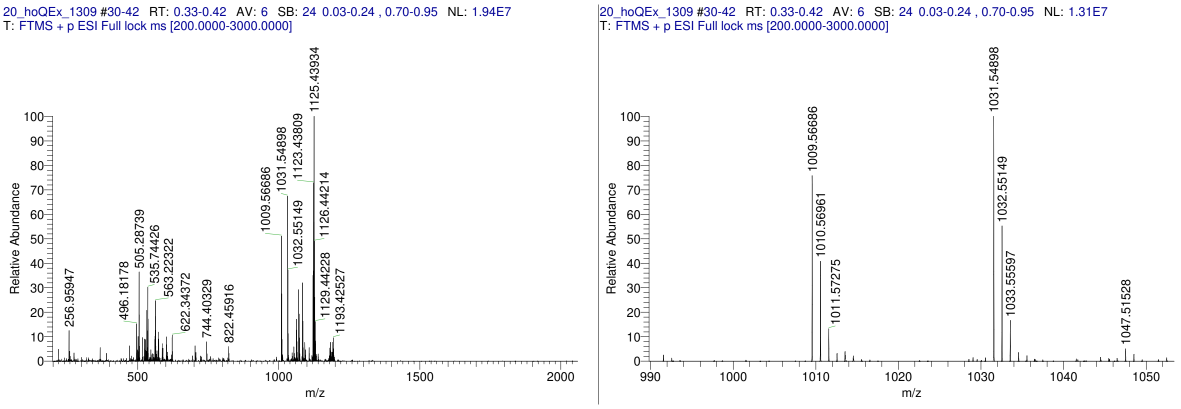

Figure S128. HR-ESI-MS spectrum of compound **9**

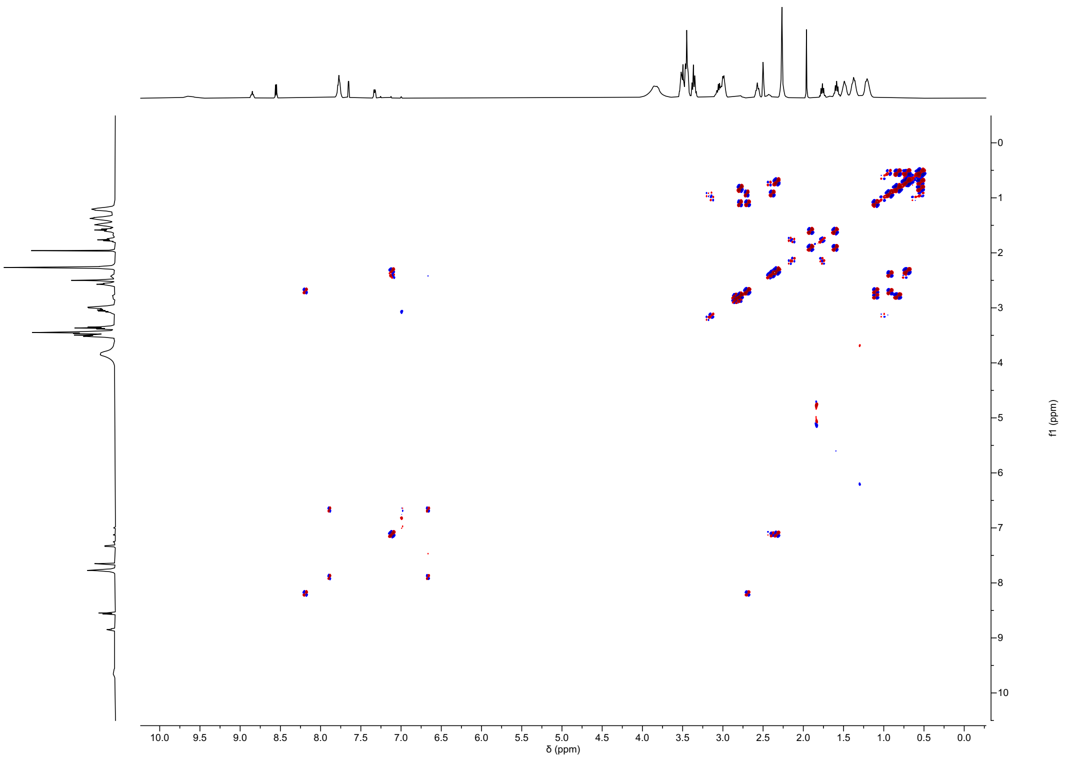

Figure S129. ^{1}H-^{1}H (COSY, DMSO-d^{6}, 298 K) 2D NMR spectrum of compound **9**

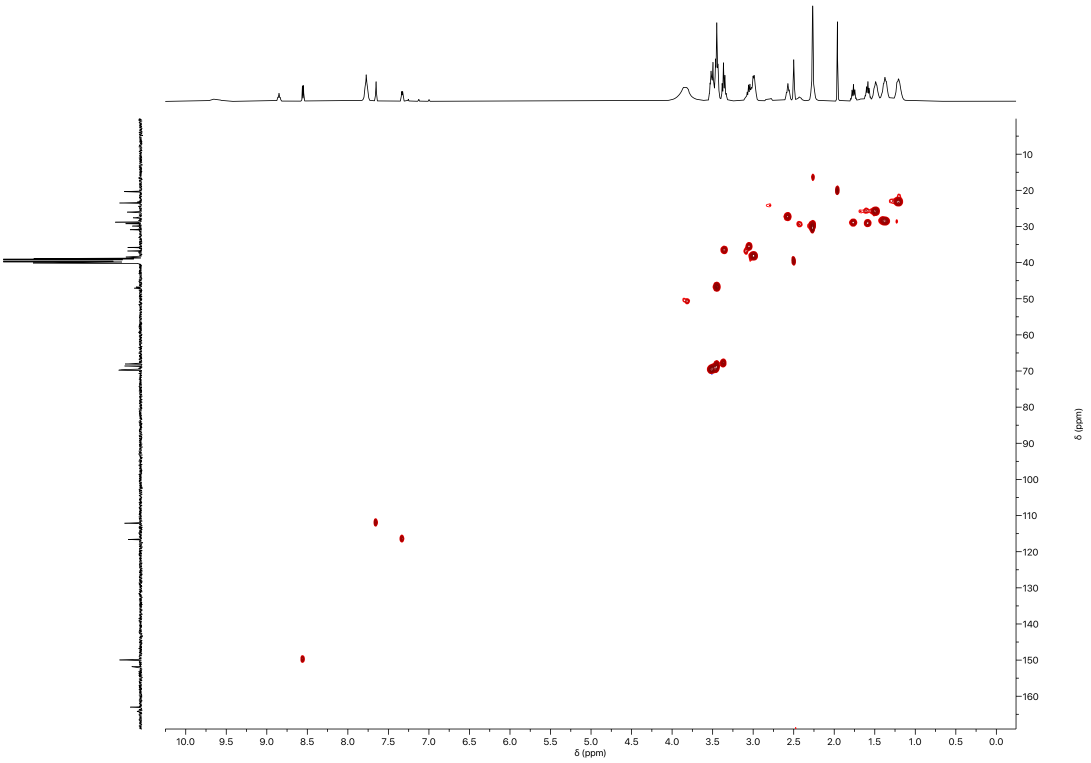

Figure S130. ^{1}H-^{13}C (HSQC, DMSO-d^{6}, 298 K) 2D NMR spectrum of compound **9**

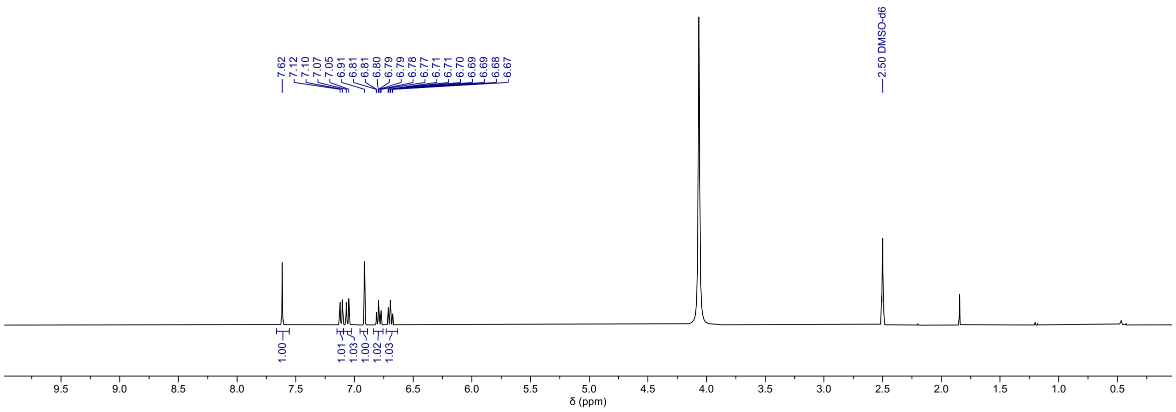

Figure S131. ^{1}H (400 MHz, $CDCl_3$, 298 K) NMR spectrum of compound **42**

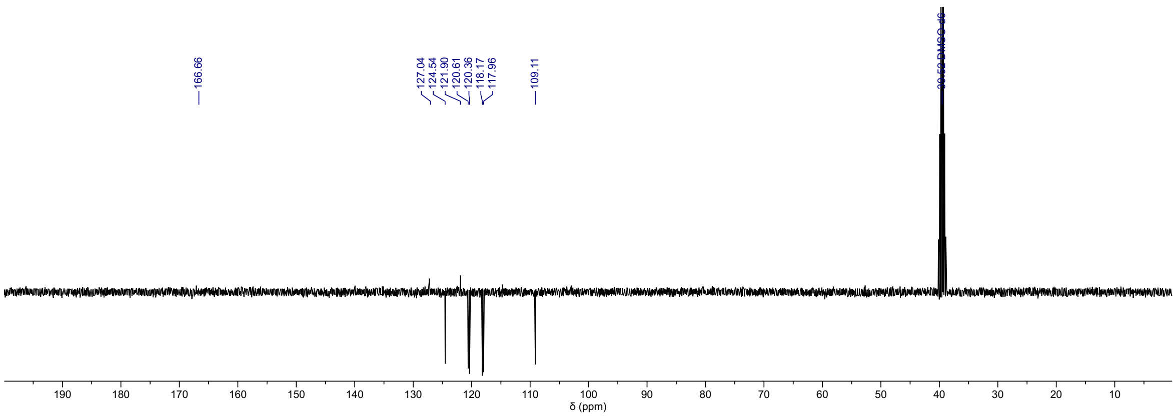

Figure S132. ^{13}C (101 MHz, $CDCl_3$, 298 K) NMR spectrum of compound **42**

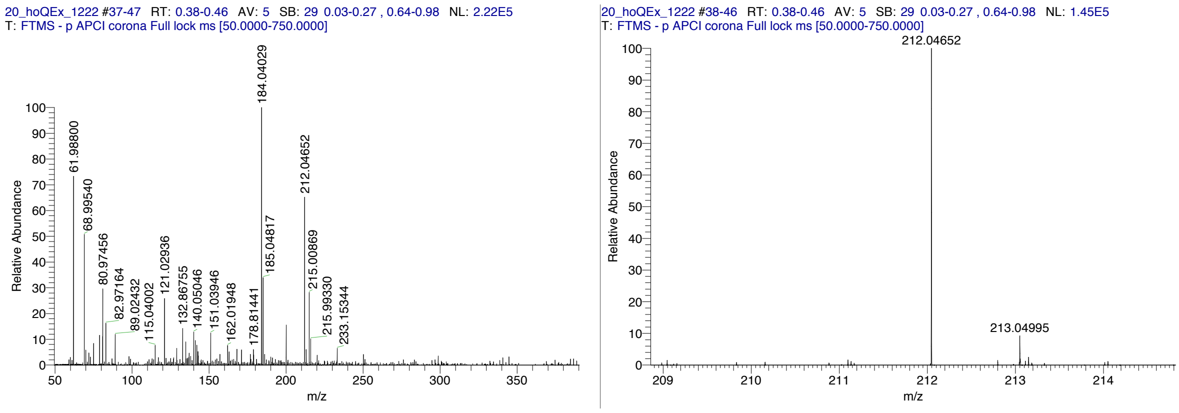

Figure S133. HR-ESI-MS spectrum of compound **42**

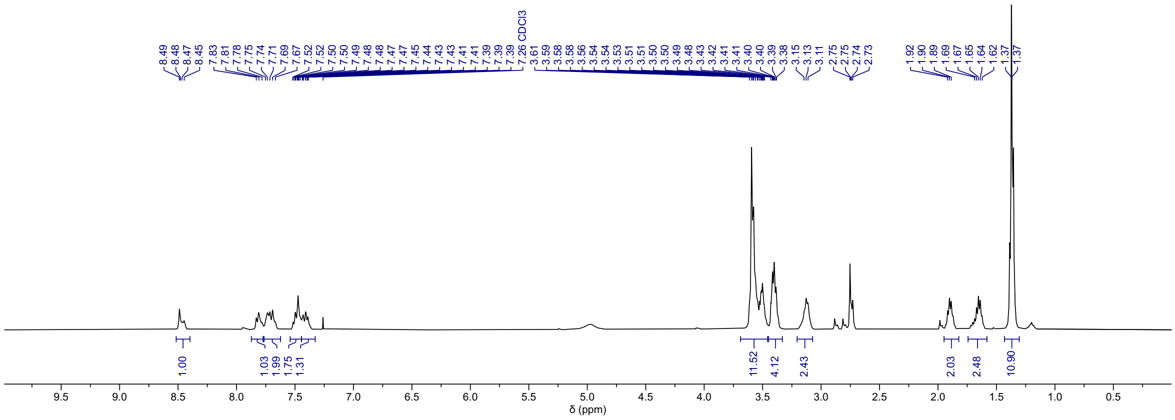

Figure S134. ^{1}H (400 MHz, $CDCl_3$, 298 K) NMR spectrum of compound **43**

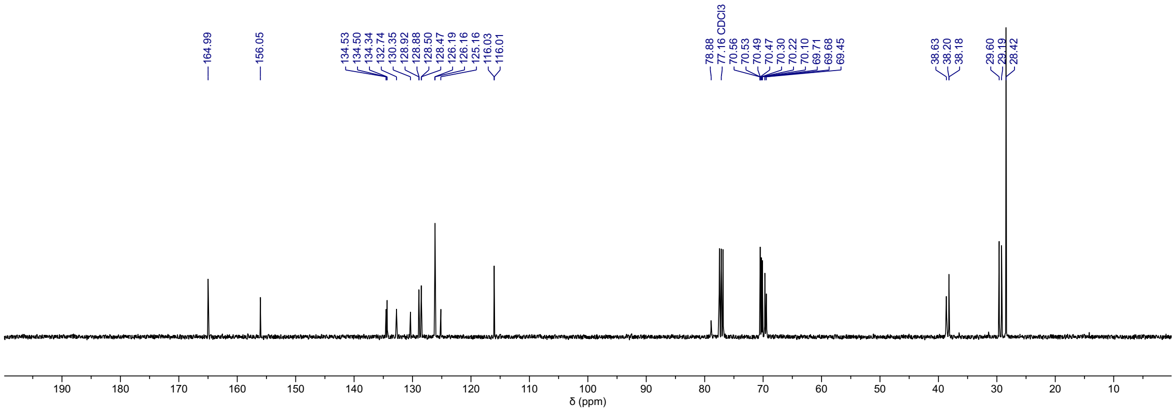

Figure S135. ^{13}C (101 MHz, $CDCl_3$, 298 K) NMR spectrum of compound **43**

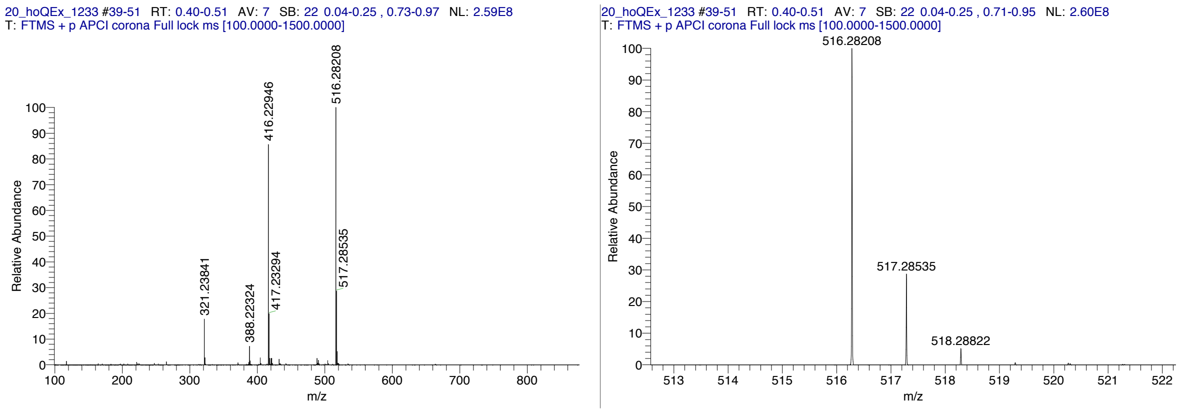

Figure S136. HR-ESI-MS spectrum of compound **43**

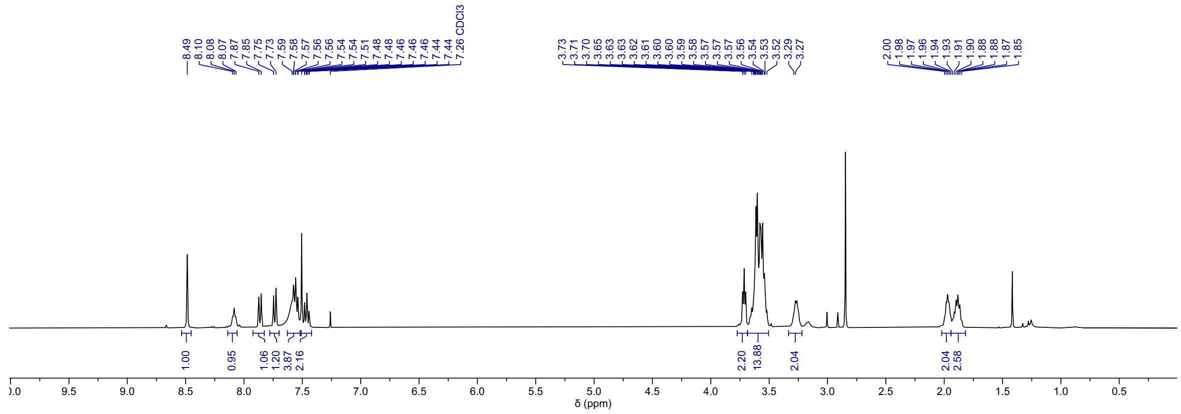

Figure S137. ^{1}H (400 MHz, $CDCl_3$, 298 K) NMR spectrum of compound **44**

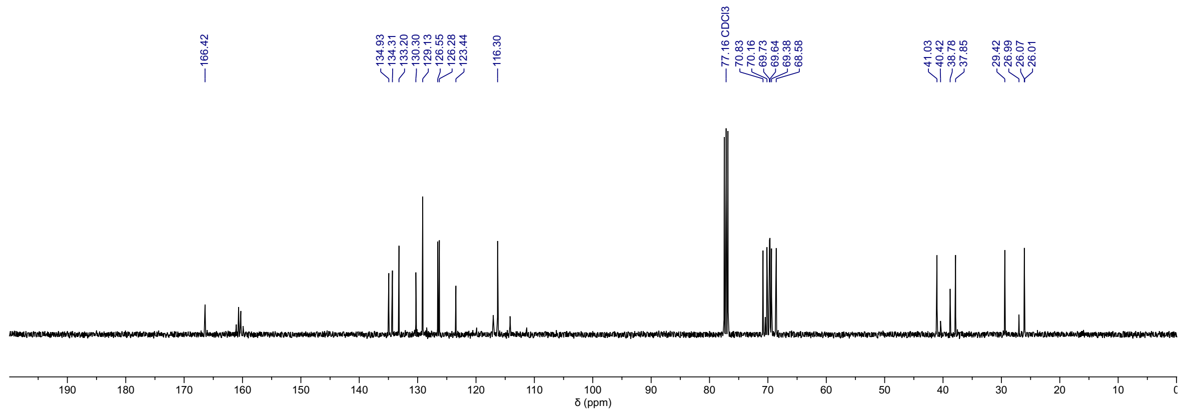

Figure S138. ^{13}C (101 MHz, $CDCl_3$, 298 K) NMR spectrum of compound **44**

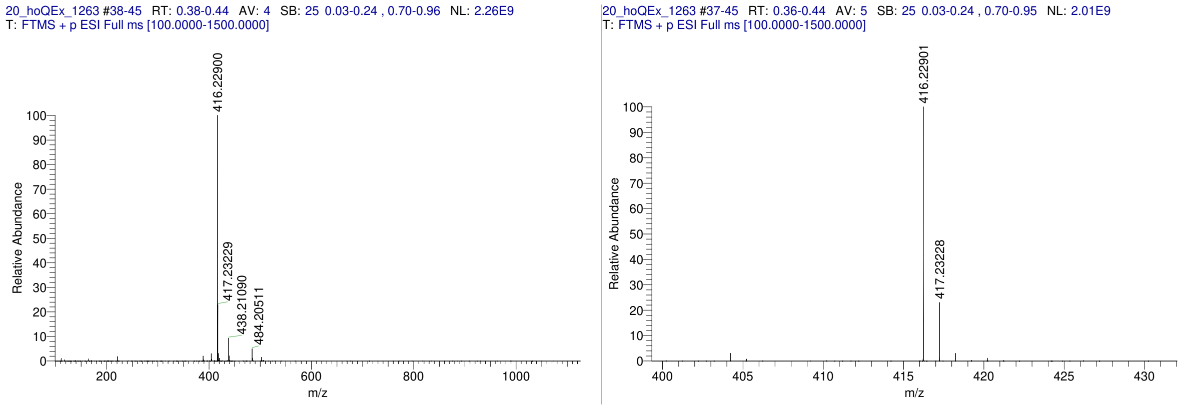

Figure S139. HR-ESI-MS spectrum of compound **44**

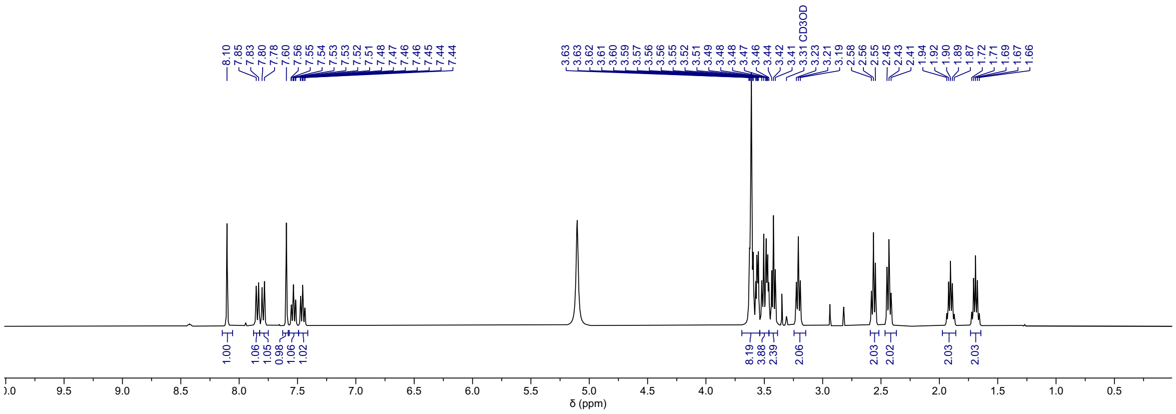

Figure S140. ^{1}H (400 MHz, MeOD-d^4, 298 K) NMR spectrum of compound **45**

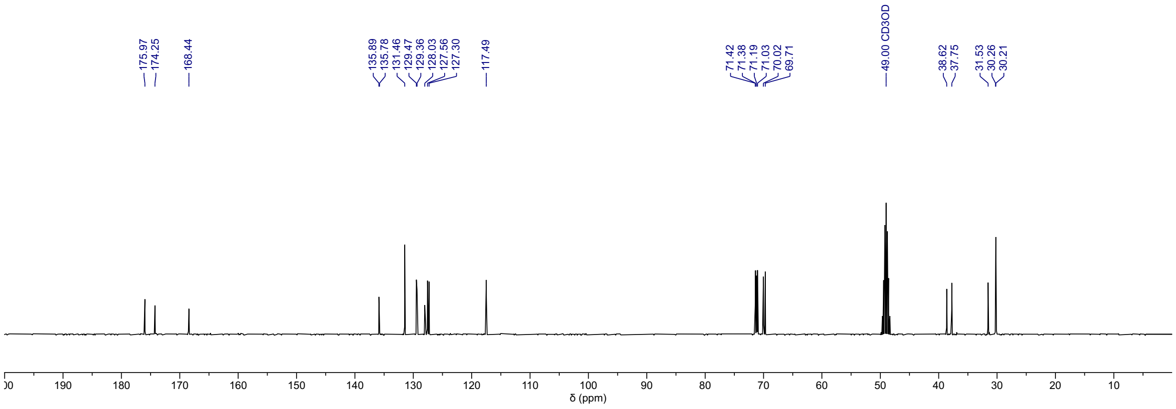

Figure S141. ^{13}C (101 MHz, MeOD-d^4, 298 K) NMR spectrum of compound **45**

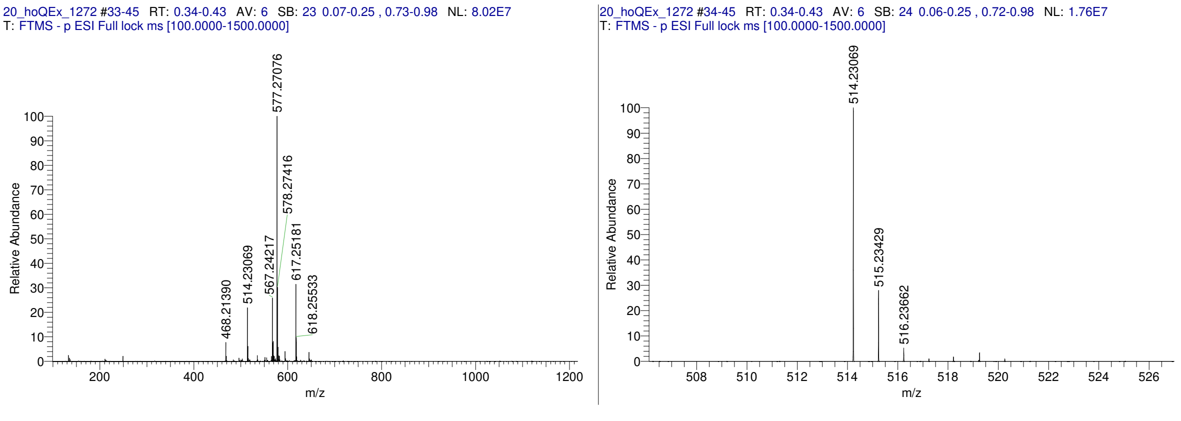

Figure S142. HR-ESI-MS spectrum of compound **45**

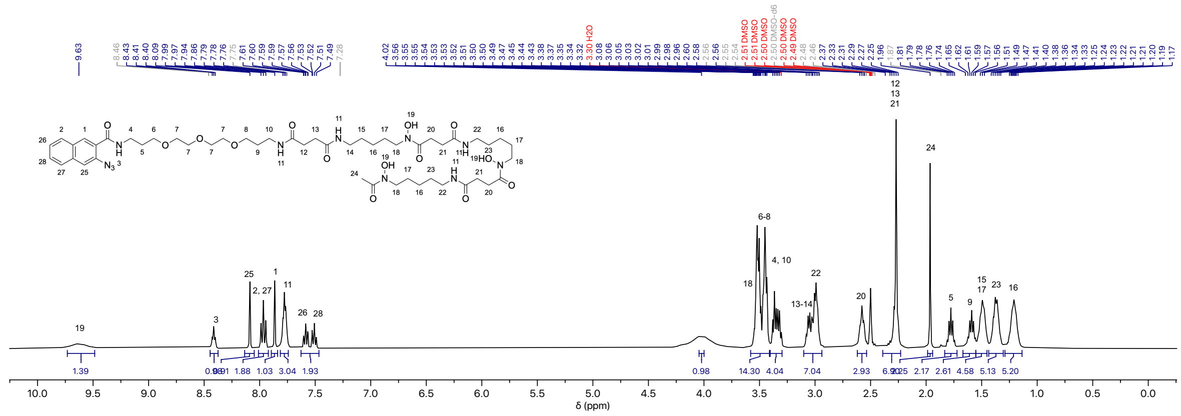

Figure S143. ^{1}H (400 MHz, DMSO-d^{6}, 298 K) NMR spectrum of compound **10**

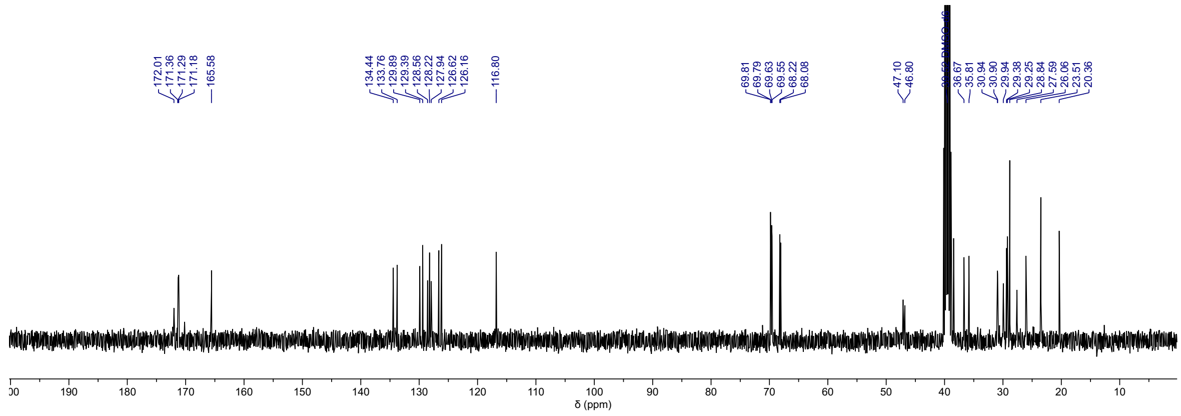

Figure S144. ^{13}C (101 MHz, DMSO-d^{6}, 298 K) NMR spectrum of compound **10**

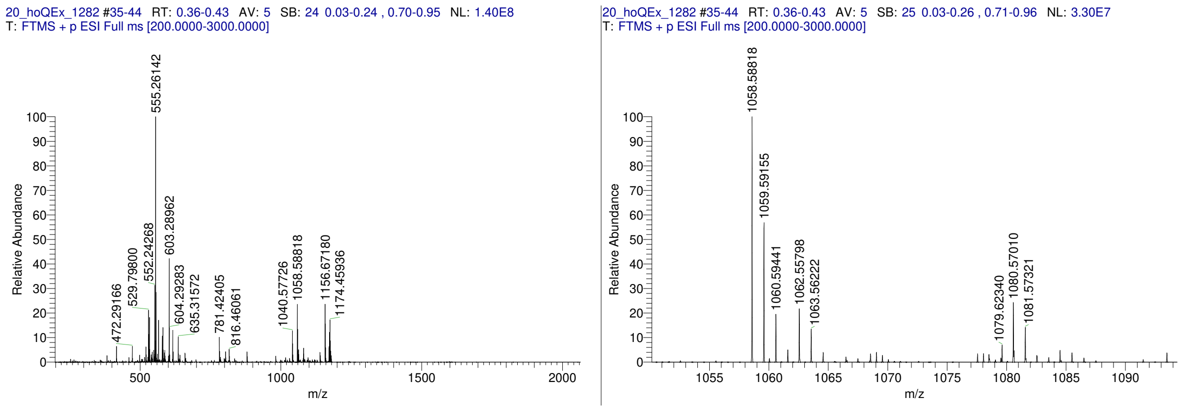

Figure S145. HR-ESI-MS spectrum of compound **10**

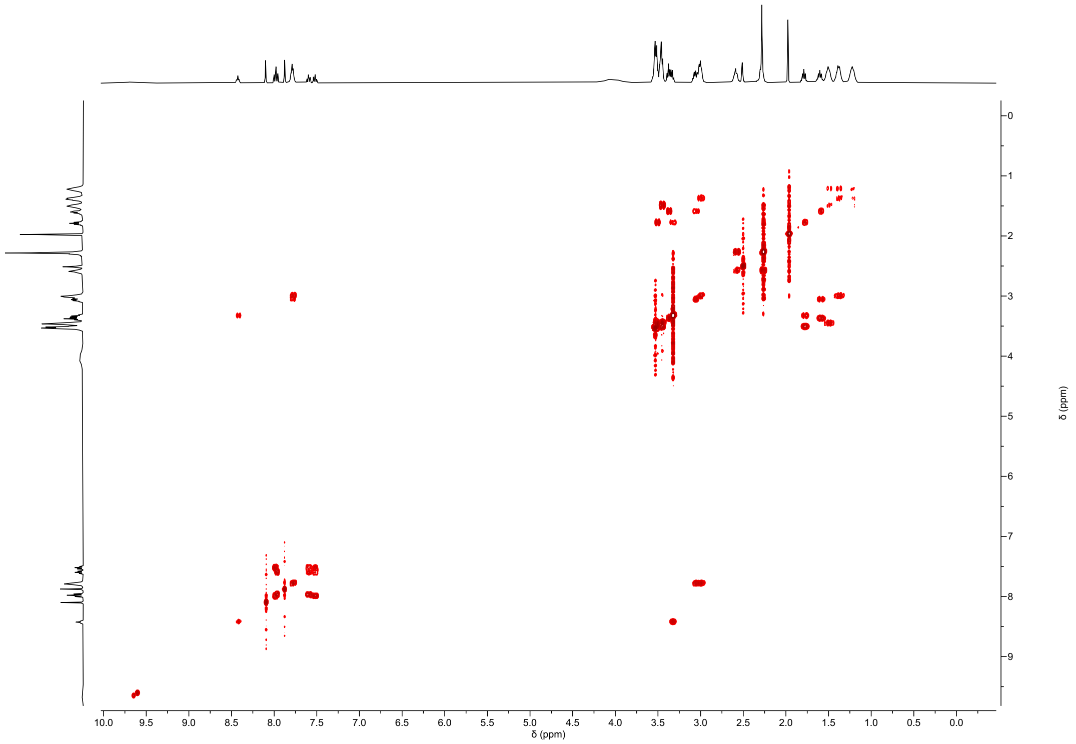

Figure S146. ^1H-^1H (COSY, DMSO-d^6, 298 K) 2D NMR spectrum of compound **10**

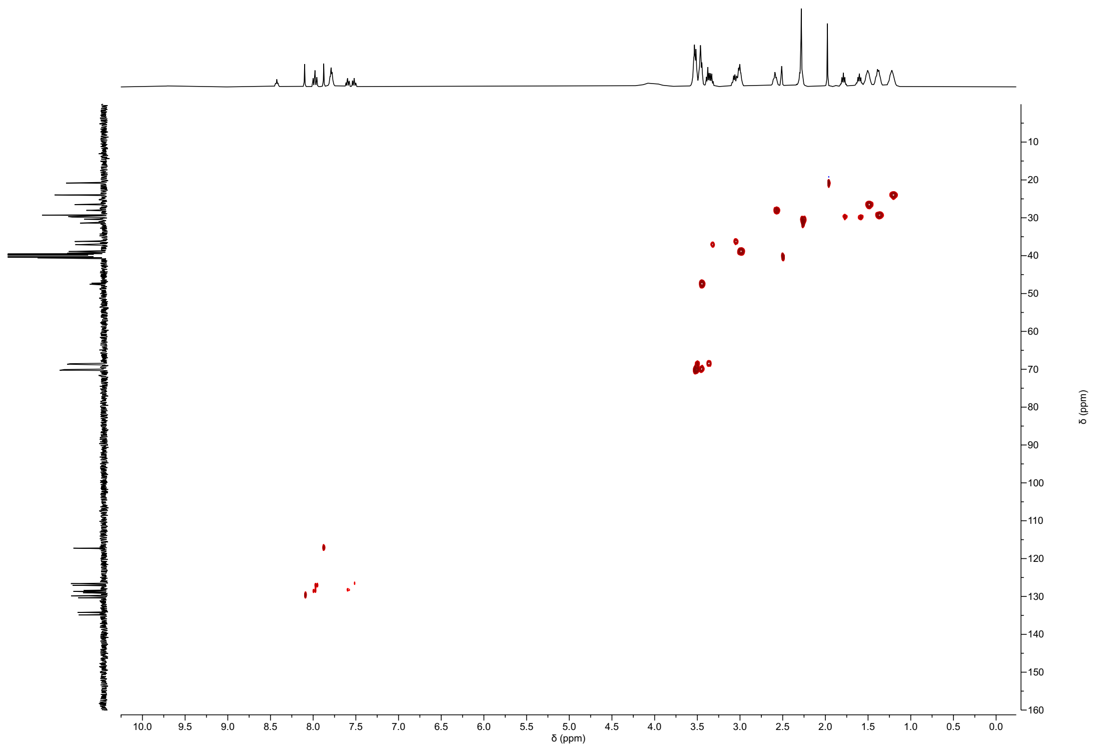

Figure S147. ^1H-^{13}C (HSQC, DMSO-d^6, 298 K) 2D NMR spectrum of compound **10**

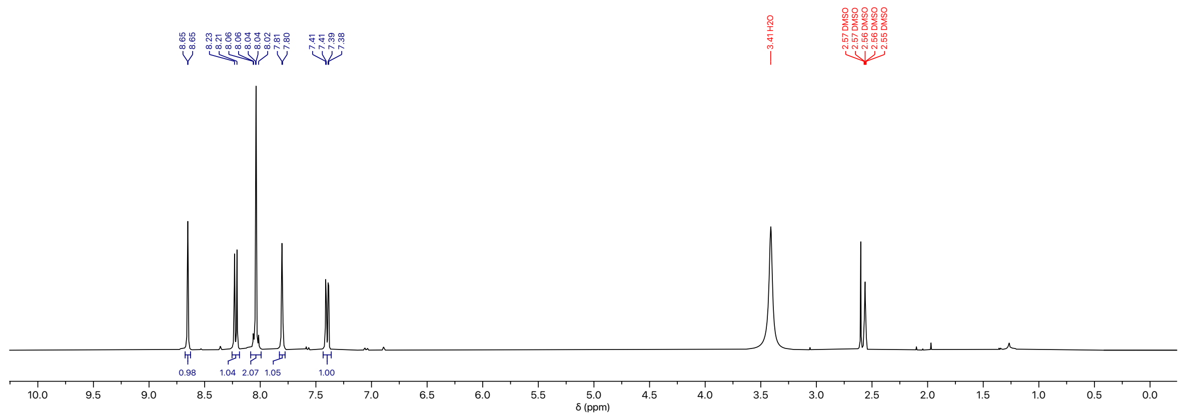

Figure S148. ^{1}H (400 MHz, DMSO-d^{6}, 298 K) NMR spectrum of compound **46**

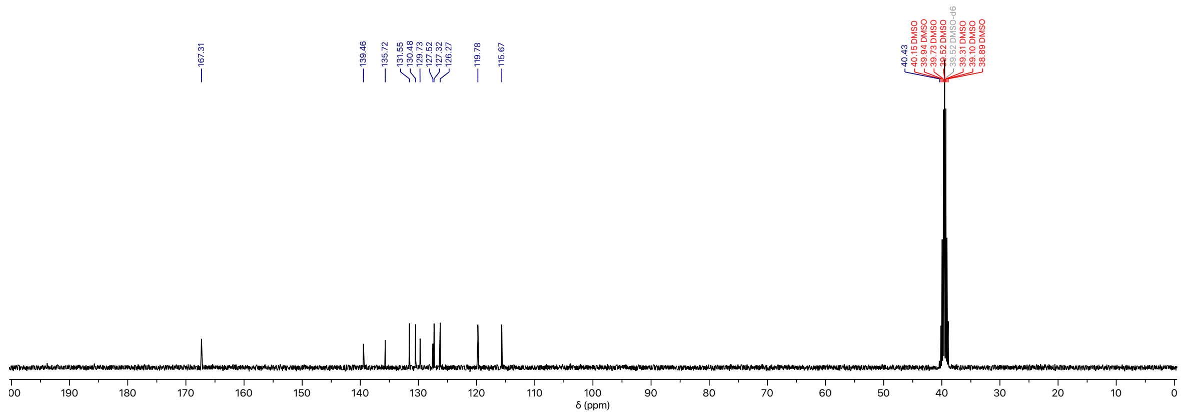

Figure S149. ^{13}C (101 MHz, DMSO-d^{6}, 298 K) NMR spectrum of compound **46**

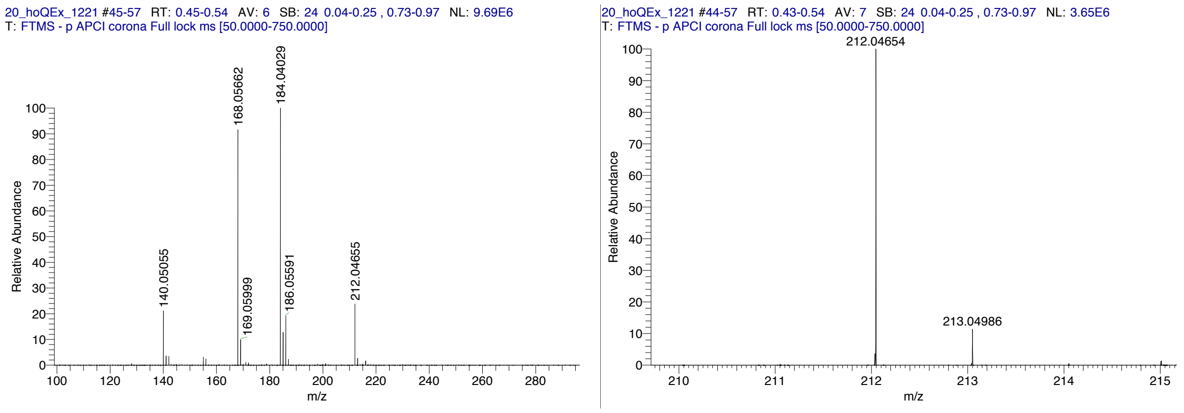

Figure S150. HR-ESI-MS spectrum of compound **46**

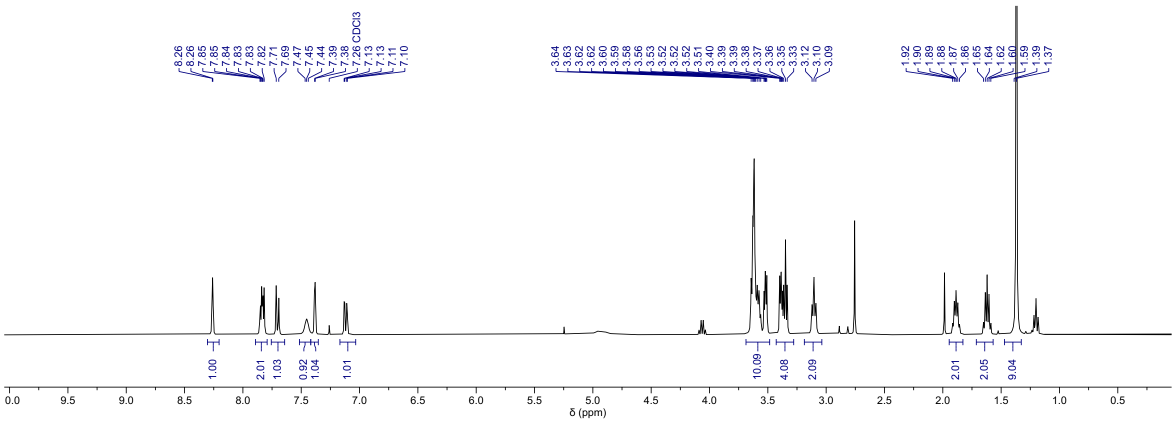

Figure S151. ^{1}H (400 MHz, $CDCl_3$, 298 K) NMR spectrum of compound **47**

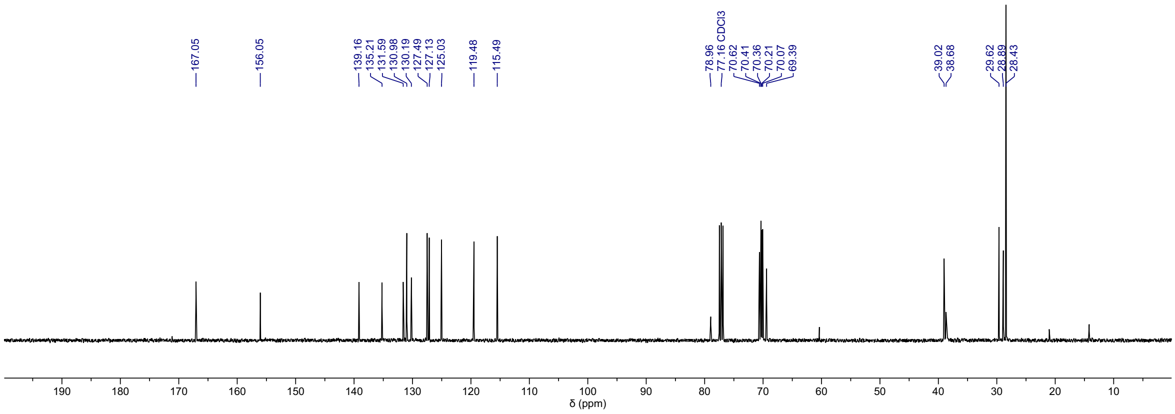

Figure S152. ^{13}C (101 MHz, $CDCl_3$, 298 K) NMR spectrum of compound **47**

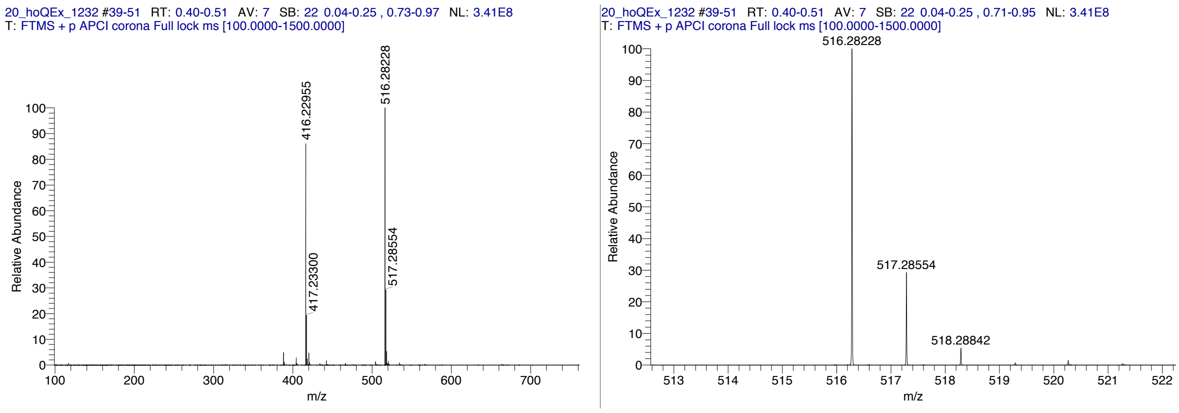

Figure S153. HR-ESI-MS spectrum of compound **47**

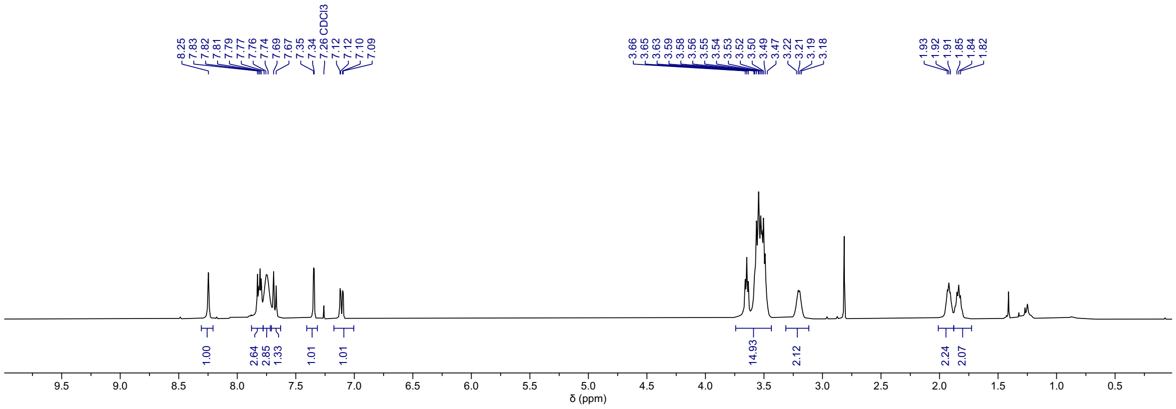

Figure S154. ^{1}H (400 MHz, $CDCl_3$, 298 K) NMR spectrum of compound **48**

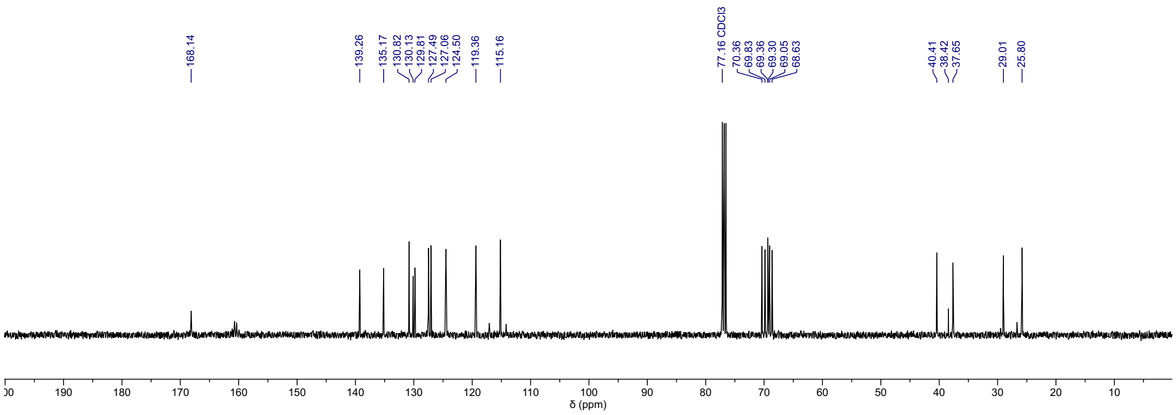

Figure S155. ^{13}C (101 MHz, $CDCl_3$, 298 K) NMR spectrum of compound **48**

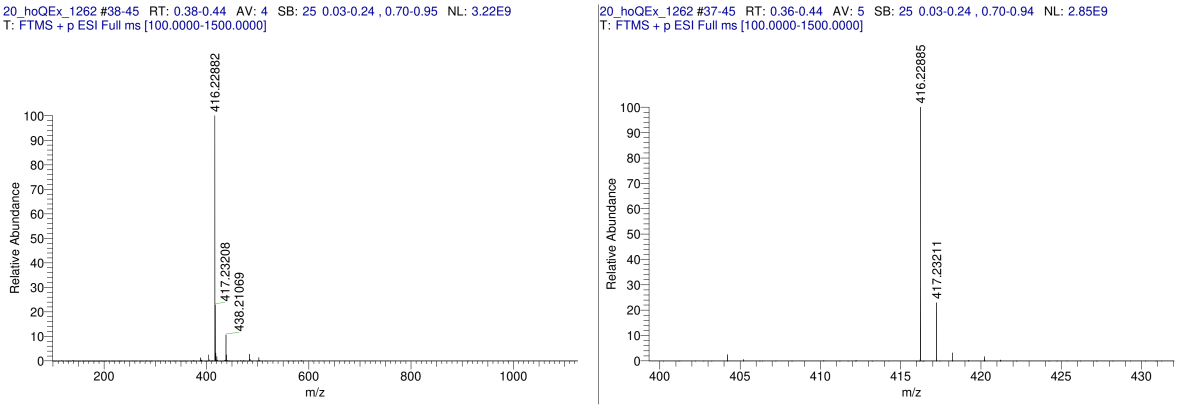

Figure S156. HR-ESI-MS spectrum of compound **48**

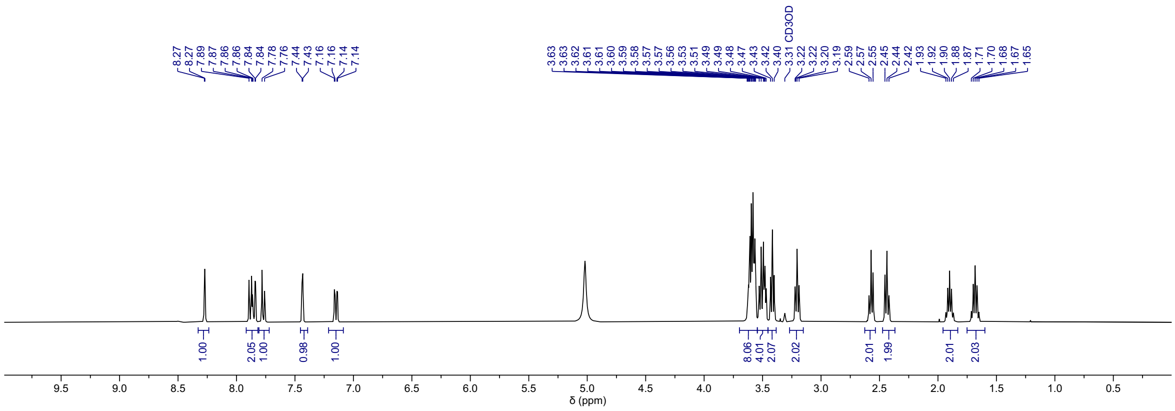

Figure S157. ^{1}H (400 MHz, MeOD-d^4, 298 K) NMR spectrum of compound **49**

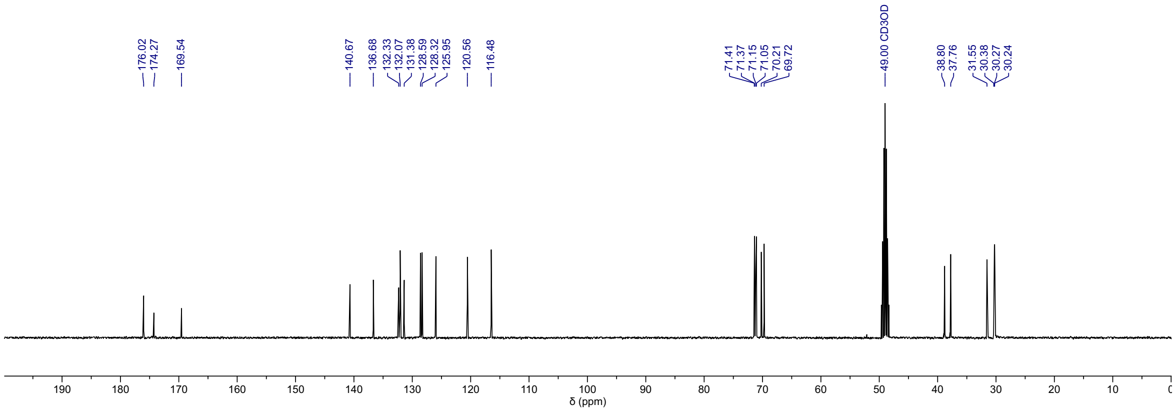

Figure S158. ^{13}C (101 MHz, MeOD-d^4, 298 K) NMR spectrum of compound **49**

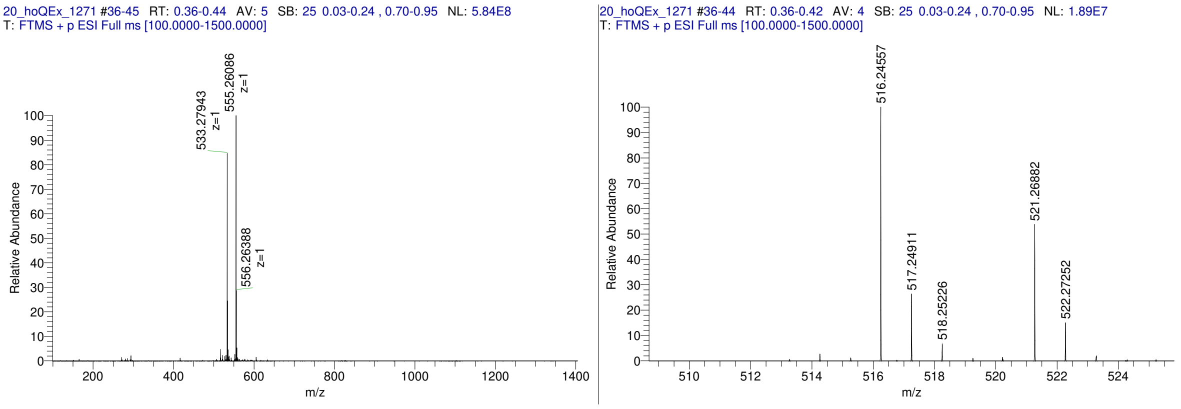

Figure S159. HR-ESI-MS spectrum of compound **49**

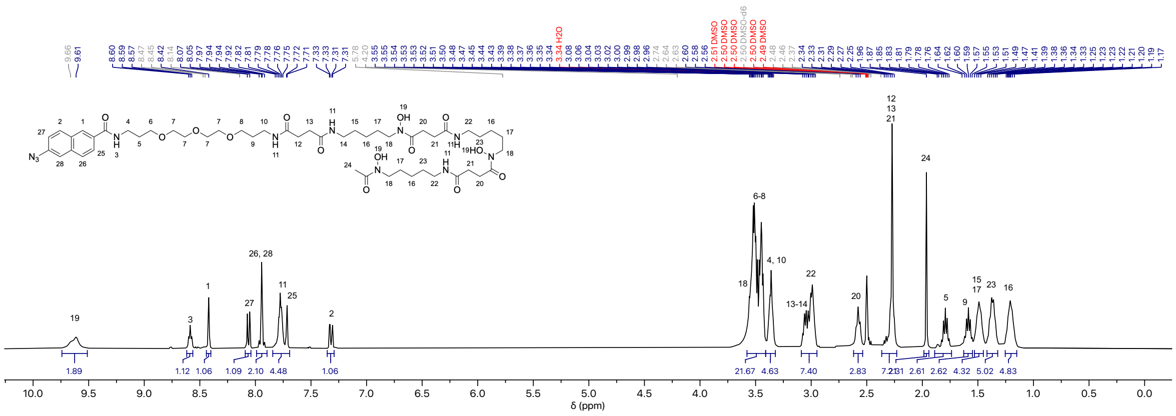

Figure S160. ^{1}H (400 MHz, DMSO-d^{6}, 298 K) NMR spectrum of compound **11**

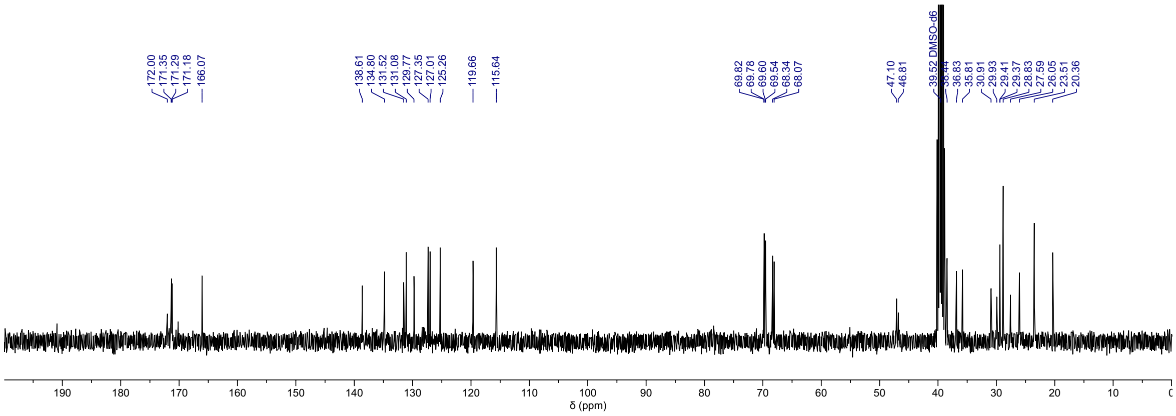

Figure S161. ^{13}C (101 MHz, DMSO-d^{6}, 298 K) NMR spectrum of compound **11**

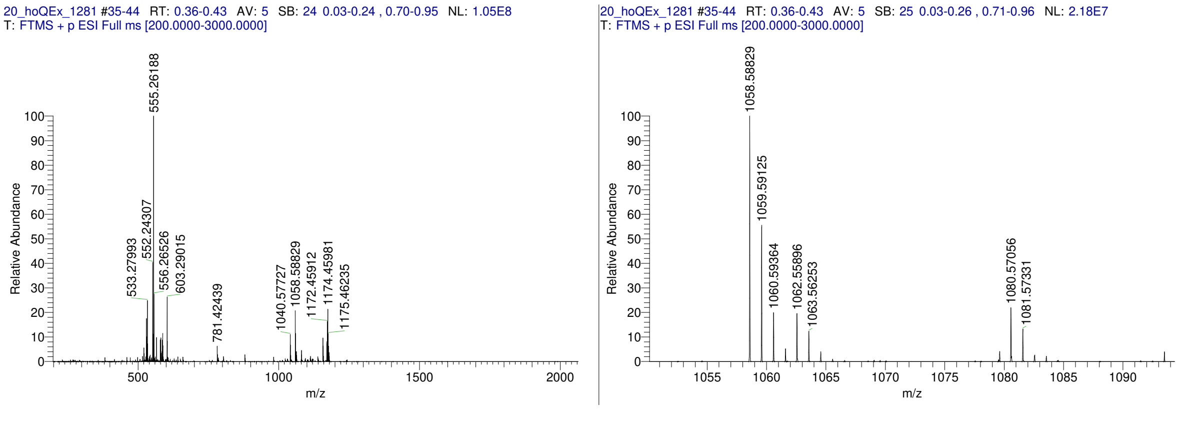

Figure S162. HR-ESI-MS spectrum of compound **11**

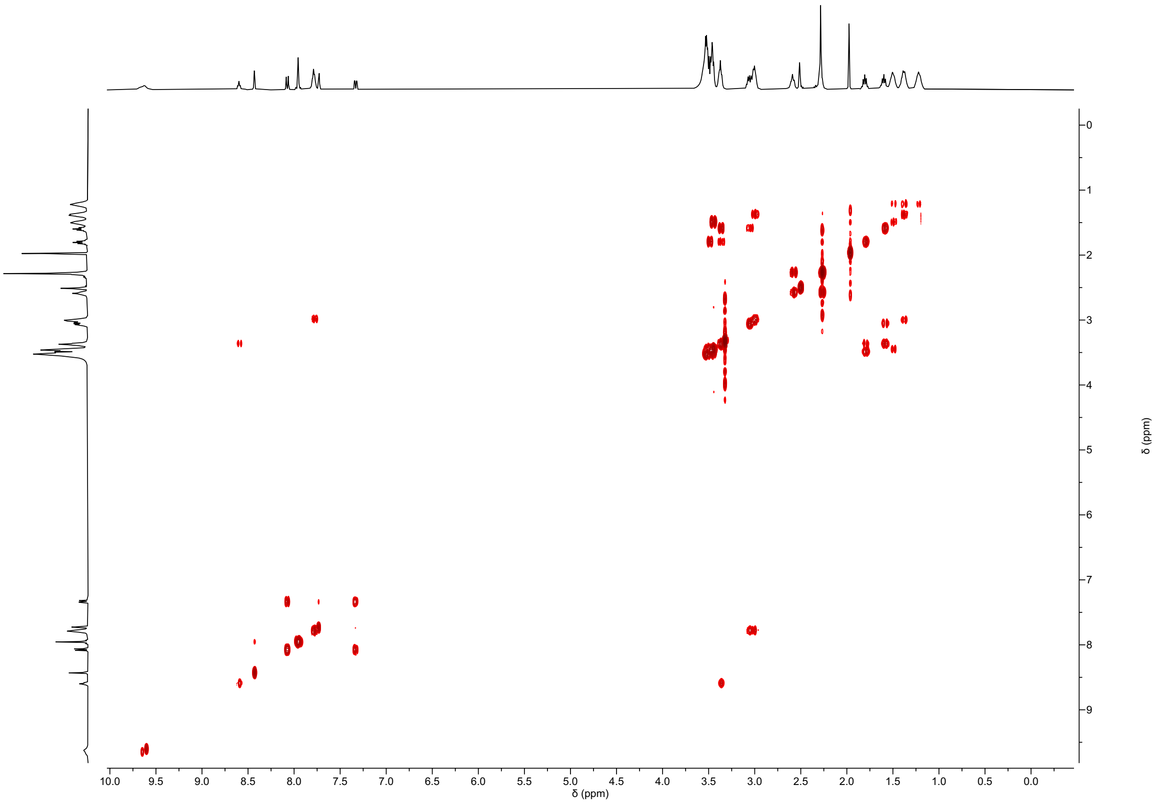

Figure S163. ^{1}H-^{1}H (101 MHz, DMSO-d^{6}, 298 K) 2D NMR spectrum of compound **11**

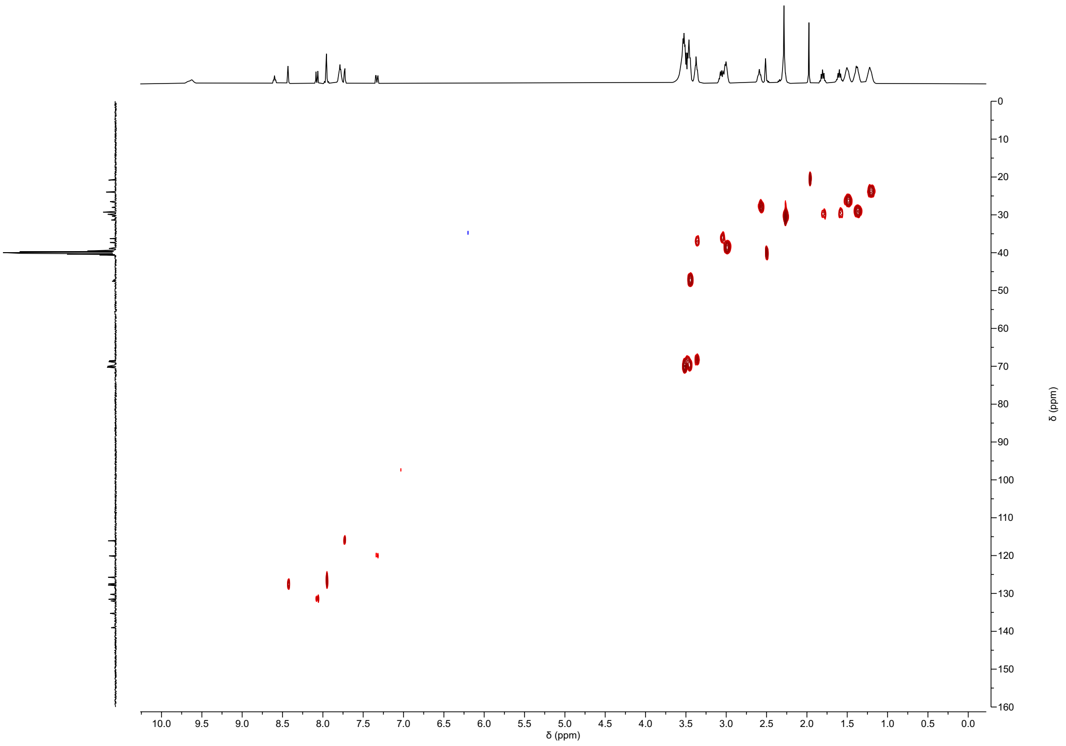

Figure S164. ^{1}H-^{13}C (101 MHz, DMSO-d^{6}, 298 K) 2D NMR spectrum of compound **11**

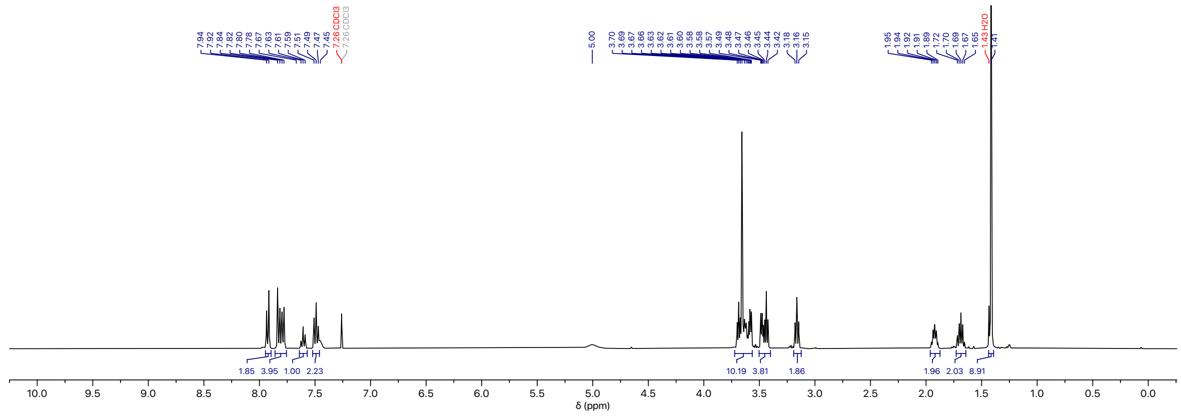

Figure S165. ^{1}H (400 MHz, $CDCl_3$, 298 K) NMR spectrum of compound **50**

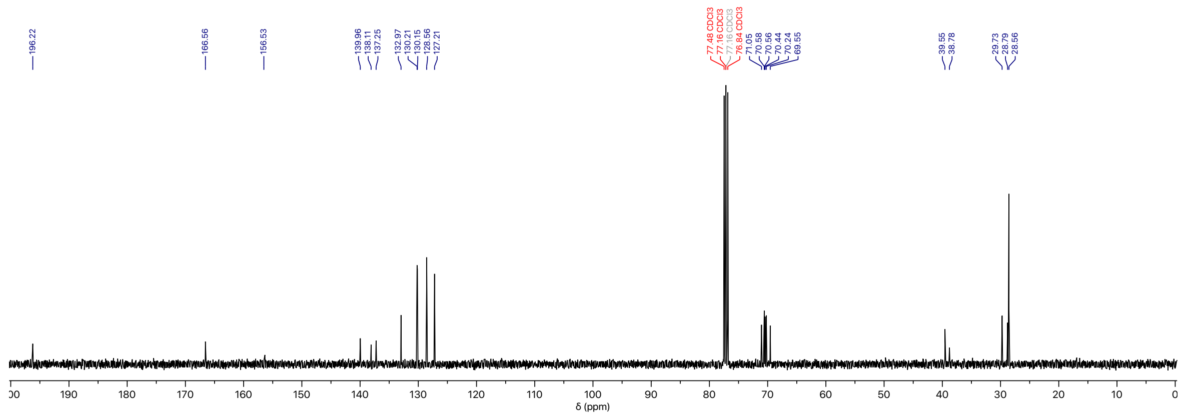

Figure S166. ^{13}C (101 MHz, $CDCl_3$, 298 K) NMR spectrum of compound **50**

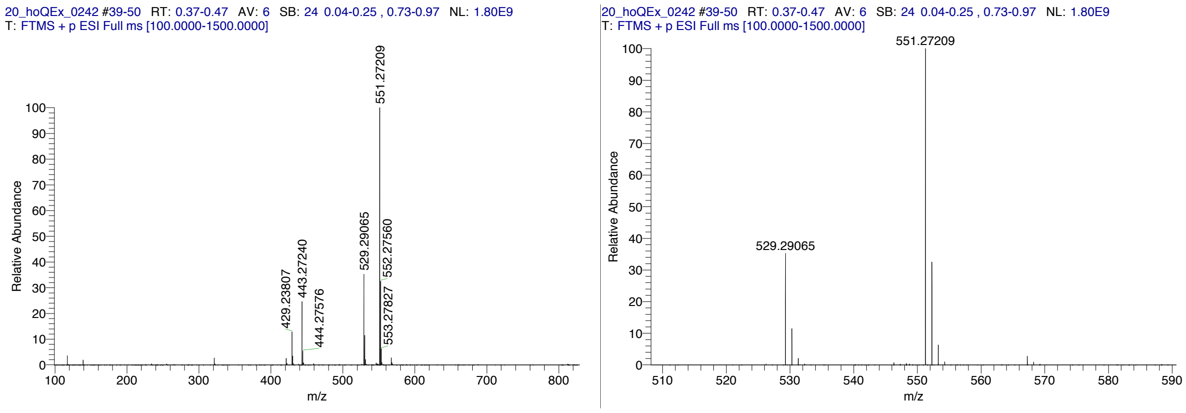

Figure S167. HR-ESI-MS spectrum of compound **50**

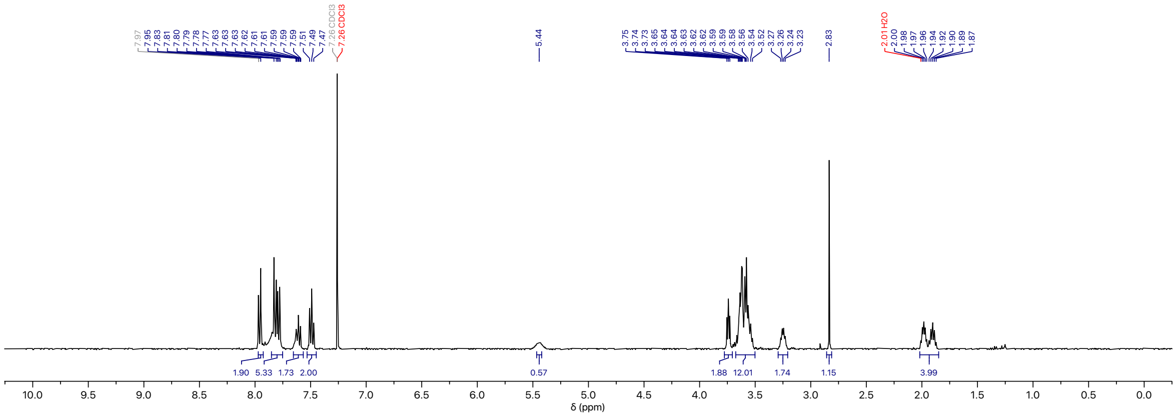

Figure S168. ^{1}H (400 MHz, $CDCl_3$, 298 K) NMR spectrum of compound **51**

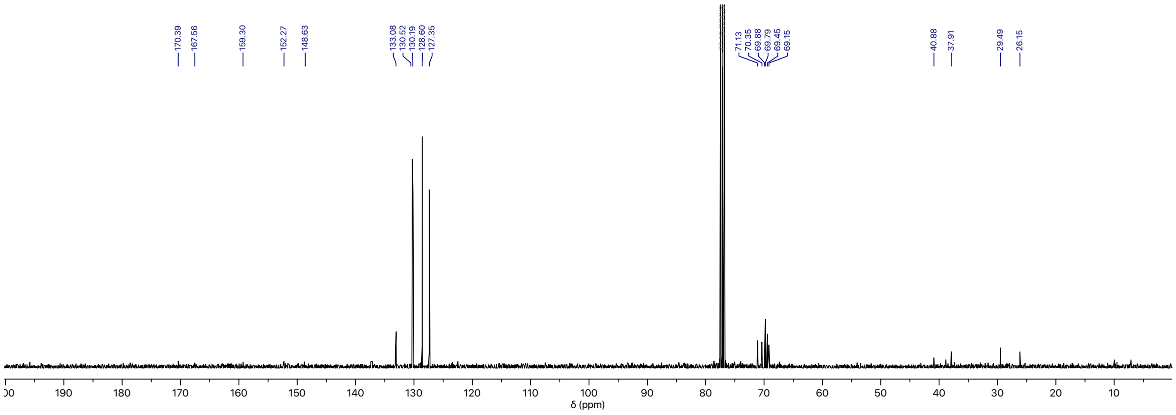

Figure S169. ^{13}C (101 MHz, $CDCl_3$, 298 K) NMR spectrum of compound **51**

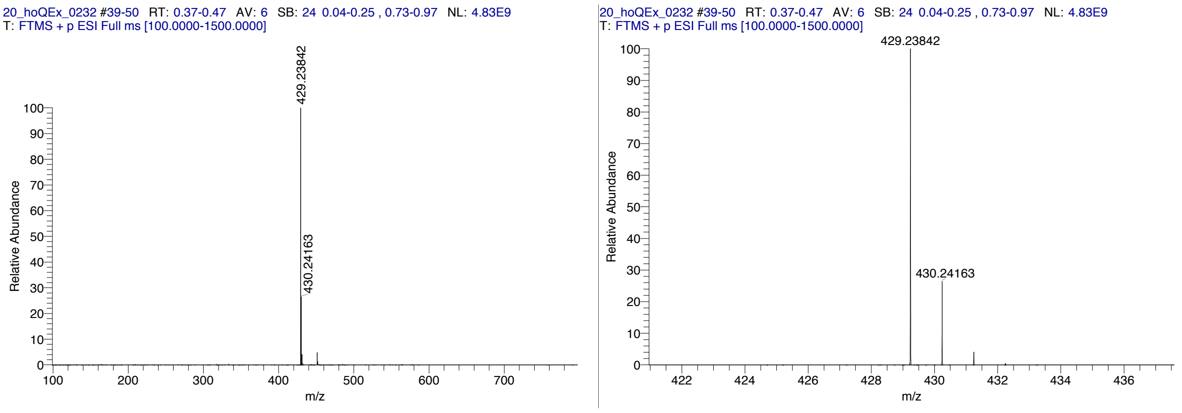

Figure S170. HR-ESI-MS spectrum of compound **51**

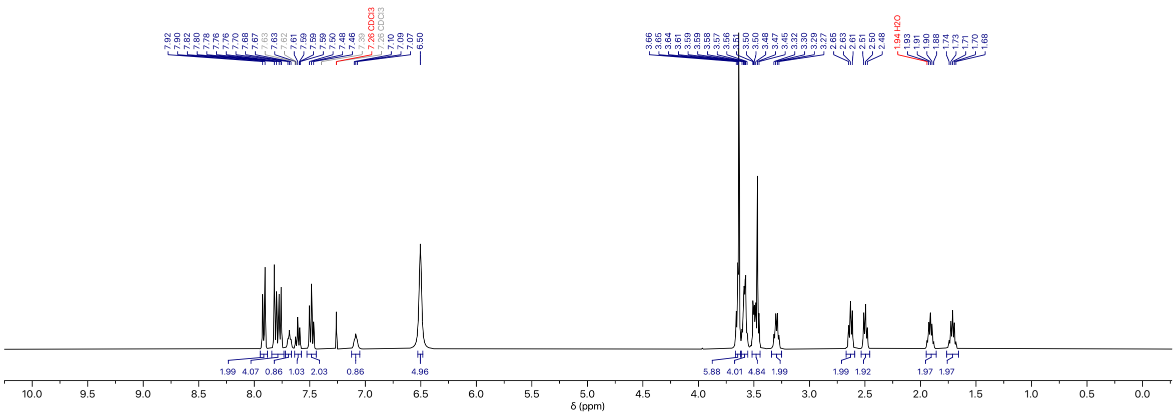

Figure S171. ^{1}H (400 MHz, $CDCl_3$, 298 K) NMR spectrum of compound **52**

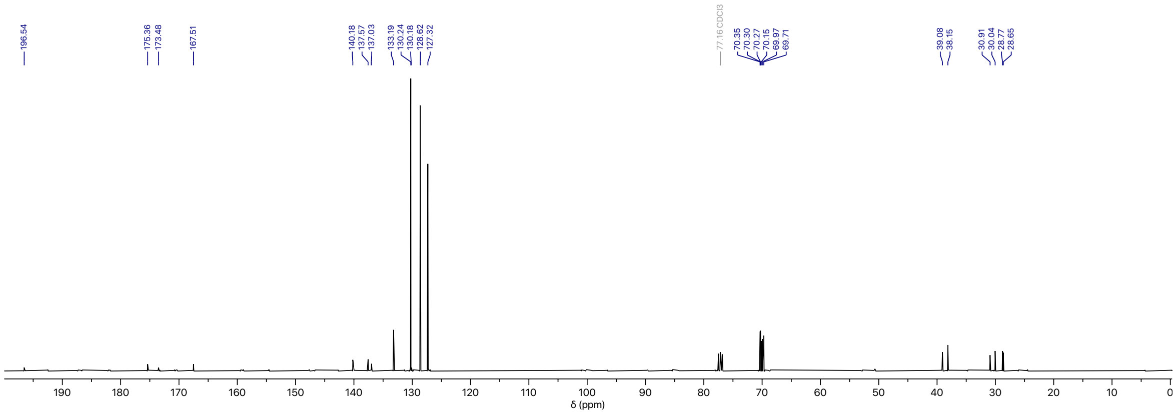

Figure S172. ^{13}C (101 MHz, $CDCl_3$, 298 K) NMR spectrum of compound **52**

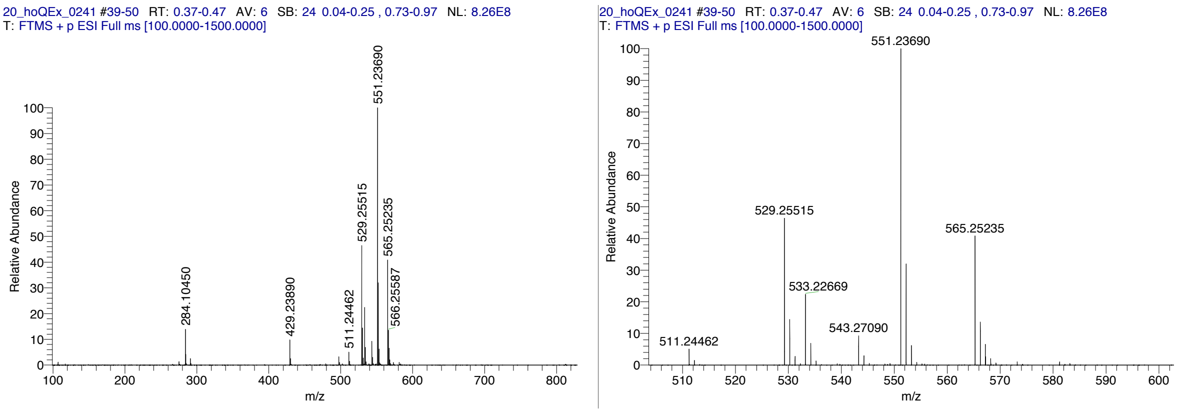

Figure S173. HR-ESI-MS spectrum of compound **52**

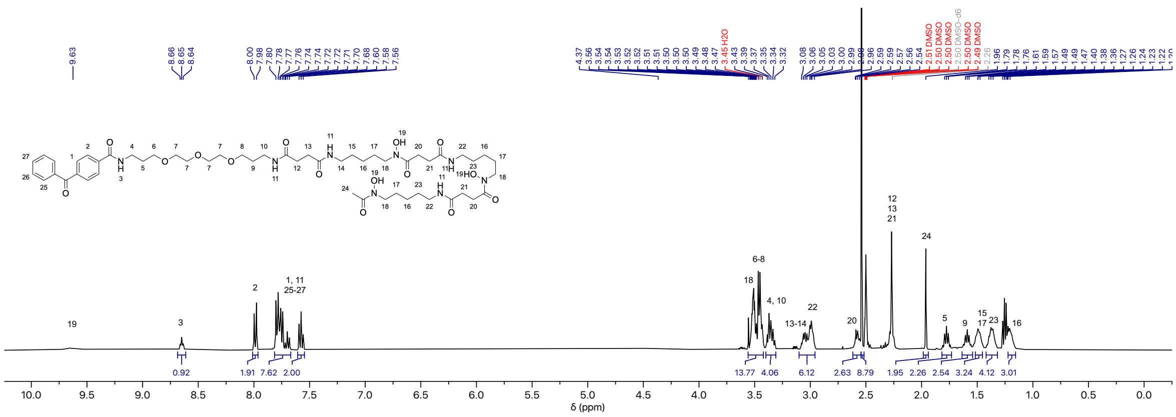

Figure S174. ^{1}H (400 MHz, DMSO-d^{6}, 298 K) NMR spectrum of compound **12**

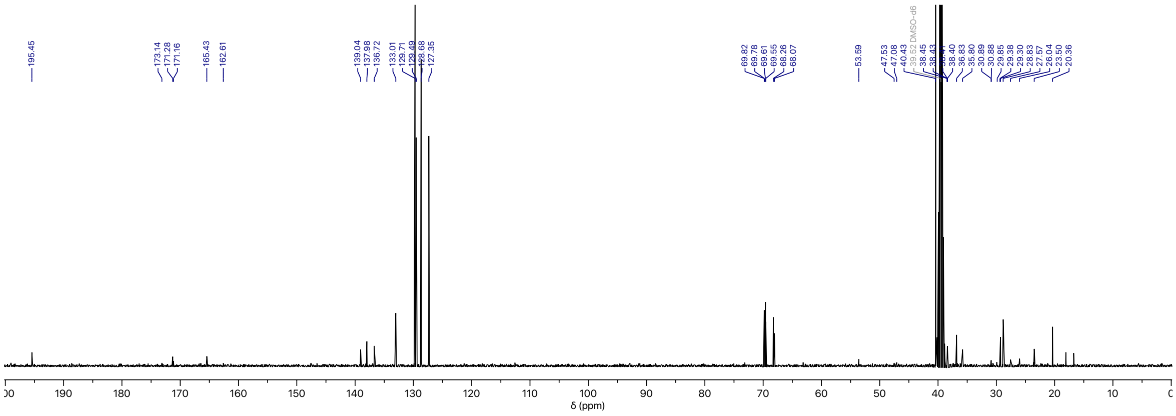

Figure S175. ^{13}C (101 MHz, DMSO-d^{6}, 298 K) NMR spectrum of compound **12**

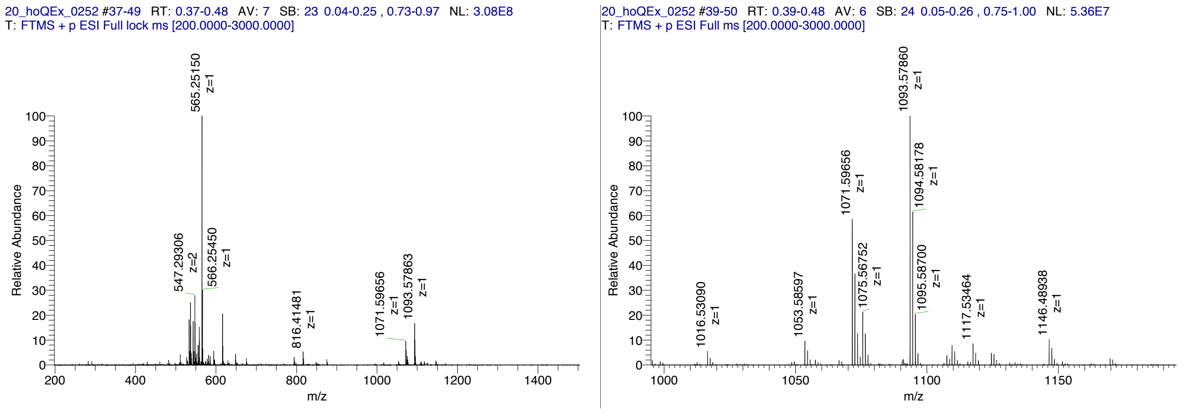

Figure S176. HR-ESI-MS spectrum of compound **12**

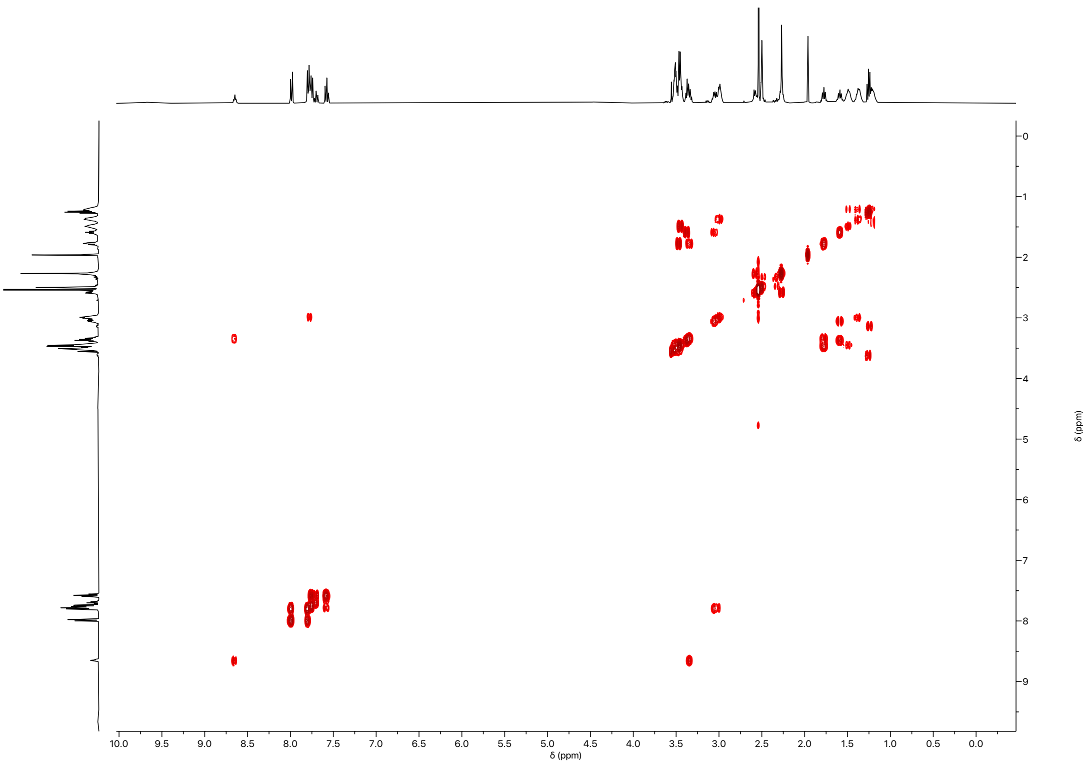

Figure S177. ^{1}H-^{1}H (COSY, DMSO-d^6, 298 K) 2D NMR spectrum of compound **12**

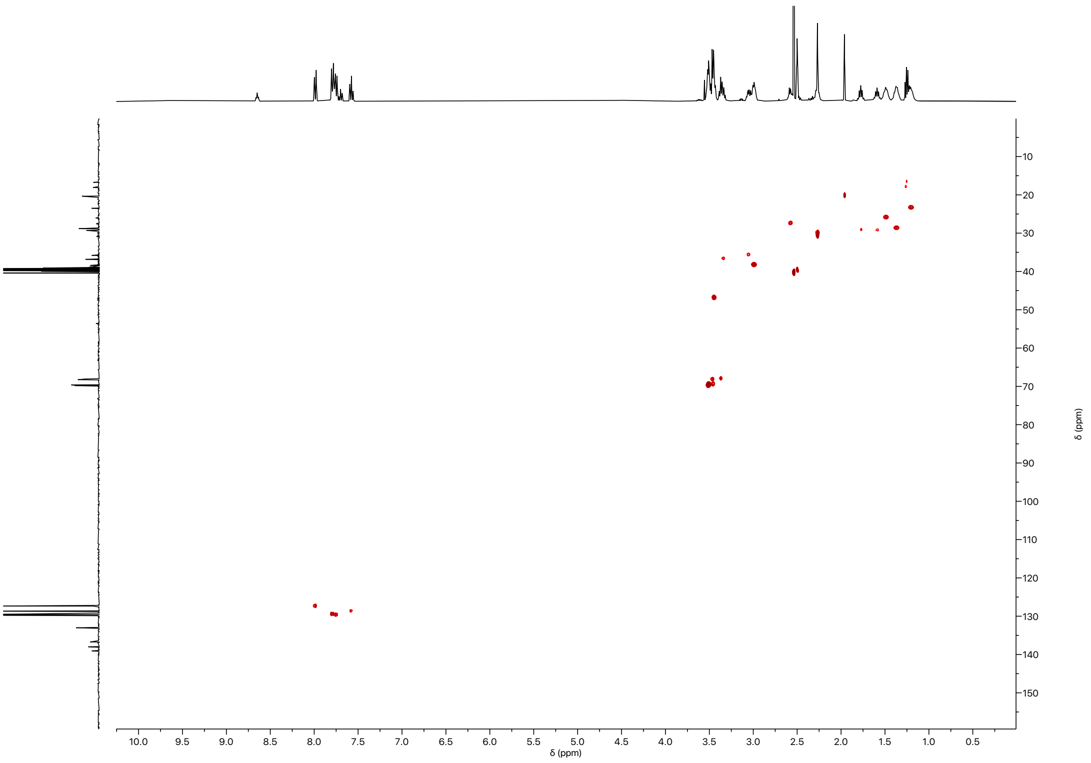

Figure S178. ^{1}H-^{13}C (HSQC, DMSO-d^6, 298 K) 2D NMR spectrum of compound **12**

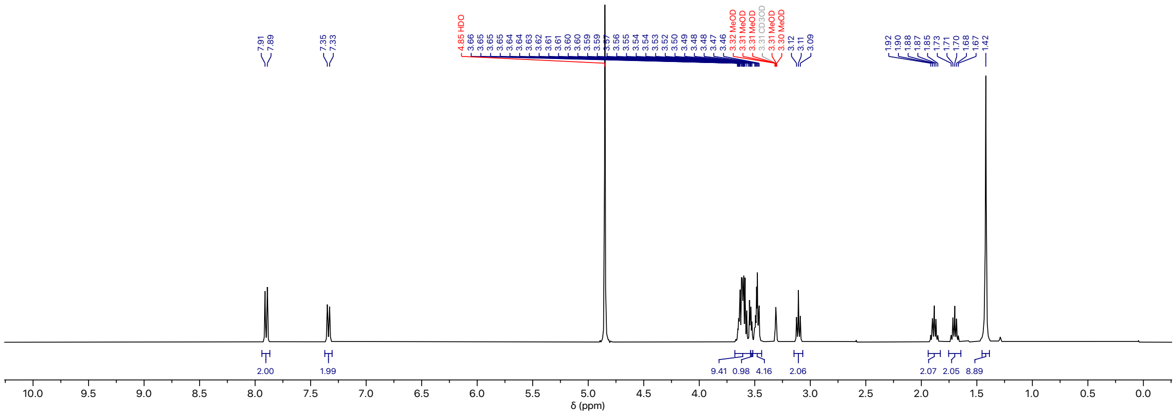

Figure S179. ^{1}H (400 MHz, MeOD-d^{4}, 298 K) NMR spectrum of compound **53**

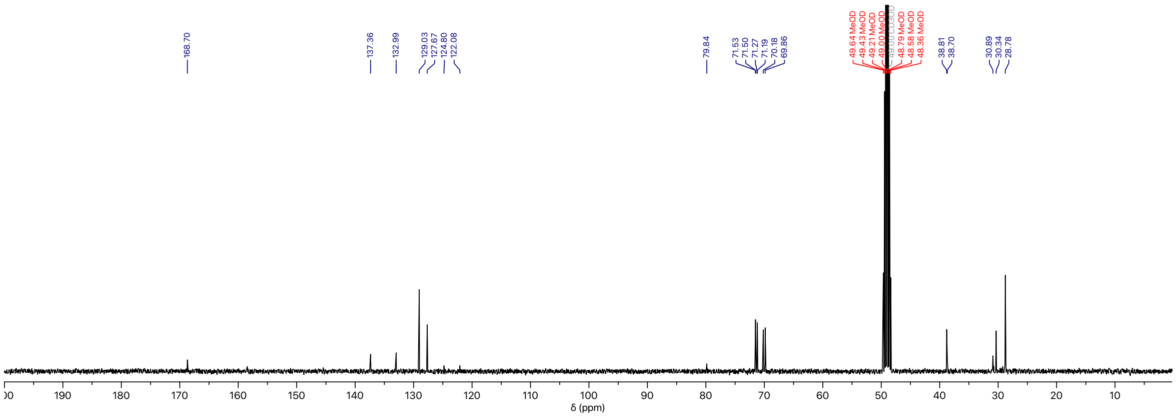

Figure S180. ^{13}C (101 MHz, MeOD-d^{4}, 298 K) NMR spectrum of compound **53**

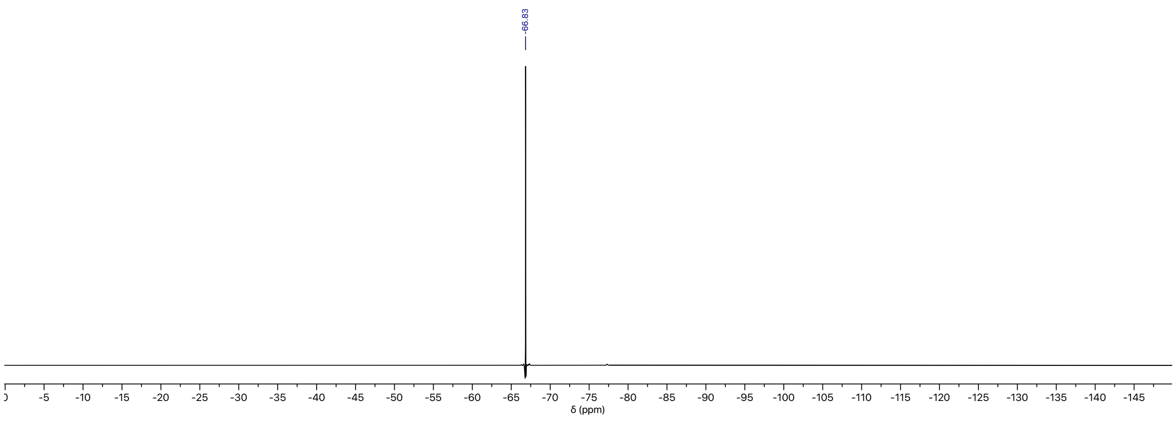

Figure S181. ^{19}F (376 MHz, MeOD-d^{4}, 298 K) NMR spectrum of compound **53**

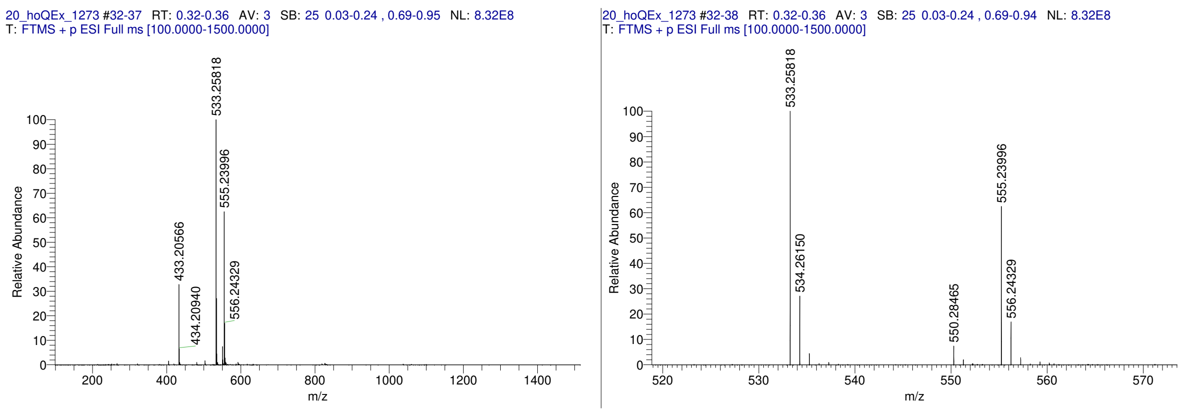

Figure S182. HR-ESI-MS spectrum of compound **53**

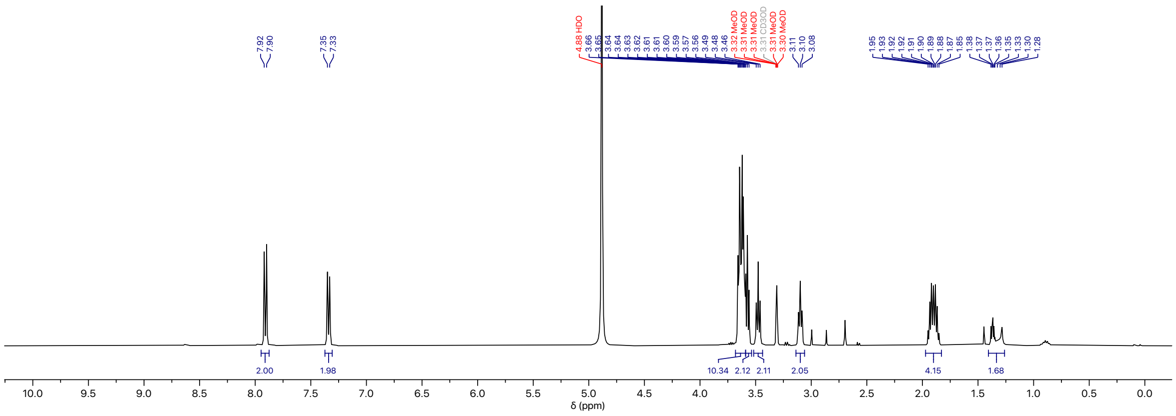

Figure S183. ^{1}H (400 MHz, MeOD-d^{4}, 298 K) NMR spectrum of compound **54**

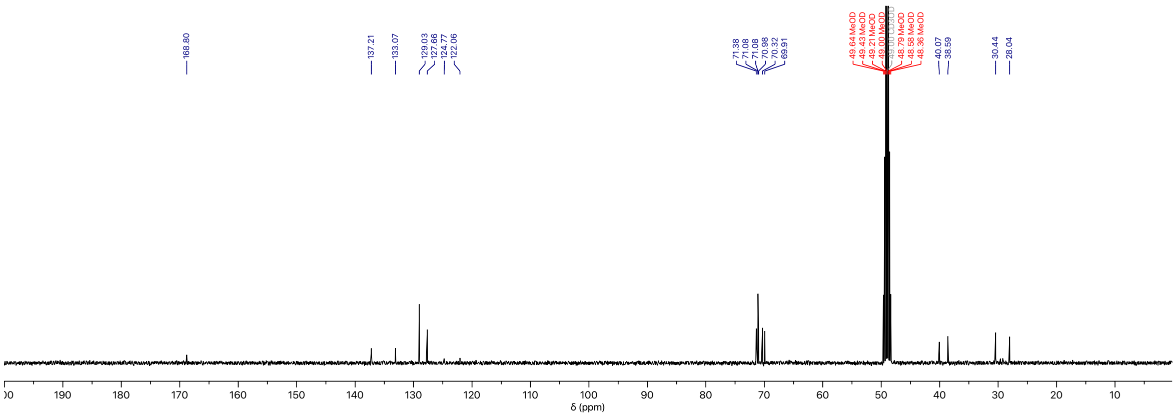

Figure S184. ^{13}C (101 MHz, MeOD-d^{4}, 298 K) NMR spectrum of compound **54**

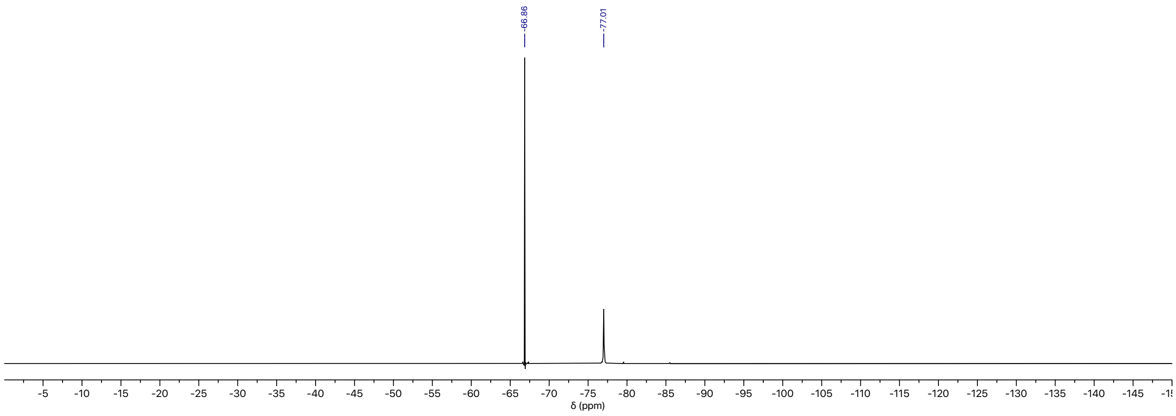

Figure S185. ^{19}F (376 MHz, MeOD-d^4, 298 K) NMR spectrum of compound **54**

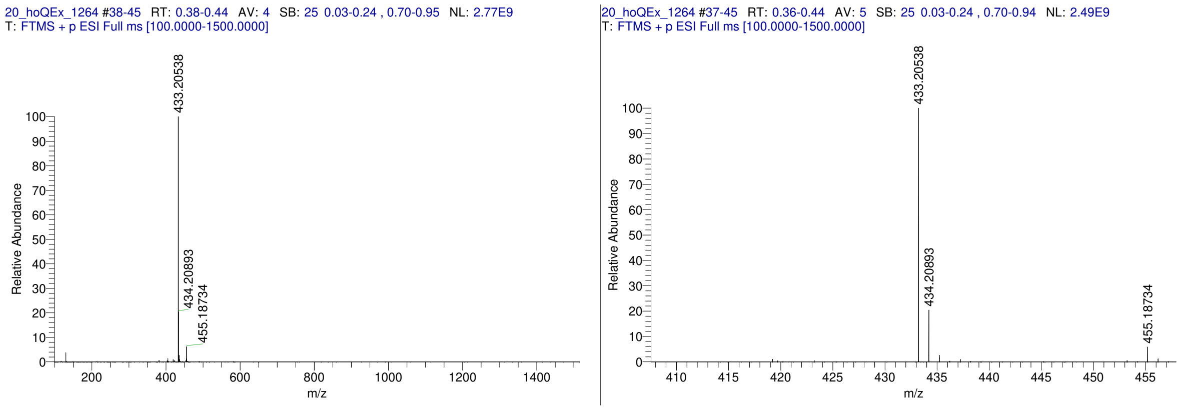

Figure S186. HR-ESI-MS spectrum of compound **54**

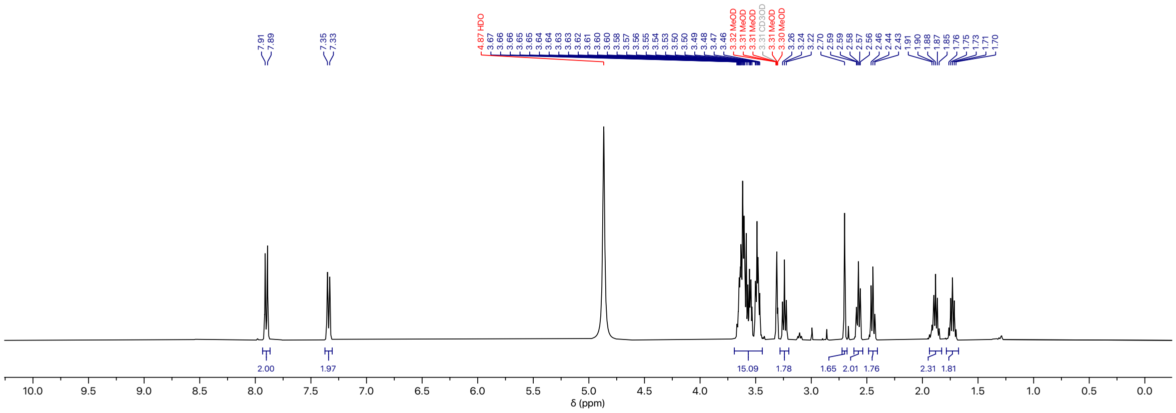

Figure S187. ^{1}H (400 MHz, MeOD-d^4, 298 K) NMR spectrum of compound **55**

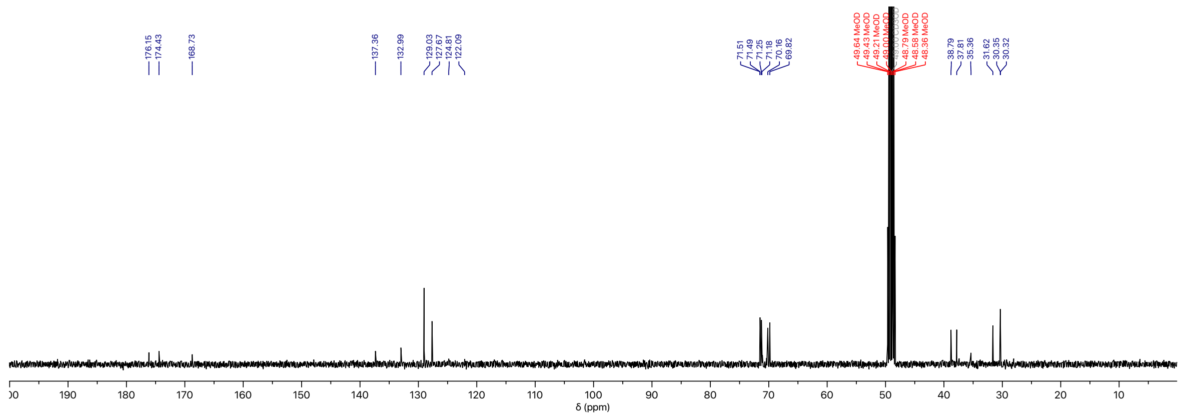

Figure S188. ^{13}C (101 MHz, MeOD-d^4, 298 K) NMR spectrum of compound **55**

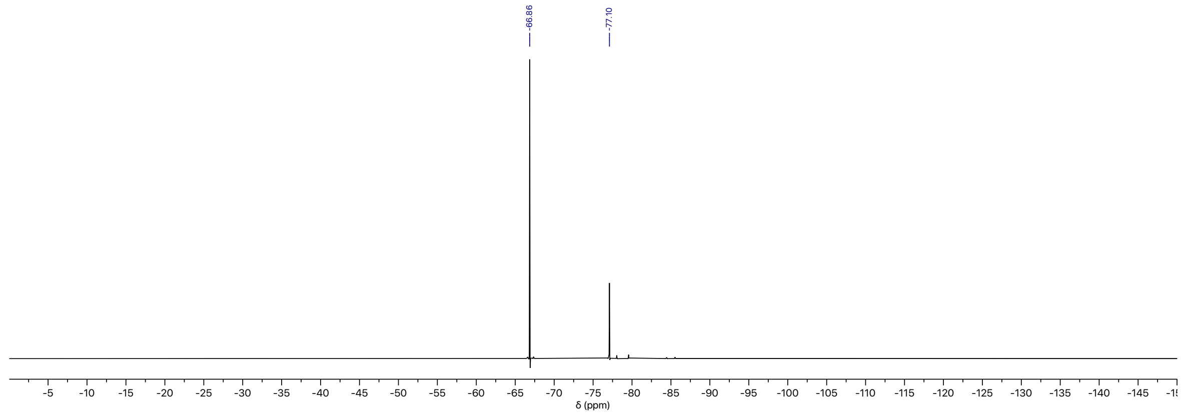

Figure S189. ^{19}F (376 MHz, MeOD-d^4, 298 K) NMR spectrum of compound **55**

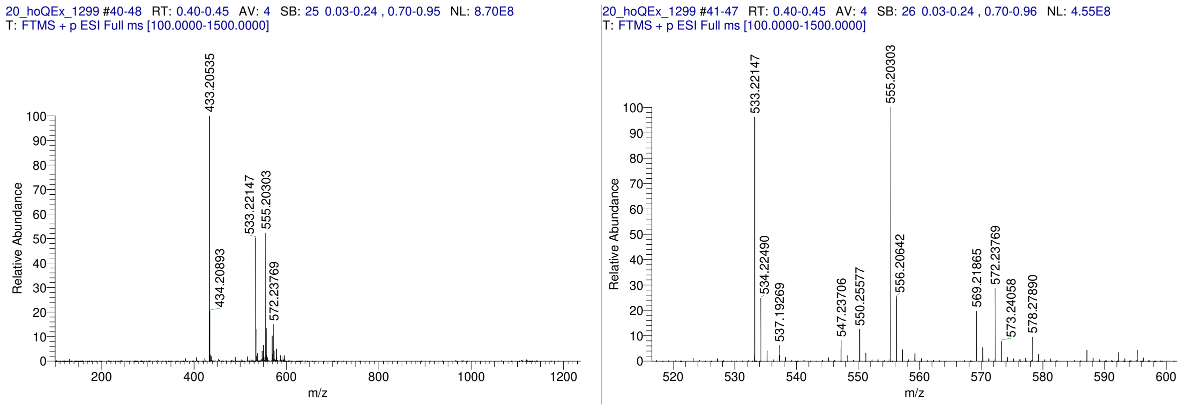

Figure S190. HR-ESI-MS spectrum of compound **55**

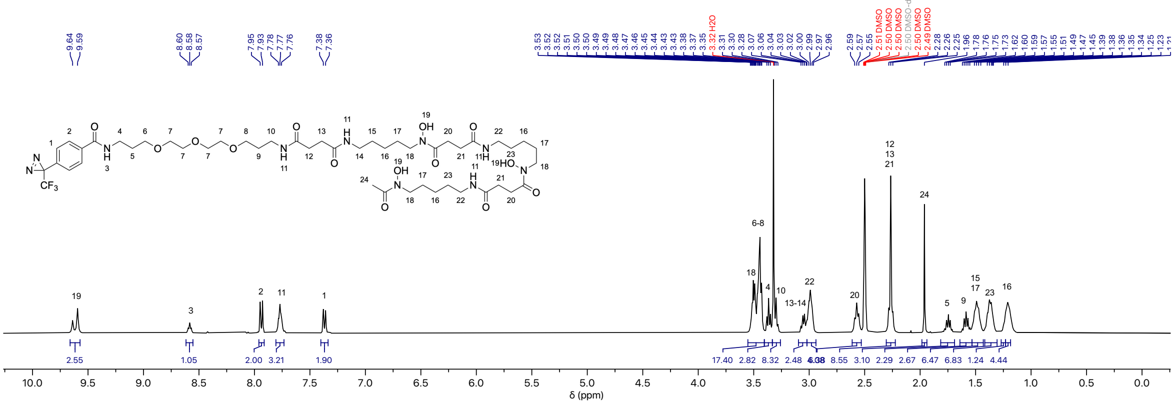

Figure S191. ^{1}H (400 MHz, DMSO-d[6], 298 K) NMR spectrum of compound **13**

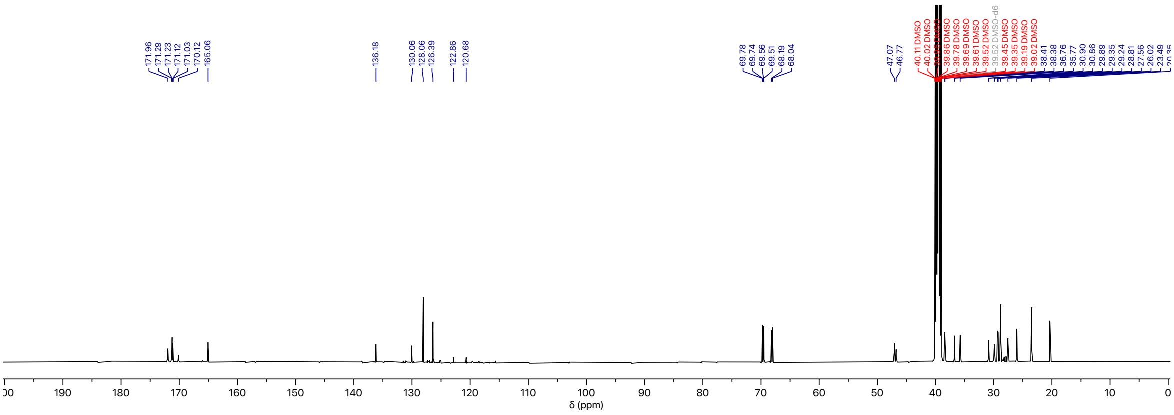

Figure S192. ^{13}C (126 MHz, DMSO-d[6], 298 K) NMR spectrum of compound **13**

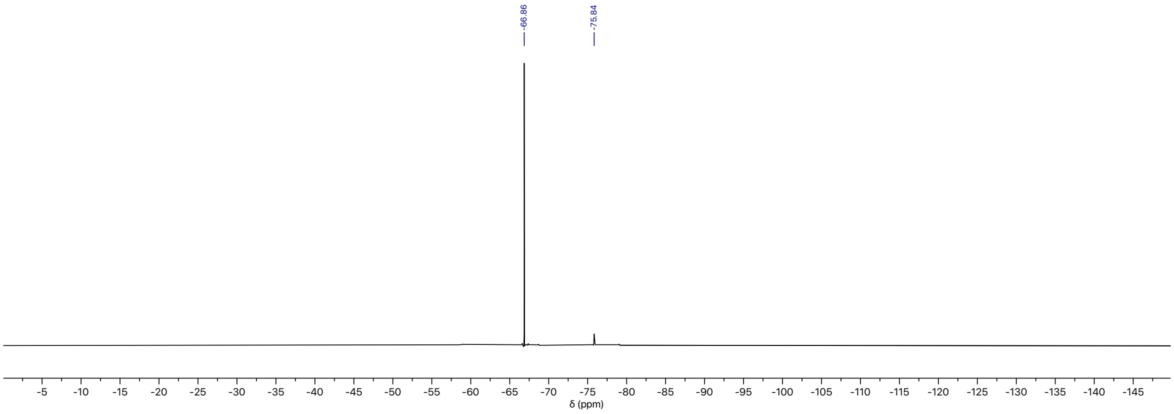

Figure S193. ^{19}F (376 MHz, DMSO-d[6], 298 K) NMR spectrum of compound **13**

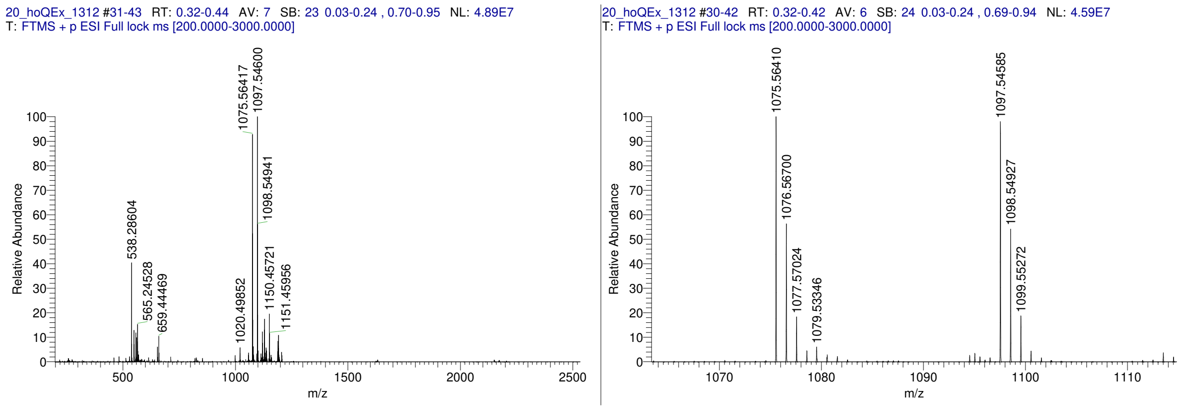

Figure S194. HR-ESI-MS spectrum of compound **13**

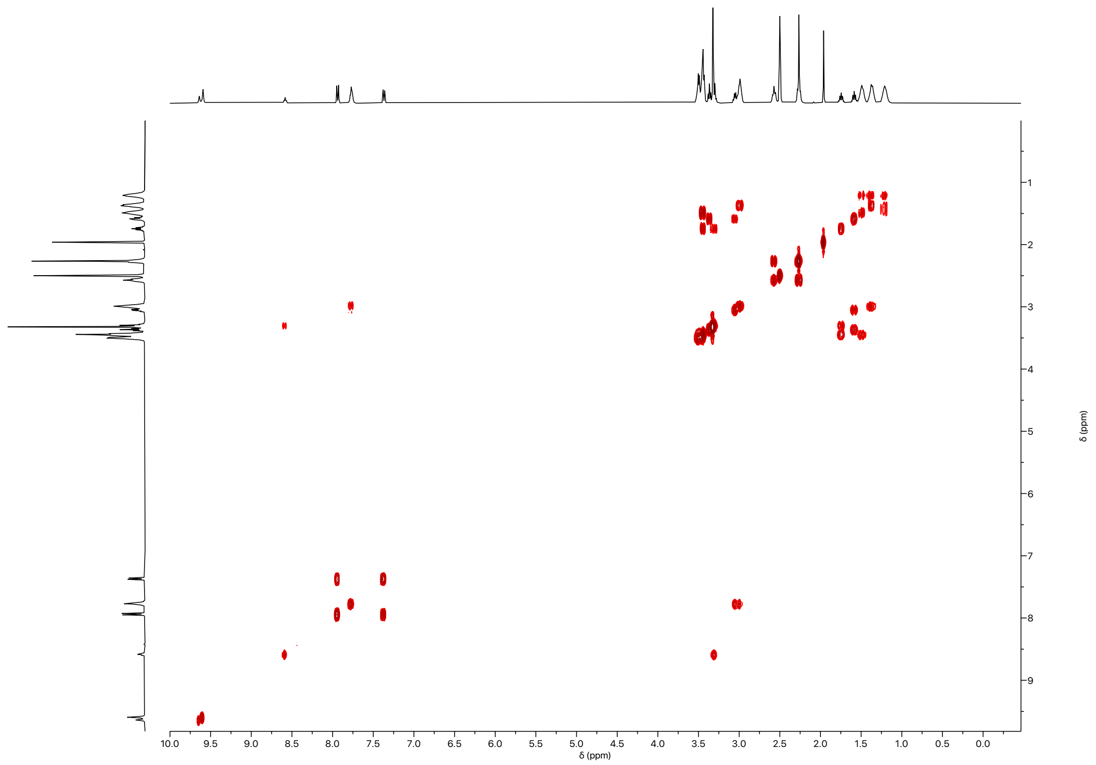

Figure S195. ^{1}H-^{1}H (COSY, DMSO-d^{6}, 298 K) 2D NMR spectrum of compound **13**

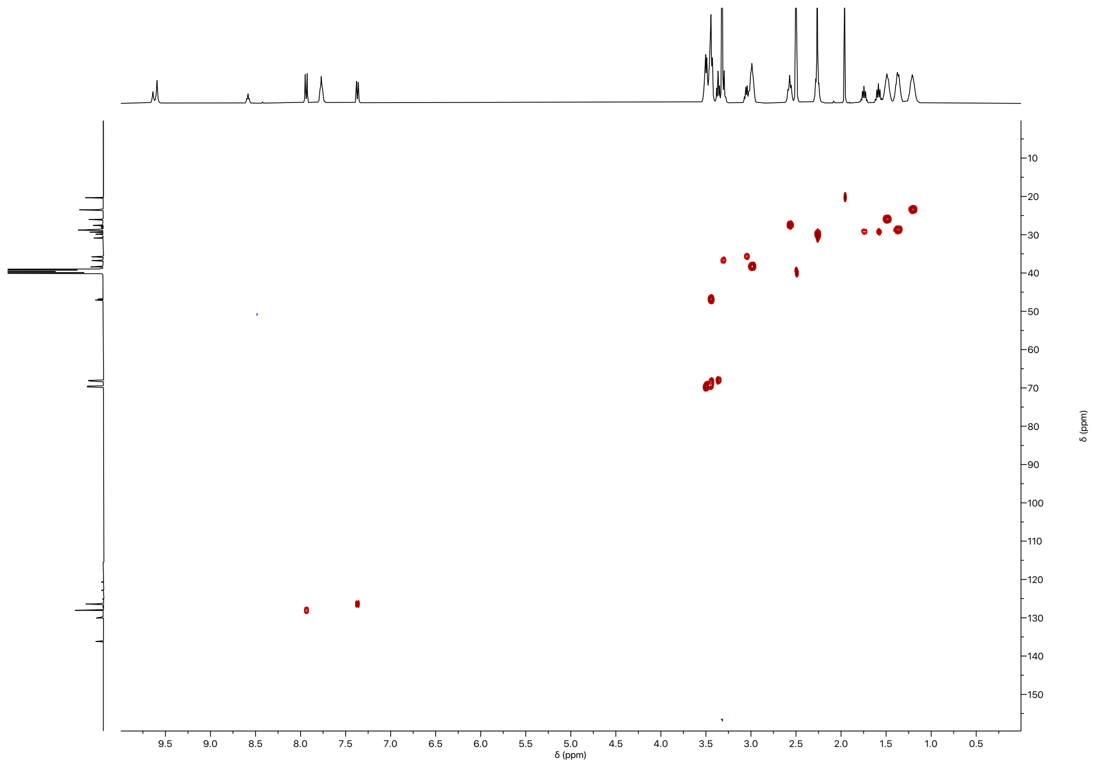

Figure S196. ^{1}H-^{13}C (HSQC, DMSO-d^{6}, 298 K) 2D NMR spectrum of compound **13**

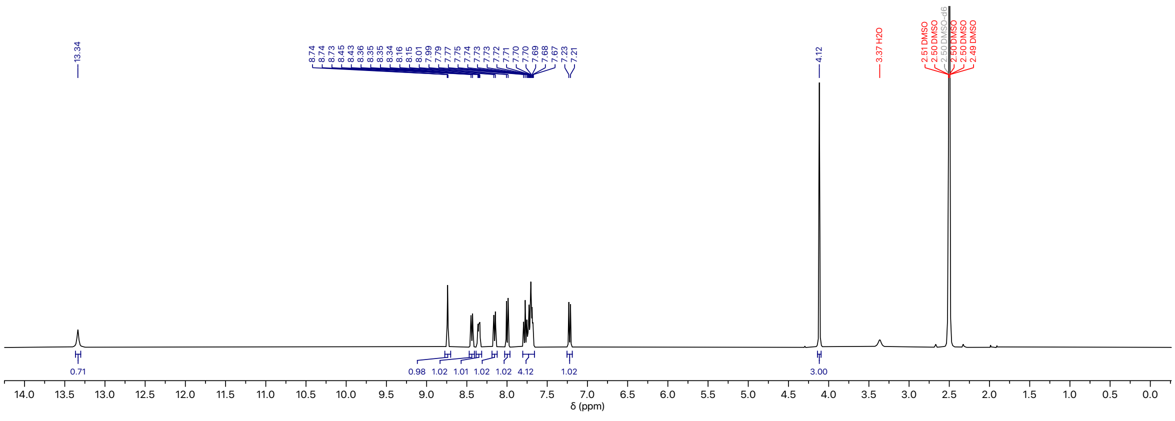

Figure S197. ^{1}H (400 MHz, DMSO-d^{6}, 298 K) NMR spectrum of compound **56**

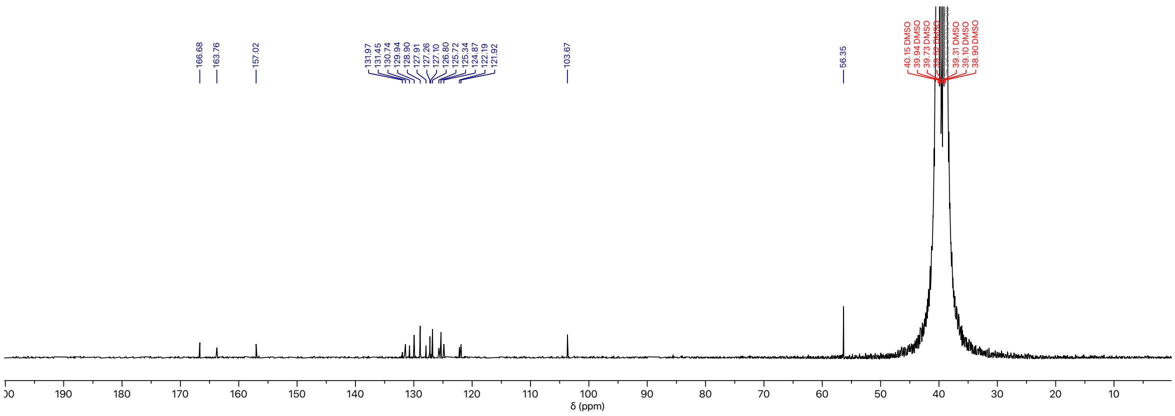

Figure S198. ^{13}C (101 MHz, DMSO-d^{6}, 298 K) NMR spectrum of compound **56**

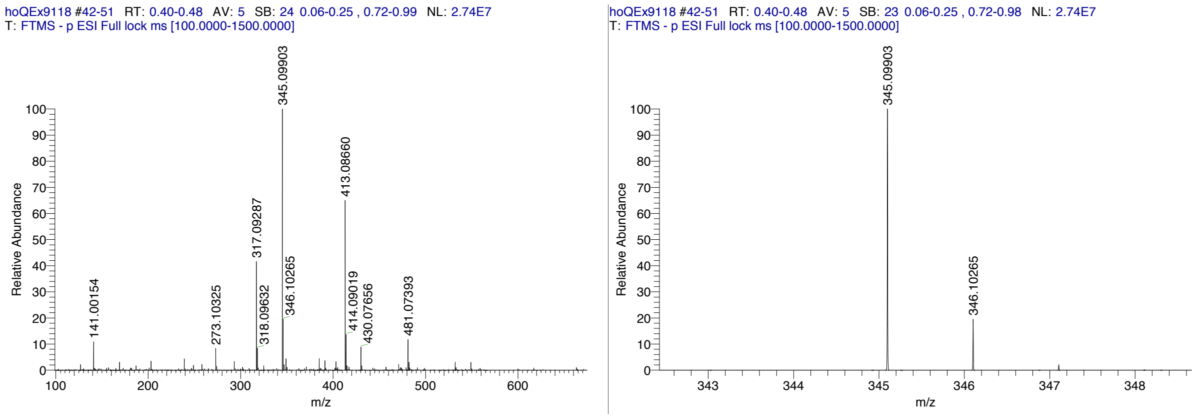

Figure S199. HR-ESI-MS spectrum of compound **56**

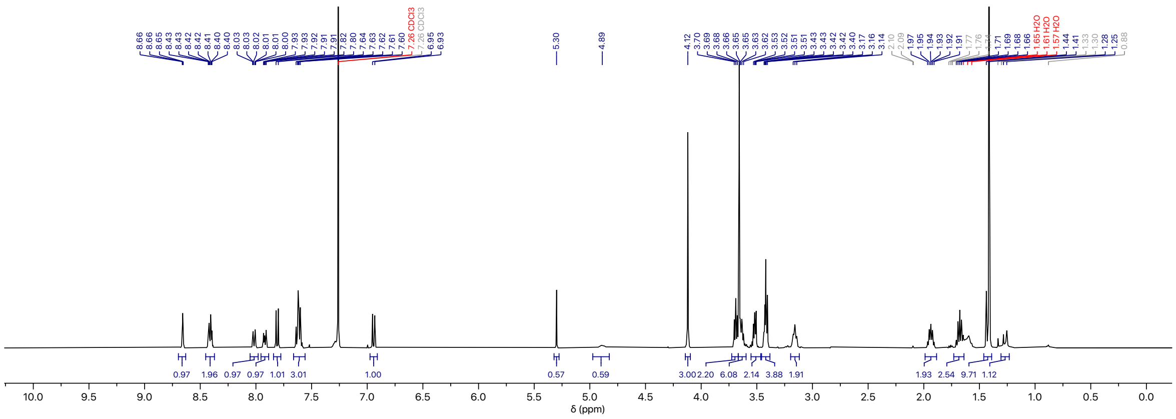

Figure S200. ^{1}H (400 MHz, $CDCl_3$, 298 K) NMR spectrum of compound **57**

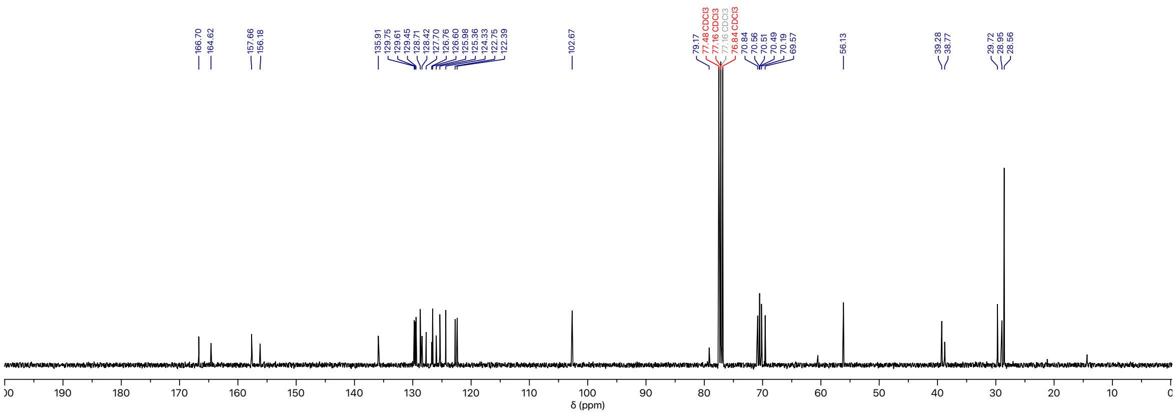

Figure S201. ^{13}C (101 MHz, $CDCl_3$, 298 K) NMR spectrum of compound **57**

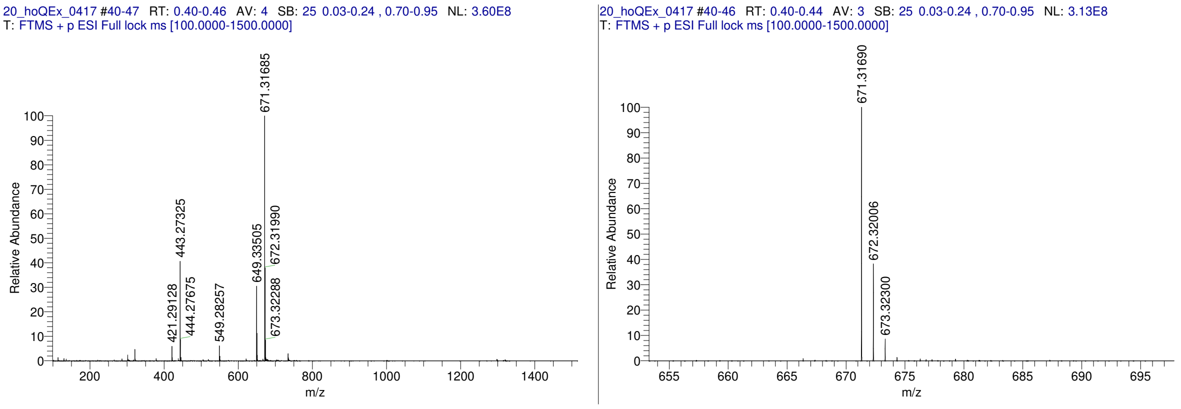

Figure S202. HR-ESI-MS spectrum of compound **57**

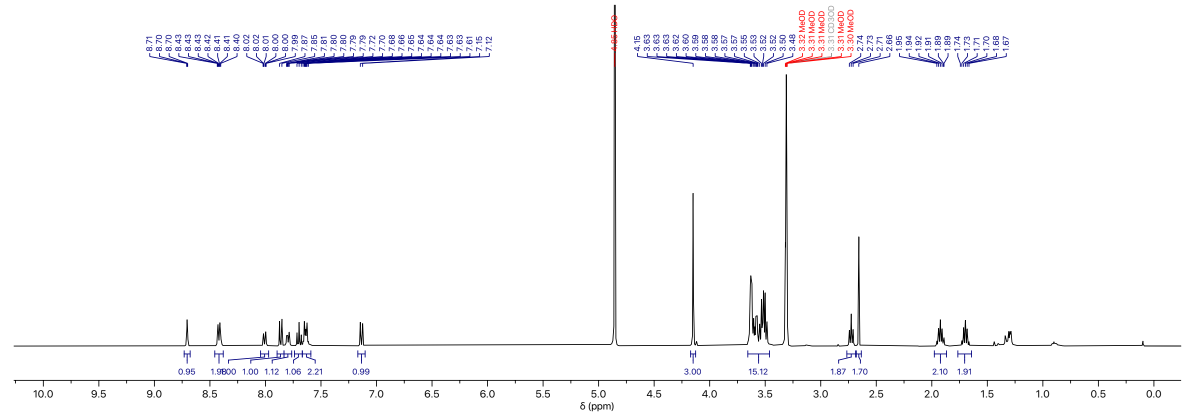

Figure S203. ^{1}H (400 MHz, MeOD-d^4, 298 K) NMR spectrum of compound **58**

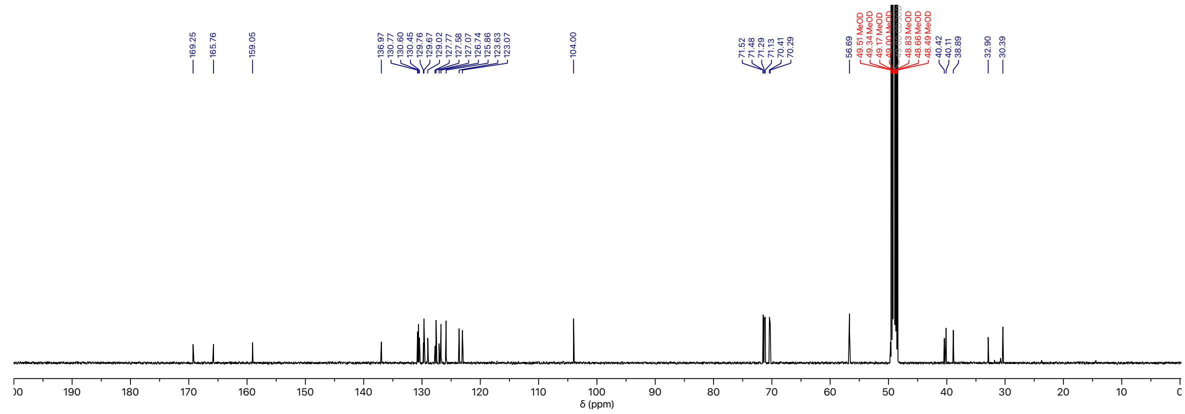

Figure S204. ^{13}C (126 MHz, $CDCl_3$, 298 K) NMR spectrum of compound **58**

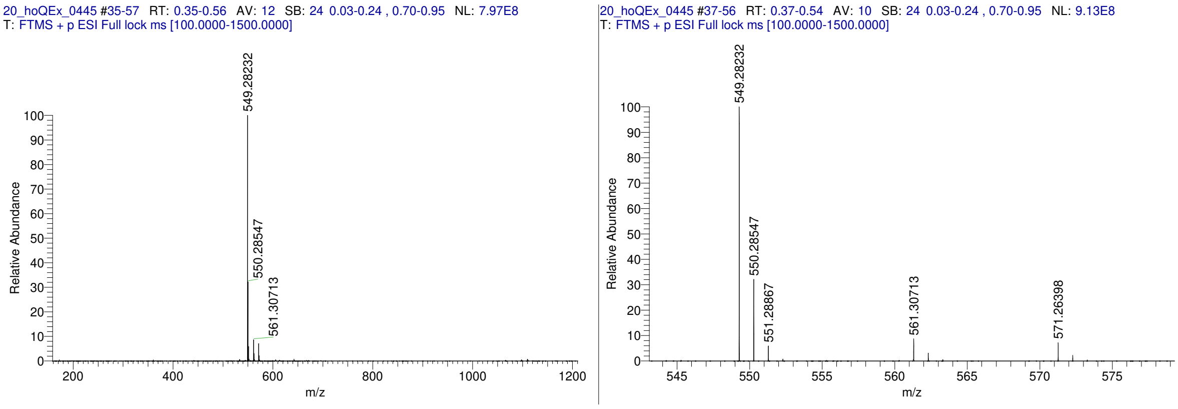

Figure S205. HR-ESI-MS spectrum of compound **58**

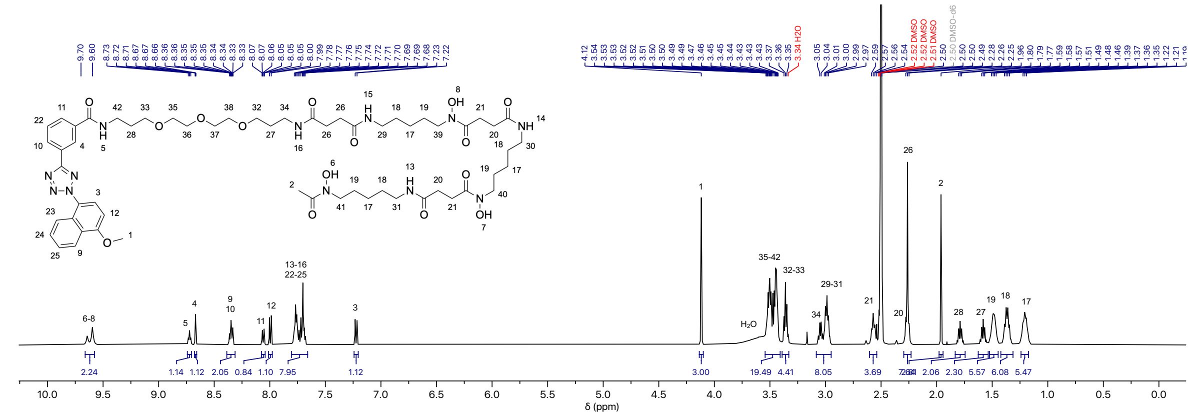

Figure S206. ^{1}H (400 MHz, DMSO-d^{6}, 298 K) NMR spectrum of compound **14**

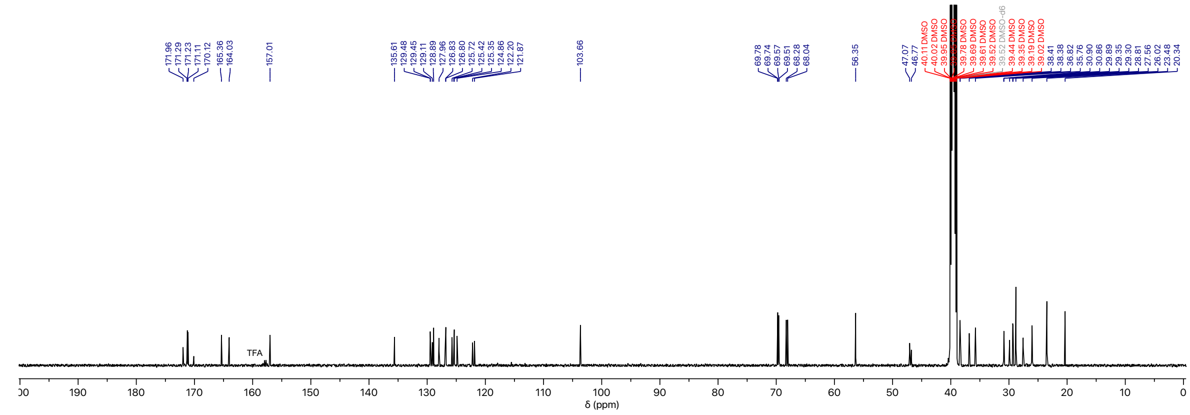

Figure S207. ^{13}C (126 MHz, DMSO-d^{6}, 298 K) NMR spectrum of compound **14**

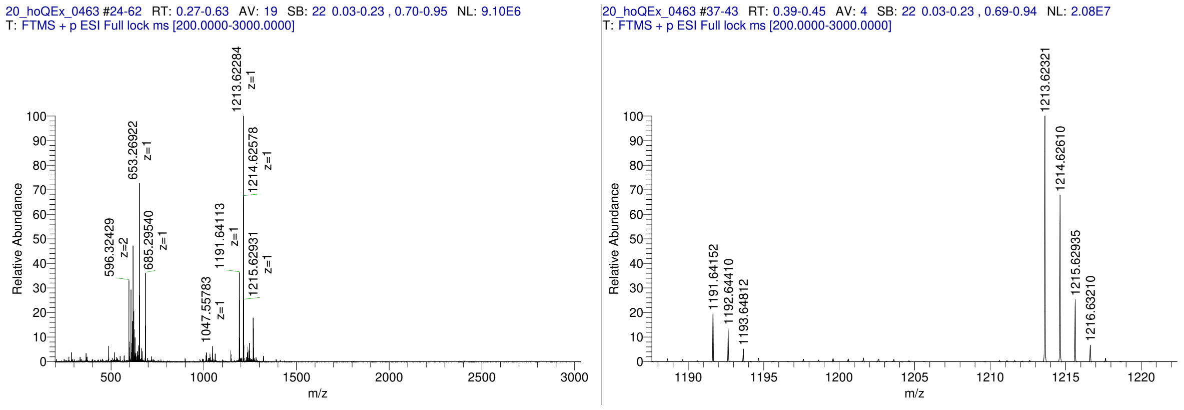

Figure S208. HR-ESI-MS spectrum of compound **14**

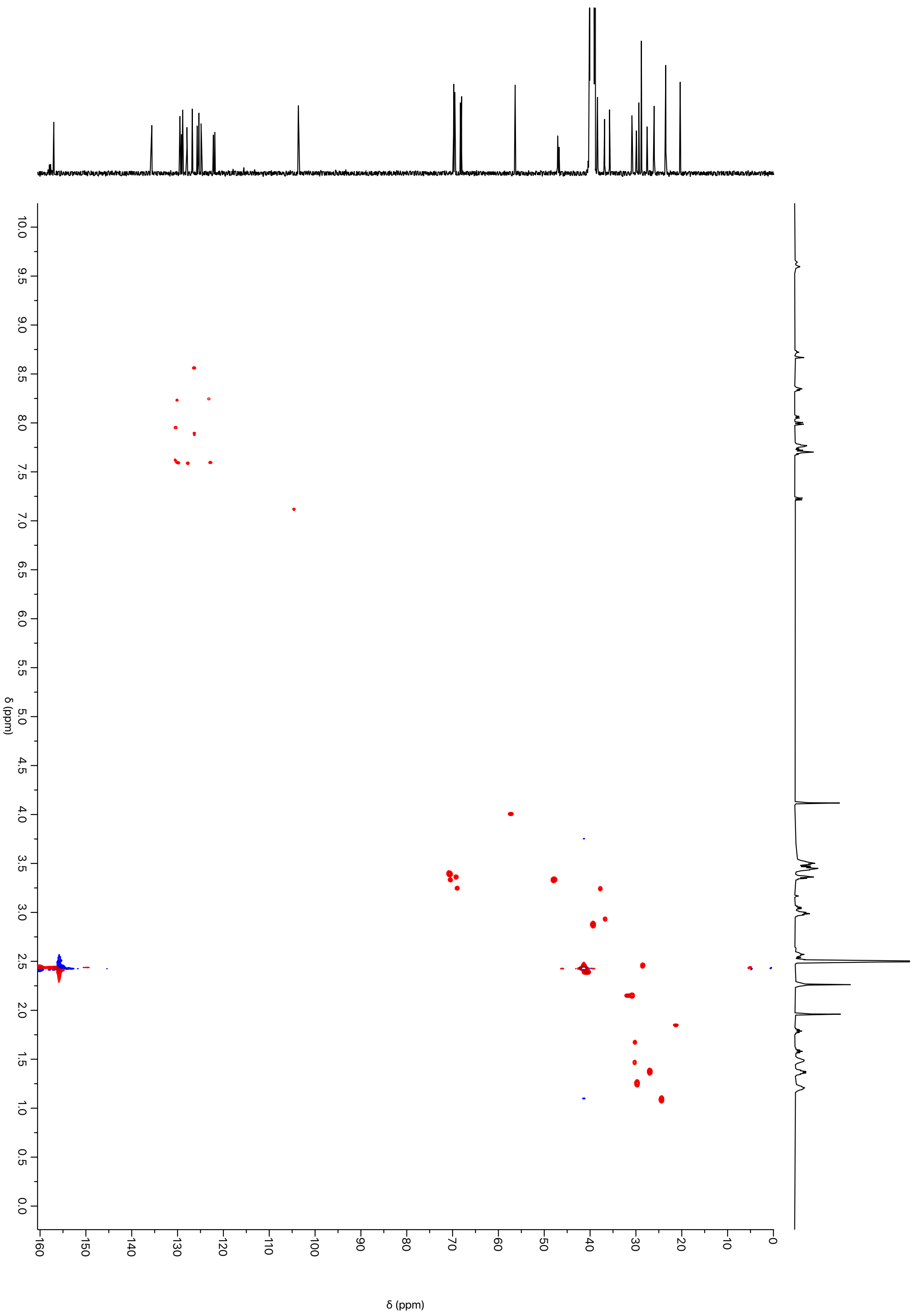

Figure S209. ^{1}H-^{13}C (HSQC, DMSO-d^{6}, 298 K) 2D NMR spectrum of compound **14**

Synthesis of non-PEGylated DFO-ArN3 derivatives

Synthesis of DFO-para-ArN3 (60)

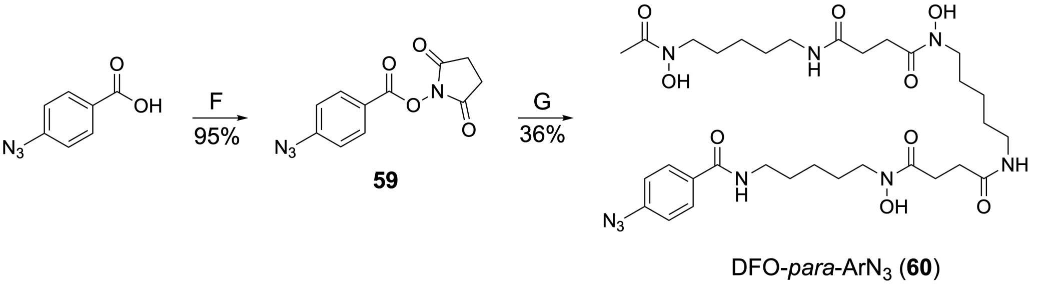

Scheme S13. Synthetic pathway toward DFO-*para*-ArN3 (**60**)

Compound 59

Compound **59** was prepared from 4-aminobenzoic acid (100 mg, 0.61 mmol) according to **General Procedure F** to give **59** as a pale-yellow solid (151 mg, 95% yield). **^{1}H NMR:** ($CDCl_3$, 400 MHz, 298 K) δ (ppm) 8.13 (d, J = 8.8 Hz, 2H, CH_{Ar}), 7.13 (d, J = 8.7 Hz, 2H, CH_{Ar}), 2.91 (s, 4H, CH_2). ^{1}H NMR spectroscopic data agrees with published values.[7]

Compound 60

Compound **60** was prepared from compound **59** (26 mg, 0.1 mmol) according to **General Procedure G** to give **60** as an off-white solid (13 mg, 36% yield). **^{1}H NMR:** (DMSO-d^6, 500 MHz, 298 K) δ (ppm) 9.63 (s, 1H), 8.44 (t, J = 5.7 Hz, 1H, N*H*), 7.88 (d, J = 8.7 Hz, 2H, CH_{Ar}), 7.78 (d, J = 5.9 Hz, 2H, O*H*), 7.19 (d, J = 8.7 Hz, 2H, CH_{Ar}), 3.50 – 3.42 (m, 6H, CH_2), 3.22 (q, J = 6.6 Hz, 2H, CH_2), 2.99 (q, J = 6.5 Hz, 4H, CH_2), 2.56 (q, J = 7.8 Hz, 4H, CH_2), 2.26 (t, J = 7.4 Hz, 4H, CH_2), 1.96 (s, 3H, CH_3), 1.56 – 1.46 (m, 8H, CH_2), 1.38 (m, 5H, CH_2), 1.30 – 1.18 (m, 6H, CH_2). **^{13}C{^{1}H} NMR:** (DMSO-d^6, 126 MHz, 298 K) δ (ppm) 172.0 (C=O), 171.3 (C=O), 170.1 (C=O), 165.1 (C=O), 142.1 (C_{qt}), 131.2 (C_{qt}), 129.0 (CH_{Ar}), 118.8 (CH_{Ar}), 47.1 (CH_2), 46.8 (CH_2), 38.4 (CH_2), 29.9 (CH_2), 28.8 (CH_2), 27.6 (CH_2), 26.1 (CH_2), 26.0 (CH_2), 23.6 (CH_2), 23.5 (CH_2), 20.3 (CH_3). **HR-ESI-MS:** *m/z* calcd. for $[C_{32}H_{51}N_9O_9+H]^+$ 706.3810, found 706.3884.

Synthesis of DFO-4F-p-ArN$_3$ (62)

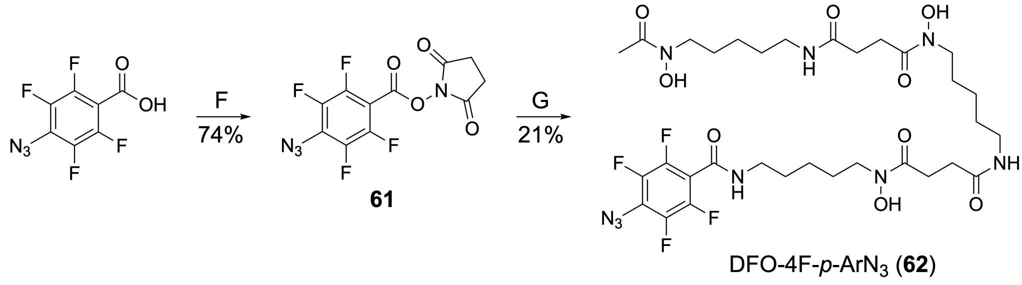

Scheme S14. Synthetic pathway toward DFO-4F-*p*-ArN$_3$ (**62**)

Compound 61

Compound **61** was prepared from 4-azido-2,3,5,6-tetrafluorobenzoic acid (43 mg, 0.183 mmol) according to **General Procedure F** to give **61** as an off-white solid (45 mg, 74% yield). **^{1}H NMR:** ($CDCl_3$, 500 MHz, 298 K) δ (ppm) 2.92 (s, 4H, CH_2).

Compound 62

Compound **62** was prepared from compound **61** (16 mg, 0.047 mmol) according to **General Procedure G** to give **62** as a pale-green solid (6 mg, 21% yield). **^{1}H NMR:** (DMSO-d^6, 500 MHz, 298 K) δ (ppm) 9.67 – 9.59 (m, 3H), 8.86 (t, J = 5.6 Hz, 1H, N*H*), 7.77 (t, J = 5.5 Hz, 2H, O*H*), 3.45 (t, J = 7.2 Hz, 8H, CH_2), 3.23 (q, J = 6.6 Hz, 2H, CH_2), 3.00 (td, J = 7.2, 3.6 Hz, 4H, CH_2), 2.56 (dt, J = 11.2, 6.6 Hz, 5H, CH_2), 2.26 (t, J = 7.4 Hz, 4H, CH_2), 1.96 (s, 3H, CH_3), 1.50 (m, 8H, CH_2), 1.38 (m, 5H, CH_2), 1.27 (m, 2H, CH_2), 1.25 – 1.16 (m, 5H, CH_2). **^{13}C{^{1}H} NMR:** (DMSO-d^6, 126 MHz, 298 K) δ (ppm) 172.0 (C=O), 171.3 (C=O), 170.1 (C=O), 156.7 (C=O), 47.0 (CH_2), 46.8 (CH_2), 38.4 (CH_2), 34.3 (CH_2), 29.9 (CH_2), 28.8 (CH_2), 28.3 (CH_2), 27.6 (CH_2), 26.0 (CH_2), 25.9 (CH_2), 23.5 (CH_2), 23.3 (CH_2), 20.3 (CH_3). **HR-ESI-MS:** *m*/*z* calcd. for $[C_{32}H_{47}F_4N_9O_9+H]^+$ 776.35111, found 778.35063; *m*/*z* calcd. for $[C_{32}H_{47}F_4N_9O_9+Na]^+$ 800.33306, found 800.33236.

Synthesis of DFO-m-NO$_2$-ArN$_3$ (63)

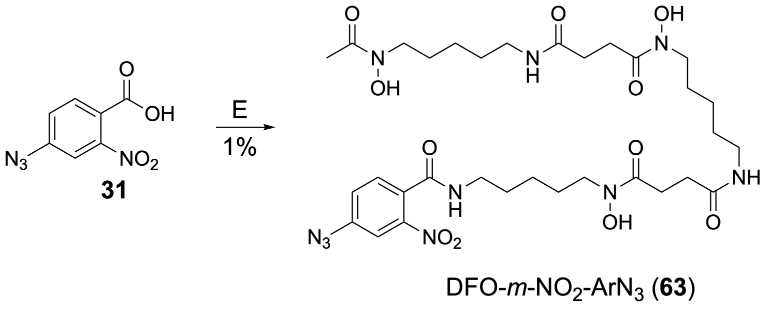

Scheme S15. Synthetic pathway toward DFO-*m*-NO_2-ArN_3 (**63**)

Compound 63

Compound **63** was prepared from compound **31** (76 mg, 0.1 mmol) according to **General Procedure E** to give **63** as an off-white solid (3 mg, 1% yield). **^{1}H NMR:** (DMSO-d^6, 500 MHz, 298 K) δ (ppm) 9.67 – 9.59 (m, 3H), 8.66 (t, *J* = 5.7 Hz, 1H, N*H*), 7.77 (t, *J* = 5.6 Hz, 2H, O*H*), 7.70 (d, *J* = 2.3 Hz, 1H, CH_{Ar}), 7.61 (d, *J* = 8.3 Hz, 1H, CH_{Ar}), 7.50 (dd, *J* = 8.3, 2.3 Hz, 1H, CH_{Ar}), 3.50 – 3.42 (m, 9H, CH_2), 3.18 (q, *J* = 6.6 Hz, 2H, CH_2), 3.00 (dq, *J* = 7.2, 5.1, 3.7 Hz, 4H, CH_2), 2.57 (dt, *J* = 7.9, 3.8 Hz, 4H, CH_2), 2.26 (t, *J* = 7.3 Hz, 4H, CH_2), 1.96 (s, 3H, CH_3), 1.51 (m, 9H, CH_2), 1.37 (m, 5H, CH_2), 1.32 – 1.18 (m, 8H, CH_2). **^{13}C{^{1}H} NMR:** (DMSO-d^6, 126 MHz, 298 K) δ (ppm) 172.0 (C=O), 171.3 (C=O), 170.1 (C=O), 164.4 (C=O), 148.5 (C_{qt}), 142.0 (C_{qt}), 130.5 (CH_{Ar}), 128.4 (C_{qt}), 123.3 (CH_{Ar}), 114.8 (CH_{Ar}), 47.1 (CH_2), 46.8 (CH_2), 38.4 (CH_2), 29.9 (CH_2), 28.8 (CH_2), 28.4 (CH_2), 27.6 (CH_2), 26.0 (CH_2), 23.5 (CH_2), 23.4 (CH_2), 20.3 (CH_3). **HR-ESI-MS:** *m/z* calcd. for $[C_{32}H_{51}N_{10}O_{11}–H]^-$ 749.35823, found 749.35995.

Synthesis of DFO-2,6-naphthyl-N_3 (65)

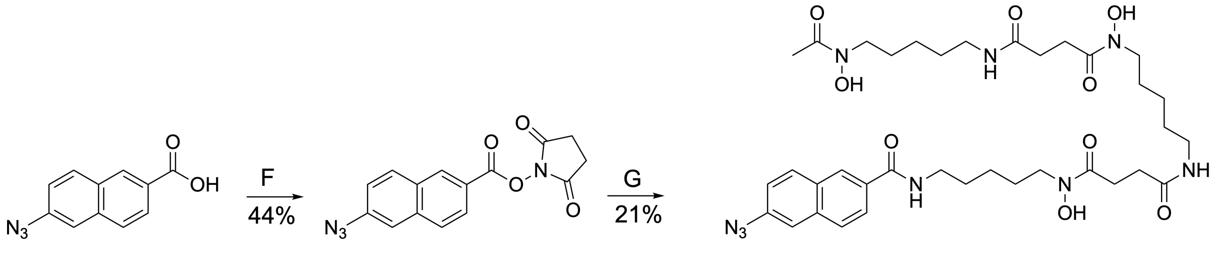

46 **64** DFO-2,6-naphthyl-N_3 (**65**)

Scheme S16. Synthetic pathway toward DFO-2,6-naphthyl-N_3 (**65**)

Compound 64

Compound **64** was prepared from compound **46** (56 mg, 0.549 mmol) according to **General Procedure F** to give **64** as a pale-orange solid (35 mg, 44% yield). **^{1}H NMR:** ($CDCl_3$, 400 MHz, 298 K) δ (ppm) 8.71 (d, *J* = 1.6 Hz, 1H, CH_{Ar}), 8.10 (dd, *J* = 8.6, 1.7 Hz, 1H, CH_{Ar}), 7.97 (d, *J* = 8.8 Hz, 1H, CH_{Ar}), 7.85 (d, *J* = 8.7 Hz, 1H, CH_{Ar}), 7.51 (d, *J* = 2.2 Hz, 1H, CH_{Ar}), 7.26 (dd, *J* = 8.9, 2.1 Hz, 3H, CH_{Ar}), 2.94 (s, 4H, CH_2).

Compound 65

Compound **65** was prepared from compound **64** (27 mg, 0.1 mmol) according to **General Procedure G** to give **65** as an off-white solid (8 mg, 21% yield). **^{1}H NMR:** (DMSO-d^6, 500 MHz, 298 K) δ (ppm) 9.62 (s, 3H), 8.58 (s, 1H, N*H*), 8.42 (s, 1H, CH_{Ar}), 8.07 (d, *J* = 8.8 Hz, 1H, CH_{Ar}), 7.94 (d, *J* = 3.8 Hz, 2H, CH_{Ar}), 7.75 (d, *J* = 22.1 Hz, 3H, O*H*), 7.33 (d, *J* = 7.8 Hz, 1H, CH_{Ar}), 3.47 (dt, *J* = 18.8, 7.1 Hz, 9H, CH_2), 2.99 (q, *J* = 9.8, 8.3 Hz, 6H, CH_2), 2.57 (m, 8H, CH_2), 2.26 (m, 6H, CH_2), 1.96 (s, 3H, CH_2), 1.53 (m, 12H, CH_2), 1.42 – 1.27 (m, 10H, CH_2), 1.21 (m, 7H, CH_2). **^{13}C{^{1}H} NMR:** (DMSO-d^6, 126 MHz, 298 K) δ (ppm) 172.0 (C=O), 172.0 (C=O), 171.3 (C=O), 170.1 (C=O), 166.0 (C=O), 138.6 (C_{qt}), 134.8 (C_{qt}), 131.6 (C_{qt}), 131.1 (CH_{Ar}), 129.7 (C_{qt}), 127.3 (CH_{Ar}), 127.0 (CH_{Ar}), 125.3 (CH_{Ar}), 119.6 (CH_{Ar}), 115.6 (CH_{Ar}), 47.1 (CH_2), 46.8 (CH_2), 38.4 (CH_2), 30.7 (CH_2), 29.9 (CH_2), 28.9 (CH_2), 28.8 (CH_2), 27.6 (CH_2), 26.0 (CH_2), 23.7 (CH_2), 23.5 (CH_2), 20.3 (CH_3). **HR-ESI-MS:** *m*/*z* calcd. for $[C_{36}H_{53}N_9O_9+Na]^+$ 778.38639, found 778.44824.

Synthesis of DFO-Benzophenone (66)

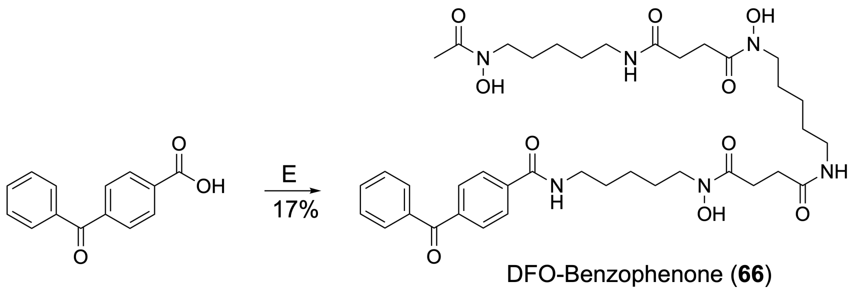

Scheme S17. Synthetic pathway toward DFO-Benzophenone (**66**)

Compound 66

Compound **66** was prepared from 4-benzoylbenzoic acid (104 mg, 0.458 mmol) according to **General Procedure E** to give **66** as a white solid (41 mg, 17% yield). **^{1}H NMR:** (DMSO-d^{6}, 400 MHz, 298 K) δ (ppm) 9.62 (m, 3H, O*H*), 8.65 (t, *J* = 5.4, 1H, CH_{Ar}), 7.98 (d, *J* = 8.5, 2H, CH_{Ar}), 7.80 – 7.74 (m, 6H, CH_{Ar} + N*H*), 7.72 – 7.68 (m, 1H, CH_{Ar}), 7.58 (d, *J* = 7.5, 2H, CH_{Ar}), 3.50 – 3.43 (m, 6H, CH_2), 3.28 – 3.25 (m, 2H, CH_2), 3.00 – 2.99 (m, 4H, CH_2), 2.59 – 2.56 (m, 4H, CH_2), 2.26 (t, *J* = 7.2, 4H, CH_2), 1.96 (s, 3H, CH_3), 1.58 – 1.47 (m, 8H, CH_2), 1.39 – 1.36 (m, 4H, CH_2), 1.31 – 1.21 (m, 6H, CH_2). **^{13}C{^{1}H} NMR:** (DMSO-d^{6}, 126 MHz, 298 K) δ (ppm). 195.4 (C=O), 172.0 (C=O), 171.3 (C=O), 170.1 (C=O), 165.4 (C=O), 139.0 (C_{qt}), 138.0 (C_{qt}), 136.7 (C_{qt}), 133.0 (CH_{Ar}); 129.7 (CH_{Ar}), 129.5 (CH_{Ar}), 128.7 (CH_{Ar}), 127.3 (CH_{Ar}), 47.1 (CH_2), 46.8 (CH_2), 39.3 (CH_2), 38.4 (CH_2), 34.3 (CH_2), 29.9 (CH_2), 28.8 (CH_2), 28.7 (CH_2), 27.6 (CH_2), 26.0 (CH_2), 23.6 (CH_2), 23.5 (CH_2), 20.3 (CH_3) (^{13}C-NMR was measured of a different batch than ^{1}H-NMR and contains an unknown impurity). **HR-ESI-MS:** *m*/*z* calcd. for $[C_{39}H_{56}N_6O_{10}+Na]^+$ 791.39556, found 791.39432.

Synthesis of DFO-Diazirine (67)

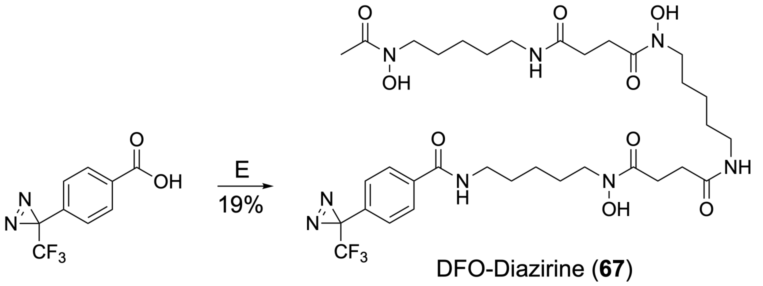

Scheme S18. Synthetic pathway toward DFO-Diazirine (**67**)

Compound 67

Compound **67** was prepared from 4-(3-(trifluoromethyl)-3*H*-diazirin-3-yl)benzoic acid (52 mg, 0.458 mmol) according to **General Procedure E** to give **67** as a white solid (23 mg, 19% yield). **^{1}H NMR:** (DMSO-d^6, 500 MHz, 298 K) δ (ppm) 9.65 – 9.61 (m, 3H, O*H*), 8.59 (t, *J* = 5.3, 1H, N*H*), 7.94 (d, *J* = 8.5, 2H, CH_{Ar}), 7.77 (m, 2H, N*H*), 7.36 (d, *J* = 8.1, 2H, CH_{Ar}), 3.48 – 3.43 (m, 6H, CH_2), 3.26 – 3.21 (m, 2H, CH_2), 3.00 – 2.99 (m, 4H, CH_2), 2.59 – 2.55 (m, 4H, CH_2), 2.26 (m, 4H, CH_2), 1.96 (s, 3H, CH_3), 1.51 – 1.50 (m, 8H, CH_2), 1.39 – 1.34 (m, 4H, CH_2), 1.28 – 1.21 (m, 6H, CH_2). **^{13}C{^{1}H} NMR:** (DMSO-d^6, 126 MHz, 298 K) δ (ppm) 172.4 – 165.5 (C=O), 136.7 (C_{qt}), 130.49 (C_{qt}), 128.5 (CH_{Ar}), 126.9 (CH_{Ar}), 47.6 – 47.2 (CH_2), 39.4 (CH_2), 38.9 (2 x CH_2), 30.7 (2 x CH_2), 29.3 – 29.2 (4 x CH_2), 28.0 (2 x CH_2), 26.5 (2 x CH_2), 24.1 – 24.0 (3 x CH_2), 20.1 (CH_3). **HR-ESI-MS:** *m*/*z* calcd. for $[C_{34}H_{51}F_3N_8O_9+Na]^+$ 795.36288, found 795.36237.

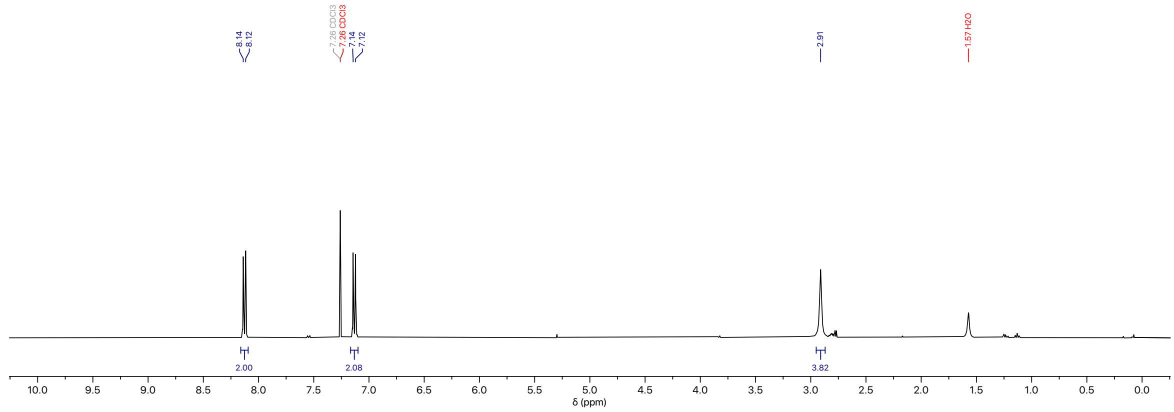

Figure S210. ^{1}H (400 MHz, $CDCl_3$, 298 K) NMR spectrum of compound **59**

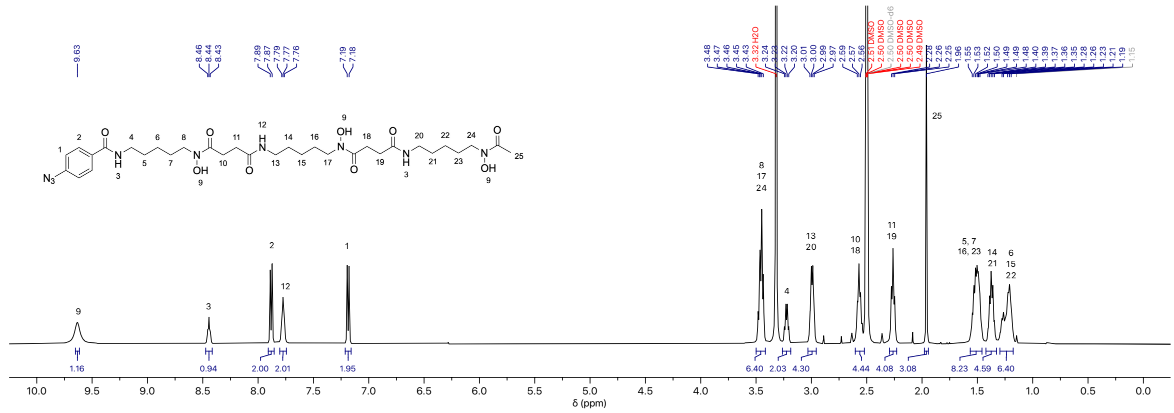

Figure S211. ^{1}H (500 MHz, DMSO-d^6, 298 K) NMR spectrum of compound **60**

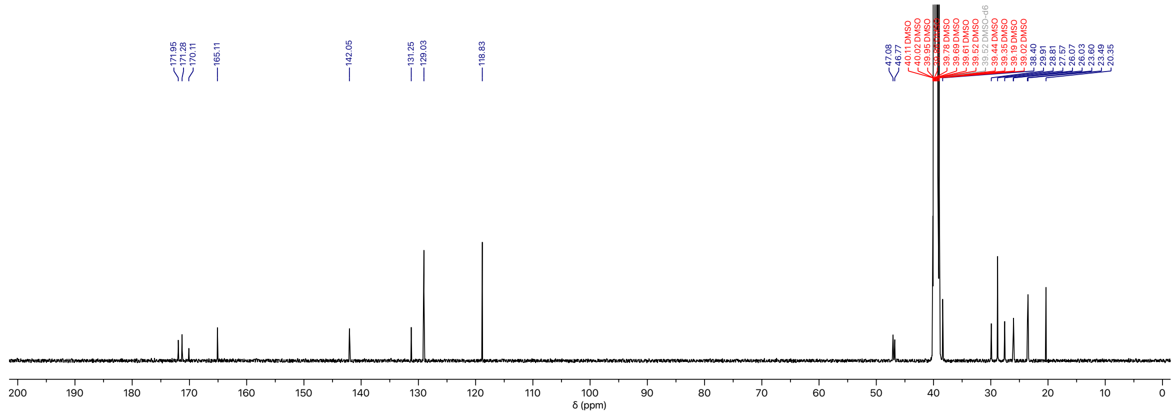

Figure S212. ^{13}C (126 MHz, DMSO-d^6, 298 K) NMR spectrum of compound **60**

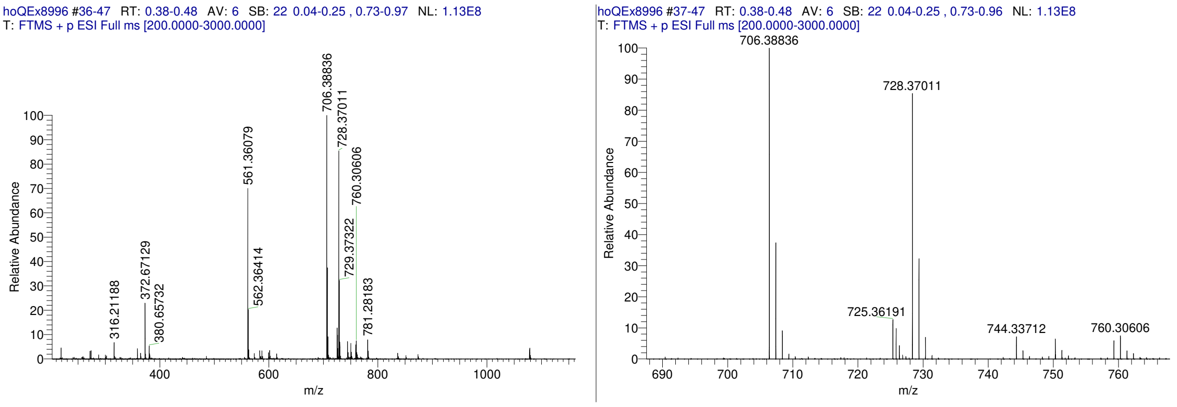

Figure S213. HR-ESI-MS spectrum of compound **60**

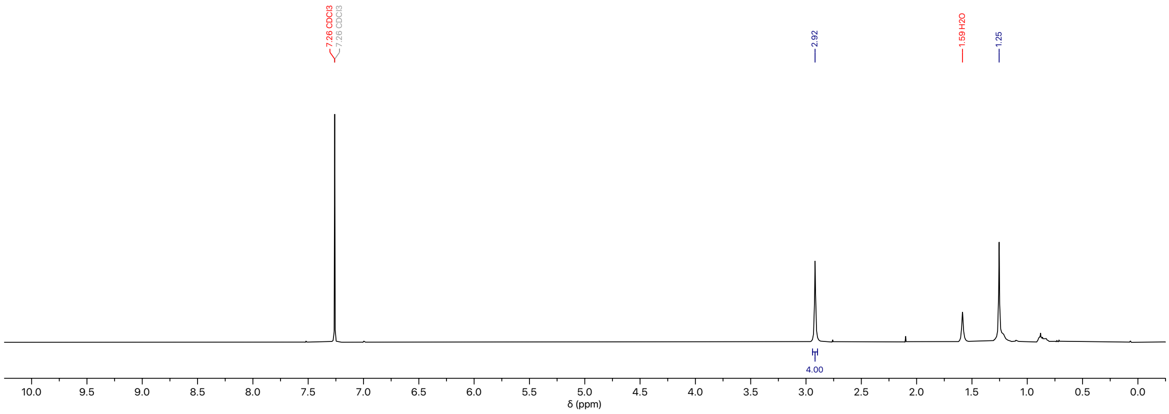

Figure S214. ^{1}H (500 MHz, $CDCl_3$, 298 K) NMR spectrum of compound **61**

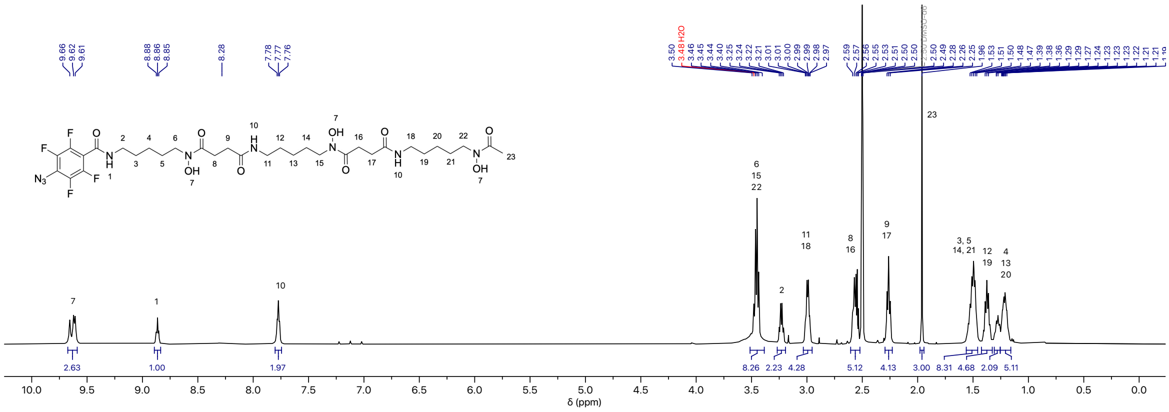

Figure S215. ^{1}H (500 MHz, DMSO-d^6, 298 K) NMR spectrum of compound **62**

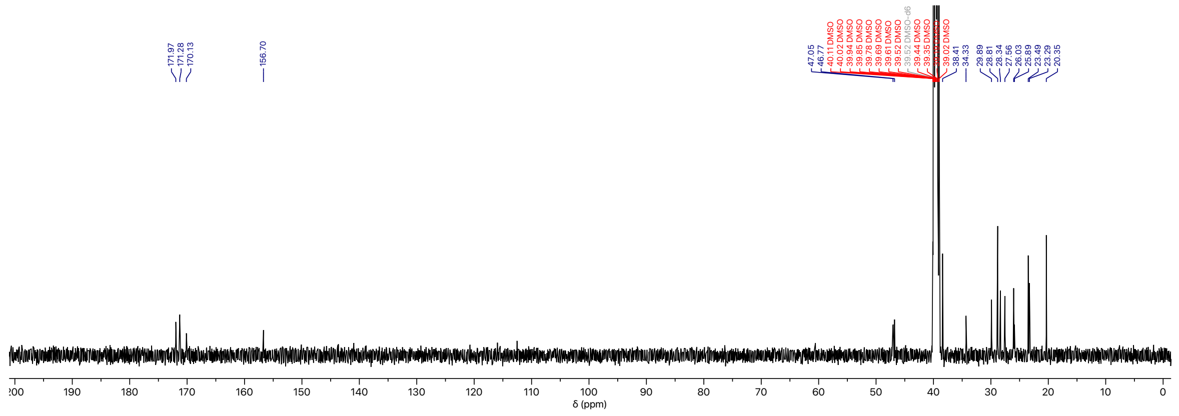

Figure S216. ^{13}C (126 MHz, DMSO-d^6, 298 K) NMR spectrum of compound **62**

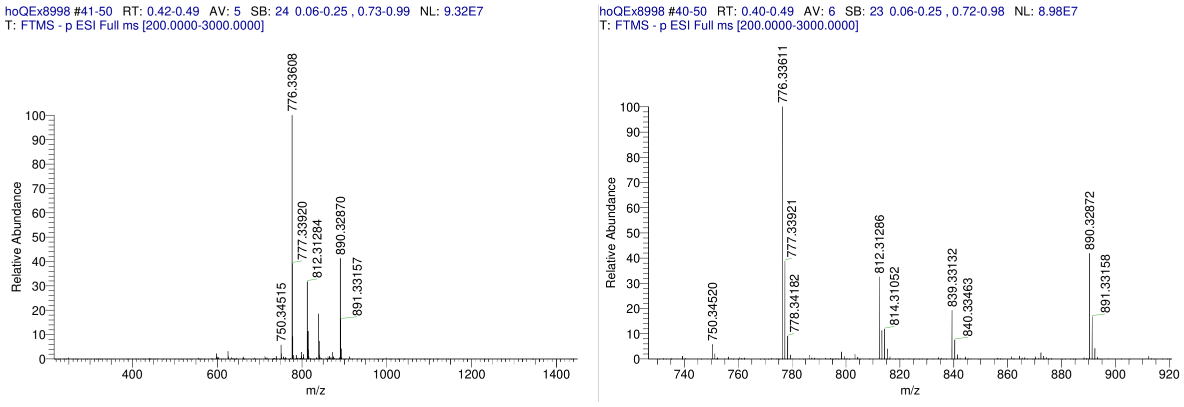

Figure S217. HR-ESI-MS spectrum of compound **62**

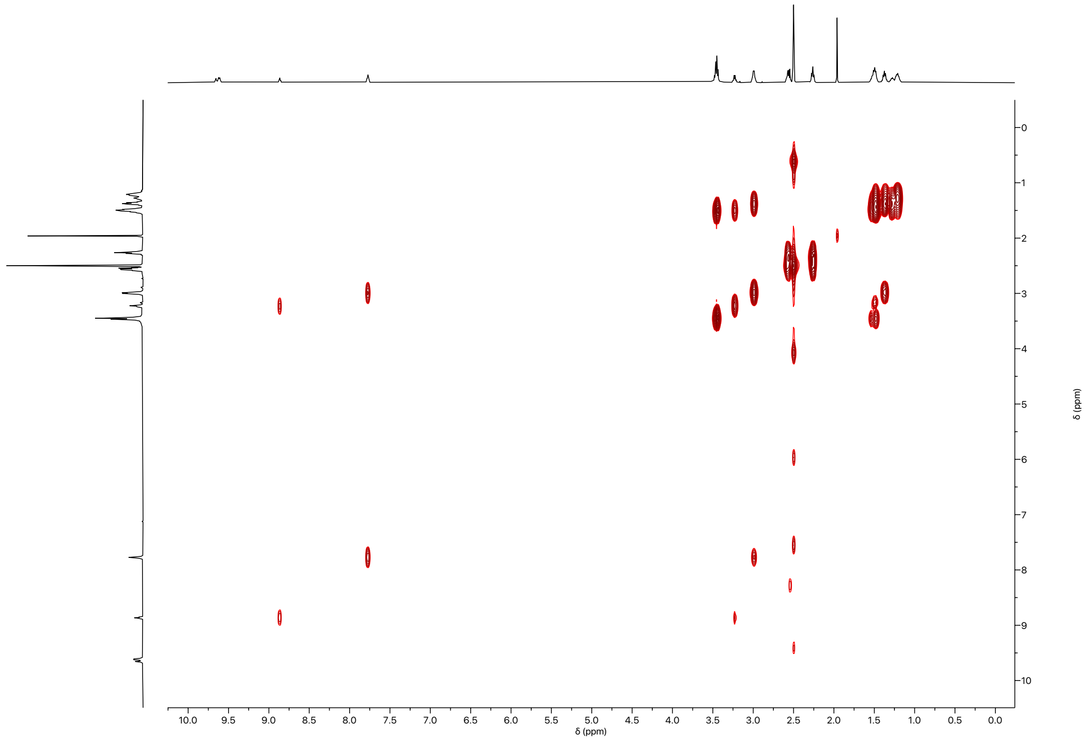

Figure S218. ^{1}H-^{1}H (COSY, DMSO-d^6, 298 K) 2D NMR spectrum of compound **62**

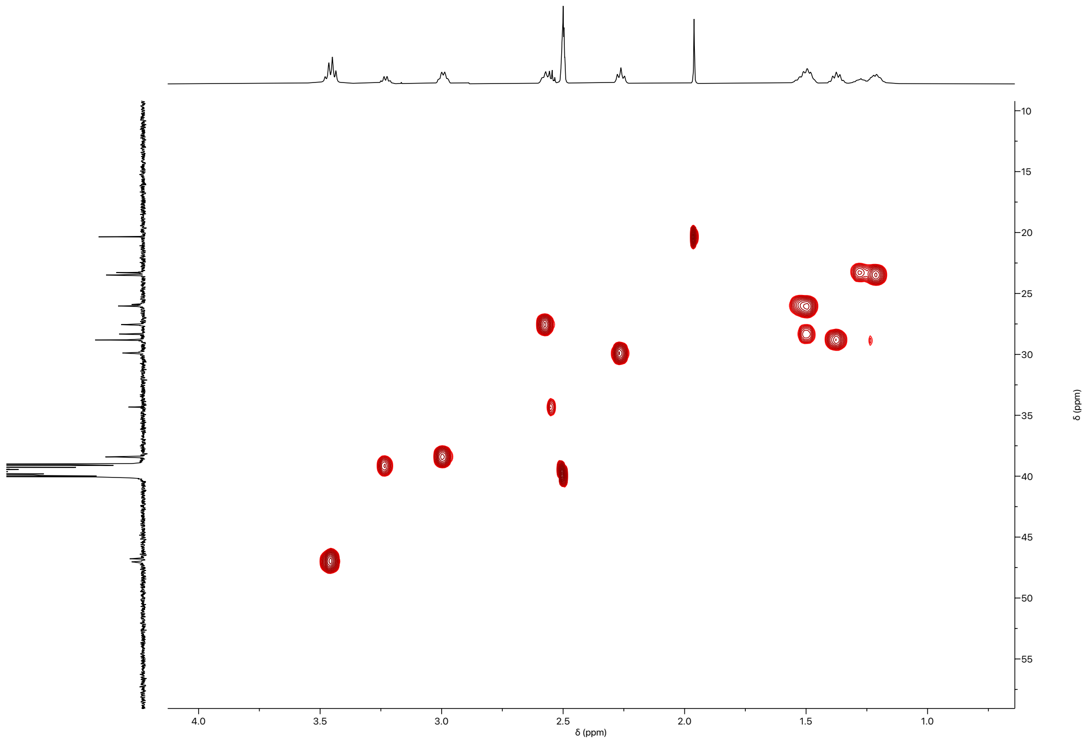

Figure S219. ^{1}H-^{13}C (HSQC, DMSO-d^6, 298 K) 2D NMR spectrum of compound **62**

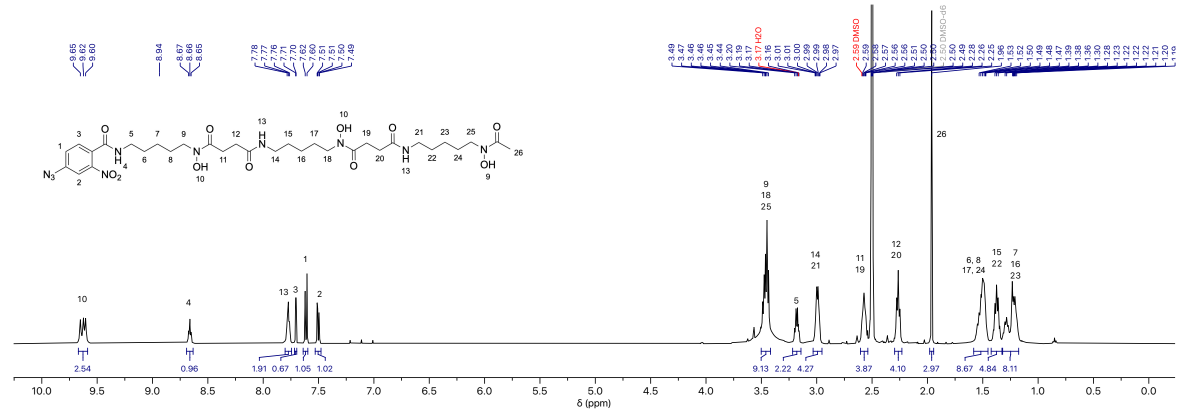

Figure S220. ^{1}H (500 MHz, DMSO-d^{6}, 298 K) NMR spectrum of compound **63**

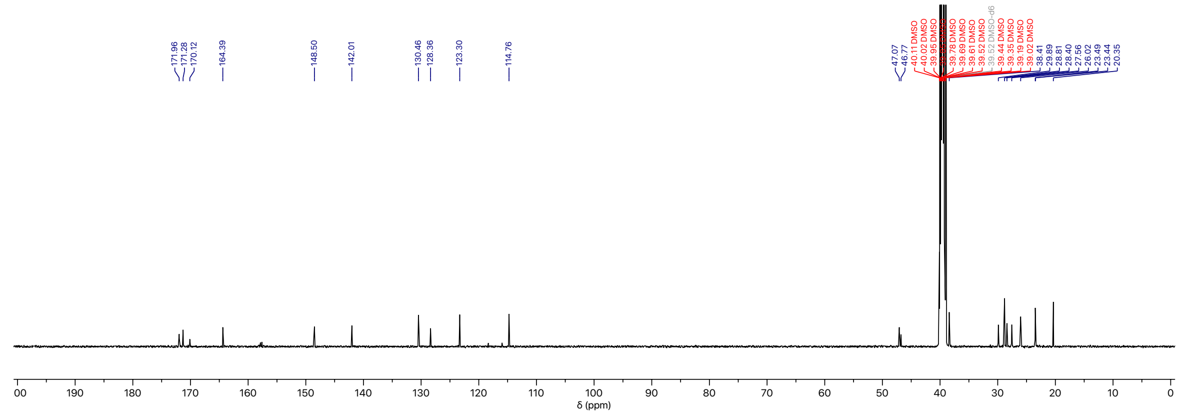

Figure S221. ^{13}C (126 MHz, DMSO-d^{6}, 298 K) NMR spectrum of compound **63**

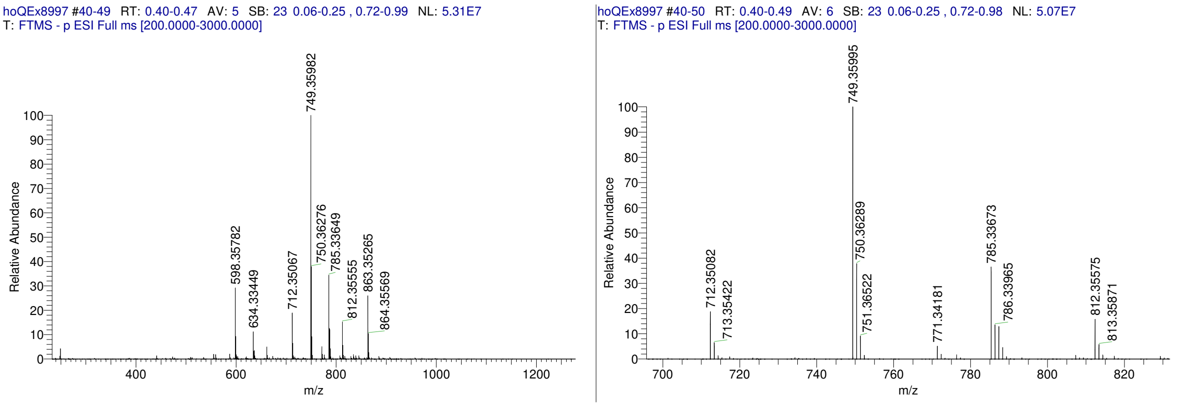

Figure S222. HR-ESI-MS spectrum of compound **63**

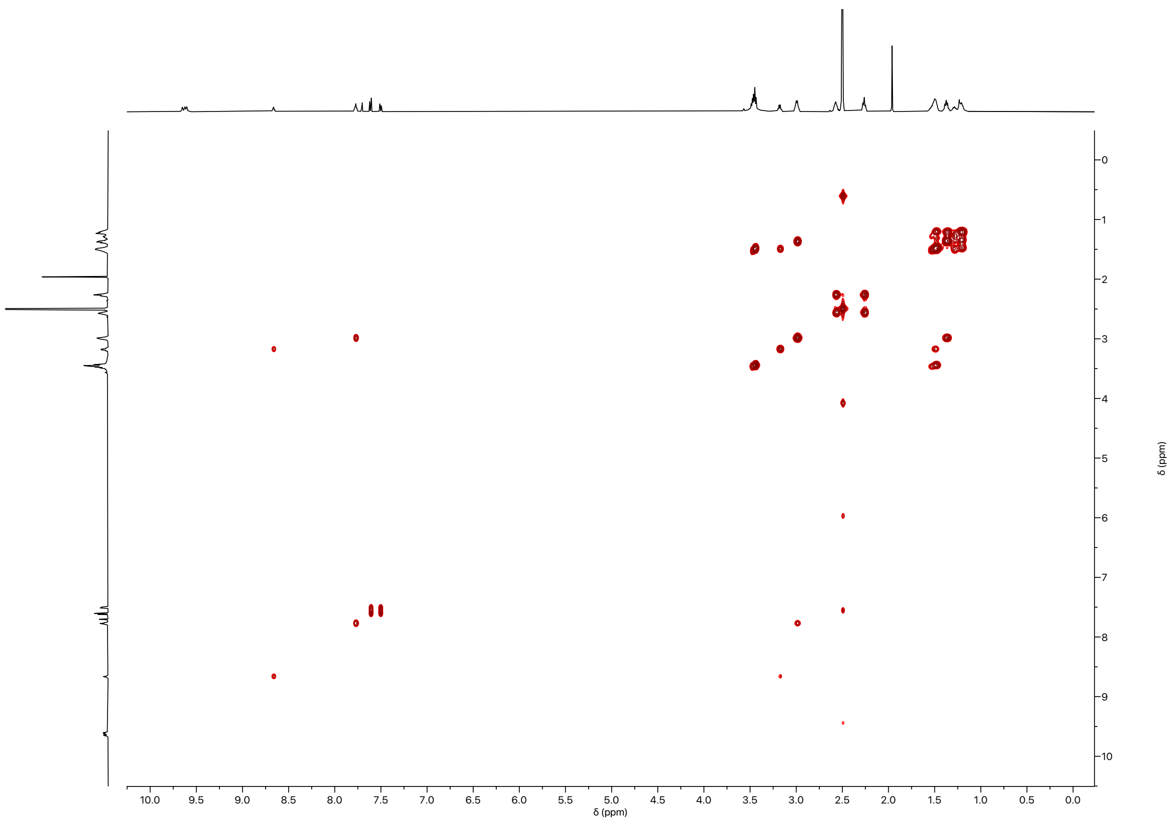

Figure S223. ^{1}H-^{1}H (COSY, DMSO-d^{6}, 298 K) 2D NMR spectrum of compound **63**

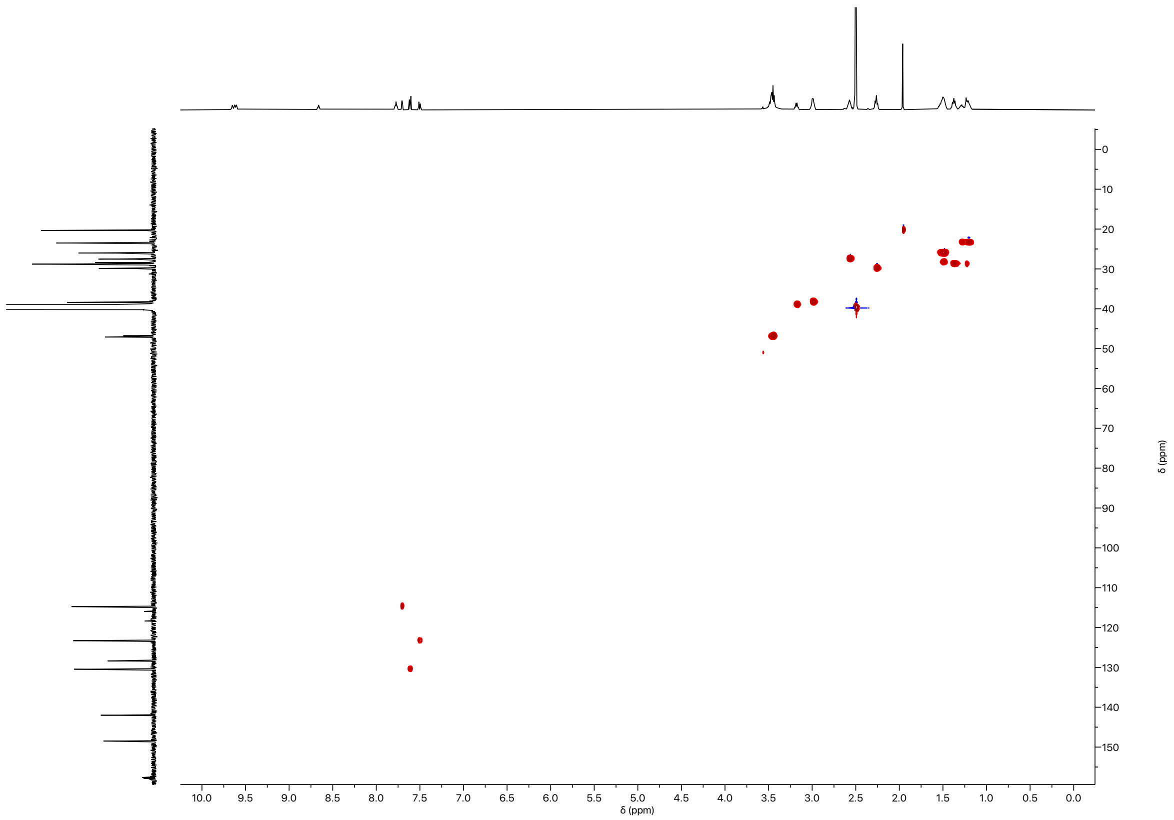

Figure S224. ^{1}H-^{13}C (HSQC, DMSO-d^{6}, 298 K) 2D NMR spectrum of compound **63**

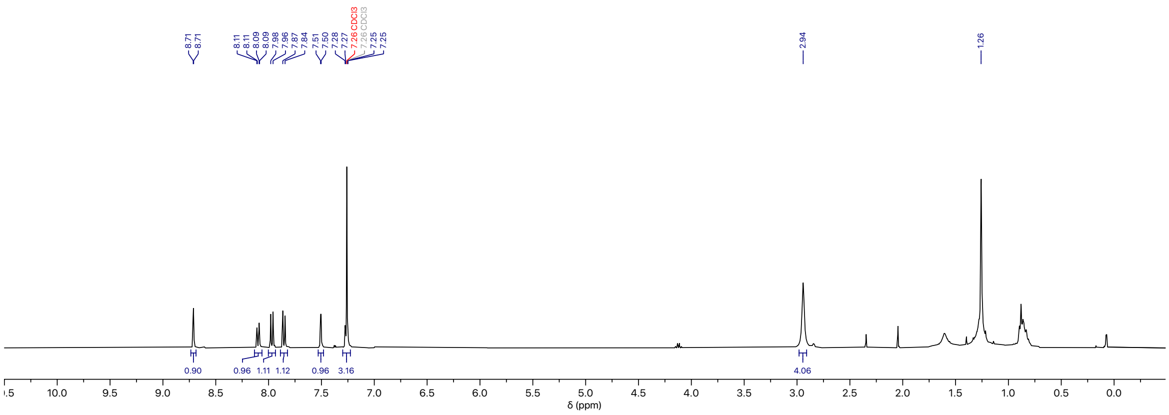

Figure S225. ^{1}H (400 MHz, $CDCl_3$, 298 K) NMR spectrum of compound **64**

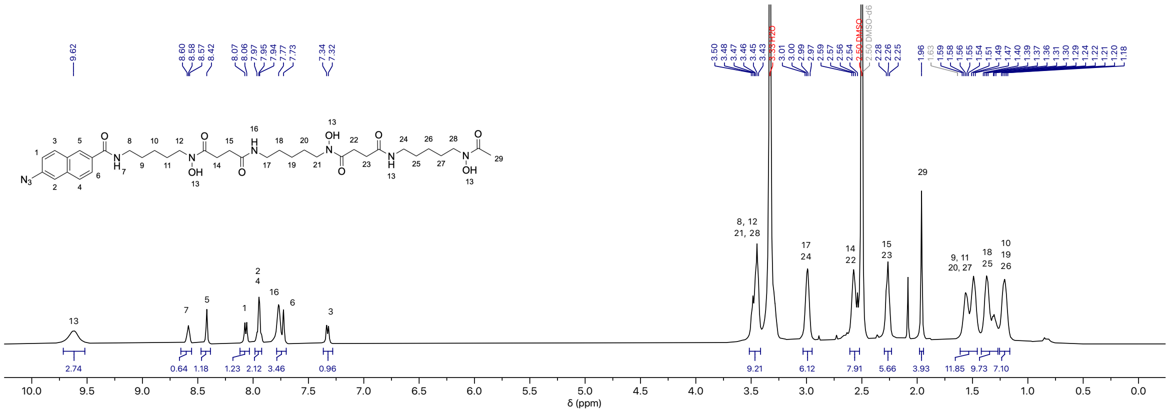

Figure S226. ^{1}H (500 MHz, DMSO-d^6, 298 K) NMR spectrum of compound **65**

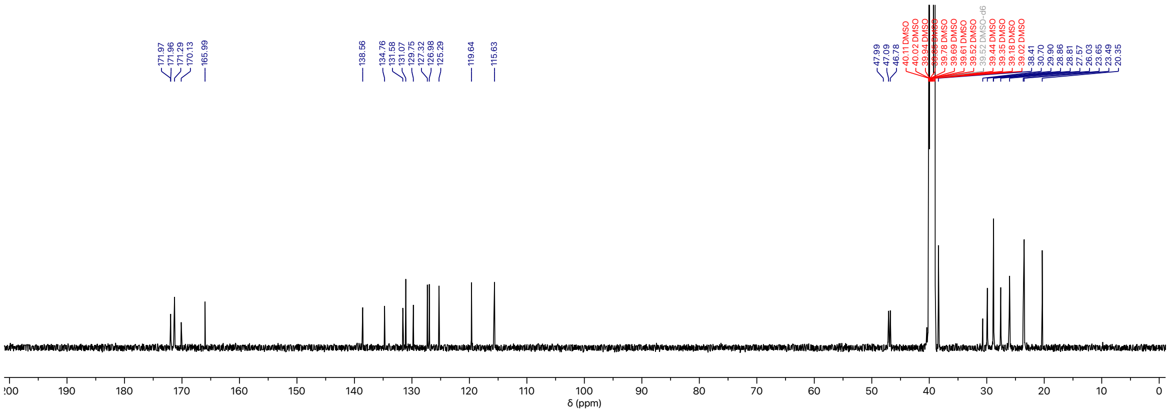

Figure S227. ^{13}C (126 MHz, DMSO-d^6, 298 K) NMR spectrum of compound **65**

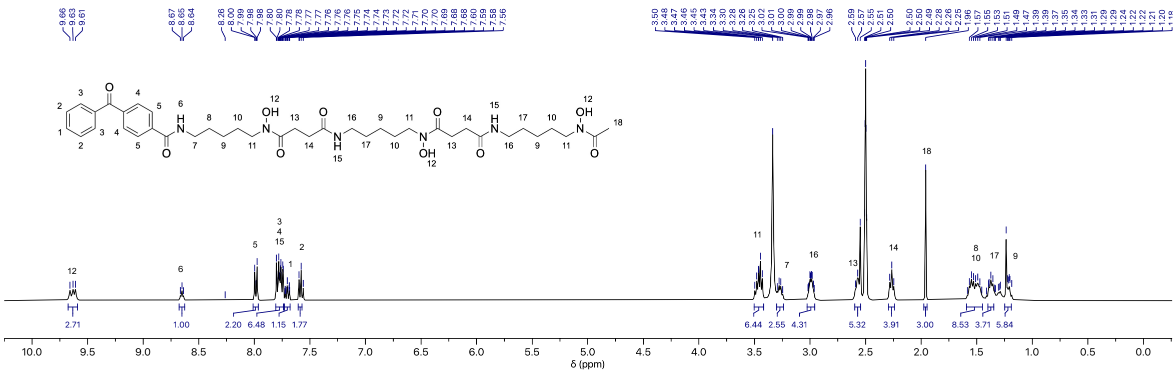

Figure S228. ^{1}H (400 MHz, DMSO-d^{6}, 298 K) NMR spectrum of compound **66**

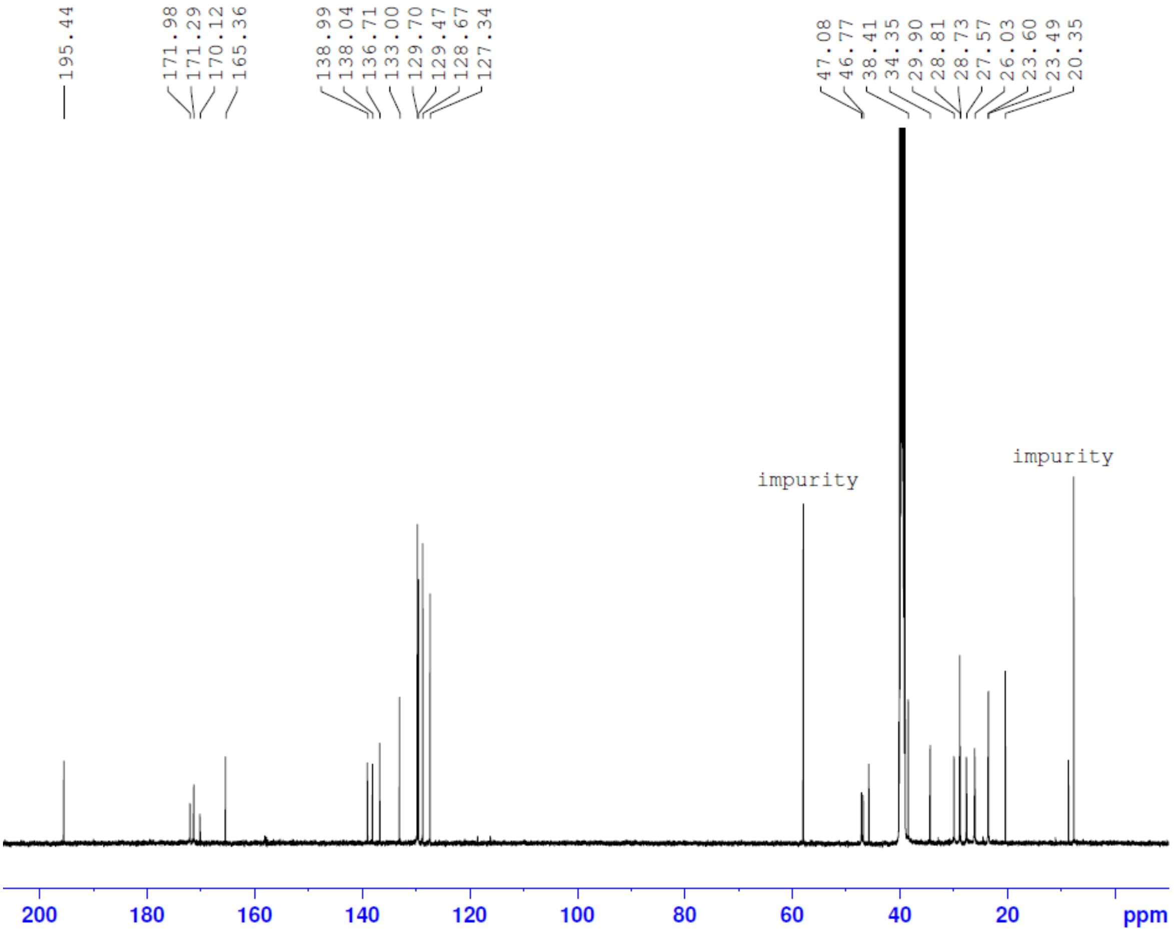

Figure S229. ^{13}C (126 MHz, DMSO-d^{6}, 298 K) NMR spectrum of compound **66**

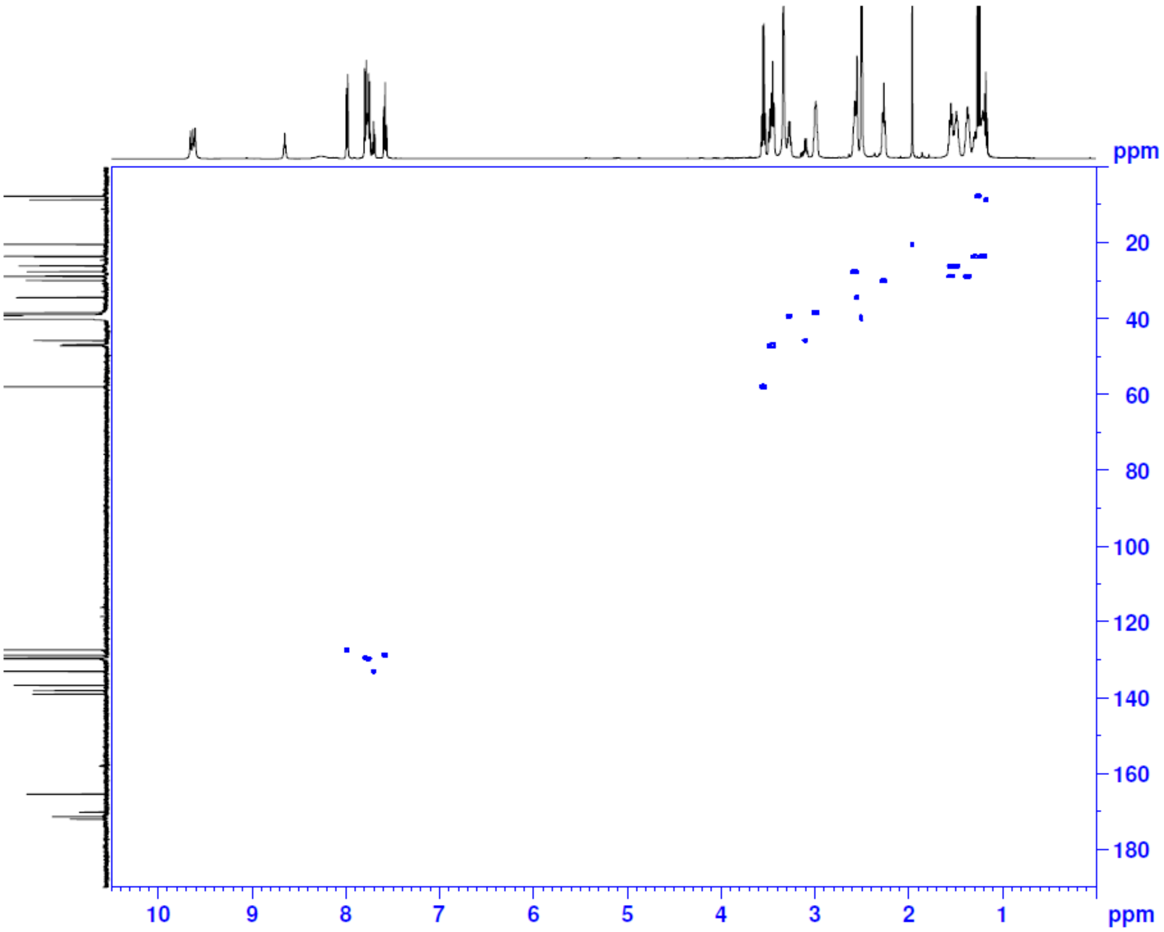

Figure S230. ^{1}H-^{13}C (HSQC, DMSO-d^{6}, 298 K) 2D NMR spectrum of compound **66**

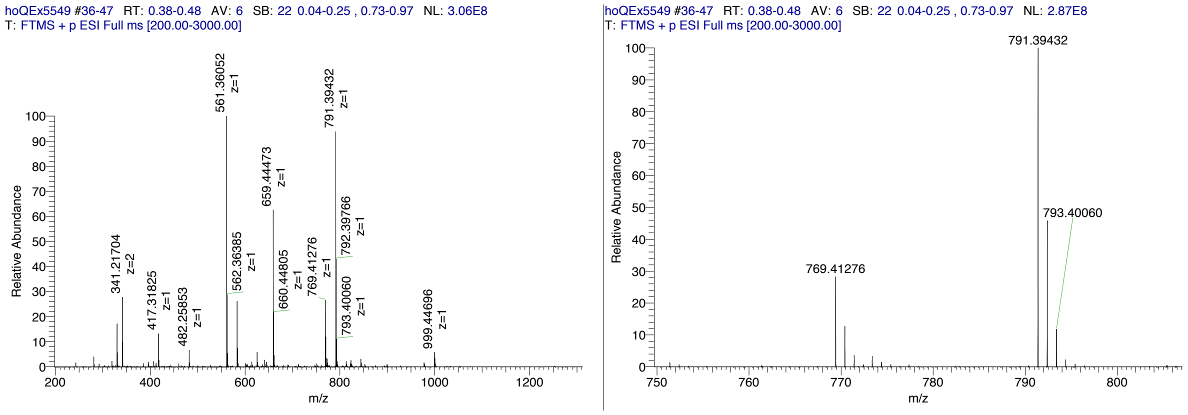

Figure S231. HR-ESI-MS spectrum of compound **66**

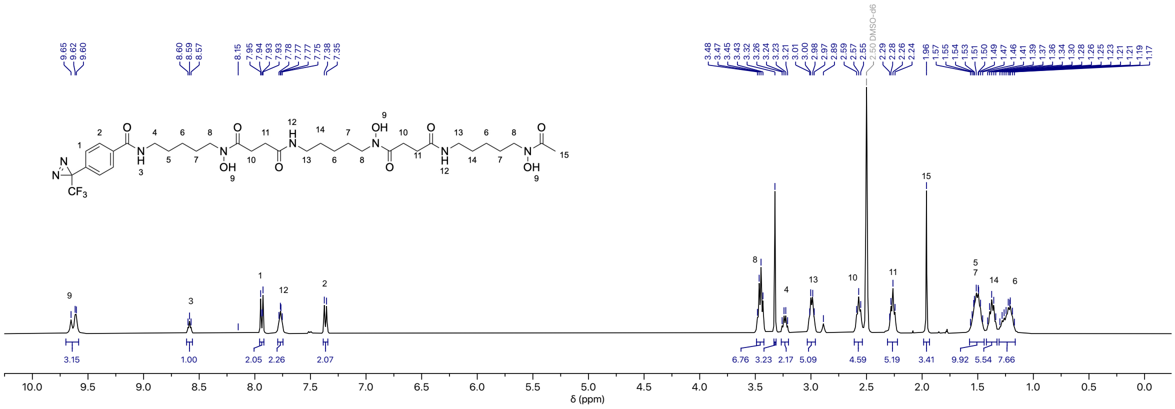

Figure S232. ^{1}H (500 MHz, DMSO-d^{6}, 298 K) NMR spectrum of compound **67**

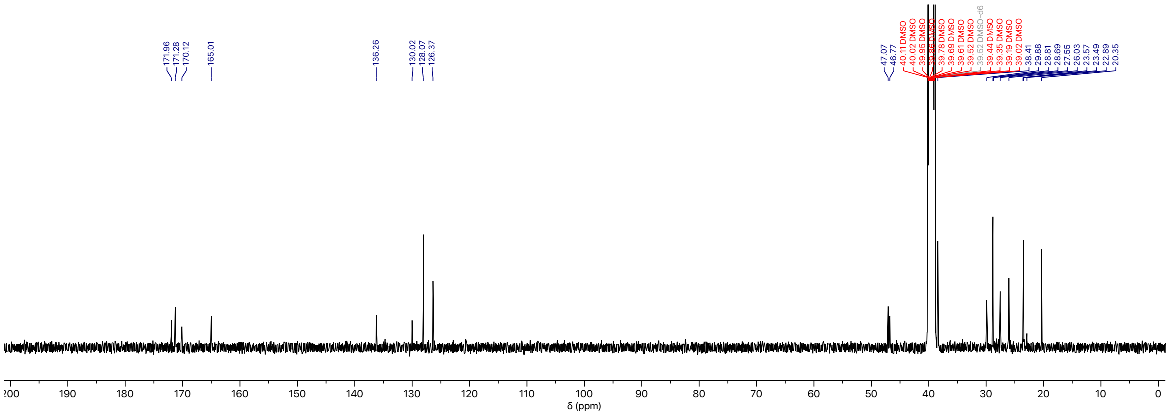

Figure S233. ^{13}C (126 MHz, DMSO-d^{6}, 298 K) NMR spectrum of compound **67**

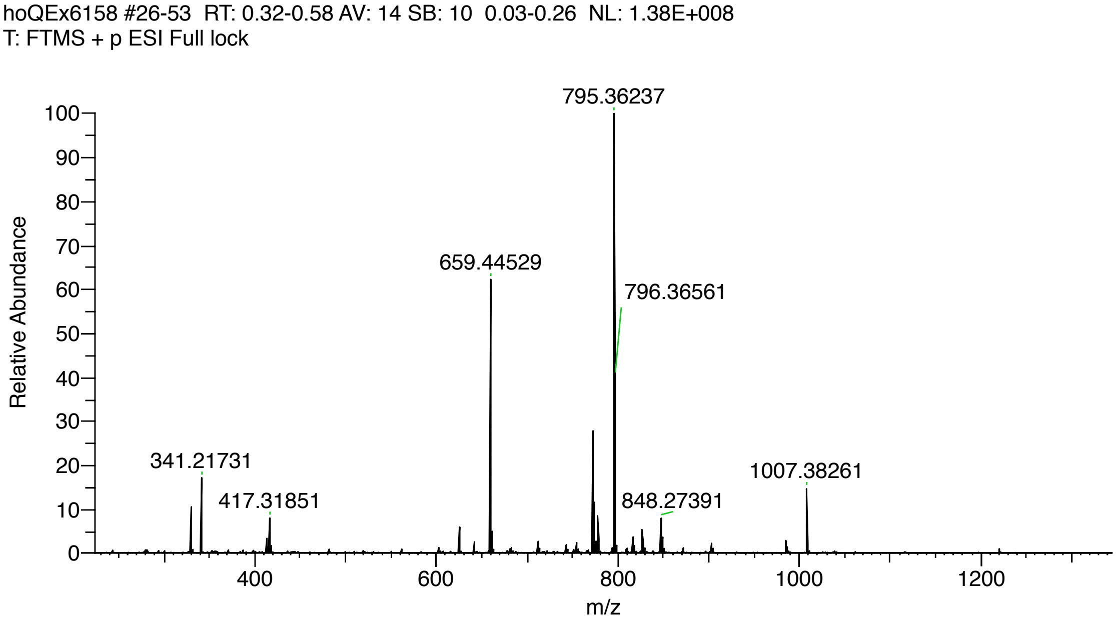

Figure S234. HR-ESI-MS spectrum of compound **67**

Radiochemistry results

^{89}Zr-photoradiolabeling of with non-PEGylated DFO compounds

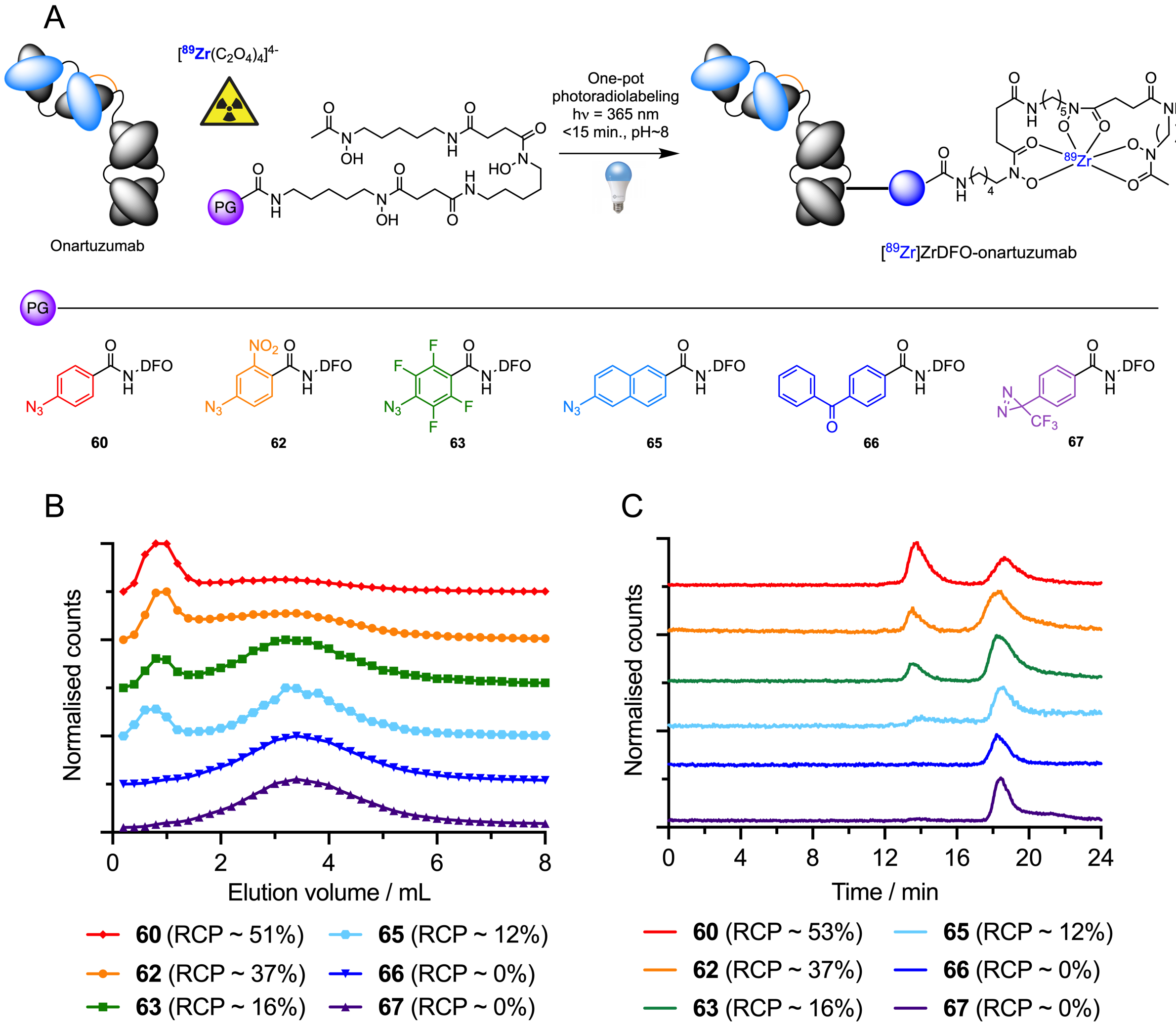

Figure S235. Data on the one-pot ^{89}Zr-radiolabeling and photo-induced protein conjugation between the photoactivatable non-PEGylated DFO derivatives, and the monovalent, monoclonal scFv-Fc engineered antibody fragment onartuzumab.

(A) General reaction scheme and chemical structures of the non-PEGylated photoactivatable DFO derivatives. (B) Elution profiles obtained from manual size-exclusion chromatography on the crude reaction samples using analytical PD-10 columns. (C) Automated SEC-HPLC chromatograms of the crude reaction mixtures obtained after one-pot photoradiolabeling of onartuzumab. The formation of radiolabeled [^{89}Zr]ZrDFO-onartuzumab conjugates is confirmed by the presence of a radiolabeled peak that coelutes with the onartuzumab protein at 13.7 min. Small-molecule byproducts elute at longer retention times between ~17.5 to 21.0 min.

natZr labeling of compounds 1 to 14

The $^{nat}Zr^{+}$ complexes of compounds **1** to **14** (~1 mg, 1 equiv.) were prepared by incubation with Zr(acac)$_4$ (~0.5 mg, 1.1 equiv.) in a 1:9 DMSO:MeOH (v/v; 1 mL) solution at 50 ºC for 1 h. After this time, compounds ^{nat}Zr-**1**$^{+}$ to ^{nat}Zr-**14**$^{+}$ were characterized by reverse-phase HPLC and HR-ESI-MS (solvent A = H_2O + 0.1% TFA; solvent B = MeOH; gradient = 0-2 min 60% A 40% B, 2-8 min 40-100% B, 8-11 min 100% B; (**Figure S236A** and **Table S1**).

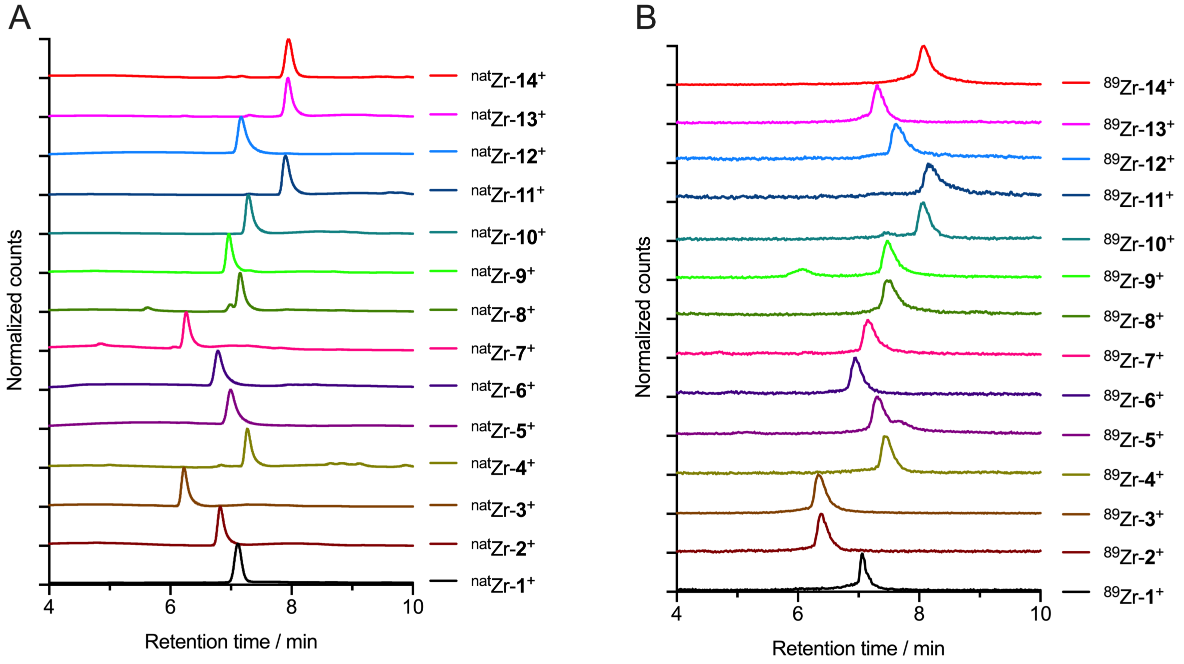

Figure S236. Reverse-phase HPLC chromatograms for (A) the ^{nat}Zr and (B) the ^{89}Zr complexes formed with compounds **1** to **14**.

Table S1. Reverse-phase HPLC retention times and HR-ESI-MS data for the $^{nat}Zr/^{89}Zr$ complexes of compounds **1** to **14**.

	**Reverse-phase HPLC R_t (min)**			**HR-ESI-MS analysis**		
**Compound**	Ligand	^{nat}Zr-complex	^{89}Zr-complex	Molecular Formula	calcd.	found
**1**	8.80	7.11	7.10	$C_{46}H_{74}N_{11}O_{14}Zr$	1094.44583	1094.44643
**2**	8.81	6.82	6.38	$C_{46}H_{74}N_{11}O_{14}Zr$	1094.44583	1094.44561
**3**	9.03	6.22	6.33	$C_{46}H_{74}N_{11}O_{14}Zr$	1094.44583	1094.44607
**4**	9.17	7.27	7.42	$C_{48}H_{78}N_{11}O_{14}Zr$	1122.47713	1122.47526
**5**	8.82	6.99	7.32	$C_{47}H_{76}N_{11}O_{15}Zr$	1124.45639	1124.45560
**6**	8.72	6.79	6.95	$C_{46}H_{73}ClN_{11}O_{14}Zr$	1128.40685	1128.40549
**7**	8.18	6.26	7.15	$C_{46}H_{73}N_{12}O_{16}Zr$	1139.43090	1139.42930
**8**	8.25	7.15	7.52	$C_{46}H_{70}F_4N_{11}O_{14}Zr$	1166.40814	1166.40785
**9**	8.27	6.96	7.47	$C_{45}H_{73}N_{12}O_{14}Zr$	1095.44107	1095.43965
**10**	9.06	7.29	8.07	$C_{50}H_{76}N_{11}O_{14}Zr$	1144.46148	1144.46173
**11**	9.69	7.90	8.15	$C_{50}H_{76}N_{11}O_{14}Zr$	1144.46148	1144.46031
**12**	8.80	7.17	7.62	$C_{53}H_{79}N_8O_{15}Zr$	1157.47064	1157.46860
**13**	9.64	7.94	7.30	$C_{48}H_{74}F_3N_{10}O_{14}Zr$	1161.43796	1161.43850
**14**	9.30	7.95	8.10	$C_{58}H_{83}N_{12}O_{15}Zr$	1277.51424	1277.51464

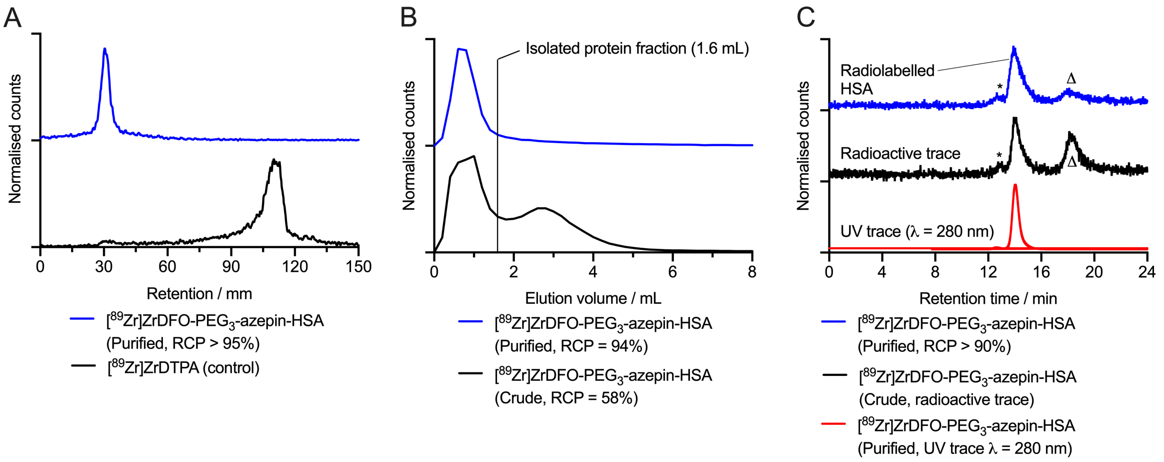

Figure S237. Characterization data for the radiochemical synthesis of [^{89}Zr]ZrDFO-PEG$_3$-*p*-azepin-HSA using DFO-PEG$_3$-*para*-ArN$_3$ (**1**) (irradiation at 395 nm). (A) Radio-iTLC chromatograms, (B) analytical PD-10-SEC profiles, and (C) SEC-HPLC chromatograms of the crude and purified product (*: aggregated protein, Δ: radiolabeled small molecules).[5]

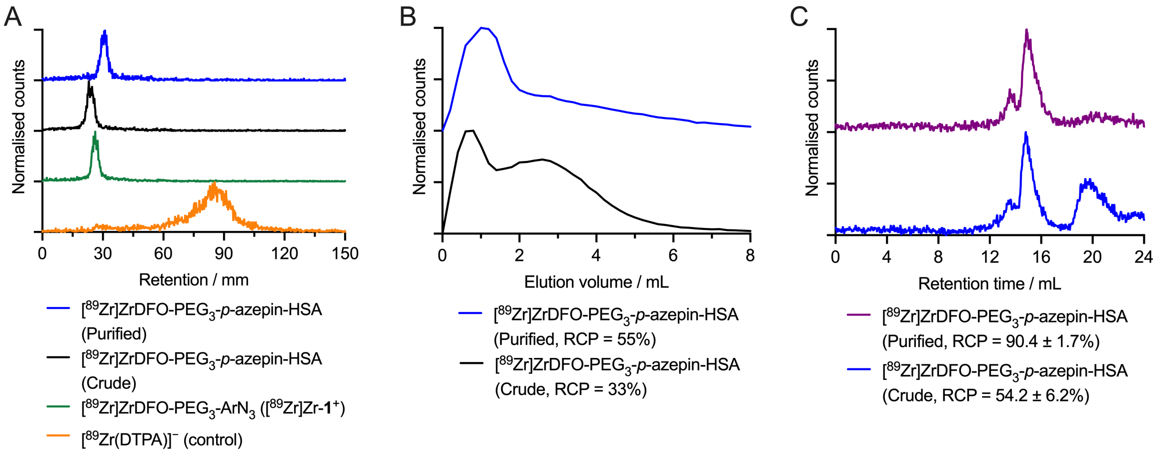

Figure S238. Characterization data for the radiochemical synthesis of [^{89}Zr]ZrDFO-PEG$_3$-*p*-azepin-HSA using DFO-PEG$_3$-*para*-ArN$_3$ (**1**) (irradiation in the visible region at 450 nm). (A) Radio-iTLC chromatograms, (B) analytical PD-10-SEC profiles, and (C) SEC-HPLC chromatograms of the crude and purified product (*: aggregated protein, Δ: radiolabeled small molecules).

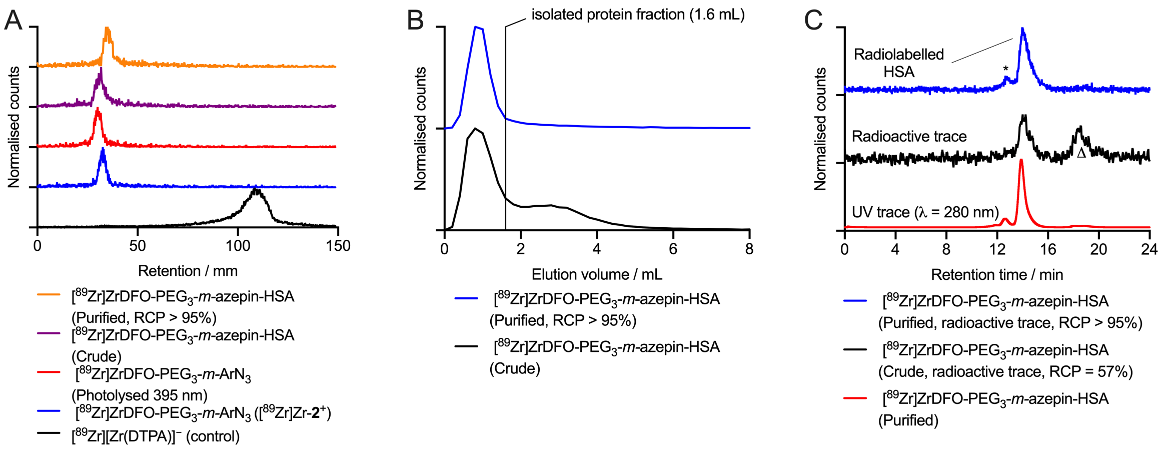

Figure S239. Characterization data for the radiochemical synthesis of [^{89}Zr]ZrDFO-PEG$_3$-*m*-azepin-HSA using DFO-PEG$_3$-*meta*-ArN$_3$ (**2**). (A) Radio-iTLC chromatograms, (B) analytical PD-10-SEC profiles, and (C) SEC-HPLC chromatograms of the crude and purified product (*: aggregated protein, Δ: radiolabeled small molecules).

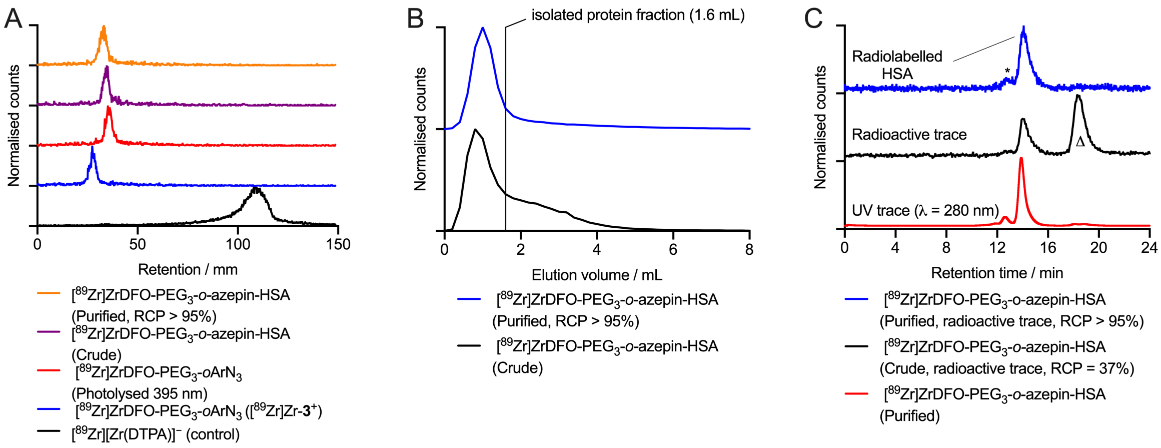

Figure S240. Characterization data for the radiochemical synthesis of [^{89}Zr]ZrDFO-PEG$_3$-*o*-azepin-HSA using DFO-PEG$_3$-*ortho*-ArN$_3$ (**3**). (A) Radio-iTLC chromatograms, (B) analytical PD-10-SEC profiles, and (C) SEC-HPLC chromatograms of the crude and purified product (*: aggregated protein, Δ: radiolabeled small molecules).

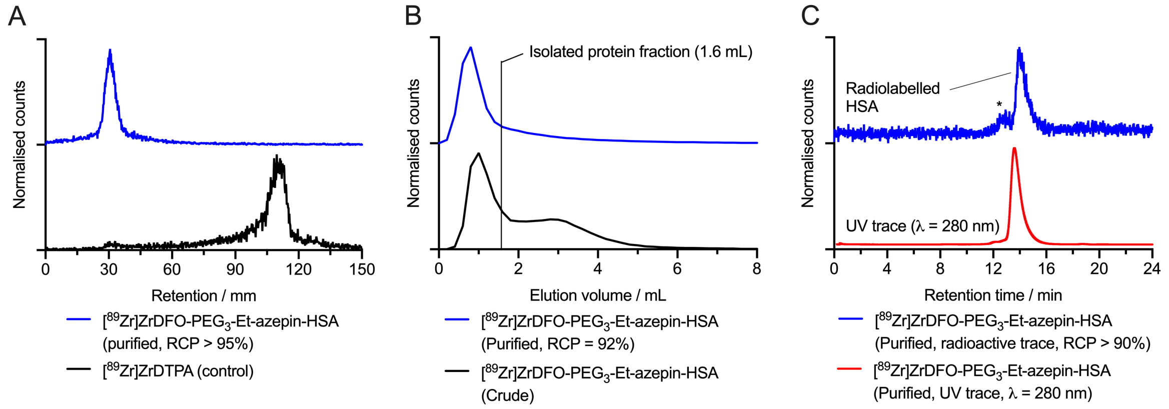

Figure S241. Characterization data for the radiochemical synthesis of [^{89}Zr]ZrDFO-PEG$_3$-*p*-Et-azepin-HSA using DFO-PEG$_3$-*p*-EtArN$_3$ (**4**). (A) Radio-iTLC chromatograms, (B) analytical PD-10-SEC profiles, and (C) SEC-HPLC chromatograms of the purified product (blue) and UV/vis (280 nm; red) of the protein. (*: aggregated protein, Δ: radiolabeled small molecules).[5]

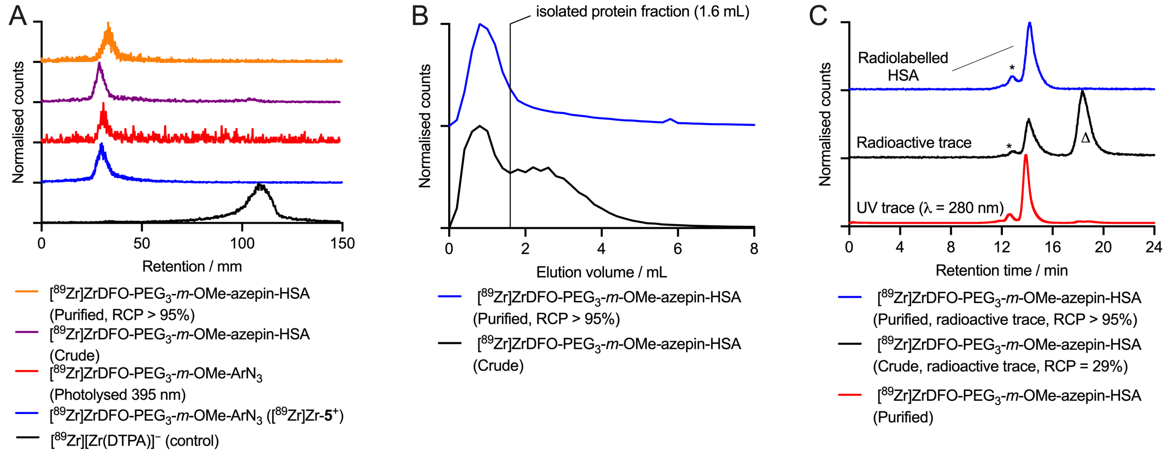

Figure S242. Characterization data for the radiochemical synthesis of [^{89}Zr]ZrDFO-PEG$_3$-*m*-OMe-azepin-HSA using DFO-PEG$_3$-*m*-OMe-ArN$_3$ (**5**). (A) Radio-iTLC chromatograms, (B) analytical PD-10-SEC profiles, and (C) SEC-HPLC chromatograms of the crude and purified product (*: aggregated protein, Δ: radiolabeled small molecules).

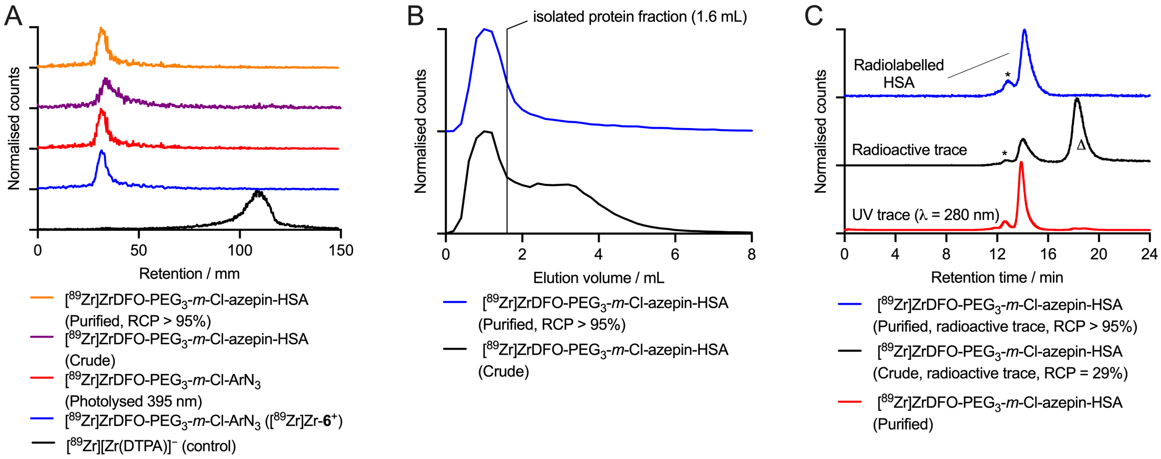

Figure S243. Characterization data for the radiochemical synthesis of [^{89}Zr]ZrDFO-PEG$_3$-*m*-Cl-azepin-HSA using DFO-PEG$_3$-*m*-Cl-ArN$_3$ (**6**). (A) Radio-iTLC chromatograms, (B) analytical PD-10-SEC profiles, and (C) SEC-HPLC chromatograms of the crude and purified product (*: aggregated protein, Δ: radiolabeled small molecules).

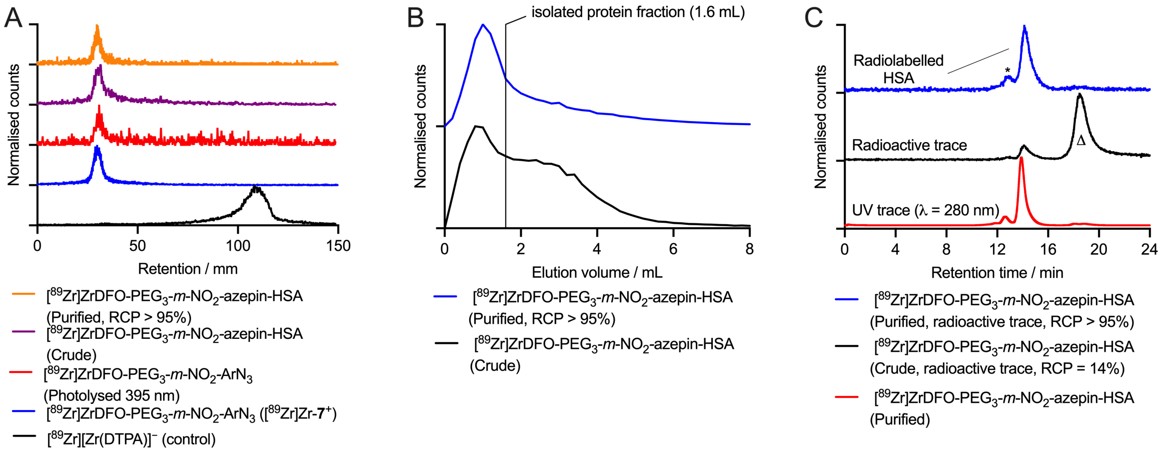

Figure S244. Characterization data for the radiochemical synthesis of [^{89}Zr]ZrDFO-PEG$_3$-*m*-NO$_2$-azepin-HSA using DFO-PEG$_3$-*m*-NO$_2$-ArN$_3$ (**7**). (A) Radio-iTLC chromatograms, (B) analytical PD-10-SEC profiles, and (C) SEC-HPLC chromatograms of the crude and purified product (*: aggregated protein, Δ: radiolabeled small molecules).

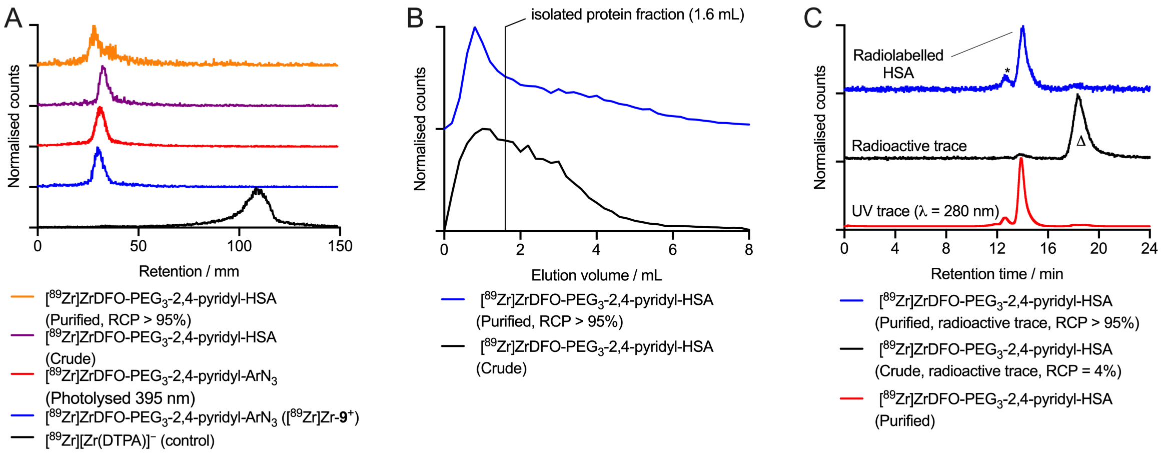

Figure S245. Characterization data for the radiochemical synthesis of [^{89}Zr]ZrDFO-PEG$_3$-2,4-pyridyl-HSA using DFO-PEG$_3$-2,4-pyridyl-ArN$_3$ (**9**). (A) Radio-iTLC chromatograms, (B) analytical PD-10-SEC profiles, and (C) SEC-HPLC chromatograms of the crude and purified product (*: aggregated protein, Δ: radiolabeled small molecules).

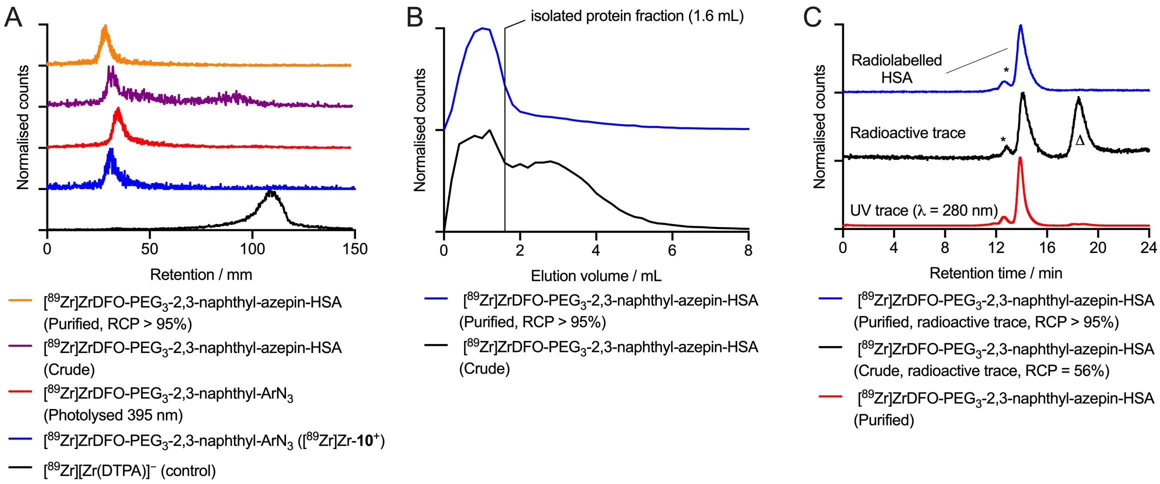

Figure S246. Characterization data for the radiochemical synthesis of [^{89}Zr]ZrDFO-PEG$_3$-2,3-naphthyl-azepin-HSA using DFO-PEG$_3$-2,3-naphthyl-ArN$_3$ (**10**). (A) Radio-iTLC chromatograms, (B) analytical PD-10-SEC profiles, and (C) SEC-HPLC chromatograms of the crude and purified product (*: aggregated protein, Δ: radiolabeled small molecules).

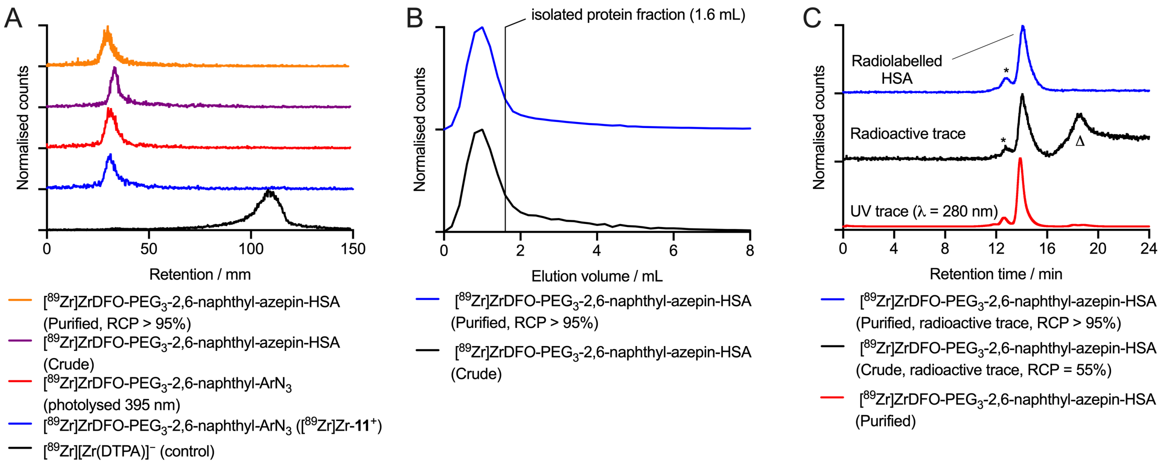

Figure S247. Characterization data for the radiochemical synthesis of [^{89}Zr]ZrDFO-PEG$_3$-2,6-naphthyl-HSA using DFO-PEG$_3$-2,6-naphthyl-ArN$_3$ (**11**). (A) Radio-iTLC chromatograms, (B) analytical PD-10-SEC profiles, and (C) SEC-HPLC chromatograms of the crude and purified product (*: aggregated protein, Δ: radiolabeled small molecules).

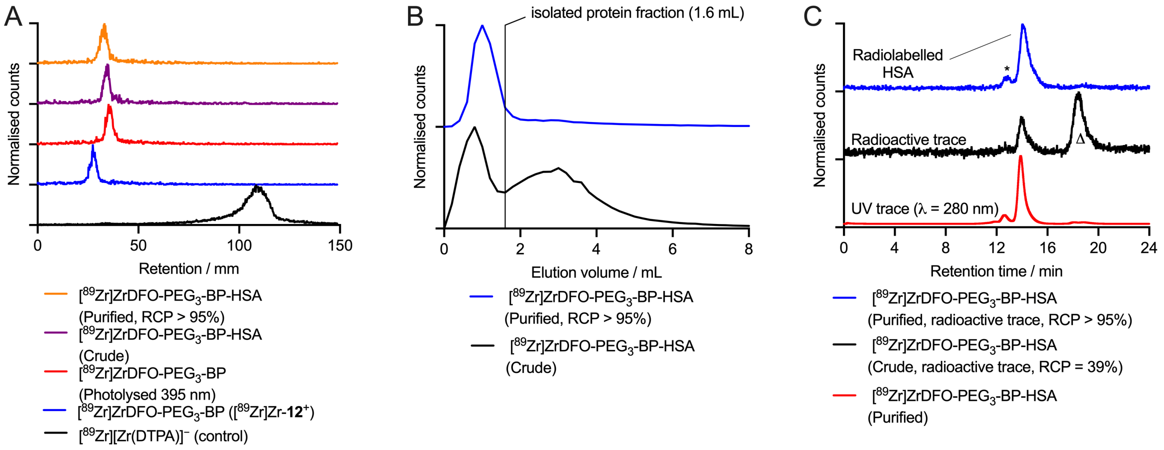

Figure S248. Characterization data for the radiochemical synthesis of [^{89}Zr]ZrDFO-PEG$_3$-BP-HSA using DFO-PEG$_3$-BP (**12**). (A) Radio-iTLC chromatograms, (B) analytical PD-10-SEC profiles, and (C) SEC-HPLC chromatograms of the crude and purified product (*: aggregated protein, Δ: radiolabeled small molecules).

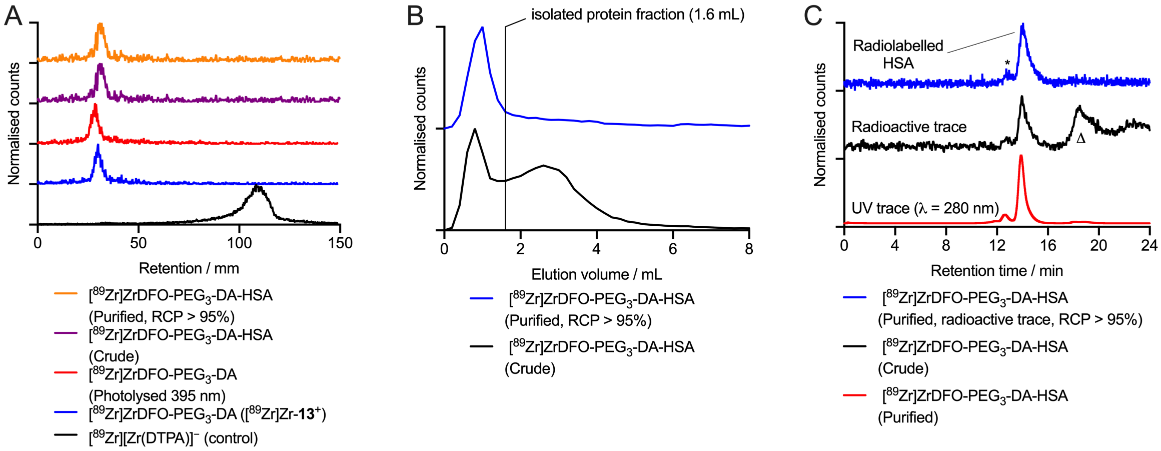

Figure S249. Characterization data for the radiochemical synthesis of [^{89}Zr]ZrDFO-PEG$_3$-DA-HSA using DFO-PEG$_3$-DA (**13**). (A) Radio-iTLC chromatograms, (B) analytical PD-10-SEC profiles, and (C) SEC-HPLC chromatograms of the crude and purified product (*: aggregated protein, Δ: radiolabeled small molecules).

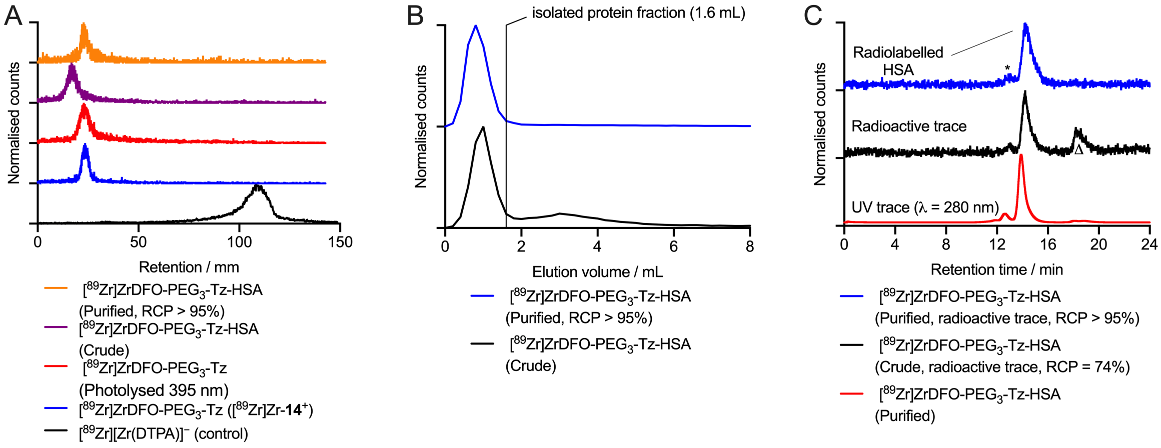

Figure S250. Characterization data for the radiochemical synthesis of [^{89}Zr]ZrDFO-PEG$_3$-Tz-HSA using DFO-PEG$_3$-Tz (**14**). (A) Radio-iTLC chromatograms, (B) analytical PD-10-SEC profiles, and (C) SEC-HPLC chromatograms of the crude and purified product (*: aggregated protein, Δ: radiolabeled small molecules).

Table S2. Experimentally measured decay-corrected isolated radiochemical yields (RCYs) for the photoradiosynthesis of ^{89}ZrDFO-PEG$_3$-HSA derivatives using compounds **1** to **14**.

**Compound**	Mean RCY / %	Standard deviation / %	Replicates (*n*)
**1**	62.3	3.6	5
**2**	58.6	1.7	3
**3**	50.8	3.5	3
**4**	48.0	2.9	3
**5**	43.5	1.2	3
**6**	42.2	3.1	3
**7**	26.6	0.6	3
**8**	40.3	1.4	3
**9**	18.1	1.8	3
**10**	45.5	2.5	3
**11**	49.0	1.2	3
**12**	29.6	2.8	3
**13**	24.3	2.3	3
**14**	61.8	4.9	3

Computational results

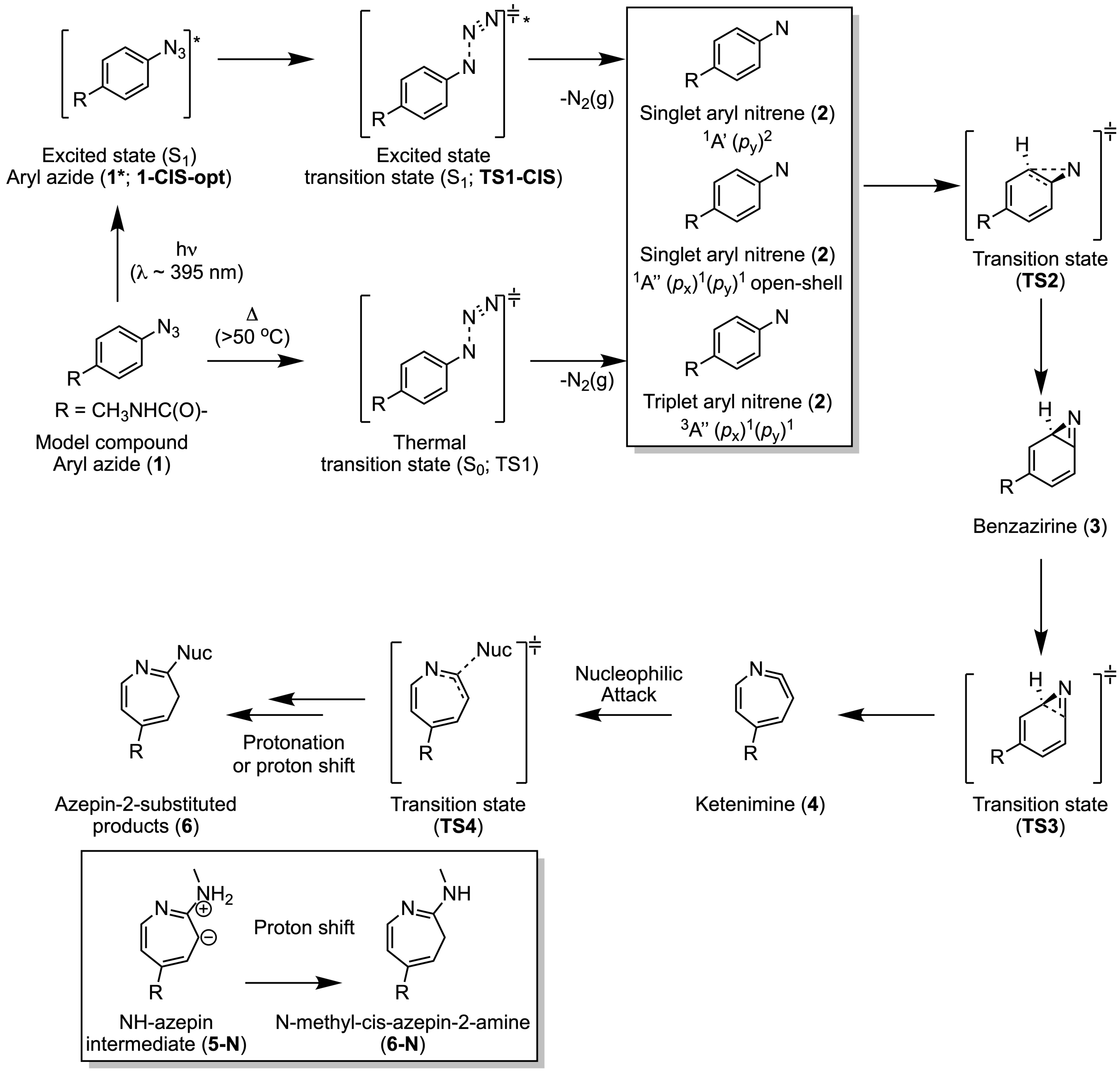

Figure S251. Proposed mechanism of photochemical and thermal activation, intramolecular rearrangement, and nucleophilic attack at model aryl azide compounds.

Table S3. Calculated energetics for the photo-initiated reaction of two model aryl azides with methylamine and acetate using the PW6B95/6-311++G(d,p) methodology with a PCM (water) solvation model.

	**Calculated relative energetics**											
**PW6B95/6-311++G(d,p)/PCM**	Phenylazide (PhN_3) (model **H**)			*p*-$CH_3C(O)NH$-ArN_3 (model inverted amide)			*p*-Me-ArN_3 (model **4**)			*p*-$CH_3NHC(O)$-ArN_3 (model **1**)		
**Species**	ΔG / kJ mol^{-1}	ΔH / kJ mol^{-1}	ΔS / J K^{-1} mol^{-1}	ΔG / kJ mol^{-1}	ΔH / kJ mol^{-1}	ΔS / J K^{-1} mol^{-1}	ΔG / kJ mol^{-1}	ΔH / kJ mol^{-1}	ΔS / J K^{-1} mol^{-1}	ΔG / kJ mol^{-1}	ΔH / kJ mol^{-1}	ΔS / J K^{-1} mol^{-1}
Ground state (S_0) potential energy surface												
Aryl azide (**1**)	0.0	0.0	0.0	0.0	0.0	0.0	0	0	0	0	0	0
TS1	155.4	161.5	20.4	148.0	151.1	10.1	151.4	156.2	16.2	156.4	163.5	23.6
Aryl nitrene (**2**) $^1A''$ state ($(p_y)^1(p_y)^1$; sum method) [a]	26.5	67.5	137.5	11.4	52.6	138.0	17.7	60.0	142.1	26.4	68.6	141.4
TS2	71.2	113.7	142.6	56.9	97.5	136.2	64.0	102.4	129.0	78.8	120.3	139.3
Benzazirine (**3**)	12.2	55.7	145.8	16.6	57.0	135.6	15.4	54.3	130.5	17.1	59.4	141.9
TS3	30.7	73.2	142.6	39.0	80.3	138.4	37.7	75.8	127.8	29.6	70.8	138.1
Ketenimine (**4**)	-0.6	43.5	147.9	3.7	45.5	140.3	4.0	43.2	131.6	6.7	50.3	146.2
TS4-$MeNH_2$	50.3	49.2	-4.0	47.9	46.7	-4.0	55.3	48.3	-23.4	47.7	48.4	2.4
NH-azepin intermediate (**5-N**)	20.9	15.3	-18.8	14.1	5.1	-29.9	22.8	12.6	-34.1	2.8	-5.1	-26.5
N-methyl-*cis*-azepin-2-amine (**6-N**)	-176.9	-184.7	-26.1	-179.0	-188.5	-31.6	-172.8	-185.5	-42.6	-176.1	-185.1	-30.2
Δ (TS2 – $^1A''$ nitrene)	44.7	46.2	5.1	45.5	45.0	-1.9	46.3	42.4	-13.1	52.3	51.7	-2.1
Δ (TS3 – Benzazirine **3**)	18.5	17.5	-3.2	22.4	23.2	2.8	22.3	21.5	-2.7	12.5	11.4	-3.8
Δ (TS4 – Ketenimine **4**)	50.9	5.7	-151.9	44.2	1.2	-144.4	51.3	5.1	-155.0	41.0	-1.9	-143.7
Excited state (S_1) potential energy surface: CIS calculations												
Aryl azide (**1-CIS; 1***) (optimized S_1 state; root=1)	200.7	200.3	-1.5	188.4	184.9	-11.8	192.5	192.1	-1.2	202.7	204.1	4.6
**TS1-CIS** (optimized TS for N_2 loss on S_1 surface; root = 1)	207.0	206.7	-1.0	194.1	192.5	-5.5	204.8	207.9	10.5	204.3	205.4	3.7
*Alternative arylnitrene (**2**) spin states*												
$^3A''$ state, open shell $(p_y)^1(p_y)^1$	-50.4	-4.8	153.0	-58.8	-13.2	152.9	-56.8	-9.6	158.5	-48.5	-1.8	156.5
$^1A'$ state, closed shell, $(p_y)^2$	60.6	106.5	153.7	27.7	70.6	144.2	45.1	89.8	149.9	135.1	182.3	158.4

$^1A''$ state, open shell $(p_y)^1(p_y)^1$ [b]	-11.9	31.4	145.2	-23.7	19.7	145.5	-19.6	25.2	150.3	-11.0	33.4	148.9
$^1A''$ annihilated spin expectation value, $\langle S^2A \rangle$ [c]	0.234			0.182			0.217			0.237		

[a] Energies of the $^1A''$ state were recalculated using the sum method described by Ziegler *et al*. where $E(S_1) = 2 \times E(50{:}50) - E(T_0)$.[4]

[b] Note: relative energies for aryl nitrene (**2**) are taken from the DFT calculations whereby the wavefunction is spin contaminated $\langle S^2 \rangle = 1.0$. These numbers correspond to the values of $E(50{:}50)$.

[c] The spin-annihilation method reduced the $\langle S^2 \rangle$ expectation value.

Table S4. Calculated energetics for the photo-initiated reaction of two model aryl azides with methylamine and acetate using the PW6B95/6-311++G(d,p) methodology with a PCM (water) solvation model.

	**Calculated relative energetics**											
**PW6B95/6-311++G(d,p)/PCM**	*m*-$CH_3NHC(O)$-ArN_3 (model **2**)			*o*-$CH_3NHC(O)$-ArN_3 (model **3**)			4F-*p*-ArN_3 (model **8**)			o-MeO-*p*-ArN_3 (model **5**)		
**Species**	ΔG / kJ mol^{-1}	ΔH / kJ mol^{-1}	ΔS / J K^{-1} mol^{-1}	ΔG / kJ mol^{-1}	ΔH / kJ mol^{-1}	ΔS / J K^{-1} mol^{-1}	ΔG / kJ mol^{-1}	ΔH / kJ mol^{-1}	ΔS / J K^{-1} mol^{-1}	ΔG / kJ mol^{-1}	ΔH / kJ mol^{-1}	ΔS / J K^{-1} mol^{-1}
Ground state (S_0) potential energy surface												
Aryl azide (**1**)	0.0	0.0	0.0	0.0	0.0	0.0	0	0	0	0	0	0
TS1	154.9	161.1	21.0	134.7	136.3	5.4	143.8	150.5	22.5	181.4	187.7	21.2
Aryl nitrene (**2**) $^1A''$ state ($(p_y)^1(p_y)^1$; sum method) [a]	27.9	71.3	145.5	12.1	52.2	134.7	32.0	73.0	137.6	54.2	98.4	148.1
Attack on the side away (a) from the substituent												
TS2a	76.3	119.5	144.9	57.9	100.5	142.7	85.6	131.4	153.6	101.0	148.2	158.4
Benzazirine (**3a**)	15.6	58.6	144.2	-0.2	43.7	147.2	-10.7	33.4	147.8	36.4	79.5	144.6
TS3a	29.3	70.8	139.2	10.7	56.8	154.8	29.1	70.4	138.6	62.2	104.0	140.3
Ketenimine (**4a**)	-8.8	34.3	144.6	-22.3	21.7	147.9	-14.9	29.5	148.9	17.3	62.0	149.7
TS4a-$MeNH_2$	35.6	34.6	-3.4	24.6	24.9	1.0	17.2	17.1	-0.1	65.4	62.4	-9.9
NH-azepin intermediate (**5a**)	0.2	-6.5	-22.7	-49.9	-62.7	-42.9	-84.2	-92.1	-26.4	6.3	0.3	-20.2
N-methyl-*cis*-azepin-2-amine (**6a**)	-184.8	-193.2	-28.2	-175.9	-186.8	-36.5	-239.8 ^{X}<br>-237.4 ^{Y}	-248.5 ^{X}<br>-246.8 ^{Y}	-29.0 ^{X}<br>-31.6 ^{Y}	-166.3	-174.5	-27.5
Δ (TS2a – $^1A''$ nitrene)	48.4	48.2	-0.5	45.8	48.2	8.0	53.6	58.4	16.1	46.8	49.8	10.3
Δ (TS3a – Benzazirine **3a**)	13.7	12.3	-5.0	10.9	13.1	7.6	39.8	37.0	-9.2	25.8	24.5	-4.4
Δ (TS4a – Ketenimine **4a**)	44.4	0.3	-148.0	46.9	3.1	-146.9	32.0	-12.4	-148.9	48.0	0.5	-159.6
Attack on the substiuent side (b) toward the substituent												
TS2b	72.0	113.6	139.5	60.5	99.5	130.6	N/A	N/A	N/A	78.5	121.6	144.4
Benzazirine (**3b**)	5.2	47.9	143.3	-8.3	35.1	145.5	N/A	N/A	N/A	21.4	64.1	143.3
TS3b	32.5	73.6	138.0	16.5	57.2	136.5	N/A	N/A	N/A	54.5	95.3	136.7
Ketenimine (**4b**)	6.7	49.9	144.9	-8.8	34.4	145.1	N/A	N/A	N/A	31.3	77.1	153.7
TS4b-$MeNH_2$	43.2	43.6	1.2	35.5	34.3	-4.1	N/A	N/A	N/A	72.0	74.5	8.5
NH-azepin intermediate (**5b**)	-7.4	-15.4	-26.8	6.8	1.1	-19.1	N/A	N/A	N/A	25.1	19.2	-19.7
N-methyl-*cis*-azepin-2-amine (**6b**)	-183.4	-192.7	-31.4	-189.2	-197.7	-28.6	N/A	N/A	N/A	-154.2	-161.4	-24.0
Δ (TS2b – $^1A''$ nitrene)	44.1	42.3	-5.9	48.5	47.2	-4.2	N/A	N/A	N/A	24.3	23.2	-3.7

Δ (TS3b – Benzazirine **3b**)	27.3	25.7	-5.4	24.8	22.1	-9.1				33.1	31.1	-6.6
Δ (TS4b – Ketenimine **4b**)	36.5	-6.3	-143.7	44.4	-0.1	-149.2				40.7	-2.6	-145.2
Excited state (S_1) potential energy surface: CIS calculations												
Aryl azide (**1-CIS; 1***) (optimized S_1 state; root=1)	203.5	204.0	1.7	203.0	204.2	4.1	215.2	214.8	-1.5	201.8	202.0	0.7
**TS1-CIS** (optimized TS for N_2 loss on S_1 surface; root = 1)	203.5	204.0	1.7	206.0	207.4	4.6	214.5	213.2	-4.3	203.6	203.8	0.6
*Alternative arylnitrene (**2**) spin states*												
$^3A''$ state, open shell $(p_y)^1(p_y)^1$	-49.6	-2.1	159.3	-62.4	-14.1	162.0	-48.5	-0.3	161.8	-22.9	25.9	163.4
$^1A'$ state, closed shell, $(p_y)^2$	144.2	191.0	157.2	42.9	87.2	148.6	51.0	98.3	158.8	99.2	145.7	156.1
$^1A''$ state, open shell $(p_y)^1(p_y)^1$ [b]	-10.8	34.6	152.4	-25.2	19.1	148.4	-8.3	36.3	149.7	15.7	62.1	155.8
$^1A''$ annihilated spin expectation value, $\langle S^2A \rangle$ [c]	0.245			0.224			0.244			0.243		

[a] Energies of the $^1A''$ state were recalculated using the sum method described by Ziegler *et al*. where $E(S_1) = 2 \times E(50{:}50) - E(T_0)$.[4]

[b] Note: relative energies for aryl nitrene (**2**) are taken from the DFT calculations whereby the wavefunction is spin contaminated $\langle S^2 \rangle = 1.0$. These numbers correspond to the values of $E(50{:}50)$.

[c] The spin-annihilation method reduced the $\langle S^2 \rangle$ expectation value.

[X] F atom in an equatorial position. [Y] F atom in an axial position

Table S5. Calculated energetics for the photo-initiated reaction of two model aryl azides with methylamine and acetate using the PW6B95/6-311++G(d,p) methodology with a PCM (water) solvation model.

	**Calculated relative energetics**											
**PW6B95/6-311++G(d,p)/PCM**	o-Cl-*p*-ArN_3 (model **6**)			o-NO_2-*p*-ArN_3 (model **7**)			2,3-naphthyl-N_3 (model **10**)			2,6-naphthyl-N_3 (model **11**)		
**Species**	ΔG / kJ mol^{-1}	ΔH / kJ mol^{-1}	ΔS / J K^{-1} mol^{-1}	ΔG / kJ mol^{-1}	ΔH / kJ mol^{-1}	ΔS / J K^{-1} mol^{-1}	ΔG / kJ mol^{-1}	ΔH / kJ mol^{-1}	ΔS / J K^{-1} mol^{-1}	ΔG / kJ mol^{-1}	ΔH / kJ mol^{-1}	ΔS / J K^{-1} mol^{-1}
Ground state (S_0) potential energy surface												
Aryl azide (**1**)	0.0	0.0	0.0	0.0	0.0	0.0	0	0	0	0	0	0
TS1	158.6	164.6	20.3	154.4	161.4	23.3	139.8	149.2	31.6	146.8	150.4	12.1
Aryl nitrene (**2**) $^1A''$ state $((p_y)^1(p_y)^1$; sum method) [a]	16.8	46.9	100.8	31.0	75.6	149.6	17.7	67.2	166.0	17.4	54.7	125.2
Attack on the side away (a) from the substituent												
TS2a	80.7	123.6	143.9	84.2	126.1	140.5	22.5	73.7	171.7	No stationary point found		
Benzazirine (**3a**)	19.3	64.4	151.3	24.8	67.0	141.3	-33.8	18.2	174.5	No stationary point found		
TS3a	34.5	77.0	142.7	35.4	76.6	138.0	39.6	90.2	169.8	No stationary point found		
Ketenimine (**4a**)	-8.0	35.9	147.1	-3.1	41.9	150.7	28.0	80.4	175.8	-32.4	11.4	147.0
TS4a-MeNH_2	33.9	37.5	12.2	33.6	28.3	-17.8	77.0	83.2	20.9	19.1	17.3	-6.2
NH-azepin intermediate (**5a**)	-14.7	-20.4	-19.1	-69.0	-82.7	-46.0	-14.2	-15.7	-5.1	-53.4	-59.9	-21.6
N-methyl-*cis*-azepin-2-amine (**6a**)	-189.6	-196.7	-23.9	-209.7	-217.7	-26.8	-140.2	-140.7	-1.6	-214.7	-222.7	-26.8
Δ (TS2a – $^1A''$ nitrene)	63.9	76.7	43.0	53.1	50.4	-9.1	4.8	6.5	5.7	No stationary point found		
Δ (TS3a – Benzazirine **3a**)	15.2	12.7	-8.6	10.6	9.6	-3.3	73.4	72.0	-4.7	No stationary point found		
Δ (TS4a – Ketenimine **4a**)	41.9	1.7	-134.9	36.6	-13.6	-168.5	49.0	2.8	-155.0	51.6	5.9	-153.2
Attack on the substiuent side (b) toward the substituent												
TS2b	80.0	122.6	142.9	81.1	124.5	145.6	102.6	152.1	166.0	57.3	100.4	144.8
Benzazirine (**3b**)	5.9	50.3	149.0	7.6	52.0	148.9	29.3	80.2	170.4	-22.4	21.3	146.7
TS3b	30.1	72.8	143.3	29.1	68.7	132.9	30.3	79.1	163.8	52.0	94.6	142.9
Ketenimine (**4b**)	8.0	51.0	144.4	-4.0	37.9	140.6	-41.9	10.1	174.6	37.7	81.6	147.2
TS4b-MeNH_2	42.8	44.5	5.6	25.2	11.9	-44.7	-2.7	8.3	36.8	84.6	86.5	6.5
NH-azepin intermediate (**5b**)	-12.4	-18.8	-21.5	-91.3	-102.2	-36.6	-59.3	-56.6	9.0	-7.5	-11.8	-14.3
N-methyl-*cis*-azepin-2-amine (**6b**)	-187.5	-196.6	-30.6	-225.7	-235.6	-33.3	-223.5	-223.4	0.4	-141.8	-149.3	-25.3
Δ (TS2b – $^1A''$ nitrene)	63.2	75.8	42.1	50.1	48.9	-4.0	84.8	84.8	0.0	39.9	45.7	19.6
Δ (TS3b – Benzazirine **3b**)	24.2	22.5	-5.7	21.5	16.7	-16.1	0.9	-1.1	-6.6	74.4	73.3	-3.7
Δ (TS4b – Ketenimine **4b**)	34.9	-6.5	-138.8	29.2	-26.0	-185.3	39.2	-1.9	-137.7	46.9	4.9	-140.7
Excited state (S_1) potential energy surface: CIS calculations												

Aryl azide (**1-CIS; 1***) (optimized S_1 state; root=1)	207.1	207.7	1.9	212.2	213.3	3.6	227.7	234.4	22.5	194.9	194.9	-0.1
**TS1-CIS** (optimized TS for N_2 loss on S_1 surface; root = 1)	207.5	207.8	1.1	210.1	212.4	7.7	251.5	246.9	-15.2	198.6	192.6	-20.0
*Alternative arylnitrene (**2**) spin states*												
$^3A''$ state, open shell $(p_y)^1(p_y)^1$	-44.6	-0.6	147.5	-46.9	0.9	160.5	-66.8	-12.2	183.2	-52.1	-10.4	139.8
$^1A'$ state, closed shell, $(p_y)^2$	72.1	120.8	163.4	78.9	125.8	157.0	Note [d]			Note [e]		
$^1A''$ state, open shell $(p_y)^1(p_y)^1$ [b]	-13.9	23.1	124.2	-7.9	38.3	155.1	-24.5	27.5	174.6	-17.3	22.2	132.5
$^1A''$ annihilated spin expectation value, $<S^2A>$ [c]	0.252			0.258			0.228					

[a] Energies of the $^1A''$ state were recalculated using the sum method described by Ziegler *et al*. where $E(S_1) = 2 \times E(50:50) - E(T_0)$.[4]

[b] Note: relative energies for aryl nitrene (**2**) are taken from the DFT calculations whereby the wavefunction is spin contaminated $<S^2> = 1.0$. These numbers correspond to the values of $E(50:50)$.

[c] The spin-annihilation method reduced the $<S^2>$ expectation value.

[d] Attempts to optimise the structure of the closed shell $^1A_1$ state for this nitrene led to the formation of TS2a along the pathway to the formation of the benzazirine bicycle.

[e] Attempts to optimize the structure of the closed shell $^1A_1$ state for this nitrene led to the direct formation of the ring opened ketenimine structure **4a**. No transition states for the nitrene to Benzazirine formation (TS2a) were found.

Table S6. Calculated energetics for the photo-initiated reaction of two model aryl azides with methylamine and acetate using the PW6B95/6-311++G(d,p) methodology with a PCM (water) solvation model.

	**Calculated relative energetics**		
**PW6B95/6-311++G(d,p)/PCM**	2,4-pyridyl-N_3 (model **9**)		
**Species**	Δ*G* / kJ mol^{-1}	Δ*H* / kJ mol^{-1}	Δ*S* / J K^{-1} mol^{-1}
Ground state (S_0) potential energy surface			
Aryl azide (**1**)	0.0	0.0	0.0
TS1	158.1	165.2	23.8
Aryl nitrene (**2**) $^1A''$ state ($(p_y)^1(p_y)^1$; sum method) [a]	51.9	94.1	141.5
Attack on the side away (a) from the substituent			
TS2a	108.2	149.5	138.5
Benzazirine (**3a**)	27.9	70.8	144.0
TS3a	37.2	80.1	143.9
Ketenimine (**4a**)	10.2	54.9	150.0
TS4a-$MeNH_2$	47.9	52.4	15.1
NH-azepin intermediate (**5a**)	-4.6	-10.9	-21.2
N-methyl-*cis*-azepin-2-amine (**6a**)	-193.4	-200.9	-25.4
Δ (TS2a – $^1A''$ nitrene)	56.3	55.4	-3.0
Δ (TS3a – Benzazirine **3a**)	9.3	9.3	-0.1
Δ (TS4a – Ketenimine **4a**)	37.7	-2.5	-134.9
Attack on the substiuent side (b) toward the substituent			
TS2b	108.2	149.5	138.6
Benzazirine (**3b**)	28.6	72.3	146.4
TS3b	41.4	82.8	138.7
Ketenimine (**4b**)	10.6	53.2	142.8
TS4b-$MeNH_2$	47.8	40.9	-22.9
NH-azepin intermediate (**5b**)	-15.7	-24.1	-28.1
N-methyl-*cis*-azepin-2-amine (**6b**)	-194.5	-204.5	-33.7
Δ (TS2b – $^1A''$ nitrene)	56.3	55.4	-3.0

Δ (TS3b – Benzazirine **3b**)	12.8	10.5	-7.7
Δ (TS4b – Ketenimine **4b**)	37.2	-12.2	-165.7
Excited state (S_1) potential energy surface: CIS calculations			
Aryl azide (**1-CIS; 1***) (optimized S_1 state; root=1)	231.5	226.1	-18.1
**TS1-CIS** (optimized TS for N_2 loss on S_1 surface; root = 1)	225.3	226.4	3.5
*Alternative arylnitrene (**2**) spin states*			
$^3A''$ state, open shell $(p_y)^1(p_y)^1$	-32.4	14.1	155.8
$^1A'$ state, closed shell, $(p_y)^2$	129.4	176.2	157.0
$^1A''$ state, open shell $(p_y)^1(p_y)^1$ [b]	9.8	54.1	148.6
$^1A''$ annihilated spin expectation value, <S^2A> [c]	0.270		

[a] Energies of the $^1A''$ state were recalculated using the sum method described by Ziegler *et al*. where $E(S_1) = 2 \times E(50{:}50) - E(T_0)$.[4]

[b] Note: relative energies for aryl nitrene (**2**) are taken from the DFT calculations whereby the wavefunction is spin contaminated <S^2> = 1.0. These numbers correspond to the values of *E*(50:50).

[c] The spin-annihilation method reduced the <S^2> expectation value.

Note: In Figures S252 – S272 below, the electronic configurations $^1A_2$ (open shell singlet) and $^3A_2$ (open shell triplet) apply to pseudo-C_{2v} symmetry. The equivalent representations in pseudo-C_s symmetry are ^{1}A'' and ^{3}A''. All calculations were performed without symmetry constraints, but the symmetry labels are retained for simplicity in identifying the different electronic states of the nitrene. Caution should be used when comparing the number of the calculated S_1 species with any experimental data since the DFT/CIS methodology used provides an incomplete description of the complexity of the electronically excited state species.

Figure S252. DFT calculated (PW6B95/6-311++G(d,p)/PCM) reaction coordinate showing the reaction of unsubstituted phenylazide, PhN_3 (non-substituted model **H**) with methylamine ($MeNH_2$).

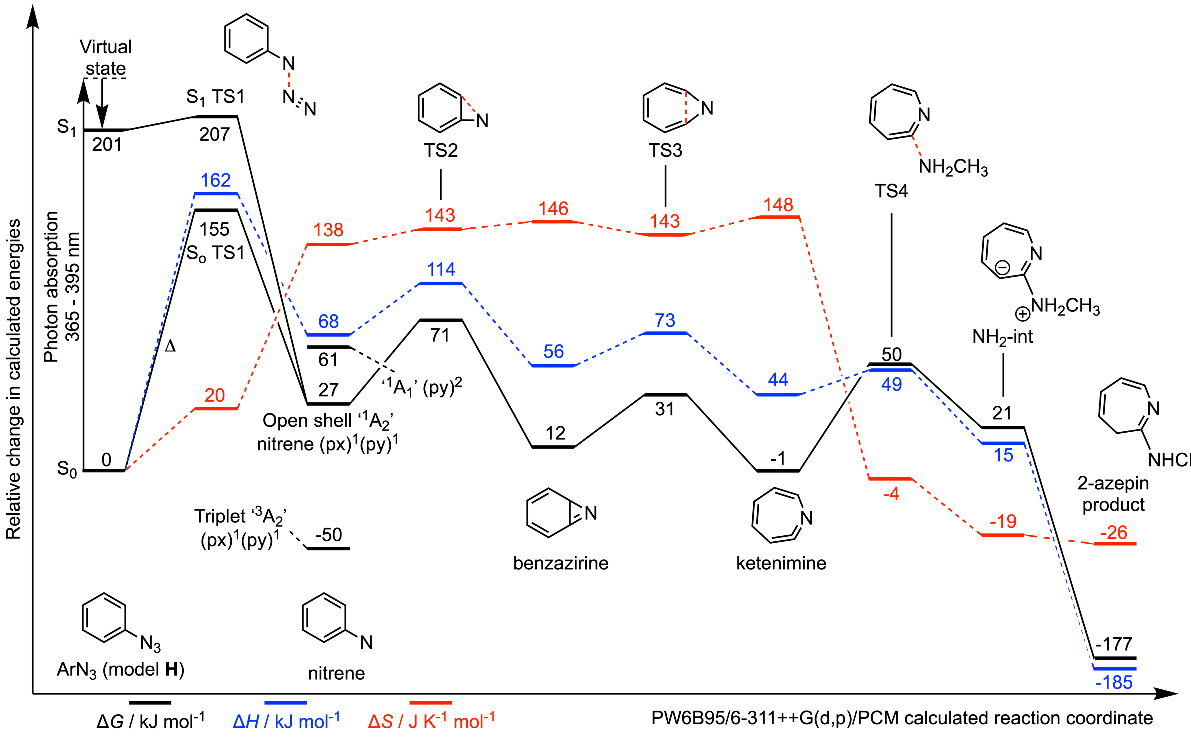

Figure S253. DFT calculated (PW6B95/6-311++G(d,p)/PCM) reaction coordinate showing the reaction of $CH_3C(O)NH$-ArN_3 aryl azide (model inverted amide) with methylamine.

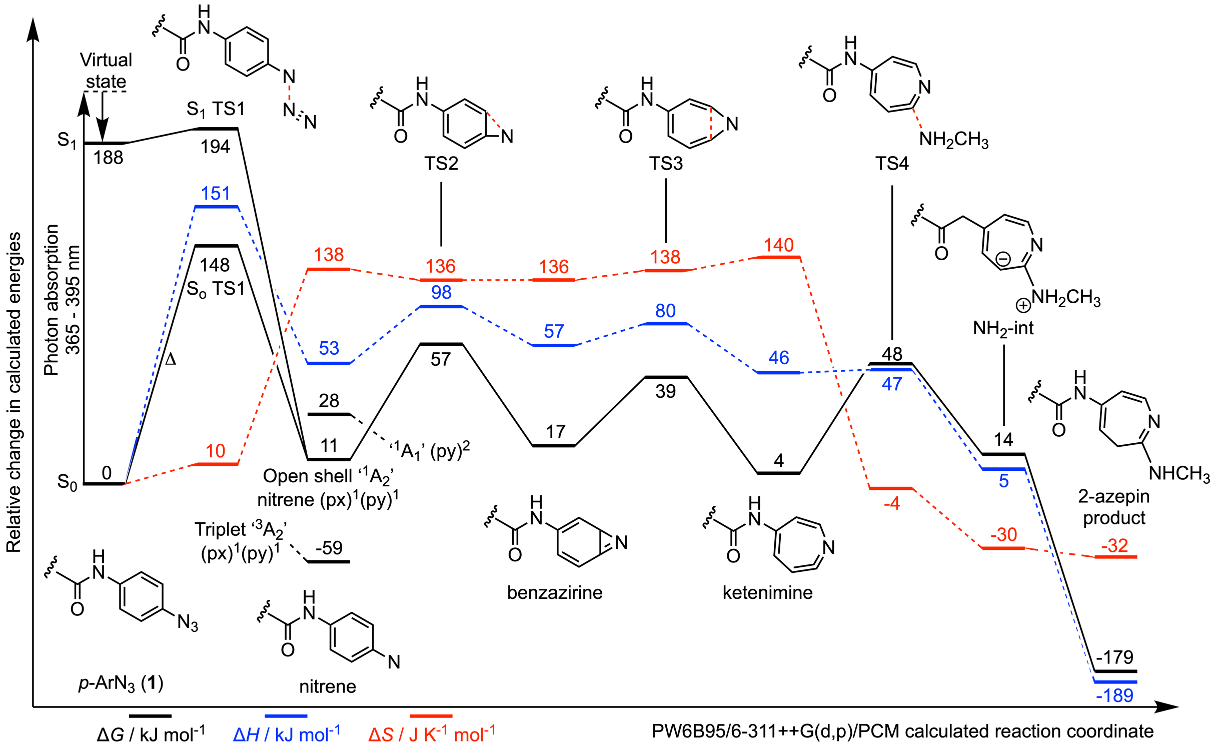

Figure S254. DFT calculated (PW6B95/6-311++G(d,p)/PCM) reaction coordinate showing the reaction of *para*-ArN3 (model **1**) with methylamine.

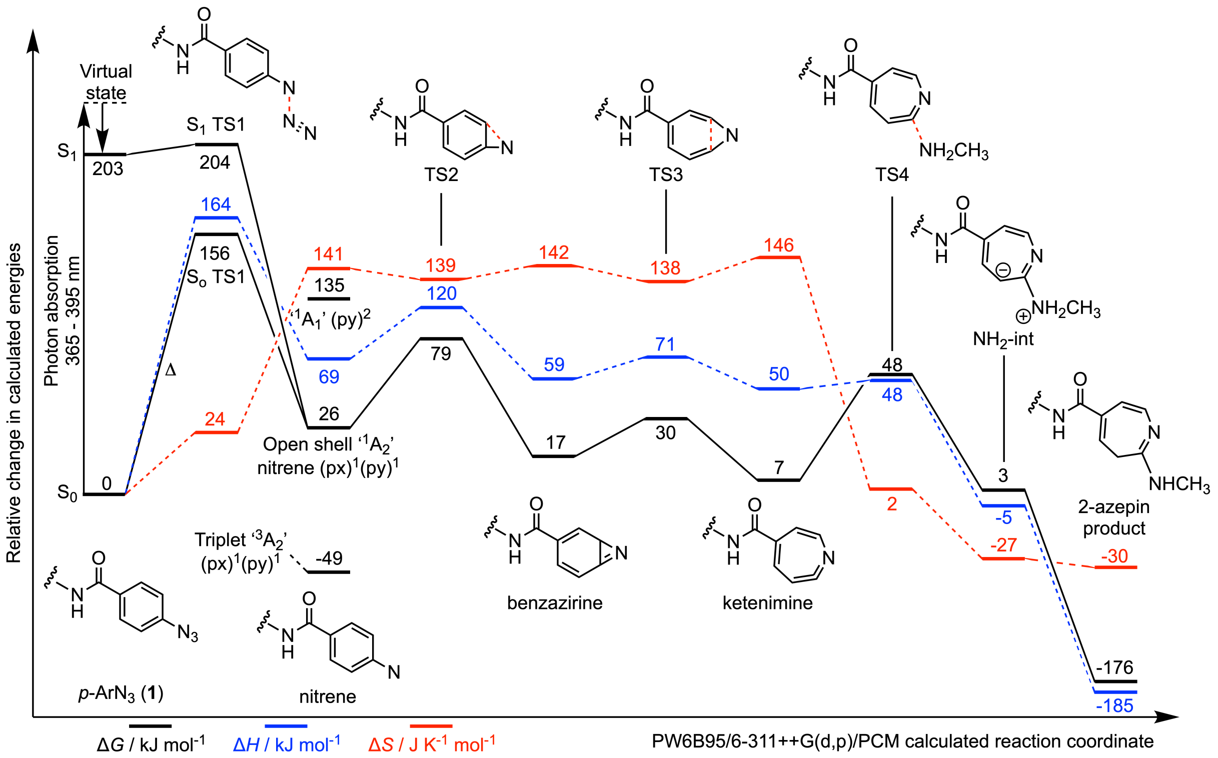

Figure S255. DFT calculated (PW6B95/6-311++G(d,p)/PCM) reaction coordinate showing the reaction of *meta*-ArN3 (model **2** pathway a) with methylamine.

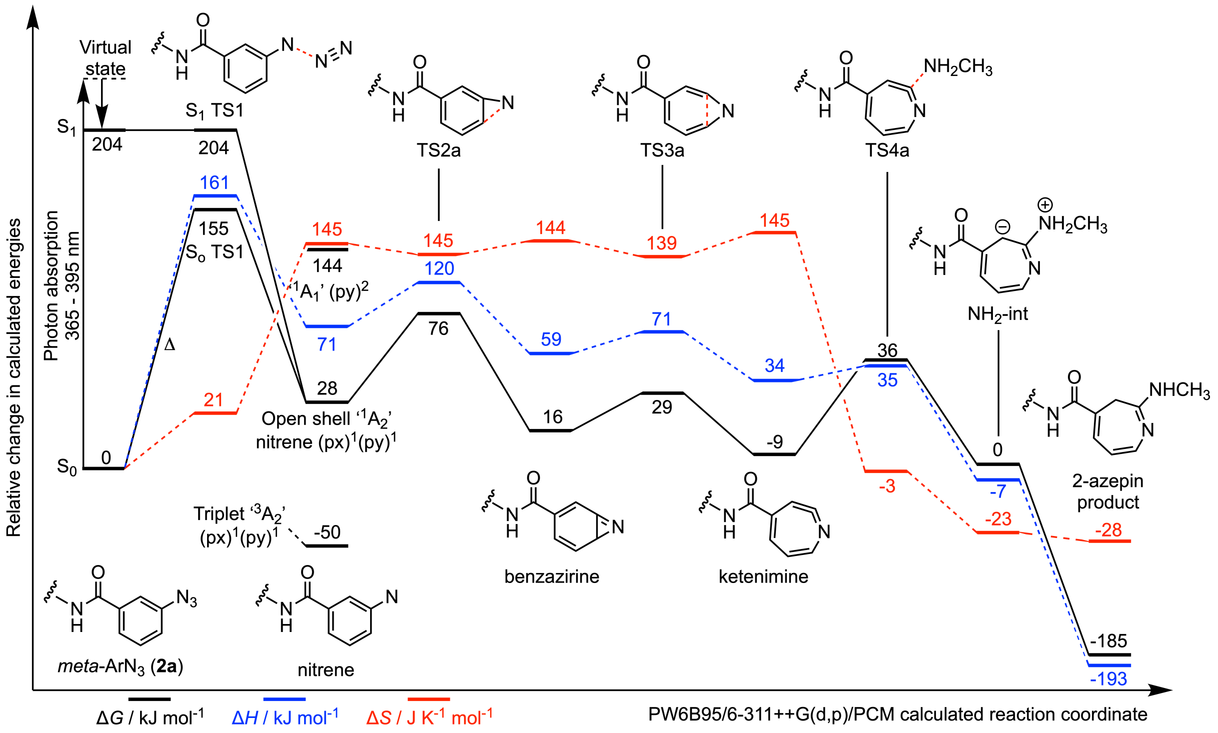

Figure S256. DFT calculated (PW6B95/6-311++G(d,p)/PCM) reaction coordinate showing the reaction of *meta*-ArN3 (model **2** pathway b) with methylamine.

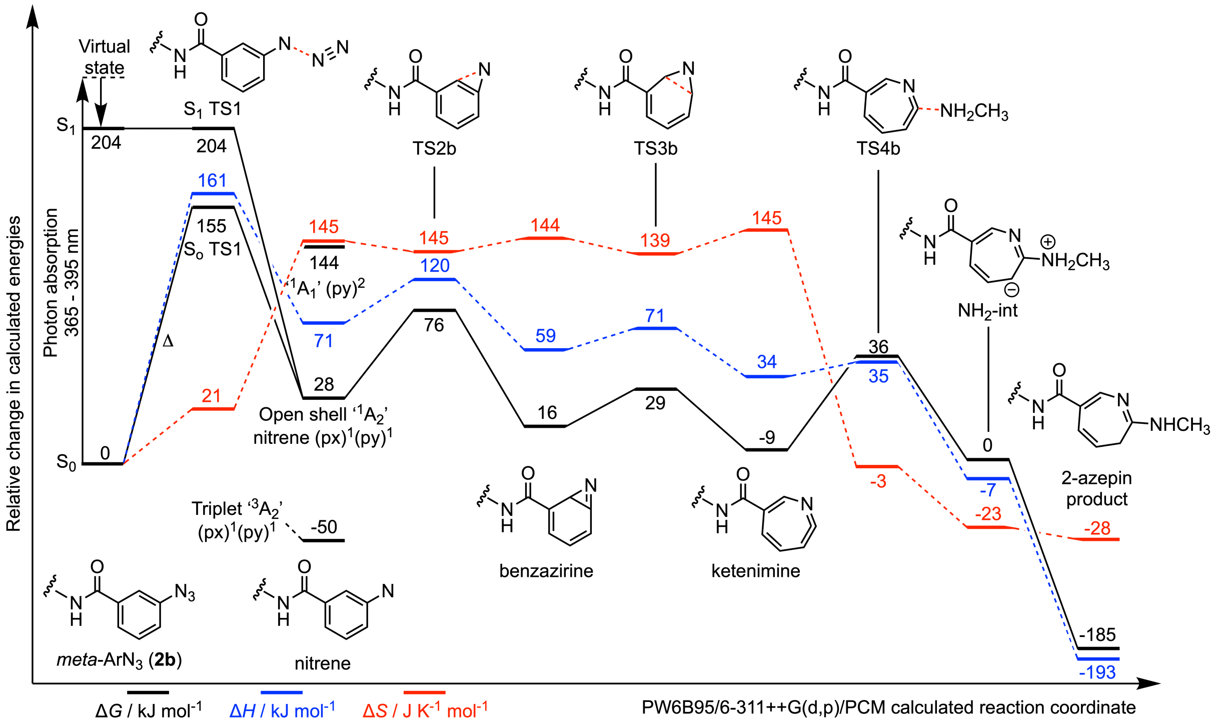

Figure S257. DFT calculated (PW6B95/6-311++G(d,p)/PCM) reaction coordinate showing the reaction of *ortho*-ArN3 (model **3** pathway a) with methylamine.

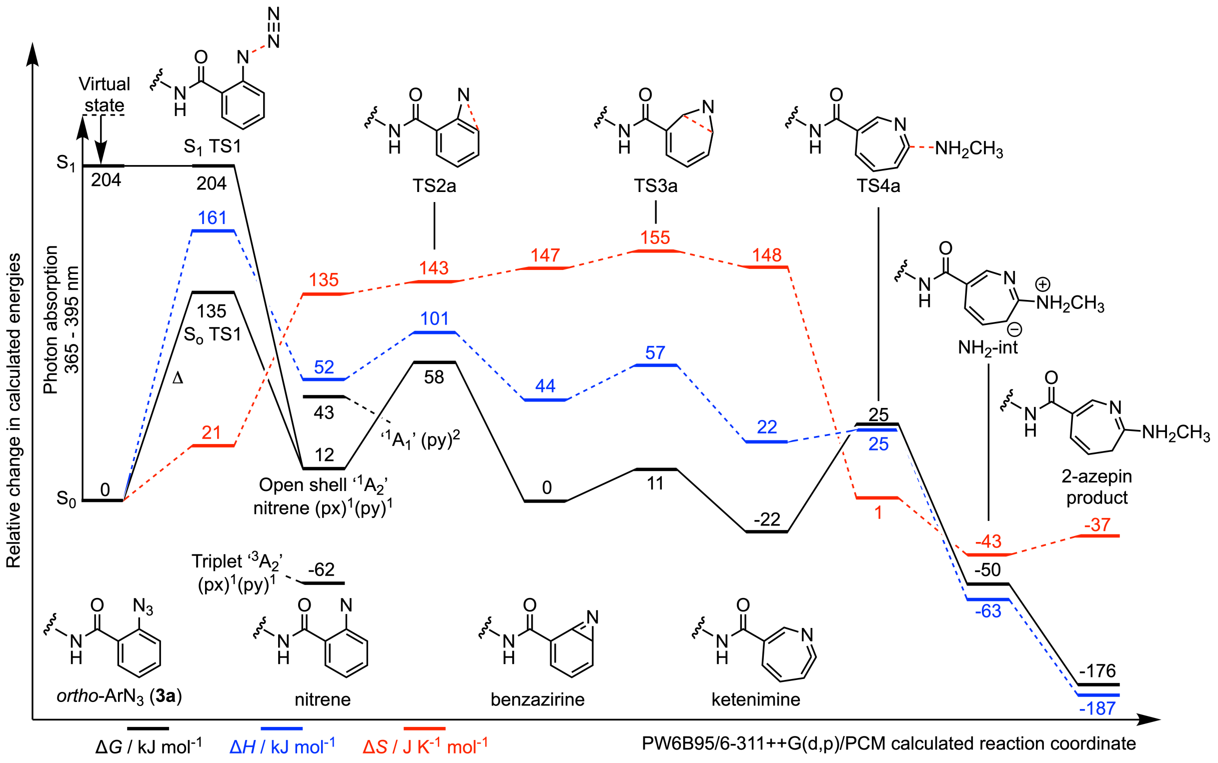

Figure S258. DFT calculated (PW6B95/6-311++G(d,p)/PCM) reaction coordinate showing the reaction of *ortho*-ArN₃ (model **3** pathway b) with methylamine.

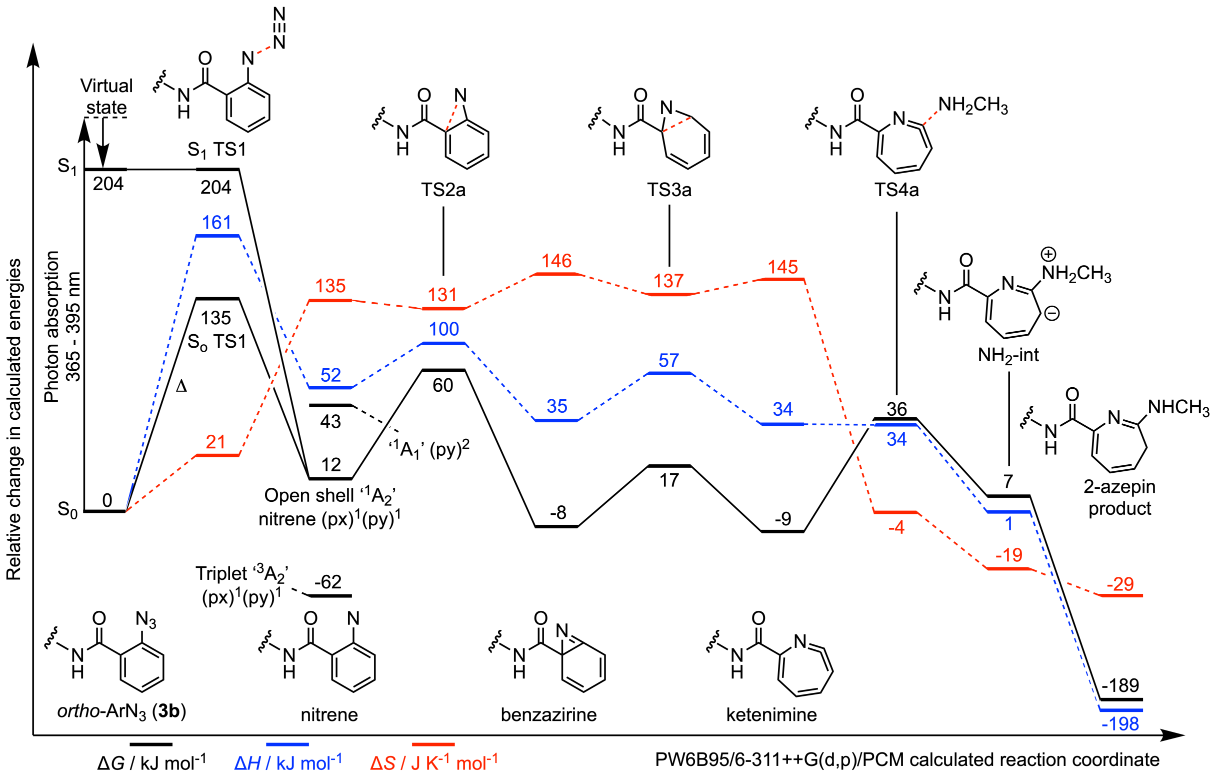

Figure S259. DFT calculated (PW6B95/6-311++G(d,p)/PCM) reaction coordinate showing the reaction of *para*-EtArN_3 (model **4**) with methylamine.

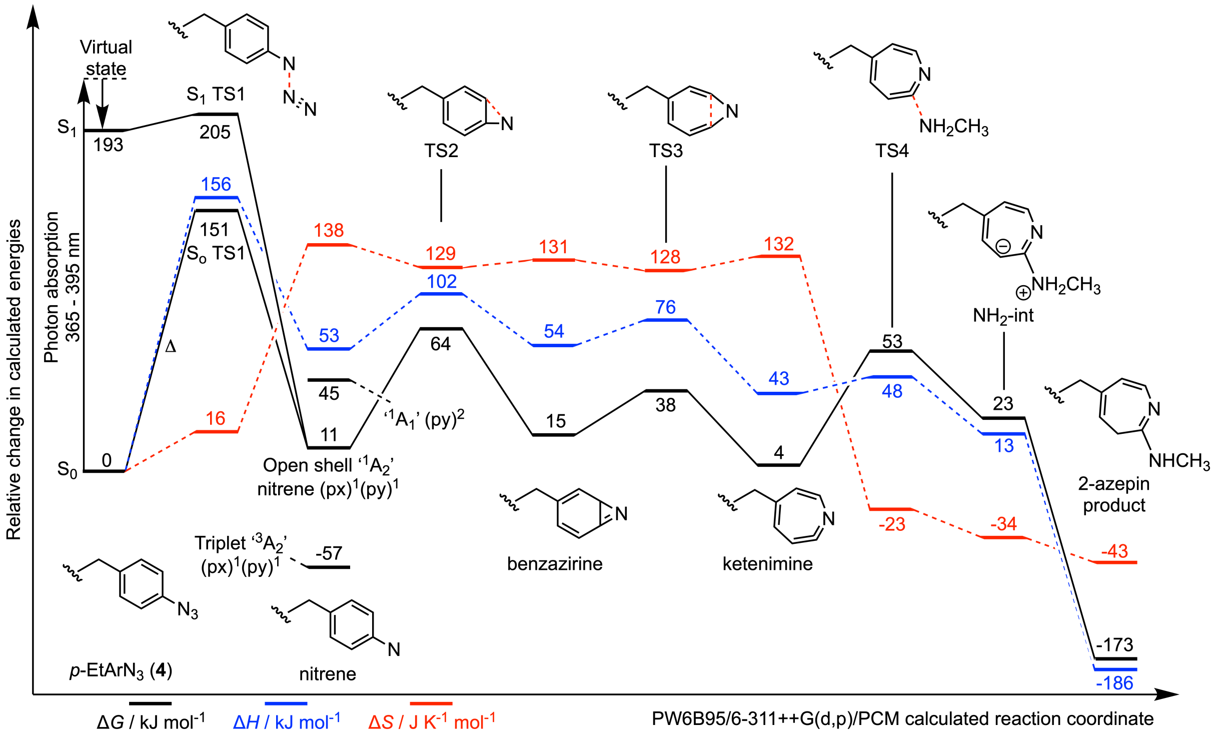

Note: for this model DFT compound the structure was truncated by splitting the C–C bond of the ethylene unit and replacing with H creating *p*-azidotoluene.

Figure S260. DFT calculated (PW6B95/6-311++G(d,p)/PCM) reaction coordinate showing the reaction of *ortho*-MeO-ArN3 (model **5** pathway a) with methylamine.

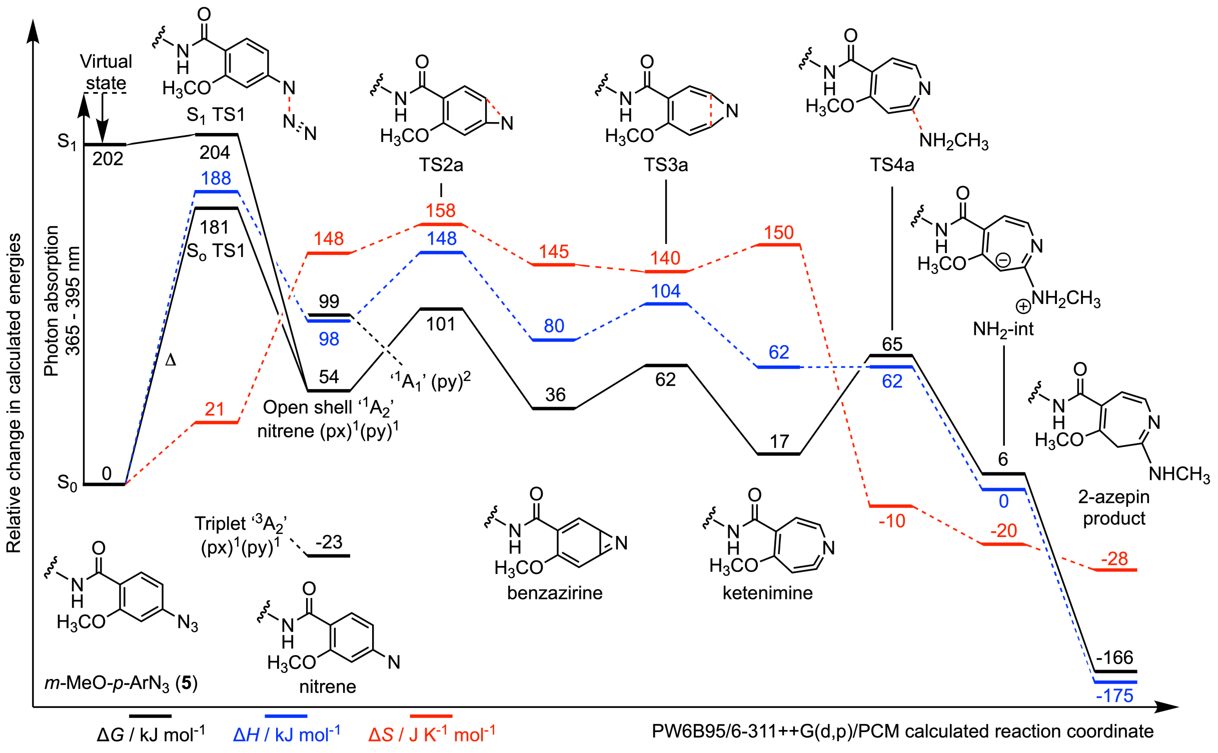

Figure S261. DFT calculated (PW6B95/6-311++G(d,p)/PCM) reaction coordinate showing the reaction of *ortho*-MeO-ArN3 (model **5** pathway b) with methylamine.

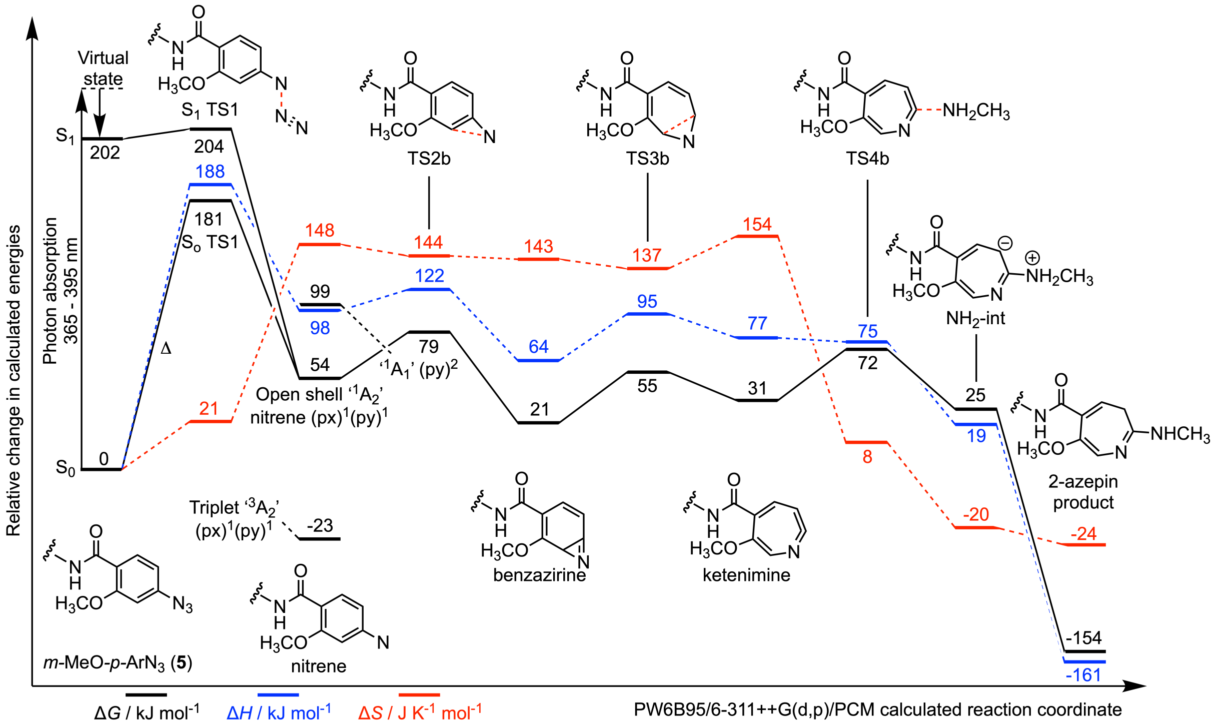

Figure S262. DFT calculated (PW6B95/6-311++G(d,p)/PCM) reaction coordinate showing the reaction of *ortho*-Cl-ArN3 (model **6** pathway a) with methylamine.

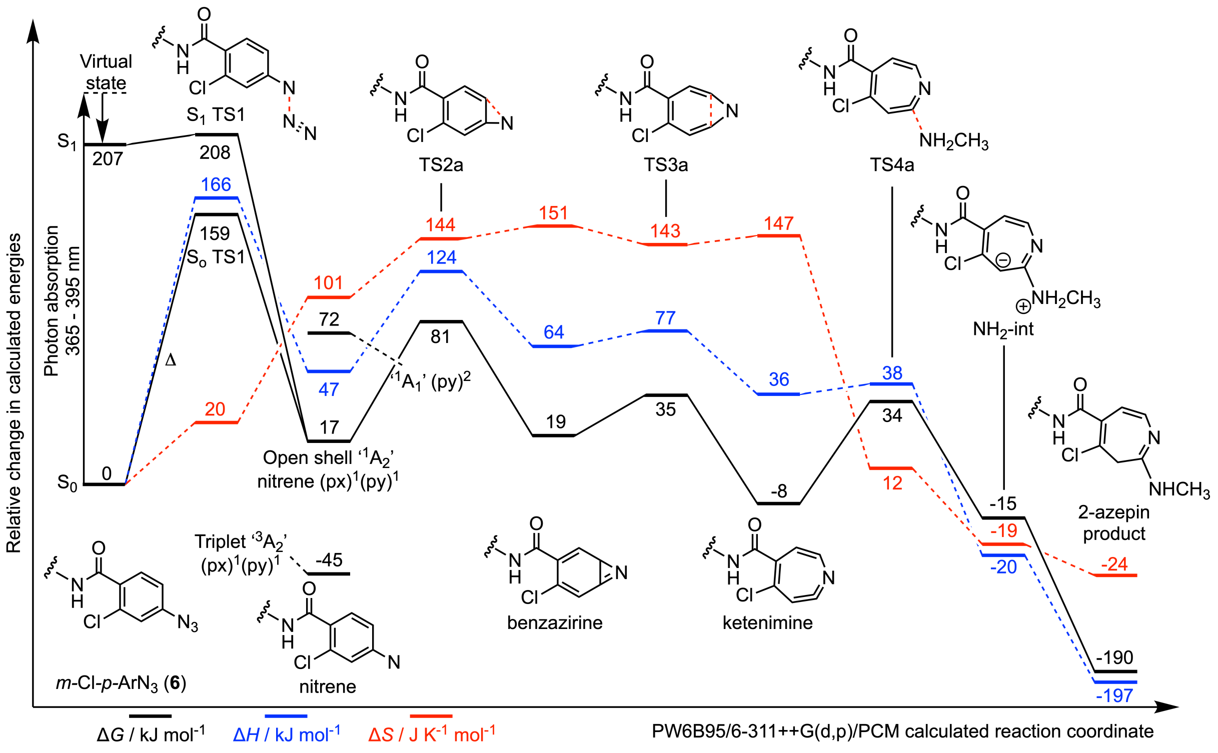

Figure S263. DFT calculated (PW6B95/6-311++G(d,p)/PCM) reaction coordinate showing the reaction of *ortho*-Cl-ArN3 (model **6** pathway b) with methylamine.

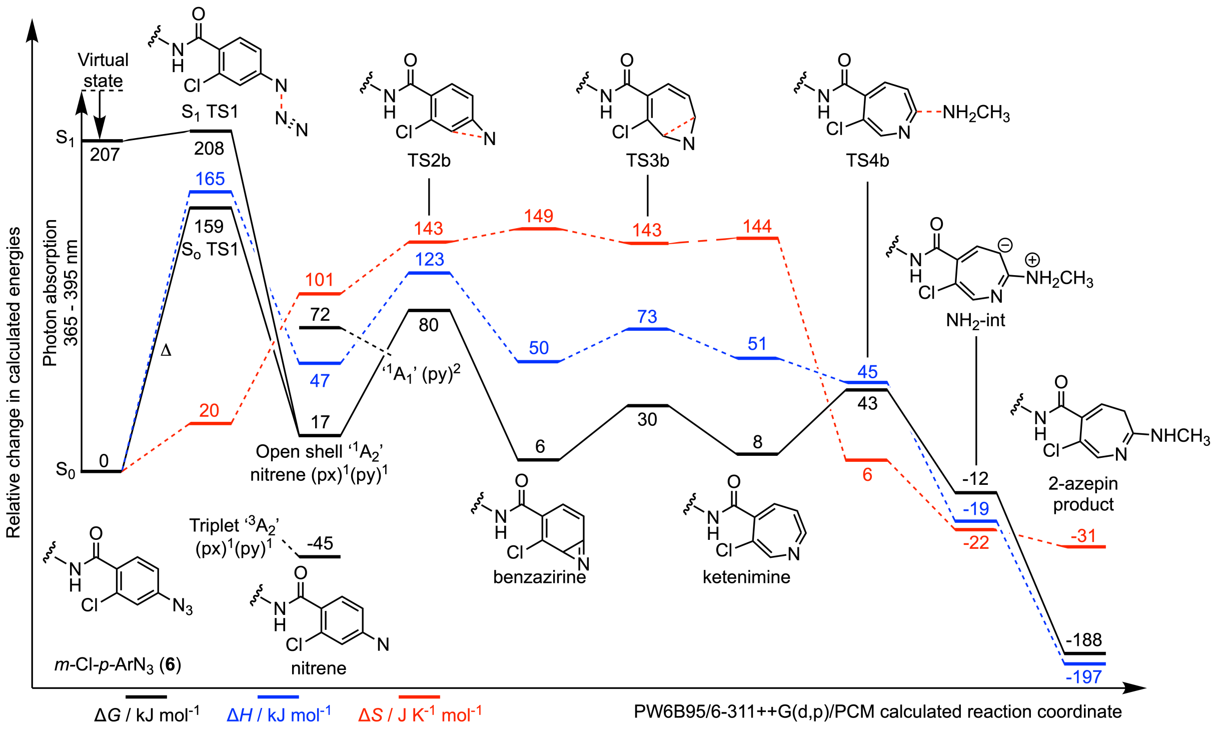

Figure S264. DFT calculated (PW6B95/6-311++G(d,p)/PCM) reaction coordinate showing the reaction of *ortho*-NO_2-ArN_3 (model **7** pathway a) with methylamine.

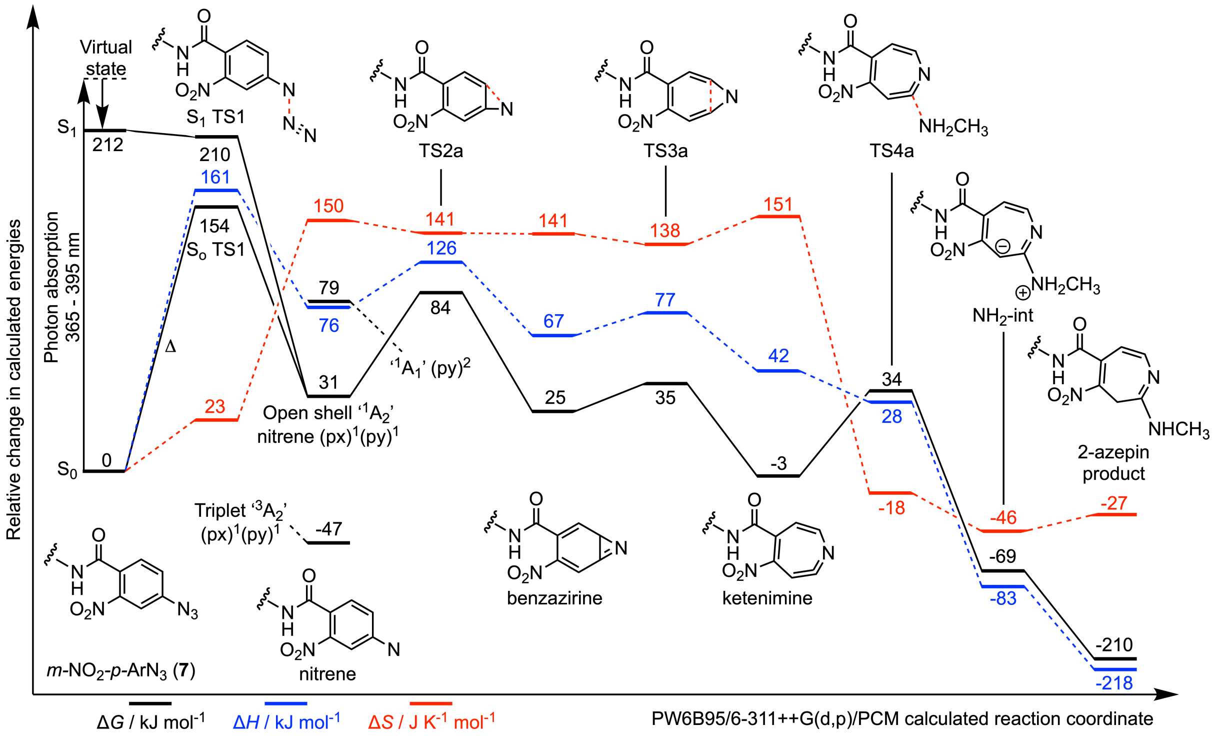

Figure S265. DFT calculated (PW6B95/6-311++G(d,p)/PCM) reaction coordinate showing the reaction of *ortho*-NO_2-ArN_3 (model **7** pathway b) with methylamine.

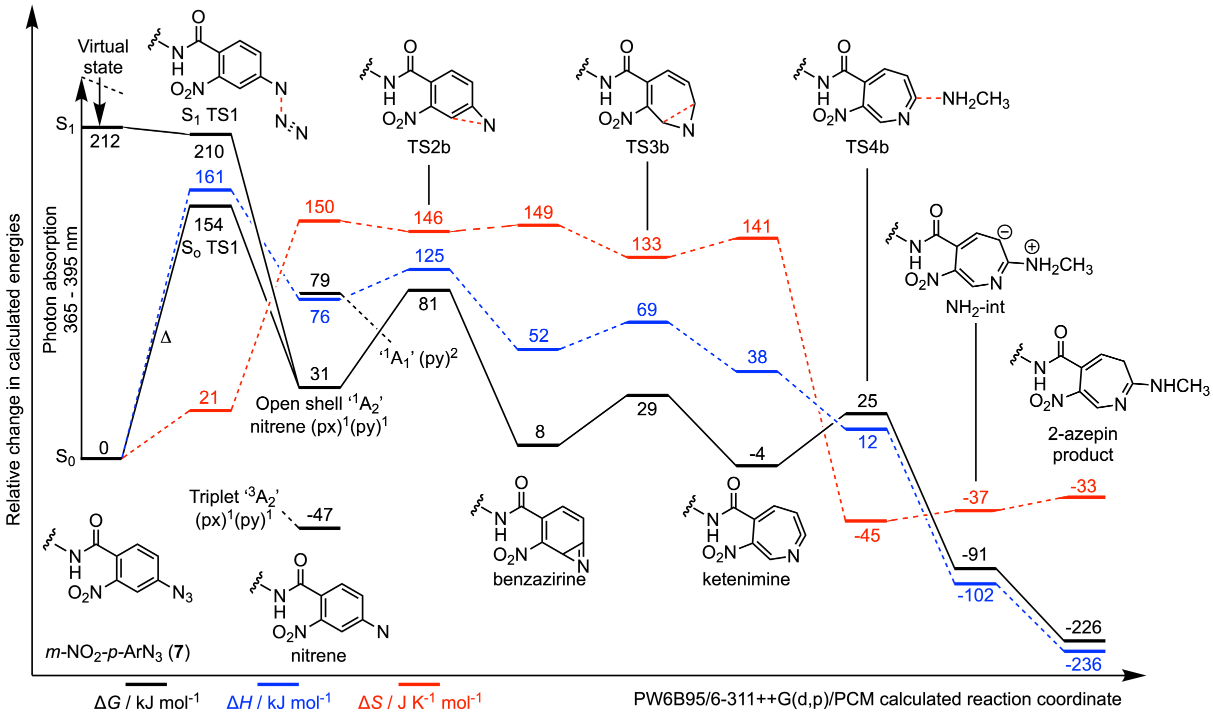

Figure S266. DFT calculated (PW6B95/6-311++G(d,p)/PCM) reaction coordinate showing the reaction of 4F-*p*-ArN3 (model **8**) with methylamine.

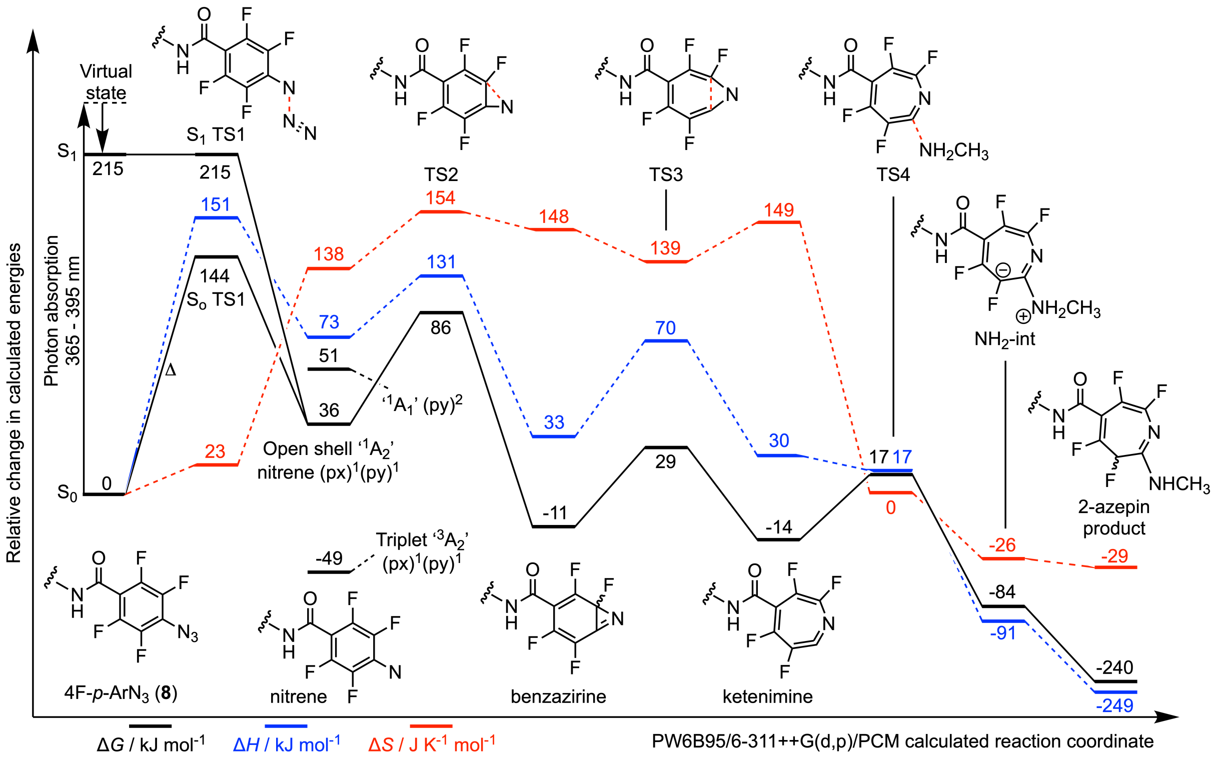

Figure S267. DFT calculated (PW6B95/6-311++G(d,p)/PCM) reaction coordinate showing the reaction of 2,4-pyridyl-ArN3 (model **9** pathway a) with methylamine.

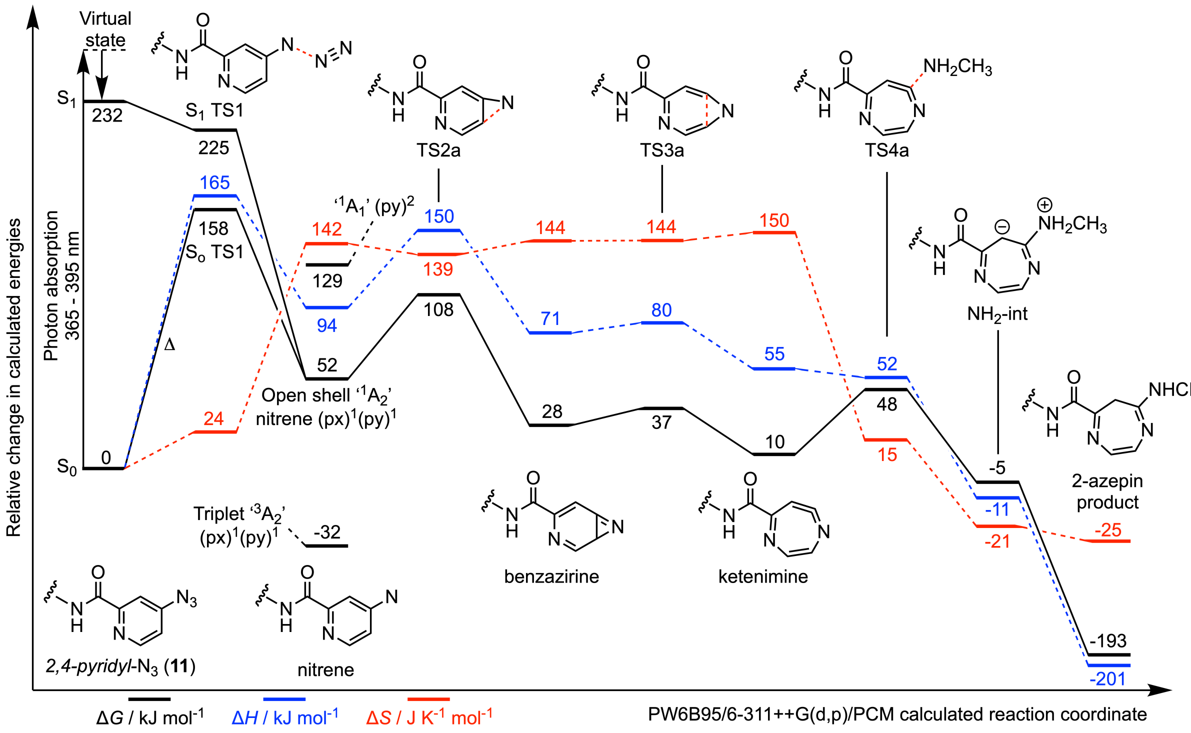

Figure S268. DFT calculated (PW6B95/6-311++G(d,p)/PCM) reaction coordinate showing the reaction of 2,4-pyridyl-ArN3 (model **9** pathway b) with methylamine.

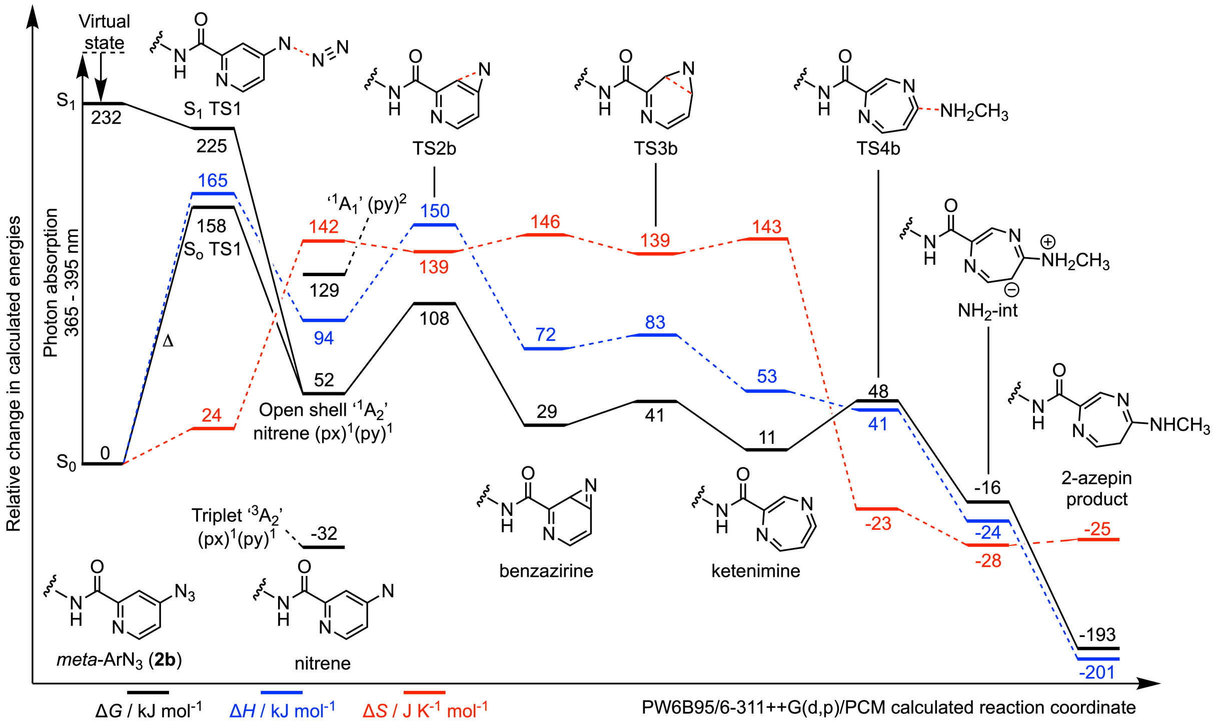

Figure S269. DFT calculated (PW6B95/6-311++G(d,p)/PCM) reaction coordinate showing the reaction of 2,3-naphthyl-ArN$_3$ (model **10** pathway a) with methylamine.

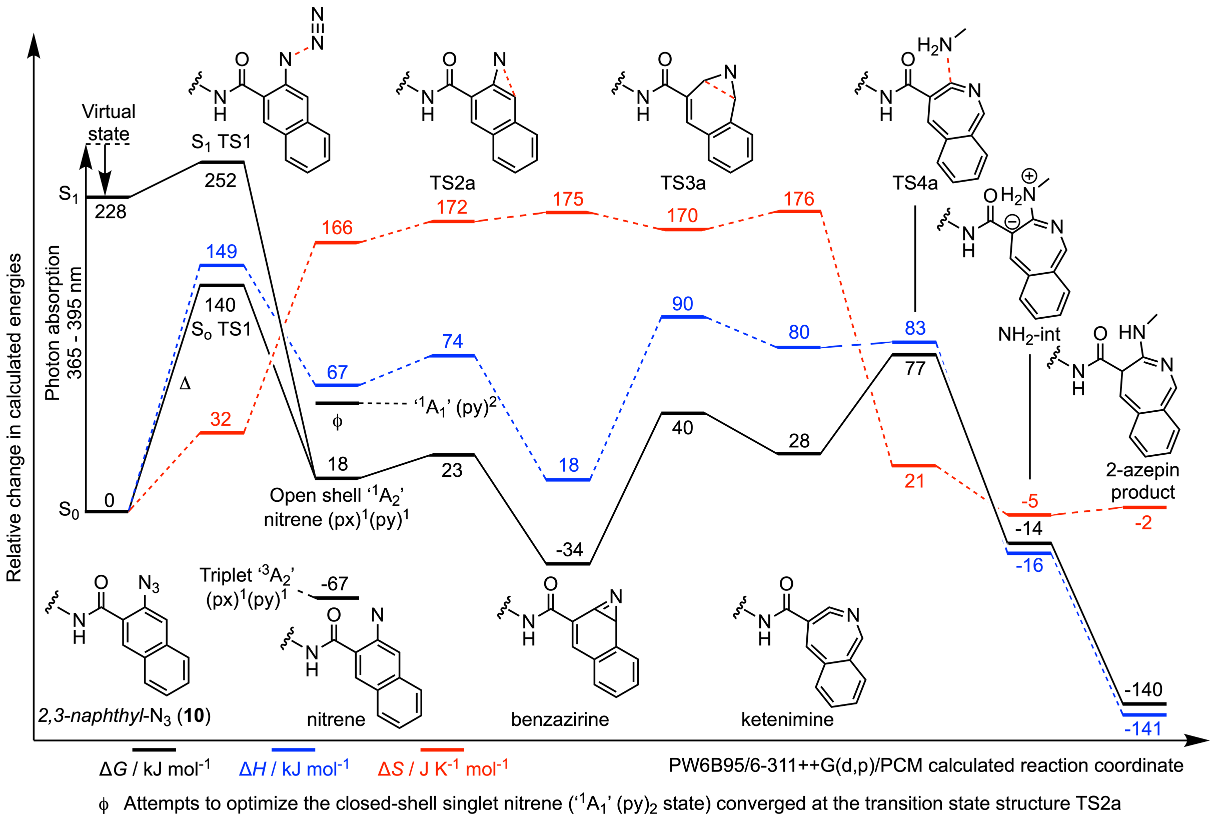

Figure S270. DFT calculated (PW6B95/6-311++G(d,p)/PCM) reaction coordinate showing the reaction of 2,3-naphthyl-ArN$_3$ (model **10** pathway b) with methylamine.

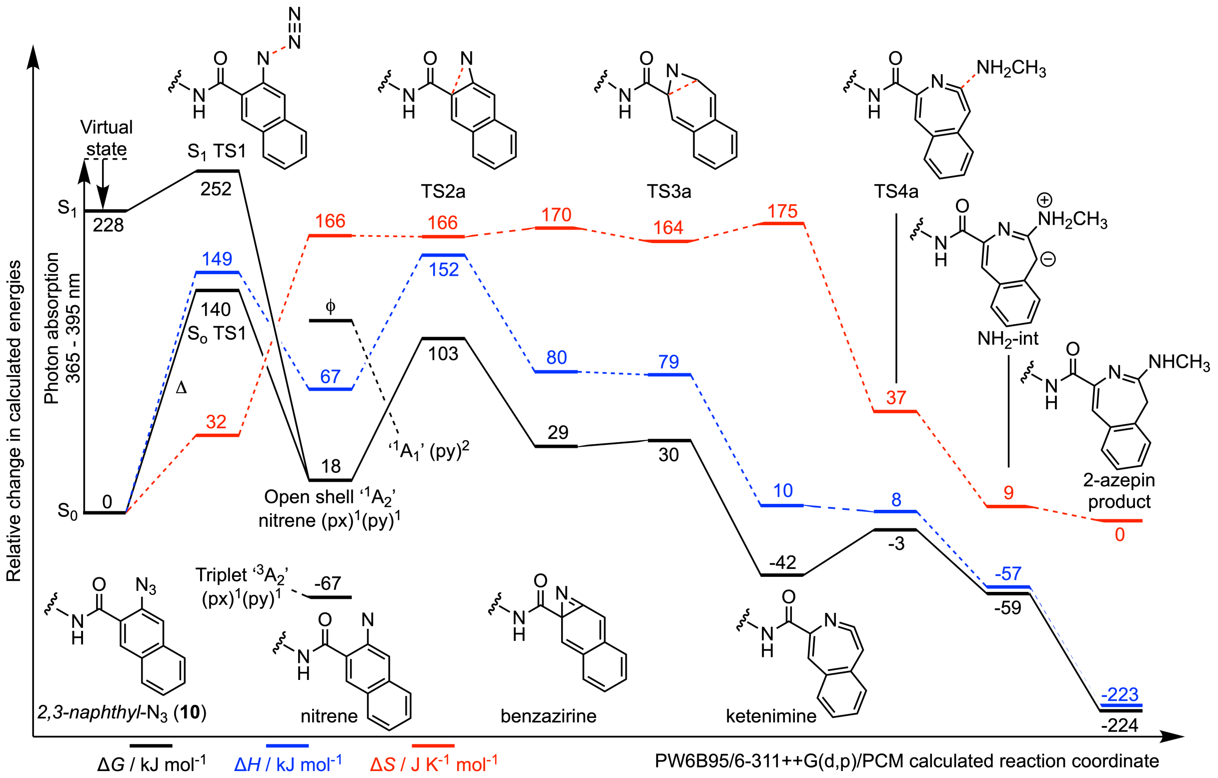

ϕ Attempts to optimize the closed-shell singlet nitrene ('1A1' (py)2 state) converged at the transition state structure TS2a

Figure S271. DFT calculated (PW6B95/6-311++G(d,p)/PCM) reaction coordinate showing the reaction of 2,6-naphthyl-ArN3 (model **11** pathway a) with methylamine.

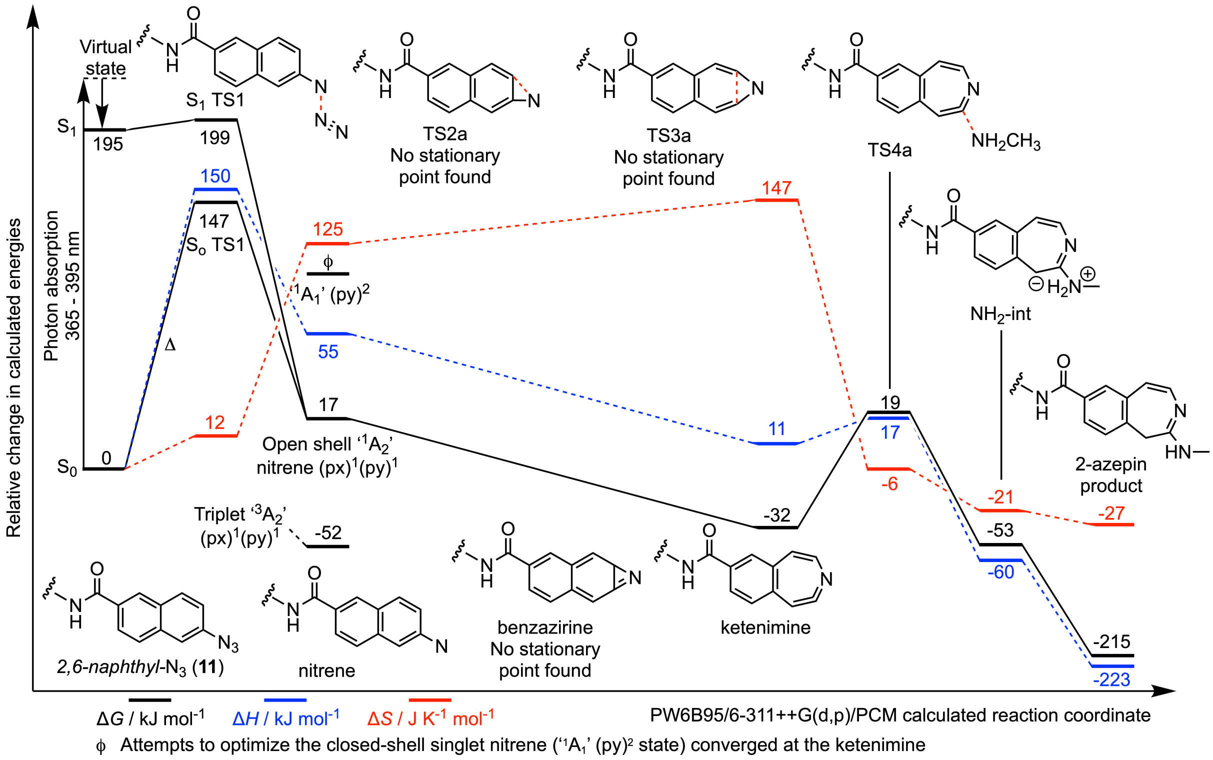

Figure S272. DFT calculated (PW6B95/6-311++G(d,p)/PCM) reaction coordinate showing the reaction of 2,6-naphthyl-ArN$_3$ (model **11** pathway b) with methylamine.

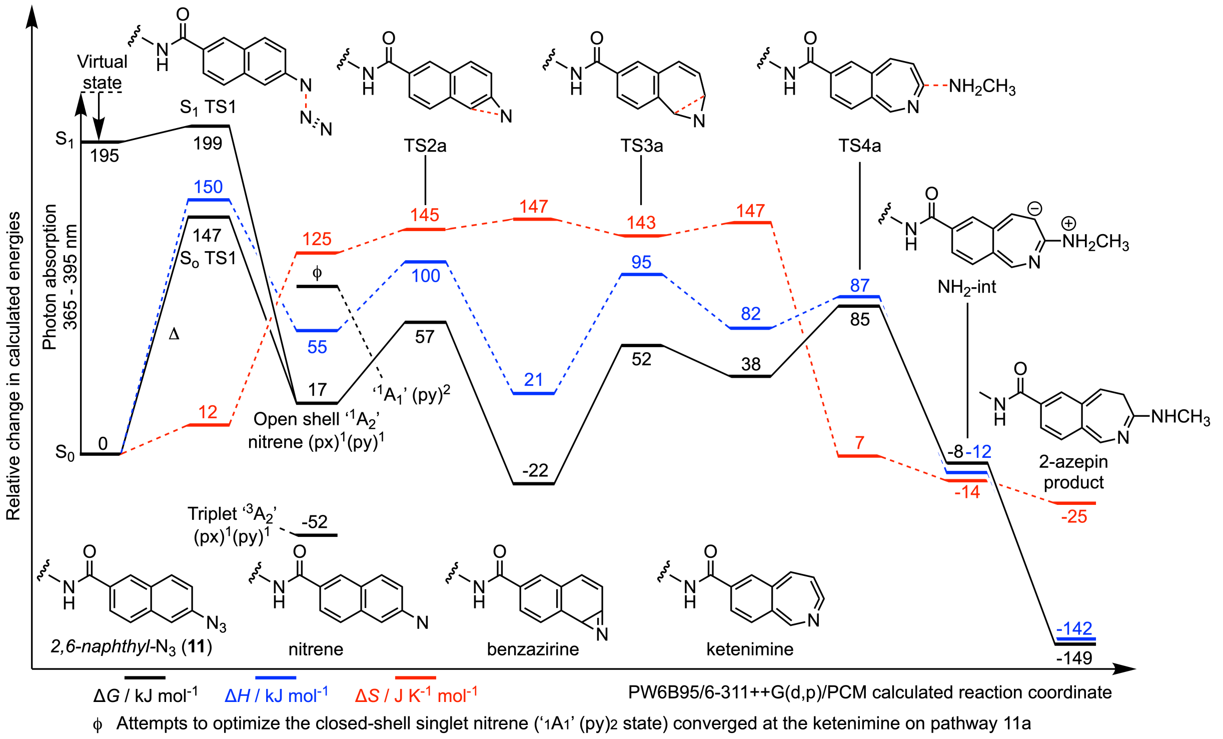

Table S7. Calculated energetics relative to the starting materials using the PW6B95/6-311++G(d,p) methodology with a PCM (water) solvation model for the C-H abstraction and bond insertion of nitrenes into the C-H bonds of model alanine and methionine.

	**Singlet $^1TS_{CH(abs)}$**			**Triplet $^3TS_{CH(abs)}$**			**C-H insertion product**		
Model compound	ΔG / kJ mol^{-1}	ΔH / kJ mol^{-1}	ΔS / J K^{-1} mol^{-1}	ΔG / kJ mol^{-1}	ΔH / kJ mol^{-1}	ΔS / J K^{-1} mol^{-1}	ΔG / kJ mol^{-1}	ΔH / kJ mol^{-1}	ΔS / J K^{-1} mol^{-1}
**Ala-Ha**									
**1**	73.3	68.8	-15.1	49.2	46.7	-8.1	-237.2	-255.3	-60.8
**5**	88.67	85.60	-10.29	67.80	64.77	-10.16	-223.31	-241.08	-59.60
**6**	81.85	71.27	-35.48	54.48	52.52	-6.57	-240.25	-258.09	-59.85
**7**	79.51	71.92	-25.46	55.73	50.40	-17.88	-246.91	-265.48	-62.28
**8**	72.00	61.16	-36.37	45.02	40.33	-15.74	-248.45	-270.00	-72.28
**9**	95.28	87.78	-25.18	70.23	64.59	-18.93	-246.42	-263.80	-58.30

Table S8. Calculated energetics relative to the starting materials using the PW6B95/6-311++G(d,p) methodology with a PCM (water) solvation model for the nucleophilic addition of various biologically relevant nucleophile models to the ketenimine produced after photoactivation of model compound **1**.

	**TS4 (Nucleophilic attack at the ketenimine)**				**Intermediate**			**Product**		
Nucleophile	ΔG / kJ mol^{-1}	ΔH / kJ mol^{-1}	ΔS / J K^{-1} mol^{-1}	Imaginary frequency / cm^{-1}	ΔG / kJ mol^{-1}	ΔH / kJ mol^{-1}	ΔS / J K^{-1} mol^{-1}	ΔG / kJ mol^{-1}	ΔH / kJ mol^{-1}	ΔS / J K^{-1} mol^{-1}
**$MeNH_2$**	47.7	48.4	2.4	-73.9	2.8	-5.1	-26.5	-176.1	-185.1	-30.2
**$CH_3CO_2^-$**	55.6	50.0	-18.8	-151.7	-4.5	-13.7	-30.8	-86.1	-97.1	-36.7
**MeOH**	105.0	98.3	-22.7	-96.2	Dissociates			-145.4	-156.6	-37.8
**MeS^-**	163.6	169.2	18.8	-144.6	Dissociates			-135.7	-146.2	-35.2
**Tyr-OH (*ortho*-C)**	134.8	129.9	-16.4	-374.0117	129.2	123.0	-20.6	-166.1	-175.7	-32.0
**Tyr-O⁻ (*ortho*-C)**	198.8	207.6	29.5	-251.9668	140.2	146.6	21.6	-166.1	-175.7	-32.0
**Tyr-O⁻ (phenolate)**	173.0	187.4	48.3	-82.3	63.1	74.5	38.3	-128.4	-127.8	1.9
**His [3+2]cycloaddition**	103.7	93.8	-33.1	-344.4	No stable intermediate			22.9	5.2	-59.2
**His (C-2 attack)**	108.3	100.9	-24.6	-403.0	101.6	94.9	-22.5	-141.9	-152.4	-35.3
**Water H_2O**	105.7	130.4	82.8	-236.8	Dissociates	o	o	-137.7	-116.1	72.5
**Hydroxide HO^-**	86.2	115.4	98.1	-129.7	-109.3	-84.1	84.8	-137.7	-116.1	72.5

Table S9. Calculated energetics relative to the starting materials using the PW6B95/6-311++G(d,p) methodology with a PCM (water) solvation model for the C-H abstraction transition state and product formed between the excited triplet ketyl biradical of benzophenone (BP) and selected amino acid model compounds.

	$^3TS_{Habs}$			Product		
Substrate	ΔG / kJ mol^{-1}	ΔH / kJ mol^{-1}	ΔS / J K^{-1} mol^{-1}	ΔG / kJ mol^{-1}	ΔH / kJ mol^{-1}	ΔS / J K^{-1} mol^{-1}
**Ala-Hα**	353.6	299.0	-183.2	120.2	53.8	-222.8
**Ala-Hβ**	353.9	309.4	-149.5	86.4	29.7	-190.4
**Phe-Hα**	354.2	303.7	-169.4	138.2	74.6	-213.2
**Phe-Hβ**	347.7	285.4	-208.9	82.9	21.0	-207.5
**Ser-Hα**	360.1	312.8	-158.6	141.8	81.7	-201.7
**Ser-OH**	362.7	315.9	-157.0	77.3	22.9	-182.3
**Cys-Hα**	347.6	296.2	-172.5	132.1	66.5	-220.0
**Cys-Hβ**	332.8	282.9	-167.4	83.3	21.4	-207.5
**Cys-SH**	-	-	-	67.7	19.0	-163.4
**Cys-Hβ-SSMe**	281.8	333.3	282.3	30.8	93.3	28.9
**Asp-Hα**	327.7	279.7	-161.1	216.1	172.7	-145.4
**Asp-Hβ**	343.6	298.7	-150.8			
**Glu-Hα**	329.4	275.2	-181.9	109.1	40.1	-231.4
**Glu-Hγ**	329.5	282.2	-158.7	96.1	34.2	-207.9
**Lys-Hα**	349.3	295.6	-180.2	132.1	65.1	-224.7
**Lys-NH**	332.8	294.8	-127.6	114.1	56.9	-192.0
**Met-Hα**	345.9	292.8	-178.0	128.0	61.8	-222.3
**Met-Hγ**	317.6	261.3	-188.9	104.9	35.0	-234.7
**Met-Hε**	317.7	276.3	-138.8	74.5	8.1	-222.9
**Tyr-Hα**	329.2	281.7	-159.1	139.4	87.6	-173.6
**Tyr-Hβ**	330.4	281.8	-163.0	139.8	84.1	-186.8
**Water**	353.6	338.6	-50.6	56.7	39.0	-59.4

Table S10. Calculated energetics relative to the starting materials using the PW6B95/6-311++G(d,p) methodology with a PCM (water) solvation model for the formation of different carbene electronic states, and the associated C-H abstraction transition states and products formed between model diazirine 13 and selected amino acid model compounds.

	Carbene			TS_{CB}			Product		
Substrate	ΔG / kJ mol^{-1}	ΔH / kJ mol^{-1}	ΔS / J K^{-1} mol^{-1}	ΔG / kJ mol^{-1}	ΔH / kJ mol^{-1}	ΔS / J K^{-1} mol^{-1}	ΔG / kJ mol^{-1}	ΔH / kJ mol^{-1}	ΔS / J K^{-1} mol^{-1}
**Ala-Hα**									
**Carbene (closed shell singlet)**	3.41	52.13	163.40	71.21	58.12	-43.91	-247.48	-269.28	-73.12
**Carbene (open shell singlet)**	1.92	50.51	162.95	68.64	56.69	-40.08	-247.48	-269.28	-73.12
**Carbene (open shell triplet)**	-10.23	42.96	178.38	45.22	42.60	-8.78	-247.48	-269.28	-73.12
Substate reactions with triplet carbene									
**Ala-Hα**	-10.23	42.96	178.38	45.22	42.60	-8.78	-247.48	-269.28	-73.12
**Ala-Hβ**	-10.23	42.96	178.38	92.40	86.12	-21.06	-268.86	-288.78	-66.81
**Phe-Hα**	-10.23	42.96	178.38	45.58	44.14	-4.83	-253.65	-289.70	-120.91
**Phe-Hβ**	-10.23	42.96	178.38	78.50	70.29	-27.53	-235.97	-257.19	-71.19
**Ser-Hα**	-10.23	42.96	178.38	56.92	59.15	7.49	-239.38	-254.80	-51.71
**Ser-OH**	-10.23	42.96	178.38	69.58	75.10	18.52	-221.73	-233.71	-40.18
**Cys-Hα**	-10.23	42.96	178.38	46.26	42.59	-12.30	-248.86	-270.13	-71.33
**Cys-Hβ**	-10.23	42.96	178.38	57.37	58.98	5.41	-238.10	-251.84	-46.07
**Cys-SH**	-10.23	42.96	178.38	49.89	28.35	-72.24	-270.28	-275.96	-19.06
**Cys-Hβ-SSMe**	-10.23	42.96	178.38	57.96	59.10	3.85	-244.50	-258.78	-47.87
**Asp-Hα**	-10.23	42.96	178.38	56.32	58.82	8.40	-243.16	-260.05	-56.63
**Asp-Hβ**	-10.23	42.96	178.38	83.37	76.37	-23.47	-230.21	-244.79	-48.91
**Glu-Hα**	-10.23	42.96	178.38	44.03	40.97	-10.28	-252.72	-271.27	-62.21
**Glu-Hγ**	-10.23	42.96	178.38	28.85	28.16	-2.34	-279.37	-295.70	-54.77
**Lys-Hα**	-10.23	42.96	178.38	49.83	43.91	-19.85	-249.37	-269.18	-66.45
**Lys-NH**	-10.23	42.96	178.38	87.15	93.24	20.44	-206.48	-217.29	-36.25
**Met-Hα**	-10.23	42.96	178.38	45.92	40.68	-17.59	-258.25	-532.36	-919.37
**Met-Hγ**	-10.23	42.96	178.38	43.19	41.94	-4.19	-276.19	-301.63	-85.34
**Met-Hε**	-10.23	42.96	178.38	57.12	35.46	-72.65	-271.96	-288.19	-54.43
**Tyr-Hα**	-10.23	42.96	178.38	64.11	56.84	-24.38	-255.42	-269.21	-46.27
**Tyr-Hβ**	-10.23	42.96	178.38	64.08	56.84	-24.29	-254.88	-269.21	-48.07
**Water**	-10.23	42.96	178.38	59.94	98.16	128.19	-266.49	-239.01	92.17

Table S11. Calculated energetics relative to the starting materials using the PW6B95/6-311++G(d,p) methodology with a PCM (water) solvation model for the nucleophilic attack at the nitrile imine formed between model tetrazole 14 and different biologically relevant nucleophiles.

	TS_{Nuc}			Intermediate-1			Intermediate-2		
Substrate	ΔG / kJ mol^{-1}	ΔH / kJ mol^{-1}	ΔS / J K^{-1} mol^{-1}	ΔG / kJ mol^{-1}	ΔH / kJ mol^{-1}	ΔS / J K^{-1} mol^{-1}	ΔG / kJ mol^{-1}	ΔH / kJ mol^{-1}	ΔS / J K^{-1} mol^{-1}
**Pathway c**									
**$CH_3CO_2^-$**	62.8	69.0	20.9	-15.2	-18.3	-537.7			
**HO^-**	32.6	48.0	51.6	-132.5	-123.7	29.3	-298.6	-295.9	9.0
**MeS^-**	44.4	57.9	45.2	-73.6	-69.8	12.9			
**Pathway d**									
**$MeNH_2$**	53.4	58.9	18.7	-21.2	-23.8	-8.6			
**MeSH**	131.1	133.4	7.8	129.6	131.1	5.2			
**MeOH**	83.6	90.8	24.0	70.6	70.4	-0.7			
**Water H_2O**	104.2	138.6	115.3	No stable intermediate			-298.6	-295.9	9.0

	TS_{Acyl}			Product		
Substrate	ΔG / kJ mol^{-1}	ΔH / kJ mol^{-1}	ΔS / J K^{-1} mol^{-1}	ΔG / kJ mol^{-1}	ΔH / kJ mol^{-1}	ΔS / J K^{-1} mol^{-1}
**Pathway c**						
**$CH_3CO_2^-$**	12.3	-2.9	-51.0	-215.9	-232.7	-56.3
**HO^-**				-345.0	-345.6	-2.3
**MeS^-**				-95.1	-94.9	0.8
**Pathway d**						
**$MeNH_2$**				-103.2	-103.1	0.5
**MeSH**				-90.4	-87.7	9.2
**MeOH**				-95.1	-94.9	0.8
**Water H_2O**				-345.0	-345.6	-2.3

References

1. Schröder, T., Gartner, M., Grab, T. & Bräse, S. A new azide staining reagent based on 'click chemistry'. *Org. Biomol. Chem.* **5**, 2767–2769 (2007).
2. Zanzonico, P. Routine Quality Control of Clinical Nuclear Medicine Instrumentation : A Brief Review *. *J Nucl Med* **49**, 1114–1132 (2019).
3. Frisch, M. J. *et al.* Gaussian16 (Revision A.03), Gaussian Inc. Wallingford CT. *Gaussian16 (Revision A.03)* (2016).
4. Ziegler, T. & Rank, A. On the Calculation of Multiplet Energies by the Hartree-Fock-Slater Method. *Theor. Chim. Acta* **43**, 261–271 (1977).
5. Guillou, A., Earley, D. F. & Holland, J. P. Light-activated protein-conjugation and 89Zr-radiolabelling with water-soluble desferrioxamine derivatives. *Chem. Eur. J.* **26**, 7185–7189 (2020).
6. Fay, R. & Holland, J. P. Tuning Tetrazole Photochemistry for Protein Ligation and Molecular Imaging. *Chem. - A Eur. J.* **27**, 4893–4897 (2021).
7. Patra, M., Klingler, S., Eichenberger, L. S. & Holland, J. P. Simultaneous Photoradiochemical Labeling of Antibodies for Immuno-Positron Emission Tomography. *iScience* **13**, 416–431 (2019).
